# Palliative and end-of-life care in people with and without intellectual disabilities in primary care: identification, survival time, and healthcare utilization

**DOI:** 10.1093/fampra/cmag010

**Published:** 2026-04-01

**Authors:** Freya Tyrer, Joanne Miksza, Francesco Zaccardi, Clare L Gillies, Kamlesh Khunti, Erica Borgstrom, Elizabeth Tilley, Irene Tuffrey-Wijne

**Affiliations:** Leicester Real World Evidence Unit, Diabetes Research Centre, University of Leicester, Leicester, LE5 4PW, United Kingdom; Observational & Pragmatic Research Institute, Norwich, NR11 6UN, United Kingdom; Leicester Real World Evidence Unit, Diabetes Research Centre, University of Leicester, Leicester, LE5 4PW, United Kingdom; Leicester Real World Evidence Unit, Diabetes Research Centre, University of Leicester, Leicester, LE5 4PW, United Kingdom; Leicester Real World Evidence Unit, Diabetes Research Centre, University of Leicester, Leicester, LE5 4PW, United Kingdom; Leicester Real World Evidence Unit, Diabetes Research Centre, University of Leicester, Leicester, LE5 4PW, United Kingdom; School of Health, Wellbeing & Social Care, The Open University, Milton Keynes, MK7 6AA, United Kingdom; School of Health, Wellbeing & Social Care, The Open University, Milton Keynes, MK7 6AA, United Kingdom; Faculty of Health, Science, Social Care & Education, Kingston University, Kingston-upon-Thames, KT2 7LB, United Kingdom

**Keywords:** intellectual disabilities, end-of-life, palliative, primary care, comorbidities, survival, health utilization

## Abstract

**Background:**

Evidence on end-of-life care (EOLC) provision for people with intellectual disabilities in primary care is limited.

**Methods:**

A retrospective cohort study of adults (18+ years) who died between 1 January 2010 and 31 March 2021 from linked Clinical Practice Research Datalink in England. Age- and gender-adjusted prevalence of comorbidities for people with and without IDs was compared by EOLC record assignment. Survival time and healthcare utilization after the first EOLC record was compared by ID status. Underlying cause of death was investigated.

**Results:**

Of 1.1 million adults identified, 2147 (21.8%) with IDs and 313 847 (28.4%) without IDs (unadjusted) had an EOLC record. Among the ID population, those with an EOLC record had disproportionately higher prevalence of dysphagia [+6.5 percentage points (95% CI: 4.9, 8.1)] and dementia [+5.2 percentage points (3.9,6.7)] than those without an EOLC record. Survival after the first EOLC record was shorter for people with IDs compared to those without IDs: 26% [absolute risk: 0.26 (0.24, 0.28)] vs 16% [absolute risk: 0.16 (0.16, 0.17)] died within 7 days. Primary care and hospital utilization rates were also lower [incidence rate ratio 0.96 (0.84, 0.97) and 0.42 (0.41, 0.44), respectively]. The most common cause of death in the ID population was cancer (38% of all deaths) followed by chromosomal conditions (11%).

**Conclusions:**

EOLC needs in primary care appear to be identified later for people with IDs. Their primary and hospital healthcare utilization patterns are also lower, despite having unique and complex health needs.

Key messages

- This is the first study of its kind to demonstrate that people with intellectual disabilities (IDs) have shorter survival than the general population after being assigned an end-of-life care (EOLC) record at their GP surgery.
- People with IDs with an EOLC record were disproportionately affected by dysphagia and dementia than people with IDs who did not have a EOLC record.
- After being assigned an end-of-life care record, the population with IDs had lower healthcare resource utilization (primary and general hospital care) and similar rates of specialist palliative care referrals compared to the population without IDs.
- Our findings suggest sub-optimal EOLC that restricts options available for EOLC planning. There is an urgent need to investigate ways in which identification of EOLC can be improved for this population so that appropriate provision can be instigated.

## Introduction

Around one million people living in England have intellectual disabilities (IDs), also known as learning disabilities in the UK, characterized by significant intellectual impairments and deficits in adaptive behaviours before the age of 18 years [1]. They experience significant health inequalities compared to those without IDs, including multiple long-term conditions, a shorter average life expectancy of between 12 and 23 years and a disproportionate burden of ‘avoidable’ deaths [2-4]. As they approach the ends of their lives, it is important that they are well supported, treated equitably and receive good quality end-of-life care (EOLC), defined by high-quality treatment, comfort, support, and dignity in the final months or year of life [5].

Evidence points to inequities in the provision of end-of-life care for this population [6-9], and failure to meet equality, diversity and inclusion principles in the UK [10]. Even in general healthcare settings, the combined effects of communication difficulties and poor health literacy mean that people with IDs may depend on others to notice and address their health concerns. When they approach the ends of their lives, these challenges mean that signs of ill health, frailty, and co-occurring long-term conditions can go unnoticed [11-14], leading to delays in identification of EOLC needs and sub-standard levels of care [15].

NHS England has set out its vision to improve EOLC throughout England via its NICE Guidelines (2019) and Ambitions Framework (2021) [6, 8]. Both policy initiatives place strong emphasis on the unpredictable care needs of people with IDs. The Ambitions Framework advocates evidence to inform service provision and training and also highlights six areas of improvement for achieving excellence: (i) individualized care; (ii) fair access to care; (iii) maximizing comfort and wellbeing; (iv) coordinated care; (v) preparedness of staff; and (vi) preparedness of communities. This study aims to contribute to the evidence by describing the current status of end-of-life care provision, identification of EOLC needs, and how these needs are met in the primary healthcare setting.

As primary care is the first point of contact for people with health concerns in England, general practitioners (GPs) play a pivotal role in identifying when people are nearing the ends of their lives, managing patients with EOLC needs, and taking steps to ensure appropriate EOL support [12]. However, with limited time with patients, workload pressures and reported lack of support in making clinical decisions for people with IDs [16, 17], it is unclear if palliative care needs can be adequately identified, managed, and signposted in the general practice setting. Theoretically, appropriate identification and referral to specialist palliative care services will improve the quality of care that people with IDs receive and reduce healthcare utilization through inappropriate hospital admissions and GP consultations. However, evidence on EOLC provision for people with IDs is extremely limited [18-21]. We urgently need a better understanding of EOLC for people with IDs in England to provide evidence for existing policy ambitions and make recommendations on how these can be achieved.

This study aimed to shed light on how EOLC is recorded and provided in primary care for people with and without IDs by investigating (i) recording of EOLC; (ii) comorbidities; (iii) survival time; (iv) healthcare utilization; and (v) causes of death.

## Methods

### Study design and participants

This study is reported in accordance with the REporting of studies Conducted using Observational Routinely collected health Data (RECORD) checklist [22] (Supplementary Table S1). We conducted a retrospective cohort study on adults living in England using primary care electronic health records from the Clinical Practice Research Datalink (CPRD) [23] Aurum and GOLD, linked (patient-level) with hospital episodes statistics (HES), 2019 index of multiple deprivation (IMD) information (based on patient postcode) [24] and deaths data (Office for National Statistics [ONS]) (Approved protocol ID: 24_004154). The CPRD is broadly representative of the population of the UK as a whole in relation to age, gender and ethnicity [23]. Approximately 75% of GP surgeries in England on the CPRD have data linkage. We followed the PECO (population, exposures, comparator and outcomes) framework [25] as below.

#### Population

The study population comprised adults (aged 18+ years) with and without IDs registered at an up-to-standard (using the CPRD's quality criteria) GP surgery from 1 January 2010 to 31 March 2021 (when last ONS deaths and HES data were available) who died while registered with the GP surgery.

#### Exposures

There were two exposure measures for this study: (i) presence of IDs and (ii) EOLC record (see Supplementary Table S2 for Read and SNOMED codes). ID codes were identified from previous research [26] and additional searches for genetic syndromes likely to be ID (e.g. Borjeson–Forssman–Lehmann syndrome, Langer–Giedion syndrome). The most common ID code was for an ID health assessment (32% of all ID cohort; see Supplementary Table S2 for more information). EOLC Read and SNOMED codes were based on NHS England's business rules [27]. EOLC records included diagnoses, treatments, referrals, and specific operational records (e.g. ‘Gold standards framework supportive care’) that referred to EOLC, terminal and/or palliative care. The most common EOLC code identified was ‘Palliative care’ (37% of overall sample—Supplementary Table S2). People with IDs entered the cohort (‘index date’) at the date of their first EOLC record and exited when they died. We only included individuals with a first ID diagnosis on or before their first EOLC record to prevent immortal time bias [28] (see Supplementary Figure S1).

#### Comparators

The comparator groups were individuals drawn from the same sample who did not have IDs (ever in their records prior to cohort entry). We also compared people with and without EOLC records by ID status (as a 4 × 4 comparison). People in the comparator population without EOLC records entered the cohort (‘index date’) 1-year prior to their death.

#### Outcomes

Outcome measures were underlying cause of death [as measured using International Classification of Diseases (ICD-10) codes—Supplementary Table S4], time to death, and healthcare utilization. Healthcare utilization was measured as rate of: face-to-face/telephone/video consultations with the GP or primary care nurse (Supplementary Table S5), hospital visits, and referrals to specialist palliative care services (Supplementary Table S6). Place of death used available ONS mortality data which categories place of death into (i) communal establishment (hospital, nursing home, residential home, hostel, school, religious establishment); (ii) home (usual residence—not a communal establishment); and (iii) other [29].

### Covariates

Covariates included in this analysis were age at index date; gender (‘indeterminate” gender was excluded for this analysis due to the small sample size); ethnicity (white; South Asian; Black African/Caribbean; mixed; other/missing [Supplementary Table S7]); alcohol use; smoking (current, ex-smoker and never); body mass index; IMD quintile; and co-existing health conditions (reported in the ‘exposures’ section).

Co-existing health conditions used both hospital episodes (ICD-10 codes) and GP Read and SNOMED codes (Supplementary Table S3) and were based on the Learning from Lives and Deaths (LeDeR) programme of work [3]. These included cancers; cardiovascular diseases (CVD); chronic kidney disease (CKD); degenerative disorders; dementia; diabetes; deep vein thrombosis (DVT) or pulmonary embolism; dysphagia; epilepsy; hypertension; mental ill-health (depression, anxiety, psychotic illness, bipolar disorders, attention-deficit hyperactivity disorders, eating disorders, and personality disorders); osteoporosis; respiratory disorders; and sensory impairments (visual/hearing). Autism was coded separately (outside mental ill health) and cerebral palsy (not traditionally classed as a degenerative condition) was also added as these are highly prevalent conditions in the ID population [30].

### Statistical methods

Differences in the characteristics of people with and without an EOLC record (aim i) were compared separately for people with and without IDs using non-parametric tests for significance (chi-squared and Mann–Whitney *U*-tests). To assess whether comorbidities were disproportionately higher or lower among those with an EOLC record (aim ii), we fitted logistic regression models by EOLC record status for each comorbidity (i.e. the outcome), also adjusting for age and gender. We calculated mean prevalence using the predicted probability of the specific comorbidity for each individual and difference in prevalence between people with and without an EOLC record (i.e. prevalence of comorbidity with EOLC record minus prevalence of comorbidity without EOLC record). Bootstrapping (2000 replications) was used to estimate the 95% confidence intervals (CI).

Among the population of individuals with an EOLC record, survival time for people with and without IDs after receiving their first EOLC flag (aim iii) was compared using time-to-event analyses standardized (‘standsurv” in Stata) for the overall age and gender profile. We fitted a flexible parametric model using time-varying effects (ID and age) and interaction terms (ID and age) to relax the proportionality assumption. For this analysis, individuals who lived beyond 2 years (730 days) after their first EOLC record were censored. Differential rates of healthcare utilization (number of GP/nurse consultations, hospital visits, palliative care referrals) were compared using Poisson regression (aim iv), adjusting for the overall age and gender profile and exposure time, which was fitted as a (log) offset. Causes of death (aim v) were compared for people with and without an EOLC record and by ID status. Causes of death (aim v) were plotted for people with and without an EOLC record.

## Results

### Study population

A data flow chart of the selection of patients for this study is shown in Supplementary Figure S1. In total, 1.1 million adults (9858 adults with IDs and 1 103 871 adults without) were identified who died while registered at the GP surgery between 1 January 2010 and 31 March 2021. Of these, 2147 (21.8%) of people with IDs and 313 847 (28.4%) of people without IDs had an EOLC record prior to death. Median length of GP registration prior to the first EOLC record (index date) was 14.7 years (interquartile range: 2.6–27.9 years).

Table 1 shows the characteristics of the study population by EOLC record status. Of people with an EOLC record, those with IDs were significantly younger than those without IDs (mean age difference of 15 years: 66 vs 81 years; *P* < .001), more likely to be male (50.2% vs 47%; *P* < .001), live in the most deprived IMD quintile (25.1% vs 19.6%; *P* < .001), and to have obesity (*P* < .001). They were less likely to smoke or drink alcohol (*P* < .001).

**Table 1:** Characteristics of study population by ID status and presence of EOLC record.

	EOLC record					No eOLC record				
	Intellectual disabilities		No intellectual disabilities		*P*-value	Intellectual disabilities		No intellectual disabilities		*P*-value
	*N*	(%)	*N*	(%)		*N*	(%)	*N*	(%)	
Total	2147	(100.0)	313 847	(100.0)	—	7711	(100.0)	790 024	(100.0)	—
**Sociodemographic variables^a^**										
Age (years) Median (IQR)	66.0	(57.0–76.0)	81.0	(71.0–88.0)	<.001	61.0	(49.0–71.0)	79.0	(68.0–86.0)	<.001
Gender: Male	1077	(50.2)	147 618	(47.0)	.004	4462	(57.9)	388 919	(49.2)	<.001
Ethnicity: White	1951	(94.9)	287 312	(95.5)	.76	6735	(93.7)	695 856	(94.8)	<.001
South Asian	38	(1.8)	5489	(1.8)		219	(3.1)	16 622	(2.3)	
Black African/Caribbean	31	(1.5)	4030	(1.3)		107	(1.5)	10 685	(1.6)	
Mixed	7	(0.3)	802	(0.3)		27	(0.4)	2012	(0.3)	
Other	29	(1.4)	3360	(1.1)		102	(1.4)	8922	(1.2)	
IMD quintile: 1—Least deprived	269	(12.5)	64 180	(20.5)	<.001	880	(11.4)	149 561	(19.0)	<.001
2	390	(18.2)	65 553	(20.9)		1342	(17.4)	157 280	(20.0)	
3	457	(21.3)	62 084	(19.8)		1580	(20.5)	155 280	(19.7)	
4	493	(23.0)	60 280	(19.2)		1768	(22.9)	158 817	(20.2)	
5—Most deprived	538	(25.1)	61 498	(19.6)		2137	(27.7)	166 734	(21.2)	
**Clinical measurements^a^**										
BMI: Underweight	173	(10.9)	20 026	(10.0)	<.001	424	(8.2)	33 473	(7.4)	<.001
Normal weight	592	(37.3)	81 828	(41.0)		1694	(32.7)	169 395	(37.5)	
Overweight	407	(25.6)	57 574	(28.9)		1379	(26.6)	133 647	(29.6)	
Obese	415	(26.1)	40 034	(20.1)		1685	(32.5)	115 055	(25.5)	
Blood pressure: Systolic	124.5	(19.5)	129.2	(19.0)	<.001	125.7	(19.1)	132.5	(18.5)	<.001
Diastolic	73.7	(11.3)	72.7	(11.4)	.14	73.9	(10.9)	73.5	(11.1)	.28
Smoking status: Non-smoker	1113	(66.1)	82 316	(36.4)	<.001	4404	(64.9)	255 146	(38.9)	<.001
Smoker	311	(18.5)	48 988	(21.7)		1507	(22.2)	152 956	(23.3)	
Ex-smoker	259	(15.4)	94 800	(41.9)		875	(12.9)	248 383	(37.8)	
Alcohol status: Non-drinker	343	(58.0)	19 181	(25.8)	<.001	1549	(48.4)	63 792	(22.2)	<.001
Drinker	237	(40.1)	52 660	(70.8)		1571	(49.1)	214 281	(74.6)	
Ex-drinker	11	(1.9)	2516	(3.4)		80	(2.5)	9078	(3.2)	
**Comorbidities**										
Multiple long-term conditions: ≥2 conditions below	901	(42.0)	141 875	(45.2)	.003	2348	(30.5)	285 968	(36.2)	<.001
Autism	21	(1.0)	22	(0.01)	<.001	77	(1.0)	129	(0.02)	<.001
Cancer	731	(34.0)	155 788	(49.6)	<.001	680	(8.8)	140 267	(17.8)	<.001
Cardiovascular diseases	223	(10.4)	47 411	(15.1)	<.001	1041	(13.5)	177 292	(22.4)	<.001
Cerebral palsy	15	(0.7)	18	(0.01)	<.001	101	(1.3)	106	(0.01)	<.001
Chronic kidney disease	123	(5.7)	27 093	(8.6)	<.001	526	(6.8)	83 963	(10.6)	<.001
Degenerative disorders	36	(1.7)	7533	(2.4)	.03	100	(1.3)	16 284	(2.1)	<.001
Dementia	265	(12.3)	38 358	(12.2)	.89	545	(7.1)	90 695	(11.5)	<.001
Diabetes	199	(9.3)	31 589	(10.1)	.24	823	(10.7)	89 516	(11.3)	.07
DVT/Pulmonary embolism	117	(5.4)	26 313	(8.4)	<.001	301	(3.9)	35 194	(4.5)	.02
Dysphagia	319	(14.9)	18 755	(6.0)	<.001	648	(8.4)	26 993	(3.4)	<.001
Epilepsy	308	(14.3)	4182	(1.3)	<.001	972	(12.6)	10 717	(1.4)	<.001
Hypertension	147	(6.8)	37 198	(11.9)	<.001	634	(8.2)	107 547	(13.6)	<.001
Mental ill-health (not autism)	171	(8.0)	22 634	(7.2)	.19	604	(7.8)	56 622	(7.2)	.02
Osteoporosis	61	(2.8)	13 082	(4.2)	.003	232	(3.0)	36 175	(4.6)	<.001
Respiratory disorder	153	(7.1)	30 636	(9.8)	<.001	546	(7.1)	85 764	(10.9)	<.001
Sensory impairment	239	(11.1)	19 906	(6.3)	<.001	749	(9.7)	47 657	(6.0)	<.001

^a^Missing data: ethnicity: *n* = 69 393; IMD quintile: *n* = 2608; BMI: *n* = 455 928; systolic and diastolic blood pressure: *n* = 942 424; smoking status: *n* = 222 671; alcohol consumption: *n* = 748 430

IQR: interquartile range.

Of people with an EOLC record (regardless of ID status), multiple long-term conditions (≥2 chronic conditions) were high—present in >40%. The profile of nearly all comorbidities was very different for those with IDs compared to those without IDs. The ID population had higher prevalence of dysphagia, epilepsy, sensory impairments, cerebral palsy, and autism compared to people without IDs (*P* < .001 for all) and lower prevalence of cancers, CVD, CKD, degenerative conditions, DVT/pulmonary embolism, hypertension, osteoporosis, and respiratory conditions (*P* < .001).

**Figure 1:**
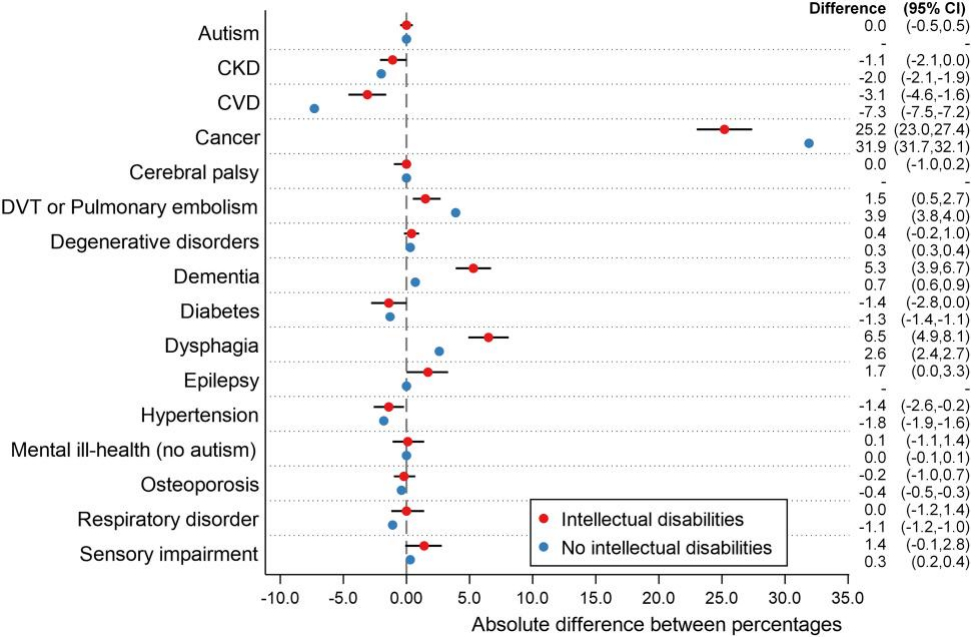
Age- and gender-adjusted absolute differences between the percentage of individuals with an EOLC record and those without an EOLC record.

Age- and gender-adjusted differences in the prevalence of comorbidities by EOLC status (prevalence of the condition in those with an EOLC record minus prevalence in those without) is shown in Fig. 1. Compared to people with IDs without an EOLC record, people with IDs and an EOLC record were more likely to have cancer [+25.2 percentage points (95% CI: 23.0, 27.4)], dysphagia [+6.5 percentage points (4.9, 8.1)], dementia [+5.3 percentage points (3.9, 6.7)], and DVT/pulmonary embolism [+1.5 percentage points (0.5, 2.7)]. The same comorbidities were more prevalent for people without IDs and an EOLC record, but differential cancer and DVT/pulmonary embolism rates were higher [+31.9 percentage points (31.7, 32.7) and +3.9 percentage points (3.8, 4.0), respectively) and lower for dysphagia [+2.6 percentage points (2.4, 2.7)] and dementia [+0.7 percentage points (0.6, 0.9)].

Conversely, people with IDs with an EOLC record (compared to those without one) were less likely to have CVD [−3.1 percentage points (−4.6, −1.6)] and hypertension [−1.4 percentage points (−2.6, −2.0)]. These effects were greater among those without IDs (CVD: −7.3; hypertension: −1.8): CKD, diabetes and respiratory disorders were also less prevalent (−1.1 to −2.0 percentage point differences).

#### Time to death

**Figure 2:**
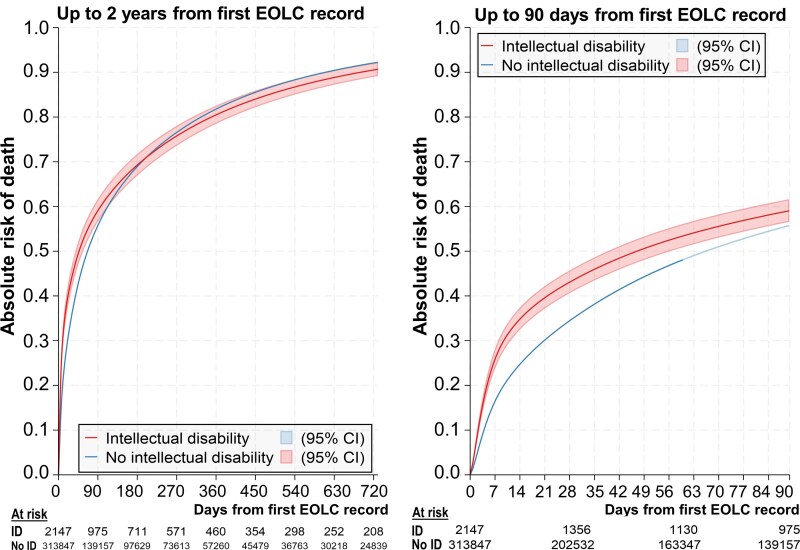
Survival (standardized for overall age and gender) after first EOLC record among adults with and without intellectual disabilities.

Figure 2 shows the (overall age and gender) standardized differences in survival after individuals' first EOLC record for up to 90 days and 2 years from EOLC record (Supplementary Figure S2 shows the unadjusted estimates). People with IDs were more likely to die in the first 90 days after receiving an EOLC record than those without IDs. 26% of people with IDs died within 7 days of receiving an EOLC record [risk of death: 0.26; (95% CI): 0.24, 0.28] compared with 16% in people without IDs (risk of death: 0.16; 0.16, 0.17). After the first 90 days, the two groups had similar survival patterns: most of the population with and without IDs (81% vs 82%) had died by one year and more than 90% had died within two years of having an EOLC record (91% vs 92%).

#### Healthcare utilization

The crude rate of primary care consultation rates after receiving the first EOLC record was similar for people with and without IDs, averaging 1.5 consultations per month for both groups. After adjustment for age and gender, people with IDs had 4% lower primary care consultation rates [IRR: 0.96 (0.84, 0.97); *P* < .001] and about half the number of hospitalizations as those without IDs [IRR: 0.42 (0.41, 0.44); *P* < .001) (Table 2). Specialist palliative care referral rates were generally low, with an average of 0.03 per month in both groups and tended to occur close to the first EOLC record (Supplementary Figure S3 shows the crude monthly rates): only 16% (*n* = 343) and 15% (*n* = 1804) of individuals with and without IDs, respectively, were referred at least once on or after being assigned an EOLC record (*P* = .48 for difference).

**Table 2:** Crude consultations, hospitalization and referral rates per month and age–gender-adjusted incidence rate ratio of ID to no intellectual disabilities after receipt of EOLC record.

	Intellectual disabilities		Crude rate per month (95% CI)	No intellectual disabilities		Crude rate per month (95% CI)	Incidence rate ratio^a^ (95% CI)	*P*-value
	*N*	PY		*N*	PY			
**Face-to-face/video/telephone consultations**								
All (GP or practice nurse)	19 866	13 412.9	1.48 (1.46,1.50)	2 628 587	1 807 313.0	1.45 (1.45,1.46)	0.96 (0.84,0.97)	<.001
GP	18 444	13 412.9	1.38 (1.36,1.40)	2 417 493	1 807 313.0	1.34 (1.34,1.34)	0.96 (0.95,0.98)	<.001
Practice nurse	1422	13 412.9	0.11 (0.10,0.11)	211 094	1 807 313.0	0.12 (0.12,0.12)	0.90 (0.85,0.95)	<.001
**Hospitalizations**								
Admissions	3050	13 412.9	0.23 (0.22,0.24)	654 550	1 807 313.0	0.36 (0.36,0.36)	0.42 (0.41,0.44)	<.001
**Specialist palliative care referrals**								
All	404	13 412.9	0.03 (0.03,0.03)	58 453	1 807 313.0	0.03 (0.03,0.03)	0.95 (0.86,1.04)	.27

^a^Adjusted for age and gender

PY: person-years.

#### Place and cause of death

More people with IDs and an EOLC record died in communal establishments (including their “home” if they lived in a nursing/residential home) than those without IDs and an EOLC record [IDs vs no IDs: *n* = 1463 (68.1%) vs *n* = 206 665 (65.9%); *P* = .03], compared with dying at “home” (i.e. their own property; *n* = 663 (30.9%) vs *n* = 103 044 (32.8%)] or elsewhere [*n* = 21 (1.0%) vs *n* = 4135 (1.3%)].

**Figure 3:**
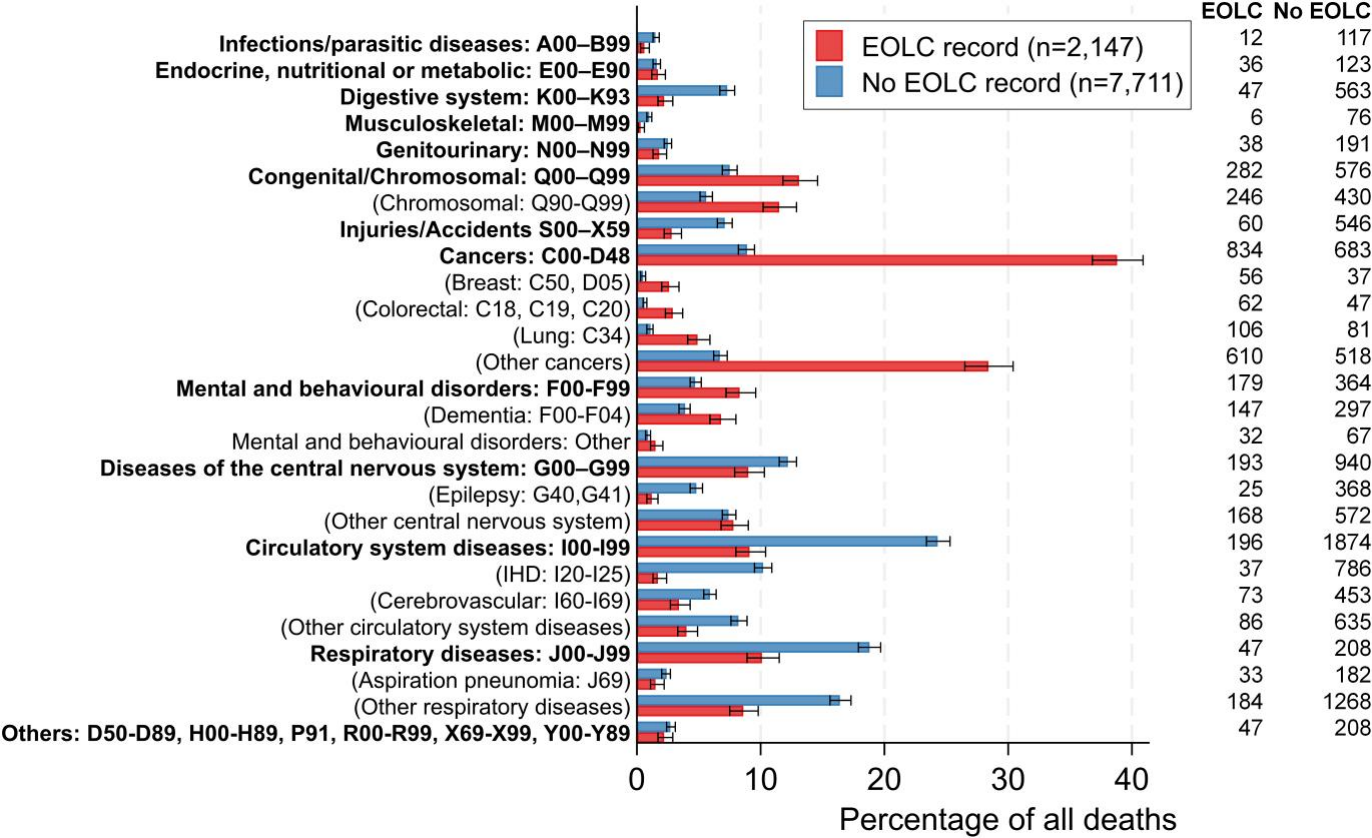
Underlying cause of death among individuals with intellectual disabilities: comparison between people with an EOLC record and those without. Error bars represent Wilson confidence intervals.

Figure 3 shows the causes of death for the population with IDs by EOLC status. Among those with IDs and an EOLC record, causes of death were dominated by cancers, making up 38.8% of all deaths compared with 8.9% of those with IDs who did not have an EOLC record (*P* < .001) (Fig. 3). Conversely, causes of death due to circulatory system diseases (9.1% vs 24.3%) and central nervous system diseases (8.9% vs 12.2%) were less prevalent (*P* < .001). Of note, 246 people with IDs and an EOLC record (11.4%; *n* = 241 [98.0%] Down syndrome) had an underlying cause of death of chromosomal conditions compared with 430 [5.6%; *n* = 391 (90.9%) Down syndrome] of those with IDs who did not have an EOLC record (*P* < .001).

## Discussion

In this large primary care population of more than 1.1 million individuals in England, we found that people with IDs were identified with EOLC needs closer to death than the rest of the population, with only 74% surviving beyond 7 days from their first notification, compared with 84% of those without IDs. As expected, people with IDs had a different comorbidity profile from those without IDs, but those with EOLC needs also had a disproportionate burden of dysphagia and dementia. Having been identified with EOLC needs, primary care consultations and hospitalizations were lower among people with IDs than those without IDs and specialist palliative care referrals were comparable. People with IDs and EOLC needs were more likely to die in communal care establishments, with the most common causes of death being cancers (38%) and chromosomal conditions (11%).

EOLC research remains a challenging area due to its sensitive nature, ethical considerations and complexity [31, 32]. Whilst we accept that not all deaths are predictable, it is a concern that more than one-quarter of people (26%) with IDs did not survive beyond the first week of receiving an EOLC record compared with less than one-fifth (16%) of people without IDs. These findings are consistent with a survey of 97 Dutch physicians which revealed that, when considering their last case of a patient with IDs, 20% did not foresee their death until the last week of life and 30% did not discuss palliative care until the last week [12]. In the UK, research has found that only 34% of deaths among the ID population are anticipated [14]. Lower survival rates after receiving an EOLC record may reflect delays in EOLC identification in this population such that their needs are only identified at a stage when they are deteriorating rapidly. However, they may also be a marker of poor healthcare quality whereby services are not able to meet the needs of people with IDs in the same way as other people so they die sooner. In either scenario, such a short timeframe is unlikely to be sufficient to manage symptoms and care effectively, or enable people to prepare for death. It is reassuring that, beyond the first 90 days of receiving an EOLC record, survival experience was similar for people with and without IDs, suggesting that timely identification of EOLC needs can lead to comparable outcomes.

People with IDs who had EOLC needs had a significantly higher prevalence of dysphagia and dementia in this study than those without EOLC needs. It seems likely that these conditions trigger an EOLC record in people with IDs as frailty is a common risk factor for both conditions [33, 34]. As most patients with EOLC needs are managed in primary care, we highlight the support of speech and language expertise to take account of co-existing dementia and dysphagia. It is not surprising that cancers dominated the causes of death reporting among people with IDs as cancers are common and have a prognostic trajectory that makes mortality predictions more reliable [35]. Circulatory system diseases, such as myocardial infarction and heart failure, tend to be acute which means that people have less time to plan for them. However, it is noteworthy that CVD among those with an EOLC record was one-third lower in the ID population (10% vs 15%), which could suggest barriers to symptom communication, such as chest pain. Whilst we also found that a greater proportion of people with IDs in this study died in a communal care establishment or hospital, we are unable to make inferences beyond this as people with IDs' normal residence may have been in a communal care establishment. We recommend further work to explore if preferences of where to die are addressed and met in this population.

A substantial proportion of individuals of people with IDs and an EOLC record had an underlying cause of death recorded of Down syndrome or another aetiological condition related to IDs. This proportion was around twice that (11% vs 6%) of the population of people with IDs who did not have an EOLC record. Although the reporting of genetic syndromes and “intellectual disability” as the underlying cause of death is not a new finding [36], it is believed to indicate uncertainty surrounding the death itself [37]. Such uncertainty seems unlikely to be a reason for this reporting as the person was known to be nearing the ends of their lives.

Hospitalizations and primary care consultations following first identification of EOLC needs were lower among people with IDs compared to the comparison group and specialist palliative care referrals were similar. These findings differ from studies among the general ID population where primary care consultation and hospitalization rates are generally higher than in those without IDs [38, 39]. This may reflect the provision of EOLC needs in specialist IDs services (we do not have data from secondary care), but it is equally possible that needs were not met or that reluctance to go into hospital prompted more emergency hospital admissions for which we did not have available data linkage. People with IDs may also have had longer hospital stays, which would equate to fewer hospital admissions per person. We urgently need more work to explore healthcare utilization and EOLC pathways for this group of individuals, particularly given the unique comorbidities identified in this client group which are likely to require specialized care strategies.

The CPRD has been found to be broadly representative of the population of the UK as a whole in relation to age, gender and ethnicity [23]. However, EOLC is a broad and complex discipline and we are limited to electronic health-related information, which precludes investigation into other relevant quality of care indicators, such as carer support and patient-reported outcome measures. Moreover, it is not always the GP that identifies EOLC needs in primary care; the wider primary care staff team, including administrators, are able to input notes in the patients' records [40]. Although people with IDs were less likely to have an EOLC record in this particular study population (21.8% vs 28.4%), we restricted to people who died, so missed people who lived longer and transferred to other GP surgeries. The prevalence of approximately 0.7% for IDs observed using our data are comparable to estimates across Europe and Australasia [26] but we note that people with mild intellectual impairments can be missed under a system that focuses on care need.

## Conclusions

In summary, our findings suggest that early identification of EOLC needs is sub-optimal for people with IDs and that referral rates to specialist palliative care services are low. There is an urgent need to investigate ways in which identification of EOLC need can be improved for this population so that appropriate and timely EOLC provision can be instigated. The substantial differences in the long-term conditions of people with IDs nearing the ends of their lives, including dysphagia and dementia, also highlight the need for individualized EOLC that is specially tailored to meet their unique needs. The benefits of timely identification of EOLC need and subsequent healthcare utilization, care provision and patient and carer experience need further investigation. For this, we require an in-depth exploration of EOLC service provision, quality and accessibility for people with IDs to ensure care models are both equitable and responsive to their complex needs.

## Supplementary Material

cmag010_Supplementary_Data

## Data Availability

Data may be obtained from a third party and are not publicly available. The data used for this study involve an extract from an established research database, the Clinical Practice Research Datalink (CPRD) with linked mortality data from the Office for National Statistics. The data controller for the CPRD is the Department of Health and Social Care.
