# Supplementary material for: Palliative and end-of-life care in people with and without intellectual disabilities in primary care: identification, survival time, and healthcare utilization
Source: Fam Pract. 2026 Apr 1;43(2):cmag010. doi: 10.1093/fampra/cmag010 (PMC13042290; doi:10.1093/fampra/cmag010)
Supplement: cmag010_Supplementary_Data [file cmag010_supplementary_data.pdf]

Palliative and end-of-life care in people with and without intellectual disabilities in primary care: identification, survival time and healthcare utilisation

Supplementary Material

Table S1: RECORD (Reporting of studies conducted using observational routinely-collected data) checklist *)

	Item No.	STROBE items	Location in manuscript where items are reported	RECORD items	Location in manuscript where items are reported
**Title and Abstract**					
	1	(a) Indicate the study's design with a commonly used term in the title or the abstract<br><br>(b) Provide in the abstract an informative and balanced summary of what was done and what was found	Retrospective cohort study listed in methods of abstract<br><br>See abstract	RECORD 1.1: The type of data used should be specified in the title or abstract. When possible, the name of the databases used should be included.<br><br>RECORD 1.2: If applicable, the geographic region and timeframe within which the study took place should be reported in the title or abstract.<br><br>RECORD 1.3: If linkage between databases was conducted for the study, this should be clearly stated in the title or abstract.	CPRD and linked databases listed in abstract<br><br>England listed in the abstract and dates of data collection<br><br>Linked data specified in the abstract
Background rationale	2	Explain the scientific background and rationale for the investigation being reported	See Introduction		
Objectives	3	State specific objectives, including any prespecified hypotheses	See Introduction		

Study Design	4	Present key elements of study design early in the paper	See Methods (Study design and participants)		
Setting	5	Describe the setting, locations, and relevant dates, including periods of recruitment, exposure, follow-up, and data collection	See Methods (Study design and participants, Population		
Participants	6	(a) *Cohort study* - Give the eligibility criteria, and the sources and methods of selection of participants. Describe methods of follow-up *Case-control study* - Give the eligibility criteria, and the sources and methods of case ascertainment and control selection. Give the rationale for the choice of cases and controls *Cross-sectional study* - Give the eligibility criteria, and the sources	See Population	RECORD 6.1: The methods of study population selection (such as codes or algorithms used to identify subjects) should be listed in detail. If this is not possible, an explanation should be provided.<br><br>RECORD 6.2: Any validation studies of the codes or algorithms used to select the population should be referenced. If validation was conducted for this study and not published elsewhere, detailed methods and results should be provided.<br><br>RECORD 6.3: If the study involved linkage of databases, consider use of a flow diagram or other graphical display to demonstrate the data linkage process, including the number of individuals with linked data at each stage.	All codes used in the study are described in the supplementary material<br><br>Cleaning algorithms, citations and sources of data are contained in the supplementary material (e.g. Table S3, BMI – Table S6). Frequency of codes are explored in more detail (e.g. EOLC codes in Table S2)<br><br>See Figure S1 for data flow diagram

		and methods of selection of participants<br><br>(a) *Cohort study* For matched studies, give matching criteria and number of exposed and unexposed *Case-control study* - For matched studies, give matching criteria and the number of controls per case			
Variables	7	Clearly define all outcomes, exposures, predictors, potential confounders, and effect modifiers. Give diagnostic criteria, if applicable.	See Methods	RECORD 7.1: A complete list of codes and algorithms used to classify exposures, outcomes, confounders, and effect modifiers should be provided. If these cannot be reported, an explanation should be provided.	See supplementary tables
Data sources/ measurement	8	For each variable of interest, give sources of data and details of methods of assessment (measurement).	See supplementary tables of codes and Methods		

		Describe comparability of assessment methods if there is more than one group	Where calculated data used (e.g. BMI), the same cleaning algorithms are used for people with and without intellectual disabilities		
Bias	9	Describe any efforts to address potential sources of bias	Median length of registration prior to EOLC record and interquartile range listed: 99% [98% intellectual disabilities] of individuals have ≥6 months look-back period prior to index date (supporting likelihood of first EOLC record). Diagnosis of intellectual disability must occur before EOLC record to mitigate immortal time bias		
Study size	10	Explain how the study size was arrived at	See Methods. All available individuals taken.		
Quantitative variables	11	Explain how quantitative variables were handled in the analyses. If applicable, describe which groupings	See Methods – groupings listed and codes provided in the supplementary material		

		were chosen, and why			
Statistical methods	12	(a) Describe all statistical methods, including those used to control for confounding<br>(b) Describe any methods used to examine subgroups and interactions<br>(c) Explain how missing data were addressed<br>(d) *Cohort study* - If applicable, explain how loss to follow-up was addressed *Case-control study* - If applicable, explain how matching of cases and controls was addressed *Cross-sectional study* - If applicable, describe analytical methods taking account of sampling strategy<br>(e) Describe any sensitivity	See Statistical Methods<br>See Statistical Methods<br>Missing data included as a separate category and referred to in the discussion<br>Only included people who died whilst registered at the relevant GP surgery. Right censoring not used.		

		analyses			
Data access and cleaning methods		-		RECORD 12.1: Authors should describe the extent to which the investigators had access to the database population used to create the study population.<br><br>RECORD 12.2: Authors should provide information on the data cleaning methods used in the study.	CPRD used for this research, so the authors did not have access to the database population.<br><br>Some data cleaning methods listed in supplementary material. Complete cleaning algorithms for GOLD and Aurum (in Stata) are available on request
Linkage		-		RECORD 12.3: State whether the study included person-level, institutional-level, or other data linkage across two or more databases. The methods of linkage and methods of linkage quality evaluation should be provided.	Person-level inkage
Participants	13	(a) Report the numbers of individuals at each stage of the study (*e.g.*, numbers potentially eligible, examined for eligibility, confirmed eligible, included in the study, completing follow-up, and analysed)<br><br>(b) Give reasons for non- participation at each stage.	See supplementary flowchart S1<br><br>See supplementary flowchart S1	RECORD 13.1: Describe in detail the selection of the persons included in the study (*i.e.,* study population selection) including filtering based on data quality, data availability and linkage. The selection of included persons can be described in the text and/or by means of the study flow diagram.	See supplementary flowchart S1

		(c) Consider use of a flow diagram	See supplementary flowchart S1		
Descriptive data	14	(a) Give characteristics of study participants (*e.g.*, demographic, clinical, social) and information on exposures and potential confounders<br>(b) Indicate the number of participants with missing data for each variable of interest<br>(c) *Cohort study* - summarise follow-up time (*e.g.*, average and total amount)	See Table 1<br>See Table 1<br>See Results section		
Outcome data	15	*Cohort study* - Report numbers of outcome events or summary measures over time<br>*Case-control study* - Report numbers in each exposure category, or summary measures of exposure<br>*Cross-sectional study* - Report numbers of outcome events or summary measures	See Results		
Main results	16	(a) Give unadjusted estimates and, if applicable, confounder- adjusted estimates and their	Unadjusted estimates are shown in the supplementary material. Adjustments were		

		precision (e.g., 95% confidence interval). Make clear which confounders were adjusted for and why they were included<br><br>(b) Report category boundaries when continuous variables were categorized<br><br>(c) If relevant, consider translating estimates of relative risk into absolute risk for a meaningful time period	made for age and gender (and stratification for comorbidities) in this analysis<br><br>Categories listed<br><br>Absolute risks presented for survival estimates		
Other analyses	17	Report other analyses done— e.g., analyses of subgroups and interactions, and sensitivity analyses	See Results		
Key results	18	Summarise key results with reference to study objectives	See first paragraph of Discussion		
Limitations	19	Discuss limitations of the study, taking into account sources of potential bias or imprecision. Discuss both direction and magnitude of any potential bias	See limitations	RECORD 19.1: Discuss the implications of using data that were not created or collected to answer the specific research question(s). Include discussion of misclassification bias, unmeasured confounding, missing data, and changing eligibility over time, as they pertain to the study being reported.	See limitations

Interpretation	20	Give a cautious overall interpretation of results considering objectives, limitations, multiplicity of analyses, results from similar studies, and other relevant evidence	See interpretation of findings		
Generalisability	21	Discuss the generalisability (external validity) of the study results	See Discussion		
**Other Information**					
Funding	22	Give the source of funding and the role of the funders for the present study and, if applicable, for the original study on which the present article is based	See funding information		
Accessibility of protocol, raw data, and programming code		..		RECORD 22.1: Authors should provide information on how to access any supplemental information such as the study protocol, raw data, or programming code.	Study protocol is listed. Programming code available on request.

* Benchimol EI, Smeeth L, Guttmann A, Harron K, Moher D, Petersen I, et al. The REporting of studies Conducted using Observational Routinely-collected health Data (RECORD) Statement. PLOS Medicine. 2015;12(10):e1001885.

Table S2: Clinical codes for intellectual disabilities and end-of-life care (EOLC)

Clinical codes for intellectual disabilities

The most common intellectual disability health codes (most recent; >50%) were for intellectual disability-specific health assessments (medcodes 2474674015, 302211000000112, 751221000000117, 96895, 32952) [n=3120; 32%] followed by “On learning disability register” (medcodes 2548475019, 96895) [n=1364; 14%) and “Learning disability health action plan completed” (medcodes 2534201018, 43436) [n=708; 7%).

CPRD GOLD: Intellectual disabilities		
**medcode**	**readcode**	**readterm**
302	E310.00	Moderate mental retardation, IQ in range 35-49
1278	E310.11	Imbecile
1362	E3...00	Mental retardation
1543	PJ0..00	Down's syndrome - trisomy 21
1680	E30..11	Educationally subnormal
1787	E30..00	Mild mental retardation, IQ in range 50-70
2730	13Z3.00	Low I.Q.
4246	6664	Mental handicap problem
4477	Eu81z11	[X]Learning disability NOS
4479	PK5..00	Tuberous sclerosis
4825	E311.00	Severe mental retardation, IQ in range 20-34
6123	Eu71.00	[X]Moderate mental retardation
10628	PJyy200	Fragile X chromosome
10759	PJ0z.00	Down's syndrome NOS
10956	PKy9300	Prader - Willi syndrome
11322	P020.00	Iniencephaly - closed
15846	PJ7..00	Klinefelter's syndrome
16087	PKy4.00	William syndrome
16855	Eu81z12	[X]Learning disorder NOS
18017	PJ52400	Polyploidy
18415	PJ0..12	Trisomy 21
18415	PJ0..12	Trisomy 21
18537	P233.11	Dandy-Walker syndrome
19038	PJ1z.11	Trisomy 13 NOS
19062	PJ51.00	Partial trisomy syndromes
19445	9HB..00	Learning disabilities administration status
21418	PKy6011	Cornelia de Lange syndrome
22760	918e.00	On learning disability register
23489	PJ0..11	Mongolism
25306	PKyz511	Angelman syndrome
27280	PKy0.12	Prader-Willi syndrome
27533	C03z.12	Cretinism
27691	Eu72.11	[X]Severe mental subnormality
28740	Eu70.00	[X]Mild mental retardation
28962	Eu7..00	[X]Mental retardation
30362	R034y11	[D]Global retardation
30486	P228011	Agenesis of corpus callosum
31042	Eu84200	[X]Rett's syndrome
31426	Pky1.00	Laurence-Moon-Biedl syndrome
31599	E141.11	Heller's syndrome
31795	PJ31.00	Cri-du-chat syndrome
32511	9HB3.00	Learning disabilities health assessment
32588	ZL1B500	Under care of psychiatrist for mental handicap
32603	PJyy400	Fragile X syndrome
32820	Eu7zz00	[X]Unsp mental retardation without mention impairment behav
32952	9HB5.00	Learning disabilities annual health assessment
33642	PJ2..00	Edward's syndrome - trisomy 18
33948	PJ32.11	Wolff - Hirschorn syndrome
33948	PJ32.11	Wolf Hirschhorn syndrome
33949	Eu70.12	[X]Mild mental subnormality
34161	P225.00	Holoprosencephaly
34174	Eu84112	[X]Mental retardation with autistic features
34719	PKy6400	Seckel syndrome
34734	Eu71.11	[X]Moderate mental subnormality
34913	PJ52300	Triploidy
35665	PJ1..00	Patau's syndrome - trisomy 13
36045	Eu81z13	[X]Learn acquisition disab NOS
36143	Eu72.00	[X]Severe mental retardation

36567	PKy6100	Cockayne syndrome
36871	PJ33300	Smith-Magenis syndrome
37105	PJ3..00	Monosomies and deletions from the autosomes
37591	PJ50100	Trisomy 7
37702	PJ50.00	Whole chromosome trisomy syndromes
37867	E3z..00	Mental retardation NOS
37887	Eu7z.11	[X]Mental deficiency NOS
37911	Eu7z.12	[X]Mental subnormality NOS
37924	PKy5F00	Coffin-Lowry syndrome
39016	Eu70y00	[X]Mild mental retardation, other impairments of behaviour
39017	PJ2z.00	Edward's syndrome NOS
39166	C0A1.00	Congenital iodine-deficiency syndrome, myxoedematous type
39412	Eu70100	[X]Mld mental retard sig impairment behav req attent/treatmt
41207	E141100	Residual disintegrative psychoses
41391	9HB1.00	Learning disabilities health action plan offered
41461	PKy0.11	Prader-Willi Syndrome
42520	Eu7yy00	[X]Other mental retardation, other impairments of behaviour
42589	Eu7z.00	[X]Unspecified mental retardation
42701	PJ00.00	Trisomy 21, meiotic nondisjunction
42886	Eu7z000	[X]Unsp mental retard with statement no or min impairm behav
43436	9HB4.00	Learning disabilities health action plan completed
43445	9HB2.00	Learning disabilities health action plan reviewed
43447	9HB0.00	Learning disabilities health action plan declined
43565	PJ11.00	Trisomy 13, mosaicism
45133	E312.00	Profound mental retardation with IQ less than 20
45512	PJ50x00	Whole chromosome trisomy, mosaicism
46133	PJ12.00	Trisomy 13, translocation
46429	Eu84313	[X]Heller's syndrome
46504	Eu70000	[X]Mld mental retard with statement no or min impairm behav
46787	PJ2z.11	TRISOMY 18 NOS
47449	C031.00	Goitrous cretin
49236	PJ33111	18p- syndrome
50606	Eu70z00	[X]Mild mental retardation without mention impairment behav
50751	Eu72100	[X]Sev mental retard sig impairment behav req attent/treatmt
50947	Eu72000	[X]Sev mental retard with statement no or min impairm behav
51268	Eu73.00	[X]Profound mental retardation
51622	E312.11	Idiocy
51954	E30..12	Feeble-minded
52602	Eu84400	[X]Overactive disorder assoc mental retard/stereotype movts
54179	E31z.00	Other specified mental retardation NOS
54377	PJ52.00	Trisomies of autosomes NEC
54490	PJ70.00	Klinefelter's phenotype, karyotype 47XXY
54717	P22..00	Reduction deformities of brain
54881	Eu71100	[X]Mod mental retard sig impairment behav req attent/treatmt
55560	Eu72z00	[X]Sev mental retardation without mention impairment behav
55848	Eu72y00	[X]Severe mental retardation, other impairments of behaviour
56143	E141.00	Disintegrative psychosis
56545	PJ71.11	Klinefelter's syndrome, XXXY
56547	Eu7y100	[X]Oth mental retard sig impairment behav req attent/treatmt
56577	E31..00	Other specified mental retardation
57043	PKy6600	Dubowitz syndrome
57199	E3y..00	Other specified mental retardation
57458	PKy1.11	Bardet-Biedl syndrome
57806	PJ30.11	Deletion of long arm of chromosome 21
59218	P10y000	Dandy-Walker syndrome with spina bifida
59407	Eu71y00	[X]Mod retard oth behav impair
59439	PJ7z.00	Klinefelter's syndrome NOS
60062	Eu73z00	[X]Prfnd mental retardation without mention impairment behav
60473	Eu71z00	[X]Mod mental retardation without mention impairment behav
60913	Eu71000	[X]Mod mental retard with statement no or min impairm behav
61499	PJ02.00	Trisomy 21, translocation
61532	C372z00	Other disorder of purine or pyrimidine metabolism NOS
61532	C372.00	Other disorders of purine and pyrimidine metabolism
61627	PJ0z.11	Trisomy 21 NOS
62222	Eu84312	[X]Disintegrative psychosis
62414	PJ71.12	Klinefelter's syndrome, XXXXY
62644	C372011	Lesch - Nyhan syndrome
63273	Eu7yz00	[X]Other mental retardation without mention impairment behav
63821	P02..00	Iniencephaly
64541	P22z.00	Reduction deformities of brain NOS
64822	P22y200	Gillespie syndrome

65091	PJ33211	18q- syndrome
65382	C31yX00	Disorder of glycoprotein metabolism, unspecified
65468	Eu73.11	[X]Profound mental subnormality
65509	PJ50300	Trisomy 9
66383	Eu7zy00	[X]Unspecified mental retardatn, other impairments of behav
66566	PJ32.00	Deletion of short arm of chromosome 4
66783	Eu7z100	[X]Unsp mentl retard sig impairment behav req attent/treatmt
67234	PJ21.00	Trisomy 18, mosaicism
67298	PJ33000	Deletion of long arm of chromosome 13
67513	C03..11	Cretinism
67854	PJ71.00	Klinefelter's syndrome,male with more than two X chromosomes
67927	C372300	Lesch-Nyhan syndrome
68109	PJ74.00	Klinefelter's syndrome, XY/XXY mosaic
68299	Eu84300	[X]Other childhood disintegrative disorder
68482	C372000	Hypoxanthine-guanine-phosphoribosyltransferase deficiency
69476	PJ50200	Trisomy 8
70008	Eu7y000	[X]Oth mental retard with statement no or min impairm behav
70102	Eu73000	[X]Profound ment retrd wth statement no or min impairm behav
70198	PJ50600	Trisomy 12
71196	Eu7y.00	[X]Other mental retardation
71632	E30..13	Moron
71815	PJ51000	Major partial trisomy
72139	PJ10.00	Trisomy 13, meiotic nondisjunction
72265	PJ1z.00	Patau's syndrome NOS
72331	C0A0.00	Congenital iodine-deficiency syndrome, neurological type
73990	F101.11	Amaurotic familial idiocy
84154	Eu70.11	[X]Feeble-mindedness
90276	Eu73y00	[X]Profound mental retardation, other impairments of behavr
91262	PJ73.00	Klinefelter's syndrome, XXYY
93133	PJ22.00	Trisomy 18, translocation
93441	P224.00	Arhinencephaly
93694	PJ9..00	Mowat-Wilson syndrome
95440	PJ33100	Deletion of long arm of chromosome 18
96170	C377.00	Disorders of glycoprotein metabolism
96257	PJ72.00	Klinefelter's syndrome, male with 46XX karyotype
96787	C0A..00	Congenital iodine deficiency syndrome
96895	69DB.00	Learning disability health examination
97059	PKyz700	Angelman's syndrome
97927	PJ33200	Deletion of short arm of chromosome 18
98100	Eu73100	[X]Profound ment retard sig impairmnt behav req attent/treat
98293	Eu81500	[X]Severe learning disability
98342	Eu81400	[X]Moderate learning disability
98395	PJ3z.00	Monosomies and deletions from the autosomes NOS
98617	PKyz711	Angelman syndrome
98941	PJ31.11	Deletion of short arm of chromosome 5
99674	PJ51100	Minor partial trisomy
99774	Eu81600	[X]Mild learning disability
100024	PJ50800	Trisomy 22
100024	PJ0..13	Trisomy 22
100174	PJ51z00	Partial trisomy syndrome NOS
100507	PJ33400	Jacobsen syndrome
100648	Eu81700	[X]Profound learning disability
100729	9HB7.00	Did not attend learning disabilities annual health assessmnt
100730	9HB6.00	Learning disabilities annual health assessment declined
100854	9hL0.00	Exc learn disability quality indicators: informed dissent
100965	9HB6.11	Learning disabilities annual health check declined
100980	9hL..00	Exception reporting: learning disability quality indicators
101309	PJ02.11	Partial trisomy 21 in Down's syndrome
101732	PJ50y00	Other specified whole chromosome trisomy syndrome
101982	PJ50z00	Whole chromosome trisomy syndrome NOS
101999	Eu84311	[X]Dementia infantalis
102102	PJ12.11	Partial trisomy 13 in Patau's syndrome
102234	9HB7.11	Did not attend learning disabilities annual health check
102437	9hL1.00	Exc learn disability quality indicators: patient unsuitable
103187	8Ce6.00	Preferred place of care - learning disability unit
103536	PJ51500	15q partial trisomy syndrome
103582	PJ33600	Chromosome 22q11 deletion syndrome
103805	PJ33500	Greig cephalopolysyndactyly syndrome
103873	PJ20.00	Trisomy 18, meiotic nondisjunction
103882	P01..00	Craniorachischisis
104196	PJ33800	Chromosome 4q deletion syndrome

105180	F1y0.00	Fragile X associated tremor ataxia syndrome
105277	Pyu0300	[X]Other specified congenital malformations of brain
105514	PJ33A00	Kleefstra syndrome
106114	PJ51400	Trisomy 9p syndrome
106219	9mA..00	Learning disability annual health check invitation
106247	9mA2.00	Learning disability annual health check letter invitation
106248	9mA1.00	Learning disability annual health check telephone invitation
106249	9mA0.00	Learning disability annual health check verbal invitation
106272	9mA2000	Learning disability annual health check invtation 1st letter
106274	9mA2100	Learning disability annual health check invtation 2nd letter
106276	9mA2200	Learning disability annual health check invtation 3rd letter
106563	PJ33212	18p- syndrome
106590	PKy5K00	Cohen syndrome
106704	P22yz00	Other reduction deformity of brain NOS
106856	PJ33900	Langer-Giedion syndrome
107029	PJ51200	10q partial trisomy syndrome
107119	PJ52z00	Trisomy of autosomes NEC NOS
107123	PJ30.00	Antimongolism syndrome
107162	PJ22.11	Partial trisomy 18 in Edward's syndrome
107378	P22y.00	Other specified reduction deformities of brain
107597	9hL1.00	Excluded from diabetic retinopthy screen as learn disability
107670	PJ50w00	Whole chromosome trisomy, meitotic nondisjunction
107822	PKy6900	Borjeson-Forssman-Lehmann syndrome
107919	PJ01.11	Trisomy 21, mitotic nondisjunction
107968	Eu81800	[X]Specific learning disability
108375	PG5F.00	Acrodysostosis
108623	C372.11	Lesch - Nyhan syndrome
108881	94Z9.00	Preferred place of death: learning disability unit
108947	PKyz.11	Cockayne's syndrome
109223	PJ50311	Trisomy 9 mosaic syndrome
110792	9Nh4.00	Under care of community learning disability team
110833	PJ50400	Trisomy 10
111120	PJ33113	18q deletion syndrome
112650	PJ33700	3p deletion syndrome
113018	PJ33112	18q- syndrome
114798	PJ51300	Trisomy 4p syndrome
114817	P021.00	Open iniencephaly
114820	E141000	Active disintegrative psychoses

CPRD Aurum: Intellectual disabilities

medcode	term	snomedconceptID
2090010	Fragile X syndrome	613003
5179014	Iniencephaly	2438005
9538013	Agenesis of corpus callosum	5102002
10374011	Laurence-Moon-Biedl syndrome	5619004
12877016	Tuberous sclerosis	7199000
17479015	Coffin-Siris syndrome	10007009
18114013	Lesch-Nyhan syndrome	10406007
25776014	Coffin-Lowry syndrome	15182000
35440018	Cockayne syndrome	21086008
36300015	Borjeson-Forssman-Lehmann syndrome	21634003
51766016	Holoprosencephaly	30915001
51767013	Arhinencephaly	30915001
53817017	Craniorachischisis	32219008
55063010	Greig cephalopolysyndactyly syndrome	32985001
63896011	Cornelia de Lange syndrome	40354009
68472012	Mongolism	41040004
68519010	Langer-Giedion syndrome	41069008
81676016	Trisomy 4p syndrome	49024004
94131019	Cohen syndrome	56604005
96285014	Seckel syndrome	57917004
110901011	Acrodysostosis	66758006
116814019	15q partial trisomy syndrome	70324008
119577010	Heller's syndrome	71961003
119579013	Disintegrative psychosis	71961003
121311016	10q partial trisomy syndrome	73035005
127638013	Angelman syndrome	76880004
128696013	Trisomy 9p syndrome	77527000
148214012	Prader-Willi syndrome	89392001
151009017	Mental retardation	110359009
187765019	Amaurotic familial idiocy	102454019

261108013	Trisomy 21 in amniotic foetal cell	168118008
264621015	Mental handicap problem	170695009
293559017	Other disorder of purine or pyrimidine metabolism NOS	238006008
293820017	[X]Other glycoprotein metabolism disorders	238045003
293824014	[X]Other disorders of purine and pyrimidine metabolism	238006008
293834017	[X]Disorder of glycoprotein metabolism, unspecified	238045003
294938012	Active disintegrative psychoses	191692007
294939016	Residual disintegrative psychoses	191693002
294941015	Disintegrative psychosis NOS	71961003
295661017	Other specified mental retardation	110359009
295662012	Other specified mental retardation NOS	110359009
295664013	Intellectual disability	110359009
296557014	[X]Mild mental retardation, other impairments of behaviour	86765009
296565012	[X]Mod retard oth behav impair	61152003
296574014	[X]Severe mental retardation, other impairments of behaviour	40700009
296586012	[X]Other mental retardation	110359009
296592018	[X]Other mental retardation, other impairments of behaviour	110359009
312735010	Iniencephaly - closed	203927003
312737019	Iniencephaly NOS	2438005
312878011	Reduction deformities of brain	204032005
312902012	Other specified reduction deformities of brain	204032005
312905014	Other reduction deformity of brain NOS	204032005
315350012	Trisomy 13, meiotic nondisjunction	205619006
315359013	Monosomies and deletions from the autosomes	205627002
315360015	Antimongolism syndrome	254274004
315362011	Deletion of long arm of chromosome 13	205630009
315385012	Monosomies and deletions from the autosomes NOS	254274004
315389018	Trisomy 6	205647005
315390010	Trisomy 7	205648000
315391014	Trisomy 8	205649008
315392019	Trisomy 9	205650008
315393012	Trisomy 10	205651007
315394018	Trisomy 11	205652000
315395017	Trisomy 12	205653005
315396016	Other trisomy C syndromes	270521004
315397013	Trisomy 22	205655003
315398015	Whole chromosome trisomy, meitotic nondisjunction	254269007
315399011	Whole chromosome trisomy, mosaicism	205657006
315400016	Whole chromosome trisomy, mitotic nondisjunction	205657006
315401017	Other specified whole chromosome trisomy syndrome	270521004
315402012	Whole chromosome trisomy syndrome NOS	270521004
315403019	Partial trisomy syndromes	205660004
315404013	Major partial trisomy	205661000
315405014	Minor partial trisomy	205662007
315406010	Partial trisomy syndrome NOS	205660004
315407018	Trisomies of autosomes NEC	270521004
315413010	Trisomy of autosomes NEC NOS	270521004
315629012	Biemond's syndrome	205828009
315463017	Klinefelter's syndrome, XXYY	205699007
315464011	Klinefelter's syndrome, XY/XXY mosaic	205700008
315465012	Klinefelter's syndrome NOS	22053006
315486012	Fragile X chromosome	315486012
315655013	[X]Other reduction deformities of brain	204032005
315656014	[X]Other specified congenital malformations of brain	88425004
329968011	Cretinism	217710005
377071011	Gillespie syndrome	253176002
378494019	Partial trisomy 21 in Down's syndrome	254264002
378496017	Edward's syndrome NOS	51500006
378497014	Partial trisomy 18 in Edward's syndrome	254266000
378499012	Patau's syndrome NOS	21111006
378500015	Partial trisomy 13 in Patau's syndrome	254268004
398888012	Other disorders of purine and pyrimidine metabolism	238006008
400767012	Reduction deformities of brain NOS	204032005
401902015	[X]Unspecified mental retardation	110359009
401910019	[X]Other childhood disintegrative disorder	35919005
405346013	Deletion of long arm of chromosome 18	270889005
405347016	18q- syndrome	270889005
405348014	18p- syndrome	270890001
405349018	Deletion of short arm of chromosome 18	270890001
413177014	Educationally subnormal	276854003
415431010	Goitrous cretin	278503003

500490013	Triploidy	66651005
502310015	Polyploidy	72991005
502891013	Congenital iodine deficiency syndrome	75065003
507246016	Mild mental retardation, IQ in range 50-70	86765009
1221474011	Deletion of short arm of chromosome 4	17122004
1222495016	[D]Global retardation	224958001
1224878018	Trisomy 21 NOS	41040004
1224879014	Trisomy 18 NOS	51500006
1224880012	Trisomy 13 NOS	21111006
1224941015	Dubowitz syndrome	2593002
1232445010	William syndrome	63247009
1233229018	Deletion of short arm of chromosome 5	70173007
1234038018	Angelman's syndrome	76880004
1780502018	Smith-Magenis syndrome	401315004
2162372014	Klinefelter's syndrome	22053006
2474674015	Learning disabilities health assessment	413126003
2474708013	Learning disabilities health action plan declined	413162002
2474709017	Learning disabilities health action plan reviewed	413163007
2534201018	Learning disabilities health action plan completed	712491005
2548475019	On learning disability register	416075005
2900005013	Fragile X associated tremor ataxia syndrome	448045004
3505252012	Alpha-thalassaemia intellectual disability syndrome linked to chromosome 16	734349003
3528198012	Trisomy 13	737540008
3528201019	Trisomy 18	737541007
3528203016	Trisomy 21	737542000
9881000006115	Other specified mental retardation	110359009
53891000006113	Wolff - Hirschorn syndrome	17122004
60541000006119	Whole chromosome trisomy syndromes	205646001
88251000006115	Trisomy 13, mitotic nondisjunction	205620000
88261000006118	Trisomy 13, mosaicism	205620000
88271000006113	Trisomy 13, translocation	254268004
88291000006114	Trisomy 18, meiotic nondisjunction	205623003
88301000006110	Trisomy 18, mitotic nondisjunction	205624009
88311000006113	Trisomy 18, mosaicism	205624009
88321000006117	Trisomy 18, translocation	254266000
88351000006114	Trisomy 21, meiotic nondisjunction	205615000
88361000006111	Trisomy 21, mitotic nondisjunction	205616004
88371000006116	Trisomy 21, mosaicism	205616004
88381000006118	Trisomy 21, translocation	254264002
88391000006115	Trisomy 22	205655003
117831000006117	Suspect trisomy 21 foetus	415683005
146051000006113	Severe mental retardation, IQ in range 20-34	40700009
189601000000111	Learning disabilities administration status	112851000000104
189611000000113	Learning disabilities health action plan offered	112861000000101
201751000006110	Profound mental retardation with IQ less than 20	31216003
212911000006116	Prader - Willi syndrome	89392001
212921000006112	Prader-Willi Syndrome	89392001
215821000000119	Moron	86765009
222121000000113	Trisomy 21	41040004
222131000000110	Klinefelter's syndrome, XXXY	275263003
222141000000118	Klinefelter's syndrome, XXXXY	275264009
223441000000119	Lesch - Nyhan syndrome	10406007
239991000006111	Patau's syndrome - trisomy 13	21111006
255141000006118	Open iniencephaly	203928008
302211000000112	Learning disabilities annual health assessment	199751000000100
376631000006115	[X]Dementia infantalis	71961003
377461000006113	[X]Disintegrative psychosis	35919005
386751000006111	[X]Feeble-mindedness	86765009
388761000006114	[X]Heller's syndrome	71961003
395051000006119	[X]Learn acquisition disab NOS	110359009
395061000006117	[X]Learning disability NOS	1855002
395071000006112	[X]Learning disorder NOS	1855002
398201000006115	[X]Mental deficiency NOS	110359009
398231000006111	[X]Mental retardation	110359009
398241000006118	[X]Mental retardation with autistic features	231536004
398251000006116	[X]Mental subnormality NOS	110359009
398381000006119	[X]Mild mental retardation	86765009
398391000006116	Mild intellectual disability	86765009
398411000006116	[X]Mild mental subnormality	86765009
398651000006118	[X]Mld mental retard sig impairment behav req attent/treatmt	86765009
398661000006116	[X]Mld mental retard with statement no or min impairm behav	86765009

398761000006113	[X]Mod mental retard sig impairment behav req attent/treatmt	61152003
398771000006118	[X]Mod mental retard with statement no or min impairm behav	61152003
398781000006115	[X]Mod mental retardation without mention impairment behav	61152003
398811000006118	Moderate intellectual disability	61152003
398821000006114	[X]Moderate mental subnormality	61152003
404851000006117	[X]Oth mental retard sig impairment behav req attent/treatmt	110359009
404861000006115	[X]Oth mental retard with statement no or min impairm behav	110359009
405381000006115	[X]Oth specif trisomies & partial trisomies of autosomes	270521004
411791000006118	[X]Other mental retardation without mention impairment behav	110359009
417681000006116	[X]Overactive disorder assoc mental retard/stereotype movts	35919005
423361000006118	[X]Prfnd mental retardation without mention impairment behav	31216003
423481000006119	[X]Profound ment retard sig impairmnt behav req attent/treat	31216003
423491000006116	[X]Profound ment retrd wth statement no or min impairm behav	31216003
423501000006112	Profound intellectual disability	31216003
423511000006110	[X]Profound mental retardation, other impairments of behavr	31216003
423521000006119	[X]Profound mental subnormality	31216003
424971000006111	[X]Rett's syndrome	68618008
426521000006114	[X]Sev mental retard sig impairment behav req attent/treatmt	40700009
426531000006112	[X]Sev mental retard with statement no or min impairm behav	40700009
426541000006119	[X]Sev mental retardation without mention impairment behav	40700009
426591000006111	Severe intellectual disability	40700009
426611000006117	[X]Severe mental subnormality	40700009
430061000006113	[X]Unsp mental retard with statement no or min impairm behav	110359009
430071000006118	[X]Unsp mental retardation without mention impairment behav	110359009
430081000006115	[X]Unsp mentl retard sig impairment behav req attent/treatmt	110359009
431231000006115	[X]Unspecified mental retardatn, other impairments of behav	110359009
557191000000119	Mowat-Wilson syndrome	703535000
571541000006116	Cockayne's syndrome	21086008
583281000006118	Congenital iodine-deficiency syndrome, mixed type	237565000
583291000006115	Congenital iodine-deficiency syndrome, myxoedematous type	75065003
583301000006119	Congenital iodine-deficiency syndrome, neurological type	237566004
600251000000118	Jacobsen syndrome	715438008
600791000006112	Cretinism	217710005
600811000006111	Cri-du-chat syndrome	70173007
607731000006114	Dandy-Walker syndrome	14447001
612441000006114	Deletion of long arm of chromosome 21	254274004
623971000006112	Disorder of glycoprotein metabolism, unspecified	238045003
624501000006112	Disorders of glycoprotein metabolism	238045003
628281000006114	Down's syndrome - trisomy 21	41040004
636711000006113	Edward's syndrome - trisomy 18	51500006
667621000006119	Feeble-minded	86765009
700071000006118	Moderate mental retardation, IQ in range 35-49	61152003
733951000006111	Low I.Q.	102942005
747231000006115	Lesch - Nyhan syndrome	10406007
751221000000117	Learning disability health examination	442127005
753551000006117	Klinefelter's phenotype, karyotype 47XXY	405769009
753581000006113	Klinefelter's syndrome, male with 46XX karyotype	205698004
753631000006111	Klinefelter's syndrome,male with more than two X chromosomes	22053006
785941000006115	Imbecile	61152003
787151000006113	Idiocy	31216003
787441000006118	Hypoxanthine-guanine-phosphoribosyltransferase deficiency	124275001
857391000006110	Referral to learning disability team	857391000006106
882761000006114	Mental subnormality	91138005
882771000006119	Mild mental retardation	86765009
882781000006116	Moderate mental retardation	61152003
882791000006118	Severe mental retardation	40700009
882801000006117	Mental subnormality NOS	686511000000102
893501000006110	Fragile X syndrome	205720009
940371000006118	Fragile X syndrome	940371000006102
968201000006114	Mental retardation, congenital heart disease, blepharophimosis, blepharoptosis and hypoplastic teeth	412787009
988941000006119	Mental subnormality NOS	91138005
1009521000006116	Cause of learning disabilities	1009521000006100
1009531000006118	Cause of learning disabilities: Down's syndrome	1009531000006102
1009541000006111	Cause of learning disabilities: Tuberous sclerosis	1009541000006107
1009551000006113	Cause of learning disabilities: Birth trauma	1009551000006109
1009561000006110	Cause of learning disabilities: Meningitis/encephalitis	1009561000006106
1009571000006115	Cause of learning disabilities: Fragile X syndrome	1009571000006104
1009581000006117	Cause of learning disabilities: Late effect of head injury	1009581000006101
1009591000006119	Cause of learning disabilities: Brain tumour	1009591000006103
1009601000006110	Cause of learning disabilities: Congenital hydrocephalus	1009601000006106
1009611000006113	Cause of learning disabilities: Microcephaly	1009611000006109

1009621000006117	Cause of learning disabilities: Phenylketonuria	1009621000006101
1009631000006119	Cause of learning disabilities: Prader-Willi syndrome	1009631000006103
1009641000006112	Cause of learning disabilities: Smith-Magenis syndrome	1009641000006108
1009651000006114	Cause of learning disabilities: Rett syndrome	1009651000006105
1009661000006111	Cause of learning disabilities: Congenital rubella	1009661000006107
1009671000006116	Cause of learning disablts: Unknown/awaiting investigation	1009671000006100
1009671000006116	Cause of learning disablts: Unknown/awaiting investigation	1009671000006116
1009681000006118	Cause of learning disabilities: Unknown/despite investigation	1009681000006102
1009691000006115	Cause of learning disabilities: Other	1009691000006104
1009801000006111	Level of support for person with adult learning disablts. (ALD)	1009801000006107
1129781000000117	[X]Severe learning disability	508171000000105
1129811000000119	[X]Moderate learning disability	984671000000103
1142901000000119	Learning disabilities annual health assessment declined	514021000000103
1158111000000115	Preferred place of care - learning disability unit	518661000000109
1158321000000112	Preferred place of death: learning disability unit	518741000000100
1162851000000116	Learning disability annual health check invitation	520801000000100
1162891000000112	Learning disability annual health check verbal invitation	520821000000109
1162931000000119	Learning disability annual health check telephone invitation	520841000000102
1164251000000114	Learning disability annual health check letter invitation	521421000000100
1175631000000111	Exception reporting: learning disability quality indicators	717271000000102
1177021000000114	Learning disabilities annual health check declined	514021000000103
1177051000000116	Did not attend learning disabilities annual health check	514041000000105
1550041000000110	[X]Mild learning disability	984661000000105
1550051000000113	[X]Profound learning disability	984681000000101
1620441000006116	Learning disability	1620441000006100
1694831000006112	Pitt-Hopkins syndrome	702344008
1704791000006112	Excluded from diabetic retinopthy screen as learn disability	374841000000109
1705901000006118	Angelman syndrome	76880004
1741521000006113	Did not attend learning disabilities annual health assessmnt	514041000000105
1741551000006116	Excepted from learning disability quality indicators - informed dissent	716441000000105
1741561000006119	Excepted from learning disability quality indicators - patient unsuitable	716451000000108
1755011000006114	Learning disability annual health check invtation 1st letter	712781000000107
1755021000006118	Learning disability annual health check invitation second letter	712801000000108
1755031000006115	Learning disability annual health check invitation third letter	712821000000104
1781041000006119	Fragile X associated tremor ataxia syndrome	759431000000104
1786301000006110	18q- syndrome	270889005
1786311000006113	18p- syndrome	270890001
1798581000000119	Chromosome 22q11 deletion syndrome	449818005
1798621000000119	Chromosome 4q deletion syndrome	37506004
1823961000006113	Learning disability confirmed	1823961000006109
1855811000006118	Learning disability monitoring in primary care	1855811000006102
1855831000006112	Learning disability monitoring in secondary care	1855831000006108
1856971000006112	Did not attend learning disability monitoring	1856971000006108
1856981000006110	Learning disability follow-up	1856981000006106
1860901000006112	Preferred place of care no longer learning disability unit	1860901000006108
1872721000006112	Special education needs - specific learning disability	1872721000006108
1887331000006119	[X]Specific learning disability	889211000000104
2008481000006110	Learning disabilities: reasonable adjustments	2008481000006106
2107961000000111	3p deletion syndrome	449819002
2108161000000114	18q deletion syndrome	270889005
2108171000000119	Trisomy 9 mosaic syndrome	74350000
2114791000000110	Kleefstra syndrome	724207001
2249771000000115	Under care of community learning disability team	870651000000103
2508311000006110	FRAXA - Fragile X syndrome	613003
2528351000006115	General learning disability	1855002
2537911000006119	Iniencephalus	2438005
2589171000006110	Bardet-Biedl syndrome	5619004
2614591000006110	Tuberous sclerosis syndrome	7199000
2614641000006117	TS - Tuberous sclerosis	7199000
2665621000006114	Lesch-Nyhan disease	10406007
2730191000000119	Profound intellectual development disorder with impairment of behaviour	1089731000000104
2730211000000115	Severe intellectual development disorder without significant impairment of behaviour	1089741000000108
2730231000000111	Severe intellectual development disorder with significant impairment of behaviour	1089751000000106
2730271000000113	Severe intellectual development disorder with impairment of behaviour	1089771000000102
2730291000000112	Moderate intellectual development disorder without significant impairment of behaviour	1089781000000100
2730311000000113	Moderate intellectual development disorder with significant impairment of behaviour	1089791000000103
2730351000000112	Moderate intellectual development disorder with minimal impairment of behaviour	1089811000000102

2730371000000115	Moderate intellectual development disorder with impairment of behaviour	1089821000000108
2730391000000116	Mild intellectual development disorder without significant impairment of behaviour	1089831000000105
2730411000000116	Mild intellectual development disorder with significant impairment of behaviour	1089841000000101
2730431000000112	Mild intellectual development disorder with minimal impairment of behaviour	1089851000000103
2731751000006110	Amaurotic idiocy, early juvenile type	14637005
2731761000006112	Amaurotic idiocy, late infantile type	14637005
2731801000006115	Amaurotic idiocy early juvenile type	14637005
2731811000006117	Amaurotic idiocy late infantile type	14637005
2771711000006112	Chromosome 4 short arm deletion syndrome	17122004
2771701000006114	4p partial monosomy syndrome	17122004
2835541000006116	Complete trisomy 13 syndrome	21111006
2835561000006117	D>1< trisomy syndrome	21111006
2851171000006117	Klinefelter syndrome	22053006
2998331000006113	Holoprosencephaly sequence	30915001
2998361000006116	Familial alobar holoprosencephaly	30915001
2998371000006111	HPE - Holoprosencephaly	484738017
3003301000006113	Profound mental handicap	31216003
3003311000006111	Profound mental retardation (Intelligence Quotient below 20)	31216003
3003321000006115	Profound learning disability with intelligence quotient less than 20	31216003
3003341000006110	Profound learning disability, intelligence quotient less than 20	31216003
3003361000006114	Profound learning disability	31216003
3018711000006116	Craniorachischisis (fissure skull/vertebral column)	32219008
3065731000006116	Trisomy X syndrome	35111009
3155151000006112	Severe learning disability	40700009
3155171000006119	Severe mental handicap	40700009
3155181000006116	Severe mental retardation (Intelligence Quotient 20-34)	40700009
3155191000006118	Severe learning disability, intelligence quotient in range 20-34	40700009
3161031000006112	Complete trisomy 21 syndrome	41040004
3161041000006119	Down syndrome	41040004
3161051000006117	T21 - Trisomy 21	41040004
3293221000006118	Trisomy for short arm of chromosome 4	49024004
3334491000006116	Complete trisomy 18 syndrome	51500006
3334501000006112	Edwards syndrome	51500006
3428711000006117	Congenital malformation of brain	57148006
3462421000006114	Cutis laxa-corneal clouding-oligophrenia syndrome	59252009
3470721000006112	Dyslexia, learning disturbance	2920630019
3493641000006112	Moderate mental handicap	61152003
3493641000006112	Moderate mental handicap	1232179016
3493651000006114	Moderate mental retardation (Intelligence Quotient 35-49)	61152003
3493661000006111	Moderate learning disability, intelligence quotient in range 35-49	61152003
3493681000006118	Moderate learning disability	1666321000000118
3501701000006113	Amaurotic idiocy, juvenile type	61663001
3501751000006112	Amaurotic idiocy juvenile type	61663001
3507751000006119	Adult-type amaurotic idiocy	62009002
3507761000006117	Late familial amaurotic idiocy	62009002
3507771000006112	Amaurotic idiocy late familial	62009002
3507791000006113	Amaurotic idiocy adult type	62009002
3528631000006110	Williams syndrome	63247009
3562141000006117	Mucopolysaccharidosis type I-H	65327002
3583901000006111	Triploidy syndrome	66651005
3616531000006110	Rett's disorder	68618008
3616571000006113	RTS - Rett syndrome	68618008
3640931000006110	5p partial monosomy syndrome	70173007
3640971000006113	Partial deletion of short arm of chromosome 5 syndrome	70173007
3670581000006118	Childhood disintegrative disorder	71961003
3670601000006111	Dementia infantilis	71961003
3670621000006118	CDD - Childhood disintegrative disorder	71961003
3687431000006110	Polyploidy syndrome	72991005
3708621000006115	Complete trisomy 9 syndrome	74350000
3720391000006119	Endemic cretinism - hypothyroid	75065003
3750051000006112	Angelman syndrome	76880004
3790911000006117	Lowe syndrome	79385002
3910901000006110	Mild mental handicap	86765009
3910921000006117	Mild mental retardation (Intelligence Quotient 50-70)	86765009
3910931000006119	Mild learning disability, intelligence quotient in range 50-70	86765009
3910951000006114	Mild learning disability	86765009
3980931000006116	MR - Mental retardation	91138005
4164891000006115	Kohlschutter's syndrome	109478007
4611091000006111	Trisomy 21 in amniotic foetal cell	168118008
4611111000006119	Trisomy 21 in amniotic fetal cell	168118008

4822501000006117	Iniencephaly - open	203928008
4822661000006115	Dandy-Walker syndrome with spina bifida	203957007
4830941000006112	Trisomy 21- meiotic nondisjunction	205615000
4830961000006111	Mosaic Down syndrome	205616004
4831001000006119	Trisomy 18 - meiotic nondisjunction	205623003
4831021000006112	Monosomy and deletion from autosome	205627002
4831091000006114	Whole chromosome trisomy syndrome	205646001
4831401000006114	Klinefelter's syndrome - male with 46 XX karyotype	205698004
4831411000006112	Klinefelter syndrome - male with 46 XX karyotype	205698004
4831431000006118	Klinefelter syndrome, XXYY	205699007
4831451000006113	Klinefelter syndrome, XY/XXY mosaic	205700008
4904351000006117	Congenital iodine deficiency syndrome	217710005
4976471000006117	Intellectual disability	228156007
5095461000006114	Prune belly syndrome with pulmonic stenosis, mental retardation and deafness	236529001
5108931000006112	Congenital iodine deficiency syndrome - mixed type	237565000
5108941000006119	Endemic cretinism - mixed type	237565000
5108951000006117	Congenital iodine deficiency syndrome - neurological type	237566004
5108961000006115	Endemic cretinism - neurological type	237566004
5115691000006114	Purine and pyrimidine metabolism disorder	238006008
5116431000006115	Glycoprotein metabolism disorder	238045003
5339911000006112	Trisomy 21 - translocation	254264002
5339921000006116	Partial trisomy 21 in Down syndrome	254264002
5339941000006111	Trisomy 18 - translocation	254266000
5339951000006113	Partial trisomy 18 in Edward syndrome	254266000
5339971000006115	Trisomy 13 - translocation	254268004
5339981000006117	Partial trisomy 13 in Patau syndrome	254268004
5339991000006119	Whole chromosome trisomy - meiotic nondisjunction	254269007
5569261000006110	Family history: Mental handicap	275101005
5571371000006116	Klinefelter's syndrome XXXY	275263003
5571381000006118	Klinefelter syndrome XXXY	275263003
5571391000006115	Klinefelter's syndrome XXXXY	275264009
5571401000006118	Klinefelter syndrome XXXXY	275264009
5579621000006110	Developmental handicap screening	275976000
5591451000006112	ESN - Educationally subnormal	276854003
5646491000006112	Family history of trisomy 21 down syndrome	3285396017
5954321000006111	Referral to psychiatrist for mental handicap	306294000
6348371000006113	Translocation Down syndrome	371045000
6682941000006119	Oculo-cerebro-cutaneous syndrome (aplasia cutis, skin tags, eye & brain defects)	403554008
6718291000006110	Klinefelter's syndrome, XXY	405769009
6718311000006114	Klinefelter's syndrome karyotype 47 XXY	405769009
6718331000006115	Klinefelter syndrome, XXY	405769009
6762801000006118	Learning disability	408468001
6881331000006110	Suspect trisomy 21 foetus	415683005
6989771000006117	X-linked mental retardation with marfanoid habitus syndrome	422437002
7349161000006111	FXTAS - Fragile X associated tremor ataxia syndrome	448045004
7375341000006111	22q11 deletion syndrome	449818005
7375381000006117	Chromosome 3p deletion syndrome	449819002
7519841000006110	Ohdo syndrome, Maat-Kievit-Brunner type	699297004
7519871000006119	Ohdo syndrome, Say-Barber-Biesecker-Young-Simpson variant	699298009
7525391000006116	Renpenning syndrome	699669001
7558801000006110	Chromosome 2q37 deletion syndrome	702357000
7567011000006112	Lubs X-linked mental retardation syndrome	702816000
7577251000006111	Microcephaly, mental retardation and distinct features, with or without Hirschsprung disease	703535000
7577261000006113	Hirschsprung disease-mental retardation syndrome	703535000
7617481000006115	Lymphangioleiomyomatosis due to tuberous sclerosis syndrome	707433009
7732201000006113	Distal partial deletion of long arm of chromosome 11	715438008
7750931000006111	FRAXE intellectual disability syndrome	716709002
7758091000006113	X-linked epilepsy with learning disability and behaviour disorder syndrome	717223008
7769521000006118	Wolf Hirschhorn syndrome	718226002
7772291000006116	Clinical Outcomes in Routine Evaluation Learning Disabilities	718440004
7772301000006115	CORE-LD (Clinical Outcomes in Routine Evaluation-Learning Disabilities)	718440004
7774181000006117	Achalasia microcephaly syndrome	718573009
7775921000006119	Oro-facial digital syndrome type 9	718680001
7777561000006111	Spondyloepiphyseal dysplasia, craniosynostosis, cleft palate, cataract and intellectual disability syndrome	718766002
7780351000006110	X-linked intellectual disability Schimke type	719010001
7781071000006113	Shprintzen Goldberg craniosynostosis syndrome	719069008
7781091000006114	Shprintzen-Goldberg syndrome	719069008
7782161000006110	X-linked intellectual disability with cerebellar hypoplasia syndrome	719136005

7782551000006115	Syndromic X-linked intellectual disability type 7	719160009
7785901000006115	Microcephalus with brachydactyly and kyphoscoliosis syndrome	719378009
7788761000006111	19q13.11 microdeletion syndrome	719599008
7791221000006119	Chromosome Xp11.3 microdeletion syndrome	719808002
7791271000006118	X-linked intellectual disability Cabezas type	719811001
7792771000006118	Chromosome Xq28 trisomy syndrome	719909009
7803961000006117	Aniridia and intellectual disability syndrome	720468000
7804931000006111	Autosomal recessive limb girdle muscular dystrophy type 2K	720523006
7806401000006115	Cerebro-facio-thoracic dysplasia	720635002
7809711000006116	Filippi syndrome	720954000
7810331000006116	Alport syndrome, intellectual disability, midface hypoplasia, elliptocytosis syndrome	720982007
7811761000006116	Deafness and intellectual disability Martin Probst type syndrome	721087008
7828621000006116	Spastic paraplegia, intellectual disability, palmoplantar hyperkeratosis syndrome	722209002
7832001000006117	Male hypergonadotropic hypogonadism, intellectual disability, skeletal anomaly syndrome	722459008
7842691000006118	Isodicentric chromosome 15 syndrome	723332005
7842741000006116	Faciocardiorenal syndrome	723333000
7850381000006114	Retinitis pigmentosa, intellectual disability, deafness, hypogenitalism syndrome	724001005
7865911000006117	Temple Baraitser syndrome	725140007
7874821000006113	Intellectual disability Buenos Aires type	725906006
7881411000006110	Weaver Williams syndrome	726670008
7952761000006111	Epilepsy telangiectasia syndrome	733032006
7953441000006114	Pseudoprogeria syndrome	733086003
7955161000006118	Dementia with Down syndrome	733194007
7957681000006110	Facial dysmorphism, macrocephaly, myopia, Dandy-Walker malformation syndrome	733417008
7958391000006116	Microcephalus, glomerulonephritis, marfanoid habitus syndrome	733472005
8191411000006110	[X]Severe learning disability	508171000000105
8195041000006117	Cause of learning disability	518831000000103
8334081000006112	Down syndrome height centile	926081000000108
8334111000006118	Down syndrome weight centile	926111000000100
8337561000006119	Significant learning disability	931001000000105
8337591000006110	Significant intellectual disability	931001000000105
8459481000006112	Learning disability annual health check invitation SMS (short message service) text message	1083061000000109
8459511000006116	Learning disability annual health check invitation email	1083091000000103
11903451000006118	Suspect trisomy 21 fetus	415683005
12007891000006110	Wiedemann Steiner syndrome	763618001
12009301000006116	Hypotonia, speech impairment, severe cognitive delay syndrome	763722004
12009521000006116	Intellectual disability, alacrima, achalasia syndrome	763741001
12009571000006115	Intellectual disability, brachydactyly, Pierre Robin syndrome	763744009
12009911000006119	Macrocephaly and developmental delay syndrome	763773007
12010191000006115	Malan overgrowth syndrome	763795006
12010231000006113	Agenesis of corpus callosum and abnormal genitalia syndrome	763797003
12022541000006113	Intellectual disability Birk-Barel type	764861005
12047711000006118	Nijmegen breakage syndrome-like disorder	766753005
12053971000006116	22q11.2 deletion syndrome	767263007
12077271000006118	PPP2R5D-related intellectual disability	768677000
12177531000006112	Profound intellectual disability	31216003
12178711000006112	Hyperphosphatasaemia with intellectual disability	33982008
12181611000006117	Severe intellectual disability	40700009
12190771000006114	Moderate intellectual disability	61152003
12202441000006114	Mild intellectual disability	86765009
12222301000006114	Whole chromosome trisomy meiotic nondisjunction	254269007
12321891000006112	Intellectual disability, congenital heart disease, blepharophimosis, blepharoptosis and hypoplastic teeth	412787009
12337641000006113	X-linked intellectual disability with marfanoid habitus	422437002
12359021000006117	Lubs X-linked intellectual disability syndrome	702816000
12359591000006114	Hirschsprung disease-intellectual disability syndrome	703535000
12461271000006111	[V]Screening for unspecified mental disorder or developmental handicap	926541000000111
12487761000006115	Mental retardation	91138005
12487771000006110	Mental retardation NOS	91138005
12620351000006114	STOMP (Stopping Over-Medication of People with Learning Disability, Autism or Both) medication review	1106111000000108
12702311000006113	Whole chromosome trisomy - meiotic nondisjunction	254269007
12703781000006112	Mild mental retardation (I.Q. 50-70)	86765009
12703791000006110	Moderate mental retardation (I.Q. 35-49)	61152003
12703921000006110	Profound mental retardation (I.Q. below 20)	31216003
12703941000006115	Severe mental retardation (I.Q. 20-34)	40700009

12704331000006114	Endemic cretinism	75065003
12728531000006116	Other specified mental retardation NOS	686511000000102
12759421000006112	FXTAS - Fragile X associated tremor ataxia syndrome	759431000000104
13491161000006116	10q22.3q23.3 microdeletion syndrome	770401007
13491541000006114	Early-onset epileptic encephalopathy and intellectual disability due to GRIN2A mutation	770431001
13492091000006117	Microcephalic primordial dwarfism Alazami type	770564004
13492711000006113	Ring chromosome 12 syndrome	770595006
13494421000006116	3q27.3 microdeletion syndrome	770719004
13494971000006112	Intellectual disability, seizures, hypotonia, ophthalmologic, skeletal anomalies syndrome	770755007
13497191000006119	Autosomal recessive intellectual disability, motor dysfunction, multiple joint contracture syndrome	770901001
13498421000006112	Monosomy 9p	771072001
13501631000006113	Polymicrogyria with optic nerve hypoplasia	771336003
13510661000006110	White Sutton syndrome	772127009
13520601000006115	Cyclin-dependent kinase-like 5 deficiency	773230003
13521831000006119	CK syndrome	773329005
13522641000006119	Severe feeding difficulties, failure to thrive, microcephaly due to ASXL3 deficiency syndrome	773400009
13522691000006111	Intellectual disability with strabismus syndrome	773405004
13523971000006111	Autosomal recessive cerebellar ataxia, epilepsy, intellectual disability syndrome due to TUD deficiency	773498006
13524851000006112	Intellectual disability, craniofacial dysmorphism, cryptorchidism syndrome	773581009
13525931000006114	Distal Xq28 microduplication syndrome	773670004
13530051000006113	AHDC1-related intellectual disability, obstructive sleep apnoea, mild dysmorphism syndrome	774068004
13621171000006117	Distal trisomy 18q	782676009
13622001000006116	Severe intellectual disability, progressive spastic diplegia syndrome	782723007
13622051000006117	Intellectual disability, facial dysmorphism syndrome due to SETD5 haploinsufficiency	782736007
13622071000006110	Diffuse cerebral and cerebellar atrophy, intractable seizures, progressive microcephaly syndrome	782737003
13622301000006119	Intellectual disability, coarse face, macrocephaly, cerebellar hypotrophy syndrome	782753000
13622561000006113	Congenital muscular dystrophy with intellectual disability and severe epilepsy	782772000
13624181000006116	Infantile spasms, psychomotor retardation, progressive brain atrophy, basal ganglia disease syndrome	782886007
13627911000006119	Congenital muscular dystrophy with intellectual disability	783174004
13632231000006117	White matter hypoplasia, corpus callosum agenesis, intellectual disability syndrome	783703004
13697811000006116	Significant learning disability	1239331000000100
13784441000006119	Learning disability	110359009
13904831000006116	Alopecia, epilepsy, intellectual disability syndrome Moynahan type	788417006
13943451000006115	Dandy-Walker malformation with hydrocephalus	840471002
14132561000006113	Bilateral megalencephaly	879919001
14134231000006111	12q15 deletion syndrome	880081006
14135491000006110	3p25.3 deletion syndrome	890123006
14137521000006116	Bilateral frontal polymicrogyria	890285006
14137531000006118	Bilateral frontoparietal polymicrogyria	890286007
14152591000006118	Molybdenum cofactor deficiency complementation group B	1003368009
14152831000006119	Mosaic 1q duplication	1003389000
14259191000006116	Intellectual disability care plan	962361000000105
14358121000006118	Under care of learning disability team	1366181000000109
14552291000006113	Early-onset epilepsy, intellectual disability, brain anomalies syndrome	1172627007
14552331000006118	TBCK-related intellectual disability syndrome	1172628002
14753781000006110	PHIP-related behavioural problems, intellectual disability, obesity, dysmorphic features syndrome	1208987006

Clinical codes for end-of-life care

Almost 75% of all first EOLC records for both CPRD Aurum and CPRD GOLD constituted 6 codes:

- “Palliative Care” (medcodes 7060, 9996, 460928017): 38.8% intellectual disabilities; 37.0% no intellectual disabilities
- “On gold standards palliative care framework” (medcodes 12739, 2534178018): 15.7% intellectual disabilities; 16.4% no intellectual disabilities
- “Completion of DS1500 terminal illness medical report” (medcodes 19051, 22519): 3.3% intellectual disabilities; 7.2% no intellectual disabilities
- “Palliative treatment” (medcodes 18511, 1488778012): 4.9% intellectual disabilities; 4.8% no intellectual disabilities

- “Referral to [specialist] palliative care services” (9755, 449190016): 6.5% intellectual disabilities; 5.5% no intellectual disabilities
- “End of life care” (medcodes 106773, 41051000000112): 5.1% intellectual disabilities; 4.0% no intellectual disabilities

CPRD GOLD: End-of-life care		
medcode	readcode	readterm
6924	9EB5.00	Completion of DS1500 terminal illness medical report
7060	ZV57C00	[V]Palliative care
9755	8H7g.00	Referral to palliative care service
9996	Z172.00	Palliative care
10019	8BAP.00	Specialist palliative care
10784	8BAR.00	Specialist palliative care treatment - inpatient
11318	8BAN.00	Community specialist palliative care
11978	ZL9AR00	Seen by palliative care physician
12739	8CM1.00	On gold standards palliative care framework
18551	8BJ1.00	Palliative treatment
22288	8HH7.00	Referred to community specialist palliative care team
26352	8H6A.00	Refer to terminal care consult
26353	1Z01.00	Terminal illness - late stage
26354	8BAT.00	Specialist palliative care treatment - outpatient
28899	ZL18R00	Under care of palliative care physician
34531	8H7L.00	Refer for terminal care
38123	8BAQ.00	Final days pathway
48775	9Nh0.00	Under the care of community palliative care team
49651	8BAS.00	Specialist palliative care treatment - daycare
73313	9b9B.00	Palliative medicine
74909	8CM4.00	Liverpool care pathway for the dying
96936	9NlJ.00	Seen by palliative care service
98251	8BAe.00	Anticipatory palliative care
98521	8Cc4.00	Management of multiple sclerosis in palliative phase
100126	9Ng7.00	On end of life care register
100466	8CM1300	Gold standards framework supportive care stage 3 - last days: category D - days prognosis
100525	8CM1100	Gold standards framework supportive care stage 2 - increasing decline
100607	8CM1000	Gold standards framework supportive care stage 1 - advancing disease
100660	2JE..00	Last days of life
101636	8CM1200	Gold standards framework supportive care stage 3 - last days: category C - weeks prognosis
102415	8CM1400	Gold standards framework supportive care stage 3 - last days: category B - months prognosis
103941	8IEE.00	Referral to community palliative care team declined
104282	8CMQ.00	On Liverpool care pathway for the dying
104463	9NgD.00	Under care of palliative care service
105214	9NNd.00	Under care of palliative care specialist nurse
105222	8CMW300	End of life care pathway
105306	8CM1600	Gold standards framework prognostic indicator stage B (green) - months prognosis
105314	8CM1700	Gold standards framework prognostic indicator stage C (yellow) - weeks prognosis
105391	9K9..00	Palliative care handover form completed
105447	8CM1800	Gold standards framework prognostic indicator stage D (red) - days prognosis
105757	9G8..00	Ambulance service notified of patient on end of life care register
105975	9c0P.00	Current palliative oncology treatment
106582	9NNf000	Under care of palliative care physician
106662	8CMb.00	Integrated care priorities for end of life
106667	8B2a.00	Prescription of palliative care anticipatory medication
106773	8BA2.11	End of life care
107906	67Q0.00	Last days of life discussed
108547	38QK.00	Palliative Care Problem Severity Score (observable entity)
110429	2Jf..00	Last weeks of life
111170	2Jg..00	Last months of life

CPRD Aurum: End-of-life care		
medcode	term	snomedconceptID
253508010	Terminal illness - late stage	162608008
283686014	Refer to terminal care consult	183569005
283721013	Refer for terminal care	183595007
447934018	Under care of palliative care physician	305496007
449190016	Referral to palliative care service	306237005
460928017	Palliative care	103735009
1488512015	Palliative medicine	394806003
1488778012	Palliative treatment	103735009

1488785011	Community specialist palliative care	395086005
1488791013	Specialist palliative care	395092004
1488802018	Final days pathway	395103003
1489327013	Specialist palliative care treatment - inpatient	395670002
1489349019	Specialist palliative care treatment - daycare	395694002
1489350019	Specialist palliative care treatment - outpatient	395695001
2534178018	On gold standards palliative care framework	414937009
3291966014	Has end of life care plan	713673000
3516617014	Palliative care medication review	1841000124106
3634081012	Palliative care Outcome Scale symptom list for end-stage renal disease score (observable entity)	761866001
63101000000114	Referred to community specialist palliative care team	25411000000109
306641000000111	Under care of palliative care service	201511000000105
403791000000114	Under the care of community palliative care team	246931000000107
480211000000115	Liverpool care pathway for the dying	873411000000105
631591000006116	Completion of DS1500<br>terminal illness medical report	514591000000108
641051000000112	End of life care	182964004
699911000000117	Management of multiple sclerosis in palliative phase	367681000000102
939921000006110	Primary care palliative care	939921000006106
980801000006113	End-of-life care	980801000006109
1059561000000114	Anticipatory palliative care	443761007
1137331000000111	Last days of life	511401000000102
1175491000000110	On end of life care register	526631000000108
1539541000006119	Heart failure monitoring - palliative care	1539541000006103
1672181000006115	Palliative care	1672181000006104
1715961000000114	Last days of life discussed	707988003
1740641000006110	Gold standards framework supportive<br>care stage 1 - advancing disease	518901000000101
1740651000006112	Gold standards framework supportive<br>care stage 2 - increasing decline	518941000000103
1740661000006114	Gold standards framework supportive<br>care stage 3 - last days: category C - weeks prognosis	519041000000106
1740671000006119	Gold standards framework supportive<br>care stage 3 - last days: category D - days prognosis	519061000000107
1752691000000114	Referral to community palliative care team declined	783841000000101
1753901000006113	Gold standards framework supportive care stage 3 - last days: category B - months prognosis	754401000000106
1844211000006119	Gold standards framework prognostic indicator stage B (green) - months prognosis	845721000000108
1844221000006110	Gold standards framework prognostic indicator stage C (yellow) - weeks prognosis	845751000000103
1844231000006113	Gold standards framework prognostic indicator stage D (red) - days prognosis	845771000000107
1845661000006119	Ambulance service notified of patient on end of life care register	854021000000106
1934771000006113	On electronic palliative care coordination system register	1934771000006109
1964511000006116	Palliative care phase of illness	1964511000006100
1964521000006112	Palliative care phase of illness - stable	1964521000006108
1964531000006110	Palliative care phase of illness - unstable	1964531000006106
1964541000006117	Palliative care phase of illness - deteriorating	1964541000006101
1964551000006115	Palliative care phase of illness - dying	1964551000006104
1964561000006118	Palliative care phase of illness - unknown	1964561000006102
2131431000000111	On Liverpool care pathway for the dying	818221000000103
2194521000000118	Palliative care handover form completed	846161000000108
2195881000000116	Current palliative oncology treatment	846841000000101
2197921000000117	End of life care pathway	847771000000100
2204781000000112	Under care of palliative care specialist nurse	850951000000107
2250601000000111	Prescription of palliative care anticipatory medication	871021000000106
2257971000000114	Integrated care priorities for end of life	874311000000104
2320981000000117	Palliative Care Problem Severity Score (observable entity)	902471000000105
2436201000000111	Last weeks of life	955231000000109
2465821000000115	Last months of life	968211000000101
2588971000000111	End of life care plan offered	1025611000000105
2611041000000116	Has end of life care key worker	719240009

2611081000000112	Has end of life care key general practitioner	1034241000000106
2611121000000110	Has end of life care key nurse	719238004
2733521000000119	Referral to end of life care service	1091311000000108
2737191000000115	Under care of hospice at home service	1092551000000109
2742951000000111	End of life advance care planning declined	1095091000000103
2743031000000110	Offer of end of life advance care plan review	1095131000000100
2743071000000112	Has end of life advance care plan	1095151000000107
2743091000000111	End of life advance care plan review declined	1095161000000105
4614671000006117	Palliative course of radiotherapy	168528006
5081281000006119	Palliative procedure for pancreatic pain relief	235513003
5939711000006116	Admission to palliative care department	305381007
5943751000006118	Seen by palliative care physician	305686008
5945581000006115	Seen by palliative care medicine service	305824005
5954251000006113	Referral to palliative care physician	306288008
6238721000006118	Palliative procedure	362964009
6249711000006119	Palliative procedure	363676003
6442711000006112	End-of-life care treatments and procedures	385736008
6557171000006110	Specialist palliative care treatment	395669003
7698451000006111	Palliative debulking of tumour for symptom relief	712999003
7699221000006110	End of life care planning	713058002
8053921000006113	Provision of palliative care in hospital, hospice or care home	193121000000109
8197121000006117	Palliative treatment of lymphoedema	526141000000106
8319091000006116	Palliative Care Problem Severity Score - pain (observable entity)	902501000000103
8319111000006113	Palliative Care Problem Severity Score - other symptoms (observable entity)	902511000000101
8319161000006111	Palliative Care Problem Severity Score - family/carer (observable entity)	902531000000109
11932591000006113	Palliative care phase of illness - dying (high complexity)	11932591000006108
11932641000006115	Palliative care phase of illness - dying (low complexity)	11932641000006104
12117321000006112	Assessment using Palliative Care Outcomes Collaboration assessment tools	1099101000000108
14811961000006113	On palliative care register	1403151000000103
[No records]	Assessment using Integrated Palliative care Outcome Scale Staff Version (3 day recall period)	718890006
[No records]	Assessment using Integrated Palliative care Outcome Scale 5 Patient Version (1 week recall period)	718893008
[No records]	Assessment using Integrated Palliative care Outcome Scale Staff Version (1 week recall period)	718895001
[No records]	Assessment using Integrated Palliative care Outcome Scale 5 Patient Version (3 day recall period)	718898004
[No records]	Assessment using Integrated Palliative care Outcome Scale Patient Version (3 day recall period)	718899007
[No records]	Assessment using Integrated Palliative care Outcome Scale 5 Staff Version (1 week recall period)	718901003
[No records]	Assessment using Integrated Palliative care Outcome Scale 5 Staff Version (3 day recall period)	718903000
[No records]	Assessment using Integrated Palliative care Outcome Scale Patient Version (1 week recall period)	718904006
[No records]	Liverpool care pathway for the dying patient declined	783941000000105
[No records]	Palliative Care Problem Severity Score - psychological/spiritual (observable entity)	902521000000107
[No records]	Palliative care phase measure phase (observable entity)	1092801000000106

Table S3: Clinical codes for health conditions, including from hospital episode statistics (HES)

Clinical codes for autism

GOLD: clinical, referral, tests; Aurum: consultations, observations.
Excludes Rett syndrome and other childhood disintegrative disorder (e.g. Heller syndrome)

Additional sources of information in addition to clinical opinion and in-house code repository (https://clinicalcodes.rss.mhs.man.ac.uk/medcodes/article/192/codelist/res192-autism_read/ and https://www.thelancet.com/cms/10.1016/j.eclinm.2022.101656/attachment/b9868933-63cc-4565-82ad-3947fa3b8302/mmc1.pdf)

CPRD GOLD: Autism		
**medcode**	**Readcode**	**readterm**
1276	E140.12	Autism
2950	Eu84500	[X]Asperger's syndrome
3637	Eu84000	[X]Childhood autism
7226	Eu84.00	[X]Pervasive developmental disorders
7302	E140.13	Childhood autism
9982	Eu84011	[X]Autistic disorder
22098	E140.00	Infantile autism
24044	Eu84100	[X]Atypical autism
36662	E140z00	Infantile autism NOS
42941	Eu84z11	[X]Autistic spectrum disorder
44327	Eu84z00	[X]Pervasive developmental disorder, unspecified
47948	Eu84y00	[X]Other pervasive developmental disorders
50337	Eu84012	[X]Infantile autism
51375	Eu84511	[X]Autistic psychopathy
63251	E140000	Active infantile autism
69016	E140100	Residual infantile autism
34174	Eu84112	[X]Mental retardation with autistic features

CPRD Aurum: Autism		
**medcode**	**term**	**snomedconceptID**
72723012	Childhood autism	43614003
294935010	Active infantile autism	191689008
294936011	Residual infantile autism	191690004
294937019	Infantile autism NOS	408857007
296678017	[X]Other pervasive developmental disorders	35919005
296679013	[X]Pervasive developmental disorder, unspecified	35919005
2535850012	Autistic spectrum disorder	35919005
2579633010	Autism	35919005
363931000006110	Asperger's syndrome	23560001
366531000006111	Atypical autism	231536004
366581000006112	Autistic disorder	408856003
366591000006110	[X]Autistic psychopathy	23560001
370801000006114	[X]Childhood autism	43614003
389671000006119	[X]Infantile autism	408857007
420581000006111	Autism spectrum disorder	35919005
781051000006117	Infantile autism	408857007
3078471000006111	Pervasive developmental disorder	35919005
3202381000006119	Autistic disorder of childhood onset	43614003
3202401000006119	Autism, childhood onset	43614003
5024421000006110	Atypical autistic syndrome	231536004
6383321000006111	Autistic spectrum disorder with isolated skills	373618009
6768081000006116	Autism	408856003
6768091000006118	Autism disorder	408856003
7565791000006114	High-functioning autism	702732007
13502801000006112	Autism epilepsy syndrome due to branched chain ketoacid dehydrogenase kinase deficiency	771448004
13503701000006112	Autism spectrum disorder due to AUTS2 deficiency	771512003
13963361000006116	Pervasive developmental disorder with disorder of intellectual development without loss of previously acquired skills	870262000
13963471000006120	Autism spectrum disorder with disorder of intellectual development and impaired functional language without loss of previously acquired skills	870267006
13964061000006120	Pervasive developmental disorder with cognitive developmental delay and marked impairment of functional language	870305003
13964071000006114	Autism spectrum disorder with disorder of intellectual development and impaired functional language	870305003

398241000006118	[X]Mental retardation with autistic features	231536004

Hospital episode statistics: Autism	
**icd**	**description**
F84.0	Childhood autism
F84.1	Atypical autism
F84.5	Asperger syndrome
F84.8	Other pervasive developmental disorders
F84.9	Pervasive developmental disorders, unspecified

Clinical codes for cancers

GOLD: clinical, referral, tests; Aurum: consultations, observations.
In situ, early-stage, juvenile carcinomas are not included in this definition.

Additional sources of information in addition to clinical opinion and in-house code repository: MiFoot study https://www.mifoot.org.uk/; Matthews et al. 2016: https://datacompass.lshtm.ac.uk/id/eprint/365/)

CPRD GOLD: Cancers		
**medcode**	**readcode**	**readterm**
10335	1O0..00	Cancer confirmed
32351	44a4.00	Squamous cell carcinoma antigen level
52946	4C53.00	Bone marrow: myeloma cells
10178	4M0..00	Gleason grading of prostate cancer
18503	4M00.00	Gleason prostate grade 2-4 (low)
18612	4M01.00	Gleason prostate grade 5-7 (medium)
26081	4M02.00	Gleason prostate grade 8-10 (high)
60918	4M20.00	Lymphoma stage I
94935	4M21.00	Lymphoma stage II
32240	4M22.00	Lymphoma stage III
71672	4M23.00	Lymphoma stage IV
37793	4M4..00	FIGO staging of gynaecological malignancy
101198	4M70.00	Clark melanoma level 1
104609	4M71.00	Clark melanoma level 2
96280	4M72.00	Clark melanoma level 3
102116	4M73.00	Clark melanoma level 4
30283	7B2C700	Intravesical install chemotherapeutic agent for malignancy
18270	7G03K00	Excision malignant skin tumour
11834	7G05600	Excision biopsy of rodent ulcer
93402	7G05D00	Excision biopsy of basal cell carcinoma
26197	8B3p.00	Administration of cancer treatment
5019	8BAD000	Cancer chemotherapy
32411	8BC6.00	Cancer treatment started
**54336**	8BCF.00	Cancer hospital treatment completed
10292	8CL0.00	Cancer diagnosis discussed
11075	8CL1.00	Cancer diagnosis discussed with significant other
22382	8CL2.00	Cancer diagnosis discussed with patient
44952	9Ok3.00	Date cancer diagnosis received in primary care
26076	9Ok5.00	Cancer pain and symptom management
59054	9Ok7.00	Cancer rehabilitation and readaption
90118	9OkC.00	Patient on regional cancer register
94000	9Ow1.00	Bowel cancer detected by national screening programme
101836	A788600	Human immunodeficiency virus with secondary cancers
67575	A788W00	HIV disease resulting in unspecified malignant neoplasm
27853	A789500	HIV disease resulting in Kaposi's sarcoma
108054	A789511	HIV disease resulting in Kaposi sarcoma
44617	A789600	HIV disease resulting in Burkitt's lymphoma
66367	A789700	HIV dis resulting oth types of non-Hodgkin's lymphoma
105324	A789800	HIV disease resulting in multiple malignant neoplasms
51708	A789X00	HIV dis reslt/oth mal neopl/lymph,h'matopoetc+reltd tissu
69767	AyuC600	[X]HIV disease resulting in other non-Hodgkin's lymphoma
112035	AyuC800	[X]HIV disease resulting in other malignant neoplasms
112036	AyuC900	[X]HIV disease resulting in unspecified malignant neoplasm
19415	B0...00	Malignant neoplasm of lip, oral cavity and pharynx
24374	B0...11	Carcinoma of lip, oral cavity and pharynx
14712	B00..00	Malignant neoplasm of lip
9984	B00..11	Carcinoma of lip
73962	B000.00	Malignant neoplasm of upper lip, vermilion border
66270	B000000	Malignant neoplasm of upper lip, external
50296	B000100	Malignant neoplasm of upper lip, lipstick area

98740	B000z00	Malignant neoplasm of upper lip, vermilion border NOS
67446	B001.00	Malignant neoplasm of lower lip, vermilion border
66384	B001000	Malignant neoplasm of lower lip, external
95480	B001100	Malignant neoplasm of lower lip, lipstick area
101707	B001z00	Malignant neoplasm of lower lip, vermilion border NOS
99493	B002.00	Malignant neoplasm of upper lip, inner aspect
111289	B002000	Malignant neoplasm of upper lip, buccal aspect
99001	B002100	Malignant neoplasm of upper lip, frenulum
98500	B002200	Malignant neoplasm of upper lip, mucosa
90610	B002300	Malignant neoplasm of upper lip, oral aspect
100721	B002z00	Malignant neoplasm of upper lip, inner aspect NOS
71147	B003.00	Malignant neoplasm of lower lip, inner aspect
67504	B003000	Malignant neoplasm of lower lip, buccal aspect
91843	B003100	Malignant neoplasm of lower lip, frenulum
89909	B003200	Malignant neoplasm of lower lip, mucosa
94441	B003300	Malignant neoplasm of lower lip, oral aspect
96782	B003z00	Malignant neoplasm of lower lip, inner aspect NOS
61692	B004.00	Malignant neoplasm of lip unspecified, inner aspect
73614	B004000	Malignant neoplasm of lip unspecified, buccal aspect
68399	B004200	Malignant neoplasm of lip unspecified, mucosa
100144	B004300	Malignant neoplasm of lip, oral aspect
96783	B005.00	Malignant neoplasm of commissure of lip
18882	B006.00	Malignant neoplasm of overlapping lesion of lip
37553	B007.00	Malignant neoplasm of lip, unspecified
100906	B00z000	Malignant neoplasm of lip, unspecified, external
94251	B00z100	Malignant neoplasm of lip, unspecified, lipstick area
69761	B00zz00	Malignant neoplasm of lip, vermilion border NOS
10283	B01..00	Malignant neoplasm of tongue
43431	B010.00	Malignant neoplasm of base of tongue
69671	B010.11	Malignant neoplasm of posterior third of tongue
34409	B010000	Malignant neoplasm of base of tongue dorsal surface
91035	B010z00	Malignant neoplasm of fixed part of tongue NOS
43642	B011.00	Malignant neoplasm of dorsal surface of tongue
107258	B011100	Malignant neoplasm of midline of tongue
43781	B011z00	Malignant neoplasm of dorsum of tongue NOS
36161	B012.00	Malignant neoplasm of tongue, tip and lateral border
62840	B013.00	Malignant neoplasm of ventral surface of tongue
102142	B013000	Malignant neoplasm of anterior 2/3 of tongue ventral surface
63979	B013100	Malignant neoplasm of frenulum linguae
38488	B013z00	Malignant neoplasm of ventral tongue surface NOS
58121	B014.00	Malignant neoplasm of anterior 2/3 of tongue unspecified
37096	B015.00	Malignant neoplasm of tongue, junctional zone
24852	B016.00	Malignant neoplasm of lingual tonsil
47205	B017.00	Malignant overlapping lesion of tongue
41530	B01y.00	Malignant neoplasm of other sites of tongue
40557	B01z.00	Malignant neoplasm of tongue NOS
20292	B02..00	Malignant neoplasm of major salivary glands
4388	B020.00	Malignant neoplasm of parotid gland
51786	B021.00	Malignant neoplasm of submandibular gland
70928	B022.00	Malignant neoplasm of sublingual gland
70696	B02y.00	Malignant neoplasm of other major salivary glands
50475	B02z.00	Malignant neoplasm of major salivary gland NOS
43400	B03..00	Malignant neoplasm of gum
32024	B030.00	Malignant neoplasm of upper gum
45408	B040.00	Malignant neoplasm of anterior portion of floor of mouth
45986	B041.00	Malignant neoplasm of lateral portion of floor of mouth
17912	B042.00	Malignant neoplasm, overlapping lesion of floor of mouth
56709	B04y.00	Malignant neoplasm of other sites of floor of mouth
36716	B04z.00	Malignant neoplasm of floor of mouth NOS
14792	B05..00	Malignant neoplasm of other and unspecified parts of mouth
31364	B050.00	Malignant neoplasm of cheek mucosa
30402	B050.11	Malignant neoplasm of buccal mucosa
103796	B051.00	Malignant neoplasm of vestibule of mouth
95772	B051000	Malignant neoplasm of upper buccal sulcus
97530	B051100	Malignant neoplasm of lower buccal sulcus
37590	B052.00	Malignant neoplasm of hard palate
40292	B053.00	Malignant neoplasm of soft palate
37516	B054.00	Malignant neoplasm of uvula
70819	B055.00	Malignant neoplasm of palate unspecified
96003	B055000	Malignant neoplasm of junction of hard and soft palate
69951	B055100	Malignant neoplasm of roof of mouth

28559	B055z00	Malignant neoplasm of palate NOS
37724	B056.00	Malignant neoplasm of retromolar area
37916	B05y.00	Malignant neoplasm of other specified mouth parts
55015	B05z.00	Malignant neoplasm of mouth NOS
37549	B05z000	Kaposi's sarcoma of palate
22893	B06..00	Malignant neoplasm of oropharynx
16241	B060.00	Malignant neoplasm of tonsil
26448	B060000	Malignant neoplasm of faucial tonsil
101988	B060100	Malignant neoplasm of palatine tonsil
102151	B060200	Malignant neoplasm of overlapping lesion of tonsil
53884	B060z00	Malignant neoplasm tonsil NOS
24397	B061.00	Malignant neoplasm of tonsillar fossa
55066	B062.00	Malignant neoplasm of tonsillar pillar
51926	B062000	Malignant neoplasm of faucial pillar
99185	B062100	Malignant neoplasm of glossopalatine fold
61510	B062200	Malignant neoplasm of palatoglossal arch
93842	B062300	Malignant neoplasm of palatopharyngeal arch
100002	B062z00	Malignant neoplasm of tonsillar fossa NOS
39554	B063.00	Malignant neoplasm of vallecula
46728	B064.00	Malignant neoplasm of anterior epiglottis
26134	B064000	Malignant neoplasm of epiglottis, free border
91895	B064100	Malignant neoplasm of glossoepiglottic fold
73439	B064z00	Malignant neoplasm of anterior epiglottis NOS
48519	B065.00	Malignant neoplasm of junctional region of epiglottis
56355	B066.00	Malignant neoplasm of lateral wall of oropharynx
90124	B067.00	Malignant neoplasm of posterior wall of oropharynx
67323	B06y.00	Malignant neoplasm of oropharynx, other specified sites
91037	B06yz00	Malignant neoplasm of other specified site of oropharynx NOS
43200	B06z.00	Malignant neoplasm of oropharynx NOS
24675	B07..00	Malignant neoplasm of nasopharynx
94390	B070.00	Malignant neoplasm of roof of nasopharynx
95429	B071.00	Malignant neoplasm of posterior wall of nasopharynx
33388	B071000	Malignant neoplasm of adenoid
46548	B071100	Malignant neoplasm of pharyngeal tonsil
96869	B071z00	Malignant neoplasm of posterior wall of nasopharynx NOS
59004	B072.00	Malignant neoplasm of lateral wall of nasopharynx
37940	B072000	Malignant neoplasm of pharyngeal recess
102205	B072z00	Malignant neoplasm of lateral wall of nasopharynx NOS
44139	B073.00	Malignant neoplasm of anterior wall of nasopharynx
106915	B073100	Malignant neoplasm of nasopharyngeal soft palate surface
99386	B073200	Malignant neoplasm posterior margin nasal septum and choanae
100918	B073z00	Malignant neoplasm of anterior wall of nasopharynx NOS
66422	B074.00	Malignant neoplasm, overlapping lesion of nasopharynx
55630	B07y.00	Malignant neoplasm of other specified site of nasopharynx
28665	B07z.00	Malignant neoplasm of nasopharynx NOS
34012	B08..00	Malignant neoplasm of hypopharynx
43548	B080.00	Malignant neoplasm of postcricoid region
39897	B081.00	Malignant neoplasm of pyriform sinus
57248	B082.00	Malignant neoplasm aryepiglottic fold, hypopharyngeal aspect
64462	B083.00	Malignant neoplasm of posterior pharynx
88362	B08y.00	Malignant neoplasm of other specified hypopharyngeal site
28451	B08z.00	Malignant neoplasm of hypopharynx NOS
46114	B0z..00	Malig neop other/ill-defined sites lip, oral cavity, pharynx
16297	B0z0.00	Malignant neoplasm of pharynx unspecified
95016	B0z1.00	Malignant neoplasm of Waldeyer's ring
39084	B0z2.00	Malignant neoplasm of laryngopharynx
49758	B0zy.00	Malignant neoplasm of other sites lip, oral cavity, pharynx
39430	B0zz.00	Malignant neoplasm of lip, oral cavity and pharynx NOS
15709	B1...00	Malignant neoplasm of digestive organs and peritoneum
3357	B1...11	Carcinoma of digestive organs and peritoneum
1062	B10..00	Malignant neoplasm of oesophagus
61695	B100.00	Malignant neoplasm of cervical oesophagus
41362	B101.00	Malignant neoplasm of thoracic oesophagus
63470	B102.00	Malignant neoplasm of abdominal oesophagus
50789	B103.00	Malignant neoplasm of upper third of oesophagus
54171	B104.00	Malignant neoplasm of middle third of oesophagus
42416	B105.00	Malignant neoplasm of lower third of oesophagus
67497	B106.00	Malignant neoplasm, overlapping lesion of oesophagus
98142	B107.00	Siewert type I adenocarcinoma
53591	B10y.00	Malignant neoplasm of other specified part of oesophagus
30700	B10z.00	Malignant neoplasm of oesophagus NOS

4865	B10z.11	Oesophageal cancer
8386	B11..00	Malignant neoplasm of stomach
32022	B110.00	Malignant neoplasm of cardia of stomach
100584	B110000	Malignant neoplasm of cardiac orifice of stomach
22894	B110100	Malignant neoplasm of cardio-oesophageal junction of stomach
94278	B110111	Malignant neoplasm of gastro-oesophageal junction
37859	B110z00	Malignant neoplasm of cardia of stomach NOS
21620	B111.00	Malignant neoplasm of pylorus of stomach
48237	B111000	Malignant neoplasm of prepylorus of stomach
41215	B111100	Malignant neoplasm of pyloric canal of stomach
59092	B111z00	Malignant neoplasm of pylorus of stomach NOS
19318	B112.00	Malignant neoplasm of pyloric antrum of stomach
32362	B113.00	Malignant neoplasm of fundus of stomach
43572	B114.00	Malignant neoplasm of body of stomach
42193	B115.00	Malignant neoplasm of lesser curve of stomach unspecified
55434	B116.00	Malignant neoplasm of greater curve of stomach unspecified
51690	B117.00	Malignant neoplasm, overlapping lesion of stomach
97499	B118.00	Siewert type II adenocarcinoma
96094	B119.00	Siewert type III adenocarcinoma
55019	B11y.00	Malignant neoplasm of other specified site of stomach
65312	B11y000	Malignant neoplasm of anterior wall of stomach NEC
96802	B11y100	Malignant neoplasm of posterior wall of stomach NEC
65372	B11yz00	Malignant neoplasm of other specified site of stomach NOS
14800	B11z.00	Malignant neoplasm of stomach NOS
6806	B12..00	Malignant neoplasm of small intestine and duodenum
18613	B120.00	Malignant neoplasm of duodenum
43479	B121.00	Malignant neoplasm of jejunum
33871	B122.00	Malignant neoplasm of ileum
63995	B123.00	Malignant neoplasm of Meckel's diverticulum
66166	B124.00	Malignant neoplasm, overlapping lesion of small intestine
99896	B12y.00	Malignant neoplasm of other specified site small intestine
43390	B12z.00	Malignant neoplasm of small intestine NOS
1220	B13..00	Malignant neoplasm of colon
9088	B130.00	Malignant neoplasm of hepatic flexure of colon
6935	B131.00	Malignant neoplasm of transverse colon
10864	B132.00	Malignant neoplasm of descending colon
2815	B133.00	Malignant neoplasm of sigmoid colon
3811	B134.00	Malignant neoplasm of caecum
22163	B134.11	Carcinoma of caecum
18632	B135.00	Malignant neoplasm of appendix
10946	B136.00	Malignant neoplasm of ascending colon
18619	B137.00	Malignant neoplasm of splenic flexure of colon
93478	B138.00	Malignant neoplasm, overlapping lesion of colon
101700	B139.00	Hereditary nonpolyposis colon cancer
48231	B13y.00	Malignant neoplasm of other specified sites of colon
28163	B13z.00	Malignant neoplasm of colon NOS
9118	B13z.11	Colonic cancer
35357	B14..00	Malignant neoplasm of rectum, rectosigmoid junction and anus
27855	B140.00	Malignant neoplasm of rectosigmoid junction
1800	B141.00	Malignant neoplasm of rectum
7219	B141.11	Carcinoma of rectum
5901	B141.12	Rectal carcinoma
24370	B142.00	Malignant neoplasm of anal canal
9491	B142.11	Anal carcinoma
46159	B142000	Malignant neoplasm of cloacogenic zone
27897	B143.00	Malignant neoplasm of anus unspecified
55659	B14y.00	Malig neop other site rectum, rectosigmoid junction and anus
50974	B14z.00	Malignant neoplasm rectum,rectosigmoid junction and anus NOS
8918	B15..00	Malignant neoplasm of liver and intrahepatic bile ducts
25535	B150.00	Primary malignant neoplasm of liver
16126	B150000	Primary carcinoma of liver
31210	B150100	Hepatoblastoma of liver
68410	B150200	Primary angiosarcoma of liver
22187	B150300	Hepatocellular carcinoma
44399	B150z00	Primary malignant neoplasm of liver NOS
16915	B151.00	Malignant neoplasm of intrahepatic bile ducts
65124	B151000	Malignant neoplasm of interlobular bile ducts
110775	B151100	Malignant neoplasm of interlobular biliary canals
89593	B151200	Malignant neoplasm of intrahepatic biliary passages
58088	B151400	Malignant neoplasm of intrahepatic gall duct
61643	B151z00	Malignant neoplasm of intrahepatic bile ducts NOS

26393	B152.00	Malignant neoplasm of liver unspecified
36147	B153.00	Secondary malignant neoplasm of liver
38978	B15z.00	Malignant neoplasm of liver and intrahepatic bile ducts NOS
54103	B16..00	Malignant neoplasm gallbladder and extrahepatic bile ducts
16105	B160.00	Malignant neoplasm of gallbladder
31393	B160.11	Carcinoma gallbladder
23433	B161.00	Malignant neoplasm of extrahepatic bile ducts
72445	B161000	Malignant neoplasm of cystic duct
52537	B161100	Malignant neoplasm of hepatic duct
7982	B161200	Malignant neoplasm of common bile duct
36495	B161211	Carcinoma common bile duct
105613	B161300	Malignant neoplasm of sphincter of Oddi
74896	B161z00	Malignant neoplasm of extrahepatic bile ducts NOS
10949	B162.00	Malignant neoplasm of ampulla of Vater
35039	B163.00	Malignant neoplasm, overlapping lesion of biliary tract
60312	B16y.00	Malignant neoplasm other gallbladder/extrahepatic bile duct
15907	B16z.00	Malignant neoplasm gallbladder/extrahepatic bile ducts NOS
8166	B17..00	Malignant neoplasm of pancreas
8771	B170.00	Malignant neoplasm of head of pancreas
40810	B171.00	Malignant neoplasm of body of pancreas
39870	B172.00	Malignant neoplasm of tail of pancreas
35535	B173.00	Malignant neoplasm of pancreatic duct
35795	B174.00	Malignant neoplasm of Islets of Langerhans
97875	B175.00	Malignant neoplasm, overlapping lesion of pancreas
109782	B176.00	Somatostatinoma of pancreas
48537	B17y.00	Malignant neoplasm of other specified sites of pancreas
96635	B17y000	Malignant neoplasm of ectopic pancreatic tissue
95783	B17yz00	Malignant neoplasm of specified site of pancreas NOS
34388	B17z.00	Malignant neoplasm of pancreas NOS
44108	B18..00	Malignant neoplasm of retroperitoneum and peritoneum
21330	B180.00	Malignant neoplasm of retroperitoneum
65159	B180100	Malignant neoplasm of perinephric tissue
24048	B180200	Malignant neoplasm of retrocaecal tissue
61555	B180z00	Malignant neoplasm of retroperitoneum NOS
17874	B181.00	Mesothelioma of peritoneum
101907	B182.00	Overlapping malign lesion of retroperitoneum and peritoneum
46613	B18y.00	Malignant neoplasm of specified parts of peritoneum
59388	B18y100	Malignant neoplasm of mesocaecum
30165	B18y200	Malignant neoplasm of mesorectum
50898	B18y300	Malignant neoplasm of omentum
64516	B18y400	Malignant neoplasm of parietal peritoneum
39413	B18y500	Malignant neoplasm of pelvic peritoneum
69821	B18y600	Malignant neoplasm of the pouch of Douglas
90290	B18y700	Malignant neoplasm of mesentery
64106	B18yz00	Malignant neoplasm of specified parts of peritoneum NOS
16298	B18z.00	Malignant neoplasm of retroperitoneum and peritoneum NOS
11009	B1z..00	Malig neop oth/ill-defined sites digestive tract/peritoneum
17559	B1z0.00	Malignant neoplasm of intestinal tract, part unspecified
11628	B1z0.11	Cancer of bowel
65460	B1z1.00	Malignant neoplasm of spleen NEC
108667	B1z1000	Angiosarcoma of spleen
72224	B1z1100	Fibrosarcoma of spleen
93778	B1z1z00	Malignant neoplasm of spleen NOS
94776	B1z2.00	Malignant neoplasm, overlapping lesion of digestive system
56918	B1zy.00	Malignant neoplasm other spec digestive tract and peritoneum
51255	B1zz.00	Malignant neoplasm of digestive tract and peritoneum NOS
34075	B2...00	Malig neop of respiratory tract and intrathoracic organs
45307	B2...11	Carcinoma of respiratory tract and intrathoracic organs
26652	B20..00	Malig neop nasal cavities, middle ear and accessory sinuses
23389	B200.00	Malignant neoplasm of nasal cavities
71204	B200000	Malignant neoplasm of cartilage of nose
98911	B200100	Malignant neoplasm of nasal conchae
62761	B200200	Malignant neoplasm of septum of nose
62182	B200300	Malignant neoplasm of vestibule of nose
42856	B200z00	Malignant neoplasm of nasal cavities NOS
24456	B201.00	Malig neop auditory tube, middle ear and mastoid air cells
107916	B201000	Malignant neoplasm of auditory (Eustachian) tube
98537	B201100	Malignant neoplasm of tympanic cavity
54613	B201200	Malignant neoplasm of tympanic antrum
71946	B201300	Malignant neoplasm of mastoid air cells
73537	B201z00	Malig neop auditory tube, middle ear, mastoid air cells NOS

32174	B202.00	Malignant neoplasm of maxillary sinus
54636	B203.00	Malignant neoplasm of ethmoid sinus
15684	B204.00	Malignant neoplasm of frontal sinus
65215	B205.00	Malignant neoplasm of sphenoidal sinus
39590	B206.00	Malignant neoplasm, overlapping lesion of accessory sinuses
96971	B20y.00	Malig neop other site nasal cavity, middle ear and sinuses
55246	B20z.00	Malignant neoplasm of accessory sinus NOS
319	B21..00	Malignant neoplasm of larynx
318	B210.00	Malignant neoplasm of glottis
26165	B211.00	Malignant neoplasm of supraglottis
22441	B212.00	Malignant neoplasm of subglottis
43111	B213.00	Malignant neoplasm of laryngeal cartilage
63460	B213000	Malignant neoplasm of arytenoid cartilage
37805	B213100	Malignant neoplasm of cricoid cartilage
107878	B213200	Malignant neoplasm of cuneiform cartilage
47862	B213300	Malignant neoplasm of thyroid cartilage
97332	B213z00	Malignant neoplasm of laryngeal cartilage NOS
50579	B214.00	Malignant neoplasm, overlapping lesion of larynx
55374	B215.00	Malignant neoplasm of epiglottis NOS
26813	B21y.00	Malignant neoplasm of larynx, other specified site
9237	B21z.00	Malignant neoplasm of larynx NOS
13243	B22..00	Malignant neoplasm of trachea, bronchus and lung
15221	B220.00	Malignant neoplasm of trachea
103946	B220100	Malignant neoplasm of mucosa of trachea
37810	B220z00	Malignant neoplasm of trachea NOS
12870	B221.00	Malignant neoplasm of main bronchus
17391	B221000	Malignant neoplasm of carina of bronchus
33444	B221100	Malignant neoplasm of hilus of lung
21698	B221z00	Malignant neoplasm of main bronchus NOS
10358	B222.00	Malignant neoplasm of upper lobe, bronchus or lung
20170	B222.11	Pancoast's syndrome
31700	B222000	Malignant neoplasm of upper lobe bronchus
25886	B222100	Malignant neoplasm of upper lobe of lung
44169	B222z00	Malignant neoplasm of upper lobe, bronchus or lung NOS
31268	B223.00	Malignant neoplasm of middle lobe, bronchus or lung
41523	B223000	Malignant neoplasm of middle lobe bronchus
39923	B223100	Malignant neoplasm of middle lobe of lung
54134	B223z00	Malignant neoplasm of middle lobe, bronchus or lung NOS
31188	B224.00	Malignant neoplasm of lower lobe, bronchus or lung
18678	B224000	Malignant neoplasm of lower lobe bronchus
12582	B224100	Malignant neoplasm of lower lobe of lung
42566	B224z00	Malignant neoplasm of lower lobe, bronchus or lung NOS
36371	B225.00	Malignant neoplasm of overlapping lesion of bronchus & lung
7484	B226.00	Mesothelioma
38961	B22y.00	Malignant neoplasm of other sites of bronchus or lung
3903	B22z.00	Malignant neoplasm of bronchus or lung NOS
2587	B22z.11	Lung cancer
31573	B23..00	Malignant neoplasm of pleura
67107	B230.00	Malignant neoplasm of parietal pleura
106194	B231.00	Malignant neoplasm of visceral pleura
9600	B232.00	Mesothelioma of pleura
98104	B23y.00	Malignant neoplasm of other specified pleura
34742	B23z.00	Malignant neoplasm of pleura NOS
62556	B24..00	Malignant neoplasm of thymus, heart and mediastinum
27483	B240.00	Malignant neoplasm of thymus
95644	B241.00	Malignant neoplasm of heart
63430	B241000	Malignant neoplasm of endocardium
65605	B241200	Malignant neoplasm of myocardium
94975	B241300	Malignant neoplasm of pericardium
101885	B241400	Mesothelioma of pericardium
50289	B241z00	Malignant neoplasm of heart NOS
27715	B242.00	Malignant neoplasm of anterior mediastinum
92720	B243.00	Malignant neoplasm of posterior mediastinum
61064	B24X.00	Malignant neoplasm of mediastinum, part unspecified
100232	B24y.00	Malig neop of other site of heart, thymus and mediastinum
66750	B24z.00	Malignant neoplasm of heart, thymus and mediastinum NOS
39531	B25..00	Malig neo, overlapping lesion of heart, mediastinum & pleura
66646	B26..00	Malignant neoplasm, overlap lesion of resp & intrathor orgs
44356	B2z..00	Malig neop other/ill-defined sites resp/intrathoracic organs
65793	B2z0.00	Malig neop of upper respiratory tract, part unspecified
29283	B2zy.00	Malignant neoplasm of other site of respiratory tract

42569	B2zz.00	Malignant neoplasm of respiratory tract NOS
18608	B3...00	Malig neop of bone, connective tissue, skin and breast
9902	B3...11	Carcinoma of bone, connective tissue, skin and breast
12539	B3...12	Sarcoma of bone and connective tissue
18314	B30..00	Malignant neoplasm of bone and articular cartilage
59036	B300.00	Malignant neoplasm of bones of skull and face
53594	B300000	Malignant neoplasm of ethmoid bone
53599	B300100	Malignant neoplasm of frontal bone
59520	B300200	Malignant neoplasm of malar bone
95458	B300300	Malignant neoplasm of nasal bone
55953	B300400	Malignant neoplasm of occipital bone
50298	B300500	Malignant neoplasm of orbital bone
54747	B300600	Malignant neoplasm of parietal bone
55595	B300700	Malignant neoplasm of sphenoid bone
62104	B300800	Malignant neoplasm of temporal bone
50299	B300900	Malignant neoplasm of zygomatic bone
17475	B300A00	Malignant neoplasm of maxilla
96445	B300B00	Malignant neoplasm of turbinate
44452	B300C00	Malignant neoplasm of vomer
69146	B300z00	Malignant neoplasm of bones of skull and face NOS
33833	B301.00	Malignant neoplasm of mandible
16704	B302.00	Malignant neoplasm of vertebral column
46939	B302000	Malignant neoplasm of cervical vertebra
32372	B302100	Malignant neoplasm of thoracic vertebra
54691	B302200	Malignant neoplasm of lumbar vertebra
49701	B302z00	Malignant neoplasm of vertebral column NOS
27528	B303.00	Malignant neoplasm of ribs, sternum and clavicle
37842	B303000	Malignant neoplasm of rib
49491	B303100	Malignant neoplasm of sternum
66639	B303200	Malignant neoplasm of clavicle
60403	B303300	Malignant neoplasm of costal cartilage
67763	B303400	Malignant neoplasm of costo-vertebral joint
54493	B303500	Malignant neoplasm of xiphoid process
51237	B303z00	Malignant neoplasm of rib, sternum and clavicle NOS
71810	B304.00	Malignant neoplasm of scapula and long bones of upper arm
49054	B304000	Malignant neoplasm of scapula
105797	B304100	Malignant neoplasm of acromion
61741	B304200	Malignant neoplasm of humerus
92371	B304300	Malignant neoplasm of radius
64848	B304400	Malignant neoplasm of ulna
65880	B304z00	Malig neop of scapula and long bones of upper arm NOS
73530	B305.00	Malignant neoplasm of hand bones
106069	B305.11	Malignant neoplasm of carpal bones
72464	B305.12	Malignant neoplasm of metacarpal bones
57988	B305000	Malignant neoplasm of carpal bone - scaphoid
69104	B305100	Malignant neoplasm of carpal bone - lunate
110993	B305800	Malignant neoplasm of first metacarpal bone
108638	B305A00	Malignant neoplasm of third metacarpal bone
94427	B305C00	Malignant neoplasm of fifth metacarpal bone
86812	B305D00	Malignant neoplasm of phalanges of hand
73556	B305z00	Malignant neoplasm of hand bones NOS
54631	B306.00	Malignant neoplasm of pelvic bones, sacrum and coccyx
44609	B306000	Malignant neoplasm of ilium
59223	B306100	Malignant neoplasm of ischium
51921	B306200	Malignant neoplasm of pubis
40966	B306300	Malignant neoplasm of sacral vertebra
66908	B306400	Malignant neoplasm of coccygeal vertebra
50152	B306500	Malignant sacral teratoma
38938	B306z00	Malignant neoplasm of pelvis, sacrum or coccyx NOS
68055	B307.00	Malignant neoplasm of long bones of leg
56513	B307000	Malignant neoplasm of femur
50402	B307100	Malignant neoplasm of fibula
40814	B307200	Malignant neoplasm of tibia
62630	B307z00	Malignant neoplasm of long bones of leg NOS
105475	B308.00	Malignant neoplasm of short bones of leg
111779	B308000	Malignant neoplasm of patella
95182	B308100	Malignant neoplasm of talus
72212	B308200	Malignant neoplasm of calcaneum
34878	B308300	Malignant neoplasm of medial cuneiform
69927	B308800	Malignant neoplasm of first metatarsal bone
111426	B308900	Malignant neoplasm of second metatarsal bone

92382	B308B00	Malignant neoplasm of fourth metatarsal bone
58949	B308D00	Malignant neoplasm of phalanges of foot
103354	B308z00	Malignant neoplasm of short bones of leg NOS
67451	B30W.00	Malignant neoplasm/overlap lesion/bone+articulr cartilage
43614	B30X.00	Malignant neoplasm/bones+articular cartilage/limb,unspfd
16075	B30z.00	Malignant neoplasm of bone and articular cartilage NOS
19437	B30z000	Osteosarcoma
34451	B31..00	Malignant neoplasm of connective and other soft tissue
43475	B310.00	Malig neop of connective and soft tissue head, face and neck
59382	B310000	Malignant neoplasm of soft tissue of head
40014	B310100	Malignant neoplasm of soft tissue of face
48517	B310200	Malignant neoplasm of soft tissue of neck
60035	B310300	Malignant neoplasm of cartilage of ear
49463	B310400	Malignant neoplasm of tarsus of eyelid
108389	B310500	Malignant neoplasm soft tissues of cervical spine
73718	B310z00	Malig neop connective and soft tissue head, face, neck NOS
53989	B311.00	Malig neop connective and soft tissue upper limb/shoulder
50222	B311000	Malignant neoplasm of connective and soft tissue of shoulder
64345	B311100	Malignant neoplasm of connective and soft tissue, upper arm
57482	B311200	Malignant neoplasm of connective and soft tissue of fore-arm
19321	B311300	Malignant neoplasm of connective and soft tissue of hand
91586	B311400	Malignant neoplasm of connective and soft tissue of finger
63988	B311500	Malignant neoplasm of connective and soft tissue of thumb
104913	B311z00	Malig neop connective soft tissue upper limb/shoulder NOS
66088	B312.00	Malig neop of connective and soft tissue of hip and leg
102949	B312000	Malignant neoplasm of connective and soft tissue of hip
44805	B312100	Malig neop of connective and soft tissue thigh and upper leg
54965	B312200	Malig neop connective and soft tissue of popliteal space
30542	B312300	Malig neop of connective and soft tissue of lower leg
54222	B312400	Malignant neoplasm of connective and soft tissue of foot
99572	B312500	Malignant neoplasm of connective and soft tissue of toe
90546	B312z00	Malig neop connective and soft tissue hip and leg NOS
22290	B313.00	Malignant neoplasm of connective and soft tissue of thorax
29160	B313000	Malignant neoplasm of connective and soft tissue of axilla
54186	B313100	Malignant neoplasm of diaphragm
72522	B313200	Malignant neoplasm of great vessels
104139	B313300	Malig neoplasm of connective and soft tissues of thor spine
98408	B313z00	Malig neop of connective and soft tissue of thorax NOS
45071	B314.00	Malignant neoplasm of connective and soft tissue of abdomen
66488	B314000	Malig neop of connective and soft tissue of abdominal wall
94272	B314100	Malig neoplasm of connective and soft tissues of lumb spine
60247	B314z00	Malig neop of connective and soft tissue of abdomen NOS
51965	B315.00	Malignant neoplasm of connective and soft tissue of pelvis
70463	B315000	Malignant neoplasm of connective and soft tissue of buttock
67324	B315100	Malig neop of connective and soft tissue of inguinal region
59152	B315200	Malignant neoplasm of connective and soft tissue of perineum
110192	B315300	Malig neopl of connective and soft tissue - sacrum or coccyx
58836	B315z00	Malig neop of connective and soft tissue of pelvis NOS
57471	B316.00	Malig neop of connective and soft tissue trunk unspecified
111311	B317.00	Malignant neoplasm, overlap lesion connective & soft tissue
65233	B31y.00	Malig neop connective and soft tissue other specified site
15182	B31z.00	Malignant neoplasm of connective and soft tissue, site NOS
104128	B31z000	Kaposi's sarcoma of soft tissue
865	B32..00	Malignant melanoma of skin
70637	B320.00	Malignant melanoma of lip
54632	B321.00	Malignant melanoma of eyelid including canthus
57260	B322.00	Malignant melanoma of ear and external auricular canal
59061	B322000	Malignant melanoma of auricle (ear)
102145	B322100	Malignant melanoma of external auditory meatus
73744	B322z00	Malignant melanoma of ear and external auricular canal NOS
47252	B323.00	Malignant melanoma of other and unspecified parts of face
41278	B323000	Malignant melanoma of external surface of cheek
71136	B323100	Malignant melanoma of chin
47094	B323200	Malignant melanoma of eyebrow
68133	B323300	Malignant melanoma of forehead
45139	B323400	Malignant melanoma of external surface of nose
58958	B323500	Malignant melanoma of temple
67806	B323z00	Malignant melanoma of face NOS
65625	B324.00	Malignant melanoma of scalp and neck
55881	B324000	Malignant melanoma of scalp
45306	B324100	Malignant melanoma of neck

99257	B324z00	Malignant melanoma of scalp and neck NOS
38689	B325.00	Malignant melanoma of trunk (excluding scrotum)
49814	B325000	Malignant melanoma of axilla
32768	B325100	Malignant melanoma of breast
53629	B325200	Malignant melanoma of buttock
34259	B325300	Malignant melanoma of groin
109002	B325400	Malignant melanoma of perianal skin
95629	B325500	Malignant melanoma of perineum
43715	B325600	Malignant melanoma of umbilicus
43463	B325700	Malignant melanoma of back
51209	B325800	Malignant melanoma of chest wall
45760	B325z00	Malignant melanoma of trunk, excluding scrotum, NOS
65164	B326.00	Malignant melanoma of upper limb and shoulder
50505	B326000	Malignant melanoma of shoulder
54685	B326100	Malignant melanoma of upper arm
45755	B326200	Malignant melanoma of fore-arm
62475	B326300	Malignant melanoma of hand
25602	B326400	Malignant melanoma of finger
63997	B326500	Malignant melanoma of thumb
55292	B326z00	Malignant melanoma of upper limb or shoulder NOS
46255	B327.00	Malignant melanoma of lower limb and hip
73536	B327000	Malignant melanoma of hip
51873	B327100	Malignant melanoma of thigh
54305	B327200	Malignant melanoma of knee
39878	B327300	Malignant melanoma of popliteal fossa area
37872	B327400	Malignant melanoma of lower leg
42714	B327500	Malignant melanoma of ankle
61246	B327600	Malignant melanoma of heel
41490	B327700	Malignant melanoma of foot
36899	B327800	Malignant melanoma of toe
53369	B327900	Malignant melanoma of great toe
64327	B327z00	Malignant melanoma of lower limb or hip NOS
111079	B328.00	Malignant melanoma stage IA
109827	B329.00	Malignant melanoma stage IB
110139	B32A.00	Malignant melanoma stage IIA
110180	B32B.00	Malignant melanoma stage IIB
111413	B32C.00	Malignant melanoma stage IIC
111672	B32D.00	Malignant melanoma stage IIIA
111943	B32E.00	Malignant melanoma stage IIIB
110961	B32F.00	Malignant melanoma stage IIIC
109745	B32G.00	Malignant melanoma stage IV M1a
110483	B32H.00	Malignant melanoma stage IV M1b
111162	B32J.00	Malignant melanoma stage IV M1c
42153	B32y.00	Malignant melanoma of other specified skin site
96585	B32y000	Overlapping malignant melanoma of skin
28556	B32z.00	Malignant melanoma of skin NOS
4632	B33..00	Other malignant neoplasm of skin
876	B33..11	Basal cell carcinoma
1940	B33..13	Rodent ulcer
37016	B33..14	Malignant neoplasm of sebaceous gland
40443	B33..15	Malignant neoplasm of sweat gland
3445	B33..16	Epithelioma basal cell
18245	B330.00	Malignant neoplasm of skin of lip
43087	B331.00	Malignant neoplasm of eyelid including canthus
36731	B331000	Malignant neoplasm of canthus
55550	B331100	Malignant neoplasm of upper eyelid
41958	B331200	Malignant neoplasm of lower eyelid
53515	B332.00	Malignant neoplasm skin of ear and external auricular canal
33997	B332000	Malignant neoplasm of skin of auricle (ear)
62080	B332100	Malignant neoplasm of skin of external auditory meatus
33271	B332200	Malignant neoplasm of pinna NEC
62399	B332z00	Malig neop skin of ear and external auricular canal NOS
27370	B333.00	Malignant neoplasm skin of other and unspecified parts face
30645	B333000	Malignant neoplasm of skin of cheek, external
49403	B333100	Malignant neoplasm of skin of chin
55670	B333200	Malignant neoplasm of skin of eyebrow
30576	B333300	Malignant neoplasm of skin of forehead
16202	B333400	Malignant neoplasm of skin of nose (external)
21327	B333500	Malignant neoplasm of skin of temple
46008	B333z00	Malignant neoplasm skin other and unspec part of face NOS
54234	B334.00	Malignant neoplasm of scalp and skin of neck

37165	B334000	Malignant neoplasm of scalp
43619	B334100	Malignant neoplasm of skin of neck
73760	B334z00	Malignant neoplasm of scalp or skin of neck NOS
57446	B335.00	Malignant neoplasm of skin of trunk, excluding scrotum
70380	B335000	Malignant neoplasm of skin of axillary fold
37969	B335100	Malignant neoplasm of skin of chest, excluding breast
30543	B335200	Malignant neoplasm of skin of breast
18618	B335300	Malignant neoplasm of skin of abdominal wall
67748	B335400	Malignant neoplasm of skin of umbilicus
66319	B335500	Malignant neoplasm of skin of groin
46458	B335600	Malignant neoplasm of skin of perineum
45077	B335700	Malignant neoplasm of skin of back
62305	B335800	Malignant neoplasm of skin of buttock
23480	B335900	Malignant neoplasm of perianal skin
66447	B335A00	Malignant neoplasm of skin of scapular region
15868	B335z00	Malignant neoplasm of skin of trunk, excluding scrotum, NOS
30747	B336.00	Malignant neoplasm of skin of upper limb and shoulder
43122	B336000	Malignant neoplasm of skin of shoulder
42707	B336100	Malignant neoplasm of skin of upper arm
30577	B336200	Malignant neoplasm of skin of fore-arm
54352	B336300	Malignant neoplasm of skin of hand
25245	B336400	Malignant neoplasm of skin of finger
64406	B336500	Malignant neoplasm of skin of thumb
60526	B336z00	Malignant neoplasm of skin of upper limb or shoulder NOS
57442	B337.00	Malignant neoplasm of skin of lower limb and hip
70988	B337000	Malignant neoplasm of skin of hip
58601	B337100	Malignant neoplasm of skin of thigh
56954	B337200	Malignant neoplasm of skin of knee
68197	B337300	Malignant neoplasm of skin of popliteal fossa area
33682	B337400	Malignant neoplasm of skin of lower leg
64270	B337500	Malignant neoplasm of skin of ankle
104025	B337600	Malignant neoplasm of skin of heel
70587	B337700	Malignant neoplasm of skin of foot
65782	B337800	Malignant neoplasm of skin of toe
67914	B337900	Malignant neoplasm of skin of great toe
61194	B337z00	Malignant neoplasm of skin of lower limb or hip NOS
93352	B338.00	Squamous cell carcinoma of skin
24375	B339.00	Dermatofibrosarcoma protuberans
42429	B33X.00	Malignant neoplasm overlapping lesion of skin
18354	B33y.00	Malignant neoplasm of other specified skin sites
2492	B33z.00	Malignant neoplasm of skin NOS
93490	B33z.11	Squamous cell carcinoma of skin NOS
27931	B33z000	Kaposi's sarcoma of skin
3968	B34..00	Malignant neoplasm of female breast
348	B34..11	Ca female breast
26853	B340.00	Malignant neoplasm of nipple and areola of female breast
23380	B340000	Malignant neoplasm of nipple of female breast
64686	B340100	Malignant neoplasm of areola of female breast
59831	B340z00	Malignant neoplasm of nipple or areola of female breast NOS
31546	B341.00	Malignant neoplasm of central part of female breast
29826	B342.00	Malignant neoplasm of upper-inner quadrant of female breast
45222	B343.00	Malignant neoplasm of lower-inner quadrant of female breast
23399	B344.00	Malignant neoplasm of upper-outer quadrant of female breast
42070	B345.00	Malignant neoplasm of lower-outer quadrant of female breast
20685	B346.00	Malignant neoplasm of axillary tail of female breast
49148	B347.00	Malignant neoplasm, overlapping lesion of breast
56715	B34y.00	Malignant neoplasm of other site of female breast
95057	B34y000	Malignant neoplasm of ectopic site of female breast
38475	B34yz00	Malignant neoplasm of other site of female breast NOS
9470	B34z.00	Malignant neoplasm of female breast NOS
19423	B35..00	Malignant neoplasm of male breast
54494	B350.00	Malignant neoplasm of nipple and areola of male breast
68480	B350000	Malignant neoplasm of nipple of male breast
67884	B350100	Malignant neoplasm of areola of male breast
54202	B35z.00	Malignant neoplasm of other site of male breast
95323	B35z000	Malignant neoplasm of ectopic site of male breast
48809	B35zz00	Malignant neoplasm of male breast NOS
105488	B36..00	Local recurrence of malignant tumour of breast
19389	B3y..00	Malig neop of bone, connective tissue, skin and breast OS
41011	B3z..00	Malig neop of bone, connective tissue, skin and breast NOS
13252	B4...00	Malignant neoplasm of genitourinary organ

16874	B4...11	Carcinoma of genitourinary organ
2744	B40..00	Malignant neoplasm of uterus, part unspecified
2747	B41..00	Malignant neoplasm of cervix uteri
3230	B41..11	Cervical carcinoma (uterus)
48820	B410.00	Malignant neoplasm of endocervix
57235	B410000	Malignant neoplasm of endocervical canal
53103	B410100	Malignant neoplasm of endocervical gland
50285	B410z00	Malignant neoplasm of endocervix NOS
50297	B411.00	Malignant neoplasm of exocervix
58094	B412.00	Malignant neoplasm, overlapping lesion of cervix uteri
32955	B41y.00	Malignant neoplasm of other site of cervix
95505	B41y000	Malignant neoplasm of cervical stump
57719	B41y100	Malignant neoplasm of squamocolumnar junction of cervix
43435	B41yz00	Malignant neoplasm of other site of cervix NOS
28311	B41z.00	Malignant neoplasm of cervix uteri NOS
93762	B42..00	Malignant neoplasm of placenta
28003	B420.00	Choriocarcinoma
7046	B43..00	Malignant neoplasm of body of uterus
3213	B430.00	Malignant neoplasm of corpus uteri, excluding isthmus
72723	B430000	Malignant neoplasm of cornu of corpus uteri
68155	B430100	Malignant neoplasm of fundus of corpus uteri
2890	B430200	Malignant neoplasm of endometrium of corpus uteri
49400	B430211	Malignant neoplasm of endometrium
45793	B430300	Malignant neoplasm of myometrium of corpus uteri
45490	B430z00	Malignant neoplasm of corpus uteri NOS
43940	B431.00	Malignant neoplasm of isthmus of uterine body
59097	B431000	Malignant neoplasm of lower uterine segment
70729	B431z00	Malignant neoplasm of isthmus of uterine body NOS
16967	B432.00	Malignant neoplasm of overlapping lesion of corpus uteri
31608	B43y.00	Malignant neoplasm of other site of uterine body
33617	B43z.00	Malignant neoplasm of body of uterus NOS
19141	B44..00	Malignant neoplasm of ovary and other uterine adnexa
7805	B440.00	Malignant neoplasm of ovary
1986	B440.11	Cancer of ovary
49828	B441.00	Malignant neoplasm of fallopian tube
101778	B442.00	Malignant neoplasm of broad ligament
46153	B443.00	Malignant neoplasm of parametrium
97996	B44y.00	Malignant neoplasm of other site of uterine adnexa
65106	B44z.00	Malignant neoplasm of uterine adnexa NOS
4555	B45..00	Malig neop of other and unspecified female genital organs
37328	B450.00	Malignant neoplasm of vagina
10698	B450100	Malignant neoplasm of vaginal vault
60772	B450z00	Malignant neoplasm of vagina NOS
43761	B451.00	Malignant neoplasm of labia majora
47899	B451000	Malignant neoplasm of greater vestibular (Bartholin's) gland
59362	B451z00	Malignant neoplasm of labia majora NOS
58061	B452.00	Malignant neoplasm of labia minora
53910	B453.00	Malignant neoplasm of clitoris
4554	B454.00	Malignant neoplasm of vulva unspecified
11991	B454.11	Primary vulval cancer
26454	B45X.00	Malignant neoplasm/overlapping lesion/feml genital organs
95421	B45y.00	Malignant neoplasm of other specified female genital organ
27617	B45y000	Malignant neoplasm of overlapping lesion of vulva
20166	B45z.00	Malignant neoplasm of female genital organ NOS
780	B46..00	Malignant neoplasm of prostate
15148	B47..00	Malignant neoplasm of testis
64602	B470.00	Malignant neoplasm of undescended testis
7740	B470200	Seminoma of undescended testis
96429	B470z00	Malignant neoplasm of undescended testis NOS
19475	B471.00	Malignant neoplasm of descended testis
21786	B471000	Seminoma of descended testis
91509	B471z00	Malignant neoplasm of descended testis NOS
38510	B47z.00	Malignant neoplasm of testis NOS
2961	B47z.11	Seminoma of testis
3541	B48..00	Malignant neoplasm of penis and other male genital organs
50681	B480.00	Malignant neoplasm of prepuce (foreskin)
17841	B481.00	Malignant neoplasm of glans penis
48743	B482.00	Malignant neoplasm of body of penis
43392	B483.00	Malignant neoplasm of penis, part unspecified
72127	B484.00	Malignant neoplasm of epididymis
63331	B485.00	Malignant neoplasm of spermatic cord

47767	B486.00	Malignant neoplasm of scrotum
52570	B487.00	Malignant neoplasm, overlapping lesion of penis
67949	B48y.00	Malignant neoplasm of other male genital organ
68161	B48y000	Malignant neoplasm of seminal vesicle
47668	B48y100	Malignant neoplasm of tunica vaginalis
68824	B48y200	Malignant neoplasm, overlapping lesion male genital orgs
92329	B48yz00	Malignant neoplasm of other male genital organ NOS
63224	B48z.00	Malignant neoplasm of penis and other male genital organ NOS
779	B49..00	Malignant neoplasm of urinary bladder
38862	B490.00	Malignant neoplasm of trigone of urinary bladder
44996	B491.00	Malignant neoplasm of dome of urinary bladder
35963	B492.00	Malignant neoplasm of lateral wall of urinary bladder
19162	B493.00	Malignant neoplasm of anterior wall of urinary bladder
42012	B494.00	Malignant neoplasm of posterior wall of urinary bladder
41571	B495.00	Malignant neoplasm of bladder neck
28241	B496.00	Malignant neoplasm of ureteric orifice
42023	B497.00	Malignant neoplasm of urachus
105388	B498.00	Local recurrence of malignant tumour of urinary bladder
36949	B49y.00	Malignant neoplasm of other site of urinary bladder
47801	B49y000	Malignant neoplasm, overlapping lesion of bladder
31102	B49z.00	Malignant neoplasm of urinary bladder NOS
13559	B4A..00	Malig neop of kidney and other unspecified urinary organs
18712	B4A..11	Renal malignant neoplasm
1599	B4A0.00	Malignant neoplasm of kidney parenchyma
7978	B4A0000	Hypernephroma
12389	B4A1.00	Malignant neoplasm of renal pelvis
27540	B4A1000	Malignant neoplasm of renal calyces
101608	B4A1100	Malignant neoplasm of ureteropelvic junction
54184	B4A1z00	Malignant neoplasm of renal pelvis NOS
15223	B4A2.00	Malignant neoplasm of ureter
15644	B4A3.00	Malignant neoplasm of urethra
72174	B4A4.00	Malignant neoplasm of paraurethral glands
44884	B4Ay.00	Malignant neoplasm of other urinary organs
59286	B4Ay000	Malignant neoplasm of overlapping lesion of urinary organs
29462	B4Az.00	Malignant neoplasm of kidney or urinary organs NOS
38931	B4y..00	Malignant neoplasm of genitourinary organ OS
52594	B4z..00	Malignant neoplasm of genitourinary organ NOS
10995	B5...00	Malignant neoplasm of other and unspecified sites
8693	B5...11	Carcinoma of other and unspecified sites
20160	B50..00	Malignant neoplasm of eye
98813	B500.00	Malig neop eyeball excl conjunctiva, cornea, retina, choroid
59041	B500000	Malignant neoplasm of ciliary body
59381	B500100	Malignant neoplasm of iris
106569	B500200	Malignant neoplasm of crystalline lens
56718	B500z00	Malignant neoplasm of eyeball NOS
45667	B501.00	Malignant neoplasm of orbit
86996	B501000	Malignant neoplasm of connective tissue of orbit
63104	B501z00	Malignant neoplasm of orbit NOS
64817	B502.00	Malignant neoplasm of lacrimal gland
63657	B503.00	Malignant neoplasm of conjunctiva
73992	B504.00	Malignant neoplasm of cornea
28069	B505.00	Malignant neoplasm of retina
15991	B506.00	Malignant neoplasm of choroid
71584	B507.00	Malignant neoplasm of lacrimal duct
101805	B507000	Malignant neoplasm of lacrimal sac
65357	B507100	Malignant neoplasm of nasolacrimal duct
45922	B508.00	Malignant neoplasm, overlapping lesion of eye and adnexa
108363	B509.00	Malignant melanoma of eye
40437	B50y.00	Malignant neoplasm of other specified site of eye
54956	B50z.00	Malignant neoplasm of eye NOS
18617	B51..00	Malignant neoplasm of brain
10851	B51..11	Cerebral tumour - malignant
15711	B510.00	Malignant neoplasm cerebrum (excluding lobes and ventricles)
48073	B510000	Malignant neoplasm of basal ganglia
61399	B510100	Malignant neoplasm of cerebral cortex
99913	B510300	Malignant neoplasm of globus pallidus
70942	B510400	Malignant neoplasm of hypothalamus
62126	B510500	Malignant neoplasm of thalamus
54133	B510z00	Malignant neoplasm of cerebrum NOS
42426	B511.00	Malignant neoplasm of frontal lobe
46792	B512.00	Malignant neoplasm of temporal lobe

67236	B512000	Malignant neoplasm of hippocampus
47556	B512z00	Malignant neoplasm of temporal lobe NOS
19226	B513.00	Malignant neoplasm of parietal lobe
39088	B514.00	Malignant neoplasm of occipital lobe
52511	B515.00	Malignant neoplasm of cerebral ventricles
46789	B515000	Malignant neoplasm of choroid plexus
45154	B516.00	Malignant neoplasm of cerebellum
44089	B517.00	Malignant neoplasm of brain stem
64557	B517000	Malignant neoplasm of cerebral peduncle
49132	B517100	Malignant neoplasm of medulla oblongata
93537	B517200	Malignant neoplasm of midbrain
91240	B517300	Malignant neoplasm of pons
68641	B517z00	Malignant neoplasm of brain stem NOS
71139	B51y.00	Malignant neoplasm of other parts of brain
59170	B51y000	Malignant neoplasm of corpus callosum
65241	B51y200	Malignant neoplasm, overlapping lesion of brain
100733	B51yz00	Malignant neoplasm of other part of brain NOS
41520	B51z.00	Malignant neoplasm of brain NOS
65458	B52..00	Malig neop of other and unspecified parts of nervous system
99621	B520.00	Malignant neoplasm of cranial nerves
64971	B520000	Malignant neoplasm of olfactory bulb
70126	B520100	Malignant neoplasm of optic nerve
65599	B520200	Malignant neoplasm of acoustic nerve
101086	B520z00	Malignant neoplasm of cranial nerves NOS
28919	B521.00	Malignant neoplasm of cerebral meninges
110766	B521000	Malignant neoplasm of cerebral dura mater
109473	B521200	Malignant neoplasm of cerebral pia mater
70104	B521z00	Malignant neoplasm of cerebral meninges NOS
51115	B522.00	Malignant neoplasm of spinal cord
49714	B523.00	Malignant neoplasm of spinal meninges
67211	B523z00	Malignant neoplasm of spinal meninges NOS
24235	B524.00	Malig neopl peripheral nerves and autonomic nervous system
63568	B524000	Malignant neoplasm of peripheral nerves of head, face & neck
61716	B524100	Malignant neoplasm of peripheral nerve,upp limb,incl should
89258	B524200	Malignant neoplasm of peripheral nerve of low limb, incl hip
63695	B524300	Malignant neoplasm of peripheral nerve of thorax
86046	B524400	Malignant neoplasm of peripheral nerve of abdomen
73988	B524500	Malignant neoplasm of peripheral nerve of pelvis
50777	B524600	Malignant neoplasm,overlap lesion periph nerve & auton ns
106654	B524W00	Mal neoplasm/periph nerves+autonomic nervous system,unspc
9622	B525.00	Malignant neoplasm of cauda equina
53504	B52W.00	Malig neopl, overlap lesion brain & other part of CNS
49875	B52X.00	Malignant neoplasm of meninges, unspecified
88144	B52y.00	Malignant neoplasm of other specified part of nervous system
56490	B52z.00	Malignant neoplasm of nervous system NOS
5637	B53..00	Malignant neoplasm of thyroid gland
30511	B54..00	Malig neop of other endocrine glands and related structures
28148	B540.00	Malignant neoplasm of adrenal gland
61390	B540000	Malignant neoplasm of adrenal cortex
94220	B540100	Malignant neoplasm of adrenal medulla
70824	B540z00	Malignant neoplasm of adrenal gland NOS
4218	B541.00	Malignant neoplasm of parathyroid gland
59823	B542.00	Malignant neoplasm pituitary gland and craniopharyngeal duct
8550	B542000	Malignant neoplasm of pituitary gland
39899	B542100	Malignant neoplasm of craniopharyngeal duct
59718	B542z00	Malig neop pituitary gland or craniopharyngeal duct NOS
42460	B543.00	Malignant neoplasm of pineal gland
57047	B544.00	Malignant neoplasm of carotid body
50035	B545.00	Malignant neoplasm of aortic body and other paraganglia
51795	B545000	Malignant neoplasm of glomus jugulare
47840	B545100	Malignant neoplasm of aortic body
46905	B545200	Malignant neoplasm of coccygeal body
103995	B545z00	Malignant neoplasm of aortic body or paraganglia NOS
100083	B546.00	Neuroblastoma
87113	B54X.00	Malignant neoplasm-pluriglandular involvement,unspecified
90659	B54y.00	Malignant neoplasm of other specified endocrine gland
64195	B54z.00	Malig neop of endocrine gland or related structure NOS
9030	B55..00	Malignant neoplasm of other and ill-defined sites
68236	B550.00	Malignant neoplasm of head, neck and face
55098	B550000	Malignant neoplasm of head NOS
41931	B550100	Malignant neoplasm of cheek NOS

12490	B550200	Malignant neoplasm of nose NOS
51818	B550300	Malignant neoplasm of jaw NOS
16280	B550400	Malignant neoplasm of neck NOS
73510	B550500	Malignant neoplasm of supraclavicular fossa NOS
58903	B550z00	Malignant neoplasm of head, neck and face NOS
47286	B551.00	Malignant neoplasm of thorax
37618	B551000	Malignant neoplasm of axilla NOS
23861	B551100	Malignant neoplasm of chest wall NOS
97547	B551200	Malignant neoplasm of intrathoracic site NOS
64810	B551z00	Malignant neoplasm of thorax NOS
15976	B552.00	Malignant neoplasm of abdomen
52316	B553.00	Malignant neoplasm of pelvis
57854	B553000	Malignant neoplasm of inguinal region NOS
89916	B553100	Malignant neoplasm of presacral region
107126	B553200	Malignant neoplasm of sacrococcygeal region
55101	B553z00	Malignant neoplasm of pelvis NOS
27449	B554.00	Malignant neoplasm of upper limb NOS
31399	B555.00	Malignant neoplasm of lower limb NOS
42218	B55y.00	Malignant neoplasm of other specified sites
68787	B55y000	Malignant neoplasm of back NOS
67217	B55y100	Malignant neoplasm of trunk NOS
94355	B55y200	Malignant neoplasm of flank NOS
60052	B55yz00	Malignant neoplasm of specified site NOS
45267	B55z.00	Malignant neoplasm of other and ill defined site NOS
9618	B56..00	Secondary and unspecified malignant neoplasm of lymph nodes
7830	B56..11	Lymph node metastases
49214	B560.00	Secondary and unspec malig neop lymph nodes head/face/neck
64918	B560000	Secondary and unspec malig neop of superficial parotid LN
66775	B560100	Secondary and unspec malignant neoplasm mastoid lymph nodes
33395	B560200	Secondary and unspec malig neop superficial cervical LN
65253	B560300	Secondary and unspec malignant neoplasm occipital lymph node
92703	B560400	Secondary and unspec malig neop deep parotid lymph nodes
39433	B560500	Secondary and unspec malig neop submandibular lymph nodes
28059	B560600	Secondary and unspec malig neop of facial lymph nodes
38343	B560700	Secondary and unspec malig neop submental lymph nodes
44627	B560800	Secondary and unspec malig neop anterior cervical LN
68611	B560900	Secondary and unspec malig neop deep cervical LN
67129	B560z00	Secondary unspec malig neop lymph nodes head/face/neck NOS
64116	B561.00	Secondary and unspec malig neop intrathoracic lymph nodes
37919	B561000	Secondary and unspec malig neop internal mammary lymph nodes
105953	B561100	Secondary and unspec malig neop intercostal lymph nodes
95378	B561200	Secondary and unspec malig neop diaphragmatic lymph nodes
25366	B561300	Secondary and unspec malig neop ant mediastinal lymph nodes
55463	B561400	Secondary and unspec malig neop post mediastinal lymph nodes
58692	B561500	Secondary and unspec malig neop paratracheal lymph nodes
67797	B561600	Secondary and unspec malig neop superfic tracheobronchial LN
69392	B561700	Secondary and unspec malig neop inferior tracheobronchial LN
62124	B561800	Secondary and unspec malig neop bronchopulmonary lymph nodes
52190	B561900	Secondary and unspec malig neop pulmonary lymph nodes
93716	B561z00	Secondary and unspec malig neop intrathoracic LN NOS
52736	B562.00	Secondary and unspec malig neop intra-abdominal lymph nodes
41691	B562000	Secondary and unspec malig neop coeliac lymph nodes
72713	B562100	Secondary and unspec malig neop superficial mesenteric LN
61677	B562200	Secondary and unspec malig neop inferior mesenteric LN
18658	B562300	Secondary and unspec malig neop common iliac lymph nodes
69132	B562400	Secondary and unspec malig neop external iliac lymph nodes
44931	B562z00	Secondary and unspec malig neop intra-abdominal LN NOS
50199	B563.00	Secondary and unspec malig neop axilla and upper limb LN
37540	B563000	Secondary and unspec malig neop axillary lymph nodes
98626	B563100	Secondary and unspec malig neop supratrochlear lymph nodes
50904	B563200	Secondary and unspec malig neop infraclavicular lymph nodes
46409	B563300	Secondary and unspec malig neop pectoral lymph nodes
73538	B563z00	Secondary and unspec malig neop axilla and upper limb LN NOS
63915	B564.00	Secondary and unspec malig neop inguinal and lower limb LN
54278	B564000	Secondary and unspec malig neop superficial inguinal LN
61289	B564100	Secondary and unspec malig neop deep inguinal lymph nodes
70747	B564z00	Secondary and unspec malig neop of inguinal and leg LN NOS
6701	B565.00	Secondary and unspec malig neop intrapelvic lymph nodes
84368	B565000	Secondary and unspec malig neop internal iliac lymph nodes
101662	B565200	Secondary and unspec malig neop circumflex iliac LN
47366	B565300	Secondary and unspec malig neop sacral lymph nodes

72803	B565z00	Secondary and unspec malig neop intrapelvic LN NOS
20159	B56y.00	Secondary and unspec malig neop lymph nodes multiple sites
15507	B56z.00	Secondary and unspec malig neop lymph nodes NOS
35053	B57..00	Secondary malig neop of respiratory and digestive systems
6471	B57..11	Metastases of respiratory and/or digestive systems
24301	B57..12	Secondary carcinoma of respiratory and/or digestive systems
4137	B570.00	Secondary malignant neoplasm of lung
51551	B571.00	Secondary malignant neoplasm of mediastinum
16213	B572.00	Secondary malignant neoplasm of pleura
62584	B573.00	Secondary malignant neoplasm of other respiratory organs
64680	B574.00	Secondary malignant neoplasm of small intestine and duodenum
55946	B574000	Secondary malignant neoplasm of duodenum
110433	B574100	Secondary malignant neoplasm of jejunum
99511	B574200	Secondary malignant neoplasm of ileum
70026	B574z00	Secondary malig neop of small intestine or duodenum NOS
44529	B575.00	Secondary malignant neoplasm of large intestine and rectum
28727	B575000	Secondary malignant neoplasm of colon
62909	B575100	Secondary malignant neoplasm of rectum
36200	B575z00	Secondary malig neop of large intestine or rectum NOS
67396	B576.00	Secondary malig neop of retroperitoneum and peritoneum
35364	B576000	Secondary malignant neoplasm of retroperitoneum
27391	B576100	Secondary malignant neoplasm of peritoneum
8154	B576200	Malignant ascites
97672	B576z00	Secondary malig neop of retroperitoneum or peritoneum NOS
15103	B577.00	Secondary malignant neoplasm of liver
4403	B577.11	Liver metastases
56345	B57y.00	Secondary malignant neoplasm of other digestive organ
66083	B57z.00	Secondary malig neop of respiratory or digestive system NOS
5842	B58..00	Secondary malignant neoplasm of other specified sites
27651	B58..11	Secondary carcinoma of other specified sites
1952	B580.00	Secondary malignant neoplasm of kidney
73213	B581.00	Secondary malignant neoplasm of other urinary organs
60134	B581000	Secondary malignant neoplasm of ureter
22146	B581100	Secondary malignant neoplasm of bladder
53528	B581200	Secondary malignant neoplasm of urethra
62828	B581z00	Secondary malignant neoplasm of other urinary organ NOS
19945	B582.00	Secondary malignant neoplasm of skin
43930	B582000	Secondary malignant neoplasm of skin of head
100296	B582100	Secondary malignant neoplasm of skin of face
35999	B582200	Secondary malignant neoplasm of skin of neck
41144	B582300	Secondary malignant neoplasm of skin of trunk
63896	B582400	Secondary malignant neoplasm of skin of shoulder and arm
48828	B582500	Secondary malignant neoplasm of skin of hip and leg
9505	B582600	Secondary malignant neoplasm of skin of breast
55096	B582z00	Secondary malignant neoplasm of skin NOS
33843	B583.00	Secondary malignant neoplasm of brain and spinal cord
5198	B583000	Secondary malignant neoplasm of brain
38918	B583100	Secondary malignant neoplasm of spinal cord
5199	B583200	Cerebral metastasis
59375	B583z00	Secondary malignant neoplasm of brain or spinal cord NOS
54120	B584.00	Secondary malignant neoplasm of other part of nervous system
7654	B585.00	Secondary malignant neoplasm of bone and bone marrow
18676	B585000	Pathological fracture due to metastatic bone disease
44615	B586.00	Secondary malignant neoplasm of ovary
36401	B587.00	Secondary malignant neoplasm of adrenal gland
18616	B58y.00	Secondary malignant neoplasm of other specified sites
16760	B58y000	Secondary malignant neoplasm of breast
55090	B58y100	Secondary malignant neoplasm of uterus
73616	B58y200	Secondary malignant neoplasm of cervix uteri
97832	B58y211	Secondary cancer of the cervix
70736	B58y300	Secondary malignant neoplasm of vagina
60335	B58y400	Secondary malignant neoplasm of vulva
65490	B58y411	Secondary cancer of the vulva
21590	B58y500	Secondary malignant neoplasm of prostate
34145	B58y600	Secondary malignant neoplasm of testis
49145	B58y700	Secondary malignant neoplasm of penis
104480	B58y800	Secondary malignant neoplasm of epididymis and vas deferens
45824	B58y900	Secondary malignant neoplasm of tongue
22524	B58yz00	Secondary malignant neoplasm of other specified site NOS
16500	B58z.00	Secondary malignant neoplasm of other specified site NOS
47810	B59..00	Malignant neoplasm of unspecified site

13569	B590.00	Disseminated malignancy NOS
6170	B590.11	Carcinomatosis
26034	B591.00	Other malignant neoplasm NOS
51352	B592.00	Malignant neoplasms of independent (primary) multiple sites
65466	B592X00	Kaposi's sarcoma of multiple organs
11035	B593.00	Primary malignant neoplasm of unknown site
54679	B594.00	Secondary malignant neoplasm of unknown site
104324	B595.00	Malignant tumour of unknown origin
54267	B59z.00	Malignant neoplasm of unspecified site NOS
49525	B59zX00	Kaposi's sarcoma, unspecified
38736	B5y..00	Malignant neoplasm of other and unspecified site OS
1056	B5z..00	Malignant neoplasm of other and unspecified site NOS
12323	B6...00	Malignant neoplasm of lymphatic and haemopoietic tissue
37112	B6...11	Malignant neoplasm of histiocytic tissue
41369	B60..00	Lymphosarcoma and reticulosarcoma
1481	B600.00	Reticulosarcoma
60242	B600000	Reticulosarcoma of unspecified site
71031	B600100	Reticulosarcoma of lymph nodes of head, face and neck
70374	B600300	Reticulosarcoma of intra-abdominal lymph nodes
95058	B600700	Reticulosarcoma of spleen
99240	B600z00	Reticulosarcoma NOS
27416	B601.00	Lymphosarcoma
71625	B601000	Lymphosarcoma of unspecified site
71238	B601100	Lymphosarcoma of lymph nodes of head, face and neck
62380	B601200	Lymphosarcoma of intrathoracic lymph nodes
64670	B601300	Lymphosarcoma of intra-abdominal lymph nodes
100352	B601500	Lymphosarcoma of lymph nodes of inguinal region and leg
103245	B601700	Lymphosarcoma of spleen
104790	B601800	Lymphosarcoma of lymph nodes of multiple sites
63723	B601z00	Lymphosarcoma NOS
21402	B602.00	Burkitt's lymphoma
59115	B602100	Burkitt's lymphoma of lymph nodes of head, face and neck
100006	B602200	Burkitt's lymphoma of intrathoracic lymph nodes
97577	B602300	Burkitt's lymphoma of intra-abdominal lymph nodes
92380	B602500	Burkitt's lymphoma of lymph nodes of inguinal region and leg
71304	B602z00	Burkitt's lymphoma NOS
99887	B60y.00	Other specified reticulosarcoma or lymphosarcoma
99951	B60z.00	Reticulosarcoma or lymphosarcoma NOS
2462	B61..00	Hodgkin's disease
104291	B61..11	Hodgkin lymphoma
65489	B610.00	Hodgkin's paragranuloma
100423	B610100	Hodgkin's paragranuloma of lymph nodes of head, face, neck
98840	B610300	Hodgkin's paragranuloma of intra-abdominal lymph nodes
44196	B611.00	Hodgkin's granuloma
98909	B611100	Hodgkin's granuloma of lymph nodes of head, face and neck
64036	B612.00	Hodgkin's sarcoma
68039	B612400	Hodgkin's sarcoma of lymph nodes of axilla and upper limb
38939	B613.00	Hodgkin's disease, lymphocytic-histiocytic predominance
71142	B613000	Hodgkin's, lymphocytic-histiocytic predominance unspec site
68330	B613100	Hodgkin's, lymphocytic-histiocytic pred of head, face, neck
92245	B613200	Hodgkin's, lymphocytic-histiocytic pred intrathoracic nodes
73532	B613300	Hodgkin's, lymphocytic-histiocytic pred intra-abdominal node
93951	B613500	Hodgkin's, lymphocytic-histiocytic pred inguinal and leg
95338	B613600	Hodgkin's, lymphocytic-histiocytic pred intrapelvic nodes
106911	B613700	Hodgkin's, lymphocytic-histiocytic predominance of spleen
104743	B613800	Hodgkin's, lymphocytic-histiocytic pred of multiple sites
29876	B613z00	Hodgkin's, lymphocytic-histiocytic predominance NOS
29178	B614.00	Hodgkin's disease, nodular sclerosis
57225	B614000	Hodgkin's disease, nodular sclerosis of unspecified site
55303	B614100	Hodgkin's nodular sclerosis of head, face and neck
67506	B614200	Hodgkin's nodular sclerosis of intrathoracic lymph nodes
61149	B614300	Hodgkin's nodular sclerosis of intra-abdominal lymph nodes
65483	B614400	Hodgkin's nodular sclerosis of lymph nodes of axilla and arm
105472	B614700	Hodgkin's disease, nodular sclerosis of spleen
19140	B614800	Hodgkin's nodular sclerosis of lymph nodes of multiple sites
63054	B614z00	Hodgkin's disease, nodular sclerosis NOS
49605	B615.00	Hodgkin's disease, mixed cellularity
97863	B615000	Hodgkin's disease, mixed cellularity of unspecified site
94407	B615100	Hodgkin's mixed cellularity of lymph nodes head, face, neck
58684	B615200	Hodgkin's mixed cellularity of intrathoracic lymph nodes
108886	B615500	Hodgkin's mixed cellularity of lymph nodes inguinal and leg

94005	B615z00	Hodgkin's disease, mixed cellularity NOS
67703	B616.00	Hodgkin's disease, lymphocytic depletion
95049	B616000	Hodgkin's lymphocytic depletion of unspecified site
111942	B616100	Hodgkin's lymphocytic depletion of head, face and neck
63625	B616400	Hodgkin's lymphocytic depletion lymph nodes axilla and arm
110563	B616500	Hodgkin's lymphocytic depletion lymph nodes inguinal and leg
101715	B616700	Hodgkin's disease, lymphocytic depletion of spleen
107032	B616800	Hodgkin's lymphocytic depletion lymph nodes multiple sites
101530	B616z00	Hodgkin's disease, lymphocytic depletion NOS
104895	B617.00	Nodular lymphocyte predominant Hodgkin lymphoma
105841	B618.00	Nodular sclerosis classical Hodgkin lymphoma
108775	B619.00	Mixed cellularity classical Hodgkin lymphoma
106597	B61B.00	Lymphocyte-rich classical Hodgkin lymphoma
104484	B61C.00	Other classical Hodgkin lymphoma
53397	B61z.00	Hodgkin's disease NOS
106349	B61z.11	Hodgkin lymphoma NOS
61662	B61z000	Hodgkin's disease NOS, unspecified site
59778	B61z100	Hodgkin's disease NOS of lymph nodes of head, face and neck
59755	B61z200	Hodgkin's disease NOS of intrathoracic lymph nodes
107804	B61z300	Hodgkin's disease NOS of intra-abdominal lymph nodes
91900	B61z400	Hodgkin's disease NOS of lymph nodes of axilla and arm
99012	B61z500	Hodgkin's disease NOS of lymph nodes inguinal region and leg
94279	B61z700	Hodgkin's disease NOS of spleen
97746	B61z800	Hodgkin's disease NOS of lymph nodes of multiple sites
42461	B61zz00	Hodgkin's disease NOS
33333	B62..00	Other malignant neoplasm of lymphoid and histiocytic tissue
5179	B620.00	Nodular lymphoma (Brill - Symmers disease)
66327	B620000	Nodular lymphoma of unspecified site
45264	B620100	Nodular lymphoma of lymph nodes of head, face and neck
105203	B620200	Nodular lymphoma of intrathoracic lymph nodes
92068	B620300	Nodular lymphoma of intra-abdominal lymph nodes
111766	B620400	Nodular lymphoma of lymph nodes of axilla and upper limb
94995	B620500	Nodular lymphoma of lymph nodes of inguinal region and leg
58082	B620800	Nodular lymphoma of lymph nodes of multiple sites
65701	B620z00	Nodular lymphoma NOS
12006	B621.00	Mycosis fungoides
95949	B621000	Mycosis fungoides of unspecified site
91674	B621300	Mycosis fungoides of intra-abdominal lymph nodes
96379	B621400	Mycosis fungoides of lymph nodes of axilla and upper limb
72714	B621500	Mycosis fungoides of lymph nodes of inguinal region and leg
95012	B621800	Mycosis fungoides of lymph nodes of multiple sites
38005	B621z00	Mycosis fungoides NOS
35014	B622.00	Sezary's disease
100532	B622z00	Sezary's disease NOS
44267	B623.00	Malignant histiocytosis
69497	B623000	Malignant histiocytosis of unspecified site
94415	B623100	Malignant histiocytosis of lymph nodes head, face and neck
65642	B623300	Malignant histiocytosis of intra-abdominal lymph nodes
110903	B623800	Malignant histiocytosis of lymph nodes of multiple sites
58871	B623z00	Malignant histiocytosis NOS
27330	B624.00	Leukaemic reticuloendotheliosis
5137	B624.11	Leukaemic reticuloendotheliosis
87335	B624.12	Hairy cell leukaemia
65122	B624000	Leukaemic reticuloendotheliosis of unspecified sites
65123	B624300	Leukaemic reticuloend of intra-abdominal lymph nodes
73777	B624z00	Leukaemic reticuloendotheliosis NOS
34926	B625.00	Letterer-Siwe disease
4870	B625.11	Histiocytosis X (acute, progressive)
102715	B625000	Letterer-Siwe disease of unspecified sites
102158	B625200	Letterer-Siwe disease of intrathoracic lymph nodes
54083	B625800	Letterer-Siwe disease of lymph nodes of multiple sites
47204	B625z00	Letterer-Siwe disease NOS
15036	B626.00	Malignant mast cell tumours
103900	B626000	Mast cell malignancy of unspecified site
100615	B626500	Mast cell malignancy of lymph nodes inguinal region and leg
31324	B626800	Mast cell malignancy of lymph nodes of multiple sites
89657	B626z00	Malignant mast cell tumour NOS
3604	B627.00	Non - Hodgkin's lymphoma
104391	B627.11	Non-Hodgkin lymphoma
28639	B627000	Follicular non-Hodgkin's small cleaved cell lymphoma
70842	B627100	Follicular non-Hodg mixed sml cleavd & lge cell lymphoma

49262	B627200	Follicular non-Hodgkin's large cell lymphoma
50668	B627300	Diffuse non-Hodgkin's small cell (diffuse) lymphoma
108182	B627400	Diffuse non-Hodgkin's small cleaved cell (diffuse) lymphoma
50695	B627500	Diffuse non-Hodgkin mixed sml & lge cell (diffuse) lymphoma
53551	B627600	Diffuse non-Hodgkin's immunoblastic (diffuse) lymphoma
17460	B627700	Diffuse non-Hodgkin's lymphoblastic (diffuse) lymphoma
65180	B627800	Diffuse non-Hodgkin's lymphoma undifferentiated (diffuse)
95715	B627900	Mucosa-associated lymphoma
95545	B627911	Maltoma
101114	B627A00	Diffuse non-Hodgkin's large cell lymphoma
31576	B627B00	Other types of follicular non-Hodgkin's lymphoma
21549	B627C00	Follicular non-Hodgkin's lymphoma
17182	B627C11	Follicular lymphoma NOS
70509	B627D00	Diffuse non-Hodgkin's centroblastic lymphoma
102594	B627E00	Diffuse large B-cell lymphoma
105966	B627F00	Extranod marg zone B-cell lymphom mucosa-assoc lymphoid tiss
105038	B627G00	Mediastinal (thymic) large B-cell lymphoma
31794	B627W00	Unspecified B-cell non-Hodgkin's lymphoma
39798	B627X00	Diffuse non-Hodgkin's lymphoma, unspecified
104152	B628.00	Follicular lymphoma
105889	B628000	Follicular lymphoma grade 1
105095	B628100	Follicular lymphoma grade 2
107166	B628200	Follicular lymphoma grade 3
105020	B628300	Follicular lymphoma grade 3a
107973	B628400	Follicular lymphoma grade 3b
106969	B628500	Diffuse follicle centre lymphoma
108719	B628600	Cutaneous follicle centre lymphoma
106063	B628700	Other types of follicular lymphoma
105792	B629.00	Multifocal multisystemic dissem Langerhans-cell histiocytosi
105335	B62A.00	Sarcoma of dendritic cells
110191	B62B.00	Multifocal and unisystemic Langerhans-cell histiocytosis
105762	B62C.00	Unifocal Langerhans-cell histiocytosis
105083	B62D.00	Histiocytic sarcoma
105085	B62E.00	T/NK-cell lymphoma
105559	B62E100	Anaplastic large cell lymphoma, ALK-positive
105955	B62E200	Anaplastic large cell lymphoma, ALK-negative
104862	B62E300	Cutaneous T-cell lymphoma
109780	B62E400	Extranodal NK/T-cell lymphoma, nasal type
107949	B62E500	Hepatosplenic T-cell lymphoma
105709	B62E600	Enteropathy-associated T-cell lymphoma
105925	B62E700	Subcutaneous panniculitic T-cell lymphoma
105375	B62E800	Blastic NK-cell lymphoma
105636	B62E900	Angioimmunoblastic T-cell lymphoma
104934	B62Ew00	Other mature T/NK-cell lymphoma
106884	B62F.00	Nonfollicular lymphoma
106867	B62F.11	Non-follicular lymphoma
104386	B62F000	Small cell B-cell lymphoma
104620	B62F100	Mantle cell lymphoma
104412	B62F200	Lymphoblastic (diffuse) lymphoma
111682	B62Fy00	Other non-follicular lymphoma
17887	B62x.00	Malignant lymphoma otherwise specified
90201	B62x000	T-zone lymphoma
57737	B62x100	Lymphoepithelioid lymphoma
12464	B62x200	Peripheral T-cell lymphoma
62437	B62x400	Malignant reticulosis
58962	B62x500	Malignant immunoproliferative small intestinal disease
95630	B62x600	True histiocytic lymphoma
44318	B62xX00	Oth and unspecif peripheral & cutaneous T-cell lymphomas
12335	B62y.00	Malignant lymphoma NOS
57427	B62y000	Malignant lymphoma NOS of unspecified site
50696	B62y100	Malignant lymphoma NOS of lymph nodes of head, face and neck
72725	B62y200	Malignant lymphoma NOS of intrathoracic lymph nodes
42579	B62y300	Malignant lymphoma NOS of intra-abdominal lymph nodes
34089	B62y400	Malignant lymphoma NOS of lymph nodes of axilla and arm
63105	B62y500	Malignant lymphoma NOS of lymph node inguinal region and leg
71262	B62y600	Malignant lymphoma NOS of intrapelvic lymph nodes
60092	B62y700	Malignant lymphoma NOS of spleen
15504	B62y800	Malignant lymphoma NOS of lymph nodes of multiple sites
15027	B62yz00	Malignant lymphoma NOS
65434	B62z.00	Malignant neoplasms of lymphoid and histiocytic tissue NOS
108037	B62z000	Unspec malig neop lymphoid/histiocytic of unspecified site

64427	B62z100	Unspec malig neop lymphoid/histiocytic lymph node head/neck
93384	B62z200	Unspec malig neop lymphoid/histiocytic of intrathoracic node
103353	B62z300	Unspec malig neop lymphoid/histiocytic intra-abdominal nodes
107638	B62z400	Unspec malig neop lymphoid/histiocytic lymph node axilla/arm
71609	B62z500	Unspec malig neop lymphoid/histiocytic nodes inguinal/leg
109342	B62z600	Unspec malig neop lymphoid/histiocytic of intrapelvic nodes
101465	B62z800	Unspec malig neop lymphoid/histiocytic of multiple sites
95792	B62zz00	Lymphoid and histiocytic malignancy NOS
4944	B630.00	Multiple myeloma
43552	B630.11	Kahler's disease
15211	B630.12	Myelomatosis
22158	B630000	Malignant plasma cell neoplasm, extramedullary plasmacytoma
19028	B630100	Solitary myeloma
21329	B630200	Plasmacytoma NOS
46042	B630300	Lambda light chain myeloma
104418	B630400	Solitary plasmacytoma
39187	B631.00	Plasma cell leukaemia
19372	B64..00	Lymphoid leukaemia
4222	B64..11	Lymphatic leukaemia
4251	B640.00	Acute lymphoid leukaemia
104325	B640000	B-cell acute lymphoblastic leukaemia
8625	B641.00	Chronic lymphoid leukaemia
27790	B641.11	Chronic lymphatic leukaemia
104328	B641000	B-cell chronic lymphocytic leukaemia
107017	B641011	Chronic lymphocytic leukaemia of B-cell type
107052	B641100	Clinical stage A chronic lymphocytic leukaemia
106924	B641200	Clinical stage B chronic lymphocytic leukaemia
107163	B641300	Clinical stage C chronic lymphocytic leukaemia
72774	B642.00	Subacute lymphoid leukaemia
49725	B64y.00	Other lymphoid leukaemia
31586	B64y100	Prolymphocytic leukaemia
37461	B64y200	Adult T-cell leukaemia
108656	B64y300	B-cell prolymphocytic leukaemia
107643	B64y400	T-cell prolymphocytic leukaemia
111627	B64y411	Prolymphocytic leukaemia of T-cell type
104939	B64y500	Adult T-cell lymphoma/leukaemia (HTLV-1-associated)
38331	B64yz00	Other lymphoid leukaemia NOS
38914	B64z.00	Lymphoid leukaemia NOS
7176	B65..00	Myeloid leukaemia
4413	B650.00	Acute myeloid leukaemia
10726	B651.00	Chronic myeloid leukaemia
31701	B651.11	Chronic granulocytic leukaemia
100786	B651000	Chronic eosinophilic leukaemia
105957	B651100	Chronic myeloid leukaemia, BCR/ABL positive
102783	B651200	Chronic neutrophilic leukaemia
107236	B651300	Atypical chronic myeloid leukaemia, BCR/ABL negative
27520	B651z00	Chronic myeloid leukaemia NOS
63475	B652.00	Subacute myeloid leukaemia
70724	B653.00	Myeloid sarcoma
52327	B653000	Chloroma
39629	B653100	Granulocytic sarcoma
104788	B654.00	Acute myeloblastic leukaemia
27664	B65y100	Acute promyelocytic leukaemia
66089	B65yz00	Other myeloid leukaemia NOS
33344	B65z.00	Myeloid leukaemia NOS
35875	B66..00	Monocytic leukaemia
108715	B66..11	Histiocytic leukaemia
67700	B66..12	Monoblastic leukaemia
19974	B660.00	Acute monocytic leukaemia
27458	B661.00	Chronic monocytic leukaemia
101606	B662.00	Subacute monocytic leukaemia
108424	B663.00	Acute monoblastic leukaemia
99015	B66y.00	Other monocytic leukaemia
103645	B66yz00	Other monocytic leukaemia NOS
93342	B66z.00	Monocytic leukaemia NOS
37272	B67..00	Other specified leukaemia
42539	B670.00	Acute erythraemia and erythroleukaemia
27340	B670.11	Di Guglielmo's disease
37468	B671.00	Chronic erythraemia
63653	B671.11	Heilmeyer - Schoner disease
57671	B672.00	Megakaryocytic leukaemia

65777	B672.11	Thrombocytic leukaemia
65721	B673.00	Mast cell leukaemia
50858	B674.00	Acute panmyelosis
28276	B675.00	Acute myelofibrosis
110838	B676.00	Acute erythroid leukaemia
104273	B677.00	Myelodysplastic and myeloproliferative disease
94174	B67y.00	Other and unspecified leukaemia
72197	B67y000	Lymphosarcoma cell leukaemia
99413	B67yz00	Other and unspecified leukaemia NOS
30632	B67z.00	Other specified leukaemia NOS
25191	B68..00	Leukaemia of unspecified cell type
4072	B680.00	Acute leukaemia NOS
16416	B681.00	Chronic leukaemia NOS
54793	B682.00	Subacute leukaemia NOS
34692	B68y.00	Other leukaemia of unspecified cell type
4250	B68z.00	Leukaemia NOS
20440	B69..00	Myelomonocytic leukaemia
61500	B690.00	Acute myelomonocytic leukaemia
22050	B691.00	Chronic myelomonocytic leukaemia
104475	B692.00	Subacute myelomonocytic leukaemia
105069	B693.00	Juvenile myelomonocytic leukaemia
30646	B6y..00	Malignant neoplasm lymphatic or haematopoietic tissue OS
49301	B6z..00	Malignant neoplasm lymphatic or haematopoietic tissue NOS
50290	B6z0.00	Kaposi's sarcoma of lymph nodes
104684	B76D.00	Multiple self-healing epithelioma of Ferguson-Smith
7473	B8...00	Carcinoma in situ
60511	B80..00	Carcinoma in situ of digestive organs
33250	B80..11	Ca-in-situ of G.I. tract
95390	B800.00	Carcinoma in situ of lip, oral cavity and pharynx
37505	B800.11	Carcinoma in situ of oral cavity
42129	B800.12	Carcinoma in situ of pharynx
47737	B800000	Carcinoma in situ of lip
27944	B800100	Carcinoma in situ of tongue
50288	B800200	Carcinoma in situ of salivary glands
57866	B800300	Carcinoma in situ of gums
24801	B800400	Carcinoma in situ of floor of mouth
34823	B800500	Carcinoma in situ of cheek
30966	B800600	Carcinoma in situ of palate
36104	B800700	Carcinoma in situ of nasopharynx
50419	B800800	Carcinoma in situ of oropharynx
44663	B800900	Carcinoma in situ of hypopharynx
37187	B800z00	Carcinoma in situ of lip, oral cavity and pharynx NOS
8244	B801.00	Carcinoma in situ of oesophagus
99155	B801000	Carcinoma in situ of upper 1/3 oesophagus
64274	B801100	Carcinoma in situ of middle 1/3 oesophagus
56077	B801200	Carcinoma in situ of lower 1/3 oesophagus
44228	B801z00	Carcinoma in situ of oesophagus NOS
17093	B802.00	Carcinoma in situ of stomach
17258	B802000	Carcinoma in situ of cardia of stomach
72947	B802100	Carcinoma in situ of fundus of stomach
63087	B802200	Carcinoma in situ of body of stomach
51748	B802300	Carcinoma in situ of pyloric antrum
58883	B802400	Carcinoma in situ of pyloric canal
37774	B802z00	Carcinoma in situ of stomach NOS
6903	B803.00	Carcinoma in situ of colon
39080	B803000	Carcinoma in situ of hepatic flexure of colon
37125	B803100	Carcinoma in situ of transverse colon
47667	B803200	Carcinoma in situ of descending colon
17144	B803300	Carcinoma in situ of sigmoid colon
16916	B803400	Carcinoma in situ of caecum
47656	B803500	Carcinoma in situ of appendix
31893	B803600	Carcinoma in situ of ascending colon
22699	B803700	Carcinoma in situ of splenic flexure of colon
33561	B803z00	Carcinoma in situ of colon NOS
60477	B804.00	Carcinoma in situ of rectum and rectosigmoid junction
27811	B804000	Carcinoma in situ of rectosigmoid junction
29975	B804100	Carcinoma in situ of rectum
38883	B804z00	Carcinoma in situ of rectum or rectosigmoid junction NOS
51054	B805.00	Carcinoma in situ of anal canal
12273	B806.00	Carcinoma in situ of anus NOS
22392	B807.00	Carcinoma in situ of other and unspecified small intestine

45070	B807000	Carcinoma in situ of duodenum
63804	B807100	Carcinoma in situ of jejunum
45217	B807200	Carcinoma in situ of ileum
100183	B807300	Carcinoma in situ of Meckel's diverticulum
70728	B807z00	Carcinoma in situ other and unspecified small intestine NOS
66673	B808.00	Carcinoma in situ of liver and biliary system
51934	B808.11	Carcinoma in situ of biliary system
25310	B808000	Carcinoma in situ of liver
99580	B808100	Carcinoma in situ of intrahepatic bile ducts
37501	B808200	Carcinoma in situ of hepatic duct
46594	B808300	Carcinoma in situ of gall bladder
73164	B808400	Carcinoma in situ of cystic duct
64089	B808500	Carcinoma in situ of common bile duct
21792	B808600	Carcinoma in situ of ampulla of Vater
98540	B808z00	Carcinoma in situ of liver or biliary system NOS
44166	B80z.00	Carcinoma in situ of other and unspecified digestive organs
16931	B80z000	Carcinoma in situ of pancreas
64700	B80z100	Carcinoma in situ of spleen
64050	B81..00	Carcinoma in situ of respiratory system
11403	B810.00	Carcinoma in situ of larynx
35772	B810000	Carcinoma in situ of thyroid cartilage
36948	B810100	Carcinoma in situ of cricoid cartilage
53460	B810200	Carcinoma in situ of epiglottis
65953	B810300	Carcinoma in situ of arytenoid cartilage
31860	B810600	Carcinoma in situ of aryepiglottic fold
73076	B810700	Carcinoma in situ of vestibular fold
7697	B810800	Carcinoma in situ of vocal fold - glottis
10375	B810811	Carcinoma in situ of glottis
53882	B810z00	Carcinoma in situ of larynx NOS
51714	B811.00	Carcinoma in situ of trachea
9267	B812.00	Carcinoma in situ of bronchus and lung
49159	B812000	Carcinoma in situ of carina of bronchus
35058	B812100	Carcinoma in situ of main bronchus
37579	B812200	Carcinoma in situ of upper lobe bronchus and lung
47897	B812300	Carcinoma in situ of middle lobe bronchus and lung
52373	B812400	Carcinoma in situ of lower lobe bronchus and lung
25372	B812z00	Carcinoma in situ of bronchus or lung NOS
97954	B81y.00	Carcinoma in situ of other specified part respiratory system
59426	B81y.11	Carcinoma in situ of nasal sinuses
46497	B81y000	Carcinoma in situ of pleura
39717	B81y100	Carcinoma in situ of nasal cavity
97200	B81y400	Carcinoma in situ of Eustachian tube
68135	B81y500	Carcinoma in situ of mastoid air cells
43380	B81y600	Carcinoma in situ of maxillary sinus
26846	B81y700	Carcinoma in situ of ethmoidal sinus
39064	B81y900	Carcinoma in situ of sphenoidal sinus
95559	B81yz00	Carcinoma in situ of specified parts respiratory system NOS
62610	B81z.00	Carcinoma in situ of respiratory organ NOS
12084	B82..00	Carcinoma in situ of skin
63957	B820.00	Carcinoma in situ of skin of lip
57550	B821.00	Carcinoma in situ of skin of eyelid including canthus
50189	B822.00	Carcinoma in situ skin of ear and external auricular canal
32249	B822.11	Carcinoma in situ of ear
59614	B822000	Carcinoma in situ of skin of auricle
70295	B822z00	Carcinoma in situ skin of ear/external auricular canal NOS
49254	B823.00	Carcinoma in situ of skin of other parts of face
47789	B823000	Carcinoma in situ of skin of forehead skin
69720	B823100	Carcinoma in situ of skin of eyebrow
61103	B823300	Carcinoma in situ of skin of cheek
3135	B823400	Carcinoma in situ of skin of nose
31511	B823500	Carcinoma in situ of skin of temple
65222	B823600	Carcinoma in situ of skin of jaw
110614	B823z00	Carcinoma in situ of skin of other parts of face NOS
69345	B824.00	Carcinoma in situ of scalp and skin of neck
19665	B824000	Carcinoma in situ of scalp
54140	B824100	Carcinoma in situ of skin of neck
52328	B825.00	Carcinoma in situ of skin of trunk, excluding scrotum
8647	B825000	Carcinoma in situ of skin of breast
62939	B825100	Carcinoma in situ of skin of chest wall NOS
39390	B825200	Carcinoma in situ of skin of axilla
38032	B825300	Carcinoma in situ of skin of back

42212	B825400	Carcinoma in situ of skin of abdominal wall
57358	B825500	Carcinoma in situ of skin of groin
38777	B825600	Carcinoma in situ of skin of perineum
61321	B825700	Carcinoma in situ of skin of buttock
56374	B825800	Carcinoma in situ of perianal skin
60563	B825z00	Carcinoma in situ of skin of trunk NOS
46568	B826.00	Carcinoma in situ of skin of upper limb and shoulder
56554	B826000	Carcinoma in situ of skin of shoulder
57284	B826100	Carcinoma in situ of skin of upper arm
54790	B826200	Carcinoma in situ of skin of lower arm
49358	B826300	Carcinoma in situ of skin of hand
90339	B826z00	Carcinoma in situ of skin of upper limb or shoulder NOS
14815	B827.00	Carcinoma in situ of skin of lower limb and hip
708	B827.11	Carcinoma in situ of skin of leg
71655	B827000	Carcinoma in situ of skin of hip
46469	B827100	Carcinoma in situ of skin of thigh
69601	B827200	Carcinoma in situ of skin of knee
27542	B827300	Carcinoma in situ of skin of lower leg
67755	B827400	Carcinoma in situ of skin of foot
64630	B827z00	Carcinoma in situ of skin of lower limb or hip NOS
19686	B828.00	Melanoma in situ of skin
46536	B828000	Melanoma in situ of lip
37108	B828100	Melanoma in situ of eyelid, including canthus
72032	B828200	Melanoma in situ of ear and external auricular canal
97858	B828300	Melanoma in situ of scalp and neck
59768	B828400	Melanoma in situ of trunk
56694	B828500	Melanoma in situ of upper limb, including shoulder
47850	B828600	Melanoma in situ of lower limb, including hip
49572	B828700	Melanoma in situ of scalp
52332	B828800	Melanoma in situ of back of hand
71044	B828900	Melanoma in situ of back
54246	B828W00	Melanoma in situ, unspecified
61989	B828X00	Melanoma in situ of other and unspecified parts of face
57450	B82y.00	Carcinoma in situ of other specified sites of skin
63142	B82z.00	Carcinoma in situ of skin NOS
45681	B83..00	Carcinoma in situ of breast and genitourinary system
7833	B830.00	Carcinoma in situ of breast
10387	B830000	Lobular carcinoma in situ of breast
18694	B830100	Intraductal carcinoma in situ of breast
29898	B832.00	Carcinoma in situ of other and unspecified parts of uterus
61803	B832.11	Carcinoma in situ of body of uterus
7904	B832000	Carcinoma in situ of endometrium
44915	B833.00	Carcinoma in situ other and unspecified female genital organ
17137	B833000	Carcinoma in situ of ovary
59499	B833100	Carcinoma in situ of fallopian tube
34946	B833200	Carcinoma in situ of vagina
12119	B833300	Carcinoma in situ of vulva
69208	B833z00	Carcinoma in situ of female genital organs NOS
6328	B834.00	Carcinoma in situ of prostate
27311	B835.00	Carcinoma in situ of penis
107958	B836.00	Carcinoma in situ other and unspecified male genital organs
8177	B836000	Carcinoma in situ of testis
58879	B836300	Carcinoma in situ of scrotum
109831	B836z00	Carcinoma in situ of male genital organs NOS
7187	B837.00	Carcinoma in situ of bladder
68358	B83z.00	Carcinoma in situ of urinary organs NOS
38611	B8y..00	Carcinoma in situ of other and unspecified sites
58124	B8y0.00	Carcinoma in situ of eye
53349	B8yy.00	Carcinoma in situ of other specified site
8958	B8yy000	Carcinoma in situ of thyroid gland
46478	B8yy100	Carcinoma in situ of adrenal gland
58016	B8yy200	Carcinoma in situ of parathyroid gland
45909	B8yy300	Carcinoma in situ of pituitary gland
56640	B8yyz00	Carcinoma in situ of other specified site NOS
35136	B8z..00	Carcinoma in situ NOS
5136	B911013	Choriocarcinoma
43312	B936.11	Myeloma - solitary
38321	B936.12	Plasmacytoma NOS
50569	B93X.00	Neo/uncertn+unknwn behav/lymph,h'matopetc+rel tiss,unspcf
21868	BB02.00	[M]Neoplasm, malignant
22267	BB04.00	[M]Neoplasm, malig, uncertain whether primary or metastatic

8627	BB07.00	[M]Tumour cells, malignant
22156	BB08.00	[M]Malignant tumour, small cell type
24511	BB09.00	[M]Malignant tumour, giant cell type
32213	BB0A.00	[M]Malignant tumour, fusiform cell type
20564	BB11.00	[M]Carcinoma in situ NOS
21914	BB11.11	[M]Intraepithelial carcinoma NOS
8695	BB12.00	[M]Carcinoma NOS
3152	BB13.00	[M]Carcinoma, metastatic, NOS
9366	BB13.11	[M]Secondary carcinoma
16692	BB14.00	[M]Carcinomatosis
57336	BB16.00	[M]Epithelioma, malignant
25961	BB17.00	[M]Large cell carcinoma NOS
21609	BB18.00	[M]Carcinoma, undifferentiated type, NOS
12609	BB19.00	[M]Carcinoma, anaplastic type, NOS
26413	BB1A.00	[M]Pleomorphic carcinoma
48048	BB1B.00	[M]Giant cell and spindle cell carcinoma
35474	BB1C.00	[M]Giant cell carcinoma
6966	BB1D.00	[M]Spindle cell carcinoma
54276	BB1E.00	[M]Pseudosarcomatous carcinoma
69300	BB1F.00	[M]Polygonal cell carcinoma
61984	BB1G.00	[M]Spheroidal cell carcinoma
9291	BB1J.00	[M]Small cell carcinoma NOS
66541	BB1J.12	[M]Round cell carcinoma
9156	BB1K.00	[M]Oat cell carcinoma
67970	BB1L.00	[M]Small cell carcinoma, fusiform cell type
30988	BB1M.00	[M]Small cell carcinoma, intermediate cell
21217	BB1N.00	[M]Small cell-large cell carcinoma
106519	BB1P.00	[M]Non-small cell carcinoma
38651	BB21.00	[M]Papillary carcinoma in situ
10541	BB22.00	[M]Papillary carcinoma NOS
34395	BB24.00	[M]Verrucous carcinoma NOS
43717	BB24.11	[M]Verrucous epidermoid carcinoma
4852	BB24.12	[M]Verrucous squamous cell carcinoma
20807	BB26.00	[M]Papillary squamous cell carcinoma
67912	BB26.11	[M]Papillary epidermoid carcinoma
10134	BB29.00	[M]Squamous cell carcinoma in situ NOS
48182	BB29.11	[M]Epidermoid carcinoma in situ
19041	BB29.12	[M]Intraepidermal carcinoma NOS
19678	BB29.13	[M]Intraepithelial squamous cell carcinoma
1624	BB2A.00	[M]Squamous cell carcinoma NOS
56600	BB2A.11	[M]Epidermoid carcinoma NOS
57680	BB2A.12	[M]Spinous cell carcinoma
94873	BB2A.13	[M]Squamous cell carcinoma of skin NOS
24293	BB2B.00	[M]Squamous cell carcinoma, metastatic NOS
29787	BB2C.00	[M]Squamous cell carcinoma, keratinising type NOS
57513	BB2C.11	[M]Epidermoid carcinoma, keratinising type
59143	BB2D.00	[M]Squamous cell carcinoma, large cell, non-keratinising
41816	BB2E.00	[M]Squamous cell carcinoma, small cell, non-keratinising
45458	BB2F.00	[M]Squamous cell carcinoma, spindle cell type
31004	BB2G.00	[M]Adenoid squamous cell carcinoma
33497	BB2J.00	[M]Squamous cell carcinoma, microinvasive
45510	BB2M.00	[M]Lymphoepithelial carcinoma
3028	BB31.00	[M]Basal cell carcinoma NOS
59919	BB32.00	[M]Multicentric basal cell carcinoma
9885	BB33.00	[M]Basal cell carcinoma, morphoea type
29524	BB34.00	[M]Basal cell carcinoma, fibroepithelial type
35457	BB35.00	[M]Basosquamous carcinoma
13574	BB36.00	[M]Metatypical carcinoma
102417	BB3C.00	[M]Superficial basal cell carcinoma
102547	BB3D.00	[M]Basal cell carcinoma, nodular
103440	BB3E.00	[M]Basal cell carcinoma, micronodular
103178	BB3F.00	[M]Basal cell carcinoma, infiltrative
103066	BB3G.00	[M]Pigmented basal cell carcinoma
1950	BB4..00	[M]Transitional cell papillomas and carcinomas
21652	BB42.00	[M]Transitional cell carcinoma in situ
6436	BB43.00	[M]Transitional cell carcinoma NOS
12388	BB43.11	[M]Urothelial carcinoma
100111	BB46.00	[M]Schneiderian carcinoma
58798	BB47.00	[M]Transitional cell carcinoma, spindle cell type
38454	BB48.00	[M]Basaloid carcinoma
65216	BB49.00	[M]Cloacogenic carcinoma

9712	BB4A.00	[M]Papillary transitional cell carcinoma
101095	BB4B.00	[M]Grade 1 (Stage pTa) papillary urothelial/transit cell ca
102244	BB4C.00	[M]Grade 2 (Stage pTa) papillary urothelial/transit cell ca
101978	BB4D.00	[M]Grade 3 (Stage pTa) papillary urothelial/transit cell ca
33897	BB4z.00	[M]Transitional cell papilloma or carcinoma NOS
19091	BB5..00	[M]Adenomas and adenocarcinomas
2272	BB5..11	[M]Adenocarcinomas
27827	BB51.00	[M]Adenocarcinoma in situ
29170	BB51000	[M]Adenocarcinoma in situ in villous adenoma
37137	BB51100	[M]Adenocarcinoma in situ in tubulovillous adenoma
8930	BB52.00	[M]Adenocarcinoma NOS
44778	BB52000	[M]Adenocarcinoma in tubulovillous adenoma
5455	BB53.00	[M]Adenocarcinoma, metastatic, NOS
48223	BB54.00	[M]Scirrhous adenocarcinoma
27440	BB55.00	[M]Linitis plastica
71895	BB56.00	[M]Superficial spreading adenocarcinoma
28272	BB57.00	[M]Adenocarcinoma, intestinal type
59240	BB58.00	[M]Carcinoma, diffuse type
8101	BB5a.00	[M]Renal adenoma and carcinoma
10668	BB5a000	[M]Renal cell carcinoma
35467	BB5az00	[M]Renal adenoma or carcinoma NOS
34096	BB5b.00	[M]Granular cell carcinoma
8032	BB5B.00	[M]Pancreatic adenomas and carcinomas
63102	BB5B100	[M]Islet cell carcinoma
95609	BB5B300	[M]Insulinoma, malignant
32294	BB5B500	[M]Glucagonoma, malignant
98825	BB5B600	[M]Mixed islet cell and exocrine adenocarcinoma
21659	BB5Bz00	[M]Pancreatic adenoma or carcinoma NOS
26858	BB5C.00	[M]Gastrinoma and carcinomas
4217	BB5c.00	[M]Parathyroid adenomas and adenocarcinomas
49629	BB5C100	[M]Gastrinoma, malignant
43594	BB5Cz00	[M]Gastrinoma or carcinoma NOS
42169	BB5cz00	[M]Parathyroid adenoma or adenocarcinoma NOS
36031	BB5D.00	[M]Hepatobiliary tract adenomas and carcinomas
70516	BB5D.11	[M]Biliary tract adenomas and adenocarcinomas
8711	BB5D100	[M]Cholangiocarcinoma
40438	BB5D111	[M]Bile duct carcinoma
41313	BB5D300	[M]Bile duct cystadenocarcinoma
40240	BB5D500	[M]Hepatocellular carcinoma NOS
26814	BB5D512	[M]Hepatoma, malignant
25641	BB5D513	[M]Liver cell carcinoma
107299	BB5D700	[M]Combined hepatocellular carcinoma and cholangiocarcinoma
110147	BB5D711	[M]Hepatocholangiocarcinoma
46771	BB5D800	[M]Hepatocellular carcinoma, fibrolamellar
53987	BB5Dz00	[M]Hepatobiliary adenoma or carcinoma NOS
111238	BB5dz00	[M]Mixed cell adenoma or adenocarcinoma NOS
19263	BB5f.00	[M]Thyroid adenoma and adenocarcinoma
98781	BB5F.00	[M]Trabecular adenocarcinoma
21741	BB5f100	[M]Follicular adenocarcinoma NOS
21847	BB5f111	[M]Follicular carcinoma
59918	BB5f200	[M]Follicular adenocarcinoma, well differentiated type
61467	BB5f300	[M]Follicular adenocarcinoma, trabecular type
46761	BB5f600	[M]Papillary and follicular adenocarcinoma
68757	BB5f700	[M]Nonencapsulated sclerosing carcinoma
38685	BB5fz00	[M]Thyroid adenoma or adenocarcinoma NOS
60775	BB5h100	[M]Adrenal cortical carcinoma
33775	BB5J.00	[M]Adenoid cystic carcinoma
8606	BB5j.00	[M]Endometrioid adenomas and carcinomas
34879	BB5J.11	[M]Cylindroid adenocarcinoma
9447	BB5j200	[M]Endometrioid carcinoma
103034	BB5j500	[M]Endometrioid adenofibroma, malignant
28388	BB5jz00	[M]Endometrioid adenoma or carcinoma NOS
50140	BB5K.00	[M]Cribriform carcinoma
18255	BB5L.00	[M]Adenomatous and adenocarcinomatous polyps
52326	BB5L100	[M]Adenocarcinoma in adenomatous polyp
55741	BB5L200	[M]Adenocarcinoma in situ in adenomatous polyp
73434	BB5L300	[M]Adenocarcinoma in multiple adenomatous polyps
36286	BB5Lz00	[M]Adenomatous or adenocarcinomatous polyp NOS
6746	BB5M.00	[M]Tubular adenomas and adenocarcinomas
60045	BB5M100	[M]Tubular adenocarcinoma
39148	BB5Mz00	[M]Tubular adenoma or adenocarcinoma NOS

41702	BB5N.00	[M]Adenomatous and adenocarcinomatous polyps of colon
19731	BB5N.11	[M]Adenoma or or adenocarcinoma in polyposis coli
73275	BB5N100	[M]Adenocarcinoma in adenomatous polposis coli
39875	BB5Nz00	[M]Adenomatous or adenocarcinomatous polyps of the colon NOS
94083	BB5P.00	[M]Solid carcinoma NOS
34110	BB5R100	[M]Carcinoid tumour, malignant
23081	BB5R111	[M]Carcinoid bronchial adenoma
100625	BB5R500	[M]Carcinoid tumour, nonargentaffin, malignant
55468	BB5R600	[M]Mucocarcinoid tumour, malignant
69210	BB5R611	[M]Goblet cell tumour
56794	BB5R800	[M]Adenocarcinoid tumour
26253	BB5R900	[M]Neuroendocrine carcinoma
32641	BB5RA00	[M]Merkel cell carcinoma
26848	BB5S.00	[M]Respiratory tract adenomas and adenocarcinomas
34015	BB5S200	[M]Bronchiolo-alveolar adenocarcinoma
36530	BB5S211	[M]Alveolar cell carcinoma
16723	BB5S212	[M]Bronchiolar carcinoma
57802	BB5S400	[M]Alveolar adenocarcinoma
36221	BB5Sz00	[M]Respiratory tract adenoma or adenocarcinoma NOS
42273	BB5T.00	[M]Papillary adenomas and adenocarcinomas
35348	BB5T100	[M]Papillary adenocarcinoma NOS
96494	BB5Tz00	[M]Papillary adenoma or adenocarcinoma NOS
6920	BB5U.00	[M]Villous adenomas and adenocarcinomas
67342	BB5U100	[M]Adenocarcinoma in villous adenoma
27849	BB5U200	[M]Villous adenocarcinoma
50108	BB5Uz00	[M]Villous adenoma or adenocarcinoma NOS
26120	BB5V.00	[M]Pituitary adenomas and carcinomas
68456	BB5V100	[M]Chromophobe carcinoma
36876	BB5V311	[M]Eosinophil carcinoma
72277	BB5V700	[M]Basophil carcinoma
40622	BB5V711	[M]Mucoid cell carcinoma
57422	BB5Vz00	[M]Pituitary adenoma or carcinoma NOS
62199	BB5W.00	[M]Oxyphilic adenomas and adenocarcinomas
71497	BB5W100	[M]Oxyphilic adenocarcinoma
29008	BB5W111	[M]Hurthle cell adenocarcinoma
53129	BB5W112	[M]Oncytic adenocarcinoma
73662	BB5Wz00	[M]Oxyphilic adenoma or adenocarcinoma NOS
36882	BB5X.00	[M]Clear cell adenomas and adenocarcinomas
37354	BB5X100	[M]Clear cell adenocarcinoma NOS
72192	BB5Xz00	[M]Clear cell adenoma or adenocarcinoma NOS
16902	BB5y000	[M]Basal cell adenocarcinoma
61764	BB5y100	[M]Vipoma
49900	BB5y200	[M]Klatskin's tumour
35975	BB5z.00	[M]Adenoma or adenocarcinoma NOS
95046	BB60.00	[M]Skin appendage adenoma and carcinoma
68783	BB60100	[M]Skin appendage carcinoma
52496	BB61.00	[M]Sweat gland adenoma and adenocarcinomas
71627	BB61200	[M]Sweat gland adenocarcinoma
24312	BB62.00	[M]Apocrine adenoma and adenocarcinomas
38575	BB62100	[M]Apocrine adenocarcinoma
104973	BB62z00	[M]Apocrine adenoma or adenocarcinoma NOS
28291	BB69.00	[M]Sebaceous adenoma and adenocarcinoma
34269	BB69100	[M]Sebaceous adenocarcinoma
91842	BB69z00	[M]Sebaceous adenoma or adenocarcinoma NOS
67913	BB6A.00	[M]Ceruminous adenoma and adenocarcinoma
28625	BB71.00	[M]Mucoepidermoid carcinoma
34984	BB80.00	[M]Cystadenoma and carcinoma
34000	BB80100	[M]Cystadenocarcinoma NOS
65207	BB80z00	[M]Cystadenoma or carcinoma NOS
17151	BB81.11	[M]Ovarian cystadenoma or carcinoma
38442	BB81200	[M]Serous cystadenocarcinoma, NOS
98696	BB81400	[M]Papillary cystadenoma, borderline malignancy
65051	BB81500	[M]Papillary cystadenocarcinoma, NOS
44930	BB81800	[M]Papillary serous cystadenocarcinoma
95150	BB81B00	[M]Serous surface papillary carcinoma
28396	BB81D00	[M]Mucinous cystadenoma, borderline malignancy
51656	BB81E00	[M]Mucinous cystadenocarcinoma NOS
66876	BB81E11	[M]Pseudomucinous adenocarcinoma
54749	BB81H00	[M]Papillary mucinous cystadenocarcinoma
21131	BB81J00	[M]Serous cystadenoma, borderline malignancy
46113	BB81K00	[M]Papillary cystadenoma, borderline malignancy

6203	BB81M00	[M]Papillary serous cystadenoma, borderline malignancy
40632	BB82.00	[M]Mucinous adenoma and adenocarcinoma
12497	BB82100	[M]Mucinous adenocarcinoma
30416	BB82111	[M]Colloid adenocarcinoma
95008	BB82112	[M]Gelatinous adenocarcinoma
55429	BB82113	[M]Mucoid adenocarcionoma
59284	BB82114	[M]Mucous adenocarcinoma
64796	BB82z00	[M]Mucinous adenoma or adenocarcinoma NOS
44074	BB84.00	[M]Mucin-producing adenocarcinoma
39038	BB85.00	[M]Signet ring carcinoma
61588	BB85000	[M]Signet ring cell carcinoma
54874	BB85100	[M]Metastatic signet ring cell carcinoma
94438	BB85z00	[M]Signet ring carcinoma NOS
8351	BB91.00	[M]Infiltrating duct carcinoma
21833	BB91.11	[M]Duct carcinoma NOS
30189	BB91000	[M]Intraductal papillary adenocarcinoma with invasion
39760	BB91100	[M]Infiltrating duct and lobular carcinoma
62871	BB92.00	[M]Comedocarcinoma, noninfiltrating
58131	BB93.00	[M]Comedocarcinoma NOS
40359	BB94.00	[M]Juvenile breast carcinoma
67701	BB94.11	[M]Secretory breast carcinoma
16677	BB9B.00	[M]Medullary carcinoma NOS
47920	BB9B.11	[M]C cell carcinoma
50946	BB9C.00	[M]Medullary carcinoma with amyloid stroma
112200	BB9C.11	[M]Solid carcinoma with amyloid stroma
98883	BB9D.00	[M]Medullary carcinoma with lymphoid stroma
21861	BB9E.00	[M]Lobular carcinoma in situ
9956	BB9E000	[M]Intraductal carcinoma and lobular carcinoma in situ
12427	BB9F.00	[M]Lobular carcinoma NOS
7319	BB9G.00	[M]Infiltrating ductular carcinoma
32472	BB9H.00	[M]Inflammatory carcinoma
12300	BB9J.00	[M]Paget's disease, mammary
60803	BB9J.11	[M]Paget's disease, breast
42542	BB9K.00	[M]Paget's disease and infiltrating breast duct carcinoma
12480	BB9K000	[M]Paget's disease and intraductal carcinoma of breast
24523	BB9L.00	[M]Paget's disease, extramammary, exc Paget's disease bone
3969	BB9M.00	[M]Intracystic carcinoma NOS
37688	BBA2.00	[M]Acinar cell carcinoma
50151	BBa3.00	[M]Pineoblastoma
21758	BBa5.00	[M]Chordoma
12309	BBb..00	[M]Gliomas
12580	BBB0.00	[M]Adenosquamous carcinoma
31574	BBb0.00	[M]Glioma, malignant
8523	BBb0.11	[M]Glioma NOS
34252	BBb0.12	[M]Gliosarcoma
38551	BBb1.00	[M]Gliomatosis cerebri
16146	BBB2.00	[M]Adenocarcinoma with squamous metaplasia
68808	BBb2.00	[M]Mixed glioma
8524	BBb2.11	[M]Adenoacanthoma
39386	BBB2.11	[M]Mixed glioma
42553	BBB3.00	[M]Adenocarcinoma with cartilaginous and osseous metaplasia
94810	BBB4.00	[M]Adenocarcinoma with spindle cell metaplasia
66000	BBB5.00	[M]Adenocarcinoma with apocrine metaplasia
59415	BBB6100	[M]Thymoma, malignant
38770	BBB7.00	[M]Epithelial-myoepithelial carcinoma
20084	BBb7.00	[M]Ependymoma NOS
52751	BBb8.00	[M]Ependymoma, anaplastic type
46769	BBb8.11	[M]Ependymoblastoma
41695	BBba.00	[M]Primitive neuroectodermal tumour
107884	BBba000	[M]Peripheral neuroectodermal tumour
8547	BBbB.00	[M]Astrocytoma NOS
27748	BBbB.11	[M]Astrocytic glioma
8328	BBbC.00	[M]Astrocytoma, anaplastic type
45531	BBbE.00	[M]Gemistocytic astrocytoma
27846	BBbF.00	[M]Fibrillary astrocytoma
30273	BBbG.00	[M]Pilocytic astrocytoma
61783	BBbG.11	[M]Juvenile astrocytoma
98800	BBbG.12	[M]Piloid astrocytoma
103047	BBbH.00	[M]Spongioblastoma NOS
50235	BBbK.00	[M]Astroblastoma
23083	BBbL.00	[M]Glioblastoma NOS

9575	BBbL.11	[M]Glioblastoma multiforme
66064	BBbM.00	[M]Giant cell glioblastoma
27744	BBbQ.00	[M]Oligodendroglioma NOS
49186	BBbR.00	[M]Oligodendroglioma, anaplastic type
46404	BBbS.00	[M]Oligodendroblastoma
34763	BBbT.00	[M]Medulloblastoma NOS
65952	BBbU.00	[M]Desmoplastic medulloblastoma
31767	BBbV.00	[M]Medullomyoblastoma
37473	BBbW.00	[M]Cerebellar sarcoma NOS
27653	BBbz.00	[M]Glioma NOS
67587	BBbZ.00	[M]Pleomorphic xanthoastrocytoma
54284	BBc..00	[M]Neuroepitheliomatous neoplasms
39121	BBc0100	[M]Ganglioneuroblastoma
2123	BBc1.00	[M]Neuroblastoma NOS
67288	BBc2.00	[M]Medulloepithelioma NOS
107681	BBc3.00	[M]Teratoid medulloepithelioma
31609	BBC4.00	[M]Granulosa cell tumour, malignant
97961	BBc4.00	[M]Neuroepithelioma NOS
28836	BBc9.00	[M]Retinoblastomas
103883	BBc9100	[M]Retinoblastoma, undifferentiated type
48952	BBc9z00	[M]Retinoblastoma NOS
29580	BBCA.00	[M]Sertoli cell carcinoma
58902	BBcA.00	[M]Olfactory neurogenic tumour
51878	BBcC.00	[M]Aesthesioneuroblastoma
39388	BBcC.11	[M]Olfactory neuroblastoma
95373	BBCC100	[M]Leydig cell tumour, malignant
106131	BBcD.11	[M]Olfactory neuroepithelioma
99491	BBcz.00	[M]Neuroepitheliomatous neoplasm NOS
95818	BBD1.00	[M]Paraganglioma, malignant
27363	BBd2.00	[M]Meningioma, malignant
60347	BBd2.11	[M]Leptomeningeal sarcoma
96798	BBd2.12	[M]Meningothelial sarcoma
65047	BBDA.00	[M]Phaeochromocytoma, malignant
50605	BBdB.00	[M]Glomangiosarcoma
106134	BBDB.00	[M]Meningeal sarcomatosis
105166	BBDB.11	[M]Glomoid sarcoma
579	BBE1.00	[M]Malignant melanoma NOS
24551	BBE1.11	[M]Melanocarcinoma
44157	BBE1.13	[M]Melanosarcoma NOS
67966	BBE1.14	[M]Naevocarcinoma
51353	BBE1000	[M]Malignant melanoma, regressing
58835	BBE1100	[M]Desmoplastic melanoma, malignant
20982	BBE2.00	[M]Nodular melanoma
62941	BBe2.00	[M]Neurofibrosarcoma
68889	BBE4.00	[M]Balloon cell melanoma
69981	BBe7.00	[M]Neurilemmoma, malignant
37477	BBe7.11	[M]Schwannoma, malignant
40492	BBe9.00	[M]Triton tumour, malignant
17232	BBEA.00	[M]Amelanotic melanoma
63574	BBEC.00	[M]Malignant melanoma in junctional naevus
62088	BBEG.00	[M]Malignant melanoma in Hutchinson's melanotic freckle
11922	BBEG.11	[M]Lentigo maligna melanoma
22692	BBEG000	[M]Acral lentiginous melanoma, malignant
24208	BBEH.00	[M]Superficial spreading melanoma
73251	BBEM.00	[M]Malignant melanoma in giant pigmented naevus
23085	BBEP.00	[M]Epithelioid cell melanoma
44061	BBEQ.00	[M]Spindle cell melanoma NOS
92293	BBES.00	[M]Spindle cell melanoma, type B
40303	BBET.00	[M]Mixed epithelioid and spindle melanoma
68447	BBEV.00	[M]Blue naevus, malignant
8085	BBF1.00	[M]Sarcoma NOS
71869	BBf2.00	[M]Alveolar soft part sarcoma
31026	BBF3.00	[M]Spindle cell sarcoma
97463	BBF4.00	[M]Giant cell sarcoma (except of bone)
46581	BBF4.11	[M]Pleomorphic cell sarcoma
58837	BBF5.00	[M]Small cell sarcoma
69844	BBF5.11	[M]Round cell sarcoma
62396	BBF6.00	[M]Epithelioid cell sarcoma
17178	BBg..00	[M]Lymphomas, NOS or diffuse
31323	BBG1.00	[M]Fibrosarcoma NOS
36114	BBg1.00	[M]Malignant lymphoma NOS

1483	BBg1.11	[M]Lymphoma NOS
23711	BBg1000	[M]Malignant lymphoma, diffuse NOS
16460	BBg2.00	[M]Malignant lymphoma, non Hodgkin's type
3371	BBg2.11	[M]Non Hodgkins lymphoma
8088	BBG3.00	[M]Fibromyxosarcoma
71117	BBg3.00	[M]Malignant lymphoma, undifferentiated cell type NOS
46931	BBg4.00	[M]Malignant lymphoma, stem cell type
69301	BBg5.00	[M]Malignant lymphoma, convoluted cell type NOS
99655	BBg6.00	[M]Lymphosarcoma NOS
41754	BBg7.00	[M]Malignant lymphoma, lymphoplasmacytoid type
48253	BBG8.00	[M]Malignant lymphoma, immunoblastic type
95024	BBg8.00	[M]Infantile fibrosarcoma
94286	BBG8.11	[M]Congenital fibrosarcoma
68964	BBgA.00	[M]Malignant lymphoma, centroblastic-centrocytic, diffuse
41841	BBgB.00	[M]Malignant lymphoma, follicular centre cell NOS
69980	BBgC.00	[M]Malignant lymphoma, lymphocytic, well differentiated NOS
21463	BBgC.11	[M]Lymphocytic lymphoma NOS
60504	BBgC.12	[M]Lymphocytic lymphosarcoma NOS
51852	BBgD.00	[M]Malig lymphoma, lymphocytic, intermediate different NOS
39906	BBgE.00	[M]Malignant lymphoma, centrocytic
37680	BBGF.00	[M]Fibrous histiocytoma, malignant
72196	BBgG.00	[M]Malignant lymphoma, lymphocytic, poorly different NOS
67203	BBgG.11	[M]Lymphoblastic lymphosarcoma NOS
34352	BBgG.12	[M]Lymphoblastic lymphoma NOS
52591	BBgG.13	[M]Lymphoblastoma NOS
72241	BBgH.00	[M]Prolymphocytic lymphosarcoma
60275	BBGJ.00	[M]Malignant lymphoma, centroblastic type NOS
96231	BBgJ.00	[M]Fibroxanthoma, malignant
35034	BBGJ.11	[M]Fibroxanthosarcoma
66603	BBgK.00	[M]Malig lymphoma, follicular centre cell, non-cleaved NOS
26881	BBGL.00	[M]Dermatofibroma protuberans
46877	BBgL.00	[M]Malignant lymphoma, small lymphocytic NOS
31726	BBGM.00	[M]Malignant lymphoma, small cleaved cell, diffuse
31772	BBgM.00	[M]Dermatofibrosarcoma NOS
61251	BBgN.00	[M]Malign lymphoma,lymphocytic,intermediate differn, diffuse
31090	BBGP.00	[M]Pigmented dermatofibrosarcoma protuberans
71652	BBgP.00	[M]Malignant lymphoma, mixed small and large cell, diffuse
58015	BBgQ.00	[M]Malignant lymphomatous polyposis
33869	BBgR.00	[M]Malignant lymphoma, large cell, diffuse NOS
63994	BBgS.00	[M]Malignant lymphoma, large cell, cleaved, diffuse
71619	BBgT.00	[M]Malignant lymphoma, large cell, noncleaved, diffuse
51680	BBgV.00	[M]Malignant lymphoma, small cell, noncleaved, diffuse
51895	BBgz.00	[M]Lymphoma, diffuse or NOS
106137	BBh..00	[M]Reticulosarcomas
72433	BBh0.00	[M]Reticulosarcoma NOS
49825	BBh0.11	[M]Reticulum cell sarcoma NOS
21732	BBH1.00	[M]Myxosarcoma
100544	BBh2.00	[M]Reticulosarcoma, nodular
20710	BBj..00	[M]Hodgkin's disease
61997	BBj0.00	[M]Hodgkin's disease NOS
101429	BBj0.11	[M]Lymphogranuloma, malignant
28599	BBJ1.00	[M]Liposarcoma NOS
56041	BBj1.00	[M]Hodgkin's disease, lymphocytic predominance
101923	BBJ1.11	[M]Fibroliposarcoma
65584	BBj1000	[M]Hodgkin,s disease, lymphocytic predominance, diffuse
31537	BBj1100	[M]Hodgkin,s disease, lymphocytic predominance, nodular
51285	BBj2.00	[M]Hodgkin's disease, mixed cellularity
28628	BBJ3.00	[M]Liposarcoma, well differentiated type
96183	BBj4.00	[M]Hodgkin's disease,lymphocytic depletion,diffuse fibrosis
56676	BBJ5.00	[M]Myxoid liposarcoma
60127	BBJ5.12	[M]Myxoliposarcoma
42198	BBJ6.00	[M]Hodgkin's disease, nodular sclerosis NOS
103708	BBj6.00	[M]Round cell liposarcoma
40508	BBj6000	[M]Hodgkin,s disease, nodular sclerosis, lymphocytic predom
64343	BBj6100	[M]Hodgkin,s disease, nodular sclerosis, mixed cellularity
31741	BBj6200	[M]Hodgkin,s disease, nodular sclerosis, lymphocytic deplet
55947	BBJ7.00	[M]Pleomorphic liposarcoma
99200	BBj7.00	[M]Hodgkin's disease, nodular sclerosis, cellular phase
59651	BBJ8.00	[M]Mixed type liposarcoma
89230	BBj9.00	[M]Hodgkin's granuloma
7856	BBJH.00	[M]Dedifferentiated liposarcoma

42769	BBjz.00	[M]Hodgkin's disease NOS
20437	BBk..00	[M]Lymphomas, nodular or follicular
63699	BBk0.00	[M]Malignant lymphoma, nodular NOS
64947	BBk0.11	[M]Brill - Symmers' disease
27562	BBk0.12	[M]Follicular lymphosarcoma NOS
49253	BBk0.13	[M]Giant follicular lymphoma
10588	BBK0200	[M]Leiomyosarcoma NOS
73916	BBK0400	[M]Epithelioid leiomyosarcoma
64596	BBK0700	[M]Myxoid leiomyosarcoma
67019	BBK1100	[M]Angiomyosarcoma
98961	BBk2.00	[M]Malignant lymphoma, centroblastic-centrocytic, follicular
55268	BBK2100	[M]Myosarcoma
106970	BBk3.00	[M]Malig lymphoma, lymphocytic, well differentiated,nodular
31421	BBK3100	[M]Rhabdomyosarcoma NOS
57505	BBK3200	[M]Pleomorphic rhabdomyosarcoma
105944	BBK3300	[M]Mixed cell rhabdomyosarcoma
48275	BBK3600	[M]Embryonal rhabdomyosarcoma
63247	BBK3611	[M]Sarcoma botryoides
42082	BBK3700	[M]Alveolar rhabdomyosarcoma
39883	BBk5.00	[M]Malig lymp, follicular centre cell, cleaved, follicular
97852	BBk7.00	[M]Malignant lymphoma, centroblastic type, follicular
58953	BBk8.00	[M]Malig lymp,follicular centre cell,noncleaved,follicular
40513	BBkz.00	[M]Lymphoma, nodular or follicular NOS
46967	BBl..00	[M]Mycosis fungoides
34030	BBL0.00	[M]Endometrial stromal sarcoma
95464	BBl0.00	[M]Mycosis fungoides
97756	BBl1.00	[M]Sezary's disease
66607	BBL4.00	[M]Mixed tumour, malignant, NOS
21173	BBL5.00	[M]Mullerian mixed tumour
49811	BBL6.00	[M]Mesodermal mixed tumour
21681	BBL7100	[M]Nephroblastoma NOS
36870	BBL7111	[M]Adenosarcoma
17314	BBL7112	[M]Wilms' tumour
105862	BBL7300	[M]Mesenchymal nephroblastoma
57677	BBL8.00	[M]Hepatoblastoma
106889	BBL8.11	[M]Embryonal hepatoma
19334	BBL9.00	[M]Carcinosarcoma NOS
67934	BBLA.00	[M]Carcinosarcoma, embryonal type
61082	BBLA.11	[M]Pneumoblastoma
87003	BBLC100	[M]Mesenchymoma, malignant
98797	BBLD.00	[M]Embryonal sarcoma
63518	BBLE.00	[M]Adenosarcoma
37510	BBLG.00	[M]Carcinoma in pleomorphic adenoma
17212	BBLH.00	[M]Rhabdoid sarcoma
18771	BBLJ.00	[M]Clear cell sarcoma of kidney
48348	BBLM.00	[M]Pulmonary blastoma
99695	BBlz.00	[M]Mycosis fungoides NOS
63973	BBm0.00	[M]Microglioma
70383	BBM0100	[M]Brenner tumour, malignant
63239	BBm1.00	[M]Malignant histiocytosis
70740	BBm1.11	[M]Malignant reticulosis
47330	BBm2.00	[M]Histiocytic medullary reticulosis
59593	BBm3.00	[M]Letterer - Siwe disease
45768	BBm3.12	[M]Acute progressive histiocytosis X
57544	BBm4.00	[M]True histiocytic lymphoma
40766	BBm5.00	[M] Peripheral T-cell lymphoma NOS
26135	BBm6.00	[M] Alpha heavy chain disease
31492	BBM9.00	[M] Monocytoid B-cell lymphoma
59251	BBm9.00	[M]Cystosarcoma phyllodes, malignant
16774	BBmD.00	[M] Cutaneous lymphoma
52593	BBmE.00	[M] Gamma heavy chain disease
18383	BBmH.00	[M] Large cell lymphoma
68353	BBmJ.00	[M] Angioendotheliomatosis
9172	BBmK.00	[M]Waldenstrom's macroglobulinaemia
31671	BBn0.00	[M]Plasma cell myeloma
18744	BBn0.11	[M]Multiple myeloma
3672	BBn0.12	[M]Myeloma NOS
53647	BBn0.13	[M]Myelomatosis
39490	BBn0.14	[M]Plasmacytic myeloma
50379	BBN1.00	[M]Synovial sarcoma NOS
63864	BBN2.00	[M]Plasmacytoma NOS

105073	BBn2.00	[M]Synovial sarcoma, spindle cell type
102164	BBn2.11	[M]Monostotic myeloma
73135	BBn2.12	[M]Solitary myeloma
99702	BBn3.00	[M]Plasma cell tumour, malignant
57796	BBN4.00	[M]Synovial sarcoma, biphasic type
63286	BBN5.00	[M]Clear cell sarcoma of tendons and aponeuroses
27509	BBP1.00	[M]Mesothelioma, malignant
94239	BBp1.00	[M]Mast cell sarcoma
67339	BBp2.00	[M]Malignant mastocytosis
104720	BBP3.11	[M]Sarcomatoid mesothelioma
47734	BBP5.00	[M]Epithelioid mesothelioma, malignant
86820	BBP7.00	[M]Mesothelioma, biphasic type, malignant
21770	BBPX.00	[M]Mesothelioma, unspecified
32191	BBQ0.00	[M]Dysgerminoma
7476	BBQ1.00	[M]Seminomas
57084	BBQ1000	[M]Seminoma, anaplastic type
35223	BBQ1100	[M]Spermatocytic seminoma
9859	BBQ1z00	[M]Seminoma NOS
27971	BBQ2.00	[M]Germinoma
28941	BBQ3.00	[M]Embryonal carcinoma NOS
37621	BBQ4.00	[M]Endodermal sinus tumour
55658	BBQ4.12	[M]Orchioblastoma
20350	BBQ4.14	[M]Yolk sac tumour
102356	BBQ5.00	[M]Polyembryoma
33636	BBQ7200	[M]Teratoma, malignant, NOS
57087	BBQ7211	[M]Embryonal teratoma
43865	BBQ7212	[M]Immature teratoma
52493	BBQ7213	[M]Teratoblastoma, malignant
37542	BBQ7300	[M]Teratocarcinoma
61542	BBQ7400	[M]Malignant teratoma, undifferentiated type
21682	BBQ7500	[M]Malignant teratoma, intermediate type
65861	BBQ9.00	[M]Dermoid cyst with malignant transformation
71301	BBQA100	[M]Struma ovarii, malignant
35071	BBQB.00	[M]Mixed germ cell tumour
4637	BBr..00	[M]Leukaemias
40420	BBr0.00	[M]Leukaemias unspecified
41734	BBr0000	[M]Leukaemia NOS
6316	BBr0100	[M]Acute leukaemia NOS
22071	BBr0111	[M]Blast cell leukaemia
64963	BBr0112	[M]Blastic leukaemia
63570	BBr0113	[M]Stem cell leukaemia
72179	BBr0200	[M]Subacute leukaemia NOS
31750	BBr0300	[M]Chronic leukaemia NOS
72310	BBr0400	[M]Aleukaemic leukaemia NOS
59929	BBr0z00	[M]Leukaemia unspecified, NOS
67712	BBR2.00	[M]Choriocarcinoma
48155	BBr2.00	[M]Lymphoid leukaemias
12146	BBr2000	[M]Lymphoid leukaemia NOS
20635	BBr2011	[M]Lymphatic leukaemia
37410	BBr2100	[M]Acute lymphoid leukaemia
41500	BBr2300	[M]Chronic lymphoid leukaemia
46048	BBr2500	[M]Prolymphocytic leukaemia
50928	BBr2600	[M]Burkitt's cell leukaemia
29335	BBr2700	[M]Adult T-cell leukaemia/lymphoma
54627	BBR3.00	[M]Choriocarcinoma combined with teratoma
64618	BBr3.00	[M]Plasma cell leukaemias
29945	BBR4.00	[M]Malignant teratoma, trophoblastic
46444	BBr4.00	[M]Erythroleukaemias
70935	BBr4000	[M]Erythroleukaemia
100927	BBr4z00	[M]Erythroleukaemia NOS
35697	BBr6.00	[M]Myeloid leukaemias
71850	BBr6000	[M]Myeloid leukaemia NOS
37723	BBr6011	[M]Granulocytic leukaemia NOS
24317	BBr6012	[M]Myelosis NOS
54585	BBr6100	[M]Acute myeloid leukaemia
106483	BBr6200	[M]Subacute myeloid leukaemia
52942	BBr6300	[M]Chronic myeloid leukaemia
66694	BBr6311	[M]Naegeli-type monocytic leukaemia
57316	BBr6600	[M]Acute promyelocytic leukaemia
46263	BBr6700	[M]Acute myelomonocytic leukaemia
48049	BBr6800	[M]Chronic myelomonocytic leukaemia

62330	BBr6z00	[M]Other myeloid leukaemia NOS
106197	BBr7000	[M]Basophilic leukaemia
57713	BBr8.00	[M]Eosinophilic leukaemias
71377	BBr8000	[M]Eosinophilic leukaemia
107773	BBr8z00	[M]Eosinophilic leukaemia NOS
73088	BBr9000	[M]Monocytic leukaemia NOS
73066	BBrA.00	[M]Miscellaneous leukaemias
72222	BBrA100	[M]Megakaryocytic leukaemia
69299	BBrA111	[M]Thrombocytic leukaemia
96893	BBrA300	[M]Myeloid sarcoma
93944	BBrA311	[M]Chloroma
98009	BBrA312	[M]Granulocytic sarcoma
5915	BBrA400	[M]Hairy cell leukaemia
49327	BBrA500	[M]Acute megakaryoblastic leukaemia
102764	BBrA600	[M]Acute panmyelosis
37487	BBrA700	[M]Acute myelofibrosis
108316	BBrAz00	[M]Miscellaneous leukaemia NOS
42297	BBrz.00	[M]Leukaemia NOS
101271	BBs1.00	[M]Acute panmyelosis
62348	BBT1.00	[M]Haemangiosarcoma
22650	BBT1.11	[M]Angiosarcoma
98322	BBT7100	[M]Haemangioendothelioma, malignant
27439	BBTA.00	[M]Kaposi's sarcoma
105296	BBTD200	[M]Haemangiopericytoma, malignant
38481	BBTK.00	[M]Epithelioid haemangioendothelioma, malignant
57729	BBU1.00	[M]Lymphangiosarcoma
99665	BBV..11	[M]Juxtacortical osteogenic sarcoma
63571	BBV..12	[M]Parosteal osteosarcoma
105275	BBV..13	[M]Periosteal osteogenic sarcoma
31749	BBv0.00	[M]Monocytoid B-cell lymphoma
8660	BBV1.00	[M]Osteosarcoma NOS
49862	BBV1.11	[M]Osteoblastic sarcoma
59310	BBV1.12	[M]Osteochondrosarcoma
5052	BBV1.13	[M]Osteogenic sarcoma NOS
24539	BBV2.00	[M]Chondroblastic osteosarcoma
27965	BBv2.00	[M]AngiocentricT-cell lymphoma
21447	BBV3.00	[M]Fibroblastic osteosarcoma
22561	BBV4.00	[M]Telangiectatic osteosarcoma
60631	BBV5.00	[M]Osteosarcoma in Paget's disease of bone
4118	BBV9.00	[M]Myxoid chondrosarcoma
29337	BBVA.00	[M] Small cell osteosarcoma
7941	BBW4.00	[M]Chondrosarcoma NOS
68220	BBW4.11	[M]Fibrochondrosarcoma
63659	BBW6.00	[M]Juxtacortical chondrosarcoma
98559	BBW8.00	[M]Chondroblastoma, malignant
52684	BBW9.00	[M]Mesenchymal chondrosarcoma
68956	BBX1.00	[M]Giant cell tumour of bone, malignant
50859	BBX1.11	[M]Giant cell bone sarcoma
31673	BBX1.12	[M]Osteoclastoma, malignant
99797	BBX3.00	[M]Malignant giant cell tumour of soft parts
4473	BBY0.00	[M]Ewing's sarcoma
49023	BBY0.11	[M]Endothelial bone sarcoma
38593	BBY1.00	[M]Adamantinoma of long bones
67430	BBY1.11	[M]Tibial adamantinoma
107281	BBz0.00	[M]Neuroendocrine neoplasm
72443	BBZ2.00	[M]Odontogenic tumour, malignant
93175	BBZ2.11	[M]Intraosseous carcinoma
46741	BBZC.00	[M]Ameloblastic odontosarcoma
97593	BBZG.00	[M]Ameloblastoma, malignant
100267	BBZG.11	[M]Adamantinoma, malignant
68730	BBZN.00	[M]Ameloblastic fibrosarcoma
98483	BBZN.11	[M]Odontogenic fibrosarcoma
58973	Byu0.00	[X]Malignant neoplasm of lip, oral cavity and pharynx
35180	Byu1.00	[X]Malignant neoplasm of digestive organs
43490	Byu1100	[X]Other specified carcinomas of liver
45766	Byu1200	[X]Malignant neoplasm of intestinal tract, part unspecified
49292	Byu1300	[X]Malignant neoplsm/ill-defin sites within digestive system
35325	Byu2.00	[X]Malignant neoplasm of respiratory and intrathoracic orga
40595	Byu2000	[X]Malignant neoplasm of bronchus or lung, unspecified
66444	Byu2100	[X]Malignant neoplasm/overlap lesion/heart,mediastinm+pleura
111904	Byu2200	[X]Malignant neoplasm/upper resp tract, part unspecified

99096	Byu2300	[X]Malignant neopl/overlapping les/resp+intrathoracic organs
86997	Byu2400	[X]Malignant neoplasm/ill-defined sites within resp system
50292	Byu2500	[X]Malignant neoplasm of mediastinum, part unspecified
40749	Byu3.00	[X]Malignant neoplasm of bone and articular cartilage
73296	Byu3100	[X]Malignant neoplasm/bones+articular cartilage/limb,unspfd
63300	Byu3200	[X]Malignant neoplasm/overlap lesion/bone+articulr cartilage
43151	Byu3300	[X]Malignant neoplasm/bone+articular cartilage, unspecified
19144	Byu4.00	[X]Melanoma and other malignant neoplasms of skin
56925	Byu4000	[X]Malignant melanoma of other+unspecified parts of face
19444	Byu4100	[X]Malignant melanoma of skin, unspecified
57184	Byu4200	[X]Oth malignant neoplasm/skin of oth+unspecfd parts of face
56121	Byu4300	[X]Malignant neoplasm of skin, unspecified
40592	Byu5.00	[X]Malignant neoplasm of mesothelial and soft tissue
67034	Byu5000	[X]Mesothelioma of other sites
21715	Byu5011	[X]Mesothelioma of lung
30526	Byu5100	[X]Mesothelioma, unspecified
93665	Byu5300	[X]Kaposi's sarcoma, unspecified
101668	Byu5400	[X]Malignant neoplasm/peripheral nerves of trunk,unspecified
105072	Byu5500	[X]Mal neoplasm/overlap les/periph nerv+autonomic nerv systm
95671	Byu5700	[X]Malignant neoplasm of peritoneum, unspecified
91896	Byu5800	[X]Mal neoplasm/connective+soft tissue of trunk,unspecified
91457	Byu5900	[X]Malignant neoplasm/connective + soft tissue,unspecified
60162	Byu5A00	[X]Malignant neoplasm overlapping lesion of skin
98361	Byu5B00	[X]Kaposi's sarcoma of other sites
12499	Byu6.00	[X]Malignant neoplasm of breast
40598	Byu7.00	[X]Malignant neoplasm of female genital organs
64497	Byu7000	[X]Malignant neoplasm of uterine adnexa, unspecified
57756	Byu7100	[X]Malignant neoplasm/other specified female genital organs
55588	Byu7300	[X]Malignant neoplasm of female genital organ, unspecified
40671	Byu8.00	[X]Malignant neoplasm of male genital organs
57191	Byu8000	[X]Malignant neoplasm/other specified male genital organs
45262	Byu8200	[X]Malignant neoplasm of male genital organ, unspecified
35113	Byu9.00	[X]Malignant neoplasm of urinary tract
45260	Byu9000	[X]Malignant neoplasm of urinary organ, unspecified
35285	ByuA.00	[X]Malignant neoplasm of eye, brain and other parts of cent
68027	ByuA000	[X]Malignant neoplasm/other and unspecified cranial nerves
41515	ByuA100	[X]Malignant neoplasm/central nervous system, unspecified
63925	ByuA200	[X]Malignant neoplasm of meninges, unspecified
47633	ByuA300	[X]Malig neopl, overlap lesion brain & other part of CNS
40608	ByuB.00	[X]Malignant neoplasm of thyroid and other endocrine glands
64309	ByuB100	[X]Malignant neoplasm of endocrine gland, unspecified
35186	ByuC.00	[X]Malignant neoplasm of ill-defined, secondary and unspeci
39027	ByuC000	[X]Malignant neoplasm of other specified sites
96226	ByuC100	[X]Malignant neoplasm/overlap lesion/other+ill-defined sites
66163	ByuC200	[X]2ndry+unspcf malignant neoplasm lymph nodes/multi regions
57481	ByuC300	[X]Secondary malignant neoplasm/oth+unspc respiratory organs
88022	ByuC400	[X]Secondary malignant neoplasm/oth+unspcfd digestive organs
97091	ByuC500	[X]2ndry malignant neoplasm/bladder+oth+unsp urinary organs
68332	ByuC600	[X]2ndry malignant neoplasm/oth+unspec parts/nervous system
54253	ByuC700	[X]Secondary malignant neoplasm of other specified sites
52029	ByuC800	[X]Malignant neoplasm without specification of site
40740	ByuD.00	[X]Malignant neoplasms of lymphoid, haematopoietic and rela
43415	ByuD000	[X]Other Hodgkin's disease
67518	ByuD100	[X]Other types of follicular non-Hodgkin's lymphoma
98596	ByuD200	[X]Other types of diffuse non-Hodgkin's lymphoma
64336	ByuD300	[X]Other specified types of non-Hodgkin's lymphoma
102688	ByuD400	[X]Other malignant immunoproliferative diseases
67029	ByuD500	[X]Other lymphoid leukaemia
61693	ByuD600	[X]Other myeloid leukaemia
89762	ByuD700	[X]Other monocytic leukaemia
89329	ByuD800	[X]Other specified leukaemias
65165	ByuD900	[X]Other leukaemia of unspecified cell type
105025	ByuDA00	[X]Oth spcf mal neoplsm/lymphoid,haematopoietic+rltd tissue
72500	ByuDB00	[X]Mal neoplasm/lymphoid,haematopoietic+related tissu,unspcf
64515	ByuDC00	[X]Diffuse non-Hodgkin's lymphoma, unspecified
63375	ByuDE00	[X]Unspecified B-cell non-Hodgkin's lymphoma
8649	ByuDF00	[X]Non-Hodgkin's lymphoma, unspecified type
7940	ByuDF11	[X]Non-Hodgkin's lymphoma NOS
63598	ByuE.00	[X]Malignant neoplasms/independent (primary) multiple sites
64897	ByuE000	[X]Malignant neoplasms/independent(primary)multiple sites
102708	ByuF100	[X]Carcinoma in situ of other specified digestive organs

100781	ByuF300	[X]Carcinoma in situ of other parts of respiratory system
97628	ByuF600	[X]Melanoma in situ of other sites
101561	ByuF900	[X]Carcinoma in situ of skin, unspecified
72695	ByuFA00	[X]Carcinoma in situ of other parts of cervix
106003	ByuFC00	[X]Carcinoma in situ of oth+unspecified male genital organs
73261	ByuFF00	[X]Melanoma in situ, unspecified
53803	ByuFG00	[X]Other carcinoma in situ of breast
107587	C184.00	Multiple endocrine neoplasia syndrome type 1
10411	C333000	Waldenstrom's macroglobulinaemia
108235	C333011	Waldenstrom macroglobulinaemia
101350	C333100	Alpha heavy chain disease
99067	C333200	Gamma heavy chain disease
48145	D212000	Anaemia in ovarian carcinoma
30537	F373.00	Polyneuropathy in malignant disease
57551	F381100	Myasthenic syndrome due to other malignancy
49482	F396200	Myopathy due to malignant disease
60433	N330900	Osteoporosis in multiple myelomatosis
5069	PB0..00	Meckel's diverticulum
33781	PB0z.00	Meckel's diverticulum NOS
51029	PH32111	Mast cell disease
17521	PH32112	Mastocytosis
31561	ZV67600	[V]Follow-up examination aft surgery for malignant neoplasm
30547	ZV67700	[V]Follow-up exam after radiotherapy for malignant neoplasm
44421	ZV67A00	[V]Folow-up exam aft other treatment for malignant neoplasm
36321	ZV67B00	[V]Folow-up exam aft unspec treatment for malignant neoplasm

**CPRD Aurum: Cancers**		
**medcode**	**term**	**snomedconceptID**
3704012	Clark melanoma level 4	1556006
35572018	Clark melanoma level 3	21165006
50949017	Lymphoma stage I	30440004
62349016	Meckel's diverticulum	37373007
84190018	Clark melanoma level 2	50542000
94699019	Lymphoma stage III	56944001
110346017	Lymphoma stage IV	66445009
123978016	Mantle cell lymphoma	443487006
126918018	Lymphoma stage II	76422004
133678011	Acute panmyelosis	109991003
138122012	Clark melanoma level 1	83284001
142271018	Malignant pemphigus	85824006
144849016	Neuroblastoma	87364003
154082012	Letterer-Siwe disease of intrathoracic lymph nodes	93135004
154089015	Letterer-Siwe disease of lymph nodes of multiple sites	93139005
154231019	Malignant melanoma of perianal skin	93209006
154811011	Hodgkin's disease, nodular sclerosis of spleen	93518009
155060012	Malignant melanoma of skin	93655004
155068017	Malignant neoplasm of acromion	93661001
155072018	Malignant neoplasm of adrenal gland	363355002
155076015	Malignant tumour of anal canal	363352004
155078019	Malignant neoplasm of anterior mediastinum	449224009
155085015	Malignant neoplasm of aortic body	93677000
155089014	Malignant neoplasm of areola of female breast	93680004
155090017	Malignant neoplasm of areola of male breast	93681000
155155010	Malignant neoplasm of broad ligament	93728003
155168019	Malignant neoplasm of carotid body	447883002
155175018	Malignant neoplasm of cerebellum	449420002
155191015	Malignant neoplasm of clavicle	93757004
155193017	Malignant neoplasm of coccygeal body	93759001
155206014	Malignant neoplasm of cuboid	93769007
155207017	Malignant neoplasm of cystic duct	93770008
155209019	Malignant neoplasm of diaphragm	93772000
155211011	Malignant neoplasm of dorsal surface of tongue	187633009
155218017	Malignant neoplasm of endocervix	372097009
155222010	Malignant neoplasm of epicardium	93782004
155228014	Malignant neoplasm of ethmoid bone	93786001
155231010	Malignant neoplasm of exocervix	372099007
155246015	Malignant neoplasm of femur	93798006
155247012	Malignant neoplasm of fibula	93799003
155259011	Malignant neoplasm of frontal bone	93806005
155269017	Malignant neoplasm of glomus jugulare	93814004
155272012	Malignant neoplasm of great vessels	93817006

155287019	Malignant neoplasm of hilus of lung	93827000
155294016	Malignant neoplasm of ilium	93833009
155305011	Malignant neoplasm of intrahepatic bile ducts	187777008
155308013	Malignant neoplasm of ischium	93842002
155332018	Malignant neoplasm of lateral wall of oropharynx	448868009
155361017	Malignant neoplasm of main bronchus	372065009
155364013	Malignant neoplasm of male breast	372095001
155368011	Malignant neoplasm of mandible	448668007
155370019	Malignant neoplasm of maxilla	93888008
155402013	Malignant neoplasm of nasal bone	93916003
155417012	Malignant neoplasm of nipple of female breast	93924008
155418019	Malignant neoplasm of nipple of male breast	93925009
155421017	Malignant neoplasm of occipital bone	93927001
155425014	Malignant neoplasm of optic nerve	363498002
155441010	Malignant neoplasm of parametrium	448674007
155444019	Malignant neoplasm of parietal bone	93945001
155446017	Malignant neoplasm of parietal peritoneum	93947009
155447014	Malignant neoplasm of parietal pleura	449067008
155449012	Malignant neoplasm of patella	93950007
155452016	Malignant neoplasm of pelvic peritoneum	449377002
155457010	Malignant neoplasm of periadrenal tissue	93955002
155458017	Malignant neoplasm of perianal skin	93956001
155459013	Malignant neoplasm of pericardium	93957005
155468010	Primary malignant neoplasm of placenta	721567004
155473016	Malignant neoplasm of posterior mediastinum	448670003
155477015	Malignant neoplasm of presacral region	93973004
155479017	Malignant neoplasm of pubis	93975006
155483017	Malignant neoplasm of radius	93979000
155488014	Malignant tumour of rectum	363351006
155495017	Malignant neoplasm of rib	93990005
155501015	Malignant neoplasm of sacrococcygeal region	93995000
155503017	Malignant neoplasm of scapula	93997008
155517014	Malignant neoplasm of skin of ankle	94008001
155520018	Malignant neoplasm of skin of back	94011000
155521019	Malignant neoplasm of skin of breast	94012007
155522014	Malignant neoplasm of skin of buttock	94013002
155525011	Malignant neoplasm of skin of chin	94016005
155529017	Malignant neoplasm of skin of eyebrow	94020009
155533012	Malignant neoplasm of skin of finger	94023006
155534018	Malignant neoplasm of skin of foot	94024000
155536016	Malignant neoplasm of skin of forehead	94026003
155537013	Malignant neoplasm of skin of groin	94027007
155538015	Malignant neoplasm of skin of hand	94028002
155539011	Malignant neoplasm of skin of hip	94029005
155540013	Malignant neoplasm of skin of knee	94030000
155542017	Malignant neoplasm of skin of lip	94032008
155545015	Malignant neoplasm of skin of neck	94034009
155547011	Malignant neoplasm of skin of perineum	94036006
155550014	Malignant neoplasm of skin of shoulder	94039004
155552018	Malignant neoplasm of skin of thigh	94041003
155553011	Malignant neoplasm of skin of toe	94042005
155556015	Malignant neoplasm of skin of umbilicus	94044006
155582012	Malignant neoplasm of sphenoid bone	94066004
155592016	Malignant neoplasm of sternum	94073009
155604019	Malignant neoplasm of talus	94083008
155607014	Malignant neoplasm of temporal bone	94085001
155624015	Malignant neoplasm of tibia	94099002
155641018	Malignant neoplasm of ulna	94112009
155669014	Malignant neoplasm of ventral surface of tongue	94134006
155675017	Malignant neoplasm of visceral pleura	449308006
155677013	Malignant neoplasm of vomer	94142007
155681013	Malignant neoplasm of zygomatic bone	94145009
155682018	Malignant otitis externa	94146005
155711012	Secondary malignant neoplasm of adrenal gland	94161006
155773015	Secondary malignant neoplasm of bladder	94186002
155879014	Secondary malignant neoplasm of brain	94225005
155967013	Secondary malignant neoplasm of colon	94260004
156007019	Secondary malignant neoplasm of duodenum	94275007
156169017	Secondary malignant neoplasm of ileum	94335002
156231017	Secondary malignant neoplasm of jejunum	94357009
156237018	Secondary malignant neoplasm of kidney	94360002

156291015	Secondary malignant neoplasm of liver	94381002
156319016	Secondary malignant neoplasm of lung	94391008
156375015	Secondary malignant neoplasm of mediastinum	94409002
156493015	Secondary malignant neoplasm of ovary	94455000
156557015	Secondary malignant neoplasm of penis	94481001
156585018	Secondary malignant neoplasm of pleura	94493005
156607018	Secondary malignant neoplasm of prostate	94503003
156627017	Secondary malignant neoplasm of rectum	94513006
156699010	Secondary malignant neoplasm of skin of breast	94544002
156719018	Secondary malignant neoplasm of skin of face	94554003
156747019	Secondary malignant neoplasm of skin of neck	94566009
156765015	Secondary malignant neoplasm of skin of trunk	94575006
156777013	Secondary malignant neoplasm of skin	94579000
156883018	Secondary malignant neoplasm of testis	94623007
156919012	Secondary malignant neoplasm of tongue	94638008
156973018	Secondary malignant neoplasm of ureter	94659001
156977017	Secondary malignant neoplasm of urethra	94661005
156989015	Secondary malignant neoplasm of uterus	94665001
156997010	Secondary malignant neoplasm of vagina	94668004
157025019	Secondary malignant neoplasm of vulva	94681006
157075017	Mycosis fungoides of lymph nodes of axilla and upper limb	94710006
157091019	Myeloid sarcoma	94719007
157686016	Nodular lymphoma of intra-abdominal lymph nodes	95186006
157688015	Nodular lymphoma of intrathoracic lymph nodes	95188007
157692010	Nodular lymphoma of lymph nodes of multiple sites	95192000
157732017	Primary malignant neoplasm of liver	95214007
157745018	Reticulosarcoma of intra-abdominal lymph nodes	95224004
157752016	Reticulosarcoma of spleen	95231000
173816012	Overlapping malignant melanoma of skin	109267002
173941016	Mesothelioma of pericardium	109383000
173944012	Kaposi's sarcoma of skin	109386008
173946014	Kaposi's sarcoma of palate	109388009
173950019	Kaposi's sarcoma of lymph nodes	109391009
174518012	Malignant neoplasm of cloacogenic zone	363491008
174532014	Mesothelioma of peritoneum	109853004
174562015	Malignant neoplasm of descended testis	109876001
198006010	Diffuse large B-cell lymphoma	847741000000106
201612010	Acute myeloid leukaemia	91861009
201652012	Chronic lymphoid leukaemia	92814006
201656010	Chronic myeloid leukaemia	92818009
201658011	Di Guglielmo's disease	93451002
201743012	Plasma cell leukaemia	95210003
203313019	Mastocytosis	78745000
215912012	[M]Malignant lymphoma, small cleaved cell, diffuse	115244002
215916010	[M]Malignant reticulosis	128920006
216227011	Pathological fracture due to metastatic bone disease	134421000
260879014	Bone marrow: myeloma cells	167934009
287014015	Malignant neoplastic disease co-occurrent with human immunodeficiency virus infection	713572001
287031010	HIV disease resulting in multiple malignant neoplasms	713572001
288182013	[X]HIV disease resulting in other non-Hodgkin's lymphoma	442537007
288185010	Malignant neoplastic disease co-occurrent with human immunodeficiency virus infection	713572001
288186011	[X]HIV disease resulting in unspecified malignant neoplasm	86406008
288350019	Malignant neoplasm of upper lip, lipstick area	187601000
288351015	Malignant tumour of vermilion border of upper lip	363372009
288354011	Malignant neoplasm of lower lip, external	187604008
288355012	Malignant tumour of vermilion border of lower lip	363373004
288362015	Malignant neoplasm of upper lip, oral aspect	187606005
288363013	Malignant neoplasm of upper lip, inner aspect	187606005
288364019	Malignant neoplasm of upper lip, mucosa	187606005
288372017	Malignant neoplasm of upper lip, frenulum	187608006
288377011	Malignant neoplasm of upper lip, inner aspect NOS	187606005
288380012	Malignant neoplasm of lower lip, buccal aspect	187613005
288382016	Malignant neoplasm of lower lip, frenulum	187614004
288387010	Malignant neoplasm of lower lip, inner aspect NOS	271568003
288388017	Malignant tumour of labial mucosa	187622006
288390016	Malignant neoplasm of lip unspecified, frenulum	302815008
288391017	Malignant neoplasm of lip unspecified, mucosa	187622006
288393019	Malignant neoplasm of lip, oral aspect	187622006
288403012	Malignant neoplasm of lip, inner aspect NOS	187622006
288404018	Malignant neoplasm of overlapping lesion of lip	187624007

288406016	Malignant neoplasm of other sites of lip	363348004
288407013	Malignant neoplasm of vermilion border of lip unspecified	421249001
288408015	Malignant neoplasm of lip, unspecified, external	363348004
288409011	Malignant tumour of lipstick area of lip	275399006
288410018	Malignant neoplasm of lip, vermilion border NOS	421249001
288411019	Malignant neoplasm of base of tongue dorsal surface	187631006
288412014	Malignant neoplasm of fixed part of tongue NOS	363376007
288414010	Malignant neoplasm of anterior 2/3 of tongue dorsal surface	187634003
288417015	Malignant neoplasm of midline of tongue	187635002
288418013	Malignant neoplasm of dorsum of tongue NOS	187633009
288419017	Malignant neoplasm of tongue, tip and lateral border	187637005
288423013	Malignant neoplasm of anterior 2/3 of tongue ventral surface	187640005
288427014	Malignant neoplasm of frenulum linguae	187641009
288429012	Malignant neoplasm of ventral surface of tongue	1090271000000101
288430019	Malignant neoplasm of anterior 2/3 of tongue unspecified	363360003
288433017	Malignant neoplasm of tongue, junctional zone	187644001
288434011	Malignant neoplasm of other sites of tongue	363375006
288435012	Malignant neoplasm of tongue NOS	363375006
288437016	Malignant neoplasm of other major salivary glands	363378008
288438014	Malignant tumour of major salivary gland	363378008
288439018	Malignant neoplasm of other sites of gum	363382005
288440016	Malignant neoplasm of gum NOS	363382005
288443019	Malignant neoplasm of anterior portion of floor of mouth	187652003
288444013	Malignant neoplasm of lateral portion of floor of mouth	187653008
288448011	Malignant neoplasm of other sites of floor of mouth	363385007
288449015	Malignant tumour of floor of mouth	363385007
288450015	Malignant neoplasm of other and unspecified parts of mouth	363505006
288453018	Malignant neoplasm of vestibule of mouth	187658004
288454012	Malignant neoplasm of upper buccal sulcus	187659007
288459019	Malignant neoplasm of lower buccal sulcus	187660002
288460012	Malignant neoplasm of upper labial sulcus	187661003
288465019	Malignant neoplasm of lower labial sulcus	187662005
288468017	Malignant tumour of vestibule of mouth	187658004
288469013	Malignant tumour of palate	363390005
288470014	Malignant neoplasm of junction of hard and soft palate	187666008
288471013	Malignant neoplasm of palate NOS	363390005
288472018	Overlapping malignant neoplasm of mouth	1092831000000100
288473011	Malignant neoplasm of other specified mouth parts	363505006
288474017	Malignant neoplasm of mouth NOS	363505006
288478019	Malignant neoplasm tonsil NOS	363393007
288485015	Malignant neoplasm of faucial pillar	187675005
288486019	Malignant neoplasm of tonsillar pillar	187675005
288505015	Malignant tumour of tonsillar fossa	363394001
288506019	Malignant neoplasm of anterior epiglottis	187681002
288507011	Malignant neoplasm of epiglottis, free border	187682009
288508018	Malignant neoplasm of glossoepiglottic fold	187683004
288509014	Malignant neoplasm of anterior epiglottis NOS	187681002
288510016	Malignant neoplasm of junctional region of epiglottis	187685006
288515014	Malignant neoplasm of posterior wall of oropharynx	187688008
288516010	Malignant neoplasm of oropharynx, other specified sites	363392002
288517018	Malignant neoplasm of other specified site of oropharynx NOS	363392002
288518011	Malignant neoplasm of oropharynx NOS	363392002
288522018	Malignant neoplasm of nasopharynx	187692001
288529010	Malignant neoplasm of posterior wall of nasopharynx	187693006
288532013	Malignant neoplasm of pharyngeal tonsil	187694000
288533015	Malignant neoplasm of adenoid	187694000
288541015	Malignant tumour of posterior wall of nasopharynx	187693006
288546013	Malignant neoplasm of pharyngeal recess	187697007
288548014	Malignant neoplasm of opening of auditory tube	187698002
288550018	Malignant tumour of lateral wall of nasopharynx	363398003
288552014	Malignant neoplasm of anterior wall of nasopharynx	187700006
288554010	Malignant neoplasm of floor of nasopharynx	187701005
288556012	Malignant neoplasm of nasopharyngeal soft palate surface	187702003
288561014	Malignant tumour of anterior wall of nasopharynx	187700006
288563012	Malignant neoplasm of other specified site of nasopharynx	187692001
288564018	Malignant tumour of nasopharynx	187692001
288568015	Malignant neoplasm of posterior pharynx	187709007
288570012	Malignant neoplasm of other specified hypopharyngeal site	363399006
288571011	Malignant tumour of hypopharynx	363399006
288573014	Malignant neoplasm of pharynx unspecified	363507003
288578017	Malignant neoplasm of Waldeyer's ring	187716008

288582015	Malignant neoplasm of lip, oral cavity and pharynx NOS	271323007
288589012	Malignant neoplasm of cervical oesophagus	187722004
288594012	Malignant neoplasm of thoracic oesophagus	187723009
288597017	Malignant neoplasm of abdominal oesophagus	187724003
288602012	Malignant neoplasm of upper third of oesophagus	187725002
288607018	Malignant neoplasm of middle third of oesophagus	187726001
288608011	Malignant neoplasm of lower third of oesophagus	187727005
288614016	Malignant neoplasm of other specified part of oesophagus	363402007
288625013	Malignant neoplasm of cardia of stomach	187732006
288628010	Malignant neoplasm of cardiac orifice of stomach	187733001
288631011	Malignant neoplasm of gastro-oesophageal junction	187734007
288632016	Malignant neoplasm of cardio-oesophageal junction of stomach	187734007
288633014	Malignant neoplasm of cardia of stomach NOS	187732006
288635019	Malignant neoplasm of pylorus of stomach	187736009
288638017	Malignant neoplasm of pyloric canal of stomach	187738005
288639013	Malignant neoplasm of pylorus of stomach NOS	187736009
288641014	Malignant neoplasm of pyloric antrum of stomach	187740000
288644018	Malignant neoplasm of fundus of stomach	187741001
288647013	Malignant neoplasm of body of stomach	187742008
288649011	Malignant tumour of lesser curve of stomach	269459004
288650011	Malignant neoplasm of greater curve of stomach unspecified	269460009
288652015	Malignant neoplasm of other specified site of stomach	363349007
288653013	Malignant neoplasm of anterior wall of stomach	1090981000000107
288654019	Malignant neoplasm of posterior wall of stomach	1090971000000105
288655018	Malignant neoplasm of other specified site of stomach NOS	363349007
288656017	Malignant tumour of stomach	363349007
288659012	Malignant neoplasm of Meckel's diverticulum	187752007
288663017	Malignant neoplasm of small intestine NOS	363509000
288670017	Malignant neoplasm, overlapping lesion of colon	187757001
288671018	Malignant neoplasm of other specified sites of colon	363406005
288675010	Malignant neoplasm of rectum, rectosigmoid junction and anus	187760008
288685011	Malignant tumour of anus	363490009
288688013	Malignant neoplasm of liver and intrahepatic bile ducts	187767006
288690014	Primary carcinoma of liver	187769009
288692018	Primary malignant neoplasm of liver NOS	95214007
288694017	Malignant neoplasm of interlobular bile ducts	187773007
288697012	Malignant neoplasm of intrahepatic canaliculi	187776004
288698019	Malignant neoplasm of intrahepatic gall duct	187777008
288699010	Malignant neoplasm of intrahepatic bile ducts NOS	187777008
288700011	Malignant neoplasm of liver	93870000
288702015	Malignant neoplasm of liver and intrahepatic bile ducts NOS	187767006
288707014	Malignant neoplasm of hepatic duct	187784000
288711015	Malignant neoplasm of sphincter of Oddi	187786003
288712010	Malignant neoplasm of extrahepatic bile ducts NOS	363416002
288717016	Malignant neoplasm of body of pancreas	187791002
288720012	Malignant neoplasm of tail of pancreas	187792009
288723014	Malignant neoplasm of pancreatic duct	187793004
288727010	Malignant neoplasm of Islets of Langerhans	187794005
288731016	Malignant neoplasm of other specified sites of pancreas	363418001
288734012	Malignant neoplasm of ectopic pancreatic tissue	187798008
288735013	Malignant neoplasm of specified site of pancreas NOS	363418001
288736014	Malignant neoplasm of pancreas NOS	363418001
288739019	Malignant neoplasm of retroperitoneum and peritoneum	187801002
288741018	Malignant neoplasm of perinephric tissue	187803004
288742013	Malignant neoplasm of retrocaecal tissue	187804005
288744014	Malignant neoplasm of retroperitoneum NOS	363420003
288747019	Malignant neoplasm of specified parts of peritoneum	187808008
288748012	Malignant neoplasm of mesocolon	187809000
288749016	Malignant neoplasm of mesocaecum	187810005
288751017	Malignant neoplasm of mesorectum	187811009
288754013	Malignant neoplasm of the pouch of Douglas	187814001
288756010	Malignant neoplasm of specified parts of peritoneum NOS	187808008
288757018	Malignant tumour of peritoneum and retroperitoneum	187801002
288759015	Malignant tumour of intestine	363508008
288760013	Malignant neoplasm of spleen NEC	363499005
288761012	Angiosarcoma of spleen	187821001
288762017	Fibrosarcoma of spleen	187822008
288763010	Malignant tumour of spleen	363499005
288764016	Malignant neoplasm, overlapping lesion of digestive system	187824009
288766019	Malignant neoplasm of digestive tract and peritoneum NOS	255077007
288771014	Malignant neoplasm of cartilage of nose	187829004

288772019	Malignant neoplasm of nasal conchae	187830009
288775017	Malignant neoplasm of vestibule of nose	187831008
288776016	Malignant neoplasm of nasal cavities NOS	363422006
288779011	Malignant neoplasm of auditory (Eustachian) tube	187834000
288784017	Malignant neoplasm of tympanic cavity	187835004
288787012	Malignant neoplasm of tympanic antrum	187836003
288790018	Malignant neoplasm, overlapping lesion of accessory sinuses	187838002
288792014	Malignant tumour of nasal sinuses	363506007
288794010	Malignant neoplasm of glottis	187841006
288797015	Malignant neoplasm of supraglottis	187842004
288799017	Malignant neoplasm of arytenoid cartilage	187843009
288800018	Malignant neoplasm of cricoid cartilage	187844003
288801019	Malignant neoplasm of cuneiform cartilage	187845002
288802014	Malignant neoplasm of thyroid cartilage	187846001
288803016	Malignant tumour of laryngeal cartilage	363431006
288806012	Malignant neoplasm of larynx, other specified site	363429002
288807015	Malignant neoplasm of larynx NOS	363429002
288808013	Malignant neoplasm of lower respiratory tract	430621000
288809017	Malignant neoplasm of cartilage of trachea	187853005
288810010	Malignant neoplasm of mucosa of trachea	187854004
288811014	Malignant neoplasm of trachea NOS	363432004
288813012	Malignant neoplasm of carina of bronchus	187857006
288815017	Malignant neoplasm of main bronchus NOS	372065009
288819011	Malignant neoplasm of upper lobe bronchus	187861000
288820017	Malignant neoplasm of upper lobe of lung	187862007
288821018	Malignant neoplasm of upper lobe, bronchus or lung NOS	269464000
288822013	Malignant neoplasm of middle lobe, bronchus or lung	187864008
288823015	Malignant neoplasm of middle lobe bronchus	187865009
288824014	Malignant neoplasm of middle lobe of lung	187866005
288825010	Malignant neoplasm of middle lobe, bronchus or lung NOS	187864008
288826011	Malignant neoplasm of lower lobe, bronchus or lung	187868006
288827019	Malignant neoplasm of lower lobe bronchus	187869003
288828012	Malignant neoplasm of lower lobe of lung	187870002
288829016	Malignant neoplasm of lower lobe, bronchus or lung NOS	187868006
288832018	Malignant neoplasm of other sites of bronchus or lung	363358000
288839010	Malignant neoplasm of other specified pleura	363433009
288840012	Malignant tumour of pleura	363433009
288841011	Malignant neoplasm of thymus, heart and mediastinum	187881004
288847010	Malignant tumour of heart	363435002
288852017	Malignant neoplasm of heart, thymus and mediastinum NOS	187881004
288857011	Malignant neoplasm of other site of respiratory tract	93986008
288858018	Malignant neoplasm of respiratory system	449096009
288869015	Malignant neoplasm of bones of skull and face	187900002
288872010	Malignant neoplasm of malar bone	187903000
288875012	Malignant neoplasm of orbital bone	187906008
288883018	Malignant neoplasm of bones of skull and face NOS	187900002
288885013	Malignant neoplasm of cervical vertebra	187916000
288886014	Malignant neoplasm of thoracic vertebra	187917009
288887017	Malignant neoplasm of lumbar vertebra	187918004
288888010	Malignant neoplasm of vertebral column NOS	363438000
288889019	Malignant neoplasm of ribs, sternum and clavicle	187920001
288894019	Malignant neoplasm of costal cartilage	187925006
288896017	Malignant neoplasm of xiphoid process	187927003
288897014	Malignant neoplasm of rib, sternum and clavicle NOS	187920001
288898016	Malignant neoplasm of scapula and long bones of upper arm	187929000
288901015	Malignant neoplasm of humerus	187932002
288909018	Malignant neoplasm of carpal bone - scaphoid	187937008
288910011	Malignant neoplasm of carpal bone - lunate	187938003
288911010	Malignant neoplasm of carpal bone - triquetrum	187939006
288912015	Malignant neoplasm of carpal bone - pisiform	187940008
288913013	Malignant neoplasm of carpal bone - trapezium	187941007
288914019	Malignant neoplasm of carpal bone - trapezoid	187942000
288915018	Malignant neoplasm of carpal bone - capitate	187943005
288916017	Malignant neoplasm of carpal bone - hamate	187944004
288917014	Malignant neoplasm of first metacarpal bone	187945003
288918016	Malignant neoplasm of second metacarpal bone	187946002
288919012	Malignant neoplasm of third metacarpal bone	187947006
288920018	Malignant neoplasm of fourth metacarpal bone	187948001
288921019	Malignant neoplasm of fifth metacarpal bone	187949009
288922014	Malignant neoplasm of phalanges of hand	187950009
288923016	Malignant neoplasm of hand bones NOS	269467007

288924010	Malignant neoplasm of pelvic bones, sacrum and coccyx	187952001
288928013	Malignant neoplasm of sacral vertebra	187956003
288929017	Malignant neoplasm of coccygeal vertebra	187957007
288930010	Malignant neoplasm of pelvis, sacrum or coccyx NOS	187952001
288931014	Malignant neoplasm of long bones of leg	449627008
288935017	Malignant neoplasm of long bone of lower limb	449627008
288941012	Malignant neoplasm of calcaneum	187967002
288942017	Malignant neoplasm of medial cuneiform	187968007
288943010	Malignant neoplasm of intermediate cuneiform	187969004
288944016	Malignant neoplasm of lateral cuneiform	187970003
288946019	Malignant neoplasm of navicular	187972006
288947011	Malignant neoplasm of first metatarsal bone	187973001
288948018	Malignant neoplasm of second metatarsal bone	187974007
288949014	Malignant neoplasm of third metatarsal bone	187975008
288950014	Malignant neoplasm of fourth metatarsal bone	187976009
288951013	Malignant neoplasm of fifth metatarsal bone	187977000
288952018	Malignant neoplasm of phalanges of foot	187978005
288953011	Malignant neoplasm of short bones of leg NOS	712525007
288959010	Malignant neoplasm of bone and articular cartilage	1090821000000101
288960017	Malignant neoplasm of connective and other soft tissue	269469005
288962013	Malignant neoplasm of cartilage of ear	187987001
288963015	Malignant neoplasm of tarsus of eyelid	187988006
288964014	Malignant neoplasm soft tissues of cervical spine	187989003
288967019	Malignant neoplasm of connective and soft tissue of shoulder	187992004
288968012	Malignant neoplasm of connective and soft tissue, upper arm	187993009
288969016	Malignant neoplasm of connective and soft tissue of fore-arm	187994003
288970015	Malignant neoplasm of connective and soft tissue of hand	187995002
288971016	Malignant neoplasm of connective and soft tissue of finger	187996001
288972011	Malignant neoplasm of connective and soft tissue of thumb	187997005
288975013	Malignant neoplasm of connective and soft tissue of hip	188000002
288979019	Malignant neoplasm of connective and soft tissue of foot	188004006
288980016	Malignant neoplasm of connective and soft tissue of toe	188005007
288985014	Malignant neoplasm of connective and soft tissue of thorax	188009001
288986010	Malignant neoplasm of connective and soft tissue of axilla	188010006
288991011	Malignant neoplasm of connective and soft tissue of abdomen	188015001
288995019	Malignant neoplasm of connective and soft tissue of pelvis	188019007
288999013	Malignant neoplasm of connective and soft tissue of buttock	188020001
289001014	Malignant neoplasm of connective and soft tissue of perineum	188022009
289007013	Malignant neoplasm of connective and soft tissue, site NOS	269469005
289008015	Kaposi's sarcoma of soft tissue	188029000
289009011	Malignant melanoma of lip	188030005
289011019	Malignant melanoma of ear and external auricular canal	188032002
289012014	Malignant melanoma of auricle (ear)	188033007
289013016	Malignant melanoma of external auditory meatus	188034001
289014010	Malignant melanoma of ear and external auricular canal NOS	188032002
289015011	Malignant melanoma of skin of face	93225001
289017015	Malignant melanoma of chin	188038003
289019017	Malignant melanoma of forehead	188040008
289021010	Malignant melanoma of temple	188042000
289022015	Malignant melanoma of skin of face	93225001
289023013	Malignant melanoma of scalp and neck	188044004
289024019	Malignant melanoma of scalp	188045003
289025018	Malignant melanoma of neck	188046002
289026017	Malignant melanoma of scalp and/or neck	188044004
289027014	Malignant melanoma of skin of trunk	93651008
289028016	Malignant melanoma of axilla	188049009
289029012	Malignant melanoma of breast	188050009
289030019	Malignant melanoma of buttock	188051008
289031015	Malignant melanoma of groin	188052001
289033017	Malignant melanoma of perineum	188054000
289034011	Malignant melanoma of umbilicus	188055004
289037016	Malignant melanoma of trunk	269579005
289039018	Malignant melanoma of shoulder	188060000
289040016	Malignant melanoma of upper arm	188061001
289041017	Malignant melanoma of fore-arm	188062008
289042012	Malignant melanoma of hand	188063003
289043019	Malignant melanoma of finger	188064009
289044013	Malignant melanoma of thumb	188065005
289045014	Malignant melanoma of upper limb or shoulder NOS	269580008
289046010	Malignant melanoma of lower limb and hip	188067002
289047018	Malignant melanoma of hip	188068007

289048011	Malignant melanoma of thigh	188069004
289049015	Malignant melanoma of knee	188070003
289050015	Malignant melanoma of popliteal fossa area	188071004
289051016	Malignant melanoma of lower leg	188072006
289052011	Malignant melanoma of ankle	188073001
289053018	Malignant melanoma of heel	188074007
289054012	Malignant melanoma of foot	188075008
289055013	Malignant melanoma of toe	188076009
289056014	Malignant melanoma of great toe	188077000
289057017	Malignant melanoma of lower limb or hip NOS	188067002
289060012	Malignant melanoma of other specified skin site	93655004
289063014	Malignant melanoma of skin NOS	93655004
289066018	Malignant neoplasm of sebaceous gland	94000008
289079010	Malignant neoplasm of skin of auricle (ear)	188090007
289080013	Malignant neoplasm of skin of external auditory meatus	188091006
289084016	Malignant neoplasm of skin of cheek, external	188095002
289088018	Malignant neoplasm of skin of nose (external)	188099008
289089014	Malignant neoplasm of skin of temple	188100000
289091018	Malignant neoplasm of scalp and skin of neck	188102008
289092013	Malignant neoplasm of scalp	188103003
289094014	Malignant neoplasm of scalp and/or skin of neck	188102008
289095010	Malignant neoplasm of skin of trunk	372126009
289096011	Malignant neoplasm of skin of axillary fold	188107002
289097019	Malignant neoplasm of skin of chest, excluding breast	372126009
289099016	Malignant neoplasm of skin of abdominal wall	188110009
289107010	Malignant neoplasm of skin of trunk, excluding scrotum, NOS	372126009
289108017	Malignant neoplasm of skin of upper limb and shoulder	188119005
289110015	Malignant neoplasm of skin of upper arm	188121000
289111016	Malignant neoplasm of skin of fore-arm	188122007
289114012	Malignant neoplasm of skin of thumb	188125009
289115013	Malignant neoplasm of skin of upper limb or shoulder NOS	188119005
289116014	Malignant neoplasm of skin of lower limb and hip	188127001
289121012	Malignant neoplasm of skin of popliteal fossa area	188132000
289122017	Malignant neoplasm of skin of lower leg	188133005
289124016	Malignant neoplasm of skin of heel	188135003
289127011	Malignant neoplasm of skin of great toe	188138001
289128018	Malignant neoplasm of skin of lower limb or hip NOS	188127001
289131017	Malignant neoplasm of other specified skin sites	372130007
289132012	Malignant neoplasm of skin NOS	372130007
289137018	Malignant neoplasm of nipple and areola of female breast	188147009
289140018	Malignant neoplasm of nipple or areola of female breast NOS	188147009
289141019	Malignant neoplasm of central part of female breast	188151006
289142014	Malignant neoplasm of upper-inner quadrant of female breast	188152004
289143016	Malignant neoplasm of lower-inner quadrant of female breast	188153009
289144010	Malignant neoplasm of upper-outer quadrant of female breast	188154003
289145011	Malignant neoplasm of lower-outer quadrant of female breast	188155002
289146012	Malignant neoplasm of axillary tail of female breast	188156001
289147015	Malignant neoplasm, overlapping lesion of breast	188157005
289148013	Malignant neoplasm of other site of female breast	372064008
289149017	Malignant neoplasm of ectopic site of female breast	188159008
289150017	Malignant neoplasm of other site of female breast NOS	372064008
289151018	Malignant neoplasm of female breast NOS	372064008
289153015	Malignant neoplasm of nipple and areola of male breast	188163001
289156011	Malignant neoplasm of nipple or areola of male breast NOS	188163001
289157019	Malignant neoplasm of other site of male breast	372095001
289158012	Malignant neoplasm of ectopic site of male breast	188168005
289159016	Malignant neoplasm of male breast NOS	372095001
289166015	Malignant neoplasm of uterus	371973000
289171010	Malignant neoplasm of endocervical canal	188176007
289172015	Malignant neoplasm of endocervical gland	188177003
289173013	Malignant neoplasm of endocervix NOS	372097009
289175018	Malignant neoplasm, overlapping lesion of cervix uteri	188180002
289178016	Malignant neoplasm of other site of cervix	363354003
289179012	Malignant neoplasm of cervical stump	188183000
289180010	Malignant neoplasm of squamocolumnar junction of cervix	188184006
289181014	Malignant neoplasm of other site of cervix NOS	363354003
289182019	Malignant neoplasm of cervix uteri NOS	363354003
289184018	Choriocarcinoma	188188009
289187013	Malignant neoplasm of corpus uteri, excluding isthmus	188189001
289188015	Malignant neoplasm of cornu of corpus uteri	188190005
289189011	Malignant neoplasm of fundus of corpus uteri	188191009

289190019	Malignant neoplasm of endometrium of corpus uteri	188192002
289191015	Malignant neoplasm of myometrium of corpus uteri	188193007
289192010	Malignant neoplasm of corpus uteri NOS	188189001
289193017	Malignant neoplasm of isthmus of uterine body	188195000
289194011	Malignant neoplasm of lower uterine segment	188195000
289195012	Malignant neoplasm of isthmus of uterine body NOS	188195000
289196013	Malignant neoplasm of overlapping lesion of corpus uteri	188198003
289197016	Malignant neoplasm of other site of uterine body	371972005
289198014	Malignant neoplasm of body of uterus NOS	371972005
289199018	Malignant neoplasm of uterine adnexa	428322007
289202011	Malignant neoplasm of round ligament	188204000
289203018	Malignant neoplasm of other site of uterine adnexa	428322007
289204012	Malignant neoplasm of uterine adnexa NOS	428322007
289206014	Malignant neoplasm of Gartner's duct	188208002
289207017	Malignant neoplasm of vaginal vault	188209005
289208010	Malignant neoplasm of vagina NOS	363445000
289209019	Malignant neoplasm of greater vestibular (Bartholin's) gland	188211001
289210012	Malignant neoplasm of labia majora NOS	363446004
289217010	Malignant neoplasm of other specified female genital organ	363514001
289219013	Malignant tumour of female genital organ	363514001
289223017	Malignant neoplasm of undescended testis	188219004
289224011	Malignant neoplasm of ectopic testis	188220005
289234019	Malignant neoplasm of undescended testis NOS	188219004
289242018	Malignant neoplasm of penis and other male genital organs	363515000
289246015	Malignant neoplasm of body of penis	188230001
289249010	Malignant tumour of penis	363516004
289251014	Malignant neoplasm of other male genital organ	363515000
289252019	Malignant neoplasm of seminal vesicle	188234005
289257013	Malignant neoplasm of tunica vaginalis	188235006
289259011	Malignant neoplasm of other male genital organ NOS	363515000
289260018	Malignant neoplasm of penis and other male genital organ NOS	363515000
289262014	Malignant neoplasm of trigone of urinary bladder	188239000
289267015	Malignant neoplasm of dome of urinary bladder	188240003
289270016	Malignant neoplasm of lateral wall of urinary bladder	188241004
289271017	Malignant neoplasm of anterior wall of urinary bladder	188242006
289272012	Malignant neoplasm of posterior wall of urinary bladder	188243001
289275014	Malignant neoplasm of bladder neck	188244007
289276010	Malignant neoplasm of ureteric orifice	188245008
289279015	Malignant neoplasm of other site of urinary bladder	399326009
289280017	Malignant neoplasm, overlapping lesion of bladder	188247000
289281018	Malignant neoplasm of urinary bladder NOS	399326009
289285010	Malignant neoplasm of kidney parenchyma	188250002
289288012	Hypernephroma	702391001
289294016	Malignant neoplasm of ureteropelvic junction	188253000
289296019	Malignant neoplasm of renal pelvis NOS	363457009
289297011	Malignant neoplasm of other urinary organs	419052002
289298018	Malignant neoplasm of overlapping lesion of urinary organs	188256008
289299014	Malignant tumour of urinary system	419052002
289301019	Malignant neoplasm of genitourinary organ NOS	271468000
289308013	Malignant neoplasm of ciliary body	188263008
289311014	Malignant neoplasm of iris	188264002
289314018	Malignant neoplasm of crystalline lens	188265001
289315017	Malignant neoplasm of sclera	188266000
289316016	Malignant neoplasm of eyeball NOS	188261005
289317013	Malignant neoplasm of connective tissue of orbit	188268004
289318015	Malignant neoplasm of extraocular muscle of orbit	188269007
289319011	Malignant neoplasm of orbit NOS	363462005
289322013	Malignant neoplasm of lacrimal gland	188272000
289325010	Malignant neoplasm of lacrimal sac	188273005
289326011	Malignant neoplasm of nasolacrimal duct	188274004
289327019	Malignant neoplasm of lacrimal duct NOS	280959007
289329016	Malignant neoplasm of other specified site of eye	363461003
289330014	Malignant neoplasm of eye NOS	363461003
289337012	Malignant neoplasm of basal ganglia	188281006
289338019	Malignant neoplasm of cerebral cortex	188282004
289339010	Malignant neoplasm of corpus striatum	188283009
289341011	Malignant neoplasm of globus pallidus	188285002
289343014	Malignant neoplasm of hypothalamus	188286001
289345019	Malignant neoplasm of thalamus	188287005
289346018	Malignant neoplasm of cerebrum NOS	188280007
289347010	Malignant neoplasm of hippocampus	188289008

289348017	Malignant neoplasm of uncus	188290004
289349013	Malignant neoplasm of temporal lobe NOS	363468009
289352017	Malignant neoplasm of choroid plexus	188292007
289353010	Malignant neoplasm of floor of cerebral ventricle	188293002
289354016	Malignant neoplasm of cerebral ventricle NOS	363471001
289355015	Malignant neoplasm of cerebral peduncle	188295009
289356019	Malignant neoplasm of medulla oblongata	188296005
289357011	Malignant neoplasm of midbrain	188297001
289358018	Malignant neoplasm of pons	188298006
289360016	Malignant neoplasm of other parts of brain	428061005
289361017	Malignant neoplasm of corpus callosum	188301005
289362012	Malignant neoplasm of tapetum	188302003
289364013	Malignant neoplasm of other part of brain NOS	428061005
289365014	Malignant neoplasm of brain NOS	428061005
289369015	Malignant neoplasm of olfactory bulb	188308004
289374011	Malignant neoplasm of cranial nerves NOS	188307009
289375012	Malignant neoplasm of cerebral dura mater	188312005
289376013	Malignant neoplasm of cerebral arachnoid mater	188313000
289378014	Malignant neoplasm of cerebral pia mater	188315007
289379018	Malignant neoplasm of cerebral meninges NOS	363474009
289380015	Malignant neoplasm of spinal dura mater	188317004
289381016	Malignant neoplasm of spinal arachnoid mater	188318009
289382011	Malignant neoplasm of spinal pia mater	188319001
289383018	Malignant neoplasm of spinal meninges NOS	363497007
289388010	Malignant neoplasm of peripheral nerve of thorax	188325002
289389019	Malignant neoplasm of peripheral nerve of abdomen	188326001
289390011	Malignant neoplasm of peripheral nerve of pelvis	188327005
289396017	Malignant neoplasm of other specified part of nervous system	372063002
289397014	Malignant neoplasm of nervous system	372063002
289406011	Malignant tumour of adrenal gland	363355002
289409016	Malignant neoplasm of craniopharyngeal duct	188340000
289413011	Primary malignant neoplasm of paraganglion	93941005
289419010	Malignant neoplasm of aortic body or paraganglia NOS	93941005
289421017	Malignant neoplasm of other specified endocrine gland	371982006
289423019	Malignant neoplasm of other and ill-defined sites	302817000
289424013	Malignant neoplasm of head, neck and face	188353002
289425014	Malignant tumour of head and neck	255056009
289426010	Carcinoma of cheek	447949005
289427018	Malignant neoplasm of nose	1090251000000105
289428011	Malignant neoplasm of jaw	1090921000000106
289429015	Malignant tumour of neck	363489000
289430013	Malignant neoplasm of supraclavicular fossa	1090951000000101
289431012	Malignant neoplasm of head, neck and face NOS	188353002
289432017	Malignant neoplasm of thorax	188361007
289435015	Malignant tumour of axilla	363502009
289436019	Malignant neoplasm of chest wall	712750007
289437011	Malignant neoplasm of intrathoracic site NOS	428100006
289438018	Malignant neoplasm of thorax NOS	188361007
289441010	Malignant neoplasm of abdomen	188366002
289442015	Malignant neoplasm of inguinal region	1090941000000104
289445018	Malignant neoplasm of pelvis NOS	363484005
289446017	Malignant neoplasm of upper limb NOS	363503004
289447014	Malignant tumour of lower limb	363504005
289448016	Malignant neoplasm of other specified sites	363346000
289449012	Malignant neoplasm of back	1090321000000107
289450012	Malignant neoplasm of trunk	1090311000000101
289451011	Malignant neoplasm of flank	1090301000000103
289452016	Malignant neoplasm of specified site NOS	363346000
289515018	Secondary malignant neoplasm of respiratory tract	94515004
289530010	Secondary malignant neoplasm of gastrointestinal tract	94313005
289535017	Secondary malignant neoplasm of other urinary organs	94663008
289536016	Secondary malignant neoplasm of other urinary organ NOS	94663008
289538015	Secondary malignant neoplasm of skin of head	188454009
289542017	Secondary malignant neoplasm of skin of shoulder and arm	188458007
289543010	Secondary malignant neoplasm of skin of hip and leg	188459004
289545015	Secondary malignant neoplasm of skin NOS	94579000
289546019	Secondary malignant neoplasm of brain and spinal cord	188462001
289549014	Secondary malignant neoplasm of brain or spinal cord NOS	188462001
289550014	Secondary malignant neoplasm of other part of nervous system	94442001
289553011	Secondary malignant neoplasm of cervix uteri	188469005
289554017	Secondary cancer of the cervix	188469005

289557012	Secondary malignant neoplasm of epididymis and vas deferens	188471005
289559010	Secondary malignant neoplasm of other specified site NOS	128462008
289561018	Malignant neoplasm of unspecified site	302817000
289565010	Other malignant neoplasm NOS	363346000
289566011	Malignant neoplasms of independent (primary) multiple sites	188478004
289571016	Malignant neoplasm of unspecified site NOS	363346000
289574012	Malignant neoplasm of other and unspecified site NOS	363346000
289580016	Lymphosarcoma and reticulosarcoma	188487008
289581017	Reticulosarcoma of unspecified site	373168002
289582012	Reticulosarcoma of lymph nodes of head, face and neck	188489006
289590012	Reticulosarcoma NOS	373168002
289591011	Lymphosarcoma	188498009
289592016	Lymphosarcoma of unspecified site	188498009
289593014	Lymphosarcoma of lymph nodes of head, face and neck	188500005
289594015	Lymphosarcoma of intrathoracic lymph nodes	188501009
289595019	Lymphosarcoma of intra-abdominal lymph nodes	188502002
289599013	Lymphosarcoma of spleen	188506004
289600011	Lymphosarcoma of lymph nodes of multiple sites	188507008
289601010	Lymphosarcoma NOS	188498009
289603013	Burkitt's lymphoma of lymph nodes of head, face and neck	188510001
289604019	Burkitt's lymphoma of intrathoracic lymph nodes	188511002
289605018	Burkitt's lymphoma of intra-abdominal lymph nodes	188512009
289612010	Burkitt's lymphoma NOS	118617000
289613017	Other specified reticulosarcoma or lymphosarcoma	188487008
289614011	Reticulosarcoma or lymphosarcoma NOS	188487008
289621011	Hodgkin's paragranuloma of intra-abdominal lymph nodes	93493001
289629013	Hodgkin's granuloma of lymph nodes of head, face and neck	188534006
289643015	Hodgkin's sarcoma of lymph nodes of axilla and upper limb	188547001
289659015	Hodgkin's, lymphocytic-histiocytic predominance NOS	118607005
289660013	Hodgkin's disease, nodular sclerosis of unspecified site	118608000
289669014	Hodgkin's disease, nodular sclerosis NOS	118608000
289670010	Hodgkin's disease, mixed cellularity of unspecified site	118609008
289679011	Hodgkin's disease, mixed cellularity NOS	118609008
289680014	Hodgkin's lymphocytic depletion of unspecified site	118610003
289699017	Hodgkin's disease, lymphocytic depletion of spleen	188592008
289701017	Hodgkin's disease, lymphocytic depletion NOS	118610003
289702012	Hodgkin's disease NOS	118599009
289703019	Hodgkin's disease NOS, unspecified site	118599009
289713010	Other malignant neoplasm of lymphoid and histiocytic tissue	277606000
289717011	Nodular lymphoma of unspecified site	269476000
289718018	Nodular lymphoma of lymph nodes of head, face and neck	188609000
289721016	Nodular lymphoma of lymph nodes of axilla and upper limb	188612002
289726014	Nodular lymphoma NOS	269476000
289727017	Mycosis fungoides of unspecified site	118618005
289738017	Mycosis fungoides of lymph nodes of multiple sites	188627002
289739013	Mycosis fungoides NOS	118618005
289749011	Sezary's disease NOS	118611004
289773015	Leukaemic reticuloendotheliosis NOS	118613001
289776011	Letterer-Siwe disease of unspecified sites	118614007
289780018	Letterer-Siwe disease NOS	118614007
289791013	Mast cell malignancy of lymph nodes of multiple sites	188669003
289799010	Diffuse non-Hodgkin's small cell (diffuse) lymphoma	188674006
289801013	Diffuse non-Hodgkin's small cleaved cell (diffuse) lymphoma	188675007
289812013	Diffuse non-Hodgkin's lymphoma undifferentiated (diffuse)	188679001
289813015	Other types of follicular non-Hodgkin's lymphoma	308121000
289820010	Malignant lymphoma (clinical)	118600007
289829011	Malignant lymphoma NOS	118600007
289830018	Malignant lymphoma NOS of unspecified site	118600007
289831019	Malignant lymphoma of lymph nodes of head, face AND/OR neck	93195001
289832014	Malignant lymphoma of intrathoracic lymph nodes	93193008
289842011	Malignant lymphoma of intra-abdominal lymph nodes	93191005
289845013	Malignant lymphoma of intrapelvic lymph nodes	93192003
289846014	Malignant lymphoma of spleen	93198004
289847017	Malignant lymphoma of lymph nodes of multiple sites	93197009
289849019	Malignant neoplasms of lymphoid and histiocytic tissue NOS	118600007
289867014	Malignant plasma cell neoplasm, extramedullary plasmacytoma	188718006
289875015	Lymphatic leukaemia	188725004
289876019	Lymphoid leukaemia	188725004
289880012	Subacute lymphoid leukaemia	188726003
289881011	Other lymphoid leukaemia	188725004
289885019	Adult T-cell leukaemia	188729005

289888017	Other lymphoid leukaemia NOS	188725004
289889013	Lymphoid leukaemia NOS	188725004
289892012	Myeloid leukaemia	188732008
289895014	Chronic eosinophilic leukaemia	188733003
289898011	Chronic neutrophilic leukaemia	188734009
289900013	Chronic myeloid leukaemia NOS	92818009
289901012	Subacute myeloid leukaemia	188736006
289903010	Chloroma	188737002
289904016	Granulocytic sarcoma	188738007
289911017	Other myeloid leukaemia NOS	188732008
289912012	Myeloid leukaemia NOS	188732008
289917018	Monocytic leukaemia	188744006
289919015	Monoblastic leukaemia	188744006
289921013	Histiocytic leukaemia	188744006
289923011	Chronic monocytic leukaemia	188745007
289924017	Subacute monocytic leukaemia	188746008
289926015	Other monocytic leukaemia	188744006
289930017	Other monocytic leukaemia NOS	188744006
289933015	Monocytic leukaemia NOS	188744006
289934014	Other specified leukaemia	93143009
289948014	Thrombocytic leukaemia	188754005
289949018	Megakaryocytic leukaemia	188754005
289955011	Other and unspecified leukaemia	93143009
289959017	Other and unspecified leukaemia NOS	93143009
289960010	Other specified leukaemia NOS	93143009
289963012	Leukaemia of unspecified cell type	93143009
289971011	Other leukaemia of unspecified cell type	93143009
289974015	Myelomonocytic leukaemia	188768003
289980011	Subacute myelomonocytic leukaemia	188770007
289987014	Malignant neoplasm lymphatic or haematopoietic tissue NOS	269475001
291329017	Carcinoma, undifferentiated	38549000
291330010	Carcinoma, anaplastic	58248003
291343010	Papillary carcinoma	25910003
291344016	Verrucous carcinoma	89906000
291364014	[M]Transitional cell papilloma or carcinoma NOS	118287003
291375013	[M]Pancreatic adenomas and carcinomas	126859007
291378010	[M]Gastrinoma and carcinomas	16189002
291379019	[M]Gastrinoma or carcinoma NOS	16189002
291380016	[M]Hepatobiliary tract adenomas and carcinomas	126853008
291403016	[M]Solid carcinoma NOS	81920005
291414018	[M]Carcinoid tumour, malignant	189607006
291419011	[M]Carcinoid tumour, nonargentaffin, malignant	189607006
291438019	[M]Pituitary adenomas and carcinomas	128665000
291445019	[M]Renal adenoma and carcinoma	115215004
291462012	[M]Endometrioid adenomas and carcinomas	123844007
291472010	[M]Skin appendage adenoma and carcinoma	126488004
291493013	[M]Cystadenoma and carcinoma	408871000000109
291528014	[M]Signet ring carcinoma NOS	87737001
291536017	[M]Comedocarcinoma NOS	722237009
291538016	Medullary carcinoma	32913002
291540014	Lobular carcinoma	89740008
291638011	[M]Liposarcoma NOS	49430005
291669010	[M]Mixed tumour, malignant, NOS	8145008
291674019	[M]Mesenchymal nephroblastoma	25081006
291700017	[M]Clear cell sarcoma of tendons and aponeuroses	12622007
291711013	[M]Mesothelioma, unspecified	115232000
291715016	[M]Seminoma NOS	443677002
291782010	[M]Chondrosarcoma NOS	14990007
291824019	[M]Choroid plexus papilloma, malignant	88252006
291828016	[M]Spongioblastoma NOS	63634009
291843019	[M]Neuroepitheliomatous neoplasms	115241005
291854012	[M]Retinoblastomas	19906005
291887011	[M]Lymphomas, NOS or diffuse	414628006
291891018	[M]Malignant lymphoma, diffuse NOS	115244002
291892013	[M]Malignant lymphoma, undifferentiated cell type NOS	115244002
291894014	[M]Malignant lymphoma, convoluted cell type NOS	115244002
291900014	[M]Malignant lymphoma, centroblastic-centrocytic, diffuse	115244002
291902018	[M]Malignant lymphoma, follicular centre cell NOS	115244002
291903011	[M]Malignant lymphoma, lymphocytic, well differentiated NOS	115244002
291905016	[M]Malignant lymphoma, centrocytic	115244002
291907012	[M]Malignant lymphoma, follicular centre cell, cleaved NOS	115244002

291909010	[M]Prolymphocytic lymphosarcoma	115244002
291910017	[M]Malignant lymphoma, centroblastic type NOS	115244002
291913015	[M]Malignant lymphomatous polyposis	74654000
291914014	[M]Malignant lymphoma, large cell, cleaved, diffuse	115244002
291923012	[M]Reticulosarcoma NOS	189982000
291931019	[M]Hodgkin's disease, lymphocytic predominance	128799007
291943018	[M]Malignant lymphoma, centroblastic-centrocytic, follicular	115244002
291953017	[M]Lymphoma, nodular or follicular NOS	414628006
291955012	[M]Mycosis fungoides NOS	90120004
291957016	[M]Microglioma	21964009
291958014	[M]Histiocytic medullary reticulosis	128920006
291960011	[M] Alpha heavy chain disease	6381009
291962015	[M] Gamma heavy chain disease	6381009
291984015	[M]Leukaemias unspecified	128931003
291985019	[M]Leukaemia NOS	128931003
291988017	[M]Subacute leukaemia NOS	128931003
291992012	[M]Aleukaemic leukaemia NOS	128931003
291995014	[M]Leukaemia unspecified, NOS	128931003
292005016	[M]Lymphoid leukaemias	128935007
292014014	[M]Plasma cell leukaemias	128922003
292017019	Plasma cell leukaemia	128922003
292024018	[M]Erythroleukaemia NOS	14317002
292032014	[M]Myeloid leukaemias	128934006
292035011	[M]Subacute myeloid leukaemia	128934006
292041016	[M]Other myeloid leukaemia NOS	128934006
292046014	[M]Eosinophilic leukaemias	190055003
292062015	Monocytic leukaemia	37810007
292065018	[M]Miscellaneous leukaemias	128931003
292073010	[M]Acute megakaryoblastic leukaemia	52220008
292075015	[M]Acute myelofibrosis	80570006
292077011	[M]Miscellaneous leukaemia NOS	128931003
292083014	[M]Monocytoid B-cell lymphoma	128803008
292099015	Malignant tumour of digestive organ	255077007
292101010	Liver carcinoma	109841003
292102015	[X]Malignant neoplasm of intestinal tract, part unspecified	363508008
292105018	Malignant neoplasm of bronchus or lung	1090881000000100
292110019	Malignant tumour of mediastinum	363494000
292111015	Malignant neoplasm of skeletal system	443679004
292116013	[X]Melanoma and other malignant neoplasms of skin	372130007
292118014	[X]Malignant melanoma of skin, unspecified	93655004
292120012	[X]Malignant neoplasm of skin, unspecified	372130007
292121011	[X]Malignant neoplasm of mesothelial and soft tissue	387837005
292126018	Cancer, mesothelioma	109378008
292128017	[X]Kaposi's sarcoma, unspecified	109385007
292132011	[X]Malignant neoplasm of peritoneum, unspecified	363492001
292135013	Overlapping malignant neoplasm of skin	109264009
292137017	Malignant tumour of breast	254837009
292138010	[X]Malignant neoplasm of female genital organs	363514001
292139019	[X]Malignant neoplasm of uterine adnexa, unspecified	428322007
292142013	[X]Malignant neoplasm of female genital organ, unspecified	363514001
292143015	Malignant tumour of male genital organ	363515000
292146011	[X]Malignant neoplasm of male genital organ, unspecified	363515000
292147019	[X]Malignant neoplasm of urinary tract	419052002
292148012	Malignant neoplasm of urinary organ	448233000
292152012	Malignant tumour of meninges	363497007
292154013	Malignant neoplasm of endocrine gland	371982006
292156010	Malignant neoplasm of endocrine gland	371982006
292158011	[X]Malignant neoplasm of other specified sites	363346000
292165015	[X]Secondary malignant neoplasm of other specified sites	128462008
292166019	[X]Malignant neoplasm without specification of site	302817000
292169014	[X]Other Hodgkin's disease	118599009
292170010	[X]Other types of follicular non-Hodgkin's lymphoma	308121000
292173012	[X]Other types of diffuse non-Hodgkin's lymphoma	109962001
292174018	[X]Other specified types of non-Hodgkin's lymphoma	118601006
292177013	[X]Other lymphoid leukaemia	188725004
292178015	[X]Other myeloid leukaemia	188732008
292181013	[X]Other monocytic leukaemia	188744006
292182018	[X]Other specified leukaemias	93143009
292185016	[X]Other leukaemia of unspecified cell type	93143009
292193016	[X]Unspecified B-cell non-Hodgkin's lymphoma	1091921000000103
293380012	Waldenstrom's macroglobulinaemia	190818004

297584015	Myasthenic syndrome due to other malignancy	193209005
312137011	Osteoporosis in multiple myelomatosis	739301006
313874017	Meckel's diverticulum NOS	37373007
342807011	Hepatocellular carcinoma	109841003
342834014	Hepatoblastoma of liver	109843000
342851019	Primary angiosarcoma of liver	109844006
344657018	T-zone lymphoma	109975001
344670010	Lymphoepithelioid lymphoma	109976000
344806015	True histiocytic lymphoma	109988003
344818012	Kahler's disease	109989006
344819016	Myelomatosis	109989006
344820010	Multiple myeloma	109989006
344846014	Acute myelofibrosis	109991003
353772013	Malignant ascites	236005001
376870016	[M]Fibroxanthosarcoma	115225009
378691015	FIGO staging of gynaecological malignancy	254383006
379088010	Carcinoma of rectum	254582000
379089019	Rectal carcinoma	254582000
379246013	Multiple self-healing epithelioma of Ferguson-Smith	254659009
379315011	Rodent ulcer	254701007
380059013	Malignant tumour of unknown origin	255052006
380092019	Carcinoma of genitourinary organ	255066001
380093012	Sarcoma of bone and connective tissue	255067005
380094018	Carcinoma of bone, connective tissue, skin and breast	255068000
380095017	Carcinoma of lip, oral cavity and pharynx	255069008
380129018	Carcinoma of caecum	255081007
380137014	Anal carcinoma	363352004
380139012	Carcinoma common bile duct	255086002
380145016	Malignant neoplasm of carpal bones	255090000
380146015	Malignant neoplasm of metacarpal bones	255091001
380180017	Seminoma of testis	255107005
380193010	Secondary carcinoma of respiratory and/or digestive systems	269473008
380206018	Secondary carcinoma of other specified sites	128462008
380211016	Carcinoma of other and unspecified sites	363346000
403673017	Malignant neoplasm of digestive organs and peritoneum	269456006
403674011	Malignant neoplasm of oesophagus NOS	363402007
403682011	Malignant tumour of colon	363406005
403688010	Malignant neoplasm of upper lobe, bronchus or lung	269464000
403689019	Malignant tumour of lung	363358000
403691010	Malignant neoplasm of bone and articular cartilage	443679004
403697014	Malignant neoplasm of hand bones	269467007
403698016	Malignant neoplasm of short bone of lower limb	712525007
403700013	Malignant neoplasm of skin	372130007
403701012	Malignant tumour of vulva	363367000
403702017	Malignant neoplasm of testis NOS	363449006
403705015	Malignant neoplasm of lymphatic and haemopoietic tissue	269475001
403710016	Lymphoid and histiocytic malignancy NOS	269475001
403754010	Malignant melanoma	2092003
403758013	[M]Neuroblastoma NOS	87364003
403759017	[M]Malignant lymphoma, mixed lymphocytic-histiocytic NOS	115244002
403760010	[M]Lymphogranuloma, malignant	128929007
403761014	[M]Malignant lymphoma, nodular NOS	115244002
403762019	Mesothelioma (malignant, clinical disorder)	109378008
403771011	Carcinoma of lip	269515006
406052010	Malignant neoplasm of lip, oral cavity and pharynx	271323007
406263015	Malignant neoplasm of genitourinary organ	271468000
406265010	Malignant neoplastic disease	363346000
406266011	Secondary malignant neoplasm of other specified sites	128462008
406333010	Secondary malignant neoplasm of lymph node	94392001
406399011	Malignant neoplasm of lower lip, inner aspect	271568003
409844012	Malignant melanoma of eye	274087000
409845013	Secondary malignant neoplasm of unknown site	274088005
411399019	Primary vulval cancer	275419009
413089014	Dermatofibrosarcoma protuberans	276799004
414008012	B-cell chronic lymphocytic leukaemia	277473004
414151014	T-cell prolymphocytic leukaemia	277567002
414160018	B-cell acute lymphoblastic leukaemia	277571004
414220013	Acute monoblastic leukaemia	277601005
414248013	B-cell prolymphocytic leukaemia	277619001
414252013	Mucosa-associated lymphoma	277622004
414253015	Maltoma	277622004

414257019	Follicular non-Hodgkin's small cleaved cell lymphoma	277625002
414278016	Follicular non-Hodgkin's large cell lymphoma	277641001
414298014	Enteropathy-associated T-cell lymphoma	277654008
414849013	Pancoast's syndrome	278065000
444799014	Diffuse non-Hodgkin's centroblastic lymphoma	302842009
445587010	Malignant reticulosis	118612006
450901012	Carcinomatosis	307593001
451077018	Anaemia in ovarian carcinoma	307726001
451428019	Malignant neoplasm of epiglottis NOS	187842004
451429010	Follicular non-Hodgkin's lymphoma	308121000
451430017	Plasmacytoma NOS	415112005
453301018	Malignant tumour of lip	363348004
453326010	[M]Hodgkin's disease NOS	128930002
454085011	Malignant melanoma of back	310498001
454091013	Primary malignant neoplasm of unknown site	310504009
454201010	Malignant neoplasm of canthus	310599006
455404011	Malignant neoplasm of pinna NEC	277156006
455405012	Malignant neoplasm of skin of scapular region	311779007
457109011	Malignant melanoma of chest wall	313248004
457110018	Malignant neoplasm of upper eyelid	313249007
457111019	Malignant neoplasm of lower eyelid	313250007
457307018	[M]Tibial adamantinoma	56763007
457317011	Lambda light chain myeloma	313427003
457318018	Seminoma of undescended testis	313428008
457319014	Seminoma of descended testis	313429000
459378019	Local recurrence of malignant tumour of breast	314955001
459399016	Local recurrence of malignant tumour of urinary bladder	314968009
459527013	Hereditary nonpolyposis colon cancer	315058005
474077018	Chronic myelomonocytic leukaemia	127225006
482537012	Malignant neoplasm of mesentery	363370001
482641017	Malignant neoplasm of omentum	363421004
482690017	Malignant neoplasm of labia majora	363446004
482691018	Malignant neoplasm of labia minora	363447008
482730011	Malignant neoplasm of frontal lobe	363467004
482731010	Malignant neoplasm of temporal lobe	363468009
482732015	Malignant neoplasm of parietal lobe	363469001
482733013	Malignant neoplasm of occipital lobe	363470000
482734019	Malignant neoplasm of cerebral ventricles	363471001
482737014	Malignant neoplasm of cerebral meninges	363474009
482740014	Malignant neoplasm of spinal meninges	363476006
482741013	Malignant neoplasm of cauda equina	363477002
509741017	Acute lymphoid leukaemia	91857003
511250010	Secondary malignant neoplasm of breast	94297009
511385013	Secondary cancer of the vulva	94681006
1208876015	Malignant neoplasm of eye	363461003
1210553016	Malignant neoplasm of adrenal cortex	371964008
1210554010	Malignant neoplasm of adrenal medulla	371965009
1210561014	Malignant neoplasm of body of uterus	371972005
1210566016	Malignant neoplasm of clitoris	371979001
1210642019	Malignant neoplasm of female breast	372064008
1211679013	Reticulosarcoma	373168002
1215809015	Secondary malignant neoplasm of retroperitoneum	94628003
1215862010	Malignant neoplasm, overlapping lesion of oesophagus	109835005
1216015014	[M]Prolymphocytic leukaemia	128923008
1216160012	[M]Acute leukaemia NOS	24072005
1216284012	[M]Acute promyelocytic leukaemia	28950004
1216346016	[M]Acute myelomonocytic leukaemia	30962008
1216356017	[M]Mucocarcinoid tumour, malignant	31396002
1216396015	[M]Lymphoid leukaemia NOS	32280000
1216442018	[M]Triton tumour, malignant	354002
1216464010	Malignant neoplasm of caecum	363350007
1216486011	Oesophageal cancer	363402007
1216488012	Malignant neoplasm of oesophagus	363402007
1216572014	[M]Myeloid leukaemia NOS	37810007
1216595017	[M]Primitive neuroectodermal tumour	39781001
1216670013	[M]Carcinoid tumour, argentaffin, malignant	48554007
1216729017	[M]Hairy cell leukaemia	54087003
1216731014	[M]Epithelioid haemangioendothelioma, malignant	54124005
1216809013	[M]Chronic myeloid leukaemia	63364005
1216868019	[M]Basophilic leukaemia	69077002
1216940017	[M]Adult T-cell leukaemia/lymphoma	77430005

1217360013	Malignant neoplasm, overlapping lesion of hypopharynx	109368005
1217367011	Malignant neoplasm, overlapping lesion of eye and adnexa	109948008
1219467016	Malignant neoplasm, overlapping lesion of nasopharynx	109367000
1219468014	Malignant neoplasm, overlapping lesion of larynx	109369002
1219531013	Malignant overlapping lesion of tongue	109823006
1219533011	Malignant neoplasm, overlapping lesion of floor of mouth	109830000
1219534017	Malignant neoplasm, overlapping lesion of stomach	109836006
1219535016	Malignant neoplasm, overlapping lesion of small intestine	109837002
1219537012	Malignant neoplasm, overlapping lesion of biliary tract	109847004
1219538019	Malignant neoplasm, overlapping lesion of pancreas	109848009
1219540012	Malignant neoplasm, overlapping lesion of penis	109875002
1219545019	Diffuse non-Hodgkin's immunoblastic (diffuse) lymphoma	109966003
1220414014	Hodgkin's disease, nodular sclerosis	118608000
1220814017	[M]AngiocentricT-cell lymphoma	128805001
1220817012	[M]Letterer - Siwe disease	128812005
1220820016	[M]True histiocytic lymphoma	128813000
1220823019	[M]Serous cystadenoma, borderline malignancy	128849004
1220825014	[M]Papillary cystadenoma, borderline malignancy	128850004
1220826010	[M]Papillary serous cystadenoma, borderline malignancy	128851000
1221109014	[M]Hodgkin's disease	14537002
1221209015	[M] Hepatocellular carcinoma, fibrolamellar	15619004
1221282014	[M]Myxoid leiomyosarcoma	16090008
1221446018	[M]Acral lentiginous melanoma, malignant	16974005
1221504012	[M]Carcinoma in pleomorphic adenoma	17264009
1222216017	[M]Follicular carcinoma	5257006
1222219012	[M]Lymphocytic lymphoma NOS	115244002
1222220018	[M]Lymphocytic lymphosarcoma NOS	115244002
1222221019	[M]Lymphoblastic lymphoma NOS	115244002
1222289015	[M]Malignant lymphoma, lymphoplasmacytoid type	19340000
1222303015	[M]Teratoma, malignant, NOS	19467007
1222660012	[M]Osteosarcoma NOS	21708004
1224870013	[M]Hepatocellular carcinoma NOS	25370001
1225763012	[M] Cutaneous lymphoma	28054005
1225879015	[M]Myxosarcoma	28351005
1227295013	Multiple endocrine neoplasia syndrome type 1	30664006
1227435013	[M]Vipoma	31131002
1227517019	[M]Nephroblastoma NOS	31470003
1227518012	[M]Adenosarcoma	31470003
1228259019	[M]Infiltrating duct and lobular carcinoma	35232005
1228267010	[M]Granulocytic sarcoma	35287006
1228268017	[M]Chloroma	35287006
1228269013	[M]Myeloid sarcoma	35287006
1228493018	Malignant neoplasm of cervix uteri	363354003
1228504019	Malignant neoplasm of upper lip, vermilion border	363372009
1228506017	Malignant neoplasm of lower lip, lipstick area	363373004
1228509012	Malignant neoplasm of posterior third of tongue	363376007
1228510019	Malignant neoplasm of major salivary glands	363378008
1228513017	Malignant neoplasm of submandibular gland	363380002
1228523014	Malignant neoplasm of roof of mouth	363390005
1228529013	Malignant neoplasm of palatine tonsil	363393007
1228530015	Malignant neoplasm of faucial tonsil	363393007
1228532011	Malignant neoplasm of roof of nasopharynx	363397008
1228549016	Malignant neoplasm of retroperitoneum	363420003
1228552012	Malignant neoplasm of nasal cavities	363422006
1228553019	Malignant neoplasm of ethmoid sinus	363426009
1228565015	Malignant neoplasm of soft tissue of head	363439008
1228566019	Malignant neoplasm of soft tissue of face	363440005
1228567011	Malignant neoplasm of soft tissue of neck	363441009
1228570010	Cancer of ovary	363443007
1228575017	Malignant neoplasm of prepuce (foreskin)	363450006
1229372013	[M]Linitis plastica	37995004
1229424011	[M]Lipid-rich carcinoma	3839000
1229453012	[M]Astrocytoma NOS	38713004
1229491012	[M]Hodgkin's disease, nodular sclerosis, cellular phase	39086001
1229572014	[M]Malignant melanoma, regressing	39896009
1229648010	[M]Malignant lymphoma, centroblastic type, follicular	40411000
1229767019	[M]Hodgkin's disease, mixed cellularity	41529000
1229971014	[M]Pulmonary blastoma	43149009
1230100013	[M]Malignant melanoma in Hutchinson's melanotic freckle	44474009
1230128019	[M]Choriocarcinoma	44769000
1230392016	[M]Malignant lymphoma, large cell, diffuse NOS	46732000

1230735015	[M]Sezary's disease	4950009
1230800010	[M]Kaposi's sarcoma	49937004
1230828016	[M]Malignant lymphoma, mixed small and large cell, diffuse	50102004
1230838014	[M]Malignant mastocytosis	50150000
1231028010	[M]Desmoplastic melanoma, malignant	51757004
1231237017	[M]Fibrosarcoma NOS	53654007
1231541019	[M]Plasma cell myeloma	55921005
1232439019	[M]Synovial sarcoma NOS	63211008
1232576019	[M]Malignant lymphoma, small lymphocytic NOS	64575004
1232905013	[M]Dedifferentiated liposarcoma	67280001
1233222010	[M]Carcinomatosis	7010000
1233285010	[M]Hodgkin,s disease, lymphocytic predominance, nodular	70600005
1233711012	[M]Hodgkin's granuloma	74189002
1233722011	[M]Glycogen-rich carcinoma	74280008
1234006011	Dermatofibrosarcoma protuberans	76594008
1234144017	Polyneuropathy in malignant disease	77659000
1234197011	[M]Malignant lymphoma, immunoblastic type	450958009
1234296013	[M]Pleomorphic xanthoastrocytoma	78838008
1234787012	[M]Rhabdoid sarcoma	83118000
1235290011	[M]Giant cell sarcoma (except of bone)	87992000
1235321016	[M]Alveolar soft part sarcoma	88195001
1235785014	Malignant melanoma of external surface of cheek	93217003
1235786010	Malignant melanoma of eyebrow	93223008
1235798017	Malignant melanoma of external surface of nose	93643005
1235814012	Secondary malignant neoplasm of bone and bone marrow	94222008
1235875018	Secondary malignant neoplasm of peritoneum	94627008
1235961012	[M]Epithelial-myoepithelial carcinoma	9618003
1488780018	Cancer diagnosis discussed	395081000
1488798019	Cancer confirmed	395099008
1786811017	Malignant neoplasm of urinary bladder	399326009
2164653019	Osteosarcoma	307576001
2475562018	Malignant neoplasm of interlobular biliary canals	187776004
2475564017	Malignant neoplasm of turbinate	187830009
2475984012	Malignant neoplasm of palatoglossal arch	254459004
2534415018	Acute monocytic leukaemia	413441006
2534467019	Extranodal NK/T-cell lymphoma, nasal type	414166008
2534468012	Angioimmunoblastic T-cell lymphoma	1090241000000107
2549374014	Malignant sacral teratoma	416842003
2550105015	[M]Gliomas	115240006
2552865010	[M]Glioma NOS	74532006
2619456011	Malignant neoplasm of intrahepatic biliary passages	187777008
2659693012	Gamma heavy chain disease	109984001
2659756011	Peripheral T-cell lymphoma	109977009
2659763011	Alpha heavy chain disease	109982002
2659772015	Histiocytic sarcoma	109988003
2663187010	Hodgkin's disease, lymphocytic depletion	118610003
2663190016	Burkitt's lymphoma	118617000
2663191017	Mycosis fungoides	118618005
2663376010	Hodgkin's sarcoma	118606001
2663461018	Hodgkin's disease, mixed cellularity	118609008
2663473018	Hodgkin's disease	118599009
2663476014	Hodgkin's granuloma	118602004
2692069010	Malignant neoplasm of brain	428061005
2839871014	Non-Hodgkin lymphoma	118601006
2870724014	Lymphocyte-rich classical Hodgkin lymphoma	118607005
2870725010	Mediastinal (thymic) large B-cell lymphoma	444910004
2871720012	Juvenile myelomonocytic leukaemia	445227008
2872841013	Hepatosplenic T-cell lymphoma	445406001
2901912012	Diffuse follicle centre lymphoma	449220000
3526034015	Malignant melanoma of skin of right lower limb	352001000119100
3526497018	Microsatellite instability-high colorectal cancer	737058005
3634396017	Malignant perineurioma	761958009
3636681010	Primary endometrioid carcinoma of endometrium of body of uterus	762458004
81401000006110	Unspec malig neop lymphoid/histiocytic intra-abdominal nodes	118600007
81411000006113	Unspec malig neop lymphoid/histiocytic lymph node axilla/arm	118600007
81421000006117	Unspec malig neop lymphoid/histiocytic lymph node head/neck	118600007
81431000006119	Unspec malig neop lymphoid/histiocytic nodes inguinal/leg	118600007
81441000006112	Unspec malig neop lymphoid/histiocytic of intrapelvic nodes	118600007
81461000006111	Unspec malig neop lymphoid/histiocytic of multiple sites	118600007
81481000006118	Unspec malig neop lymphoid/histiocytic of unspecified site	118600007
109371000006115	Malignant teratoma of descended testis	417554000

109381000006117	Malignant teratoma of testis	416769008
139571000006117	Solitary myeloma	415112005
146651000006118	Sezary's disease	118611004
151101000006116	Secondary malig neop of large intestine or rectum NOS	94365007
151111000006118	Secondary malignant neoplasm of respiratory and digestive systems	269473008
151121000006114	Secondary malig neop of respiratory or digestive system NOS	269473008
151131000006112	Secondary malignant neoplasm of retroperitoneum and peritoneum	188445006
151141000006119	Secondary malig neop of retroperitoneum or peritoneum NOS	188445006
151151000006117	Secondary malig neop of small intestine or duodenum NOS	94580002
151321000006111	Secondary malignant neoplasm of large intestine	94365007
151331000006114	Secondary malignant neoplasm of liver	94381002
151401000006110	Secondary malignant neoplasm of other specified site NOS	128462008
151421000006117	Secondary malignant neoplastic disease	128462008
151621000006119	Secondary malignant neoplasm of small intestine	94580002
152611000006116	Secondary unspec malig neop lymph nodes head/face/neck NOS	303194003
157481000006111	Secondary malignant neoplasm of mediastinal lymph nodes	94408005
157491000006114	Secondary malignant neoplasm of anterior cervical lymph nodes	1090131000000106
157501000006118	Secondary malignant neoplasm of lymph nodes of upper limb	94398002
157511000006115	Secondary and unspec malig neop axilla and upper limb LN NOS	94398002
157521000006111	Secondary malignant neoplasm of axillary lymph nodes	94181007
157531000006114	Secondary and unspec malig neop bronchopulmonary lymph nodes	94227002
157541000006116	Secondary malignant neoplasm of circumflex iliac lymph nodes	1090211000000106
157551000006119	Secondary malignant neoplasm of coeliac lymph nodes	1090191000000107
157561000006117	Secondary malignant neoplasm of iliac lymph nodes	94336001
157571000006112	Secondary malignant neoplasm of deep cervical lymph nodes	1090121000000109
157581000006110	Secondary malignant neoplasm of deep inguinal lymph nodes	1090201000000109
157591000006113	Secondary malignant neoplasm of parotid lymph nodes	94475009
157601000006117	Secondary malignant neoplasm of diaphragmatic lymph nodes	1090161000000101
157611000006119	Secondary and unspec malig neop external iliac lymph nodes	94336001
157621000006110	Secondary malignant neoplasm of inferior epigastric lymph nodes	1090181000000105
157631000006113	Secondary malignant neoplasm of mesenteric lymph nodes	94410007
157641000006115	Secondary malignant neoplasm of tracheobronchial lymph nodes	94642006
157651000006118	Secondary malignant neoplasm of infraclavicular lymph nodes	94338000
157661000006116	Secondary and unspec malig neop inguinal and lower limb LN	94395004
157671000006111	Secondary malignant neoplasm of intercostal lymph nodes	94344001
157681000006114	Secondary malignant neoplasm of internal iliac lymph nodes	1090221000000100
157691000006112	Secondary malignant neoplasm of internal mammary lymph nodes	1090091000000108
157701000006112	Secondary and unspec malig neop intra-abdominal LN NOS	94347008
157711000006110	Secondary and unspec malig neop intra-abdominal lymph nodes	94347008
157721000006119	Secondary and unspec malig neop intrapelvic LN NOS	94350006
157731000006116	Secondary and unspec malig neop intrapelvic lymph nodes	94350006
157741000006114	Secondary and unspec malig neop intrathoracic LN NOS	94351005
157751000006111	Secondary and unspec malig neop intrathoracic lymph nodes	94351005
157771000006118	Secondary and unspec malig neop lymph nodes multiple sites	303201005
157781000006115	Secondary and unspec malig neop lymph nodes NOS	94392001
157791000006117	Secondary malignant neoplasm of obturator lymph nodes	94446003
157801000006116	Secondary malignant neoplasm of lymph nodes of face	94393006
157811000006118	Secondary and unspec malig neop of inguinal and leg LN NOS	94395004
157821000006114	Secondary malignant neoplasm of parotid lymph nodes	94475009
157831000006112	Secondary and unspec malig neop paratracheal lymph nodes	1090081000000106
157841000006119	Secondary malignant neoplasm of pectoral axillary lymph nodes	94477001
157851000006117	Secondary malignant neoplasm of popliteal lymph nodes	94494004
157861000006115	Secondary and unspec malig neop post mediastinal lymph nodes	94408005
157871000006110	Secondary malignant neoplasm of bronchopulmonary lymph nodes	94227002
157881000006113	Secondary malignant neoplasm of sacral lymph nodes	1090171000000108
157891000006111	Secondary malignant neoplasm of submandibular lymph nodes	94609005
157901000006110	Secondary malignant neoplasm of submental lymph nodes	94611001
157911000006113	Secondary malignant neoplasm of tracheobronchial lymph nodes	94642006
157921000006117	Secondary malignant neoplasm of superficial cervical lymph nodes	1090141000000102
157931000006119	Secondary malignant neoplasm of superficial inguinal lymph nodes	94612008
157941000006112	Secondary malignant neoplasm of mesenteric lymph nodes	94410007
157951000006114	Secondary malignant neoplasm of supratrochlear lymph nodes	1090101000000100
157961000006111	Secondary malignant neoplasm of mastoid lymph nodes	1090151000000104
157971000006116	Secondary malignant neoplasm of occipital lymph nodes	94449005
177051000006111	Malignant tumour of kidney	363518003
202801000006110	Prolymphocytic leukaemia	110006004
226911000000117	Date cancer diagnosis received in primary care	166851000000104
247091000006118	Overlapping malignant lesion of retroperitoneum and peritoneum	187807003
304071000006110	[M] Angioendotheliomatosis	255102004
304251000006114	[M]Acidophil carcinoma	51217003
304381000006111	Acute monocytic leukaemia	22331004

304401000006111	Acute myeloid leukaemia	17788007
304431000006115	[M]Acute panmyelosis	80570006
304471000006117	[M]Adamantinoma of long bones	56763007
304481000006119	[M]Adamantinoma, malignant	88253001
304491000006116	[M]Adenoacanthoma	15176003
304701000006118	Adenoid cystic carcinoma	11671000
304901000006115	[M]Adenosarcoma	31470003
304921000006113	Adenosquamous carcinoma	59367005
305011000006117	Adrenal cortical carcinoma	2227007
305271000006115	[M]Ameloblastic odontosarcoma	20380000
305331000006114	[M]Androblastoma, malignant	12323008
305531000006115	[M]Angiomyosarcoma	28953002
305651000006111	[M]Arrhenoblastoma, malignant	12323008
305671000006118	[M]Astroblastoma	48952003
305741000006115	[M]Balloon cell melanoma	39274007
305841000006110	Basaloid carcinoma	5843004
305941000006117	Bile duct carcinoma	70179006
306001000006110	[M]Blast cell leukaemia	24072005
306011000006113	[M]Blastic leukaemia	24072005
306091000006115	[M]Brenner tumour, malignant	42194009
306111000006112	[M]Brill - Symmers' disease	115244002
306131000006118	[M]Bronchiolar carcinoma	112677002
306171000006115	[M]Burkitt's cell leukaemia	22197008
306381000006115	[M]Carcinoma simplex	45881000
306641000006116	Cholangiocarcinoma	70179006
306661000006117	[M]Chondroblastic osteosarcoma	76312009
306681000006110	[M]Chondroblastoma, malignant	74279005
306821000006110	[M]Choriocarcinoma combined with teratoma	189857001
306901000006111	Chromophobe carcinoma	1443001
306931000006115	Chronic lymphoid leukaemia	51092000
306971000006117	Chronic myelomonocytic leukaemia	128831004
307071000006118	[M]Cloacogenic carcinoma	84570003
307251000006119	[M]Congenital fibrosarcoma	52040006
307271000006112	[M]Cribriform carcinoma	30156004
307531000006112	[M]Dermoid cyst with malignant transformation	189849004
307571000006110	[M]Desmoplastic medulloblastoma	32456001
307631000006111	Duct carcinoma	82711006
307921000006115	[M]Endometrioid adenofibroma, malignant	18105004
307971000006119	Endometrioid carcinoma	30289006
308001000006111	[M]Endothelial bone sarcoma	76909002
308031000006115	[M]Eosinophil carcinoma	51217003
308041000006113	[M]Eosinophilic leukaemia	190055003
308071000006117	[M]Ependymoblastoma	21589007
308111000006113	Epidermoid carcinoma	28899001
308121000006117	[M]Epidermoid carcinoma, keratinising type	18048008
308181000006118	[M]Epithelioid cell melanoma	37138001
308241000006115	[M]Epithelioid leiomyosarcoma	42392001
308261000006116	Malignant epithelioid mesothelioma	65278006
308291000006112	[M]Epithelioma, malignant	71298006
308371000006119	[M]Extra-adrenal paraganglioma, malignant	32512003
308461000006115	[M]Fibrillary astrocytoma	71314006
308501000006115	[M]Fibrochondrosarcoma	14990007
308551000006116	[M]Fibroliposarcoma	49430005
308701000006114	[M]Fibrous mesothelioma, malignant	54443001
308731000006118	Malignant fibroxanthoma	34360000
308801000006118	[M]Follicular lymphosarcoma NOS	115244002
308861000006117	[M]Ganglioneuroblastoma	69515008
308941000006114	Malignant gastrinoma	19756007
309071000006111	[M]Giant cell bone sarcoma	10069009
309091000006112	[M]Giant cell glioblastoma	44529004
309181000006110	[M]Giant follicular lymphoma	115244002
309261000006112	[M]Glioma NOS	74532006
309281000006119	Malignant glioma	74532006
309301000006115	[M]Gliomatosis cerebri	26138003
309361000006119	[M]Glomoid sarcoma	13875003
309401000006112	Malignant glucagonoma	66515009
309521000006115	[M]Granulocytic leukaemia NOS	37810007
309641000006111	[M]Haemangioendothelioma, malignant	33176006
309731000006110	[M]Haemangiopericytoma, malignant	84664004
309891000006115	[M]Hepatoma, malignant	25370001
309991000006111	[M]Hodgkin's disease NOS	128930002

310311000006110	Infiltrating duct carcinoma	82711006
310321000006119	Infiltrating ductular carcinoma	58477004
310341000006114	[M]Inflammatory carcinoma	32968003
310361000006113	Malignant insulinoma	20955008
310411000006117	[M]Intracystic carcinoma NOS	703545003
310531000006110	[M]Intraepithelial carcinoma NOS	109355002
310571000006113	[M]Intraosseous carcinoma	26888009
310731000006117	[M]Juvenile astrocytoma	128854008
310791000006118	[M]Juxtacortical chondrosarcoma	26211003
310801000006117	[M]Juxtacortical osteogenic sarcoma	115237006
310961000006112	[M]Lentigo maligna melanoma	302836005
310971000006117	[M]Leptomeningeal sarcoma	78303004
310991000006116	[M]Leukaemia NOS	128931003
311041000006118	Hairy cell leukaemia	54087003
311301000006118	[M]Lymphangiosarcoma	63373002
311311000006115	[M]Lymphatic leukaemia	32280000
311351000006119	[M]Lymphoblastic lymphosarcoma NOS	115244002
311361000006117	[M]Lymphoblastoma NOS	115244002
311391000006113	[M]Lymphoepithelial carcinoma	7300000
311431000006119	[M]Lymphoma NOS	21964009
311461000006111	Malignant lymphoma, follicular AND/OR nodular	115245001
311521000006118	[M]Lymphosarcoma NOS	115244002
311561000006112	[M]Malignant lymphoma, follicular centre cell, cleaved, follicular	115244002
311571000006117	[M]Malignant lymphoma, lymphocytic, intermediate differentiation, nodular	115244002
311581000006119	[M]Malignant lymphoma, lymphocytic, poorly differentiated, nodular	115244002
311591000006116	[M]Malig lymp,follicular centre cell,noncleaved,follicular	115244002
311601000006112	[M]Malignant lymphoma, follicular centre cell, non-cleaved NOS	115244002
311611000006110	[M]Malignant lymphoma, lymphocytic, intermediate differentiation NOS	115244002
311621000006119	[M]Malignant lymphoma, lymphocytic, well differentiated, nodular	115244002
311631000006116	[M]Malignant lymphoma, mixed lymphocytic-histiocytic, nodular	55020008
311641000006114	[M]Malignant lymphoma, lymphocytic, intermediate differentiation, diffuse	74654000
311671000006118	Malignant lymphoma	21964009
311801000006113	[M]Malignant lymphoma, large cell, noncleaved, diffuse	115244002
311811000006111	[M]Malignant lymphoma, lymphocytic, poorly differentiated NOS	115244002
311871000006119	[M]Malignant lymphoma, non-Hodgkin's type	1929004
311881000006116	[M]Malignant lymphoma, small cell, noncleaved, diffuse	115244002
311911000006116	[M]Malignant lymphoma, stem cell type	189962004
312021000006118	[M]Malignant teratoma, intermediate type	21912003
312031000006115	[M]Malignant teratoma, trophoblastic	65646006
312041000006113	Malignant teratoma - undifferentiated	83292005
312061000006112	[M]Malignant tumour, giant cell type	83950009
312101000006110	[M]Mast cell sarcoma	13583002
312161000006111	Medullary carcinoma with amyloid stroma	128916007
312171000006116	[M]Medullary carcinoma with lymphoid stroma	85654004
312201000006117	[M]Medullomyoblastoma	24604009
312251000006118	[M]Melanocarcinoma	2092003
312291000006112	[M]Melanosarcoma NOS	2092003
312331000006117	[M]Meningeal sarcomatosis	14494009
312361000006114	[M]Meningioma, malignant	78303004
312391000006118	[M]Meningothelial sarcoma	78303004
312421000006114	[M]Mesenchymal chondrosarcoma	56565002
312461000006115	[M]Mesenchymoma, malignant	89623007
312491000006111	[M]Mesodermal mixed tumour	84427001
312591000006110	Malignant biphasic mesothelioma	30383009
312601000006119	Mesothelioma, malignant	62064005
312631000006110	[M]Metatypical carcinoma	6641007
312841000006116	[M]Mixed cell rhabdomyosarcoma	62383007
312851000006119	[M]Mixed epithelioid and spindle melanoma	50813003
312871000006112	[M]Mixed glioma	253071005
312951000006111	[M]Mixed type liposarcoma	11073003
312961000006113	[M]Monocytic leukaemia NOS	87163000
313001000006116	[M]Monostotic myeloma	10639003
313131000006113	Mucoepidermoid carcinoma	4079000
313171000006111	[M]Mucoid adenocarcionoma	72495009
313271000006116	[M]Multiple myeloma	109989006
313301000006119	Mycosis fungoides	90120004
313311000006116	[M]Mycosis fungoides	90120004
313381000006111	[M]Myeloma NOS	55921005
313391000006114	[M]Myelomatosis	55921005
313411000006114	[M]Myelosis NOS	37810007
313501000006112	[M]Myosarcoma	20667008

313561000006113	[M]Myxoliposarcoma	27849002
313621000006115	[M]Naegeli-type monocytic leukaemia	63364005
313651000006112	[M]Naevocarcinoma	2092003
313691000006118	Malignant tumour - uncertain whether primary or metastatic	6219000
313701000006118	Malignant neoplasm	363346000
313801000006114	[M]Neurilemmoma, malignant	77418004
313931000006111	[M]Neurofibrosarcoma	404037002
314071000006112	[M]Nonencapsulated sclerosing carcinoma	62681000
314141000006117	[M]Odontogenic fibrosarcoma	27092008
314201000006112	Malignant odontogenic tumour	26888009
314241000006114	[M]Olfactory neuroepithelioma	68614005
314251000006111	[M]Olfactory neurogenic tumour	53968002
314261000006113	[M]Oligodendroblastoma	80061003
314321000006114	[M]Orchioblastoma	74409009
314351000006117	[M]Osteoblastic sarcoma	21708004
314401000006113	[M]Osteochondrosarcoma	21708004
314421000006115	[M]Osteoclastoma, malignant	10069009
314501000006114	[M]Osteosarcoma in Paget's disease of bone	33681003
314521000006116	Lymphoid leukaemia	32280000
314671000006116	Paget's disease and infiltrating duct carcinoma of breast	82591005
314851000006110	[M]Papillary cystadenoma, borderline malignancy	128850004
314891000006116	[M]Papillary epidermoid carcinoma	39056008
314991000006112	[M]Papillary serous cystadenoma, borderline malignancy	128851000
315101000006118	Malignant paraganglioma	9903002
315161000006117	[M]Parosteal osteosarcoma	115237006
315221000006118	[M]Periosteal osteogenic sarcoma	115237006
315251000006110	Malignant phaeochromocytoma	29370006
315301000006115	[M]Piloid astrocytoma	128854008
315331000006111	[M]Pineoblastoma	31671006
315461000006113	[M]Plasmacytic myeloma	55921005
315501000006113	Pleomorphic carcinoma	16741004
315541000006110	[M]Pleomorphic rhabdomyosarcoma	77455004
315581000006116	[M]Pneumoblastoma	112685006
315771000006113	[M]Pseudosarcomatous carcinoma	23109009
315951000006117	[M]Reticulosarcoma NOS	189982000
315971000006110	[M]Reticulosarcoma, nodular	189985003
315991000006111	[M]Reticulosarcomas	189982000
316001000006112	[M]Reticulum cell sarcoma NOS	189982000
316041000006114	[M]Retinoblastoma, undifferentiated type	12354007
316121000006118	[M]Round cell liposarcoma	43296007
316131000006115	[M]Round cell sarcoma	73506006
316141000006113	[M]Sarcoma botryoides	14269005
316181000006119	[M]Schneiderian carcinoma	5600009
316211000006115	[M]Schwannoma, malignant	77418004
316291000006113	[M]Secondary carcinoma	79282002
316311000006112	[M]Secretory breast carcinoma	41919003
316331000006118	[M]Seminoma, anaplastic type	72907003
316401000006117	Serous surface papillary carcinoma	15674004
316511000006118	Signet ring carcinoma	87737001
316611000006117	[M]Small cell sarcoma	73506006
316691000006110	[M]Solid carcinoma with amyloid stroma	128916007
316711000006113	[M]Solitary myeloma	10639003
316741000006112	[M]Spermatocytic seminoma	9294008
316801000006119	[M]Spindle cell melanoma, type B	40244008
317011000006112	[M]Stem cell leukaemia	24072005
317041000006111	[M]Struma ovarii, malignant	18854008
317341000006113	[M]Synovial sarcoma, biphasic type	18588008
317361000006112	[M]Synovial sarcoma, spindle cell type	37206003
317411000006116	[M]Telangiectatic osteosarcoma	78453009
317421000006112	[M]Teratoblastoma, malignant	19467007
317441000006117	[M]Teratoid medulloepithelioma	88591002
317571000006112	[M]Thrombocytic leukaemia	52220008
317611000006119	Malignant thymoma	15949004
318001000006113	Urothelial carcinoma	27090000
318051000006112	[M]Verrucous epidermoid carcinoma	89906000
318171000006118	Waldenstrom's macroglobulinaemia	35562000
318231000006110	[M]Wolffian duct carcinoma	2221008
318261000006118	[M]Yolk sac tumour	74409009
359271000006110	Secondary malignant neoplasm of urinary system	94663008
359281000006113	Secondary malignant neoplasm of nervous system	94442001
359291000006111	Secondary malignant neoplasm of lymph nodes of multiple sites	94396003

396091000006118	[X]Mal neoplasm/connective+soft tissue of trunk,unspecified	269469005
396101000006112	Malignant tumour of lymphoid haemopoietic and related tissue	269475001
396111000006110	Overlapping malignant neoplasm of peripheral nerves and autonomic nervous system	109919002
396131000006116	Malignant neoplasm of peripheral nerves and autonomic nervous system	188321006
396161000006113	Overlapping malignant neoplasm of brain and other parts of the central nervous system	109911004
396171000006118	[X]Malignant melanoma of other+unspecified parts of face	93225001
396191000006117	[X]Malignant neopl/overlapping les/resp+intrathoracic organs	93841009
396251000006115	Malignant neoplasm of central nervous system	372062007
396281000006111	[X]Malignant neoplasm of ill-defined, secondary and unspecified sites	363346000
396301000006110	[X]Malignant neoplasm of lip, oral cavity and pharynx	271323007
396381000006118	[X]Malignant neoplasm of respiratory and intrathoracic organs	448708002
396461000006117	Malignant neoplasm of multiple endocrine glands	1090931000000108
396471000006112	[X]Malignant neoplasm/bone+articular cartilage, unspecified	443679004
396481000006110	Malignant neoplasm of bone and articular cartilage of limb	1090871000000102
396491000006113	Malignant neoplasm of central nervous system	372062007
396501000006117	[X]Malignant neoplasm/connective + soft tissue,unspecified	269469005
396511000006119	[X]Malignant neoplasm/ill-defined sites within resp system	449096009
396521000006110	Malignant tumour of cranial nerve	188307009
396531000006113	Malignant tumor of female genital organ	363514001
396541000006115	[X]Malignant neoplasm/other specified male genital organs	363515000
396551000006118	Overlapping malignant neoplasm of bone and articular cartilage	109347009
396561000006116	[X]Malignant neoplasm/overlap lesion/heart,mediastinm+pleura	109384006
396571000006111	Overlapping malignant neoplasm of ill-defined site	109358000
396581000006114	Overlapping malignant neoplasm of female genital organs	109878000
396591000006112	Overlapping malignant neoplasm of male genital organs	109874003
396601000006116	Malignant neoplasm of peripheral nerves of trunk	1090901000000102
396611000006118	[X]Malignant neoplasm/upper resp tract, part unspecified	187833006
396621000006114	[X]Malignant neoplasms of lymphoid, haematopoietic and related tissue	269475001
396631000006112	[X]Malignant neoplasms/independent (primary) multiple sites	188478004
396641000006119	[X]Malignant neoplasms/independent(primary)multiple sites	188478004
396651000006117	Malignant neoplasm of gastrointestinal tract	428905002
398291000006110	[X]Mesothelioma of lung	109378008
400011000006119	[X]Non-Hodgkin's lymphoma NOS	118601006
404801000006116	[X]Oth malignant neoplasm/skin of oth+unspecfd parts of face	94047004
405081000006118	[X]Oth spcf mal neoplsm/lymphoid,haematopoietic+rltd tissue	269475001
425771000006113	Secondary malignant neoplasm of respiratory tract	94515004
425781000006111	Secondary malignant neoplasm of gastrointestinal tract	94313005
456641000006119	Acute erythraemia and erythroleukaemia	93451002
472961000000117	Patient on regional cancer register	276101000000100
473991000000119	Malignant neoplasm of descended testis	1090261000000108
531851000006119	Ca female breast	372064008
535221000006113	Carcinoma of gallbladder	372140005
537051000006118	Carcinoma of digestive organs and peritoneum	269456006
543111000006111	Cerebral tumour - malignant	428061005
543481000006111	Malignant tumour of cervix	363354003
552661000006114	Choriocarcinoma	188188009
554641000006115	Chronic granulocytic leukaemia	92818009
573611000006118	Colonic cancer	363406005
598801000000114	Excision biopsy of rodent ulcer	428796002
621091000006113	Diffuse non-Hodgkin's lymphoma, unspecified	109962001
641301000000114	Malignant multiple sclerosis	439567002
680761000006117	Neo/uncertn+unknwn behav/lymph,h'matopetc+rel tiss,unspcf	255048006
682691000006117	Myopathy due to malignant disease	71985006
683191000006119	Myeloma - solitary	415112005
704711000006119	Metastases of respiratory and/or digestive systems	269473008
705211000006111	Malignant mesothelioma of pleura	254645002
716861000006117	Mast cell disease	78745000
716871000006112	Mast cell leukaemia	110002002
719021000006111	Malignant neoplasm of frontal sinus	363427000
719931000006113	Malignant neoplasm, overlapping lesion of major salivary glands	109824000
720001000006119	Malignant neoplasm, overlapping lesion of peripheral nerves and autonomic nervous system	109919002
720011000006116	Malignant neoplasm-pluriglandular involvement,unspecified	1090931000000108
720021000006112	Malignant neoplasm/bones+articular cartilage/limb,unspfd	1090871000000102
720031000006110	Malignant neoplasm/overlap lesion/bone+articulr cartilage	109347009
720041000006117	Malignant neoplasm/overlapping lesion/feml genital organs	109878000
720051000006115	Malignant neoplasm/peripheral nerves of trunk,unspecified	1090901000000102
720341000006115	Malignant neoplasm of vertebral column	363438000
720451000006113	Malignant neoplasm other gallbladder/extrahepatic bile duct	363415003
720461000006110	Malignant neoplasm other spec digestive tract and peritoneum	255077007

720471000006115	Malignant neoplasm overlapping lesion of skin	109264009
720481000006117	Malignant neoplasm of pituitary gland and craniopharyngeal duct	188339002
720491000006119	Malignant tumour of posterior margin of nasal septum and choanae	254484001
720501000006110	Malignant neoplasm rectum,rectosigmoid junction and anus NOS	187760008
720521000006117	Malignant neoplasm of skin of face	443136000
720531000006119	Malignant neoplasm skin other and unspec part of face NOS	443136000
720561000006111	Malignant neoplasm, overlapping lesion of bone and articular cartilage of limbs	109348004
720571000006116	Malignant neoplasm, overlap lesion connective & soft tissue	188026007
720581000006118	Primary malignant neoplasm of intrathoracic organs	93841009
720591000006115	Malignant neoplasm, overlapping lesion of male genital organs	109874003
720631000006115	Overlapping malignant neoplasm of brain	109912006
720761000006115	Malignant neoplasm of upper gum	363383000
720791000006111	Malignant tumour of upper labial mucosa	187606005
720801000006112	Malignant tumour of vermilion border of lip	421249001
720951000006119	Malignant neoplasm of urachus	363456000
720961000006117	Malignant tumour of ureter	363458004
720991000006113	Malignant neoplasm of urethra	363459007
721041000006111	Malignant neoplasm of uvula	363389001
721051000006113	Malignant tumour of vagina	363445000
721091000006119	Malignant neoplasm of sweat gland	94081005
721171000006119	Malignant tumour of testis	363449006
721271000006114	Malignant neoplasm of thymus	363434003
721291000006110	Malignant tumour of thyroid gland	363478007
721311000006114	Malignant tumour of tongue	363375006
721351000006110	Malignant tumour of tonsil	363393007
721361000006112	Malignant neoplasm of tonsillar fossa	363394001
721391000006116	Malignant neoplasm of trachea	363432004
721421000006112	Malignant tumour of transverse colon	363408006
721481000006111	Malignant neoplasm of vallecula	363395000
721601000006117	Malignant tumour of small intestine	363509000
721621000006110	Malignant neoplasm of soft palate	363388009
721701000006112	Malignant neoplasm of spermatic cord	363453008
721721000006119	Malignant neoplasm of sphenoidal sinus	363428005
721751000006111	Malignant neoplasm of spinal cord	363475005
721821000006114	Malignant neoplasm of splenic flexure of colon	363413005
721851000006117	Malignant tumour of stomach	363349007
721871000006110	Malignant neoplasm of subglottis	363430007
721881000006113	Malignant neoplasm of sublingual gland	363381003
721961000006111	Malignant neoplasm of septum of nose	363423001
721991000006115	Malignant tumour of sigmoid colon	363410008
722341000006119	Malignant neoplasm of prepylorus of stomach	1090291000000102
722361000006115	Malignant tumour of prostate	399068003
722421000006115	Malignant neoplasm of pyriform sinus	363401000
722441000006110	Malignant tumour of rectosigmoid junction	363414004
722471000006119	Malignant neoplasm of renal calyces	188252005
722481000006116	Malignant tumour of renal pelvis	363457009
722511000006112	Malignant tumour of undescended testis	188219004
722521000006116	Malignant neoplasm of retina	363465007
722541000006111	Malignant neoplasm of retromolar area	363391009
722761000006119	Malignant neoplasm of pelvis	363484005
722871000006117	Malignant neoplasm of peripheral nerves of lower limb, including hip	188324003
722901000006117	Malignant neoplasm of peripheral nerves of upper limb, including shoulder	188323009
722911000006119	Malignant neoplasm of peripheral nerves of head, face and neck	188322004
722961000006116	Malignant neoplasm of pineal gland	363483004
722981000006114	Malignant neoplasm of pituitary gland	363482009
723001000006113	Malignant neoplasm of pleura	363433009
723031000006117	Malignant neoplasm of postcricoid region	363400004
723111000006110	Malignant neoplasm of scrotum	363454002
723221000006112	Malignant neoplasm of other specified site small intestine	363509000
723281000006111	Malignant tumour of ovary	363443007
723301000006110	Malignant neoplasm of overlapping lesion of bronchus and lung	109371002
723321000006117	Overlapping malignant neoplasm of tonsil	110013004
723341000006112	Overlapping malignant neoplasm of vulva	109885001
723391000006115	Malignant neoplasm of palatopharyngeal arch	187675005
723401000006118	Malignant tumour of pancreas	363418001
723441000006116	Malignant neoplasm of parathyroid gland	363481002
723451000006119	Malignant neoplasm of paraurethral glands	363460002
723501000006117	Malignant tumour of parotid gland	363379000
723541000006115	Malignant neoplasm of orbit	363462005
723571000006111	Malignant tumour of oropharynx	363392002
723601000006116	Malignant neoplasm of other and ill defined site NOS	302817000

723641000006119	Malignant neoplasm of other and unspecified site OS	363346000
723801000006115	Malignant neoplasm of other sites lip, oral cavity, pharynx	271323007
723951000006113	Malignant neoplasm of metatarsal bones of foot	712525007
724051000006110	Malignant neoplasm of myocardium	363437005
724311000006111	Malignant neoplasm of lower gum	363384006
724401000006116	Malignant tumour of lower labial mucosa	271568003
724421000006114	Malignant neoplasm of lower lip, vermilion border	363373004
724621000006112	Malignant neoplasm of mastoid air cells	363424007
724641000006117	Malignant neoplasm of maxillary sinus	363425008
724671000006113	Malignant neoplasm of mediastinum, part unspecified	363494000
724681000006111	Malignant neoplasm of meninges, unspecified	363497007
724721000006116	Malignant neoplasm of lacrimal duct	188272000
724761000006110	Malignant neoplasm of laryngeal cartilage	363431006
724781000006117	Malignant neoplasm of laryngopharynx	363399006
724791000006119	Malignant tumour of larynx	363429002
724841000006116	Malignant neoplasm of lateral wall of nasopharynx	363398003
724881000006110	Malignant neoplasm of lingual tonsil	363377003
724891000006113	Malignant neoplasm of lip	363348004
724901000006112	Malignant neoplasm of lip unspecified, buccal aspect	187622006
725101000006111	Malignant neoplasm of hard palate	363387004
725121000006118	Malignant tumour of head of pancreas	363419009
725151000006110	Malignant neoplasm of heart	363435002
725191000006116	Malignant tumour of hepatic flexure	363407001
725221000006111	Malignant neoplasm of histiocytic tissue	269475001
725241000006116	Malignant neoplasm of hypopharynx	363399006
725271000006112	Malignant tumour of ileum	363405009
725451000006118	Malignant neoplasm of jejunum	363404008
725611000006117	Malignant neoplasm of floor of mouth	363385007
725711000006113	Malignant tumour of gallbladder	363353009
725761000006111	Malignant neoplasm of genitourinary organ OS	271468000
725771000006116	Malignant neoplasm of glans penis	363451005
725811000006116	Malignant tumour of anterior pillar of fauces	254459004
725861000006118	Malignant neoplasm of gum	363382005
725951000006112	Malignant tumour of descending colon	363409003
726021000006110	Malignant tumour of duodenum	363403002
726071000006111	Malignant neoplasm of endocardium	363436001
726141000006116	Malignant neoplasm of epididymis	363452003
726201000006111	Malignant tumour of extrahepatic bile duct	363416002
726261000006112	Malignant tumour of eyelid	231829006
726271000006117	Malignant neoplasm of fallopian tube	363444001
726331000006111	Malignant neoplasm of colon	363406005
726351000006116	Malignant neoplasm of commissure of lip	363374005
726361000006119	Malignant neoplasm of common bile duct	93763008
726371000006114	Malignant neoplasm of conjunctiva	363463000
726561000006114	Malignant neoplasm of cornea	363464006
726631000006118	Malignant neoplasm of costo-vertebral joint	187926007
726641000006111	Malignant neoplasm of cranial nerves	188307009
727151000006117	Malignant neoplasm of cheek mucosa	363386008
727181000006113	Malignant neoplasm of choroid	363466008
727281000006117	Malignant neoplasm of appendix	363411007
727321000006111	Malignant tumour of ascending colon	363412000
727381000006110	Malignant tumour of base of tongue	363376007
727511000006114	Malignant neoplasm of brainstem	363473003
727521000006118	Malignant neoplasm of brain stem NOS	363473003
727531000006115	Malignant neoplasm of branchial cleft	363396004
727561000006112	Malignant neoplasm of buccal mucosa	363386008
727731000006113	Malignant melanoma of upper limb	269580008
727761000006116	Malignant neoplasm of aryepiglottic fold, hypopharyngeal aspect	187708004
727771000006111	Malignant neoplasm of cerebrum (excluding lobes and ventricles)	188280007
727781000006114	Malignant neoplasm gallbladder and extrahepatic bile ducts	363415003
727791000006112	Malignant neoplasm gallbladder/extrahepatic bile ducts NOS	363415003
727811000006111	Malignant neoplasm lymphatic or haematopoietic tissue OS	269475001
727841000006110	Malignant neoplasm of acoustic nerve	254980001
727911000006116	Malignant neoplasm of ampulla of Vater	363417006
728201000006110	Malignant melanoma of eyelid	231834005
728451000006110	Malignant neoplasm of connective and soft tissue of sacrum or coccyx	188023004
728461000006112	Malig neopl peripheral nerves and autonomic nervous system	188321006
728471000006117	Malig neopl, overlap lesion brain & other part of CNS	109911004
728481000006119	Malignant neoplasm of connective and soft tissues of lumbar spine	188017009
728491000006116	Malignant neoplasm of connective and soft tissues of thoracic spine	188013008
728721000006111	Malignant lymphoma	118600007

728771000006112	Malignant lymphoma of lymph nodes of inguinal region AND/OR lower limb	93196000
728781000006110	Malignant lymphoma of lymph nodes of axilla AND/OR upper limb	93194002
729281000006113	Malig neop connective and soft tissue other specified site	269469005
729291000006111	Malignant neoplasm of connective and soft tissue of upper limb and shoulder	187991006
729301000006112	Malig neop connective soft tissue upper limb/shoulder NOS	187991006
729311000006110	Malignant neoplasm of eyeball excluding conjunctiva, cornea, retina and choroid	188261005
729321000006119	Malignant neoplasm of nasal cavities, middle ear and accessory sinuses	187828007
729331000006116	Malignant neoplasm of bone, connective tissue, skin and breast	271467005
729341000006114	Malig neop of bone, connective tissue, skin and breast NOS	271467005
729351000006111	Malig neop of bone, connective tissue, skin and breast OS	271467005
729361000006113	Malig neop of connective and soft tissue head, face and neck	302816009
729371000006118	Malig neop of connective and soft tissue of abdomen NOS	188015001
729381000006115	Malignant neoplasm of connective and soft tissue of abdominal wall	188016000
729391000006117	Malignant neoplasm of connective and soft tissue of great toe	188006008
729401000006115	Malignant neoplasm of connective and soft tissue of hip and lower limb	187999008
729411000006117	Malignant neoplasm of connective and soft tissue of inguinal region	188021002
729421000006113	Malignant neoplasm of connective and soft tissue of lower leg	188003000
729431000006111	Malig neop of connective and soft tissue of pelvis NOS	363366009
729441000006118	Malig neop of connective and soft tissue of thorax NOS	363365008
729451000006116	Malignant neoplasm of connective and soft tissue of thigh and upper leg	188001003
729461000006119	Malignant neoplasm of connective and soft tissues of trunk	1090891000000103
729471000006114	Malig neop of endocrine gland or related structure NOS	371982006
729481000006112	Malignant tumour of urinary system	419052002
729491000006110	Malig neop of other and unspecified female genital organs	363514001
729501000006119	Malig neop of other and unspecified parts of nervous system	372063002
729511000006116	Malig neop of other endocrine glands and related structures	371982006
729521000006112	Malig neop of other site of heart, thymus and mediastinum	187881004
729531000006110	Malignant neoplasm of thoracic cavity structure	428100006
729541000006117	Malig neop of scapula and long bones of upper arm NOS	187929000
729551000006115	Malig neop of upper respiratory tract, part unspecified	187833006
729561000006118	Malig neop oth/ill-defined sites digestive tract/peritoneum	255077007
729571000006113	Malig neop other site nasal cavity, middle ear and sinuses	187828007
729581000006111	Malig neop other site rectum, rectosigmoid junction and anus	187760008
729591000006114	Malig neop other/ill-defined sites lip, oral cavity, pharynx	271323007
729601000006118	Malig neop other/ill-defined sites resp/intrathoracic organs	428100006
730001000006117	Malignant neoplasm, overlapping lesion of heart, mediastinum and pleura	109384006
730011000006119	Malignant neoplasm of auditory tube, middle ear and mastoid air cells	187833006
730021000006110	Malignant neoplasm of auditory tube, middle ear and mastoid air cells	187833006
730031000006113	Malig neop connective and soft tissue head, face, neck NOS	302816009
730041000006115	Malig neop pituitary gland or craniopharyngeal duct NOS	188339002
730241000006113	Malig neop connective and soft tissue hip and leg NOS	187999008
730251000006110	Malignant neoplasm of connective and soft tissue of popliteal space	188002005
730491000006117	Mal neoplasm/periph nerves+autonomic nervous system,unspc	188321006
731621000006113	Lymphosarcoma cell leukaemia	188725004
732441000006113	Lymph node metastases	94392001
733371000006119	Lung cancer	93880001
745001000006114	Leukaemic reticuloendotheliosis	118613001
745011000006112	Leukaemic reticuloendotheliosis	118613001
755631000006110	Kaposi's sarcoma of multiple organs	109392002
755671000006113	Kaposi's sarcoma, unspecified	109385007
763421000006116	Follicular lymphoma NOS	308121000
819461000006112	Heilmeyer - Schoner disease	109992005
825711000006111	HIV dis reslt/oth mal neopl/lymph,h'matopoetc+reltd tissu	86406008
825841000006115	HIV disease resulting in unspecified malignant neoplasm	86406008
826121000006114	Hodgkin's disease NOS	118599009
826261000006110	Hodgkin's disease, lymphocytic-histiocytic predominance	118607005
841331000006115	Cancer of bowel	363508008
853821000006110	Rodent ulcer	853821000006106
879681000006114	Lip carcinoma	269515006
879691000006112	Tongue carcinoma	363375006
880261000006119	Carcinoma breast	372064008
880351000006110	Carcinoma genital organs	271468000
880381000006119	Carcinoma body of uterus	371972005
880461000006118	Carcinoma bladder	399326009
880551000006119	Other primary carcinomas	543351000000104
880781000006115	Lymphatic tissue carcinoma	269475001
881221000006115	Hydatidiform mole - malignant	18799007
881281000006116	Malignant neoplasm NOS	86049000
881291000006118	Malignant neoplasms	86049000
881301000006117	Metastatic Carcinoma	463191000000105
980021000006113	Malignant neoplasm of endometrium	188192002

982801000006112	Recurrence of cancer confirmed	982801000006108
988771000006113	Lymphatic tissue carcinoma	539691000000104
988801000006110	Malignant neoplasm NOS	640551000000107
1591211000006111	Hairy cell leukaemia	118613001
1653961000006118	Multiple self-healing epithelioma of Ferguson-Smith	1653961000006102
1668101000000112	Diffuse non-Hodgkin's large cell lymphoma	109969005
1694771000006118	Malignant melanoma of the cilary body	1694771000006102
1694781000006115	Malignant melanoma of choroid	255021005
1697891000006116	Malignant melanoma of iris	255012009
1728161000006116	Hereditary nonpolyposis colon cancer	1728161000006100
1773111000006111	Primary malignant neoplasm of lung	93880001
1773121000006115	Small cell lung cancer	254632001
1773131000006117	Non-small cell lung cancer	254637007
1785811000006114	Fibrous mesothelioma, malignant	54443001
1803771000006112	Local recurrence of malignant tumour of urinary bladder	314968009
1803781000006110	Local recurrence of malignant tumour of breast	314955001
1806421000006110	Malignant neoplasm of skin	372130007
1814931000006110	HIV disease resulting in Kaposi sarcoma	420524008
1815011000006116	Secondary malignant neoplasm of liver and intrahepatic bile duct	813671000000107
1815031000006110	Hodgkin lymphoma	118599009
1815051000006115	Nodular sclerosis classical Hodgkin lymphoma	118608000
1815061000006118	Mixed cellularity classical Hodgkin lymphoma	118609008
1815091000006114	Other classical Hodgkin lymphoma	118599009
1815151000006116	Follicular lymphoma	308121000
1815161000006119	Follicular lymphoma grade 1	847481000000109
1815171000006114	Follicular lymphoma grade 2	847631000000107
1815181000006112	Follicular lymphoma grade 3	847651000000100
1815231000006117	Other types of follicular lymphoma	308121000
1815251000006112	Sarcoma of dendritic cells	446643000
1815331000006113	Cutaneous T-cell lymphoma	400122007
1815391000006112	Subcutaneous panniculitic T-cell lymphoma	404133000
1815431000006118	Other mature T/NK-cell lymphoma	277613000
1815451000006113	Nonfollicular lymphoma	109962001
1815491000006119	Other non-follicular lymphoma	118601006
1870401000006113	Juvenile myelomonocytic leukaemia	128832006
1877231000006117	Cancer and/or significant invasive tumors	1877231000006101
1959991000006111	[M]Metaplastic carcinoma	1959991000006107
1960021000006111	[M]Porocarcinoma	1960021000006107
2124491000000113	Follicular lymphoma grade 3a	847691000000108
2124511000000117	Follicular lymphoma grade 3b	847701000000108
2168681000000113	Chronic lymphocytic leukaemia of B-cell type	277473004
2168691000000110	Lymphoblastic (diffuse) lymphoma	109965004
2168841000000118	Waldenstrom macroglobulinaemia	190818004
2168861000000117	Non-follicular lymphoma	118601006
2233381000000115	Clinical stage A chronic lymphocytic leukaemia	863741000000108
2233421000000112	Clinical stage B chronic lymphocytic leukaemia	863761000000109
2233461000000116	Clinical stage C chronic lymphocytic leukaemia	863781000000100
2438511000000112	Malignant melanoma stage IA	956331000000107
2438551000000111	Malignant melanoma stage IB	956351000000100
2438591000000115	Malignant melanoma stage IIA	956371000000109
2438631000000115	Malignant melanoma stage IIB	956391000000108
2438671000000118	Malignant melanoma stage IIC	956411000000108
2438711000000117	Malignant melanoma stage IIIA	956431000000100
2438751000000118	Malignant melanoma stage IIIB	956451000000107
2438791000000114	Malignant melanoma stage IIIC	956471000000103
2438871000000117	Malignant melanoma stage IV M1a	956511000000107
2438911000000115	Malignant melanoma stage IV M1b	956531000000104
2438951000000116	Malignant melanoma stage IV M1c	956551000000106
2511731000006112	Angioimmunoblastic T-cell lymphoma	835009
2519761000006119	Rodent ulcer	1338007
2529491000006110	Malignant lymphoma, non-Hodgkin	1929004
2532031000006112	MM - Malignant melanoma	2092003
2534351000006110	Wolffian duct carcinoma	2221008
2549571000006110	Peripheral T-cell lymphoma	3172003
2572791000006111	Tubular carcinoma	4631006
2583521000006115	Follicular carcinoma	5257006
2599111000006117	Neoplasm, malignant, uncertain whether primary or metastatic	6219000
2606061000006118	Metatypical carcinoma	6641007
2611781000006115	Carcinomatosis	7010000
2614621000000117	Lymphoedema following breast cancer	1035841000000108
2616201000006111	Lymphoepithelial carcinoma	7300000

2629941000006115	Mixed tumour, malignant	8145008
2653191000006112	Epithelial-myoepithelial carcinoma	9618003
2660481000006118	Malignant osteoclastoma	10069009
2663661000006116	Epidermoid carcinoma - spindle cell	10288008
2697401000006114	Papillary urothelial carcinoma	12400006
2730231000006116	Hodgkin lymphoma	14537002
2730241000006114	Hodgkin's disease	14537002
2731021000000110	Secondary malignant neoplasm of paratracheal lymph nodes	1090081000000106
2731321000000112	Malignant neoplasm of respiratory tract	1090231000000103
2731421000000116	Malignant neoplasm of genital labia	1090281000000104
2732801000000115	Malignant neoplasm of soft tissues of lower limb	1090961000000103
2732861000000116	Malignant neoplasm of soft tissues of lower leg	1090991000000109
2734791000000113	Cutaneous follicle centre lymphoma	1091861000000100
2741741000006112	Neuroleptic malignant syndrome	15244003
2747671000006116	Hepatocellular carcinoma, fibrolamellar	15619004
2753081000006116	Thymoma, malignant	15949004
2769471000006119	Acral lentiginous melanoma, malignant	16974005
2773871000006111	Carcinoma ex pleomorphic adenoma	17264009
2773881000006114	Carcinoma in pleomorphic adenoma	17264009
2782281000006111	Acute myeloblastic leukaemia	17788007
2787521000006113	Malignant endometrioid adenofibroma	18105004
2799271000006115	Malignant struma ovarii	18854008
2806901000006114	Malignant lymphoma, lymphoplasmacytic	19340000
2808981000006118	Teratoma, malignant	19467007
2815771000006111	Malignant peripheral nerve sheath tumour	19897006
2845381000006118	Osteosarcoma	21708004
2848541000006110	Malignant teratoma, intermediate	21912003
2848551000006112	Malignant teratoma - intermediate	21912003
2849461000006115	Papillary carcinoma, follicular variant	21968007
2861551000006112	Carcinoma with apocrine metaplasia	22694002
2868381000006115	Sarcomatoid carcinoma	23109009
2902551000006114	Carcinoma - intestinal type	25190001
2905761000006112	Hepatocellular carcinoma	25370001
2930521000006112	Intraosseous carcinoma	26888009
2950311000006114	Cutaneous T-cell lymphoma	28054005
2952081000006118	Follicular carcinoma - well differentiated	28173006
2964701000006114	Squamous carcinoma	28899001
2965511000006118	Acute promyelocytic leukaemia	28950004
2985501000006118	Cribriform carcinoma	30156004
2989331000006118	Mesothelioma, biphasic, malignant	30383009
2999011000006111	Acute myelomonocytic leukaemia	30962008
3023761000006117	Malignant extra-adrenal paraganglioma	32512003
3030841000006117	Inflammatory carcinoma	32968003
3033901000006117	Malignant haemangioendothelioma	33176006
3067781000006111	Infiltrating duct and lobular carcinoma	35232005
3067801000006110	Lobular and ductal carcinoma	35232005
3068691000006115	Myeloid sarcoma	35287006
3108791000006116	Myeloid leukaemia	37810007
3109051000006115	Myelomonocytic leukaemia	37810007
3142071000006112	Malignant melanoma, regressing	39896009
3180131000006117	Malignant Brenner tumour	42194009
3216591000006111	Malignant melanoma in Hutchinson's melanotic freckle	44474009
3221981000006119	Choriocarcinoma	44769000
3254991000006117	Malignant lymphoma, large B-cell, diffuse	46732000
3255181000006119	Diffuse large B-cell lymphoma	46732000
3255631000006111	International Federation of Gynecology and Obstetrics endometrial cancer stage I A	46756001
3285791000006111	Malignant carcinoid tumour - argentaffin	48554007
3312281000006117	Malignant mastocytosis	50150000
3313771000006119	International Federation of Gynecology and Obstetrics endometrial cancer stage I	50237009
3327961000006112	Chronic lymphatic leukaemia	51092000
3338911000006110	Desmoplastic melanoma, malignant	51757004
3338941000006114	Malignant neurotropic melanoma	51757004
3377951000006114	Epithelioid haemangioendothelioma, malignant	54124005
3388331000006110	Sebaceous carcinoma	54734006
3395251000006115	Follicular lymphoma	55150002
3398781000006111	Malignant astrocytoma	55353007
3408441000006115	Multiple myeloma	55921005
3435751000006114	Oncocytic carcinoma	57596004
3446351000006115	Anaplastic carcinoma	58248003
3508601000006114	Malignant mesothelioma	62064005

3519261000006119	Nonencapsulated sclerosing carcinoma	62681000
3530471000006114	Chronic myeloid leukaemia	63364005
3540651000006115	Adnexal carcinoma	64000002
3550221000006113	Malignant lymphoma, small lymphocytic	64575004
3561311000006114	Epithelioid mesothelioma, malignant	65278006
3567381000006110	Malignant teratoma, trophoblastic	65646006
3567391000006113	Trophoblastic malignant teratoma	65646006
3641071000006116	Cholangiocellular carcinoma	70179006
3659761000006112	Epithelioma, malignant	71298006
3674201000006117	Follicular carcinoma, moderately differentiated	72174007
3674221000006110	Follicular carcinoma - moderately differentiated	72174007
3674231000006113	Follicular carcinoma - trabecular	72174007
3711491000006112	Glioma, malignant, no ICD-O subtype	74532006
3713291000006112	Mantle cell lymphoma	74654000
3759331000006111	Neurilemoma, malignant	77418004
3789351000006112	Secondary carcinoma	79282002
3810361000006116	Acute panmyelosis	80570006
3817091000006110	International Federation of Gynecology and Obstetrics endometrial cancer stage III	80975007
3845561000006111	Ductal carcinoma	82711006
3852661000006118	Malignant rhabdoid tumour	83118000
3855431000006116	Malignant teratoma, undifferentiated	83292005
3865781000006119	Malignant tumour, giant cell type	83950009
3865801000006115	Malignant tumour - giant cell type	83950009
3877171000006117	Malignant haemangiopericytoma	84664004
3898741000006115	Neoplasm, malignant (primary)	86049000
3898801000006113	Tumour, malignant	86049000
3898831000006117	Malignant neoplasm	86049000
3928771000006112	Malignant phyllodes tumour	87913009
3936561000006112	Tumour cells, malignant	88400008
3944831000006111	Malignant tumor, spindle cell type	88897007
3944861000006119	Malignant tumour - spindle cell type	88897007
3958791000006112	Infiltrating lobular carcinoma	89740008
3974521000006114	Serous carcinoma	90725004
4016391000006114	Malignant lymphoma of extranodal AND/OR solid organ site	93199007
4016591000006117	Malignant melanoma of skin of abdomen	93210001
4016601000006113	Malignant melanoma of skin of ankle	93211002
4016611000006111	Malignant melanoma of skin of axilla	93213004
4016621000006115	Malignant melanoma of skin of back	93214005
4016631000006117	Malignant melanoma of skin of breast	93215006
4016651000006112	Malignant melanoma of skin of cheek	93217003
4016671000006119	Malignant melanoma of skin of chest	93218008
4016701000006118	Malignant melanoma of skin of elbow	93221005
4016741000006116	Malignant melanoma of skin of eyelid	93224002
4016771000006112	Malignant melanoma of skin of foot	93227009
4016781000006110	Malignant melanoma of skin of forearm	93228004
4016791000006113	Malignant melanoma of skin of forehead	93229007
4016801000006114	Malignant melanoma of skin of groin	93230002
4024231000006119	Malignant melanoma of skin of hand	93636004
4024251000006114	Malignant melanoma of skin of knee	93638003
4024271000006116	Malignant melanoma of skin of lower limb	93641007
4024281000006118	Malignant melanoma of skin of neck	93642000
4024291000006115	Malignant melanoma of skin of nose	93643005
4024331000006110	Malignant melanoma of skin of scalp	93646002
4024341000006117	Malignant melanoma of skin of shoulder	93647006
4024361000006118	Malignant melanoma of skin of thigh	93649009
4024371000006113	Malignant melanoma of skin of toe	93650009
4024401000006111	Malignant melanoma of skin of upper limb	93653006
4024411000006114	Malignant melanoma of skin of wrist	93654000
4024441000006113	Cutaneous malignant melanoma	93655004
4024481000006119	Primary malignant neoplasm of accessory sinus	93659005
4024501000006112	Primary malignant neoplasm of acromion	93661001
4024541000006114	Primary malignant neoplasm of skin with adnexal differentiation	93663003
4024551000006111	Primary malignant neoplasm of adrenal gland	93665005
4024561000006113	Primary malignant neoplasm of alveolar ridge mucosa	93667002
4024571000006118	Primary malignant neoplasm of anal canal	93669004
4024591000006117	Primary malignant neoplasm of anterior mediastinum	93671004
4024621000006115	Primary malignant neoplasm of anus	93676009
4024651000006112	Primary malignant neoplasm of appendix	93679002
4024661000006114	Appendiceal cancer	93679002
4024671000006119	Primary malignant neoplasm of areola of female breast	93680004
4024711000006115	Primary malignant neoplasm of upper arm	93682007

4024721000006111	Primary malignant neoplasm of ascending colon	93683002
4024731000006114	Primary malignant neoplasm of axilla	93684008
4024741000006116	Primary malignant neoplasm of back	93686005
4024751000006119	Primary malignant neoplasm of base of tongue	93687001
4024761000006117	Primary malignant neoplasm of bladder	93689003
4025001000006115	Primary malignant neoplasm of body of pancreas	93715005
4025011000006117	Primary malignant neoplasm of body of penis	93716006
4025021000006113	Primary malignant neoplasm of body of stomach	93717002
4025031000006111	Primary malignant neoplasm of bone marrow	93720005
4025061000006119	Primary malignant neoplasm of bone of skull	93723007
4025081000006112	Primary malignant neoplasm of bone	93725000
4025091000006110	Primary malignant neoplasm of brain stem	93726004
4025101000006116	Primary malignant neoplasm of brain	93727008
4025131000006112	Primary malignant neoplasm of bronchus of left lower lobe	93729006
4025141000006119	Primary malignant neoplasm of bronchus of left upper lobe	93730001
4025151000006117	Primary malignant neoplasm of bronchus of right lower lobe	93731002
4025181000006113	Primary malignant neoplasm of bronchus	93734005
4025211000006112	Primary malignant neoplasm of cardia of stomach	93738008
4025221000006116	Primary malignant neoplasm of carina	93739000
4025271000006115	Primary malignant neoplasm of central nervous system	93744007
4025281000006117	Primary malignant neoplasm of central portion of female breast	93745008
4025291000006119	Primary malignant neoplasm of cerebellum	93746009
4025321000006111	Primary malignant neoplasm of cerebral ventricle	93748005
4025331000006114	Primary malignant neoplasm of cerebrum	93749002
4025351000006119	Primary malignant neoplasm of cheek	93753000
4025391000006113	Primary malignant neoplasm of ciliary body (primary)	93756008
4025411000006113	Primary malignant neoplasm of clavicle	93757004
4025461000006111	Primary malignant neoplasm of colon	93761005
4025471000006116	Primary malignant neoplasm of common bile duct	93763008
4025491000006115	Primary malignant neoplasm of conjunctiva (primary)	93764002
4025571000006117	Primary malignant neoplasm of cystic duct	93770008
4025591000006116	Primary malignant neoplasm of descending colon	93771007
4025601000006112	Primary malignant neoplasm of diaphragm	93772000
4025631000006116	Primary malignant neoplasm of duodenum	93775003
4025661000006113	Primary malignant neoplasm of endocardium	93778001
4025691000006117	Primary malignant neoplasm of endometrium	93781006
4025701000006117	Cancer of endometrium	93781006
4025741000006115	Primary malignant neoplasm of epiglottis	93784003
4025751000006118	Primary malignant neoplasm of ethmoid bone	93786001
4025771000006111	Primary malignant neoplasm of ethmoidal sinus	93787005
4025801000006113	Primary malignant neoplasm of face	93792007
4025821000006115	Primary malignant neoplasm of female breast	93796005
4025831000006117	Primary malignant neoplasm of female genital organ	93797001
4025841000006110	Primary malignant neoplasm of femur	93798006
4025861000006114	Primary malignant neoplasm of fibula	93799003
4025901000006119	Primary malignant neoplasm of floor of mouth	93802007
4025911000006116	Primary malignant neoplasm of foot	93803002
4025931000006110	Primary malignant neoplasm of frontal bone	93806005
4025951000006115	Primary malignant neoplasm of frontal lobe	93807001
4025961000006118	Primary malignant neoplasm of frontal sinus	93808006
4025981000006111	Primary malignant neoplasm of gingival mucosa	93812000
4025991000006114	Primary malignant neoplasm of glomus jugulare	93814004
4026021000006117	Primary malignant neoplasm of glottis	93816002
4026051000006114	Primary malignant neoplasm of greater curvature of stomach	93818001
4026071000006116	Primary malignant neoplasm of hand	93821004
4026081000006118	Primary malignant neoplasm of head	93824007
4026091000006115	Primary malignant neoplasm of heart	93825008
4026101000006114	Primary malignant neoplasm of hepatic flexure of colon	93826009
4026111000006112	Primary malignant neoplasm of hilus of lung	93827000
4026161000006110	Primary malignant neoplasm of hypopharynx	93831006
4026171000006115	Primary malignant neoplasm of ileum	93832004
4026181000006117	Primary malignant neoplasm of ilium	93833009
4026221000006114	Primary malignant neoplasm of inner aspect of lower lip	93836001
4026241000006119	Primary malignant neoplasm of intra-abdominal organs	93839008
4026301000006112	Primary malignant neoplasm of jaw	93845000
4026311000006110	Primary malignant neoplasm of jejunum	93846004
4026331000006116	Primary malignant neoplasm of kidney	93849006
4026351000006111	Primary malignant neoplasm of labia minora	93851005
4026361000006113	Primary malignant neoplasm of large intestine	93854002
4026421000006113	Primary malignant neoplasm of lower lobe of left lung	93864006
4026441000006118	Primary malignant neoplasm of left upper lobe of lung	93865007

4026461000006119	Primary malignant neoplasm of lesser curvature of stomach	93867004
4026471000006114	Primary malignant neoplasm of lingual tonsil	93868009
4026491000006110	CA - Liver cancer	93870000
4026501000006119	Liver cancer	93870000
4026511000006116	Malignant tumor of liver	93870000
4026561000006118	Primary malignant neoplasm of lower limb	93875005
4026631000006114	Primary malignant neoplasm of major salivary gland	93883004
4026641000006116	Primary malignant neoplasm of male breast	93884005
4026651000006119	Primary malignant neoplasm of male genital organ	93885006
4026661000006117	Primary malignant neoplasm of mandible	93886007
4026681000006110	Primary malignant neoplasm of maxilla	93888008
4026701000006113	Primary malignant neoplasm of maxillary sinus	93889000
4026731000006117	Primary malignant neoplasm of mediastinum	93891008
4026761000006114	Primary malignant neoplasm of middle ear	93894000
4026841000006115	Primary malignant neoplasm of muscle of lower limb	93905005
4026921000006118	Primary malignant neoplasm of muscle	93913006
4026941000006113	Primary malignant neoplasm of myometrium	93915004
4026951000006110	Primary malignant neoplasm of nasal bone	93916003
4026971000006117	Primary malignant neoplasm of nasal cavity	93917007
4027001000006112	Primary malignant neoplasm of neck	93922007
4027021000006119	Primary malignant neoplasm of nipple of female breast	93924008
4027111000006114	Primary malignant neoplasm of olfactory nerve	93930008
4027131000006115	Primary malignant neoplasm of orbit	93932000
4027141000006113	Primary malignant neoplasm of oropharynx	93933005
4027151000006110	Primary malignant neoplasm of ovary	93934004
4027171000006117	Primary malignant neoplasm of pancreatic duct	93939009
4027211000006115	Primary malignant neoplasm of parathyroid gland	93943008
4027261000006117	Primary malignant neoplasm of parietal peritoneum	93947009
4027321000006116	Primary malignant neoplasm of pelvic peritoneum	93952004
4027331000006118	Primary malignant neoplasm of pelvis	93953009
4027361000006110	Primary malignant neoplasm of perianal skin	93956001
4027381000006117	Primary malignant neoplasm of pericardium	93957005
4027431000006113	Primary malignant neoplasm of pharynx	93961004
4027441000006115	Primary malignant neoplasm of pineal gland	93962006
4027461000006116	Primary malignant neoplasm of pituitary gland	93964007
4027471000006111	Primary malignant neoplasm of pleura	93966009
4027491000006112	Primary malignant neoplasm of posterior hypopharyngeal wall	93968005
4027531000006112	Primary malignant neoplasm of presacral region	93973004
4027551000006117	Primary malignant neoplasm of prostate	93974005
4027561000006115	Primary malignant neoplasm of pubis	93975006
4027581000006113	Primary malignant neoplasm of pyloric antrum	93976007
4027591000006111	Primary malignant neoplasm of pylorus	93977003
4027601000006115	Primary malignant neoplasm of pyriform sinus	93978008
4027611000006117	Primary malignant neoplasm of radius	93979000
4027631000006111	Primary malignant neoplasm of rectosigmoid junction	93980002
4027671000006114	Primary malignant neoplasm of rectum	93984006
4027681000006112	Primary malignant neoplasm of renal pelvis	93985007
4027701000006110	Primary malignant neoplasm of retina (primary)	93987004
4027711000006113	Primary malignant neoplasm of retina, primary	93987004
4027721000006117	Primary malignant neoplasm of retromolar area	93989001
4027731000006119	Primary malignant neoplasm of rib	93990005
4027751000006114	Primary malignant neoplasm of right lower lobe of lung	93991009
4027761000006111	Primary malignant neoplasm of right middle lobe of lung	93992002
4027771000006116	Primary malignant neoplasm of upper lobe of right lung	93993007
4027801000006119	Primary malignant neoplasm of sacrococcygeal region	93995000
4027831000006110	Primary malignant neoplasm of scapula	93997008
4027871000006113	Primary malignant neoplasm of sebaceous gland	94000008
4027901000006113	Primary malignant neoplasm of septum of nose	94002000
4027931000006117	Primary malignant neoplasm of shoulder	94005003
4027941000006110	Primary malignant neoplasm of sigmoid colon	94006002
4027961000006114	Primary malignant neoplasm of skin of ankle	94008001
4027991000006118	Primary malignant neoplasm of skin of back	94011000
4028011000006114	Primary malignant neoplasm of skin of breast	94012007
4028031000006115	Primary malignant neoplasm of skin of buttock	94013002
4028071000006117	Primary malignant neoplasm of skin of chin	94016005
4028121000006117	Primary malignant neoplasm of skin of eyebrow	94020009
4028151000006114	Primary malignant neoplasm of skin of face	94022001
4028161000006111	Primary malignant neoplasm of skin of finger	94023006
4028181000006118	Primary malignant neoplasm of skin of foot	94024000
4028211000006119	Primary malignant neoplasm of skin of forehead	94026003
4028231000006113	Primary malignant neoplasm of skin of groin	94027007

4028251000006118	Primary malignant neoplasm of skin of hand	94028002
4028291000006112	Primary malignant neoplasm of skin of knee	94030000
4028311000006111	Primary malignant neoplasm of skin of lip	94032008
4028341000006110	Primary malignant neoplasm of skin of neck	94034009
4028371000006119	Primary malignant neoplasm of skin of perineum	94036006
4028411000006118	Primary malignant neoplasm of skin of shoulder	94039004
4028441000006119	Primary malignant neoplasm of skin of thigh	94041003
4028461000006115	Primary malignant neoplasm of skin of toe	94042005
4028551000006116	Primary malignant neoplasm of small intestine	94048009
4028561000006119	Primary malignant neoplasm of soft palate	94049001
4028591000006110	Primary malignant neoplasm of soft tissues of buttock	94052009
4028641000006117	Primary malignant neoplasm of soft tissues of lower limb	94057003
4028741000006111	Primary malignant neoplasm of spinal cord	94068003
4028761000006110	Primary malignant neoplasm of spleen	94071006
4028771000006115	Primary malignant neoplasm of splenic flexure of colon	94072004
4028781000006117	Primary malignant neoplasm of sternum	94073009
4028801000006118	Primary malignant neoplasm of subglottis	94075002
4028841000006116	Primary malignant neoplasm of supraclavicular region	94079008
4028851000006119	Primary malignant neoplasm of supraglottis	94080006
4028861000006117	Primary malignant neoplasm of sweat gland	94081005
4028881000006110	Primary malignant neoplasm of tail of pancreas	94082003
4028921000006119	Primary malignant neoplasm of temporal bone	94085001
4028941000006114	Primary malignant neoplasm of temporal lobe	94086000
4028951000006111	Primary malignant neoplasm of testis	94087009
4028971000006118	Primary malignant neoplasm of omentum	94090003
4028981000006115	Primary malignant neoplasm of retroperitoneum	94092006
4028991000006117	Primary malignant neoplasm of thigh	94093001
4029011000006119	Primary malignant neoplasm of thymus	94096009
4029031000006113	Primary malignant neoplasm of thyroid gland	94098005
4029041000006115	Primary malignant neoplasm of tibia	94099002
4029061000006116	Primary malignant neoplasm of tongue	94101009
4029071000006111	Primary malignant neoplasm of tonsillar fossa	94102002
4029081000006114	Primary malignant neoplasm of tonsillar pillar	94103007
4029091000006112	Primary malignant neoplasm of trachea	94104001
4029101000006118	Primary malignant neoplasm of transverse colon	94105000
4029131000006114	Primary malignant neoplasm of trigone of urinary bladder	94109006
4029151000006119	Primary malignant neoplasm of trunk	94111002
4029181000006110	Primary malignant neoplasm of undescended testis	94113004
4029201000006111	Primary malignant neoplasm of upper limb	94116007
4029211000006114	Primary malignant neoplasm of upper outer quadrant of female breast	94117003
4029221000006118	Primary malignant neoplasm of upper respiratory tract	94118008
4029241000006113	Primary malignant neoplasm of ureter	94121005
4029251000006110	Primary malignant neoplasm of ureteric orifice of urinary bladder	94122003
4029261000006112	Primary malignant neoplasm of urethra	94123008
4029271000006117	Primary malignant neoplasm of urinary bladder neck	94124002
4029281000006119	Primary malignant neoplasm of urinary system	94125001
4029291000006116	Primary malignant neoplasm of uterine adnexa	94126000
4029301000006115	Primary malignant neoplasm of uveal tract (primary)	94128004
4029321000006113	Primary malignant neoplasm of uvula	94129007
4029361000006119	Primary malignant neoplasm of ventral surface of tongue	94134006
4029441000006114	Primary malignant neoplasm of vulva	94143002
4029471000006118	Primary malignant neoplasm of zygomatic bone	94145009
4029721000006112	Metastatic malignant neoplasm to adenoid	94158005
4029811000006113	Cancer metastatic to adrenal gland	94161006
4029861000006111	Secondary malignant neoplasm of ampulla of Vater	94164003
4030061000006111	Secondary malignant neoplasm of appendix	94175004
4030121000006116	Secondary malignant neoplasm of ascending colon	94179005
4030131000006118	Metastatic malignant neoplasm to ascending colon	94179005
4030141000006111	Secondary malignant neoplasm of axilla	94180008
4030151000006113	Metastatic malignant neoplasm to axilla	94180008
4030171000006115	Metastatic malignant neoplasm to axillary lymph nodes	94181007
4030201000006116	Secondary malignant neoplasm of back	94183005
4030241000006119	Secondary malignant neoplasm of biliary tract	94185003
4030271000006110	Metastatic malignant neoplasm to bladder	94186002
4030311000006110	Cancer metastatic to urinary bladder	94186002
4030761000006114	Secondary malignant neoplasm of blood vessel	94211009
4030781000006116	Secondary malignant neoplasm of body of pancreas	94212002
4030881000006114	Secondary malignant neoplasm of bone marrow	94217008
4030941000006113	Secondary malignant neoplasm of bone of lower limb	94219006
4030961000006112	Secondary malignant neoplasm of bone of skull	94220000
4030971000006117	Metastatic malignant neoplasm to bone of skull	94220000

4030981000006119	Secondary malignant neoplasm of bone of upper limb	94221001
4031001000006115	Secondary malignant neoplasm of bone	94222008
4031011000006117	Metastatic malignant neoplasm to bone	94222008
4031041000006118	Secondary cancer of bone	94222008
4031081000006112	Cancer metastatic to bone	94222008
4031141000006119	Metastatic malignant neoplasm to brain	94225005
4031151000006117	Secondary cancer of brain	94225005
4031171000006110	Cancer metastatic to brain	94225005
4031221000006116	Metastatic malignant neoplasm to hilar lymph nodes	94227002
4031341000006116	Secondary malignant neoplasm of bronchus	94233006
4031411000006113	Secondary malignant neoplasm of caecum	94235004
4031441000006112	Metastatic malignant neoplasm to caecum	94235004
4031571000006117	Secondary malignant neoplasm of cauda equina	94242004
4031591000006116	Secondary malignant neoplasm of central nervous system	94243009
4031641000006114	Secondary malignant neoplasm of cerebellum	94245002
4031661000006113	Secondary malignant neoplasm of cerebral meninges	94246001
4031671000006118	Metastatic malignant neoplasm to cerebral meninges	94246001
4031711000006119	Secondary malignant neoplasm of cerebrum	94248000
4031721000006110	Metastatic malignant neoplasm to cerebrum	94248000
4031781000006114	Secondary malignant neoplasm of cervical vertebral column	94250008
4031801000006113	Secondary malignant neoplasm of cheek	94252000
4031821000006115	Secondary malignant neoplasm of chest wall	94253005
4031851000006112	Secondary malignant neoplasm of choroid	94254004
4031991000006114	Secondary malignant neoplasm of coccyx	94259009
4032021000006118	Metastatic malignant neoplasm to colon	94260004
4032051000006110	Secondary malignant neoplasm of common bile duct	94262007
4032091000006116	Secondary malignant neoplasm of soft tissues	94264008
4032251000006118	Secondary malignant neoplasm of descending colon	94271003
4032311000006111	Secondary malignant neoplasm of thoracic vertebral column	94274006
4032461000006115	Secondary malignant neoplasm of endocrine gland	94280003
4032491000006111	Secondary malignant neoplasm of endometrium	94281004
4032501000006115	Metastatic malignant neoplasm to endometrium	94281004
4032601000006119	Secondary malignant neoplasm of epitrochlear lymph nodes	94285008
4032621000006112	Secondary malignant neoplasm of oesophagus	94286009
4032651000006115	Metastatic malignant neoplasm to oesophagus	94286009
4032771000006115	Secondary malignant neoplasm of eye	94292003
4032901000006112	Secondary malignant neoplasm of female breast	94297009
4032911000006110	Metastatic malignant neoplasm to female breast	94297009
4032931000006116	Secondary malignant deposit to breast	94297009
4032951000006111	Secondary malignant neoplasm of female genital organ	94298004
4033001000006116	Secondary malignant neoplasm of femoral lymph nodes	94299007
4033021000006114	Secondary malignant neoplasm of femur	94300004
4033031000006112	Metastatic malignant neoplasm to femur	94300004
4033101000006117	Secondary malignant neoplasm of floor of mouth	94304008
4033221000006117	Secondary malignant neoplasm of frontal lobe	94309003
4033231000006119	Metastatic malignant neoplasm to frontal lobe	94309003
4033281000006118	Secondary malignant neoplasm of gallbladder	94312000
4033301000006119	Cancer metastatic to gallbladder	94312000
4033571000006118	Secondary malignant neoplasm of head of pancreas	94325008
4033591000006117	Secondary malignant neoplasm of head	94326009
4033671000006119	Secondary malignant neoplasm of hepatic flexure of colon	94328005
4033691000006118	Secondary malignant neoplasm of hilus of lung	94329002
4033851000006113	Metastatic malignant neoplasm to iliac lymph nodes	94336001
4033871000006115	Secondary malignant neoplasm of ilium	94337005
4033911000006117	Secondary malignant neoplasm of inguinal lymph nodes	94339008
4034051000006119	Secondary malignant neoplasm of intestinal lymph nodes	94345000
4034081000006110	Secondary malignant neoplasm of intestinal tract	94346004
4034101000006119	Secondary malignant neoplasm of intra-abdominal lymph nodes	94347008
4034131000006110	Secondary malignant neoplasm of intra-abdominal organs	94348003
4034191000006114	Secondary malignant neoplasm of intrapelvic lymph nodes	94350006
4034201000006112	Metastatic malignant neoplasm to intrapelvic lymph nodes	94350006
4034221000006119	Secondary malignant neoplasm of intrathoracic lymph nodes	94351005
4034241000006114	Cancer metastatic to intrathoracic lymph nodes	94351005
4034251000006111	Secondary malignant neoplasm of intrathoracic organs	94352003
4034421000006115	Metastatic malignant neoplasm to kidney	94360002
4034461000006114	Secondary renal cancer	94360002
4034481000006116	Secondary cancer of kidney	94360002
4034491000006118	Cancer metastatic to kidney	94360002
4034661000006111	Secondary malignant neoplasm of laryngeal surface of epiglottis	94369001
4034781000006112	Secondary malignant neoplasm of left lower lobe of lung	94375005
4034801000006111	Secondary malignant neoplasm of left upper lobe of lung	94376006

4034811000006114	Metastatic malignant neoplasm to left upper lobe of lung	94376006
4034891000006116	Metastatic malignant neoplasm to liver	94381002
4034901000006117	Liver secondary cancer	94381002
4034951000006118	Cancer metastatic to liver	94381002
4034981000006114	Secondary malignant neoplasm of long bone of upper limb	94383004
4035121000006111	Secondary malignant neoplasm of lumbar vertebral column	94389000
4035131000006114	Metastatic malignant neoplasm to lumbar vertebral column	94389000
4035171000006112	Metastatic malignant neoplasm to lung	94391008
4035181000006110	Secondary malignant tumour of lung	94391008
4035191000006113	Secondary malignant tumor of lung	94391008
4035211000006114	Secondary cancer of lung	94391008
4035221000006118	Cancer metastatic to lung	94391008
4035241000006113	Metastatic malignant neoplasm to lymph node	94392001
4035251000006110	Secondary lymph node cancer	94392001
4035261000006112	Cancer metastatic to lymph nodes	94392001
4035331000006111	Secondary malignant neoplasm of lymph nodes of lower limb	94395004
4035401000006112	Secondary malignant neoplasm of lymph nodes of neck	94397007
4035411000006110	Metastatic malignant neoplasm to lymph nodes of neck	94397007
4035431000006116	Cancer metastatic to neck lymph nodes	94397007
4035481000006115	Secondary malignant neoplasm of main bronchus	94399005
4035501000006113	Secondary malignant neoplasm of major salivary gland	94400003
4035521000006115	Secondary malignant neoplasm of male breast	94401004
4035531000006117	Metastatic malignant neoplasm to male breast	94401004
4035631000006118	Secondary malignant neoplasm of maxilla	94405008
4035651000006113	Secondary malignant neoplasm of maxillary sinus	94406009
4035661000006110	Metastatic malignant neoplasm to maxillary sinus	94406009
4035711000006116	Metastatic malignant neoplasm to mediastinal lymph nodes	94408005
4035841000006112	Secondary malignant neoplasm of metatarsal bone	94412004
4035981000006113	Secondary malignant neoplasm of muscle of abdomen	94418000
4036001000006112	Secondary malignant neoplasm of muscle of buttock	94419008
4036261000006117	Secondary malignant neoplasm of muscle	94432003
4036301000006114	Metastatic malignant neoplasm to myocardium	94433008
4036351000006113	Secondary malignant neoplasm of nasal cavity	94436000
4036451000006118	Secondary malignant neoplasm of neck	94441008
4036461000006116	Metastatic malignant neoplasm to neck	94441008
4036511000006118	Secondary malignant neoplasm of nipple of female breast	94443006
4036621000006113	Secondary malignant neoplasm of occipital lobe	94448002
4036701000006110	Secondary malignant neoplasm of optic nerve	94452002
4036751000006114	Secondary malignant neoplasm of orbit	94453007
4036841000006117	Metastatic malignant neoplasm to ovary	94455000
4036921000006115	Cancer metastatic to ovary	94455000
4037011000006112	Secondary malignant neoplasm of pancreas	94459006
4037021000006116	Metastatic malignant neoplasm to pancreas	94459006
4037061000006110	Cancer metastatic to pancreas	94459006
4037071000006115	Secondary malignant neoplasm of pancreatic duct	94460001
4037081000006117	Metastatic malignant neoplasm to pancreatic duct	94460001
4037091000006119	Secondary malignant neoplasm of para-aortic body	94461002
4037111000006111	Secondary malignant neoplasm of paraganglion	94462009
4037191000006118	Secondary malignant neoplasm of pararectal lymph nodes	94466007
4037281000006112	Secondary malignant neoplasm of parietal bone	94470004
4037291000006110	Metastatic malignant neoplasm to parietal bone	94470004
4037301000006111	Secondary malignant neoplasm of parietal lobe	94471000
4037361000006112	Secondary malignant neoplasm of parotid gland	94474008
4037401000006119	Secondary malignant neoplasm of patella	94476005
4037441000006117	Secondary malignant neoplasm of pelvic bone	94478006
4037451000006115	Metastatic malignant neoplasm to pelvic bone	94478006
4037461000006118	Secondary malignant neoplasm of pelvic peritoneum	94479003
4037481000006111	Secondary malignant neoplasm of pelvis	94480000
4037511000006115	Metastatic malignant neoplasm to penis	94481001
4037651000006118	Secondary malignant neoplasm of phalanx of hand	94487002
4037731000006116	Secondary malignant neoplasm of pituitary gland	94491007
4037851000006117	Cancer metastatic to pleura	94493005
4037911000006113	Secondary malignant neoplasm of posterior hypopharyngeal wall	94496002
4038061000006114	Metastatic malignant neoplasm to prostate	94503003
4038111000006110	Secondary malignant neoplasm of pubis	94504009
4038171000006118	Secondary malignant neoplasm of pyriform sinus	94507002
4038211000006116	Secondary malignant neoplasm of rectosigmoid junction	94509004
4038231000006110	Cancer metastatic to rectosigmoid junction	94509004
4038311000006113	Metastatic malignant neoplasm to rectum	94513006
4038321000006117	Cancer metastatic to rectum	94513006
4038331000006119	Secondary malignant neoplasm of renal pelvis	94514000

4038401000006118	Metastatic malignant neoplasm to retina	94516003
4038471000006112	Secondary malignant neoplasm of retroperitoneal lymph nodes	94519005
4038491000006113	Cancer metastatic to retroperitoneal lymph nodes	94519005
4038501000006117	Secondary malignant neoplasm of retropharyngeal lymph nodes	94520004
4038521000006110	Secondary malignant neoplasm of rib	94521000
4038531000006113	Metastatic malignant neoplasm to rib	94521000
4038541000006115	Secondary malignant neoplasm of right lower lobe of lung	94522007
4038551000006118	Metastatic malignant neoplasm to right lower lobe of lung	94522007
4038581000006114	Secondary malignant neoplasm of right upper lobe of lung	94524008
4038621000006114	Secondary malignant neoplasm of sacrococcygeal region	94526005
4038641000006119	Secondary malignant neoplasm of sacrum	94527001
4038651000006117	Metastatic malignant neoplasm to sacrum	94527001
4038681000006113	Secondary malignant neoplasm of scapula	94529003
4038881000006112	Secondary malignant neoplasm of shoulder	94537006
4038901000006114	Secondary malignant neoplasm of sigmoid colon	94538001
4038911000006112	Metastatic malignant neoplasm to sigmoid colon	94538001
4038961000006110	Secondary malignant neoplasm of skin of axilla	94542003
4038981000006117	Secondary malignant neoplasm of skin of back	94543008
4039011000006116	Metastatic malignant neoplasm to skin of breast	94544002
4039181000006112	Secondary malignant neoplasm of skin of eyelid	94553009
4039301000006117	Secondary malignant neoplasm of skin of groin	94559008
4039431000006118	Metastatic malignant neoplasm to skin of neck	94566009
4039501000006110	Secondary malignant neoplasm of skin of scalp	94570001
4039621000006118	Secondary malignant neoplasm of skin of umbilicus	94576007
4039691000006116	Metastatic malignant neoplasm to skin	94579000
4039721000006114	Malignant infiltration of skin	94579000
4039761000006115	Cancer metastatic to skin	94579000
4039831000006116	Secondary malignant neoplasm of soft tissues of abdomen	94582005
4039951000006119	Secondary malignant neoplasm of soft tissues of inguinal region	94588009
4040231000006113	Secondary malignant neoplasm of spinal cord	94600009
4040241000006115	Metastatic malignant neoplasm to spinal cord	94600009
4040261000006116	Secondary malignant neoplasm of spinal meninges	94601008
4040291000006112	Secondary malignant neoplasm of vertebral column	94602001
4040301000006113	Secondary malignant neoplasm of spine	94602001
4040311000006111	Metastatic malignant neoplasm to spine	94602001
4040321000006115	Metastatic malignant neoplasm to vertebral column	94602001
4040371000006119	CA - Secondary cancer of spine	94602001
4040401000006116	Secondary malignant neoplasm of spleen	94603006
4040411000006118	Metastatic malignant neoplasm to spleen	94603006
4040451000006117	Secondary malignant neoplasm of splenic flexure of colon	94604000
4040471000006110	Secondary malignant neoplasm of sternum	94605004
4040491000006111	Secondary malignant neoplasm of stomach	94606003
4040501000006115	Metastatic malignant neoplasm to stomach	94606003
4040511000006117	Cancer metastatic to stomach	94606003
4040631000006110	Metastatic malignant neoplasm to superficial inguinal lymph nodes	94612008
4040661000006118	Secondary malignant neoplasm of supraclavicular lymph nodes	94614009
4040681000006111	Secondary malignant neoplasm of supraclavicular region	94615005
4040801000006118	Secondary malignant neoplasm of temporal bone	94621009
4040891000006113	Secondary malignant neoplasm of the mesentery	94624001
4040931000006116	Secondary malignant neoplasm of omentum	94626004
4040971000006118	Cancer metastatic to omentum	94626004
4041021000006114	Secondary malignant peritoneal deposit	94627008
4041031000006112	Cancer metastatic to peritoneum	94627008
4041061000006115	Secondary malignant neoplasm of the retroperitoneum	94628003
4041251000006114	Secondary malignant neoplasm of thyroid gland	94634005
4041261000006111	Metastatic malignant neoplasm to thyroid gland	94634005
4041281000006118	Cancer metastatic to thyroid	94634005
4041291000006115	Secondary malignant neoplasm of tibia	94635006
4041421000006118	Secondary malignant neoplasm of trachea	94641004
4041881000006117	Metastatic malignant neoplasm to ureter	94659001
4041921000006113	Secondary malignant neoplasm of ureteric orifice of urinary bladder	94660006
4042111000006114	Secondary malignant neoplasm of uveal tract	94666000
4042161000006112	Metastatic malignant neoplasm to vagina	94668004
4042411000006119	Secondary malignant neoplasm of vocal cord	94679009
4042491000006112	Secondary malignant neoplasm of Waldeyer's ring	94682004
4163081000006112	Primary malignant neoplasm of unspecified site (clinical)	109356001
4163091000006110	Primary malignant neoplasm of unspecified site	109356001
4163271000006115	Primary malignant neoplasm of laryngeal cartilage	109370001
4163371000006112	Malignant mesothelioma	109378008
4163431000006119	Malignant mesothelioma of pericardium	109383000
4163451000006114	Overlapping malignant neoplasm of heart, mediastinum and pleura	109384006

4170271000006116	Overlapping malignant neoplasm of lip	109822001
4170391000006114	Primary malignant neoplasm of salivary gland duct	109828002
4170451000006110	Overlapping malignant neoplasm of oropharynx	109832008
4170561000006113	Overlapping malignant neoplasm of small intestine	109837002
4170611000006111	Overlapping malignant neoplasm of rectum, anus and anal canal	109839004
4170641000006110	Hepatocarcinoma	109841003
4170661000006114	Malignant hepatoma	109841003
4170671000006119	HCC - Hepatocellular carcinoma	109841003
4170701000006118	Intrahepatic bile duct carcinoma	109842005
4170841000006111	Malignant mesothelioma of peritoneum	109853004
4171631000006110	Cancer of the brain, overlapping sites	109912006
4171691000006114	Primary malignant neoplasm of meninges	109915008
4172261000006110	Malignant lymphoma - lymphoblastic	109965004
4172461000006111	Diffuse malignant lymphoma - large cell	109969005
4172471000006116	Malignant lymphoma - large cell cleaved and non-cleaved	109969005
4172591000006116	Follicular lymphoma grade 2	109971005
4309281000006110	Malignant Hodgkin's lymphoma	118599009
4309371000006115	Malignant lymphoma, non-Hodgkin's type	118601006
4309411000006119	Non-Hodgkin lymphoma	118601006
4310011000006116	Burkitt's type malignant lymphoma - undifferentiated	118617000
4377911000006111	Mastocytosis	125541005
4393561000006115	Malignant histiocytic disorder	127070008
4395121000006117	Malignant lymphoma of lymph nodes	127220001
4403031000006114	Meckel's diverticulum	127962001
4409491000006116	Secondary cancer	128462008
4409501000006112	Metastatic malignant disease	128462008
4409561000006113	Metastatic cancer	128462008
4412761000006114	Pituitary carcinoma	128665000
4412911000006114	Papillary microcarcinoma	128674003
4412951000006110	Papillary carcinoma, tall cell	128677005
4413071000006114	Porocarcinoma	128685001
4413391000006113	Metaplastic carcinoma	128705006
4413411000006113	Hepatoid carcinoma	128706007
4414691000006119	GIST, malignant	128756002
4416081000006119	Letterer-Siwe disease	128812005
4416161000006111	Histiocytic sarcoma	128813000
4419201000006112	Myoepithelial carcinoma	128884000
4419511000006112	Microcystic adnexal carcinoma	128896007
4420051000006115	Prolymphocytic leukaemia	128923008
4420081000006111	Mast cell leukaemia	128924002
4745551000006110	Malignant tumour of anterior two-thirds of tongue - dorsal surface	187634003
4745711000006119	Malignant tumour of anterior floor of mouth	187652003
4745741000006115	Malignant tumour of lateral floor of mouth	187653008
4745801000006113	Malignant tumour of upper buccal sulcus	187659007
4745831000006117	Malignant tumour of lower buccal sulcus	187660002
4745931000006110	Malignant tumour of tonsillar pillar	187675005
4746111000006112	Malignant tumour of posterior wall of oropharynx	187688008
4746171000006115	Cancer of nasopharynx	187692001
4746191000006119	CA - Cancer of nasopharynx	187692001
4746261000006115	Malignant tumour of adenoid	187694000
4746321000006119	Malignant tumour of pharyngeal tonsil	187694000
4746331000006116	Malignant tumour of nasopharyngeal tonsil	187694000
4746341000006114	Malignant tumour of pharyngeal recess	187697007
4746461000006119	Malignant tumour of nasopharyngeal soft palate surface	187702003
4746491000006110	Malignant tumour aryepiglottic fold - hypopharyngeal aspect	187708004
4746581000006111	Malignant tumour of cervical part of oesophagus	187722004
4746621000006111	Malignant tumour of thoracic part of oesophagus	187723009
4746661000006117	Malignant tumour of abdominal part of oesophagus	187724003
4746701000006113	Malignant tumour of upper third of oesophagus	187725002
4746761000006114	Malignant tumour of middle third of oesophagus	187726001
4746821000006110	Malignant tumour of lower third of oesophagus	187727005
4746881000006114	Malignant tumour of cardia	187732006
4746951000006110	Malignant neoplasm of gastro-esophageal junction	187734007
4746981000006119	Malignant tumour of pylorus	187736009
4747031000006116	Malignant tumour of pyloric antrum	187740000
4747061000006113	Malignant tumour of fundus of stomach	187741001
4747101000006111	Malignant tumour of body of stomach	187742008
4747141000006113	Malignant tumour of Meckel's diverticulum	187752007
4747311000006112	Malignant tumour of body of pancreas	187791002
4747351000006113	Malignant tumour of tail of pancreas	187792009
4747371000006115	Malignant tumor of tail of pancreas	187792009

4747391000006119	Malignant tumour of pancreatic duct	187793004
4747701000006110	Malignant tumour of nasal vestibule	187831008
4747861000006118	Malignant tumour of glottis	187841006
4747901000006113	Malignant tumour of supraglottis	187842004
4748651000006115	Malignant neoplasm of connective and soft tissue of forearm	187994003
4748911000006110	Malignant neoplasm, overlapping lesion of connective and soft tissue	188026007
4748961000006113	Malignant melanoma of ear and/or external auditory canal	188032002
4749171000006112	Malignant melanoma of forearm	188062008
4749501000006113	Malignant neoplasm of skin of forearm	188122007
4749971000006110	Malignant tumour of ectopic testis	188220005
4750011000006114	Malignant tumour of body of penis	188230001
4750071000006117	Malignant tumour of seminal vesicle	188234005
4750101000006110	Malignant tumour of tunica vaginalis	188235006
4750131000006119	Malignant tumour of trigone of urinary bladder	188239000
4750181000006118	Malignant tumour of vault of bladder	188240003
4750191000006115	Malignant tumour of bladder dome	188240003
4750271000006111	Malignant tumour of bladder neck	188244007
4750281000006114	Malignant tumor of bladder neck	188244007
4750301000006113	Malignant tumour of ureteric orifice	188245008
4750361000006114	Malignant tumour of kidney parenchyma	188250002
4750401000006116	Malignant tumour of renal calyx	188252005
4750411000006118	Malignant neoplasm of renal calyx	188252005
4750431000006112	Malignant tumour of pelviureteric junction	188253000
4750501000006115	Malignant tumour of ciliary body	188263008
4750531000006111	Malignant tumour of iris	188264002
4750601000006119	Malignant tumour of lacrimal gland	188272000
4750711000006112	Malignant tumour of hypothalamus	188286001
4750771000006115	Malignant tumour of choroid plexus	188292007
4751071000006110	Malignant tumour of craniopharyngeal duct	188340000
4751131000006113	Malignant tumour of thorax	188361007
4751171000006111	Malignant tumour of abdomen	188366002
4752991000006118	Diffuse malignant lymphoma - small non-cleaved cell	188674006
4753031000006113	Malignant lymphoma - small cleaved cell	188675007
4753071000006111	Malignant lymphoma - mixed small and large cell	188676008
4757371000006118	Malignant lymphoma, stem cell type	189962004
5028071000006111	Malignant neoplasm of eyelid	231829006
5033571000006110	Malignant otitis externa due to Pseudomonas aeruginosa	232230009
5060881000006113	Malignant vasovagal syndrome	234164004
5083021000006111	Malignant oesophageal stricture	235615004
5083031000006114	Malignant esophageal stricture	235615004
5141001000006116	Polymyositis with malignant disease	239898008
5141031000006112	Dermatomyositis with malignant disease	239901009
5144281000006116	Malignant neoplasm of nasopharyngeal wall	240163000
5168481000006119	Metastatic malignant neoplasm to nasopharynx	241861008
5324131000006119	Malignant squamous tumour	252988009
5324641000006117	Malignant endometrioid tumour	253014007
5324731000006118	Fibrolamellar hepatocellular carcinoma	253018005
5325101000006111	Malignant stromal tumour	253048008
5325451000006118	Malignant pineal germinoma	253079007
5343881000006114	Malignant tumour of tip of tongue	254412006
5344051000006112	Carcinoma of lingual tonsil	254423005
5344111000006110	Carcinoma of anterior part of floor of mouth	254427006
5344271000006113	Carcinoma of hard palate	254434008
5344281000006111	Carcinoma of soft palate	254435009
5344291000006114	Carcinoma of uvula	254436005
5344461000006117	Carcinoma of lower buccal sulcus	254445006
5344641000006119	Carcinoma of retromolar area	254457002
5344701000006111	Malignant tumour of anterior tonsillar pillar	254459004
5344711000006114	Malignant tumour of palatoglossal arch	254459004
5344771000006117	Carcinoma of parotid gland	254462001
5344821000006113	Carcinoma of submandibular gland	254465004
5346011000006116	Malignant tumour of parapharyngeal space	254530002
5346111000006117	Carcinoma of cervical part of oesophagus	254535007
5346251000006112	Carcinoma of middle third of oesophagus	254549003
5346271000006119	Carcinoma of lower third of oesophagus	254551004
5346291000006118	Carcinoma of cardia	254553001
5346301000006117	Carcinoma of fundus of stomach	254555008
5346311000006119	Carcinoma of body of stomach	254557000
5346321000006110	Carcinoma of pyloric antrum	254559002
5346331000006113	Carcinoma of pylorus	254561006
5346341000006115	Carcinoma of lesser curve of stomach	254563009

5346371000006111	Carcinoma of greater curve of stomach	254567005
5346381000006114	Carcinoma of duodenum	254570009
5346521000006117	Malignant tumour of anorectal junction	254586002
5346761000006115	Carcinoma of ampulla of Vater	254609000
5346771000006110	Ampullary carcinoma	254609000
5346791000006111	Malignant tumour of endocrine pancreas	254611009
5346811000006110	Carcinoma of endocrine pancreas	254612002
5346821000006119	Endocrine pancreatic carcinoma	254612002
5346931000006114	Adenoid cystic carcinoma of trachea	254619006
5347001000006117	Carcinoma of bronchus	254622008
5347031000006113	Bronchial carcinoma	254622008
5347041000006115	Bronchogenic carcinoma	254622008
5347081000006114	Malignant tumour of lung parenchyma	254625005
5347131000006114	Carcinoma of lung parenchyma	254628007
5347171000006112	SCLC - Small cell lung cancer	254632001
5347301000006115	NSCLC - Non-small cell lung cancer	254637007
5347561000006114	Malignant epithelial neoplasm of skin	254650008
5347661000006110	Spindle cell squamous carcinoma of skin	254653005
5347701000006119	Plantar verrucous carcinoma	254655003
5348661000006111	Malignant skin tumour with eccrine differentiation	254707006
5348711000006117	Eccrine porocarcinoma	254708001
5348791000006110	Adenoid cystic eccrine carcinoma	254711000
5348821000006118	Microcystic adnexal carcinoma	254712007
5348881000006119	Mucoepidermoid carcinoma of skin	254713002
5349281000006110	Superficial spreading malignant melanoma of skin	254730000
5349301000006114	SSMM - Superficial spreading malignant melanoma of skin	254730000
5349321000006116	Nodular malignant melanoma of skin	254731001
5349351000006113	Acral lentiginous malignant melanoma of skin	254732008
5351261000006118	Malignant tumour of mesothelial tissue	254824006
5351341000006112	Malignant lipomatous tumour	254828009
5351501000006117	Malignant tumor of breast	254837009
5351511000006119	Breast cancer	254837009
5351521000006110	CA - Breast cancer	254837009
5351531000006113	Malignant neoplasm of breast	254837009
5351541000006115	Carcinoma of breast	254838004
5351551000006118	CA - Carcinoma of breast	254838004
5351571000006111	Inflammatory carcinoma of breast	254840009
5351611000006118	Malignant phyllodes tumour of breast	254844000
5351621000006114	Malignant phyllodes tumor of breast	254844000
5351711000006114	Malignant epithelial tumour of ovary	254849005
5351731000006115	Carcinoma of ovary	254849005
5351761000006112	Endometrioid carcinoma ovary	254852002
5351811000006117	Undifferentiated carcinoma of ovary	254856004
5351881000006112	Malignant sex cord tumour of ovary	254860001
5352081000006114	Choriocarcinoma of ovary	254870004
5352211000006114	Endometrial carcinoma	254878006
5352331000006111	Adenosquamous carcinoma of cervix	254888007
5352401000006112	Carcinoma of vagina	254893005
5352471000006118	Malignant melanoma of vulva	254896002
5352541000006110	Carcinoma of prostate	254900004
5352561000006114	Prostate carcinoma	254900004
5352571000006119	Prostate cancer	254900004
5352611000006112	Carcinoma of glans penis	254904008
5352651000006113	Malignant tumour of skin of penis	254908006
5352681000006117	Cancer of penile skin	254908006
5352691000006119	Malignant tumour of penile skin	254908006
5352701000006119	Carcinoma of foreskin	254909003
5352751000006115	Regressed malignant testicular tumour	254912000
5353491000006112	Pituitary carcinoma	254955001
5353761000006111	Malignant tumour of optic nerve and sheath	254972008
5353781000006118	Malignant astrocytoma of optic nerve	254973003
5353811000006116	Malignant meningioma of optic nerve sheath	254975005
5354201000006116	Malignant haemangiopericytoma of orbit	254995004
5354371000006118	Malignant melanoma of conjunctiva	255004001
5354501000006119	Malignant melanoma of ciliary body	255015006
5354731000006117	Follicular thyroid carcinoma	255028004
5354751000006112	Papillary thyroid carcinoma	255029007
5354761000006114	PTC - Papillary thyroid carcinoma	255029007
5354771000006119	Mixed follicular and papillary thyroid carcinoma	255030002
5354781000006116	Papillary thyroid carcinoma, follicular variant	255030002
5354791000006118	Follicular variant of papillary thyroid carcinoma	255030002

5354801000006117	Anaplastic thyroid carcinoma	255031003
5354811000006119	Medullary thyroid carcinoma	255032005
5354861000006116	Adrenal carcinoma	255035007
5354891000006112	Parathyroid carcinoma	255037004
5355201000006117	Malignant tumor of unknown origin	255052006
5355231000006113	Malignant tumour - unknown primary	255052006
5355241000006115	UKP - Malignant tumour - unknown primary	255052006
5355251000006118	CA - Cancer of unknown origin	255052006
5355271000006111	Cancer - unknown origin	255052006
5355631000006110	Malignant tumour of salivary gland	255072001
5355681000006111	Malignant tumour of ear, nose and throat	255073006
5355701000006114	Malignant tumour of nasal cavity and nasopharynx	255074000
5355761000006110	Malignant tumour of oesophagus, stomach and duodenum	255078002
5355791000006119	Carcinoma of cecum	255081007
5355801000006118	Malignant tumour of anus and anal canal	255083005
5355881000006110	Malignant polyp of biliary tract	255087006
5355941000006114	Malignant tumour of epidermal appendage	255093003
5355981000006115	Malignant tumour of dermis	255096006
5356111000006119	Carcinoma of bladder	255108000
5356161000006116	Malignant tumour of pituitary and hypothalamus	255112006
5356221000006117	Secondary lymphangitic carcinoma	255118005
5356231000006119	Lymphangitis carcinomatosa	255119002
5356241000006112	Carcinomatosis of peritoneal cavity	255121007
5356261000006111	Peritoneal carcinomatosis	255121007
5356331000006110	Malignant infiltration of peripheral nerve	255128001
5441321000006113	Occult carcinoma	261481006
5477411000006119	Microinvasive carcinoma	264550001
5511561000006114	Malignant neoplasm of digestive organs and peritoneum	269456006
5511601000006114	Malignant tumour of greater curve of stomach	269460009
5511671000006115	Malignant neoplasm of soft tissue	269469005
5511991000006111	Tongue carcinoma	269516007
5512021000006115	Carcinoma of colon	269533000
5512031000006117	Carcinoma of the rectosigmoid junction	269544008
5512051000006112	Rectosigmoid junction cancer	269544008
5512081000006116	Malignant melanoma of head and neck	269578002
5512111000006110	Malignant melanoma of lower limb	269581007
5531741000006110	Carcinoma of base of tongue	271943005
5559981000006116	Palate carcinoma	274084007
5559991000006118	Tonsil carcinoma	274085008
5567551000006115	Malignant lymphoma - lymphocytic, intermediate differentiation	274905008
5572831000006119	Carcinoma ventral surface of tongue	275394001
5572861000006111	Carcinoma of midline of tongue	275397008
5573751000006114	Carcinoma of tongue base - dorsal surface	275490009
5590011000006111	Amelanotic malignant melanoma of skin	276751004
5590621000006114	Malignant tumour of fibrous tissue	276797002
5590891000006110	Late gastric cancer	276810009
5591061000006117	Malignant melanoma of anus	276821000
5591081000006110	Malignant melanoma of rectum	276822007
5591131000006110	Malignant glioma of brain	276826005
5591141000006117	Malignant glioma of spinal cord	276827001
5591711000006117	Carcinoma of fallopian tube	276870001
5591891000006116	Carcinoma of Bartholin's gland	276876007
5593241000006110	Carcinoma of larynx	276975007
5593251000006112	Laryngeal carcinoma	276975007
5595371000006112	Malignant tumour of external ear	277156006
5600821000006119	Malignant white blood cell disorder	277543005
5602351000006117	Follicular malignant lymphoma - mixed cell type	277624003
5602371000006110	Follicular malignant lymphoma - small cleaved cell	277625002
5602571000006115	Follicular malignant lymphoma - large cell	277641001
5602911000006119	Malignant lymphoma of testis	277664004
5604701000006114	Malignant peritoneal local recurrence	277782009
5607891000006118	Malignant teratoma of mediastinum	278042005
5607901000006119	Malignant seminoma of mediastinum	278043000
5608041000006119	Malignant lymphoma of thyroid gland	278051002
5608051000006117	Malignant lymphoma of breast	278052009
5608071000006110	Infiltrating lobular carcinoma of breast	278054005
5608081000006113	Lobular carcinoma of breast	278054005
5608211000006113	Endometrioid carcinoma of prostate	278060005
5613211000006119	Malignant infiltration of soft tissue	278433008
5699721000006117	Carcinoma of anal canal	285310000
5699751000006114	Carcinoma of sigmoid colon	285312008

5701321000006112	Carcinoma of cervix	285432005
5720811000006114	Carcinoma liver and/or biliary system	286887005
5720831000006115	Carcinoma of upper limb bones/scapula	286889008
5720851000006110	Carcinoma of skin of head/neck	286891000
5720861000006112	Carcinoma of breast - upper, inner quadrant	286893002
5720871000006117	Carcinoma of breast - lower, inner quadrant	286894008
5720881000006119	Carcinoma of breast - upper, outer quadrant	286895009
5720891000006116	Carcinoma breast - lower, outer quadrant	286896005
5720901000006117	Carcinoma of breast - axillary tail	286897001
5720941000006115	Secondary carcinoma of gastrointestinal tract	286902000
5911421000006114	Malignant tumour of soft tissue of head, face and neck	302816009
5911441000006119	Malignant tumour of unknown origin or ill-defined site	302817000
5911951000006111	Malignant lymphoma - small lymphocytic	302841002
5912011000006117	Diffuse malignant lymphoma - centroblastic	302842009
5912081000006112	Nodular malignant lymphoma, lymphocytic - well differentiated	302845006
5914251000006115	Malignant lymphoma, follicular centre cell	303055001
5970661000006117	Malignant lymphoma - lymphoplasmacytic	307623001
5971001000006115	Malignant lymphoma, centroblastic type, follicular	307647008
6012841000006119	Cholangiocarcinoma	312104005
6012851000006117	Cholangiocarcinoma of biliary tract	312104005
6012931000006119	Carcinoma of ascending colon	312111009
6012941000006112	Carcinoma of transverse colon	312112002
6012951000006114	Carcinoma of descending colon	312113007
6012961000006111	Carcinoma of hepatic flexure	312114001
6012971000006116	Carcinoma of splenic flexure	312115000
6051841000006117	Local recurrence of malignant tumour of tongue	314951005
6051861000006118	Local recurrence of malignant tumour of buccal cavity	314952003
6051881000006111	Local recurrence of malignant tumour of thyroid gland	314953008
6051891000006114	Local recurrence of malignant tumor of thyroid gland	314953008
6051901000006113	Local recurrence of malignant tumour of lung	314954002
6051911000006111	Local recurrence of malignant tumor of lung	314954002
6051931000006117	Local recurrence of malignant tumor of breast	314955001
6051991000006118	Local recurrence of malignant tumour of oesophagus	314960002
6052031000006112	Local recurrence of malignant tumour of gallbladder	314962005
6052051000006117	Local recurrence of malignant tumour of liver	314963000
6052061000006115	Local recurrence of malignant tumor of liver	314963000
6052071000006110	Local recurrence of malignant tumour of pancreas	314964006
6052091000006111	Local recurrence of malignant tumour of colon	314965007
6052111000006119	Local recurrence of malignant tumour of rectum	314966008
6052131000006113	Local recurrence of malignant tumour of kidney	314967004
6052161000006116	Local recurrence of malignant tumor of urinary bladder	314968009
6052171000006111	Local recurrence of malignant tumour of prostate	314969001
6052181000006114	Local recurrence of malignant tumor of prostate	314969001
6052191000006112	Local recurrence of malignant tumour of cervix	314970000
6052231000006119	Local recurrence of malignant tumour of bone	314973003
6052251000006114	Local recurrence of malignant tumour of soft tissue	314974009
6052291000006115	Local recurrence of malignant melanoma of skin	314976006
6052381000006111	Metastasis from malignant melanoma of skin	314987003
6052391000006114	Metastasis from malignant tumour of skin	314988008
6052401000006111	Metastasis from malignant tumor of skin	314988008
6052411000006114	Metastasis from malignant tumour of soft tissues	314989000
6052431000006115	Metastasis from malignant tumour of bone	314990009
6052451000006110	Metastasis from malignant tumour of adrenal gland	314991008
6052471000006117	Metastasis from malignant tumour of cervix	314992001
6052491000006116	Metastasis from malignant tumour of uterus	314993006
6052511000006110	Metastasis from malignant tumour of prostate	314994000
6052521000006119	Metastasis from malignant tumor of prostate	314994000
6052531000006116	Cancer of the prostate with metastasis	314994000
6052541000006114	Metastatic prostate cancer	314994000
6052551000006111	Metastasis from malignant tumour of bladder	314995004
6052571000006118	Metastasis from malignant tumour of kidney	314996003
6052591000006117	Metastasis from malignant tumour of rectum	314997007
6052601000006113	Metastasis from malignant tumor of rectum	314997007
6052611000006111	Metastasis from malignant tumour of colon	314998002
6052621000006115	Metastasis from malignant tumor of colon	314998002
6052631000006117	Metastasis from malignant tumour of pancreas	314999005
6052641000006110	Metastasis from malignant tumor of pancreas	314999005
6052651000006112	Metastasis from malignant tumour of liver	315000005
6052661000006114	Metastasis from malignant tumor of liver	315000005
6052671000006119	Metastasis from malignant tumour of gallbladder	315001009
6052691000006118	Metastasis from malignant tumour of stomach	315002002

6052711000006115	Metastasis from malignant tumour of oesophagus	315003007
6052731000006114	Metastasis from malignant tumour of breast	315004001
6052741000006116	Metastasis from malignant tumor of breast	315004001
6052751000006119	Metastasis from malignant tumour of bronchus	315005000
6052771000006112	Metastasis from malignant tumour of lung	315006004
6052781000006110	Metastasis from malignant tumor of lung	315006004
6052791000006113	Metastasis from malignant tumour of thyroid	315007008
6052831000006118	Metastasis from malignant tumour of tongue	315009006
6053471000006110	HNPCC - hereditary nonpolyposis colon cancer	315058005
6131001000006118	Carcinoma of head of pancreas	326072005
6206231000006116	Metastatic malignant neoplasm to lateral axillary lymph nodes	359780007
6206321000006114	Secondary malignant neoplasm of dome of urinary bladder	359785002
6242751000006114	Malignant tumour	363346000
6242761000006111	Malignant tumor	363346000
6242781000006118	CA - Cancer	363346000
6242861000006118	Cancer of stomach	363349007
6242871000006113	Gastric cancer	363349007
6242881000006111	CA - Cancer of stomach	363349007
6242891000006114	Malignant tumour of caecum	363350007
6242911000006111	CA - Cancer of caecum	363350007
6242921000006115	Cancer of caecum	363350007
6242961000006114	Cancer of cecum	363350007
6242971000006119	Cecal cancer	363350007
6242981000006116	Malignant tumour of rectum	363351006
6243001000006117	Rectal cancer	363351006
6243011000006119	CA - Cancer of rectum	363351006
6243031000006113	Malignant tumor of anal canal	363352004
6243041000006115	Cancer of anal canal	363352004
6243051000006118	Malignant tumour of gallbladder	363353009
6243091000006112	Malignant tumor of cervix	363354003
6243101000006118	Cancer of cervix	363354003
6243111000006115	Malignant neoplasm of cervix	363354003
6243141000006116	Cervical cancer	363354003
6243151000006119	Malignant tumour of adrenal gland	363355002
6243181000006110	Cancer of adrenal gland	363355002
6243261000006112	CA - Lung cancer	363358000
6243271000006117	Malignant tumour of middle ear	363359008
6243281000006119	Malignant tumor of middle ear	363359008
6243291000006116	Malignant tumour of anterior two-thirds of tongue	363360003
6243311000006117	Malignant tumour of soft tissue of shoulder	363363001
6243341000006118	Malignant tumour of soft tissue of hip	363364007
6243371000006114	Malignant tumour of soft tissue of thorax	363365008
6243401000006112	Malignant tumour of soft tissue of pelvis	363366009
6243441000006114	Cancer of vulva	363367000
6243451000006111	Carcinoma of body of pancreas	363368005
6243461000006113	Carcinoma of tail of pancreas	363369002
6243591000006118	Malignant tumour of commissure of lip	363374005
6243651000006113	CA - Cancer of tongue	363375006
6243661000006110	Cancer of tongue	363375006
6243671000006115	Malignant tumour of base of tongue	363376007
6243761000006118	Malignant tumour of lingual tonsil	363377003
6243831000006119	Cancer of parotid gland	363379000
6243841000006112	CA - Cancer of parotid gland	363379000
6243851000006114	Malignant tumour of submandibular gland	363380002
6243861000006111	Malignant tumor of submandibular gland	363380002
6243881000006118	Malignant tumour of sublingual gland	363381003
6243901000006116	Malignant tumour of gum	363382005
6243921000006114	Malignant tumour of alveolar mucosa	363382005
6243931000006112	Malignant tumour of gingiva	363382005
6243961000006115	Cancer of gum	363382005
6243981000006113	Malignant tumour of upper gingiva	363383000
6244021000006112	Malignant tumour of lower gingiva	363384006
6244111000006117	Cancer of floor of mouth	363385007
6244121000006113	CA - Cancer of floor of mouth	363385007
6244141000006118	Malignant tumour of buccal mucosa	363386008
6244171000006114	Malignant tumour of cheek mucosa	363386008
6244211000006111	CA - Cancer of buccal mucosa	363386008
6244221000006115	Cancer of cheek mucosa	363386008
6244241000006110	Malignant tumour of hard palate	363387004
6244261000006114	Malignant tumour of soft palate	363388009
6244281000006116	Malignant tumour of uvula	363389001

6244321000006110	Malignant tumour of roof of mouth	363390005
6244351000006118	Cancer of palate	363390005
6244371000006111	Malignant tumour of retromolar area	363391009
6244411000006112	Cancer of oropharynx	363392002
6244421000006116	CA - Cancer of oropharynx	363392002
6244451000006113	Malignant tumour of tonsil	363393007
6244511000006113	Cancer of tonsil	363393007
6244541000006112	CA - Cancer of tonsil	363393007
6244571000006116	Malignant tumour of vallecula	363395000
6244611000006114	Malignant tumour of roof of nasopharynx	363397008
6244701000006116	Malignant tumour of laryngopharynx	363399006
6244731000006112	Malignant tumour of postcricoid region	363400004
6244751000006117	Malignant tumour of pharyngo-oesophageal junction	363400004
6244771000006110	Malignant tumour of pyriform fossa	363401000
6244781000006113	Malignant tumor of pyriform fossa	363401000
6244811000006110	Malignant tumour of oesophagus	363402007
6244831000006116	Cancer of oesophagus	363402007
6244851000006111	CA - Cancer of oesophagus	363402007
6244891000006117	Esophageal cancer	363402007
6244901000006118	Malignant neoplasm of esophagus	363402007
6244911000006115	Malignant tumour of duodenum	363403002
6244931000006114	Duodenal cancer	363403002
6244941000006116	Malignant tumour of jejunum	363404008
6244991000006113	Malignant tumor of colon	363406005
6245001000006118	CA - Cancer of colon	363406005
6245011000006115	Cancer of colon	363406005
6245031000006114	Malignant tumour of hepatic flexure	363407001
6245081000006110	Malignant tumor of transverse colon	363408006
6245101000006119	Transverse colon cancer	363408006
6245111000006116	Malignant tumour of descending colon	363409003
6245131000006110	Descending colon cancer	363409003
6245151000006115	Malignant tumor of sigmoid colon	363410008
6245161000006118	Sigmoid colon cancer	363410008
6245171000006113	Malignant tumour of appendix	363411007
6245181000006111	Malignant tumor of appendix	363411007
6245201000006112	Malignant tumor of ascending colon	363412000
6245211000006110	Ascending colon cancer	363412000
6245221000006119	Malignant tumour of splenic flexure	363413005
6245231000006116	Malignant tumor of splenic flexure	363413005
6245251000006111	Splenic flexure colon cancer	363413005
6245281000006115	Rectosigmoid colon cancer	363414004
6245291000006117	Malignant tumour of biliary tract	363415003
6245321000006114	Malignant tumor of extrahepatic bile duct	363416002
6245351000006117	Malignant tumour of ampulla of Vater	363417006
6245361000006115	Malignant tumor of ampulla of Vater	363417006
6245381000006113	Malignant tumor of pancreas	363418001
6245391000006111	CA - Cancer of pancreas	363418001
6245401000006113	Pancreatic cancer	363418001
6245411000006111	CA - Pancreatic cancer	363418001
6245421000006115	Malignant tumour of head of pancreas	363419009
6245451000006112	Malignant retroperitoneal tumour	363420003
6245511000006112	Malignant tumour of nasal cavity	363422006
6245541000006111	Malignant tumour of nasal septum	363423001
6245561000006110	Malignant tumour of mastoid air cells	363424007
6245581000006117	Malignant tumour of maxillary sinus	363425008
6245601000006110	Malignant tumour of maxillary antrum	363425008
6245621000006117	Malignant tumour of ethmoid sinus	363426009
6245651000006114	Malignant tumour of frontal sinus	363427000
6245671000006116	Malignant tumour of sphenoid sinus	363428005
6245701000006115	Malignant tumor of larynx	363429002
6245711000006117	CA - Cancer of larynx	363429002
6245721000006113	Laryngeal cancer	363429002
6245731000006111	Cancer of larynx	363429002
6245741000006118	Malignant tumour of subglottis	363430007
6245791000006110	Malignant tumour of trachea	363432004
6245811000006114	Tracheal cancer	363432004
6245871000006117	Cancer of pleura	363433009
6245891000006116	Malignant tumour of thymus	363434003
6245931000006113	Malignant tumour of endocardium	363436001
6245951000006118	Malignant tumour of myocardium	363437005
6245971000006111	Malignant tumour of vertebral column	363438000

6246001000006113	Cancer of spine	363438000
6246021000006115	Malignant tumour of soft tissue of head	363439008
6246051000006112	Malignant tumour of soft tissue of face	363440005
6246081000006116	Malignant tumour of soft tissue of neck	363441009
6246121000006119	Malignant tumor of ovary	363443007
6246131000006116	CA - Cancer of ovary	363443007
6246151000006111	Ovarian cancer	363443007
6246161000006113	Malignant tumour of fallopian tube	363444001
6246171000006118	Malignant tumor of fallopian tube	363444001
6246201000006119	Cancer of vagina	363445000
6246241000006117	Malignant tumor of testis	363449006
6246251000006115	Malignant tumour of foreskin	363450006
6246271000006113	Malignant tumour of prepuce	363450006
6246301000006110	Cancer of foreskin	363450006
6246311000006113	Malignant neoplasm of foreskin	363450006
6246321000006117	Malignant tumour of glans penis	363451005
6246341000006112	Cancer of glans penis	363451005
6246351000006114	Malignant tumour of epididymis	363452003
6246371000006116	Malignant tumour of spermatic cord	363453008
6246401000006118	Malignant tumour of scrotum	363454002
6246471000006112	Malignant tumour of urachus	363456000
6246511000006119	Malignant tumour of ureter	363458004
6246531000006113	Cancer of ureter	363458004
6246551000006118	Malignant tumour of urethra	363459007
6246571000006111	Malignant urethral tumour	363459007
6246581000006114	Malignant urethral tumor	363459007
6246601000006116	CA - Cancer of urethra	363459007
6246651000006117	Malignant tumour of eye	363461003
6246671000006110	Malignant tumour of orbit	363462005
6246691000006111	Malignant orbital tumour	363462005
6246711000006114	Malignant tumour of conjunctiva	363463000
6246731000006115	Malignant tumour of cornea	363464006
6246751000006110	Malignant tumour of retina	363465007
6246771000006117	Malignant retinal tumour	363465007
6246791000006116	Malignant tumour of choroid	363466008
6246801000006115	Malignant tumor of choroid	363466008
6246891000006110	Malignant tumour of spinal cord	363475005
6246901000006114	Malignant tumor of spinal cord	363475005
6246931000006118	Malignant tumour of thyroid gland	363478007
6246951000006113	Thyroid cancer	363478007
6246971000006115	Malignant tumour of parathyroid gland	363481002
6246991000006119	Parathyroid cancer	363481002
6247001000006110	Malignant tumour of pituitary gland	363482009
6247051000006114	Malignant tumour of pineal gland	363483004
6247091000006115	Malignant tumour of pelvis	363484005
6247111000006112	Malignant tumour of minor salivary gland	363485006
6247131000006118	Malignant tumour of vocal cord	363486007
6247141000006111	Malignant tumor of vocal cord	363486007
6247171000006115	CA - Cancer of vocal cord	363486007
6247181000006117	Cancer of vocal cord	363486007
6247341000006114	Malignant tumour of peritoneum	363492001
6247351000006111	Malignant tumor of peritoneum	363492001
6247361000006113	Peritoneal cancer	363492001
6247371000006118	CA - Cancer of peritoneum	363492001
6247381000006115	Cancer of peritoneum	363492001
6247391000006117	Malignant tumour of bronchus	363493006
6247451000006116	Malignant neoplasm of mediastinum	363494000
6247481000006112	Malignant tumour of muscle	363495004
6247521000006112	Malignant tumour of soft tissue of abdomen	363496003
6247561000006118	Malignant tumour of optic nerve	363498002
6247631000006114	Malignant tumour of face	363501002
6247671000006112	Malignant tumour of upper limb	363503004
6247711000006111	Malignant tumour of oral cavity	363505006
6247721000006115	Malignant tumor of oral cavity	363505006
6247731000006117	Malignant tumour of mouth	363505006
6247751000006112	Cancer of oral cavity	363505006
6247761000006114	CA - Mouth cancer	363505006
6247771000006119	Mouth cancer	363505006
6247781000006116	Malignant neoplasm of mouth	363505006
6247811000006119	Malignant tumour of pharynx	363507003
6247821000006110	Malignant tumor of pharynx	363507003

6247831000006113	Cancer of pharynx	363507003
6247841000006115	CA - Cancer of pharynx	363507003
6247851000006118	Malignant neoplasm of pharynx	363507003
6247871000006111	Malignant tumor of intestine	363508008
6247891000006112	Malignant neoplasm of intestine	363508008
6247911000006114	Intestinal cancer	363508008
6247931000006115	CA - Cancer of bowel	363508008
6247961000006112	Malignant tumour of small bowel	363509000
6247971000006117	Malignant tumor of small bowel	363509000
6247981000006119	Malignant tumour of large intestine	363510005
6248021000006116	CA - Cancer of large bowel	363510005
6248031000006118	Colorectal cancer	363510005
6248101000006113	Cancer of penis	363516004
6248131000006117	CA - Cancer of penis	363516004
6248141000006110	Malignant tumour of urinary tract proper	363517008
6248191000006118	Malignant tumor of kidney	363518003
6248201000006115	Renal malignant tumour	363518003
6248211000006117	Renal malignant tumor	363518003
6248231000006111	CA - Cancer of kidney	363518003
6248241000006118	Renal cancer	363518003
6250441000006117	Primary malignant neoplasm of gastrointestinal tract	363745004
6300141000006111	Malignant neoplasm of primary, secondary, or uncertain origin	367651003
6324621000006110	Malignant tumour involving rectum by direct extension from uterine cervix	369452007
6324741000006114	Malignant tumour involving rectum by separate metastasis from prostate	369458006
6324831000006112	Malignant tumour involving ureter by direct extension from bladder	369463005
6325291000006111	Malignant tumour involving prostate by separate metastasis from bladder	369486003
6325321000006119	Secondary malignant neoplasm of seminal vesicle	369488002
6325351000006111	Malignant tumour involving seminal vesicle by direct extension from prostate	369490001
6325811000006119	Primary malignant neoplasm of left fallopian tube	369513003
6325821000006110	Secondary malignant neoplasm of left fallopian tube	369514009
6325851000006118	Malignant tumour involving left fallopian tube by direct extension from ovary	369516006
6325931000006115	Primary malignant neoplasm of right fallopian tube	369520005
6325951000006110	Primary malignant neoplasm of left ovary	369522002
6325961000006112	Secondary malignant neoplasm of left ovary	369523007
6326081000006112	Primary malignant neoplasm of right ovary	369529006
6326091000006110	Secondary malignant neoplasm of right ovary	369530001
6327071000006112	Malignant tumour involving vagina by direct extension from uterine cervix	369580007
6327351000006117	Malignant tumour involving an organ by direct extension from bladder	369594008
6327431000006117	Malignant tumour involving an organ by direct extension from prostate	369598006
6327511000006112	Malignant tumour involving an organ by separate metastasis from bladder	369602008
6327531000006118	Malignant tumour involving an organ by separate metastasis from endometrium	369603003
6327571000006115	Malignant tumour involving an organ by separate metastasis from ovary	369605005
6327591000006119	Malignant tumour involving an organ by separate metastasis from prostate	369606006
6327631000006119	Malignant tumour involving an organ by separate metastasis from uterus	369608007
6336641000006112	Choriocarcinoma, metastatic	370079006
6362241000006116	Primary malignant neoplasm of ampulla of Vater	371967001
6362271000006112	Primary malignant neoplasm of biliary tract	371970002
6362301000006114	Malignant tumour of body of uterus	371972005
6362341000006111	Malignant tumour of uterus	371973000
6362351000006113	Malignant tumor of uterus	371973000
6362361000006110	CA - Cancer of uterus	371973000
6362381000006117	Uterine cancer	371973000
6362391000006119	Malignant neoplasm of border of tongue	371974006
6362421000006110	Primary malignant neoplasm of buccal mucosa	371976008
6362431000006113	Primary malignant neoplasm of caecum	371977004
6362551000006117	Primary malignant neoplasm of endocrine gland	371983001
6362561000006115	Primary malignant neoplasm of oesophagus	371984007
6362571000006110	Primary malignant neoplasm of esophagus	371984007
6362581000006113	Primary malignant neoplasm of eye	371986009
6362591000006111	Primary malignant neoplasm of fallopian tube	371987000
6362601000006115	Primary malignant neoplasm of false vocal cord	371988005
6362611000006117	Primary malignant neoplasm of glans penis	371989002
6362621000006113	Primary malignant neoplasm of gum	371990006
6362631000006111	Primary malignant neoplasm of hard palate	371991005
6362641000006118	Primary malignant neoplasm of intestinal tract	371992003
6362661000006119	Primary malignant neoplasm of laryngeal aspect of aryepiglottic fold	371994002
6362671000006114	Primary malignant neoplasm of larynx	371995001
6362681000006112	Primary malignant neoplasm of lip	371996000
6362691000006110	Primary malignant neoplasm of lower gum	371997009
6362701000006110	Primary malignant neoplasm of lower third of oesophagus	371998004
6362721000006117	Primary malignant neoplasm of middle third of oesophagus	371999007

6362741000006112	Primary malignant neoplasm of minor salivary gland	372000001
6362751000006114	Primary malignant neoplasm of oral cavity	372001002
6362761000006111	Primary malignant neoplasm of palate	372002009
6362771000006116	Primary malignant neoplasm of pancreas	372003004
6362781000006118	Primary malignant neoplasm of parotid gland	372004005
6362791000006115	Primary malignant neoplasm of penis	372005006
6362801000006119	Primary malignant neoplasm of prepuce	372006007
6362841000006117	Primary malignant neoplasm of scrotum	372009000
6362851000006115	Primary malignant neoplasm of soft tissues	372010005
6362901000006113	Primary malignant neoplasm of stomach	372014001
6362911000006111	Primary malignant neoplasm of the mesentery	372015000
6362921000006115	Primary malignant neoplasm of the peritoneum	372016004
6362971000006119	Primary malignant neoplasm of tonsil	372020000
6363001000006117	Primary malignant neoplasm of upper third of oesophagus	372023003
6363021000006110	Primary malignant neoplasm of uterine cervix	372024009
6363031000006113	Primary malignant neoplasm of vagina	372025005
6363081000006114	Primary malignant neoplasm of vocal cord	372030009
6363631000006118	Female breast cancer	372064008
6363951000006117	Primary malignant neoplasm	372087000
6363981000006113	Primary malignant neoplasm of axillary tail of breast	372092003
6363991000006111	Secondary malignant neoplasm of axillary tail of breast	372093008
6364001000006119	Malignant neoplasm of axillary tail of breast	372094002
6364041000006117	Carcinoma of male breast	372096000
6364071000006113	Carcinoma of endocervix	372098004
6364111000006117	Carcinoma of extrahepatic bile duct	372101000
6364171000006114	Carcinoma of glottis	372103002
6364181000006112	Carcinoma of subglottis	372104008
6364191000006110	Carcinoma of supraglottis	372105009
6364201000006113	Carcinoma of penis	372106005
6364231000006117	Malignant neoplasm of bone of lower limb	372108006
6364271000006119	Primary malignant neoplasm of lower lobe, bronchus or lung	372110008
6364281000006116	Carcinoma of lower lobe, bronchus or lung	372111007
6364361000006116	Primary malignant neoplasm of head of pancreas	372119009
6364371000006111	Carcinoma of main bronchus	372120003
6364381000006114	Carcinoma of ribs and/or sternum and/or clavicle	372121004
6364401000006114	Malignant neoplasm of skin head and neck	372122006
6364421000006116	Primary malignant neoplasm of skin head and neck	372123001
6364431000006118	Malignant neoplasm of skin of lower limb	372124007
6364451000006113	Carcinoma of skin of lower limb	372125008
6364491000006119	Malignant neoplasm of skin of upper limb	372128005
6364531000006119	Skin cancer	372130007
6364541000006112	Cancer of skin	372130007
6364651000006110	Carcinoma of upper lobe, bronchus or lung	372136001
6364661000006112	Primary malignant neoplasm of breast	372137005
6364671000006117	Carcinoma of oesophagus	372138000
6364691000006116	Primary malignant neoplasm of gallbladder	372139008
6364711000006118	Carcinoma of vocal cord	372141009
6364721000006114	Carcinoma of pancreas	372142002
6364731000006112	Carcinoma of stomach	372143007
6364741000006119	Gastric carcinoma	372143007
6364891000006117	Malignant melanocytic lesion	372156000
6364901000006118	Malignant melanoma, metastatic	372158004
6365751000006116	Malignant melanoma	372244006
6376181000006114	Malignant neoplasm of breast lower inner quadrant	373080008
6376191000006112	Malignant neoplasm of breast lower outer quadrant	373081007
6376201000006110	Malignant neoplasm of breast upper inner quadrant	373082000
6376211000006113	Malignant neoplasm of breast upper outer quadrant	373083005
6377211000006111	pN3b: Tumor of breast with metastasis as per American Joint Committee on Cancer 6th Edition definition (breast)	373167007
6431441000006111	Carcinoma with pleomorphic, sarcomatoid or sarcomatous elements	384950003
6581501000006113	Salivary duct carcinoma	397082006
6616721000006113	Malignant tumor of prostate	399068003
6616731000006111	Malignant prostatic tumour	399068003
6616751000006116	CA - Cancer of prostate	399068003
6616761000006119	Cancer of prostate	399068003
6620961000006112	Malignant tumour of urinary bladder	399326009
6620971000006117	Malignant tumor of urinary bladder	399326009
6620981000006119	Bladder cancer	399326009
6621001000006115	CA - Bladder cancer	399326009
6636191000006118	Eccrine carcinoma of skin	400173004
6667161000006117	Multiple primary malignant melanomata	402564006
6671091000006114	Malignant fibrohistiocytic tumour of skin	402873007

6671131000006111	Malignant tumour of nerve sheath origin	402876004
6671871000006118	Vulval verrucous carcinoma of Buschke-Löwenstein	402912009
6689141000006110	Verrucous carcinoma of oral cavity	403889000
6689181000006116	Cancer of the scalp, squamous cell	403891008
6689201000006115	Squamous cell cancer of skin of face	403892001
6689621000006110	Balloon cell malignant melanoma	403922007
6689631000006113	Spindle cell malignant melanoma	403923002
6689641000006115	Spitzoid malignant melanoma	403923002
6689651000006118	Desmoplastic malignant melanoma	403924008
6689671000006111	Malignant melanoma of oral cavity	403926005
6689681000006114	Malignant melanoma of nail apparatus	403927001
6689711000006110	Trichilemmal carcinoma	403929003
6689891000006111	Eccrine ductal carcinoma	403939009
6689971000006116	Malignant cylindroma	403941005
6690251000006112	Mixed eccrine/pilar adnexal carcinoma of skin	403954003
6691671000006111	Malignant peripheral nerve sheath tumour	404037002
6691761000006113	Malignant Triton tumour	404040002
6692441000006110	Malignant infiltration of skin by underlying tumour	404091004
6692461000006114	Carcinomatous metastasis in skin	404092006
6693231000006110	Anaplastic large T-cell systemic malignant lymphoma	404134006
6702751000006112	Malignant optic glioma	404664002
6718941000006112	Cancer of the larynx, squamous cell	405822008
6719331000006114	Widespread metastatic malignant neoplastic disease	405843009
6719411000006113	CA - Disseminated cancer	405843009
6765091000006112	Infiltrating duct carcinoma of breast	408643008
6765101000006118	Invasive duct carcinoma of breast	408643008
6765111000006115	Infiltrating ductal carcinoma of breast	408643008
6765121000006111	Invasive ductal carcinoma of breast	408643008
6841351000006112	Acute myeloid leukaemia	413443009
6847711000006116	Chronic eosinophilic leukaemia	413836008
6861741000006118	Malignant histiocytic neoplasm	414645001
6861771000006114	Malignant meningeal neoplasm	414647009
6871551000006115	Primary malignant neoplasm of ear, nose AND/OR throat	415177008
6931041000006119	Malignant tumor of urinary system	419052002
6955611000006112	Malignant lymphoma of the eye region	420519005
6960371000006112	Primary intraocular non-Hodgkin malignant lymphoma	420788006
6981471000006111	Papillary carcinoma, solid	421980000
6988591000006115	Colon cancer, stage 3	422375001
6991611000006118	Undifferentiated carcinoma of nasopharynx	422541001
6992241000006113	Colon cancer, stage 2	422581008
6995681000006111	Ovarian cancer, disseminated	422782004
6996671000006119	Adenoid cystic carcinoma of salivary gland	422833009
7001961000006115	Infiltrating ductal carcinoma of breast, stage 3	423114009
7003181000006111	Adenoid cystic carcinoma of submandibular gland	423189008
7004611000006110	Carcinoma of ovary, stage 4	423274005
7004631000006116	Ovarian cancer stage 4	423274005
7004761000006116	Malignant melanoma of skin of canthus of eye	423280002
7004851000006112	Cancer of skin of neck, squamous cell	423284006
7007181000006118	Mucoepidermoid carcinoma of submandibular gland	423424005
7008241000006119	Carcinoma of ovary, stage 3	423480004
7008251000006117	Ovarian cancer stage 3	423480004
7010761000006113	Adenoid cystic carcinoma of parotid gland	423615009
7011781000006113	Malignant melanoma of retina	423673009
7012421000006113	Mucoepidermoid carcinoma of salivary gland	423708008
7014071000006111	Mucoepidermoid carcinoma of parotid gland	423793008
7017461000006117	Carcinoma of uterine cervix, invasive	423973006
7017741000006113	Cancer of vulva, disseminated	423987006
7021611000006119	Malignant melanoma of unknown origin	424190005
7023221000006112	Malignant glioma of brainstem	424276002
7024321000006115	Malignant tumour of spinal cord, intramedullary	424334007
7027281000006117	Ovarian cancer stage 2	424486004
7028481000006119	Malignant tumour of spinal cord, extramedullary	424549003
7029491000006110	Carcinoma of ovary, stage 1	424600001
7029501000006119	Cancer of ovary, stage 1	424600001
7029511000006116	Ovarian cancer stage 1	424600001
7034801000006116	Thyroid cancer metastatic to bone	424887002
7037801000006111	Carcinoma of urinary bladder, invasive	425066001
7037811000006114	Invasive bladder cancer	425066001
7039021000006115	Carcinoma ex pleomorphic adenoma of parotid gland	425127006
7040851000006113	Carcinoma of urinary bladder, superficial	425231005
7040861000006110	Superficial bladder cancer	425231005

7066961000006112	Epidermal growth factor receptor positive non-small cell lung cancer	426964009
7074671000006114	Hormone refractory prostate cancer	427492003
7074681000006112	Castration-resistant prostate cancer	427492003
7074691000006110	Castrate-resistant prostate cancer	427492003
7086121000006119	Malignant neoplasm of bone	428281000
7097371000006117	Malignant neoplasm of cerebrum	429033009
7123191000006114	Malignant neoplasm of genital structure	430556008
7157771000006116	Neuroblastoma	432328008
7278231000006113	Malignant neoplasm of anorectum	443488001
7278241000006115	Anorectal cancer	443488001
7278381000006116	Metastatic malignant melanoma	443493003
7280821000006117	Malignant neoplasm of ear	443648003
7287391000006111	Invasive carcinoma with ductal and lobular features (Mixed type carcinoma)	444057000
7291431000006114	Synchronous primary carcinomas	444327002
7295011000006114	Malignant thymoma	444596001
7295111000006113	Mixed ductal and lobular carcinoma of breast	444604002
7305001000006118	Malignant carcinoid tumour	445238008
7317761000006111	Carcinoma of uterus	446022000
7320611000006113	Primary malignant neoplasm of extrahepatic bile duct	446189008
7330371000006113	Primary malignant neoplasm of perihilar bile duct	446807009
7334851000006116	Primary malignant neoplasm of intrahepatic bile duct	447109003
7339561000006115	Primary malignant neoplasm of distal bile duct	447416008
7344901000006113	Malignant melanoma of skin of anus	447712006
7345801000006115	Carcinoma of peritoneum	447781009
7345821000006113	Carcinoma of female breast	447782002
7347011000006118	Carcinoma of vulva	447882007
7351551000006117	Malignant epithelial neoplasm of oropharynx	448214005
7351561000006115	Carcinoma of renal pelvis	448215006
7351581000006113	Carcinoma of thyroid	448216007
7351621000006113	Malignant neoplasm of cerebellopontine angle	448218008
7352111000006119	Malignant neoplasm of axial suprasellar region of brain	448248006
7352441000006113	Malignant melanoma of skin of scrotum	448273006
7352451000006110	Malignant neoplasm of connective tissue	448274000
7352781000006110	Malignant melanoma of skin of penis	448298007
7352801000006114	Malignant melanoma of skin of vulva	448300007
7352971000006114	Carcinoma of spinal cord	448314007
7352991000006110	Carcinoma of anus	448315008
7354451000006115	Choriocarcinoma of placenta	448401007
7356051000006116	Transglottic malignant neoplasm of larynx	448509007
7356731000006113	Malignant neoplasm of maxillofacial bone	448558006
7358291000006118	Carcinoma of small intestine	448664009
7358301000006117	Carcinoma of small bowel	448664009
7358321000006110	Malignant epithelial neoplasm of hypopharynx	448665005
7358391000006112	Malignant neoplasm of soft tissue of orbit	448669004
7358401000006114	Cancer of soft tissue of orbit	448669004
7358491000006119	Malignant neoplasm of digestive system	448675008
7361161000006117	Carcinoma of pineal gland	448863000
7361191000006113	Carcinoma of ureter	448864006
7361431000006116	Malignant neoplasm of intraabdominal organ	448882009
7361441000006114	Cancer of intraabdominal organ	448882009
7362291000006114	Infiltrating duct carcinoma of female breast	448952004
7362301000006110	Invasive ductal carcinoma of female breast	448952004
7362321000006117	Carcinoma of urethra	448954003
7362851000006116	Carcinoma of nose	448988009
7362871000006114	Carcinoma of brain	448989001
7362891000006110	Carcinoma of nasal cavity	448990005
7362931000006118	Carcinoma of appendix	448992002
7362951000006113	Carcinoma of lung	448993007
7362971000006115	Carcinoma of upper rectum	448994001
7363581000006117	Malignant neoplasm of anterior and lateral floor of mouth	449034009
7364151000006117	Malignant neoplasm of upper respiratory tract	449066004
7364291000006119	Carcinoma of corpus uteri	449073009
7364381000006110	Carcinoma of maxilla	449077005
7365491000006119	Carcinoma of floor of mouth	449156009
7366721000006119	Malignant neoplasm of alveolus of maxilla	449223003
7367041000006113	Nasopharyngeal carcinoma	449248000
7367161000006111	Carcinoma of pharynx	449254004
7367281000006114	Malignant neoplasm of alveolus dentalis	449260004
7369071000006110	Cancer of pelvic peritoneum	449377002
7369621000006115	Carcinoma of nasal septum	449417005
7370781000006113	Carcinoma of mandible	449487002

7372311000006118	Malignant neoplasm of alveolus of mandible	449578008
7373121000006119	Secondary malignant neoplasm of lower leg	449632009
7373151000006111	Primary malignant neoplasm of lower leg	449635006
7373161000006113	Malignant melanoma of skin of lower leg	449636007
7373171000006118	Malignant melanoma of skin of upper arm	449637003
7384781000006112	Micropapillary carcinoma	450895005
7502931000006115	Undifferentiated carcinoma of nasal sinus	697993003
7502951000006110	Sinonasal undifferentiated carcinoma	697993003
7503701000006117	Malignant melanoma of nasal cavity	698040004
7503711000006119	Malignant melanoma of vestibule of mouth	698041000
7503731000006113	Malignant melanoma of floor of mouth	698043002
7503761000006116	Malignant melanoma of buccal mucosa	698045009
7503801000006113	Nasopharyngeal carcinoma type 2b	698048006
7507131000006116	Malignant melanoma of palate	698286006
7507161000006113	Malignant melanoma of maxillary sinus	698288007
7525291000006110	Glandular malignant peripheral nerve sheath tumour	699659007
7570221000006118	Poorly differentiated carcinoma	703078002
7577631000006119	Encapsulated papillary carcinoma	703545003
7577641000006112	Encysted papillary carcinoma	703545003
7577671000006116	Intracystic papillary carcinoma	703545003
7577961000006116	Low grade serous carcinoma	703561001
7577981000006114	High grade serous carcinoma	703563003
7578171000006114	Invasive micropapillary carcinoma of breast	703578005
7610911000006117	Triple-negative breast cancer	706970001
7610921000006113	Triple negative malignant neoplasm of breast	706970001
7615721000006119	Primary carcinoma of maxillary sinus	707347009
7615731000006116	Primary carcinoma of sphenoidal sinus	707348004
7615751000006111	Primary carcinoma of frontal sinus	707349007
7617021000006110	Primary adenosquamous carcinoma of lung	707405009
7617031000006113	Adenosquamous carcinoma of lung	707405009
7617411000006110	Primary verrucous carcinoma of larynx	707427000
7617771000006113	Primary undifferentiated carcinoma of lung	707456000
7617811000006113	Primary pleomorphic carcinoma of lung	707458004
7617821000006117	Pleomorphic carcinoma of lung	707458004
7617841000006112	Sarcomatoid carcinoma of lung	707460002
7617881000006118	Primary myoepithelial carcinoma of lung	707464006
7617921000006114	Primary adenoid cystic carcinoma of lung	707466008
7620391000006118	Primary epithelial-myoepithelial carcinoma of oropharynx	707588005
7642331000006116	Diffuse sclerosing papillary thyroid carcinoma	708971008
7642361000006113	Metastatic hepatocellular carcinoma	708973006
7645511000006110	Metastatic papillary thyroid carcinoma	709191009
7645561000006113	Metastatic thymic carcinoma	709196004
7650911000006111	Malignant carcinoid tumour of small intestine	709517003
7665481000006112	Malignant odontogenic tumour of upper jaw	710196003
7696221000006119	Prostate cancer metastatic to bone	712849003
7696231000006116	Carcinoma of prostate with bony metastases	712849003
7696241000006114	Primary malignant neoplasm of prostate metastatic to bone	712849003
7700741000006110	Malignant insulinoma	713189001
7702501000006115	Malignant meningioma of meninges of brain	713327005
7706081000006115	Malignant carcinoid tumour of rectum	713573006
7706101000006111	Malignant carcinoid tumour of kidney	713574000
7706551000006117	Invasive carcinoma of breast	713609000
7748991000006110	Epstein-Barr virus associated gastric carcinoma	716586009
7750071000006110	Extraovarian primary peritoneal carcinoma	716649003
7750091000006111	Primary peritoneal serous carcinoma	716649003
7750101000006117	Serous surface papillary carcinoma	716649003
7761601000006113	Papillary thyroid carcinoma with renal papillary neoplasia	717734005
7769431000006119	Hereditary breast and ovarian cancer syndrome	718220008
7819281000006116	Primary adnexal carcinoma of skin	721540005
7819291000006118	Primary malignant sarcoma of skin	721541009
7819591000006115	Primary malignant melanoma of vagina	721563000
7820541000006113	Malignant melanoma of oesophagus	721627007
7821621000006111	Primary cloacogenic carcinoma of anal canal	721709000
7827301000006119	Hormone sensitive prostate cancer	722103009
7827321000006112	Castration-sensitive prostate cancer	722103009
7832741000006117	Primary malignant nerve sheath neoplasm of peripheral nervous system structure	722517009
7832841000006112	Primary invasive pleomorphic lobular carcinoma of breast	722524005
7832961000006115	Primary malignant neoplasm of oesophagogastric junction	722533007
7833101000006111	Primary malignant melanoma of anal canal	722543005
7835031000006115	Primary thymic carcinoma	722670005
7835041000006113	Metastatic malignant neoplasm of meninges	722671009

7835281000006114	Primary serous carcinoma of uterine adnexa	722686003
7835561000006119	Primary malignant meningioma	722718001
7842361000006118	Squamous non-small cell lung cancer	723301009
7842371000006113	Squamous NSCLC (non-small cell lung cancer)	723301009
7850981000006110	Malignant neoplasm of lower lobe of right lung	724056005
7850991000006113	Malignant neoplasm of upper lobe of left lung	724058006
7851001000006114	Malignant neoplasm of lower lobe of left lung	724059003
7851011000006112	Malignant neoplasm of right upper lobe of lung	724060008
7856231000006115	Primary urothelial carcinoma of overlapping lesion of urinary organ	724468006
7857741000006115	Primary poorly differentiated carcinoma of thyroid gland	724552002
7881241000006113	Malignant carcinoid tumour of bronchus	726653000
7881261000006112	Malignant carcinoid tumour of colon	726654006
7954281000006116	Primary urothelial carcinoma of paraurethral gland	733135005
7954411000006112	Malignant epithelial neoplasm of bronchus	733144006
7957121000006116	Primary undifferentiated carcinoma of endometrium	733360000
7958341000006113	Collecting duct carcinoma of kidney	733470002
7987461000006110	Primary adenoid cystic carcinoma of nasopharynx	7391000119103
8009411000006119	Malignant phaeochromocytoma	21851000119103
8017201000006114	Primary invasive malignant neoplasm of female breast	45221000119105
8021331000006113	Primary small cell malignant neoplasm of lung, TNM stage 1	67811000119102
8021341000006115	Primary small cell malignant neoplasm of lung, TNM stage 2	67821000119109
8021351000006118	Primary small cell malignant neoplasm of lung, TNM stage 3	67831000119107
8021361000006116	Primary small cell malignant neoplasm of lung, TNM stage 4	67841000119103
8022211000006118	Recurrent primary malignant neoplasm of vulva	71111000119109
8025911000006117	Malignant glioma of cerebrum	87091000119101
8025931000006111	Malignant glioma of hypothalamus	87111000119109
8025941000006118	Malignant glioma of cerebellum	87121000119102
8025951000006116	Malignant glioma of central nervous system	87151000119105
8030301000006113	Primary malignant inflammatory neoplasm of female breast	96291000119105
8030471000006110	Malignant neoplasm of rectosigmoid junction metastatic to brain	96981000119102
8032841000006117	Squamous cell cancer of vagina	105121000119102
8033751000006115	Primary malignant mixed Mullerian neoplasm of endometrium	107751000119102
8033781000006111	Primary malignant clear cell neoplasm of endometrium	107771000119106
8033801000006110	Primary adenosquamous carcinoma of endometrium	107791000119107
8046551000006119	Primary malignant astrocytoma of central nervous system	147101000119108
8113461000006113	Secondary malignant neoplasm of right lung	353561000119103
8113541000006110	Secondary malignant neoplasm of left lung	353741000119106
8113551000006112	Cancer metastatic to left lung	353741000119106
8113731000006110	Primary malignant neoplasm of left kidney	354351000119105
8113741000006117	Primary malignant neoplasm of right kidney	354361000119107
8195631000006115	Lymphoedema due to malignant disease	520231000000107
8223921000006116	Colorectal cancer	722731000000100
8308571000006116	Malignant neoplasm of foot and ankle	882791000000109
8455581000006113	Infiltrating ductal carcinoma of central portion of left female breast	1080111000119108
8455631000006111	Infiltrating ductal carcinoma of upper inner quadrant of left female breast	1080151000119109
8455651000006116	Infiltrating ductal carcinoma of upper outer quadrant of left female breast	1080161000119106
8455691000006110	Infiltrating ductal carcinoma of central portion of right female breast	1080191000119104
8455751000006114	Infiltrating ductal carcinoma of upper inner quadrant of right female breast	1080231000119108
8455771000006116	Infiltrating ductal carcinoma of upper outer quadrant of right female breast	1080241000119104
8455801000006119	Infiltrating lobular carcinoma of left female breast	1080261000119100
8455901000006113	Infiltrating lobular carcinoma of right female breast	1080341000119105
8456611000006112	Malignant melanoma of left choroid	1080941000119109
8456661000006110	Malignant melanoma of right choroid	1080981000119104
8456721000006112	Malignant melanoma of skin of left lower limb	1081021000119109
8457381000006110	Recurrent primary malignant neoplasm of left female breast	1081551000119106
8457401000006110	Recurrent primary malignant neoplasm of right female breast	1081561000119108
8468321000006116	Recurrent malignant neoplasm of prostate	1098981000119101
9318441000006112	Primary malignant neoplasm of uterus	10708511000119108
9479531000006110	Secondary malignant neoplasm of bilateral adrenal glands	12246561000119100
9835031000006112	Primary malignant neoplasm of both ovaries	15635721000119108
9835051000006116	Primary malignant neoplasm of bilateral female breasts	15635801000119106
9900601000006116	Secondary malignant neoplasm of lymph node from neoplasm of female breast	16260631000119100
11904321000006120	Secondary malignant neoplasm of lymph nodes of head	94394000
11918201000006112	Malignant tumor of mediastinum	363494000
11918321000006114	Malignant tumour of peripheral nerve	254986007
11925581000006116	Malignant tumor of small intestine	363509000
11925881000006120	Malignant tumor of lung	363358000
11928591000006112	Primary malignant neoplasm of descended testis	109876001
11930221000006112	Angioimmunoblastic T-cell lymphoma	413537009
11999241000006116	Adenoid cystic carcinoma of cervix uteri	763064007
12005541000006114	Metaplastic carcinoma of breast	763479005

12023651000006120	Lymphoepithelial carcinoma	764938007
12026191000006110	Renal medullary carcinoma	765095002
12056291000006116	Germline BRCA-mutated, HER2-negative metastatic breast cancer	767444009
12107851000006114	Malignant melanoma of skin of left upper limb	352201000119105
12107951000006116	Primary malignant neoplasm of bone of right upper limb	354671000119106
12204961000006112	Metastatic colorectal cancer	94365007
12222311000006112	Malignant pleural mesothelioma	254645002
12702811000006116	Hairy cell leukaemia	54087003
12703771000006114	Primary malignant neoplasm of skin	94047004
12704581000006120	Malignant tumour of posterior margin of nasal septum and choanae	254484001
12704601000006112	Malignant tumour of upper labial mucosa	187606005
12762081000006116	Mesothelioma	62064005
12762111000006112	Malignant tumor of ureter	363458004
12762131000006116	Malignant tumor of tonsil	363393007
12762141000006110	Malignant tumor of stomach	363349007
12762151000006112	Malignant tumor of rectum	363351006
12762161000006114	Malignant tumor of head of pancreas	363419009
12762171000006120	Malignant tumor of hepatic flexure	363407001
12762191000006118	Malignant tumor of gallbladder	363353009
12762201000006116	Malignant tumor of descending colon	363409003
13490261000006116	Malignant teratoma	189847002
13493911000006118	Undifferentiated carcinoma of liver and intrahepatic biliary tract	770685009
13518981000006110	Primary differentiated carcinoma of thyroid gland	772992009
13603371000006112	Secondary malignant neoplasm of colon and/or rectum	781076008
13606871000006112	Malignant neoplasm of colon and/or rectum	781382000
13606881000006112	Colorectal cancer	781382000
13621541000006120	Solid pseudopapillary carcinoma of pancreas	782697005
13627601000006110	Malignant epithelial neoplasm of salivary gland	783155007
13632761000006116	Malignant melanoma of mucous membrane	783736003
13670801000006112	Low grade malignant glioma of brain	90811000119100
13670811000006114	Grade 4 malignant glioma of brain	90831000119105
13685731000006114	Primary malignant neoplasm of right adrenal gland	350861000119106
13685931000006112	Malignant melanoma of skin of right upper limb	351961000119109
13686021000006118	Primary malignant neoplasm of axillary tail of left female breast	353421000119109
13686031000006116	Primary malignant neoplasm of female left breast	353431000119107
13686061000006112	Primary malignant neoplasm of female right breast	353511000119101
13688471000006112	Infiltrating ductal carcinoma of axillary tail of left female breast	1080091000119100
13688491000006112	Infiltrating ductal carcinoma of lower inner quadrant of left female breast	1080121000119101
13688501000006116	Infiltrating ductal carcinoma of lower outer quadrant of left female breast	1080131000119103
13688511000006118	Infiltrating ductal carcinoma of axillary tail of right female breast	1080171000119100
13688531000006112	Infiltrating ductal carcinoma of lower inner quadrant of right female breast	1080201000119101
13688541000006120	Infiltrating ductal carcinoma of lower outer quadrant of right female breast	1080211000119103
13793501000006118	Malignant neoplasm of unknown origin	255052006
13815801000006118	Malignant neoplasm of ill-defined site	363357005
13918131000006120	Malignant lymphomatoid granulomatosis	789689004
13918581000006112	Malignant middle ear paraganglioma	789721005
13924191000006120	Malignant pituitary blastoma	816205008
13927961000006116	Infiltrating duct carcinoma of prostate	823017009
13932211000006112	Malignant melanoma with BRAF V600E mutation	830150003
13932231000006118	Anaplastic lymphoma kinase fusion oncogene positive non-small cell lung cancer	830151004
13934801000006116	Carcinomatosis of peritoneum	836274002
13934811000006116	Carcinomatosis peritonei	836274002
13935271000006114	High-grade urothelial carcinoma found on urine cytology	836304000
13955941000006112	Cholangiocarcinoma of perihilar bile duct	865951006
13957741000006118	Malignant nerve sheath neoplasm of peripheral nerve of abdomen	866069006
13957751000006116	Malignant mesenchymal neoplasm of bile duct	866070007
13957761000006120	Malignant mesenchymal neoplasm of gallbladder	866071006
13957771000006114	Malignant melanoma of orbit	866072004
13958111000006116	Malignant nerve sheath neoplasm of peripheral nerve of upper limb	866093001
13958151000006116	Malignant nerve sheath neoplasm of peripheral nerve of lower limb	866097000
13959321000006116	Primary clear cell peritoneal carcinoma	866176003
13968771000006120	Malignant ulcer	870711002
13974541000006118	Malignant neoplasm of connective and soft tissue of sacrococcygeal region	871843002
13995431000006116	Locally advanced breast cancer	1082701000112100
13995451000006110	Primary malignant neoplasm of breast with axillary lymph node invasion	1082901000112103
14062101000006118	Primary malignant neoplasm of choroid	93755007
14130631000006118	Second primary cancer	878806005
14140751000006120	Primary malignant neoplasm of left lung	890528009
14140761000006116	Primary malignant neoplasm of right lung	890529001
14140771000006112	Bilateral primary malignant neoplasm of lungs	890534002
14142241000006112	Bilateral primary malignant neoplasm of kidneys	895345000

14142361000006116	Primary malignant neoplasm of left ureter	895354002
14142371000006112	Primary malignant neoplasm of right ureter	895355001
14142411000006112	Primary malignant neoplasm of left testis	895358004
14142421000006116	Primary malignant neoplasm of right testis	895359007
14419581000006116	Malignant mastocytosis	307591004
14422531000006116	Carcinoma of pyriform fossa	1141621000
14428231000006118	Malignant neoplasm of lateral border of tongue	1144252005
14433471000006114	Adnexal carcinoma of vulva	1144945000
14442381000006114	Follicular lymphoma grade 3b	1148845007
14442461000006110	Follicular lymphoma grade 3a	1148851002
14449711000006112	Primary non-Hodgkin malignant lymphoma of vitreoretinal tract	1153347002
14457401000006112	Secondary malignant neoplasm of leptomeninges	1155991005
14461831000006120	Malignant rhabdoid tumour	1156418001
14462211000006116	Choroid plexus carcinoma	1156471001
14464461000006112	Malignant mixed Müllerian neoplasm of corpus uteri	1156808001
14464491000006116	Malignant mesenchymal neoplasm of anus	1156811000
14467081000006120	Malignant neoplasm of vertebral column region	1157063004
14472801000006112	Secondary malignant neoplasm of breast	145501000119108
14474411000006118	Primary malignant neoplasm of skin of right shoulder	352471000119101
14474441000006120	Primary malignant neoplasm of neck of pancreas	352701000119102
14474531000006112	Primary malignant neoplasm of right renal pelvis	352961000119103
14474601000006120	Primary malignant neoplasm of left renal pelvis	353111000119108
14474811000006116	Left choroidal primary malignant neoplasm	354301000119106
14475621000006112	Primary carcinoma ex pleomorphic adenoma of salivary gland	683031000119101
14475631000006112	Primary carcinoma ex pleomorphic adenoma of submandibular gland	683041000119105
14476001000006116	Primary malignant glioma of frontal lobe	686431000119100
14476021000006110	Primary malignant glioma of parietal lobe	686451000119106
14476031000006112	Primary malignant glioma of temporal lobe	686461000119108
14476041000006116	Primary malignant phaeochromocytoma of left adrenal gland	686591000119102
14476051000006118	Primary malignant phaeochromocytoma of right adrenal gland	686601000119109
14477311000006116	Secondary malignant neoplasm of left kidney	1079531000119106
14484741000006118	Right ovarian primary endometrioid carcinoma	10737781000119100
14486171000006116	Secondary malignant neoplasm of left breast	12241031000119108
14486221000006112	Bilateral secondary malignant neoplasm of breasts	12246641000119104
14487061000006110	Bilateral secondary malignant neoplasm of ovaries	15930821000119104
14488961000006110	Primary malignant gastrointestinal stromal neoplasm of colon	16636051000119104
14493221000006116	Malignant melanoma of skin of right forearm	939595491000119168
14493311000006116	Malignant melanoma of skin of left wrist	985355341000119040
14498841000006116	Primary malignant neoplasm of conjunctiva of eye	93764002
14498881000006112	Primary malignant neoplasm of retina	93987004
14498921000006116	Primary malignant neoplasm of uveal tract of eye	94128004
14505271000006110	Malignant neoplasm of vertebral column	363438000
14570101000006112	Malignant neoplasm of middle lobe of right lung	1179762006
14626941000006116	Primary malignant neoplasm of overlapping sites of cervix uteri	188180002
14677241000006112	Primary urothelial carcinoma of overlapping sites of urinary organs	724468006
14728071000006118	Primary malignant astrocytoma of brain	1196844004
14728441000006116	Primary epithelioid malignant mesothelioma of pleura	1196883002
14728591000006112	Primary malignant atypical teratoid rhabdoid neoplasm of brain	1196898000
14729451000006112	Malignant perivascular epithelioid cell neoplasm	1196998001
14731251000006114	Primary salivary gland type carcinoma of palate	1197258000
14731981000006112	Metastatic malignant melanoma to skin	1197324006
14732011000006110	Metastatic carcinoma of pelvic lymph node	1197325007
14732041000006114	Metastatic carcinoma to lymph node of head and neck	1197326008
14732131000006116	Metastatic carcinoma of abdominal lymph node	1197329001
14732231000006114	Malignant melanoma of uveal tract	1197334002
14747831000006114	Sarcomatoid urothelial carcinoma of urinary bladder	1208460000
14756931000006116	HER2-expressing (human epidermal growth factor 2-expressing) colorectal malignant neoplasm	1217010007
14765531000006112	Metastasis from malignant neoplasm of colon and/or rectum	1217692004
14789271000006112	Serous carcinoma of body of uterus	1230318000
14790901000006116	Malignant middle cerebral artery syndrome	1231168008
14831861000006112	Malignant carcinoid tumour of right lung	793197981000119168
14840411000006116	Metastatic malignant neoplasm to retroperitoneum	94628003
14841931000006114	Metastatic malignant neoplasm	128462008
14869751000006110	Metastatic malignant neoplasm to lymph node from primary malignant neoplasm of female breast	16260631000119100

Hospital episode statistics: Cancers	
**icd**	**description**
C00	Malignant neoplasm of lip
C01	Malignant neoplasm of base of tongue

C02	Malignant neoplasm of other and unspecified parts of tongue
C03	Malignant neoplasm of gum
C04	Malignant neoplasm of floor of mouth
C05	Malignant neoplasm of palate
C06	Malignant neoplasm of other and unspecified parts of mouth
C07	Malignant neoplasm of parotid gland
C08	Malignant neoplasm of other and unspecified major salivary glands
C09	Malignant neoplasm of tonsil
C10	Malignant neoplasm of oropharynx
C11	Malignant neoplasm of nasopharynx
C12	Malignant neoplasm of piriform sinus
C13	Malignant neoplasm of hypopharynx
C14	Malignant neoplasm of other and ill-defined sites in the lip, oral cavity and pharynx
C15	Malignant neoplasm of oesophagus
C16	Malignant neoplasm of stomach
C17	Malignant neoplasm of small intestine
C18	Malignant neoplasm of colon
C19	Malignant neoplasm of rectosigmoid junction
C20	Malignant neoplasm of rectum
C21	Malignant neoplasm of anus and anal canal
C22	Malignant neoplasm of liver and intrahepatic bile ducts
C23	Malignant neoplasm of gallbladder
C24	Malignant neoplasm of other and unspecified parts of biliary tract
C25	Malignant neoplasm of pancreas
C26	Malignant neoplasm of other and ill-defined digestive organs
C30	Malignant neoplasm of nasal cavity and middle ear
C31	Malignant neoplasm of accessory sinuses
C32	Malignant neoplasm of larynx
C33	Malignant neoplasm of trachea
C34	Malignant neoplasm of bronchus and lung
C37	Malignant neoplasm of thymus
C38	Malignant neoplasm of heart, mediastinum and pleura
C39	Malignant neoplasm of other and ill-defined sites in the respiratory system and intrathoracic organs
C40	Malignant neoplasm of bone and articular cartilage of limbs
C41	Malignant neoplasm of bone and articular cartilage of other and unspecified sites
C43	Malignant melanoma of skin
C44	Other malignant neoplasms of skin
C45	Mesothelioma
C46	Kaposi sarcoma
C47	Malignant neoplasm of peripheral nerves and autonomic nervous system
C48	Malignant neoplasm of retroperitoneum and peritoneum
C49	Malignant neoplasm of other connective and soft tissue
C50	Malignant neoplasm of breast
C51	Malignant neoplasm of vulva
C52	Malignant neoplasm of vagina
C53	Malignant neoplasm of cervix uteri
C54	Malignant neoplasm of corpus uteri
C55	Malignant neoplasm of uterus, part unspecified
C56	Malignant neoplasm of ovary
C57	Malignant neoplasm of other and unspecified female genital organs
C58	Malignant neoplasm of placenta
C60	Malignant neoplasm of penis
C61	Malignant neoplasm of prostate
C62	Malignant neoplasm of testis
C63	Malignant neoplasm of other and unspecified male genital organs
C64	Malignant neoplasm of kidney, except renal pelvis
C65	Malignant neoplasm of renal pelvis
C66	Malignant neoplasm of ureter
C67	Malignant neoplasm of bladder
C68	Malignant neoplasm of other and unspecified urinary organs
C69	Malignant neoplasm of eye and adnexa
C70	Malignant neoplasm of meninges
C71	Malignant neoplasm of brain
C72	Malignant neoplasm of spinal cord, cranial nerves and other parts of central nervous system
C73	Malignant neoplasm of thyroid gland
C74	Malignant neoplasm of adrenal gland
C75	Malignant neoplasm of other endocrine glands and related structures
C76	Malignant neoplasm of other and ill-defined sites
C77	Secondary and unspecified malignant neoplasm of lymph nodes
C78	Secondary malignant neoplasm of respiratory and digestive organs
C79	Secondary malignant neoplasm of other and unspecified sites

C80	Malignant neoplasm, without specification of site
C81	Hodgkin lymphoma
C82	Follicular lymphoma
C83	Non-follicular lymphoma
C84	Mature T/NK-cell lymphomas
C85	Other and unspecified types of non-Hodgkin lymphoma
C86	Other specified types of T/NK-cell lymphoma
C88	Malignant immunoproliferative diseases
C90	Multiple myeloma and malignant plasma cell neoplasms
C91	Lymphoid leukaemia
C92	Myeloid leukaemia
C93	Monocytic leukaemia
C94	Other leukaemias of specified cell type
C95	Leukaemia of unspecified cell type
C96	Other and unspecified malignant neoplasms of lymphoid, haematopoietic and related tissue
C97	Malignant neoplasms of independent (primary) multiple sites

Clinical codes for cardiovascular diseases

GOLD: clinical, referral, tests; Aurum: consultations, observations.
Process measures (monitoring, tests) not included

Additional sources of information in addition to clinical opinion and in-house code repository: MiFoot study https://www.mifoot.org.uk/; Davidson et al. 2021: https://doi.org/10.17037/DATA.00002220)

CPRD GOLD: Cardiovascular diseases		
**medcode**	**readcode**	**readterm**
8610	G76z000	Iliac artery occlusion
8443	G663.00	Brain stem stroke syndrome
51326	G63y.00	Other precerebral artery occlusion
39449	G312.00	Coronary thrombosis not resulting in myocardial infarction
33899	G575000	Cardiac arrest with successful resuscitation
39693	G31y200	Subendocardial ischaemia
1676	G3z..00	Ischaemic heart disease NOS
27951	G31..00	Other acute and subacute ischaemic heart disease
29758	G30X.00	Acute transmural myocardial infarction of unspecif site
30202	G617.00	Intracerebral haemorrhage, intraventricular
55137	G311011	MI - myocardial infarction aborted
94870	G580400	Congestive heart failure due to valvular disease
40053	G671.00	Generalised ischaemic cerebrovascular disease NOS
29643	G303.00	Acute inferoposterior infarction
14898	G305.00	Lateral myocardial infarction NOS
17734	G622.00	Subdural haematoma - nontraumatic
36178	G620.00	Extradural haemorrhage - nontraumatic
23580	G60z.00	Subarachnoid haemorrhage NOS
66388	G33z000	Status anginosus
26424	G64z400	Infarction of basal ganglia
65745	Gyu6100	[X]Other subarachnoid haemorrhage
48149	G681.00	Sequelae of intracerebral haemorrhage
37657	G362.00	Ventric septal defect/curr comp fol acut myocardal infarctn
45781	G63..00	Precerebral arterial occlusion
46112	G380.00	Postoperative transmural myocardial infarction anterior wall
105479	G39..00	Coronary microvascular disease
31060	G61X.00	Intracerebral haemorrhage in hemisphere, unspecified
41835	G384.00	Postoperative subendocardial myocardial infarction
39546	Gyu3000	[X]Other forms of angina pectoris
43451	G682.00	Sequelae of other nontraumatic intracranial haemorrhage
12804	G33z700	Stable angina
4017	G32..00	Old myocardial infarction
21195	G575100	Sudden cardiac death, so described
9913	1O1..00	Heart failure confirmed
21844	G31y300	Transient myocardial ischaemia
28554	G33zz00	Angina pectoris NOS
5942	G581.13	Impaired left ventricular function
23708	G361.00	Atrial septal defect/curr comp folow acut myocardal infarct
16517	G640.00	Cerebral thrombosis
19655	G311.14	Angina at rest
39655	G311.12	Impending infarction
1431	G311.13	Unstable angina
4656	G311.11	Crescendo angina

9696	G604.00	Subarachnoid haemorrhage from posterior communicating artery
41221	G30y200	Acute septal infarction
46316	G612.00	Basal nucleus haemorrhage
35713	G34yz00	Other specified chronic ischaemic heart disease NOS
12139	G300.00	Acute anterolateral infarction
20416	G3...12	Atherosclerotic heart disease
1792	G3...13	IHD - Ischaemic heart disease
4024	G58z.00	Heart failure NOS
17307	G311200	Angina at rest
61072	G311000	Myocardial infarction aborted
34758	G641.11	Cerebral embolus
23579	G310.00	Postmyocardial infarction syndrome
111096	Gyu6700	[X]Other specified cerebrovascular diseases
10504	G64z300	Right sided cerebral infarction
108668	Gyu6000	[X]Subarachnoid haemorrhage from other intracranial arteries
46017	G30yz00	Other acute myocardial infarction NOS
6228	G68X.00	Sequelae of stroke,not specfd as h'morrhage or infarction
105250	G341111	Mural cardiac aneurysm
3999	G340000	Single coronary vessel disease
14658	G30z.00	Acute myocardial infarction NOS
30045	G616.00	External capsule haemorrhage
47607	L440.11	CVA - cerebrovascular accident in the puerperium
97122	G673300	Vertebral artery dissection
8935	G302.00	Acute inferolateral infarction
63467	G306.00	True posterior myocardial infarction
5185	G64z111	Lateral medullary syndrome
70536	G671000	Acute cerebrovascular insufficiency NOS
101138	G583.00	Heart failure with normal ejection fraction
18912	G623.00	Subdural haemorrhage NOS
4273	G621.00	Subdural haemorrhage - nontraumatic
17872	G301100	Acute anteroseptal infarction
73961	Gyu7400	[X]Other specified peripheral vascular diseases
1430	G33..00	Angina pectoris
24446	G63y100	Cerebral infarction due to embolism of precerebral arteries
56279	L440.12	Stroke in the puerperium
62342	G615.00	Bulbar haemorrhage
13564	G613.00	Cerebellar haemorrhage
33543	G6X..00	Cerebrl infarctn due/unspcf occlusn or sten/cerebrl artrs
27977	G31yz00	Other acute and subacute ischaemic heart disease NOS
51767	G666.00	Pure sensory lacunar syndrome
20284	G62z.00	Intracranial haemorrhage NOS
17278	G58z.12	Cardiac failure NOS
1223	G58..11	Cardiac failure
29553	G366.00	Thrombosis atrium,auric append&vent/curr comp foll acute MI
24783	G3...11	Arteriosclerotic heart disease
3704	G307.00	Acute subendocardial infarction
17133	G30A.00	Mural thrombosis
5254	G340100	Double coronary vessel disease
33402	G575.12	Asystole
25407	G575.11	Cardio-respiratory arrest
7347	G311100	Unstable angina
6960	G61..11	CVA - cerebrovascular accid due to intracerebral haemorrhage
18604	G61..12	Stroke due to intracerebral haemorrhage
8837	G64..00	Cerebral arterial occlusion
36523	G311.00	Preinfarction syndrome
92036	Gyu6600	[X]Occlusion and stenosis of other cerebral arteries
9985	G64z200	Left sided cerebral infarction
2099	G575.00	Cardiac arrest
69474	G365.00	Rupture papillary muscle/curr comp fol acute myocard infarct
2760	G73zz00	Peripheral vascular disease NOS
17326	G60X.00	Subarachnoid haemorrh from intracranial artery, unspecif
3149	G64z.00	Cerebral infarction NOS
39403	G683.00	Sequelae of cerebral infarction
240	G3...00	Ischaemic heart disease
6853	G73z011	Claudication
15019	G641.00	Cerebral embolism
34633	G34y.00	Other specified chronic ischaemic heart disease
41910	G605.00	Subarachnoid haemorrhage from basilar artery
67087	G341100	Other cardiac wall aneurysm
6155	G64..13	Stroke due to cerebral arterial occlusion
5943	G73..00	Other peripheral vascular disease

40429	G301000	Acute anteroapical infarction
19201	G61X100	Right sided intracerebral haemorrhage, unspecified
68401	Gyu3200	[X]Other forms of acute ischaemic heart disease
1298	G66..11	CVA unspecified
23671	G63y000	Cerebral infarct due to thrombosis of precerebral arteries
47642	G64z100	Wallenberg syndrome
101137	G583.11	HFNEF - heart failure with normal ejection fraction
22383	G3y..00	Other specified ischaemic heart disease
10079	G580.12	Right heart failure
10154	G580.13	Right ventricular failure
27975	G641000	Cerebral infarction due to embolism of cerebral arteries
24126	G360.00	Haemopericardium/current comp folow acut myocard infarct
60692	G606.00	Subarachnoid haemorrhage from vertebral artery
29939	G600.00	Ruptured berry aneurysm
23707	G580000	Acute congestive heart failure
15661	G310.11	Dressler's syndrome
884	G581.00	Left ventricular failure
23078	G34y100	Chronic myocardial ischaemia
104275	G584.00	Right ventricular failure
12555	G671z00	Generalised ischaemic cerebrovascular disease NOS
2062	G58..00	Heart failure
107440	G619.00	Lobar cerebral haemorrhage
90572	Gyu6500	[X]Occlusion and stenosis of other precerebral arteries
40338	G611.00	Internal capsule haemorrhage
31805	G62..00	Other and unspecified intracranial haemorrhage
241	G30..00	Acute myocardial infarction
7912	G614.00	Pontine haemorrhage
34803	G30y.00	Other acute myocardial infarction
9413	G31y.00	Other acute and subacute ischaemic heart disease
59940	G364.00	Ruptur chordae tendinae/curr comp fol acute myocard infarct
30330	G309.00	Acute Q-wave infarct
5051	G61..00	Intracerebral haemorrhage
5363	G64..11	CVA - cerebral artery occlusion
569	G64..12	Infarction - cerebral
8568	G37..00	Cardiac syndrome X
15252	G64z.11	Brainstem infarction NOS
5602	G64z.12	Cerebellar infarction
25583	G574011	Cardiac arrest-ventricular fibrillation
2155	G341000	Ventricular cardiac aneurysm
72562	G353.00	Subsequent myocardial infarction of other sites
34328	G311300	Refractory angina
47637	Gyu3300	[X]Other forms of chronic ischaemic heart disease
46276	G381.00	Postoperative transmural myocardial infarction inferior wall
53745	Gyu6400	[X]Other cerebral infarction
106812	G383.00	Postoperative transmural myocardial infarction unspec site
25615	G64z000	Brainstem infarction
6827	G73..13	Peripheral ischaemia
1826	G73..12	Ischaemia of legs
27494	G74y300	Embolism and thrombosis of the iliac artery unspecified
32272	G38..00	Postoperative myocardial infarction
94482	Gyu6G00	[X]Cereb infarct due unsp occlus/stenos precerebr arteries
49882	G575z00	Cardiac arrest, unspecified
36423	G36..00	Certain current complication follow acute myocardial infarct
32671	G580100	Chronic congestive heart failure
21837	G232.00	Hypertensive heart&renal dis wth (congestive) heart failure
18125	G330000	Nocturnal angina
105317	G734.00	Peripheral arterial disease
57987	G234.00	Hyperten heart&renal dis+both(congestv)heart and renal fail
398	G580.00	Congestive heart failure
24540	G34y000	Chronic coronary insufficiency
108630	Gyu6E00	[X]Subarachnoid haemorrh from intracranial artery, unspecif
39344	G676000	Cereb infarct due cerebral venous thrombosis, nonpyogenic
3530	G73z.00	Peripheral vascular disease NOS
18842	G35..00	Subsequent myocardial infarction
38907	G73y.00	Other specified peripheral vascular disease
23481	G581.11	Asthma - cardiac
73901	Gyu6.00	[X]Cerebrovascular diseases
25842	G33z.00	Angina pectoris NOS
41677	G341z00	Aneurysm of heart NOS
31595	G610.00	Cortical haemorrhage
19412	G602.00	Subarachnoid haemorrhage from middle cerebral artery

9276	G31y000	Acute coronary insufficiency
1469	G66..00	Stroke and cerebrovascular accident unspecified
1786	G60..00	Subarachnoid haemorrhage
1414	G33z300	Angina on effort
18889	G34z000	Asymptomatic coronary heart disease
12833	G668.00	Right sided CVA
28138	G34..00	Other chronic ischaemic heart disease
91774	G341300	Acquired atrioventricular fistula of heart
62626	G30y100	Acute papillary muscle infarction
1318	G700.00	Aortic atherosclerosis
6331	G341.00	Aneurysm of heart
7320	G343.00	Ischaemic cardiomyopathy
29421	G344.00	Silent myocardial ischaemia
57315	G618.00	Intracerebral haemorrhage, multiple localized
4325	G73yz00	Other specified peripheral vascular disease NOS
18118	G311400	Worsening angina
99367	Gyu6A00	[X]Other cerebrovascular disorders in diseases CE
7696	G33z200	Syncope anginosa
96838	Gyu3400	[X]Acute transmural myocardial infarction of unspecif site
91627	Gyu6300	[X]Cerebrl infarctn due/unspcf occlusn or sten/cerebrl artrs
1678	G308.00	Inferior myocardial infarction NOS
5702	G73..11	Peripheral ischaemic vascular disease
96630	Gyu6F00	[X]Intracerebral haemorrhage in hemisphere, unspecified
1655	G340.11	Triple vessel disease of the heart
40758	G6W..00	Cereb infarct due unsp occlus/stenos precerebr arteries
32854	G30B.00	Acute posterolateral myocardial infarction
110337	Gyu6C00	[X]Sequelae of stroke,not specfd as h'morrhage or infarction
27884	G580200	Decompensated cardiac failure
46166	G35X.00	Subsequent myocardial infarction of unspecified site
7780	G667.00	Left sided CVA
27964	G582.00	Acute heart failure
5255	G581000	Acute left ventricular failure
15302	G742z00	Peripheral arterial embolism and thrombosis NOS
12986	G331.00	Prinzmetal's angina
59193	G341200	Aneurysm of coronary vessels
101733	G67A.00	Cerebral vein thrombosis
9555	G33z500	Post infarct angina
1344	G340.12	Coronary artery disease
68748	G38z.00	Postoperative myocardial infarction, unspecified
9507	G307000	Acute non-Q wave infarction
20095	G330.00	Angina decubitus
68357	G31y100	Microinfarction of heart
106897	G583.12	Heart failure with preserved ejection fraction
56007	G601.00	Subarachnoid haemorrhage from carotid siphon and bifurcation
36717	G640000	Cerebral infarction due to thrombosis of cerebral arteries
17322	G664.00	Cerebellar stroke syndrome
44740	G680.00	Sequelae of subarachnoid haemorrhage
28314	G61X000	Left sided intracerebral haemorrhage, unspecified
42331	G603.00	Subarachnoid haemorrhage from anterior communicating artery
36854	G332.00	Coronary artery spasm
12229	G30X000	Acute ST segment elevation myocardial infarction
33499	G665.00	Pure motor lacunar syndrome
11424	G580300	Compensated cardiac failure
59189	G363.00	Ruptur cardiac wall w'out haemopericard/cur comp fol ac MI
9524	G580.14	Biventricular failure
2906	G580.11	Congestive cardiac failure
16408	G32..11	Healed myocardial infarction
99991	Gyu3600	[X]Subsequent myocardial infarction of unspecified site
6116	G66..13	CVA - Cerebrovascular accident unspecified
6253	G66..12	Stroke unspecified
13571	G30..16	Thrombosis - coronary
17689	G30..17	Silent myocardial infarction
1204	G30..14	Heart attack
1677	G30..15	MI - acute myocardial infarction
2491	G30..12	Coronary thrombosis
30421	G30..13	Cardiac rupture following myocardial infarction (MI)
13566	G30..11	Attack - heart
28736	G30y000	Acute atrial infarction
32450	G33z400	Ischaemic chest pain
38609	G351.00	Subsequent myocardial infarction of inferior wall
26863	G33z600	New onset angina

45809	G350.00	Subsequent myocardial infarction of anterior wall
52517	Gyu3.00	[X]Ischaemic heart diseases
11983	G311500	Acute coronary syndrome
54535	G33z100	Stenocardia
11048	G331.11	Variant angina pectoris
109035	Gyu3500	[X]Subsequent myocardial infarction of other sites
53810	Gyu6200	[X]Other intracerebral haemorrhage
54251	G311z00	Preinfarction syndrome NOS
23892	G304.00	Posterior myocardial infarction NOS
27484	G341.11	Cardiac aneurysm
57495	G63..11	Infarction - precerebral
15754	G34z.00	Other chronic ischaemic heart disease NOS
3535	G61z.00	Intracerebral haemorrhage NOS
29902	G330z00	Angina decubitus NOS
115338	G585.00	Heart failure with reduced ejection fraction
5640	G70..00	Atherosclerosis
10562	G307100	Acute non-ST segment elevation myocardial infarction
5413	G340.00	Coronary atherosclerosis
36609	G342.00	Atherosclerotic cardiovascular disease
14897	G301z00	Anterior myocardial infarction NOS
23871	G73y100	Peripheral angiopathic disease EC NOS
5387	G301.00	Other specified anterior myocardial infarction
37947	G676.00	Nonpyogenic venous sinus thrombosis

CPRD Aurum: Cardiovascular diseases		
**medcode**	**term**	**snomedconceptID**
7845011	Unstable angina	4557003
7847015	Crescendo angina	4557003
18472010	Acute congestive heart failure	10633002
59952018	Nocturnal angina	35928006
70653017	Congestive heart failure	42343007
89332015	Atherosclerotic heart disease	443502000
94251011	Acute heart failure	56675007
94884017	Acute myocardial infarction	57054005
104563015	Cerebrovascular disease	62914000
109915012	Post-myocardial infarction syndrome	66189004
125470015	Cerebral embolism	75543006
139475013	Heart failure	84114007
139482012	Cardiac failure	84114007
141306010	Left ventricular failure	85232009
144819018	Variant angina pectoris	87343002
147247018	Chronic congestive heart failure	88805009
158118014	Cerebellar infarction	95460007
206703015	Right heart failure	128404006
216207010	Left ventricular systolic dysfunction	134401001
299342019	Lacunar infarction	230698000
299707016	Acute anterior myocardial infarction	54329005
299708014	Acute anteroapical infarction	52035003
299709018	Anterior myocardial infarction NOS	54329005
299710011	Acute posterior myocardial infarction	233838001
299711010	Acute lateral myocardial infarction	58612006
299712015	True posterior myocardial infarction	194802003
299714019	Acute myocardial infarction of inferior wall	73795002
299718016	Other acute myocardial infarction	57054005
299719012	Acute atrial infarction	194809007
299720018	Other acute myocardial infarction NOS	57054005
299721019	Acute myocardial infarction NOS	57054005
299723016	Other acute and subacute ischaemic heart disease	414545008
299742017	Coronary thrombosis not resulting in myocardial infarction	194821006
299745015	Acute coronary insufficiency	194823009
299750014	Other acute and subacute ischaemic heart disease NOS	414545008
299757012	Angina pectoris	194828000
299758019	Stenocardia	194828000
299763015	Angina decubitus NOS	59021001
299765010	Angina pectoris NOS	194828000
299776014	Chronic ischaemic heart disease	413838009
299782012	Single coronary vessel disease	194842008
299783019	Double coronary vessel disease	194843003
299800012	Other specified chronic ischaemic heart disease	413838009
299804015	Other specified chronic ischaemic heart disease NOS	413838009
299805019	Other chronic ischaemic heart disease NOS	413838009

299808017	Subsequent myocardial infarction	194856005
299811016	Subsequent myocardial infarction of anterior wall	194857001
299812011	Subsequent myocardial infarction of inferior wall	194858006
299813018	Subsequent myocardial infarction of other sites	194856005
299834013	Other specified ischaemic heart disease	414545008
299835014	Ischaemic heart disease NOS	414545008
300179017	Decompensated cardiac failure	195111005
300180019	Compensated cardiac failure	195112003
300190010	Acute left ventricular failure	195114002
300244012	Subarachnoid haemorrhage from carotid siphon and bifurcation	195155004
300253017	Subarachnoid haemorrhage from vertebral artery	195160000
300257016	Subarachnoid haemorrhage NOS	21454007
300276019	External capsule haemorrhage	195167002
300277011	Intracerebral haemorrhage intraventricular	195168007
300287010	Intracerebral haemorrhage NOS	274100004
300290016	Other and unspecified intracranial haemorrhage	62914000
300298011	Intracranial haemorrhage	1386000
300310019	Multiple and bilateral precerebral arterial occlusion	195183002
300311015	Other precerebral artery occlusion	266253001
300312010	Cerebral infarct due to thrombosis of precerebral arteries	195185009
300313017	Cerebral infarction due to embolism of precerebral arteries	195186005
300314011	Precerebral artery occlusion NOS	266253001
300321011	Cerebral infarction due to thrombosis of cerebral arteries	195189003
300322016	Cerebral infarction due to embolism of cerebral arteries	195190007
300345010	Multiple and bilateral precerebral artery syndromes	195201005
300366019	Cerebellar stroke syndrome	195213000
300370010	Left sided CVA	195216008
300371014	Right sided CVA	195217004
300374018	Other cerebrovascular disease	62914000
300379011	Generalised ischaemic cerebrovascular disease NOS	302909007
300380014	Acute cerebrovascular insufficiency	29322000
300401011	Other cerebrovascular disease OS	62914000
300402016	Other cerebrovascular disease NOS	62914000
300403014	Late effects of cerebrovascular disease	195239002
300406018	Sequelae of subarachnoid haemorrhage	195240000
300407010	Sequelae of intracerebral haemorrhage	195241001
300411016	Sequelae of cerebral infarction	195243003
300418010	Other specified cerebrovascular disease	62914000
300419019	Cerebrovascular disease NOS	62914000
300550015	Peripheral arterial embolism and thrombosis NOS	195318006
300874013	[X]Other forms of angina pectoris	194828000
300876010	[X]Other forms of acute ischaemic heart disease	414545008
300879015	[X]Other forms of chronic ischaemic heart disease	413838009
300881018	[X]Subsequent myocardial infarction of other sites	194856005
300882013	[X]Subsequent myocardial infarction of unspecified site	194856005
300935019	[X]Subarachnoid haemorrhage from other intracranial arteries	21454007
300936018	[X]Other subarachnoid haemorrhage	21454007
300956017	[X]Intracerebral haemorrhage in hemisphere unspecified	274100004
350348018	Stable angina	233819005
350350014	New onset angina	233821000
350376014	Silent myocardial infarction	233843008
395772015	Heart failure NOS	84114007
447324018	Acute Q-wave infarct	304914007
450322013	Acute non-Q wave infarction	307140009
451133011	Left sided cerebral infarction	307766002
451134017	Right sided cerebral infarction	307767006
452963015	Right ventricular thrombosis	309518001
458410010	Post infarct angina	314116003
459487012	Refractory angina	315025001
459488019	Transient myocardial ischaemia	315026000
459859010	Asymptomatic coronary heart disease	315348000
481028017	Subarachnoid haemorrhage	21454007
490972013	Right ventricular failure	367363000
493287011	Congestive cardiac failure	42343007
494260016	Impending infarction	25106000
494261017	Worsening angina	4557003
494438016	Subendocardial ischaemia	46109009
495394013	Cortical haemorrhage	49422009
496232015	Internal capsule haemorrhage	52201006
498328016	Angina at rest	59021001
502878012	Cerebellar haemorrhage	75038005

503469016	Pontine haemorrhage	7713009
510016018	Biventricular congestive heart failure	92506005
1222398015	Cerebral arterial occlusion	20059004
1235225010	Prinzmetal's angina	87343002
1488382011	Acute coronary syndrome	394659003
1488804017	Heart failure confirmed	395105005
1780491019	Acute ST segment elevation myocardial infarction	401303003
1780501013	Acute non-ST segment elevation myocardial infarction	401314000
1786197015	Coronary thrombosis	398274000
1786198013	Thrombosis - coronary	398274000
2474651019	Infarction of basal ganglia	413102000
2534664018	Ischaemic heart disease	414545008
2534674015	Chronic myocardial ischaemia	413844008
2536393012	Arteriosclerotic heart disease	443502000
2536395017	Coronary artery disease	53741008
2537480011	IHD - Ischaemic heart disease	414545008
2537483013	Chronic coronary insufficiency	413844008
2619484018	MI - Myocardial infarction aborted	194821006
2675255018	Congestive heart failure due to valvular disease	426611007
3526080018	Mural thrombus of right ventricle following acute myocardial infarction	736978009
39111000006114	Other acute and subacute ischaemic heart disease	414545008
57341000006119	Wallenberg's syndrome	78569004
67081000006119	Ventricular septal defect as current complication following acute myocardial infarction	233846000
72571000006115	Unstable angina	4557003
100681000006116	Thrombosis of atrium, auricular appendage, and ventricle as current complications following acute myocardial infarction	194868001
118831000006118	Subsequent myocardial infarction of unspecified site	194856005
122361000006113	Stroke due to cerebral arterial occlusion	230691006
122401000006115	Stroke unspecified	230690007
123441000006112	Subarachnoid haemorrh from intracranial artery, unspecif	21454007
123481000006118	Subarachnoid haemorrhage from anterior communicating artery	21454007
123491000006115	Subarachnoid haemorrhage from basilar artery	276284000
123511000006114	Subarachnoid haemorrhage from middle cerebral artery	21454007
123521000006118	Subarachnoid haemorrhage from posterior communicating artery	21454007
149551000006111	Sequelae of other nontraumatic intracranial haemorrhage	363302008
149571000006118	Sequelae of stroke, not specfd as h'morrhage or infarction	195239002
149701000006118	Sequelae/other + unspecified cerebrovascular diseases	195239002
158601000006116	Rupture of cardiac wall without haemopericardium as current complication following acute myocardial infarction	194865003
158611000006118	Rupture of chordae tendinae as current complication following acute myocardial infarction	194866002
159001000006119	Rupture of papillary muscle as current complication following acute myocardial infarction	194867006
163261000006119	Right sided intracerebral haemorrhage, unspecified	195168007
218511000000117	Infarction - cerebral	432504007
219521000000119	Attack - heart	22298006
219531000000117	MI - acute myocardial infarction	57054005
223981000000118	Cardiac failure NOS	84114007
362461000006119	[X]Acute transmural myocardial infarction of unspecif site	57054005
394541000006112	[X]Ischaemic heart diseases	414545008
416991000006112	Transient cerebral ischemia	266257000
426231000006114	[X]Sequelae of other nontraumatic intracranial haemorrhage	363302008
426321000006116	[X]Sequelae of stroke, not specfd as h'morrhage or infarction	195239002
426481000006114	[X]Sequelae/other + unspecified cerebrovascular diseases	195239002
428181000006115	Subarachnoid hemorrhage	21454007
455641000006112	Acute anterolateral myocardial infarction	70211005
455651000006114	Acute anteroseptal myocardial infarction	62695002
457531000006110	Acute inferolateral myocardial infarction	65547006
460681000006116	Acute transmural myocardial infarction of unspecif site	57054005
482811000006113	Angina at rest	59021001
482941000006119	Angina pectoris NOS	194828000
496991000006111	Atherosclerotic cardiovascular disease	72092001
498031000006112	Atrial septal defect as current complication following acute myocardial infarction	194863005
503791000006114	Basal ganglia haemorrhage	195165005
524511000006116	Brainstem stroke syndrome	195212005
524541000006117	Brain stem infarction	95457000
537751000006115	Cardiac rupture after acute myocardial infarction	233847009
542251000006112	Cerebral infarction due to cerebral venous thrombosis, non-pyogenic	195230003

542261000006114	Cerebral infarction due to occlusion of precerebral artery	125081000119106
542831000006116	Cerebral embolus	75543006
543141000006110	Cerebrl infarctn due/unspcf occlusn or sten/cerebrl artrs	20059004
543291000006110	Myocardial infarction with complication	371068009
595731000006114	Coronary atherosclerosis	443502000
605461000006117	CVA - cerebrovascular accident due to cerebral artery occlusion	230691006
605471000006112	Cerebral hemorrhage	274100004
605481000006110	CVA - cerebrovascular accident in the puerperium	275434003
605491000006113	CVA - Cerebrovascular accident	230690007
605501000006117	CVA unspecified	230690007
616081000006113	Diabetes mellitus insulin-glucose infusion in acute myocardial infarction	315287002
682481000006118	Myocardial infarction aborted	194821006
741701000006114	Hypertensive heart and renal disease with (congestive) heart failure	194779001
744901000006114	Intracerebral haemorrhage (ICH)	274100004
744921000006116	Intracerebral haemorrhage in hemisphere unspecified	274100004
746571000006116	Intracerebral haemorrhage, multiple localized	195169004
748941000006115	Left sided intracerebral haemorrhage, unspecified	274100004
789941000006117	Hypertensive heart and renal disease with both (congestive) heart failure and renal failure	194781004
800691000006113	Diffuse cerebrovascular disease	302909007
813961000006116	Haemopericardium as current complication following acute myocardial infarction	194862000
854491000006113	Unstable angina	854491000006109
865281000006115	Angioplasty of coronary artery	14201006
865301000006116	Percut angiopl coronary artery	68466008
884141000006116	Coronary thrombosis	57054005
884151000006119	Myocardial Infarction	57054005
884521000006115	Stroke/CVA - undefined	685631000000102
884531000006117	Stroke	685631000000102
884661000006110	Peripheral arterial embolism	583731000000103
931961000006117	Acute coronary syndrome	194823009
933021000006113	Angioplasty of coronary artery	582911000000104
967931000006114	Acute myocardial infarction of posterolateral wall	15990001
1573101000006112	Cerebral infarction with haemorrhagic transformation	1573101000006108
1647701000000118	Heart failure with normal ejection fraction	446221000
1661371000000112	HFNEF - heart failure with normal ejection fraction	446221000
1816101000006113	Right ventricular failure	367363000
1823891000006113	Coronary heart disease confirmed	1823891000006109
1823971000006118	Peripheral arterial disease confirmed	1823971000006102
1847121000006114	Peripheral arterial disease	399957001
2227501000000110	Heart failure with preserved ejection fraction	446221000
2504341000006116	Acute left heart failure	364006
2520541000006112	Intracranial hemorrhage	1386000
2585431000006117	Chronic left-sided congestive heart failure	5375005
2609111000000119	Non-obstructive coronary atherosclerosis	719678003
2622631000006114	Intrapontine haemorrhage	7713009
2622651000006119	Pontine hemorrhage	7713009
2660881000006116	High output heart failure	10091002
2664351000006113	Chronic right-sided heart failure	10335000
2729671000000118	Acute transmural myocardial infarction	1089471000000109
2841141000006111	SAH - Subarachnoid haemorrhage	21454007
2841151000006113	SAH - Subarachnoid hemorrhage	21454007
2841171000006115	Subarachnoid intracranial hemorrhage	21454007
2855301000006112	Myocardial infarction	22298006
2855341000006114	MI - Myocardial infarction	22298006
2855351000006111	Myocardial infarct	22298006
3166141000006114	Angina, class III	41334000
3182531000006110	Congestive heart disease	42343007
3182561000006118	CHF - Congestive heart failure	42343007
3213831000006118	Right heart failure secondary to left heart failure	44313006
3244761000006113	Hypertensive heart failure	46113002
3283871000006117	Chronic heart failure	48447003
3371411000006113	CHD - Coronary heart disease	53741008
3371421000006117	Coronary heart disease	53741008
3381601000006117	Acute myocardial infarction of anterior wall	54329005
3427201000006111	AMI - Acute myocardial infarction	57054005
3452181000006112	Acute myocardial infarction of lateral wall	58612006
3459561000006119	Acute myocardial infarction of apical-lateral wall	59063002
3499001000006116	Angina, class I	61490001
3551171000006114	Acute myocardial infarction of high lateral wall	64627002
3565871000006113	Acute myocardial infarction of inferolateral wall	65547006

3576371000006117	Postmyocardial infarction syndrome	66189004
3589241000006116	Chronic right-sided congestive heart failure	66989003
3641641000006116	Acute myocardial infarction of anterolateral wall	70211005
3655161000006110	Pericarditis secondary to acute myocardial infarction	71023004
3699921000006110	Acute inferior myocardial infarction	73795002
3745741000006117	Acute myocardial infarction of inferoposterior wall	76593002
3809001000006112	Acute right-sided congestive heart failure	80479009
3868341000006118	HF - Heart failure	84114007
3886041000006118	Left heart failure	85232009
3886061000006119	Left-sided heart failure	85232009
3886071000006114	LVF - Left ventricular failure	85232009
3886751000006118	Angina, class III	85284003
3919991000006112	Prinzmetal angina	87343002
3920001000006119	Variant angina pectoris	87343002
3920011000006116	Variant angina	87343002
3920021000006112	Coronary artery spasm angina	87343002
3974561000006115	Pleural effusion due to congestive heart failure	90727007
4005301000006110	Biventricular failure	92506005
4193361000006119	Chronic left-sided heart failure	111283005
4775931000006111	Aborted myocardial infarction	194821006
4775971000006114	Angina	194828000
4775981000006112	Cardiac angina	194828000
4776021000006118	Ischemic heart disease - angina	194828000
4776031000006115	Ischaemic heart disease - angina	194828000
4776051000006110	Single vessel coronary artery disease	194842008
4776071000006117	Two coronary vessel disease	194843003
4776081000006119	Double vessel coronary artery disease	194843003
4776121000006117	Generalised ischaemic myocardial dysfunction	194849004
4777811000006117	Basal ganglia hemorrhage	195165005
4777861000006119	Intracerebral haemorrhage with intraventricular haemorrhage	195168007
4777871000006114	Intracerebral hemorrhage with intraventricular hemorrhage	195168007
4777881000006112	Intracerebral haemorrhage, multiple localised	195169004
4777981000006117	Occlusion of basilar artery	195180004
4778181000006119	Left sided cerebral hemisphere cerebrovascular accident	195216008
4778201000006118	Right sided cerebral hemisphere cerebrovascular accident	195217004
4778311000006112	Sequelae of cerebrovascular disease	195239002
5010981000006119	Stroke	230690007
5011161000006115	Lacunar stroke	230698000
5011171000006110	LACI - Lacunar infarction	230698000
5011191000006111	Pure motor lacunar infarction	230699008
5011211000006112	Pure sensory lacunar infarction	230700009
5056201000006111	Triple vessel coronary artery disease	233817007
5056461000006113	MI - Silent myocardial infarction	233843008
5056501000006113	Post-infarction ventricular septal defect	233846000
5583101000006116	Subarachnoid haemorrhage from basilar artery aneurysm	276284000
5935321000006111	Acute Q wave myocardial infarction	304914007
6043761000006113	Refractory heart failure	314206003
6043771000006118	Non-Q wave myocardial infarction	314207007
6204381000006117	Acute right-sided heart failure	359617009
6360391000006118	Atypical angina	371807002
6546111000006118	ACS - Acute coronary syndrome	394659003
6546751000006118	First myocardial infarction	394710008
6601121000006118	Coronary artery thrombosis	398274000
6601131000006115	CT - Coronary thrombosis	398274000
6632221000006112	Peripheral arterial occlusive disease	399957001
6632231000006110	PAOD - Peripheral arterial occlusive disease	399957001
6651221000006117	STEMI - ST elevation myocardial infarction	401303003
6651391000006114	NSTEMI - Non-ST segment elevation MI	401314000
6837051000006119	Basal ganglion stroke	413102000
6837061000006117	Basal ganglion infarct	413102000
6841231000006113	Acute ischaemic heart disease	413439005
6847761000006118	Chronic ischemic heart disease	413838009
6860251000006115	Ischemic heart disease	414545008
6860261000006118	IHD - Ischemic heart disease	414545008
6914191000006115	Systolic heart failure	417996009
6914971000006118	Myocardial infarction in recovery phase	418044006
6919191000006119	Diastolic heart failure	418304008
7025691000006110	Decompensated chronic heart failure	424404003
7056281000006118	Congestive heart failure due to left ventricular systolic dysfunction	426263006
7093091000006111	Recent myocardial infarction	428752002
7105241000006116	Typical angina	429559004

7107851000006112	Anterior myocardial infarction on electrocardiogram	429731003
7250571000006113	Chronic systolic heart failure	441481004
7251291000006119	Chronic diastolic heart failure	441530006
7275961000006118	Acute on chronic diastolic heart failure	443344007
7278491000006111	Atherosclerosis of coronary artery	443502000
7321121000006119	Heart failure with preserved ejection fraction	446221000
7507301000006110	Acute exacerbation of chronic congestive heart failure	698296002
7510341000006110	Symptomatic congestive heart failure	698594003
7571581000006113	Acute anterior ST segment elevation myocardial infarction	703164000
7571601000006115	Acute STEMI (ST elevation myocardial infarction) of anterior wall	703164000
7571611000006117	Acute ST segment elevation myocardial infarction of anterior wall involving right ventricle	703165004
7572221000006117	Subsequent ST segment elevation myocardial infarction of inferior wall	703209002
7572251000006114	Subsequent ST segment elevation myocardial infarction of anterior wall	703210007
7572291000006115	Subsequent STEMI (ST elevation myocardial infarction)	703211006
7572301000006119	Acute myocardial infarction during procedure	703212004
7572321000006112	Acute ST segment elevation myocardial infarction of inferior wall	703213009
7572331000006110	Acute STEMI (ST elevation myocardial infarction) of inferior wall	703213009
7572341000006117	Acute inferior ST segment elevation myocardial infarction	703213009
7572861000006110	Acute myocardial infarction of anterior wall involving right ventricle	703252002
7572881000006117	Acute ST segment elevation myocardial infarction of inferior wall involving right ventricle	703253007
7573171000006116	Heart failure with reduced ejection fraction	703272007
7573181000006118	Heart failure with reduced ejection fraction due to coronary artery disease	703273002
7573191000006115	Heart failure with reduced ejection fraction due to myocarditis	703274008
7573211000006119	Heart failure with reduced ejection fraction due to heart valve disease	703276005
7574041000006111	Mitral valve regurgitation due to acute myocardial infarction	703326006
7574081000006117	Mitral valve regurgitation due to acute myocardial infarction without papillary muscle and chordal rupture	703328007
7574481000006111	Subsequent non-ST segment elevation myocardial infarction	703360004
7574491000006114	Subsequent NSTEMI (non-ST segment elevation myocardial infarction)	703360004
7703511000006110	Subacute ischaemic heart disease	713405002
7848541000006112	Arrhythmia as current complication following acute myocardial infarction	723860000
7966931000006114	Angina associated with type 2 diabetes mellitus	791000119109
8004261000006118	Acute myocardial infarction due to left coronary artery occlusion	17531000119105
8011041000006111	Acute myocardial infarction due to right coronary artery occlusion	23311000119105
8011111000006111	Congestive heart failure with right heart failure	23341000119109
8024001000006117	Acute coronary artery occlusion not resulting in myocardial infarction	78741000119103
8030311000006111	Exacerbation of congestive heart failure	96311000119109
8048031000006119	Peripheral arterial insufficiency	153911000119104
8048061000006111	Chronic combined systolic and diastolic heart failure	153941000119100
8087751000006116	Acute ST segment elevation myocardial infarction involving left anterior descending coronary artery	285981000119103
9843951000006116	Acute ST segment elevation myocardial infarction of posterolateral wall	15712841000119100
9843961000006118	Acute ST segment elevation myocardial infarction of anterolateral wall	15712881000119104
9843981000006112	Acute ST segment elevation myocardial infarction of lateral wall	15712921000119104
9843991000006114	Acute ST segment elevation myocardial infarction of anteroseptal wall	15712961000119108
9844011000006120	Acute ST segment elevation myocardial infarction of posterior wall	15713041000119104
9844051000006118	Acute ST segment elevation myocardial infarction due to right coronary artery occlusion	15713121000119104
9844071000006112	Acute ST segment elevation myocardial infarction of septum	15713161000119100
9868461000006110	Unstable angina co-occurrent and due to coronary arteriosclerosis	15960061000119102
9868481000006116	Angina co-occurrent and due to coronary arteriosclerosis	15960141000119102
9868571000006116	Mural thrombus of left ventricle following acute myocardial infarction	15960981000119104
12122331000006116	Acute ST segment elevation myocardial infarction of inferolateral wall	12238111000119106
12122341000006114	Acute ST segment elevation myocardial infarction of inferoposterior wall	12238151000119108
12223101000006118	Intracerebral haemorrhage	274100004
12223111000006116	Intracerebral hemorrhage	274100004
12223121000006112	ICH - intracerebral haemorrhage	274100004
12223131000006114	ICH - intracerebral hemorrhage	274100004

12276271000006112	Progressive angina	371806006
13910671000006110	Heart failure with mid range ejection fraction	788950000
13930031000006112	Delayed postmyocardial infarction pericarditis	827164008
13935161000006114	Acute myocardial infarction of right ventricle	836293000
13935211000006116	Acute myocardial infarction of inferolateral wall with posterior extension	836295007
13941091000006116	Acute ST segment elevation myocardial infarction due to proximal left anterior descending coronary artery occlusion	840309000
13941111000006112	Acute STEMI (ST elevation myocardial infarction) due to proximal LAD (left anterior descending) coronary artery occlusion	840309000
13941141000006112	Acute ST segment elevation myocardial infarction due to mid left anterior descending coronary artery occlusion	840312002
13941161000006110	Acute STEMI (ST elevation myocardial infarction) due to mid LAD (left anterior descending) coronary artery occlusion	840312002
13941211000006118	Acute ST segment elevation myocardial infarction due to distal left anterior descending coronary artery occlusion	840316004
13941231000006112	Acute STEMI (ST elevation myocardial infarction) due to distal LAD (left anterior descending) coronary artery occlusion	840316004
13944841000006116	Peripheral arterial disease	840580004
13944851000006116	Peripheral arterial vascular disease	840580004
13945211000006116	Acute ST segment elevation myocardial infarction due to occlusion of anterior descending branch of left coronary artery	840609007
13945981000006116	Acute ST segment elevation myocardial infarction due to occlusion of septal branch of anterior descending branch of left coronary artery	840680009
13947721000006112	Acute ST segment elevation myocardial infarction due to occlusion of intermediate artery	846683001
13961351000006116	Acute STEMI (ST elevation myocardial infarction) due to distal RCA (right coronary artery) occlusion	868217004
13961381000006112	Acute STEMI (ST elevation myocardial infarction) due to mid RCA (right coronary artery) occlusion	868220007
13961431000006116	Acute STEMI (ST elevation myocardial infarction) due to AM (acute marginal) branch of RCA (right coronary artery) occlusion	868224003
13961451000006112	Acute STEMI (ST elevation myocardial infarction) due to PDA (posterior descending artery) branch of RCA (right coronary artery) occlusion	868225002
13961461000006112	Acute ST segment elevation myocardial infarction due to occlusion of posterior lateral branch of right coronary artery	868226001
13961471000006118	Acute STEMI (ST elevation myocardial infarction) due to PL (posterolateral) branch of RCA (right coronary artery) occlusion	868226001
14015031000006120	Myocardial infarction due to demand ischaemia	16837681000119104
14015051000006114	Acute on chronic right-sided congestive heart failure	16838951000119100
14057221000006112	Rupture of ventricle due to acute myocardial infarction	30277009
14132941000006118	Myocardial infarction with non-obstructive coronary artery	879955009
14132951000006116	MINOCA - myocardial infarction with non-obstructive coronary artery	879955009
14145371000006116	Acute myocardial infarction due to occlusion of circumflex branch of left coronary artery	896689003
14145391000006116	Acute ST segment elevation myocardial infarction due to occlusion of circumflex branch of left coronary artery	896691006
14145451000006116	Acute STEMI (ST segment elevation myocardial infarction) of apex of heart	896696001
14194701000006116	Myocardial infarction due to atherothrombotic coronary artery disease	726499301000119168
14194711000006116	Type 1 myocardial infarction	726499301000119168
14428131000006112	Mitral valve regurgitation due to acute myocardial infarction	1142308000
14488861000006120	Supraventricular tachycardia following acute myocardial infarction	16415081000119104
14546511000006116	Postmyocardial infarction pericardial effusion	1163420005
14742011000006112	Acute anterior non-ST segment elevation myocardial infarction	1204155000
14752451000006116	Ventricular thrombus following acute myocardial infarction	1208867006

Hospital episode statistics: Cardiovascular diseases	
**icd**	**description**
I60	Subarachnoid haemorrhage
I61	Intracerebral haemorrhage
I62	Other nontraumatic intracranial haemorrhage
I63	Cerebral infarction
I64	Stroke, not specified as haemorrhage or infarction
I69	Sequelae of cerebrovascular disease (EXCEPT I69.8)
I20	Angina pectoris
I21	Acute myocardial infarction
I22	Subsequent myocardial infarction

I23	Certain current complications following acute myocardial infarction
I24	Other acute ischaemic heart diseases
I25	Chronic ischaemic heart disease
I50	Heart failure
I11.0	Hypertensive heart disease with (congestive) heart failure
I13.2	Hypertensive heart and renal disease with both (congestive) heart failure and renal failure
I13.0	Hypertensive heart and renal disease with (congestive) heart failure
I73.9	Peripheral arterial disease

Clinical codes for cerebral palsy

GOLD: clinical, referral, tests; Aurum: consultations, observations.

CPRD GOLD: Cerebral palsy		
**medcode**	**readcode**	**readterm**
2019	F234.00	Infantile hemiplegia NOS
2069	F23..00	Congenital cerebral palsy
5512	F230100	Cerebral palsy with spastic diplegia
5560	F23..12	Infantile cerebral palsy
12666	F23yz00	Other infantile cerebral palsy NOS
15530	F23..11	Congenital spastic cerebral palsy
16956	G669.00	Cerebral palsy, not congenital or infantile, acute
16977	F137000	Athetoid cerebral palsy
21249	F232.00	Congenital quadriplegia
21548	F23y000	Ataxic infantile cerebral palsy
25324	F230.00	Congenital diplegia
25570	F23y200	Spastic cerebral palsy
25777	F137y00	Other specified symptomatic torsion dystonia
27966	F231.00	Congenital hemiplegia
28306	F23z.00	Congenital cerebral palsy NOS
33925	F233.00	Congenital monoplegia
37160	F230000	Congenital paraplegia
39971	F23..13	Littles disease
42406	F137.12	Athetosis - congenital
45551	F230z00	Congenital diplegia NOS
48126	F232.11	Tetraplegia - congenital
49967	F23y300	Dyskinetic cerebral palsy
52659	F23y400	Ataxic diplegic cerebral palsy
53178	F23y.00	Other congenital cerebral palsy
53755	Fyu9.00	[X]Cerebral palsy and other paralytic syndromes
55593	F233.11	Congenital spastic foot
61219	F23..14	Cerebral atonia
62081	F137z00	Symptomatic torsion dystonia NOS
64561	F137011	Vogt's disease
66314	F137111	Congenital athetosis
73943	F137.11	Athetoid cerebral palsy
90520	Fyu9000	[X]Other infantile cerebral palsy
92598	F137100	Double athetosis
95132	F23y500	Worster-Drought syndrome
99040	F230.11	Paraplegia - congenital
100627	F23y511	Congenital suprabulbar paresis
104498	F2B..00	Cerebral palsy
104580	F2B0.00	Spastic quadriplegic cerebral palsy
104654	F2Bz.00	Cerebral palsy NOS
104775	F230111	Spastic diplegic cerebral palsy
104782	F2By.00	Other cerebral palsy
104828	F23y100	Flaccid infantile cerebral palsy
105133	F2B1.00	Spastic hemiplegic cerebral palsy
107551	F23y600	Choreoathetoid cerebral palsy
112736	F2B2.00	Bilateral spastic cerebral palsy
16956	G669.00	Cerebral palsy, not congenital or infantile, acute

CPRD Aurum: Cerebral palsy		
**medcode**	**term**	**snomedconceptID**
27369019	Double athetosis	16171003
72531016	Infantile hemiplegia	1593000
124598014	Athetoid cerebral palsy	75019001
124599018	Vogt's disease	193497004
206411014	Cerebral palsy	128188000
297087017	Other specified symptomatic torsion dystonia	55776008

297088010	Symptomatic torsion dystonia NOS	55776008
297221013	Congenital paraplegia	192949002
297223011	Congenital diplegia NOS	58193001
297232013	Infantile hemiplegia NOS	1593000
297233015	Other congenital cerebral palsy	128188000
297234014	Ataxic infantile cerebral palsy	278512001
297238012	Other infantile cerebral palsy NOS	128188000
297239016	Congenital cerebral palsy NOS	128188000
297977018	Vogt's disease	193497004
299393011	[X]Cerebral palsy and other paralytic syndromes	408371000000100
299394017	[X]Other infantile cerebral palsy	128188000
345771011	Infantile cerebral palsy	128188000
345773014	Spastic cerebral palsy	230773005
345779013	Congenital spastic foot	230779009
345780011	Dyskinetic cerebral palsy	230780007
411453017	Congenital cerebral palsy	128188000
411454011	Congenital spastic cerebral palsy	230773005
411455012	Congenital quadriplegia	275468009
411456013	Congenital diplegia	58193001
450600011	Congenital athetosis	307355007
450601010	Athetosis - congenital	307355007
451123015	Cerebral palsy, not congenital or infantile, acute	307756005
497600015	Congenital monoplegia	56409008
2475877015	Ataxic diplegic cerebral palsy	58193001
3509879014	Neuromuscular scoliosis due to cerebral palsy	330471000119101
110721000006114	Tetraplegia - congenital	275468009
223801000000115	Paraplegia - congenital	192949002
497021000006113	Athetoid cerebral palsy	75019001
542541000006112	Cerebral atonia	128188000
543071000006115	Cerebral palsy with spastic diplegia	230773005
551481000000119	Congenital suprabulbar paresis	716335003
557181000000116	Worster Drought syndrome	716335003
740431000006115	Littles disease	128188000
761411000006111	Flaccid infantile cerebral palsy	192958009
1815931000006114	Spastic diplegic cerebral palsy	58193001
1815951000006119	Spastic quadriplegic cerebral palsy	48721008
1815971000006112	Other cerebral palsy	128188000
1815981000006110	Cerebral palsy NOS	128188000
2122001000000116	Spastic hemiplegic cerebral palsy	813921000000104
2283041000000116	Choreo-athetotic cerebral palsy	885831000000109
2283831000000117	Choreoathetoid cerebral palsy	885831000000109
2326271000000114	Bilateral spastic cerebral palsy	904531000000100
3200341000006112	Hemiplegic cerebral palsy	43486001
3200351000006114	Congenital hemiplegia	43486001
3288331000006116	Quadriplegic cerebral palsy	48721008
3416421000006111	Monoplegic cerebral palsy	56409008
3445431000006112	Diplegic cerebral palsy	58193001
3445451000006117	Spastic diplegic cerebral palsy	58193001
4405191000006110	Cerebral palsy (CP)	128188000
4769391000006119	Hypotonic cerebral palsy	192958009
5012421000006114	Dystonic/rigid cerebral palsy	230781006
5614181000006116	Ataxic cerebral palsy	278512001
7557921000006113	Dystonic cerebral palsy	702315006
7557961000006119	Bilateral cerebral palsy	702319000
7557971000006114	Bilateral spastic cerebral palsy	702319000
12465281000006112	[X]Cerebral palsy and other paralytic syndromes	408371000000100

Hospital episode statistics: Cerebral palsy	
**icd**	**description**
G80	Cerebral palsy

Clinical codes for chronic kidney diseases

GOLD: clinical, referral, tests; Aurum: consultations, observations.

CPRD GOLD: Chronic kidney diseases		
medcode	readcode	readterm
7804	K02..00	Chronic glomerulonephritis
10809	K021.00	Chronic membranous glomerulonephritis
65064	K023.00	Chronic rapidly progressive glomerulonephritis
47672	K01x400	Nephrotic syndrome in systemic lupus erythematosus
25055	K100300	Chronic pyonephrosis
22205	K01x411	Lupus nephritis
95188	1Z1C.11	CKD stage 3 without proteinuria
95180	1Z1F.11	CKD stage 3B with proteinuria
99312	1Z1H.11	CKD stage 4 with proteinuria
95406	1Z1J.00	Chronic kidney disease stage 4 without proteinuria
9840	K010.00	Nephrotic syndrome with proliferative glomerulonephritis
23913	K014.00	Nephrotic syndrome, minor glomerular abnormality
95508	1Z1K.00	Chronic kidney disease stage 5 with proteinuria
95177	1Z1G.00	Chronic kidney disease stage 3B without proteinuria
57926	K013.12	Steroid sensitive nephrotic syndrome
65400	K02y300	Chronic diffuse glomerulonephritis
63786	K01w.00	Congenital nephrotic syndrome
97587	1Z1J.11	CKD stage 4 without proteinuria
97683	1Z1L.11	CKD stage 5 without proteinuria
27427	K01z.00	Nephrotic syndrome NOS
4654	K100.00	Chronic pyelonephritis
99644	K012.00	Nephrotic syndrome+membranoproliferative glomerulonephritis
19316	K016.00	Nephrotic syndrome, diffuse membranous glomerulonephritis
10647	K02..11	Nephritis - chronic
11875	K02..12	Nephropathy - chronic
94965	1Z15.00	Chronic kidney disease stage 3A
109980	1Z1a.00	CKD with GFR category G4 & albuminuria category A1
110626	1Z1c.00	CKD with GFR category G4 & albuminuria category A3
109981	1Z1e.00	CKD with GFR category G5 & albuminuria category A2
72303	K01w000	Finnish nephrosis syndrome
71124	7L1A300	Haemofiltration
17365	K01B.00	Nephrotic syndrome, diffuse crescentic glomerulonephritis
56987	K01A.00	Nephrotic syndrome, dense deposit disease
95408	1Z1D.00	Chronic kidney disease stage 3A with proteinuria
95178	1Z1F.00	Chronic kidney disease stage 3B with proteinuria
95122	1Z1H.00	Chronic kidney disease stage 4 with proteinuria
40349	K013.11	Lipoid nephrosis
99160	1Z1K.11	CKD stage 5 with proteinuria
100633	1Z1G.11	CKD stage 3B without proteinuria
58750	K01x300	Nephrotic syndrome in polyarteritis nodosa
95405	1Z1L.00	Chronic kidney disease stage 5 without proteinuria
50472	K018.00	Nephrotic syn,difus endocapilary proliftv glomerulonephritis
8330	K0D..00	End-stage renal disease
57568	K100100	Chronic pyelonephritis with medullary necrosis
12479	1Z13.00	Chronic kidney disease stage 4
28684	G233.00	Hypertensive heart and renal disease with renal failure
109904	1Z1b.00	CKD with GFR category G4 & albuminuria category A2
21989	K019.00	Nephrotic syn,diffuse mesangiocapillary glomerulonephritis
53852	K05..12	End stage renal failure
10081	K05..11	Chronic uraemia
95175	1Z1E.00	Chronic kidney disease stage 3A without proteinuria
47922	K01x000	Nephrotic syndrome in amyloidosis
110133	1Z1d.00	CKD with GFR category G5 & albuminuria category A1
2471	K01x100	Nephrotic syndrome in diabetes mellitus
109657	1Z1Y.00	CKD with GFR category G3b & albuminuria category A2
95176	1Z1E.11	CKD stage 3A without proteinuria
110467	1Z1f.00	CKD with GFR category G5 & albuminuria category A3
15097	K02z.00	Chronic glomerulonephritis NOS
95571	1Z1D.11	CKD stage 3A with proteinuria
105151	K055.00	Chronic kidney disease stage 5
512	K05..00	Chronic renal failure
12585	1Z14.00	Chronic kidney disease stage 5
95179	1Z16.00	Chronic kidney disease stage 3B
104619	K053.00	Chronic kidney disease stage 3

12566	1Z12.00	Chronic kidney disease stage 3
104963	K054.00	Chronic kidney disease stage 4
57987	G234.00	Hyperten heart&renal dis+both(congestv)heart and renal fail
109905	1Z1W.00	CKD with GFR category G3a & albuminuria category A3
34998	K020.00	Chronic proliferative glomerulonephritis
22852	K015.00	Nephrotic syndrome, focal and segmental glomerular lesions
60960	K02y.00	Other chronic glomerulonephritis
21947	K017.00	Nephrotic syn difus mesangial prolifertiv glomerulonephritis
45499	K01x111	Kimmelstiel - Wilson disease
63615	K02yz00	Other chronic glomerulonephritis NOS
104981	K05..13	Chronic kidney disease
109805	1Z1V.00	CKD with GFR category G3a & albuminuria category A2
97758	K02y000	Chronic glomerulonephritis + diseases EC
61494	K022.00	Chronic membranoproliferative glomerulonephritis
94793	1Z1B.00	Chronic kidney disease stage 3 with proteinuria
6712	K050.00	End stage renal failure
109990	1Z1Z.00	CKD with GFR category G3b & albuminuria category A3
1803	K011.00	Nephrotic syndrome with membranous glomerulonephritis
99631	K100000	Chronic pyelonephritis without medullary necrosis
95123	1Z1C.00	Chronic kidney disease stage 3 without proteinuria
48855	K100500	Chronic obstructive pyelonephritis
48111	K100z00	Chronic pyelonephritis NOS
109963	1Z1X.00	CKD with GFR category G3b & albuminuria category A1
95145	1Z1B.11	CKD stage 3 with proteinuria
94373	K01y.00	Nephrotic syndrome with other pathological kidney lesions
29634	K013.00	Nephrotic syndrome with minimal change glomerulonephritis
109804	1Z1T.00	CKD with GFR category G3a & albuminuria category A1
2999	K01..00	Nephrotic syndrome
4669	K02y200	Chronic focal glomerulonephritis

CPRD Aurum: Chronic kidney diseases		
**medcode**	**term**	**snomedconceptID**
35171019	Chronic glomerulonephritis	20917003
74720014	Lipoid nephrosis	44785005
81307015	Congenital nephrotic syndrome	48796009
86957016	Nephrotic syndrome	52254009
105227013	Chronic pyelonephritis	63302006
114310015	Lupus nephritis	68815009
299673010	Hypertensive heart and renal disease with renal failure	194780003
303825013	Nephrotic syndrome with proliferative glomerulonephritis	197589005
303826014	Nephrotic syndrome with membranous glomerulonephritis	197590001
303832016	Nephrotic syndrome, minor glomerular abnormality	197593004
303833014	Nephrotic syndrome, focal and segmental glomerular lesions	197594005
303834015	Nephrotic syndrome, diffuse membranous glomerulonephritis	197595006
303838017	Nephrotic syndrome, dense deposit disease	197599000
303839013	Nephrotic syndrome, diffuse crescentic glomerulonephritis	197600002
303845017	Nephrotic syndrome in amyloidosis	197604006
303846016	Nephrotic syndrome in diabetes mellitus	197605007
303847013	Kimmelstiel - Wilson disease	197605007
303849011	Nephrotic syndrome in polyarteritis nodosa	197607004
303854019	Nephrotic syndrome with other pathological kidney lesions	52254009
303855018	Nephrotic syndrome NOS	52254009
303863017	Chronic rapidly progressive glomerulonephritis	236392004
303864011	Other chronic glomerulonephritis	20917003
303867016	Chronic focal glomerulonephritis	197618004
303868014	Chronic diffuse glomerulonephritis	197619007
303869018	Other chronic glomerulonephritis NOS	20917003
303870017	Chronic glomerulonephritis NOS	20917003
304060010	Chronic pyelonephritis without medullary necrosis	197760006
304061014	Chronic pyelonephritis with medullary necrosis	197761005
304063012	Chronic pyonephrosis	197763008
304067013	Chronic pyelonephritis NOS	63302006
349998013	Haemofiltration	233581009
354330018	Chronic obstructive pyelonephritis	236379002
396706011	Nephrotic syndrome with minimal change glomerulonephritis	266549004
509108014	Chronic uraemia	90688005

509111010	Chronic renal failure	90688005
2767154014	Chronic kidney disease stage 5	433146000
2767385013	Chronic kidney disease stage 4	431857002
2771041011	Chronic kidney disease	709044004
2773184015	Chronic kidney disease stage 3	433144002
304071000000115	Chronic kidney disease stage 3	433144002
304091000000116	Chronic kidney disease stage 4	431857002
304111000000114	Chronic kidney disease stage 5	433146000
554481000006111	Chronic glomerulonephritis + diseases EC	197616000
555201000006116	Chronic membranous glomerulonephritis	77182004
556211000006112	Chronic proliferative glomerulonephritis	197613008
557811000000119	Chronic kidney disease stage 3A	700378005
557831000000110	Chronic kidney disease stage 3B	700379002
595811000000117	Chronic kidney disease stage 3 with proteinuria	324251000000105
595871000000110	Chronic kidney disease stage 3 without proteinuria	324281000000104
595931000000116	Chronic kidney disease stage 3A with proteinuria	324311000000101
595991000000115	Chronic kidney disease stage 3A without proteinuria	324341000000100
596051000000115	Chronic kidney disease stage 3B with proteinuria	324371000000106
596131000000114	Chronic kidney disease stage 3B without proteinuria	324411000000105
596191000000110	Chronic kidney disease stage 4 with proteinuria	324441000000106
596261000000111	Chronic kidney disease stage 4 without proteinuria	324471000000100
596321000000110	Chronic kidney disease stage 5 with proteinuria	324501000000107
596401000000111	Chronic kidney disease stage 5 without proteinuria	324541000000105
618431000000114	CKD stage 3 with proteinuria	324251000000105
618441000000117	CKD stage 3 without proteinuria	324281000000104
618451000000119	CKD stage 3A with proteinuria	324311000000101
618461000000116	CKD stage 3A without proteinuria	324341000000100
618471000000111	CKD stage 3B with proteinuria	324371000000106
618481000000113	CKD stage 3B without proteinuria	324411000000105
618491000000110	CKD stage 4 with proteinuria	324441000000106
618501000000116	CKD stage 4 without proteinuria	324471000000100
618511000000119	CKD stage 5 with proteinuria	324501000000107
618521000000113	CKD stage 5 without proteinuria	324541000000105
641471000006117	End stage renal failure	46177005
641491000006116	End-stage renal disease	46177005
677561000006119	Nephrotic syndrome in systemic lupus erythematosus	68815009
677941000006114	Nephritis - chronic	197707007
678061000006111	Nephropathy - chronic	90708001
994421000006111	Chronic kidney disease stage 3	994421000006107
994441000006116	Chronic kidney disease stage 5	994441000006100
1823941000006114	Chronic kidney disease confirmed	1823941000006105
1940501000006119	CKD G4A1 - chronic kidney disease with glomerular filtration rate category G4 and albuminuria category A1	950181000000106
1940511000006116	CKD G4A2 - chronic kidney disease with glomerular filtration rate category G4 and albuminuria category A2	950211000000107
1940521000006112	CKD G4A3 - chronic kidney disease with glomerular filtration rate category G4 and albuminuria category A3	950231000000104
1940531000006110	CKD G5A1 - chronic kidney disease with glomerular filtration rate category G5 and albuminuria category A1	950251000000106
1940541000006117	CKD G5A2 - chronic kidney disease with glomerular filtration rate category G5 and albuminuria category A2	950291000000103
1940551000006115	CKD G5A3 - chronic kidney disease with glomerular filtration rate category G5 and albuminuria category A3	950311000000102
1940621000006111	CKD G3aA1 - chronic kidney disease with glomerular filtration rate category G3a and albuminuria category A1	949881000000106
1940631000006114	CKD G3aA2 - chronic kidney disease with glomerular filtration rate category G3a and albuminuria category A2	949901000000109
1940641000006116	CKD G3aA3 - chronic kidney disease with glomerular filtration rate category G3a and albuminuria category A3	949921000000100
1940651000006119	CKD G3bA1 - chronic kidney disease with glomerular filtration rate category G3b and albuminuria category A1	950061000000103
1940661000006117	CKD G3bA2 - chronic kidney disease with glomerular filtration rate category G3b and albuminuria category A2	950081000000107
1940671000006112	CKD G3bA3 - chronic kidney disease with glomerular filtration rate category G3b and albuminuria category A3	950101000000101
7147781000006115	CKD stage 4	431857002
7174791000006111	CKD stage 3	433144002
7174851000006111	CKD stage 5	433146000

7615331000006115	Anaemia in chronic kidney disease	707323002
7615361000006112	Anemia in chronic kidney disease	707323002
7643311000006115	CKD - chronic kidney disease	709044004
7966611000006114	Chronic kidney disease stage 5 due to type 2 diabetes mellitus	711000119100
7966641000006113	Chronic kidney disease stage 4 due to type 2 diabetes mellitus	721000119107
7966691000006116	Chronic kidney disease stage 3 due to type 2 diabetes mellitus	731000119105
8027451000006119	Chronic kidney disease stage 3 due to type 1 diabetes mellitus	90741000119107
8027471000006112	Chronic kidney disease stage 4 due to type 1 diabetes mellitus	90751000119109
8027491000006113	Chronic kidney disease stage 5 due to type 1 diabetes mellitus	90761000119106
8030421000006114	Hypertensive heart AND chronic kidney disease stage 4	96721000119103
8030431000006112	Hypertensive heart AND chronic kidney disease stage 3	96731000119100
8040661000006119	Chronic kidney disease stage 4 due to hypertension	129151000119102
8040671000006114	Chronic kidney disease stage 5 due to hypertension	129161000119100
8040681000006112	Chronic kidney disease stage 3 due to hypertension	129171000119106
8044011000006117	Hypertension in chronic kidney disease stage 5 due to type 2 diabetes mellitus	140101000119109
8044061000006119	Hypertension in chronic kidney disease stage 3 due to type 2 diabetes mellitus	140121000119100
8087071000006112	Chronic kidney disease stage 3 due to benign hypertension	284991000119104
8104231000006115	CKD (chronic kidney disease) stage 3 with proteinuria	324251000000105
8104261000006112	CKD (chronic kidney disease) stage 3 without proteinuria	324281000000104
8104301000006115	CKD (chronic kidney disease) stage 3A with proteinuria	324311000000101
8104381000006112	CKD (chronic kidney disease) stage 3B with proteinuria	324371000000106
8104451000006111	CKD (chronic kidney disease) stage 4 with proteinuria	324441000000106
8104481000006115	CKD (chronic kidney disease) stage 4 without proteinuria	324471000000100
8104511000006111	CKD (chronic kidney disease) stage 5 with proteinuria	324501000000107
8104551000006112	CKD (chronic kidney disease) stage 5 without proteinuria	324541000000105
8212401000006115	Anaemia co-occurrent and due to chronic kidney disease stage 3	691421000119108
8345931000006110	Chronic kidney disease with glomerular filtration rate category G3a and albuminuria category A3	949921000000100
8346001000006110	Chronic kidney disease with glomerular filtration rate category G3b and albuminuria category A1	950061000000103
8346021000006117	Chronic kidney disease with glomerular filtration rate category G3b and albuminuria category A2	950081000000107
8346041000006112	Chronic kidney disease with glomerular filtration rate category G3b and albuminuria category A3	950101000000101
12704471000006116	End-stage renal disease	46177005
14174321000006116	Chronic kidney disease stage 5 due to drug induced diabetes mellitus	368461000119103

Hospital episode statistics: Chronic kidney diseases	
**icd**	**description**
D63.1	Anaemia in chronic kidney disease
E10.2	Type 1 diabetes mellitus with renal complications
E11.2	Type 2 diabetes mellitus with renal complications
E12.2	Malnutrition-related diabetes mellitus with renal complications
E13.2	Other specified diabetes mellitus with renal complications
E14.2	Unspecified diabetes mellitus with renal complications
I12	Hypertensive renal disease
I13	Hypertensive heart and renal disease
N02	Recurrent and persistent haematuria
N03	Chronic nephritic syndrome
N04	Nephrotic syndrome
N05	Unspecified nephritic syndrome
N06	Isolated proteinuria with specified morphological lesion
N07	Hereditary nephropathy, not elsewhere classified
N08	Glomerular disorders in diseases classified elsewhere
N15.0	Balkan nephropathy
N18	Chronic kidney disease
Q61	Cystic kidney disease
Q62	Congenital obstructive defects of renal pelvis and congenital malformations of ureter
Z94.0	Kidney transplant status

Clinical codes for degenerative disorders

GOLD: clinical, referral, tests; Aurum: consultations, observations.
Cerebral palsy is not included as a degenerative disorder – see separate codes in this section

Additional sources of information in addition to clinical opinion and in-house code repository: Davidson et al. 2022: https://doi.org/10.17037/DATA.00002817)

CPRD GOLD: Degenerative disorders			
medcode	readcode	readterm	interpretation
103120	Fyu2300	[X]Other specified degenerative diseases/the basal ganglia	spinal/bulbar muscular atrophy
115773	Fyu1200	[X]Other spinal muscular atrophies and related syndromes	spinal/bulbar muscular atrophy
101525	Fyu3200	[X]Subacute combined degeneration/spinal cord in diseases CE	spinal/bulbar muscular atrophy
114983	Fyu1.00	[X]Systemic atrophies primarily affecting the CNS	other degenerative
109873	Fyu1600	[X]Systemic atrophy affecting the CNS in other diseases CE	other degenerative
66575	F151200	Adult spinal muscular atrophy	spinal/bulbar muscular atrophy
73990	F101.11	Amaurotic familial idiocy	other degenerative
36433	F152000	Amyotrophic lateral sclerosis	other degenerative
21889	F15..00	Anterior horn cell disease	other degenerative
58729	F15z.00	Anterior horn cell disease NOS	other degenerative
62945	F211.11	Balo's concentric sclerosis	multiple sclerosis
25268	F101211	Batten's disease of retina	other degenerative
32749	F391800	Becker muscular dystrophy	muscular dystrophy
73283	G552.11	Becker's disease	other degenerative
44272	F391B00	Cardiomyopathy in Duchenne muscular dystrophy	muscular dystrophy
5584	F361000	Charcot-Marie-Tooth disease	other degenerative
40968	F21y500	Concentric sclerosis	multiple sclerosis
64690	F390.00	Congenital hereditary muscular dystrophy	muscular dystrophy
22174	F390z00	Congenital hereditary muscular dystrophy NOS	muscular dystrophy
73584	F14y100	Corticostriatal-spinal degeneration	other degenerative
11542	F360000	Dejerine-Sottas disease	other degenerative
66726	F391500	Distal (Gower's) muscular dystrophy	muscular dystrophy
68009	F391y11	Distal dystrophy	muscular dystrophy
7470	F152111	Duchenne Aran muscular atrophy	muscular dystrophy
5393	F391000	Duchenne muscular dystrophy	muscular dystrophy
49737	F142200	Dyssynergia cerebellaris myoclonica	muscular dystrophy
16118	F392000	Dystrophia myotonica (Steinert's disease)	muscular dystrophy
34985	F391A00	Emery-Dreifuss muscular dystrophy	muscular dystrophy
48036	F391100	Erb's muscular dystrophy	muscular dystrophy
2298	F203.00	Exacerbation of multiple sclerosis	multiple sclerosis
42756	F4Gy100	Extraocular muscle myopathy	other degenerative
67687	F391411	Facioscapulohumeral atrophy	muscular dystrophy
36671	F391400	Facioscapulohumeral muscular dystrophy	muscular dystrophy
105180	F1y0.00	Fragile X associated tremor ataxia syndrome	other degenerative
4165	F140.00	Friedreich's ataxia	other degenerative
23730	F202.00	Generalised multiple sclerosis	multiple sclerosis
68118	F391.00	Hereditary progressive muscular dystrophy	muscular dystrophy
21425	F391z00	Hereditary progressive muscular dystrophy NOS	muscular dystrophy
3591	F134.00	Huntington's chorea	Huntington's disease
44512	F364.00	Idiopathic progressive polyneuropathy	other degenerative
66740	F380100	Juvenile or adult myasthenia gravis	other degenerative
101222	F151111	Juvenile spinal muscular atrophy	spinal/bulbar muscular atrophy
45903	F100000	Krabbe's disease	other degenerative
43394	F151100	Kugelberg - Welander disease	other degenerative
48300	F10y100	Leigh's disease	other degenerative
97454	F427K11	Lipofuscinosis NEC	other degenerative
73742	F427K00	Lipofuscinosis NOS	other degenerative
29114	A940.11	Locomotor ataxia	other degenerative
16903	F14y011	Louis - Bar syndrome	other degenerative
59855	F100300	Metachromatic leucodystrophy	other degenerative
18307	F39X.00	Mitochondrial myopathy, not elsewhere classified	other degenerative
4796	F152.00	Motor neurone disease	motor neurone disease
20120	F152z00	Motor neurone disease NOS	motor neurone disease
69613	C377100	Mucolipidosis type III	other degenerative
684	F20..00	Multiple sclerosis	multiple sclerosis
20493	F20z.00	Multiple sclerosis NOS	multiple sclerosis
40344	F200.00	Multiple sclerosis of the brain stem	multiple sclerosis
69886	F201.00	Multiple sclerosis of the spinal cord	multiple sclerosis
22454	F174.00	Multiple system atrophy	multiple system atrophy
6599	F39..00	Muscular dystrophies and other myopathies	muscular dystrophy
5964	F39B.00	Muscular dystrophy	muscular dystrophy
27515	F380z00	Myasthenia gravis NOS	other degenerative
95005	F381200	Myasthenic syndrome due to botulism	other degenerative
51640	F381.00	Myasthenic syndrome due to disease EC	other degenerative
65825	F381z00	Myasthenic syndrome due to disease NOS	other degenerative
61069	F381400	Myasthenic syndrome due to hypothyroidism	other degenerative

57551	F381100	Myasthenic syndrome due to other malignancy	other degenerative
56973	F381500	Myasthenic syndrome due to pernicious anaemia	other degenerative
47695	F381600	Myasthenic syndrome due to thyrotoxicosis	other degenerative
11346	F38z.00	Myoneural disorder NOS	other degenerative
41904	F391600	Ocular muscular dystrophy	muscular dystrophy
38448	F391700	Oculopharyngeal muscular dystrophy	muscular dystrophy
71400	F15y.00	Other anterior horn cell disease	other degenerative
50762	F130z00	Other basal ganglia degenerative disease NOS	other degenerative
21863	F130.00	Other basal ganglia degenerative diseases	other degenerative
28210	F391300	Other limb-girdle muscular dystrophy	muscular dystrophy
32016	F39y.00	Other myopathies and muscular dystrophies	muscular dystrophy
43583	F21y.00	Other specified central nervous system demyelinating disease	other degenerative
54300	F21yz00	Other specified central nervous system demyelination NOS	other degenerative
71128	F391y00	Other specified hereditary progressive muscular dystrophy	muscular dystrophy
1691	F120.00	Paralysis agitans	other degenerative
4321	F12..00	Parkinson's disease	parkinson's disease
14912	F12z.00	Parkinson's disease NOS	parkinson's disease
102921	F10y200	PEHO syndrome	other degenerative
53382	F100200	Pelizaeus-Merzbacher disease	other degenerative
91544	F391200	Pelvic muscular dystrophy	muscular dystrophy
28901	F361.00	Peroneal muscular atrophy	other degenerative
70040	F361z00	Peroneal muscular atrophy NOS	other degenerative
58758	F374800	Polyneuropathy in porphyria	other degenerative
5128	F142.00	Primary cerebellar degeneration	other degenerative
58772	F142z00	Primary cerebellar degeneration NOS	other degenerative
20845	F152400	Primary lateral sclerosis	motor neurone disease
96607	F206.00	Primary progressive multiple sclerosis	multiple sclerosis
27377	F152200	Progressive bulbar palsy	spinal/bulbar muscular atrophy
106200	F4J7100	Progressive external ophthalmoplegia	other degenerative
30987	F152100	Progressive muscular atrophy	muscular dystrophy
40553	F130400	Progressive supranuclear ophthalmoplegia	other degenerative
9385	F24y000	Progressive supranuclear palsy	progressive supranuclear palsy
26210	F391011	Pseudohypertrophic dystrophy	muscular dystrophy
95972	F207.00	Relapsing and remitting multiple sclerosis	multiple sclerosis
109176	F143.11	Roussy-Levy syndrome	other degenerative
93963	F101600	Sandhoff disease	other degenerative
96246	F208.00	Secondary progressive multiple sclerosis	multiple sclerosis
35839	F130500	Shy-Drager syndrome	multiple system atrophy
56951	F101200	Spielmeyer-Vogt (Batten) disease	other degenerative
9179	F151.00	Spinal muscular atrophy	spinal/bulbar muscular atrophy
57632	F151z00	Spinal muscular atrophy NOS	spinal/bulbar muscular atrophy
21216	F14..00	Spinocerebellar disease	other degenerative
27331	F14z.00	Spinocerebellar disease NOS	other degenerative
93910	F24y012	Steele - Richardson Oszewski syndrome	progressive supranuclear palsy
49034	F24y011	Steele Richardson Olszewsk syn	progressive supranuclear palsy
7037	F24y200	Steele-Richardson-Olszewski syndrome	progressive supranuclear palsy
68541	F392011	Steinert's disease	muscular dystrophy
104967	F13z111	Stiff person syndrome	other degenerative
25880	F13z100	Stiff-man syndrome	other degenerative
49203	F130200	Striatonigral degeneration	other degenerative
7736	F162.00	Subacute combined degeneration of spinal cord	other degenerative
69848	F21y400	Subacute necrotizing myelitis	other degenerative
52677	A412.00	Subacute sclerosing panencephalitis	other degenerative
69837	A940.12	Syphilitic posterior spinal sclerosis	other degenerative
42397	A940.00	Tabes dorsalis - neurosyphilis	other degenerative
58576	F038.00	Tropical spastic paraplegia	other degenerative
70572	F151000	Unspecified spinal muscular atrophy	spinal/bulbar muscular atrophy
102338	F21y600	Vanishing white matter disease	other degenerative
33334	F150.00	Werdnig - Hoffmann disease	motor neurone disease
70109	F151300	X-linked bulbo-spinal atrophy	spinal/bulbar muscular atrophy

CPRD Aurum: Degenerative disorders			
**medcode**	**term**	**snomedconceptID**	**intepretation**
299304013	[x]other inherited spinal muscular atrophy	5262007	spinal/bulbar muscular atrophy
414541000006119	[x]other specified degenerative diseases/the basal ganglia	943181000000103	spinal/bulbar muscular atrophy
299305014	[x]other spinal muscular atrophies and related syndromes	5262007	spinal/bulbar muscular atrophy
428171000006118	[x]subacute combined degeneration/spinal cord in diseases ce	60576007	spinal/bulbar muscular atrophy
428581000006114	[x]systemic atrophies primarily affecting the cns	230226000	other degenerative

428601000006116	[x]systemic atrophy affecting the cns in other diseases ce	230226000	other degenerative
3889821000006110	adult onset spinal muscular atrophy	85505000	spinal/bulbar muscular atrophy
3889791000006110	adult spinal muscular atrophy	85505000	spinal/bulbar muscular atrophy
2824391000006110	alpers' disease	20415001	other degenerative
3898661000006110	als - amyotrophic lateral sclerosis	86044005	other degenerative
187765019	amaurotic familial idiocy	61663001	other degenerative
3501751000006110	amaurotic idiocy juvenile type	61663001	other degenerative
3501701000006110	amaurotic idiocy, juvenile type	61663001	other degenerative
142653015	amyotrophic lateral sclerosis	86044005	other degenerative
3931641000006110	amyotrophy	88092000	other degenerative
142016013	anterior horn cell disease	85672005	other degenerative
297132016	anterior horn cell disease nos	85672005	other degenerative
5006171000006110	autosomal dominant late onset basal ganglia degeneration	230313001	other degenerative
4931000006111	Autosomal recessive limb girdle muscular dystrophy type 2K	720523006	muscular dystrophy
5007011000006110	balo concentric sclerosis	230380005	multiple sclerosis
5007041000006110	balos concentric sclerosis	230380005	multiple sclerosis
2475876012	balo's concentric sclerosis	230380005	multiple sclerosis
5007031000006110	balo's concentric sclerosis	230380005	multiple sclerosis
3501681000006110	batten-mayou disease	61663001	other degenerative
3501741000006110	batten-mayou syndrome	61663001	other degenerative
504081000006111	batten's disease of retina	61663001	other degenerative
3501721000006110	batten-spielmeyer-vogt disease	61663001	other degenerative
1481810012	Becker muscular dystrophy	387732009	muscular dystrophy
1492251019	becker's disease	387732009	other degenerative
76871000006114	Becker's muscular dystrophy	387732009	muscular dystrophy
6476871000006110	becker's muscular dystrophy	387732009	muscular dystrophy
1772621000006110	bethlem myopathy	718572004	muscular dystrophy
76861000006119	BMD - Becker muscular dystrophy	387732009	muscular dystrophy
6476861000006110	bmd - becker muscular dystrophy	387732009	muscular dystrophy
4769291000006110	brain stem multiple sclerosis	192926004	multiple sclerosis
3898641000006110	bulbar motor neuron disease	86044005	motor neurone disease
3381201000006110	bulbar palsy	54304004	spinal/bulbar muscular atrophy
3381191000006110	bulbar paralysis	54304004	spinal/bulbar muscular atrophy
5005371000006110	bulbospinal muscular atrophy	230253001	spinal/bulbar muscular atrophy
5005341000006110	bulbospinal neuronopathy	230253001	spinal/bulbar muscular atrophy
460126017	Cardiomyopathy in Duchenne muscular dystrophy	315608004	muscular dystrophy
6596811000006110	charcot marie tooth disease, type 1	398040009	other degenerative
6599331000006110	charcot marie tooth disease, type 2	398187000	other degenerative
84201015	charcot-marie-tooth disease	398100001	other degenerative
6596791000006110	charcot-marie-tooth disease of demyelinating type	398040009	other degenerative
6596741000006110	charcot-marie-tooth disease, type i	398040009	other degenerative
6599291000006110	charcot-marie-tooth disease, type ii	398187000	other degenerative
3247251000006110	chronic progressive ophthalmoplegia	46252003	other degenerative
345223012	concentric sclerosis	230380005	multiple sclerosis
178743010	Congenital hereditary muscular dystrophy	111501005	muscular dystrophy
297607019	Congenital hereditary muscular dystrophy NOS	73297009	muscular dystrophy
42971000006119	Congenital muscular dystrophy	240059009	muscular dystrophy
2541000006116	Congenital muscular dystrophy with hyperlaxity	763314009	muscular dystrophy
27911000006120	Congenital muscular dystrophy with intellectual disability	783174004	muscular dystrophy
22561000006112	Congenital muscular dystrophy with intellectual disability and severe epilepsy	782772000	muscular dystrophy
5944191000006110	continuous muscle fiber activity	305719002	other degenerative
5944201000006110	continuous muscle fibre activity	305719002	other degenerative
3208041000006110	corticostriatal spinal degeneration	43977004	other degenerative
73325012	corticostriatal-spinal degeneration	43977004	other degenerative
3247271000006110	cpeo - chronic progressive external ophthalmoplegia	46252003	other degenerative
296840017	dawson's inclusion body encephalitis	192685000	other degenerative
4767951000006110	dawson's inclusion body encephalitis	192685000	other degenerative
8342831000006110	degenerative disease of basal ganglia	943181000000103	other degenerative
4196391000006110	dejerine-sottas disease	111499002	other degenerative
3694981000006110	dentato-rubral atrophy syndrome	73495003	other degenerative
3845831000006110	diffuse progressive ossifying polymyositis	82725007	other degenerative
25401000006110	Distal (Gower's) muscular dystrophy	193230001	muscular dystrophy
625401000006110	distal (gower's) muscular dystrophy	193230001	muscular dystrophy
625421000006117	distal dystrophy	58795000	muscular dystrophy
3455151000006110	distal muscular dystrophy	58795000	muscular dystrophy
70841000006119	Distal muscular dystrophy with juvenile onset	193230001	muscular dystrophy

4770841000006110	distal muscular dystrophy with juvenile onset	193230001	muscular dystrophy
3768241000006110	dm - dystrophia myotonica	77956009	muscular dystrophy
46961000006114	DMD - Duchenne muscular dystrophy	76670001	muscular dystrophy
3746961000006110	dmd - duchenne muscular dystrophy	76670001	muscular dystrophy
2894421000006110	ds - disseminated sclerosis	24700007	multiple sclerosis
631811000006111	duchenne aran muscular atrophy	88923002	muscular dystrophy
127307018	Duchenne muscular dystrophy	76670001	muscular dystrophy
3945241000006110	duchenne-aran muscular atrophy	88923002	muscular dystrophy
6060641000006110	duchenne's disease	316841006	muscular dystrophy
297107018	dyssynergia cerebellaris myoclonica	73495003	muscular dystrophy
60101000006116	Dystrophia myotonica	1177122009	muscular dystrophy
3768221000006110	dystrophia myotonica	77956009	muscular dystrophy
633431000006115	dystrophia myotonica (steinert's disease)	77956009	muscular dystrophy
54141000006118	Dystrophia myotonica facies	248201006	muscular dystrophy
4196651000006110	emd - emery-dreifuss muscular dystrophy	111508004	muscular dystrophy
178750014	emery-dreifuss muscular dystrophy	111508004	muscular dystrophy
3776451000006110	erb muscular dystrophy	78468005	muscular dystrophy
130209011	Erb's muscular dystrophy	78468005	muscular dystrophy
297180018	exacerbation of multiple sclerosis	192929006	multiple sclerosis
660171000006114	extraocular muscle myopathy	46252003	other degenerative
2665381000006110	fa - friedreich ataxia	10394003	other degenerative
661251000006116	facioscapulohumeral atrophy	399091004	muscular dystrophy
1778554019	facioscapulohumeral muscular dystrophy	399091004	muscular dystrophy
6568101000006110	familial progressive cerebral sclerosis	396338004	other degenerative
3380791000006110	familial spinal muscular atrophy	54280009	spinal/bulbar muscular atrophy
17211000006113	Fascioscapulohumeral muscular dystrophy	399091004	muscular dystrophy
6617211000006110	fascioscapulohumeral muscular dystrophy	399091004	muscular dystrophy
3845841000006110	fibrodysplasia ossificans progressiva	82725007	other degenerative
6617201000006110	fmd - facioscapulohumeral muscular dystrophy	399091004	muscular dystrophy
3845851000006110	fop - fibrodysplasia ossificans progressiva	82725007	other degenerative
2900005013	fragile x associated tremor ataxia syndrome	448045004	other degenerative
1781041000006110	fragile x associated tremor ataxia syndrome	759431000000104	other degenerative
18091019	friedreich's ataxia	10394003	other degenerative
2665351000006110	friedreich's ataxia	10394003	other degenerative
2665361000006110	friedreich's disease	10394003	other degenerative
17161000006116	FSH - Facioscapulohumeral muscular dystrophy	399091004	muscular dystrophy
6617161000006110	fsh - facioscapulohumeral muscular dystrophy	399091004	muscular dystrophy
17191000006112	FSHD - Facioscapulohumeral muscular dystrophy	399091004	muscular dystrophy
6617191000006110	fshd - facioscapulohumeral muscular dystrophy	399091004	muscular dystrophy
7349161000006110	fxtas - fragile x associated tremor ataxia syndrome	448045004	other degenerative
12759421000006100	fxtas - fragile x associated tremor ataxia syndrome	759431000000104	other degenerative
4768711000006110	gcl - globoid cell leucodystrophy	192782005	other degenerative
297179016	generalised multiple sclerosis	192928003	multiple sclerosis
3889811000006110	generalised spinal muscular atrophy of late onset	85505000	spinal/bulbar muscular atrophy
4769321000006110	generalized multiple sclerosis	192928003	multiple sclerosis
3889801000006110	generalized spinal muscular atrophy of late onset	85505000	spinal/bulbar muscular atrophy
4768691000006110	globoid cell leucodystrophy	192782005	other degenerative
2824401000006110	gray matter degeneration	20415001	other degenerative
359673017	hauptmann-thannhauser muscular dystrophy	240072005	muscular dystrophy
297608012	Hereditary progressive muscular dystrophy	193225000	muscular dystrophy
297623019	hereditary progressive muscular dystrophy nos	73297009	muscular dystrophy
6597771000006110	hereditary sensorimotor neuropathy	398100001	other degenerative
6597711000006110	hereditary sensory and motor neuropathy	398100001	other degenerative
6597721000006110	hereditary sensory-motor neuropathy	398100001	other degenerative
6596751000006110	hereditary sensory-motor neuropathy, type i	398040009	other degenerative
4196401000006110	hereditary sensory-motor neuropathy, type iii	111499002	other degenerative
6597741000006110	hmsn	398100001	other degenerative
6597751000006110	hmsn - hereditary motor and sensory neuropathy	398100001	other degenerative
4196461000006110	hmsn iii	111499002	other degenerative
6597731000006110	hsmn	398100001	other degenerative
6597761000006110	hsmn - hereditary sensory and motor neuropathy	398100001	other degenerative
4196451000006110	hsmn iii	111499002	other degenerative
6596801000006110	hsmn, type i	398040009	other degenerative
7717301000006110	htlv-i-associated myelopathy	714279000	other degenerative
7717281000006110	human t-cell lymphotropic virus 1-associated myelopathy	714279000	other degenerative
3454551000006110	Huntington chorea	58756001	Huntington's disease
14195861000006100	Huntington disease	58756001	Huntington's disease
97642017	Huntington's chorea	58756001	Huntington's disease

3694991000006110	hunt's disease	73495003	other degenerative
4196491000006110	hypertrophic demyelinative neuropathy of infancy	111499002	other degenerative
4196471000006110	hypertrophic hereditary neuropathy	111499002	other degenerative
3846041000006110	hypokalaemic periodic paralysis	82732003	other degenerative
3846031000006110	hypokalemic periodic paralysis	82732003	other degenerative
3927641000006110	hypothyroid myopathy	87844004	other degenerative
3293681000006110	idiopathic parkinsonism	49049000	parkinson's disease
3293631000006110	idiopathic parkinson's disease	49049000	parkinson's disease
55426011	idiopathic progressive polyneuropathy	33209009	other degenerative
2975571000006110	infantile necrotising encephalomyelopathy	29570005	other degenerative
2975521000006110	infantile necrotizing encephalomyelopathy	29570005	other degenerative
6596771000006110	inherited dominant hypertrophic neuropathy	398040009	other degenerative
6599301000006110	inherited neuronal peroneal muscular atrophy	398187000	other degenerative
3142511000006110	juvenile myopathy, encephalopathy, lactic acidosis and stroke	39925003	other degenerative
5006011000006110	Juvenile onset Huntington's disease	230299004	Huntington's disease
297581011	juvenile or adult myasthenia gravis	193207007	other degenerative
90229010	juvenile spinal muscular atrophy	54280009	spinal/bulbar muscular atrophy
345068012	kennedy syndrome	230253001	spinal/bulbar muscular atrophy
4759791000006110	kerasin histiocytosis	190794006	other degenerative
4759811000006110	kerasin lipoidosis	190794006	other degenerative
3601731000006110	kiloh-nevin syndrome	67747009	other degenerative
4768731000006110	krabbe disease	192782005	other degenerative
4768701000006110	krabbe leucodystrophy	192782005	other degenerative
296966018	krabbe's disease	192782005	other degenerative
4768681000006110	krabbe's leukodystrophy	192782005	other degenerative
753451000006118	kugelberg - welander disease	54280009	other degenerative
3380781000006110	kugelberg-welander disease	54280009	other degenerative
6607251000006110	lame sickness	398565003	other degenerative
6617171000006110	landouzy-dejerine muscular dystrophy	399091004	muscular dystrophy
6617181000006110	landouzy-dejerine muscular dystrophy	399091004	muscular dystrophy
5006031000006110	Late onset Huntington's disease	230300007	Huntington's disease
2975611000006110	leigh disease	29570005	other degenerative
2975601000006110	leigh syndrome	29570005	other degenerative
2975621000006110	leighs disease	29570005	other degenerative
49474011	leigh's disease	29570005	other degenerative
4768661000006110	leukodystrophy	192781003	other degenerative
4015811000006110	leyden-mobius muscular dystrophy	93153005	muscular dystrophy
3484021000006110	lichtheim's disease	60576007	other degenerative
15801000006118	Limb-girdle muscular dystrophy	93153005	muscular dystrophy
4015801000006110	limb-girdle muscular dystrophy	93153005	muscular dystrophy
356811012	lipofuscinosis nec	14210003	other degenerative
399436016	lipofuscinosis nos	14210003	other degenerative
736381000006110	locomotor ataxia	316841006	other degenerative
3898651000006110	lou gehrig's disease	86044005	other degenerative
734711000006110	louis - bar syndrome	68504005	other degenerative
3614671000006110	louis-bar syndrome	68504005	other degenerative
641301000000114	malignant multiple sclerosis	439567002	multiple sclerosis
6461171000006110	marchiafava disease	386766007	multiple sclerosis
3256761000006110	marie cerebellar ataxia	46808003	other degenerative
3691951000006110	md - muscular dystrophy	73297009	muscular dystrophy
3142531000006110	melas - mitochondrial myopathy, encephalopathy, lactic acidosis and stroke-like episodes	39925003	other degenerative
1785557019	metachromatic leucodystrophy	396338004	other degenerative
6568031000006110	metachromatic leukodystrophy	396338004	other degenerative
6568081000006110	metachromatic leukoencephaly	396338004	other degenerative
3988991000006110	mg - myasthenia gravis	91637004	other degenerative
3142551000006110	mitochondrial myopathy, encephalopathy, lactic acidosis and stroke-like episodes	39925003	other degenerative
701491000006117	mitochondrial myopathy, not elsewhere classified	16851005	other degenerative
2912671000006110	mitochondrial ocular myopathy	25792000	other degenerative
6568061000006110	mld	396338004	other degenerative
6568111000006110	mld - metachromatic leucodystrophy	396338004	other degenerative
3101721000006110	mnd - motor neurone disease	37340000	motor neurone disease
2582971000006110	moersch-woltman syndrome	5217008	other degenerative
2582951000006110	moersch-woltmann syndrome	5217008	other degenerative
3101701000006110	motor neuron disease	37340000	motor neurone disease
486695013	motor neurone disease	37340000	motor neurone disease
297130012	motor neurone disease nos	37340000	motor neurone disease
2894411000006110	ms - multiple sclerosis	24700007	multiple sclerosis

5005991000006110	msa - multiple system atrophy	230297002	multiple system atrophy
3569341000006110	mucolipidosis iii	65764006	other degenerative
696641000006114	mucolipidosis type iii	65764006	other degenerative
41398015	multiple sclerosis	24700007	multiple sclerosis
4769341000006110	multiple sclerosis (ms) exacerbation	192929006	multiple sclerosis
7092351000006110	multiple sclerosis (ms) primary progressive	428700003	multiple sclerosis
7058051000006110	multiple sclerosis (ms) relapsing remitting	426373005	multiple sclerosis
7045281000006110	multiple sclerosis (ms) secondary progressive	425500002	multiple sclerosis
297181019	multiple sclerosis nos	24700007	multiple sclerosis
695191000006119	multiple sclerosis of the brain stem	192926004	multiple sclerosis
4769281000006110	multiple sclerosis of the brainstem	192926004	multiple sclerosis
297177019	multiple sclerosis of the spinal cord	192927008	multiple sclerosis
345122012	multiple system atrophy	230297002	multiple system atrophy
2838078010	multiple system atrophy, cerebellar variant	444024002	multiple system atrophy
2841061014	multiple system atrophy, parkinson variant	444197004	multiple system atrophy
7289421000006110	multiple system atrophy, parkinson's variant	444197004	multiple system atrophy
3845821000006110	munchmeyer disease	82725007	other degenerative
297602013	muscular dystrophies and other myopathies	75047002	muscular dystrophy
121721014	Muscular dystrophy	73297009	muscular dystrophy
42821000006112	Muscular dystrophy with predominantly proximal limb girdle distribution	240046001	muscular dystrophy
3532651000006110	myasthenia angiosclerotica	63491006	other degenerative
297582016	myasthenia gravis nos	91637004	other degenerative
683431000006111	myasthenic syndrome due to botulism	398565003	other degenerative
297583014	myasthenic syndrome due to disease ec	193209005	other degenerative
297592012	myasthenic syndrome due to disease nos	193209005	other degenerative
297588017	myasthenic syndrome due to hypothyroidism	193212008	other degenerative
297584015	myasthenic syndrome due to other malignancy	193209005	other degenerative
297589013	myasthenic syndrome due to pernicious anaemia	193213003	other degenerative
297591017	myasthenic syndrome due to thyrotoxicosis	193214009	other degenerative
297601018	myoneural disorder nos	128213006	other degenerative
5501291000006110	myoplegic dystrophy	267607008	other degenerative
3845871000006110	myositis ossificans progressiva	82725007	other degenerative
3768251000006110	myotonia dystrophica	77956009	other degenerative
7730241000006110	myotonic dystrophy type 2	715317001	other degenerative
4769101000006110	myxedema cerebellar degeneration	192876003	other degenerative
4769091000006110	myxoedema cerebellar degeneration	192876003	other degenerative
3005741000006110	neuromyositis	31384009	other degenerative
112508017	Ocular muscular dystrophy	67747009	muscular dystrophy
3753631000006110	oculopharyngeal dystrophy	77097004	muscular dystrophy
127990018	Oculopharyngeal muscular dystrophy	77097004	muscular dystrophy
3601951000006110	olivopontocerebellar degeneration	67761004	other degenerative
2968561000006110	osteochondromuscular dystrophy	29145002	muscular dystrophy
297131011	other anterior horn cell disease	85672005	other degenerative
297045019	other basal ganglia degenerative disease nos	230226000	other degenerative
297041011	other basal ganglia degenerative diseases	230226000	other degenerative
27731000006112	other limb-girdle muscular dystrophy	93153005	muscular dystrophy
883181000006113	other myoneural disorders	257277002	other degenerative
297654015	other myopathies and muscular dystrophies	75047002	muscular dystrophy
297190014	other specified central nervous system demyelinating disease	6118003	other degenerative
297195016	other specified central nervous system demyelination nos	6118003	other degenerative
399421019	Other specified hereditary progressive muscular dystrophy	73297009	muscular dystrophy
3293611000006110	paralysis agitans	49049000	other degenerative
4769111000006110	paraneoplastic cerebellar degeneration	192877007	other degenerative
3763441000006110	paraneoplastic polyneuropathy	77659000	other degenerative
3293641000006110	parkinson disease	49049000	parkinson's disease
3293661000006110	parkinsons disease	49049000	parkinson's disease
81717011	parkinson's disease	49049000	parkinson's disease
297037012	parkinson's disease nos	49049000	parkinson's disease
3381211000006110	pbp - progressive bulbar palsy	54304004	spinal/bulbar muscular atrophy
3293651000006110	pd - parkinson's disease	49049000	parkinson's disease
1705661000006110	peho syndrome	380941000000104	other degenerative
107800019	pelizaeus-merzbacher disease	64855000	other degenerative
297610014	pelvic muscular dystrophy	193227008	muscular dystrophy
3247281000006110	peo - progressive external ophthalmoplegia	46252003	other degenerative
5012271000006110	periventricular leukoencephalopathy	230769007	other degenerative
84200019	peroneal muscular atrophy	398100001	other degenerative

297522019	peroneal muscular atrophy nos	398100001	other degenerative
6596761000006110	peroneal muscular atrophy of demyelinating type	398040009	other degenerative
6599321000006110	peroneal muscular atrophy of neuronal type	398187000	other degenerative
2546871000006110	pigmentary pallidal atrophy	2992000	other degenerative
2546861000006110	pigmentary pallidal degeneration	2992000	other degenerative
3945261000006110	pma - progressive muscular atrophy	88923002	other degenerative
3691971000006110	pmd - progressive muscular dystrophy	73297009	other degenerative
297564019	polyneuropathy in porphyria	129616004	other degenerative
4428511000006110	porphyric polyneuropathy	129616004	other degenerative
3483991000006110	posterolateral sclerosis	60576007	other degenerative
3001481000006110	post-polio progressive muscular atrophy	31097004	other degenerative
39846012	primary cerebellar degeneration	23732000	other degenerative
297108011	primary cerebellar degeneration nos	23732000	other degenerative
134744017	primary lateral sclerosis	81211007	motor neurone disease
3293671000006110	primary parkinsonism	49049000	parkinson's disease
2692565012	primary progressive multiple sclerosis	428700003	multiple sclerosis
90263012	progressive bulbar palsy	54304004	spinal/bulbar muscular atrophy
77128019	progressive external ophthalmoplegia	46252003	other degenerative
3247231000006110	progressive external ophthalmoplegia	46252003	other degenerative
4196431000006110	progressive hypertrophic interstitial neuropathy	111499002	other degenerative
147444011	progressive muscular atrophy	88923002	muscular dystrophy
3001491000006110	progressive muscular atrophy following poliomyelitis	31097004	other degenerative
3547301000006110	progressive muscular atrophy of infancy	64383006	muscular dystrophy
3691961000006110	progressive muscular dystrophy	73297009	muscular dystrophy
2824431000006110	progressive neuronal degeneration with liver cirrhosis	20415001	other degenerative
2824381000006110	progressive sclerosing poliodystrophy	20415001	other degenerative
48512013	progressive supranuclear ophthalmoplegia	28978003	other degenerative
2966061000006110	progressive supranuclear ophthalmoplegia	28978003	other degenerative
11904601000006100	progressive supranuclear ophthalmoplegia	28978003	other degenerative
48513015	progressive supranuclear palsy	28978003	progressive supranuclear palsy
11904611000006100	progressive supranuclear palsy	28978003	progressive supranuclear palsy
2637771000000110	proximal myotonic myopathy	715317001	other degenerative
3569351000006110	pseudo-hurler disease	65764006	other degenerative
3569361000006110	pseudo-hurler's disease	65764006	other degenerative
98381000006110	Pseudohypertrophic dystrophy	76670001	muscular dystrophy
198381000006110	pseudohypertrophic dystrophy	76670001	muscular dystrophy
3746951000006110	pseudohypertrophic muscular dystrophy	76670001	muscular dystrophy
2966101000006110	psp - progressive supranuclear palsy	28978003	progressive supranuclear palsy
3945251000006110	pure progressive muscular atrophy	88923002	muscular dystrophy
3484011000006110	putnam-dana syndrome	60576007	other degenerative
2767491000006110	ragged red myopathy	16851005	other degenerative
1682241000006110	relapsing and remitting multiple sclerosis	426373005	multiple sclerosis
3240521000006110	roussy-levy syndrome	45853006	other degenerative
3484051000006110	sacd - subacute combined degeneration	60576007	other degenerative
40055016	sandhoff disease	23849003	other degenerative
5005931000006110	secondary parkinson disease	230292008	parkinson's disease
5005921000006110	secondary parkinson's disease	230292008	parkinson's disease
2674605012	secondary progressive multiple sclerosis	425500002	multiple sclerosis
28047015	shy-drager syndrome	16576004	multiple system atrophy
2583601000006110	sma - spinal muscular atrophy	5262007	spinal/bulbar muscular atrophy
3380831000006110	sma type iii	54280009	spinal/bulbar muscular atrophy
3380821000006110	sma3	54280009	spinal/bulbar muscular atrophy
2900061000006110	smon - subacute myelo-optico-neuropathy	25044007	other degenerative
2976321000006110	snd - striatonigral degeneration	29618004	other degenerative
2975581000006110	snem - subacute necrotising encephalomyelopathy	29570005	other degenerative
2975591000006110	snem - subacute necrotizing encephalomyelopathy	29570005	other degenerative
3501691000006110	spielmeyer-vogt disease	61663001	other degenerative
3501761000006110	spielmeyer-vogt type neuronal ceroid lipofuscinosis	61663001	other degenerative
9822014	spinal muscular atrophy	5262007	spinal/bulbar muscular atrophy
297127017	spinal muscular atrophy nos	5262007	spinal/bulbar muscular atrophy
3547321000006110	spinal muscular atrophy type i	64383006	spinal/bulbar muscular atrophy
3380841000006110	spinal muscular atrophy type iii	54280009	spinal/bulbar muscular atrophy
3547311000006110	spinal muscular atrophy, type i	64383006	spinal/bulbar muscular atrophy
3380811000006110	spinal muscular atrophy, type iii	54280009	spinal/bulbar muscular atrophy
151586018	spinocerebellar disease	91502009	other degenerative
297123018	spinocerebellar disease nos	91502009	other degenerative
2824411000006110	spongy glioneuronal dystrophy	20415001	other degenerative
4767941000006110	sspe - subacute sclerosing panencephalitis	192685000	progressive supranuclear palsy
125891000006119	steele - richardson oszewski syndrome	28978003	progressive supranuclear palsy

125901000006115	steele richardson olszewsk syn	28978003	progressive supranuclear palsy
48515010	steele-richardson-olszewski syndrome	28978003	progressive supranuclear palsy
11903671000006100	steele-richardson-olszewski syndrome	28978003	progressive supranuclear palsy
68201000006114	Steinert myotonic dystrophy syndrome	77956009	muscular dystrophy
3768201000006110	steinert myotonic dystrophy syndrome	77956009	muscular dystrophy
3768211000006110	steinert syndrome	77956009	muscular dystrophy
25971000006114	Steinert's disease	77956009	muscular dystrophy
2108221000000110	stiff person syndrome	5217008	other degenerative
9742014	stiff-man syndrome	5217008	other degenerative
2582941000006110	stiff-man syndrome	5217008	other degenerative
2976311000006110	striatonigral atrophy	29618004	other degenerative
49542018	striatonigral degeneration	29618004	other degenerative
498788015	subacute combined degeneration	60576007	other degenerative
100638016	subacute combined degeneration of spinal cord	60576007	other degenerative
2900031000006110	subacute myelo-optic neuropathy	25044007	other degenerative
2975551000006110	subacute necrotising encephalomyelopathy	29570005	other degenerative
2975561000006110	subacute necrotising encephalopathy	29570005	other degenerative
2975541000006110	subacute necrotizing encephalomyelopathy	29570005	other degenerative
2975531000006110	subacute necrotizing encephalopathy	29570005	other degenerative
5007001000006110	subacute necrotizing myelitis	230379007	other degenerative
296838010	subacute sclerosing panencephalitis	192685000	other degenerative
6568051000006110	sulfatide lipidosis	396338004	other degenerative
6568041000006110	sulphatide lipidosis	396338004	other degenerative
496159016	syphilitic posterior spinal sclerosis	316841006	other degenerative
6060631000006110	tabes dorsalis	316841006	other degenerative
496154014	tabes dorsalis - neurosyphilis	316841006	other degenerative
7717311000006110	tropical spastic paraparesis	714279000	other degenerative
494103012	tropical spastic paraplegia	714279000	other degenerative
297125013	unspecified spinal muscular atrophy	5262007	spinal/bulbar muscular atrophy
1648061000000110	vanishing white matter disease	447351004	other degenerative
1728581000006110	vanishing white matter disease	1728581000006104	other degenerative
59911000006112	werdnig - hoffmann disease	64383006	motor neurone disease
3547281000006110	werdnig-hoffmann disease	64383006	motor neurone disease
3547331000006110	whd - werdnig-hoffmann disease	64383006	motor neurone disease
5005361000006110	x-linked bulbospinal atrophy	230253001	spinal/bulbar muscular atrophy
55351000006111	x-linked bulbo-spinal atrophy	230253001	spinal/bulbar muscular atrophy

Hospital episode statistics: Degenerative disorders	
**icd**	**description**
G12	Spinal muscular atrophy and related syndromes
G20	Parkinson's disease
G23	Other degenerative diseases of basal ganglia
G31	Other degenerative diseases of nervous system, not elsewhere classified
G32	Other degenerative disorders of nervous system in diseases classified elsewhere
G35	Multiple sclerosis
G37.4	Subacute necrotizing myelitis
G37.5	Concentric sclerosis [Baló]
G70	Myasthenia gravis and other myoneural disorders

Clinical codes for dementia

GOLD: clinical, referral, tests; Aurum: consultations, observations.
Process measures (e.g. monitoring, management, referrals) are not included

CPRD GOLD: Dementia		
**medcode**	**readcode**	**readterm**
42602	E001000	Uncomplicated presenile dementia
48501	Eu02z11	[X] Presenile dementia NOS
7664	Eu00.00	[X]Dementia in Alzheimer's disease
31016	Eu01300	[X]Mixed cortical and subcortical vascular dementia
55467	E004200	Arteriosclerotic dementia with paranoia
61528	Eu00013	[X]Alzheimer's disease type 2
29386	Eu00z00	[X]Dementia in Alzheimer's disease, unspecified
64267	Eu02y00	[X]Dementia in other specified diseases classif elsewhere
55838	Eu01111	[X]Predominantly cortical dementia
8195	Eu00z11	[X]Alzheimer's dementia unspec
43089	E004000	Uncomplicated arteriosclerotic dementia
7323	E000.00	Uncomplicated senile dementia

19393	Eu01z00	[X]Vascular dementia, unspecified
59122	Fyu3000	[X]Other Alzheimer's disease
49263	Eu00000	[X]Dementia in Alzheimer's disease with early onset
60059	Eu00012	[X]Primary degen dementia, Alzheimer's type, presenile onset
6578	Eu01.00	[X]Vascular dementia
30032	E001200	Presenile dementia with paranoia
1916	E00..11	Senile dementia
15165	E001.00	Presenile dementia
51494	E00y.11	Presbyophrenic psychosis
2882	E00z.00	Senile or presenile psychoses NOS
38678	Eu00100	[X]Dementia in Alzheimer's disease with late onset
25386	E041.00	Dementia in conditions EC
30706	Eu00200	[X]Dementia in Alzheimer's dis, atypical or mixed type
1917	F110.00	Alzheimer's disease
26270	Eu02500	[X]Lewy body dementia
27677	E001300	Presenile dementia with depression
47619	Eu02z12	[X] Presenile psychosis NOS
4693	Eu02z00	[X] Unspecified dementia
18386	E002000	Senile dementia with paranoia
21887	E002100	Senile dementia with depression
8934	Eu01200	[X]Subcortical vascular dementia
11175	Eu01100	[X]Multi-infarct dementia
43292	E004300	Arteriosclerotic dementia with depression
34944	Eu02z13	[X] Primary degenerative dementia NOS
19477	E004.00	Arteriosclerotic dementia
53446	Eu04100	[X]Delirium superimposed on dementia
41089	E002z00	Senile dementia with depressive or paranoid features NOS
37014	Eu02200	[X]Dementia in Huntington's disease
16797	F110000	Alzheimer's disease with early onset
38286	A411.00	Jakob-Creutzfeldt disease
4357	Eu02z14	[X] Senile dementia NOS
1350	E00..12	Senile/presenile dementia
12621	Eu02.00	[X]Dementia in other diseases classified elsewhere
29512	F112.00	Senile degeneration of brain
55313	Eu01y00	[X]Other vascular dementia
46762	Eu00111	[X]Alzheimer's disease type 1
15249	E00y.00	Other senile and presenile organic psychoses
32057	F110100	Alzheimer's disease with late onset
46488	Eu01000	[X]Vascular dementia of acute onset
49513	E001100	Presenile dementia with delirium
11136	F111.00	Pick's disease
27935	Eu02z15	[X] Senile psychosis NOS
42279	E004z00	Arteriosclerotic dementia NOS
38438	E001z00	Presenile dementia NOS
48531	F11x700	Cerebral degeneration due to Jakob - Creutzfeldt disease
43346	Eu00113	[X]Primary degen dementia of Alzheimer's type, senile onset
8634	E004.11	Multi infarct dementia
37946	E012000	Chronic alcoholic brain syndrome
28402	Eu02000	[X]Dementia in Pick's disease
7572	F116.00	Lewy body disease
26323	Eu10711	[X]Alcoholic dementia NOS
9565	Eu01.11	[X]Arteriosclerotic dementia
62132	E02y100	Drug-induced dementia
25704	Eu00011	[X]Presenile dementia,Alzheimer's type
54106	Eu02100	[X]Dementia in Creutzfeldt-Jakob disease
33707	E00..00	Senile and presenile organic psychotic conditions
44674	E002.00	Senile dementia with depressive or paranoid features
27342	E012.11	Alcoholic dementia NOS
54505	E012.00	Other alcoholic dementia
9509	Eu02300	[X]Dementia in Parkinson's disease
11379	Eu00112	[X]Senile dementia,Alzheimer's type
56912	E004100	Arteriosclerotic dementia with delirium
37015	E003.00	Senile dementia with delirium
27759	Eu02z16	[X] Senile dementia, depressed or paranoid type
41185	Eu02400	[X]Dementia in human immunodef virus [HIV] disease
114713	F118100	Frontotemporal dementia

CPRD Aurum: Dementia		
**medcode**	**term**	**snomedconceptID**
2386018	Jakob-Creutzfeldt disease	792004
21256010	Presenile dementia	12348006
22408016	Pick's disease	13092008
26545010	Senile dementia	15662003
45046017	Alzheimer's disease	26929004
76484016	Senile degeneration of brain	45864009
294635013	Uncomplicated senile dementia	191449005
294637017	Uncomplicated presenile dementia	191451009
294638010	Presenile dementia with delirium	191452002
294641018	Presenile dementia with paranoia	191454001
294642013	Presenile dementia with depression	191455000
294643015	Presenile dementia NOS	12348006
294644014	Senile dementia with depressive or paranoid features	191457008
294645010	Senile dementia with paranoia	191458003
294646011	Senile dementia with depression	191459006
294647019	Senile dementia with depressive or paranoid features NOS	191457008
294648012	Senile dementia with delirium	191461002
294652012	Uncomplicated arteriosclerotic dementia	191463004
294653019	Arteriosclerotic dementia with delirium	191464005
294654013	Arteriosclerotic dementia with paranoia	191465006
294655014	Arteriosclerotic dementia with depression	191466007
294656010	Arteriosclerotic dementia NOS	56267009
294660013	Senile or presenile psychoses NOS	268612007
294668018	Chronic alcoholic brain syndrome	191475009
294688019	Drug-induced dementia	191493005
294718018	Dementia in conditions EC	191519005
295668011	[X]Dementia in Alzheimer's disease	26929004
295671015	[X]Dementia in Alzheimer's dis, atypical or mixed type	26929004
295672010	[X]Dementia in Alzheimer's disease, unspecified	26929004
295680015	[X]Other vascular dementia	429998004
295681016	[X]Vascular dementia, unspecified	429998004
295684012	Dementia associated with another disease	191519005
295685013	Dementia due to Pick's disease	21921000119103
295686014	Dementia due to Creutzfeldt Jakob disease	429458009
295687017	Dementia due to Huntington chorea	442344002
295688010	Dementia in Parkinsons disease	425390006
295690011	[X]Dementia in other specified diseases classif elsewhere	191519005
295714013	[X]Delirium superimposed on dementia	2776000
299325013	[X]Other Alzheimer's disease	26929004
346897015	Presbyophrenic psychosis	231438001
346929012	Alcoholic dementia	281004
401757012	Senile and presenile organic psychotic conditions	268612007
401759010	Other senile and presenile organic psychoses	268612007
401760017	Other alcoholic dementia	281004
497559016	Arteriosclerotic dementia	56267009
499946014	Alzheimer's disease with early onset	416780008
500317011	Alzheimer's disease with late onset	416975007
3635492010	Subcortical dementia	762707000
148381000006115	Senile/presenile dementia	52448006
359081000006118	[X] Presenile dementia NOS	12348006
359091000006115	[X] Presenile psychosis NOS	268612007
359101000006114	Cerebral degeneration presenting primarily with dementia	279982005
359141000006111	[X] Senile dementia NOS	15662003
359151000006113	[X] Senile dementia, depressed or paranoid type	191457008
359161000006110	[X] Senile psychosis NOS	268612007
359241000006119	Dementia	52448006
362941000006113	[X]Alcoholic dementia NOS	281004
363021000006113	[X]Alzheimer's dementia unspec	26929004
363031000006111	[X]Alzheimer's disease type 1	416975007
363041000006118	[X]Alzheimer's disease type 2	416780008
363791000006112	[X]Arteriosclerotic dementia	56267009
376531000006119	Dementia in Alzheimer's disease with early onset	416780008
376541000006112	Dementia in Alzheimer's disease with late onset	416975007

376571000006116	[X]Dementia in human immunodef virus [HIV] disease	421529006
398571000006112	Mixed cortical and subcortical vascular dementia	230287006
399031000006111	[X]Multi-infarct dementia	56267009
423221000006117	[X]Predominantly cortical dementia	56267009
423351000006115	[X]Presenile dementia,Alzheimer's type	416780008
423381000006111	[X]Primary degen dementia of Alzheimer's type, senile onset	416975007
423391000006114	[X]Primary degen dementia, Alzheimer's type, presenile onset	416780008
425901000006116	[X]Senile dementia,Alzheimer's type	416975007
428201000006119	Subcortical vascular dementia	230286002
431681000006117	Vascular dementia	429998004
431691000006119	Vascular dementia of acute onset	230285003
542651000006110	Cerebral degeneration due to Jakob - Creutzfeldt disease	192818008
696161000006115	Multi-infarct dementia	56267009
745381000006119	Lewy body disease	80098002
882171000006115	Dementia	268612007
882191000006119	Senile and presenile dementias	268612007
882201000006116	Senile dementia - simple type	191449005
882211000006118	Senile dementia-acute confused	191461002
914921000006117	[D] Vascular dementia	914921000006101
914931000006119	[D] Dementia with Lewy bodies	914931000006103
914941000006112	[D] Dementia	914941000006108
914951000006114	[D] Dementia in Alzheimer's disease	914951000006105
1823871000006112	Dementia confirmed	1823871000006108
1971401000006111	Dementia in Alzheimer's disease with early onset, without additional symptoms	1971401000006107
1971541000006114	Dementia in Alzheimer's disease with early onset, other symptoms, predominantly delusional	1971541000006105
1971661000006114	Mental & behav dis due to seds/hypntcs: resid & late-onset psychot dis, dementia	1971661000006105
1971701000006118	Mental & behav dis due to use opioids: resid & late-onset psychot dis, dementia	1971701000006102
1971771000006112	Dementia in Alzheimer's disease with early onset, other symptoms, predominantly hallucinatory	1971771000006108
1972021000006119	Unspecified dementia, without additional symptoms	1972021000006103
1972041000006114	Unspecified dementia, other symptoms, predominantly delusional	1972041000006105
1972061000006113	Unspecified dementia, other symptoms, predominantly hallucinatory	1972061000006109
1972071000006118	Unspecified dementia, other symptoms, predominantly depressive	1972071000006102
1972081000006115	Unspecified dementia, other mixed symptoms	1972081000006104
1972131000006115	Dementia in Alzheimer's disease with early onset, other symptoms, predominantly depressive	1972131000006104
1972141000006113	Dementia in Alzheimer's disease with early onset, other mixed symptoms	1972141000006109
1972171000006117	Dementia in Alzheimer's disease with late onset, without additional symptoms	1972171000006101
1972181000006119	Dementia in Alzheimer's disease with late onset, other symptoms, predominantly delusional	1972181000006103
1972191000006116	Dementia in Alzheimer's disease with late onset, other symptoms, predominantly hallucinatory	1972191000006100
1972201000006118	Dementia in Alzheimer's disease with late onset, other symptoms, predominantly depressive	1972201000006102
1972211000006115	Dementia in Alzheimer's disease with late onset, other mixed symptoms	1972211000006104
1972231000006114	Dementia in Alzheimer's dis, atypical or mixed type, without additional symptoms	1972231000006105
1972251000006119	Dementia in Alzheimer's dis, atypical or mixed type, other symptoms, predominantly delusional	1972251000006103
1972291000006113	Dementia in Alzheimer's dis, atypical or mixed type, other symptoms, predominantly hallucinatory	1972291000006109
1972311000006112	Dementia in Alzheimer's dis, atypical or mixed type, other symptoms, predominantly depressive	1972311000006108
1972341000006111	Dementia in Alzheimer's dis, atypical or mixed type, other mixed symptoms	1972341000006107
1972371000006115	Dementia in Alzheimer's disease, unspecified, without additional symptoms	1972371000006104
1972401000006117	Dementia in Alzheimer's disease, unspecified, other symptoms, predominantly delusional	1972401000006101
1972431000006113	Mental & behav dis due to cannabinoids: resid & late-onset psychot dis, dementia	1972431000006109
1972451000006118	Dementia in Alzheimer's disease, unspecified, other symptoms, predominantly depressive	1972451000006102
1972471000006111	Dementia in Alzheimer's disease, unspecified, other mixed symptoms	1972471000006107
1972481000006114	Vascular dementia of acute onset, without additional symptoms	1972481000006105
1972501000006116	Vascular dementia of acute onset, other symptoms, predominantly delusional	1972501000006100
1972571000006110	Vascular dementia of acute onset, other mixed symptoms	1972571000006106
1972661000006119	Multi-infarct dementia, other symptoms, predominantly depressive	1972661000006103
1972681000006112	Multi-infarct dementia, other mixed symptoms	1972681000006108
1972711000006113	Subcortical vascular dementia, without additional symptoms	1972711000006109

1972731000006119	Subcortical vascular dementia, other symptoms, predominantly delusional	1972731000006103
1972751000006114	Subcortical vascular dementia, other symptoms, predominantly hallucinatory	1972751000006105
1972771000006116	Subcortical vascular dementia, other symptoms, predominantly depressive	1972771000006100
1972791000006115	Subcortical vascular dementia, other mixed symptoms	1972791000006104
1972821000006112	Mixed cortical and subcortical vascular dementia, without additional symptoms	1972821000006108
1972831000006110	Mixed cortical and subcortical vascular dementia, other symptoms, predominantly delusional	1972831000006106
1972871000006113	Mixed cortical and subcortical vascular dementia, other symptoms, predominantly hallucinatory	1972871000006109
1972911000006111	Mixed cortical and subcortical vascular dementia, other symptoms, predominantly depressive	1972911000006107
1972931000006117	Mixed cortical and subcortical vascular dementia, other mixed symptoms	1972931000006101
1973171000006112	Mental and behav dis due to vol solvents: resid & late-onset psychotic dis, dementia	1973171000006108
1973221000006118	Other vascular dementia, without additional symptoms	1973221000006102
1973271000006117	Other vascular dementia, other symptoms, predominantly delusional	1973271000006101
1973341000006118	Other vascular dementia, other symptoms, predominantly hallucinatory	1973341000006102
1973381000006112	Other vascular dementia, other symptoms, predominantly depressive	1973381000006108
1973401000006112	Other vascular dementia, other mixed symptoms	1973401000006108
1973461000006113	Vascular dementia, unspecified, without additional symptoms	1973461000006109
1973531000006117	Vascular dementia, unspecified, other symptoms, predominantly hallucinatory	1973531000006101
1973551000006112	Vascular dementia, unspecified, other symptoms, predominantly depressive	1973551000006108
1973711000006116	Mental and behav dis due to hallucinogens: resid & late-onset psychot dis, dementia	1973711000006100
1973941000006119	Mental & behav dis due to use cocaine: resid & late-onset psychot dis, dementia	1973941000006103
1974271000006119	Mental and behav dis mlti drg use/oth psych subs: resid/late psychot dis, dementia	1974271000006103
1974931000006114	Mental & behav dis due to tobacco: resid & late-onset psychot dis, dementia	1974931000006105
1975591000006119	Mental & behav dis due to use alcohol: resid & late-onset psychot dis, dementia	1975591000006103
1976091000006118	Mental and behav dis due to other stimulants inc caffeine: resid/late-onset psycht dis, dementia	1976091000006102
1976831000006111	Vascular dementia, unspecified, other mixed symptoms	1976831000006107
2502971000006115	Dementia associated with alcoholism	281004
2502981000006117	Alcohol-induced persisting dementia	281004
2575731000006115	Primary degenerative dementia of the Alzheimer type, senile onset, with delirium	4817008
2575741000006113	Dementia of the Alzheimer's type, with late onset, with delirium	4817008
2603271000006110	Primary degenerative dementia of the Alzheimer type, presenile onset, uncomplicated	6475002
2664601000006115	Multi-infarct dementia with delirium	10349009
2664611000006117	Vascular dementia, with delirium	10349009
2667431000006115	Primary degenerative dementia of the Alzheimer type, presenile onset, with depression	10532003
2722931000006114	Multi-infarct dementia with depression	14070001
2729731000000113	Presenile dementia with psychosis	1089501000000102
2729771000000110	Predominantly cortical dementia	1089521000000106
2729791000000114	Predominantly cortical vascular dementia	1089531000000108
2912321000006113	Multi-infarct dementia with delusions	25772007
2929931000006112	Primary degenerative dementia of the Alzheimer type, senile onset, with depression	26852004
2931251000006113	Alzheimer dementia	26929004
3029421000006116	Inhalant-induced persisting dementia	32875003
3392901000006118	Alzheimers dementia, late onset, with delusions	55009008
3414231000006117	MID - Multi-infarct dementia	56267009
3414251000006112	VAD - Vascular dementia	56267009
3414261000006114	Multi infarct dementia	56267009
3511181000006115	Parkinson-dementia complex of Guam	62239001
3575041000006117	Primary degenerative dementia of the Alzheimer type, senile onset, uncomplicated	66108005
3653671000006113	Multi-infarct dementia, uncomplicated	70936005
3802651000006111	Dementia of the Lewy body type	80098002
3964661000006114	Subcortical atherosclerotic dementia	90099008
5005421000006117	Amyotrophic lateral sclerosis with dementia	230258005
5005601000006112	Frontotemporal dementia	230270009
5005881000006116	Patchy dementia	230289009
5618261000006114	Dementia of frontal lobe type	278857002
5618271000006119	DFT - Dementia frontal lobe type	278857002
6024061000006111	Senile dementia of the Lewy body type	312991009
6024071000006116	Lewy body dementia	312991009

6348031000006112	Senile dementia with delusion	371024007
6348061000006115	Senile dementia with psychosis	371026009
6897211000006117	Primary degenerative dementia of the Alzheimer type, presenile onset	416780008
6897241000006118	Dementia of the Alzheimers type with early onset	416780008
6897251000006116	Presenile dementia, Alzheimer's type	416780008
6897271000006114	Dementia in Alzheimer's disease - type 2	416780008
6900181000006114	Primary degenerative dementia of the Alzheimer type, senile onset	416975007
6900201000006110	Dementia of the Alzheimers type, late onset	416975007
6900221000006117	SDAT - Senile dementia, Alzheimer's type	416975007
6900241000006112	Dementia in Alzheimer's disease - type 1	416975007
6964691000006110	Presenile dementia associated with AIDS	421023003
6973421000006116	Dementia associated with AIDS	421529006
6973461000006110	Acquired immune deficiency syndrome dementia complex	421529006
7043651000006119	Dementia associated with Parkinson's Disease	425390006
7043661000006117	Dementia associated with Parkinson Disease	425390006
7263021000006116	Dementia due to Huntingtons disease	442344002
7510731000006118	Dementia associated with normal pressure hydrocephalus	698625002
7510741000006111	Dementia associated with multiple sclerosis	698626001
7511421000006118	Post-traumatic dementia with behavioural change	698687007
7511961000006119	Dementia associated with neurosyphilis	698725008
7560531000006110	Frontotemporal dementia with parkinsonism-17	702429008
7710381000006117	Dementia co-occurrent with human immunodeficiency virus infection	713844000
7750421000006118	Right temporal atrophy variant frontotemporal dementia	716667005
7755121000006115	Behavioural variant of frontotemporal dementia	716994006
7840631000006118	Ischaemic vascular dementia	723123001
7843521000006113	Rapidly progressive dementia	723390000
7863681000006115	Epilepsy co-occurrent and due to dementia	724992007
7874761000006112	Delirium co-occurrent with dementia	725898002
7874771000006117	Delirium superimposed on dementia	725898002
7955111000006116	Dementia due to chronic subdural haematoma	733191004
7955161000006118	Dementia with Down syndrome	733194007
7969281000006110	Dementia of the Alzheimer type with behavioural disturbance	1581000119101
7969371000006115	Dementia with behavioural disturbance	1591000119103
8009521000006114	Dementia due to Picks disease	21921000119103
8009531000006112	Dementia due to Pick disease	21921000119103
8010111000006117	Primary degenerative dementia	22381000119105
8014401000006116	Early onset dementia with delusions	31081000119101
8024201000006111	Mixed dementia	79341000119107
8024641000006111	Altered behaviour in Huntington's dementia	82361000119107
8024671000006115	Dementia due to multiple sclerosis with altered behaviour	82371000119101
8024691000006119	Epileptic dementia with behavioural disturbance	82381000119103
8031751000006119	Dementia due to Parkinson's disease	101421000119107
8031761000006117	Dementia due to Parkinsons disease	101421000119107
8031771000006112	Dementia due to Parkinson disease	101421000119107
8033241000006113	Multi-infarct dementia due to atherosclerosis	106021000119105
8042791000006116	Lewy body dementia with behavioural disturbance	135811000119107
8042801000006115	Lewy body dementia with behavioral disturbance	135811000119107
8089231000006110	Vascular dementia with behavioural disturbance	288631000119104
8089241000006117	Vascular dementia with behavioral disturbance	288631000119104
9896131000006110	Behavioural disturbance co-occurrent and due to late onset Alzheimer dementia	16219201000119100
9902251000006116	Vascular dementia without behavioural disturbance	16276361000119108
12106351000006118	Behavioural and psychological symptoms of dementia	10171000132106
12106371000006112	BPSD - behavioral and psychological symptoms of dementia	10171000132106
12106381000006114	BPSD - behavioural and psychological symptoms of dementia	10171000132106
12106391000006112	Behavioral and psychological symptoms of dementia	10171000132106
12370641000006110	Dementia with mixed etiology	79341000119107
12370651000006112	MVAD - Mixed vascular Alzheimer dementia	79341000119107
12370661000006114	Dementia with mixed aetiology	79341000119107
13810191000006114	SDLT - senile dementia of Lewy body type	312991009
13909621000006120	Aggression due to dementia	788861009
13909641000006114	Agitation due to dementia	788862002
13913061000006118	Disinhibited behaviour due to dementia	789170003
13934381000006118	Cortical vascular dementia	833326008
14574821000006116	Dementia due to cobalamin deficiency	1186880002
14669521000006116	Dementia with AIDS (acquired immunodeficiency syndrome)	421529006

Hospital episode statistics: Dementia	
**icd**	**description**
F00	Alzheimer's dementia
F01	Vascular dementia
F02	Dementia in other diseases classified elsewhere
F03	Unspecified dementia
G30	Alzheimer's disease

Clinical codes for diabetes

GOLD: clinical, referral, tests; Aurum: consultations, observations.
Includes diabetes monitoring and health checks, but not referrals to diabetes clinics/education programmes, dietary advice, or structure diabetes education programmes

CPRD GOLD: Diabetes		
**medcode**	**readcode**	**readterm**
506	C100112	Non-insulin dependent diabetes mellitus
711	C10..00	Diabetes mellitus
758	C10F.00	Type 2 diabetes mellitus
1038	C100011	Insulin dependent diabetes mellitus
1323	F420.00	Diabetic retinopathy
1407	C10FJ00	Insulin treated Type 2 diabetes mellitus
1549	C10E.00	Type 1 diabetes mellitus
1647	C108.00	Insulin dependent diabetes mellitus
1682	C101.00	Diabetes mellitus with ketoacidosis
1684	66A4.00	Diabetic on oral treatment
2340	F381311	Diabetic amyotrophy
2342	F372.12	Diabetic neuropathy
2378	66AJ.00	Diabetic - poor control
2471	K01x100	Nephrotic syndrome in diabetes mellitus
2475	C104.11	Diabetic nephropathy
2986	F420200	Preproliferative diabetic retinopathy
3286	F420100	Proliferative diabetic retinopathy
3550	66A..00	Diabetic monitoring
3837	F420400	Diabetic maculopathy
4513	C109.00	Non-insulin dependent diabetes mellitus
5002	F372.11	Diabetic polyneuropathy
5884	C109.11	NIDDM - Non-insulin dependent diabetes mellitus
6125	66AS.00	Diabetic annual review
6509	C108700	Insulin dependent diabetes mellitus with retinopathy
6791	C108800	Insulin dependent diabetes mellitus - poor control
7059	8H2J.00	Admit diabetic emergency
7069	F420000	Background diabetic retinopathy
7328	M037200	Cellulitis in diabetic foot
7563	66A3.00	Diabetic on diet only
7795	C106.12	Diabetes mellitus with neuropathy
8403	C109700	Non-insulin dependent diabetes mellitus - poor control
8836	66AR.00	Diabetes management plan given
8842	66A5.00	Diabetic on insulin
9013	66AJ.11	Unstable diabetes
9835	2BBL.00	O/E - diabetic maculopathy present both eyes
9881	M271200	Mixed diabetic ulcer - foot
10098	C10yy00	Other specified diabetes mellitus with other spec comps
10099	F420300	Advanced diabetic maculopathy
10418	C10ED00	Type 1 diabetes mellitus with nephropathy
10659	F464000	Diabetic cataract
10692	C10EM00	Type 1 diabetes mellitus with ketoacidosis
10755	F420600	Non proliferative diabetic retinopathy
10977	66Ac.00	Diabetic peripheral neuropathy screening
11018	8HBG.00	Diabetic retinopathy 12 month review
11129	2BBQ.00	O/E - left eye background diabetic retinopathy
11433	2BBP.00	O/E - right eye background diabetic retinopathy
11471	8B3l.00	Diabetes medication review
11599	7276	Pan retinal photocoagulation for diabetes
11626	F420z00	Diabetic retinopathy NOS

11663	M271100	Neuropathic diabetic ulcer - foot
11848	C314.11	Renal diabetes
12455	C10E.11	Type I diabetes mellitus
12640	C10FC00	Type 2 diabetes mellitus with nephropathy
12736	C10F500	Type 2 diabetes mellitus with gangrene
13067	66AZ.00	Diabetic monitoring NOS
13071	66AI.00	Diabetic - good control
13097	2BBT.00	O/E - right eye proliferative diabetic retinopathy
13099	2BBR.00	O/E - right eye preproliferative diabetic retinopathy
13101	2BBV.00	O/E - left eye proliferative diabetic retinopathy
13102	2BBW.00	O/E - right eye diabetic maculopathy
13103	2BBS.00	O/E - left eye preproliferative diabetic retinopathy
13108	2BBX.00	O/E - left eye diabetic maculopathy
13194	9OL4.00	Diabetes monitoring 1st letter
13195	9OL5.00	Diabetes monitoring 2nd letter
13196	66AD.00	Fundoscopy - diabetic check
13197	9OL1.00	Attends diabetes monitoring
13279	C104y00	Other specified diabetes mellitus with renal complications
14803	C100100	Diabetes mellitus, adult onset, no mention of complication
14889	C100111	Maturity onset diabetes
15690	C103.00	Diabetes mellitus with ketoacidotic coma
16230	C106.00	Diabetes mellitus with neurological manifestation
16490	66AH.00	Diabetic treatment changed
16491	C106.13	Diabetes mellitus with polyneuropathy
16502	C104.00	Diabetes mellitus with renal manifestation
17067	F171100	Autonomic neuropathy due to diabetes
17095	2G5A.00	O/E - Right diabetic foot at risk
17247	F35z000	Diabetic mononeuritis NOS
17262	C109600	Non-insulin-dependent diabetes mellitus with retinopathy
17313	F440700	Diabetic iritis
17545	C108F11	Type I diabetes mellitus with diabetic cataract
17858	C108.12	Type 1 diabetes mellitus
17859	C109.12	Type 2 diabetes mellitus
17869	66AL.00	Diabetic-uncooperative patient
17886	66AM.00	Diabetic - follow-up default
18056	2G5C.00	Foot abnormality - diabetes related
18142	N030000	Diabetic cheiroarthropathy
18143	C109G11	Type II diabetes mellitus with arthropathy
18167	66AT.00	Annual diabetic blood test
18209	C109012	Type 2 diabetes mellitus with renal complications
18219	C109.13	Type II diabetes mellitus
18230	C108J12	Type 1 diabetes mellitus with neuropathic arthropathy
18264	C109J12	Insulin treated Type II diabetes mellitus
18278	C109J00	Insulin treated Type 2 diabetes mellitus
18311	68A7.00	Diabetic retinopathy screening
18387	C10E700	Type 1 diabetes mellitus with retinopathy
18390	C10FM00	Type 2 diabetes mellitus with persistent microalbuminuria
18425	C10FB00	Type 2 diabetes mellitus with polyneuropathy
18496	C10F600	Type 2 diabetes mellitus with retinopathy
18505	C108.11	IDDM-Insulin dependent diabetes mellitus
18642	C10EH00	Type 1 diabetes mellitus with arthropathy
18662	8HBH.00	Diabetic retinopathy 6 month review
18683	C10E500	Type 1 diabetes mellitus with ulcer
18747	8I6F.00	Diabetic retinopathy screening not indicated
18777	C10F000	Type 2 diabetes mellitus with renal complications
21482	C102.00	Diabetes mellitus with hyperosmolar coma
21689	13AB.00	Diabetic lipid lowering diet
21983	C108012	Type 1 diabetes mellitus with renal complications
22023	66AJz00	Diabetic - poor control NOS
22487	C10N.00	Secondary diabetes mellitus
22573	C106z00	Diabetes mellitus NOS with neurological manifestation
22823	66Ab.00	Diabetic foot examination
22871	C10EP00	Type 1 diabetes mellitus with exudative maculopathy
22884	C10F.11	Type II diabetes mellitus
22967	2BBF.00	Retinal abnormality - diabetes related
24327	M271000	Ischaemic ulcer diabetic foot

24363	8A13.00	Diabetic stabilisation
24423	C108.13	Type I diabetes mellitus
24458	C109711	Type II diabetes mellitus - poor control
24490	C100000	Diabetes mellitus, juvenile type, no mention of complication
24571	F372200	Asymptomatic diabetic neuropathy
24693	C109G00	Non-insulin dependent diabetes mellitus with arthropathy
24694	C108B00	Insulin dependent diabetes mellitus with mononeuropathy
24836	C109C12	Type 2 diabetes mellitus with nephropathy
25591	C10FQ00	Type 2 diabetes mellitus with exudative maculopathy
25627	C10F700	Type 2 diabetes mellitus - poor control
25636	66Aa.00	Diabetic diet - poor compliance
26054	C10FL00	Type 2 diabetes mellitus with persistent proteinuria
26604	66AY.00	Diabetic diet - good compliance
26664	2G5B.00	O/E - Left diabetic foot at risk
26666	2G5E.00	O/E - Right diabetic foot at low risk
26667	2G5I.00	O/E - Left diabetic foot at low risk
26855	C108400	Unstable insulin dependent diabetes mellitus
27891	N030100	Diabetic Charcot arthropathy
27921	2G51000	Foot abnormality - diabetes related
28769	66AV.00	Diabetic on insulin and oral treatment
29979	C109900	Non-insulin-dependent diabetes mellitus without complication
30294	C10EL00	Type 1 diabetes mellitus with persistent microalbuminuria
30323	C10EK00	Type 1 diabetes mellitus with persistent proteinuria
30477	F420700	High risk proliferative diabetic retinopathy
31053	R054300	[D]Widespread diabetic foot gangrene
31141	9OL8.00	Diabetes monitor.phone invite
31156	2G5J.00	O/E - Left diabetic foot at moderate risk
31157	2G5F.00	O/E - Right diabetic foot at moderate risk
31171	2G5G.00	O/E - Right diabetic foot at high risk
31172	2G5K.00	O/E - Left diabetic foot at high risk
31310	C108900	Insulin dependent diabetes maturity onset
31790	F372.00	Polyneuropathy in diabetes
32359	ZRbH.00	Perceived control of insulin-dependent diabetes
32403	C107.11	Diabetes mellitus with gangrene
32556	C107.12	Diabetes with gangrene
32627	C10FN00	Type 2 diabetes mellitus with ketoacidosis
33254	C105.00	Diabetes mellitus with ophthalmic manifestation
33343	C10y.00	Diabetes mellitus with other specified manifestation
33807	C107200	Diabetes mellitus, adult with gangrene
33969	C10A100	Malnutrition-related diabetes mellitus with ketoacidosis
34152	G73y000	Diabetic peripheral angiopathy
34268	C10F200	Type 2 diabetes mellitus with neurological complications
34283	C105z00	Diabetes mellitus NOS with ophthalmic manifestation
34450	C10FK00	Hyperosmolar non-ketotic state in type 2 diabetes mellitus
34912	C109400	Non-insulin dependent diabetes mellitus with ulcer
35105	C104100	Diabetes mellitus, adult onset, with renal manifestation
35107	C104z00	Diabetes mellitus with nephropathy NOS
35116	2G5L.00	O/E - Left diabetic foot - ulcerated
35288	C10E800	Type 1 diabetes mellitus - poor control
35316	2G5H.00	O/E - Right diabetic foot - ulcerated
35383	9OLD.00	Diabetic patient unsuitable for digital retinal photography
35385	C10FH00	Type 2 diabetes mellitus with neuropathic arthropathy
35399	C107.00	Diabetes mellitus with peripheral circulatory disorder
35785	F372100	Chronic painful diabetic neuropathy
36633	C109K00	Hyperosmolar non-ketotic state in type 2 diabetes mellitus
36798	7L10000	Continuous subcutaneous infusion of insulin
37315	F3y0.00	Diabetic mononeuropathy
37648	C109J11	Insulin treated non-insulin dependent diabetes mellitus
37806	C10FF00	Type 2 diabetes mellitus with peripheral angiopathy
38161	C108711	Type I diabetes mellitus with retinopathy
38617	C101y00	Other specified diabetes mellitus with ketoacidosis
38986	C100.00	Diabetes mellitus with no mention of complication
39070	C10EE00	Type 1 diabetes mellitus with hypoglycaemic coma
39317	C106100	Diabetes mellitus, adult onset, + neurological manifestation
39420	F381300	Myasthenic syndrome due to diabetic amyotrophy
39809	C108J00	Insulin dependent diab mell with neuropathic arthropathy

40023	C102000	Diabetes mellitus, juvenile type, with hyperosmolar coma
40401	C109500	Non-insulin dependent diabetes mellitus with gangrene
40682	C10E900	Type 1 diabetes mellitus maturity onset
40837	C10EN00	Type 1 diabetes mellitus with ketoacidotic coma
40962	C109H00	Non-insulin dependent d m with neuropathic arthropathy
41049	C108712	Type 1 diabetes mellitus with retinopathy
41389	C105100	Diabetes mellitus, adult onset, + ophthalmic manifestation
41686	Cyu2000	[X]Other specified diabetes mellitus
41716	C108C00	Insulin dependent diabetes mellitus with polyneuropathy
42505	C101z00	Diabetes mellitus NOS with ketoacidosis
42567	C103000	Diabetes mellitus, juvenile type, with ketoacidotic coma
42729	C108E11	Type I diabetes mellitus with hypoglycaemic coma
42762	C109612	Type 2 diabetes mellitus with retinopathy
42831	C10E200	Type 1 diabetes mellitus with neurological complications
43139	C102100	Diabetes mellitus, adult onset, with hyperosmolar coma
43227	C10F311	Type II diabetes mellitus with multiple complications
43785	C109D00	Non-insulin dependent diabetes mellitus with hypoglyca coma
43857	C10M.00	Lipoatrophic diabetes mellitus
43921	C10E400	Unstable type 1 diabetes mellitus
43951	66AK.00	Diabetic - cooperative patient
44033	F345000	Diabetic mononeuritis multiplex
44260	C108F00	Insulin dependent diabetes mellitus with diabetic cataract
44312	9M10.00	Informed dissent for diabetes national audit
44440	C108E00	Insulin dependent diabetes mellitus with hypoglycaemic coma
44443	C108500	Insulin dependent diabetes mellitus with ulcer
44779	C109E12	Type 2 diabetes mellitus with diabetic cataract
44982	C10FE00	Type 2 diabetes mellitus with diabetic cataract
45276	C10E312	Insulin dependent diabetes mellitus with multiple complicat
45467	C109B00	Non-insulin dependent diabetes mellitus with polyneuropathy
45491	C10z.00	Diabetes mellitus with unspecified complication
45499	K01x111	Kimmelstiel - Wilson disease
45913	C109712	Type 2 diabetes mellitus - poor control
45914	C108812	Type 1 diabetes mellitus - poor control
45919	C109212	Type 2 diabetes mellitus with neurological complications
46150	C109512	Type 2 diabetes mellitus with gangrene
46290	C108y00	Other specified diabetes mellitus with multiple comps
46301	C10EC00	Type 1 diabetes mellitus with polyneuropathy
46624	C10C.11	Maturity onset diabetes in youth
46850	C108811	Type I diabetes mellitus - poor control
46917	C10FD00	Type 2 diabetes mellitus with hypoglycaemic coma
46963	C108000	Insulin-dependent diabetes mellitus with renal complications
47315	C10F711	Type II diabetes mellitus - poor control
47321	C10F100	Type 2 diabetes mellitus with ophthalmic complications
47328	2BBk.00	O/E - right eye stable treated prolif diabetic retinopathy
47341	8A12.00	Diabetic crisis monitoring
47377	C105y00	Other specified diabetes mellitus with ophthalmic complicatn
47409	C109B11	Type II diabetes mellitus with polyneuropathy
47582	C10E000	Type 1 diabetes mellitus with renal complications
47584	F420500	Advanced diabetic retinal disease
47649	C10E100	Type 1 diabetes mellitus with ophthalmic complications
47650	C10E300	Type 1 diabetes mellitus with multiple complications
47816	C109H11	Type II diabetes mellitus with neuropathic arthropathy
47954	C10F900	Type 2 diabetes mellitus without complication
48078	F372000	Acute painful diabetic neuropathy
48192	C109E11	Type II diabetes mellitus with diabetic cataract
49074	C10F400	Type 2 diabetes mellitus with ulcer
49146	C108211	Type I diabetes mellitus with neurological complications
49276	C108100	Insulin-dependent diabetes mellitus with ophthalmic comps
49554	C10EF00	Type 1 diabetes mellitus with diabetic cataract
49640	2G5W.00	O/E - left chronic diabetic foot ulcer
49655	C10F611	Type II diabetes mellitus with retinopathy
49869	C109G12	Type 2 diabetes mellitus with arthropathy
49949	C10E411	Unstable type I diabetes mellitus
50175	66AW.00	Diabetic foot risk assessment
50225	C109011	Type II diabetes mellitus with renal complications
50429	C109100	Non-insulin-dependent diabetes mellitus with ophthalm comps

50527	C10FB11	Type II diabetes mellitus with polyneuropathy
50609	L180600	Pre-existing diabetes mellitus, non-insulin-dependent
50813	C109A11	Type II diabetes mellitus with mononeuropathy
50937	8HTe.00	Referral to diabetes preconception counselling clinic
50960	L180500	Pre-existing diabetes mellitus, insulin-dependent
50972	C100z00	Diabetes mellitus NOS with no mention of complication
51261	C10E.12	Insulin dependent diabetes mellitus
51697	C10G.00	Secondary pancreatic diabetes mellitus
51756	C10FP00	Type 2 diabetes mellitus with ketoacidotic coma
51957	C108511	Type I diabetes mellitus with ulcer
52041	2BBl.00	O/E - left eye stable treated prolif diabetic retinopathy
52104	C108300	Insulin dependent diabetes mellitus with multiple complicatn
52212	Cyu2.00	[X]Diabetes mellitus
52236	C10A.00	Malnutrition-related diabetes mellitus
52237	9360	Patient held diabetic record issued
52283	C108200	Insulin-dependent diabetes mellitus with neurological comps
52303	C109000	Non-insulin-dependent diabetes mellitus with renal comps
52630	2BBo.00	O/E - sight threatening diabetic retinopathy
53200	C101000	Diabetes mellitus, juvenile type, with ketoacidosis
53238	66AG.00	Diabetic drug side effects
53392	C10F911	Type II diabetes mellitus without complication
53634	R054200	[D]Gangrene of toe in diabetic
54008	C10EJ00	Type 1 diabetes mellitus with neuropathic arthropathy
54212	C109F00	Non-insulin-dependent d m with peripheral angiopath
54600	C10E412	Unstable insulin dependent diabetes mellitus
54856	C101100	Diabetes mellitus, adult onset, with ketoacidosis
54899	C109F11	Type II diabetes mellitus with peripheral angiopathy
55075	C109411	Type II diabetes mellitus with ulcer
55239	C10EQ00	Type 1 diabetes mellitus with gastroparesis
55431	L180X00	Pre-existing diabetes mellitus, unspecified
55842	C109200	Non-insulin-dependent diabetes mellitus with neuro comps
56268	C109D11	Type II diabetes mellitus with hypoglycaemic coma
56448	C108A00	Insulin-dependent diabetes without complication
56803	C107400	NIDDM with peripheral circulatory disorder
57278	C10F011	Type II diabetes mellitus with renal complications
57333	N030011	Diabetic cheiropathy
57389	93C4.00	Patient consent given for addition to diabetic register
57621	C108D00	Insulin dependent diabetes mellitus with nephropathy
58604	C109611	Type II diabetes mellitus with retinopathy
59253	C10FG00	Type 2 diabetes mellitus with arthropathy
59288	C103y00	Other specified diabetes mellitus with coma
59365	C109C00	Non-insulin dependent diabetes mellitus with nephropathy
59725	C109111	Type II diabetes mellitus with ophthalmic complications
59903	C106.11	Diabetic amyotrophy
59991	C10D.11	Maturity onset diabetes in youth type 2
60107	C108411	Unstable type I diabetes mellitus
60208	C108J11	Type I diabetes mellitus with neuropathic arthropathy
60499	C108600	Insulin dependent diabetes mellitus with gangrene
60699	C109F12	Type 2 diabetes mellitus with peripheral angiopathy
60796	C10FL11	Type II diabetes mellitus with persistent proteinuria
61071	C109D12	Type 2 diabetes mellitus with hypoglycaemic coma
61122	C10H.00	Diabetes mellitus induced by non-steroid drugs
61210	TJ23z00	Adverse reaction to insulins and antidiabetic agents NOS
61344	C108011	Type I diabetes mellitus with renal complications
61470	66AI.00	Diabetic monitoring - higher risk albumin excretion
61523	C106y00	Other specified diabetes mellitus with neurological comps
61557	8HKE.00	Diabetology D.V. requested
61670	889A.00	Diab mellit insulin-glucose infus acute myocardial infarct
61829	C108212	Type 1 diabetes mellitus with neurological complications
62107	C109511	Type II diabetes mellitus with gangrene
62146	C109300	Non-insulin-dependent diabetes mellitus with multiple comps
62209	C10EM11	Type I diabetes mellitus with ketoacidosis
62352	C108H11	Type I diabetes mellitus with arthropathy
62384	2G5V.00	O/E - right chronic diabetic foot ulcer
62613	C10EA11	Type I diabetes mellitus without complication
62674	C10FA00	Type 2 diabetes mellitus with mononeuropathy

63017	C108911	Type I diabetes mellitus maturity onset
63357	C107100	Diabetes mellitus, adult, + peripheral circulatory disorder
63371	C10y100	Diabetes mellitus, adult, + other specified manifestation
63690	C10FR00	Type 2 diabetes mellitus with gastroparesis
63762	C10z100	Diabetes mellitus, adult onset, + unspecified complication
64283	C10zy00	Other specified diabetes mellitus with unspecified comps
64357	C10zz00	Diabetes mellitus NOS with unspecified complication
64384	L180z00	Diabetes mellitus in pregnancy/childbirth/puerperium NOS
64446	C108G00	Insulin dependent diab mell with peripheral angiopathy
64449	C108z00	Unspecified diabetes mellitus with multiple complications
64571	C109C11	Type II diabetes mellitus with nephropathy
64668	C10FJ11	Insulin treated Type II diabetes mellitus
65025	C107z00	Diabetes mellitus NOS with peripheral circulatory disorder
65062	C103z00	Diabetes mellitus NOS with ketoacidotic coma
65267	C10F300	Type 2 diabetes mellitus with multiple complications
65463	F420800	High risk non proliferative diabetic retinopathy
65616	C108H00	Insulin dependent diabetes mellitus with arthropathy
65704	C109412	Type 2 diabetes mellitus with ulcer
66145	C10EN11	Type I diabetes mellitus with ketoacidotic coma
66274	66Ah.00	Insulin needles changed for each injection
66475	66Ak.00	Diabetic monitoring - lower risk albumin excretion
66675	C10A000	Malnutrition-related diabetes mellitus with coma
66872	C108D11	Type I diabetes mellitus with nephropathy
66965	C109H12	Type 2 diabetes mellitus with neuropathic arthropathy
67853	C106000	Diabetes mellitus, juvenile, + neurological manifestation
67905	C109211	Type II diabetes mellitus with neurological complications
68105	C10EB00	Type 1 diabetes mellitus with mononeuropathy
68390	C108512	Type 1 diabetes mellitus with ulcer
68792	C10z000	Diabetes mellitus, juvenile type, + unspecified complication
68843	C103100	Diabetes mellitus, adult onset, with ketoacidotic coma
68928	TJ23.00	Adverse reaction to insulins and antidiabetic agents
69124	C107300	IDDM with peripheral circulatory disorder
69278	C109E00	Non-insulin depend diabetes mellitus with diabetic cataract
69676	C10EA00	Type 1 diabetes mellitus without complication
69748	C105000	Diabetes mellitus, juvenile type, + ophthalmic manifestation
69993	C10E600	Type 1 diabetes mellitus with gangrene
70316	C109112	Type 2 diabetes mellitus with ophthalmic complications
70448	C107000	Diabetes mellitus, juvenile +peripheral circulatory disorder
70766	C108E12	Type 1 diabetes mellitus with hypoglycaemic coma
70821	C10yz00	Diabetes mellitus NOS with other specified manifestation
72320	C109A00	Non-insulin dependent diabetes mellitus with mononeuropathy
72345	C102z00	Diabetes mellitus NOS with hyperosmolar coma
72702	C10E812	Insulin dependent diabetes mellitus - poor control
83532	66Ao.00	Diabetes type 2 review
85660	66An.00	Diabetes type 1 review
85991	C10FM11	Type II diabetes mellitus with persistent microalbuminuria
91646	C10F411	Type II diabetes mellitus with ulcer
91942	C10E311	Type I diabetes mellitus with multiple complications
91943	C10EC11	Type I diabetes mellitus with polyneuropathy
93380	C10N100	Cystic fibrosis related diabetes mellitus
93468	C10EG00	Type 1 diabetes mellitus with peripheral angiopathy
93727	C10FE11	Type II diabetes mellitus with diabetic cataract
93875	C10E712	Insulin dependent diabetes mellitus with retinopathy
93878	C10E511	Type I diabetes mellitus with ulcer
93922	C104000	Diabetes mellitus, juvenile type, with renal manifestation
94383	C10N000	Secondary diabetes mellitus without complication
95343	C10E711	Type I diabetes mellitus with retinopathy
95351	C10FA11	Type II diabetes mellitus with mononeuropathy
95636	C10ER00	Latent autoimmune diabetes mellitus in adult
95992	C108A11	Type I diabetes mellitus without complication
96235	C10E911	Type I diabetes mellitus maturity onset
96506	C10G000	Secondary pancreatic diabetes mellitus without complication
97446	C108912	Type 1 diabetes mellitus maturity onset
97474	C108412	Unstable type 1 diabetes mellitus
97849	C10E912	Insulin dependent diabetes maturity onset
97894	C10EP11	Type I diabetes mellitus with exudative maculopathy

98071	C10E112	Insulin-dependent diabetes mellitus with ophthalmic comps
98392	C10C.12	Maturity onset diabetes in youth type 1
98616	C10F211	Type II diabetes mellitus with neurological complications
98704	C10E512	Insulin dependent diabetes mellitus with ulcer
98723	C10FD11	Type II diabetes mellitus with hypoglycaemic coma
99231	C108B11	Type I diabetes mellitus with mononeuropathy
99311	C10E111	Type I diabetes mellitus with ophthalmic complications
99628	Kyu0300	[X]Glomerular disorders in diabetes mellitus
99716	C10EE12	Insulin dependent diabetes mellitus with hypoglycaemic coma
99719	C10EA12	Insulin-dependent diabetes without complication
100292	Cyu2300	[X]Unspecified diabetes mellitus with renal complications
100347	C10A500	Malnutritn-relat diabetes melitus wth periph circul complctn
100422	8HgC.00	Discharged from diabetes shared care programme
100770	C10EF12	Insulin dependent diabetes mellitus with diabetic cataract
100964	C10F111	Type II diabetes mellitus with ophthalmic complications
101311	C10EC12	Insulin dependent diabetes mellitus with polyneuropathy
101735	C10E212	Insulin-dependent diabetes mellitus with neurological comps
101801	66At100	Type II diabetic dietary review
101881	2BBr.00	Impaired vision due to diabetic retinopathy
102112	C10E611	Type I diabetes mellitus with gangrene
102163	C10ED12	Insulin dependent diabetes mellitus with nephropathy
102201	C10FC11	Type II diabetes mellitus with nephropathy
102620	C10EL11	Type I diabetes mellitus with persistent microalbuminuria
102704	66At000	Type I diabetic dietary review
102740	C108112	Type 1 diabetes mellitus with ophthalmic complications
102946	C10E012	Insulin-dependent diabetes mellitus with renal complications
103902	C10FG11	Type II diabetes mellitus with arthropathy
104323	C10F511	Type II diabetes mellitus with gangrene
104453	66At011	Type 1 diabetic dietary review
104639	C10FF11	Type II diabetes mellitus with peripheral angiopathy
105302	K08yA00	Proteinuric diabetic nephropathy
105337	C10E811	Type I diabetes mellitus - poor control
105740	2G5d.00	O/E - Left diabetic foot at increased risk
105741	2G5e.00	O/E - Right diabetic foot at increased risk
105784	C109912	Type 2 diabetes mellitus without complication
106061	C10FP11	Type II diabetes mellitus with ketoacidotic coma
106269	9m0..00	Diabetic retinopathy screening administrative status
106360	K27y700	Erectile dysfunction due to diabetes mellitus
106528	C10FN11	Type II diabetes mellitus with ketoacidosis
107452	66o..00	Further diabetic monitoring
107701	C10FK11	Hyperosmolar non-ketotic state in type II diabetes mellitus
107881	K08yA11	Clinical diabetic nephropathy
108005	C109312	Type 2 diabetes mellitus with multiple complications
108007	C108311	Type I diabetes mellitus with multiple complications
108724	C10EQ11	Type I diabetes mellitus with gastroparesis
109051	C10E612	Insulin dependent diabetes mellitus with gangrene
109103	C109911	Type II diabetes mellitus without complication
109133	L180700	Pre-existing malnutrition-related diabetes mellitus
109197	C10FH11	Type II diabetes mellitus with neuropathic arthropathy
109760	1M8..00	Diabetic peripheral neuropathic pain
109837	C10E011	Type I diabetes mellitus with renal complications
109865	C109B12	Type 2 diabetes mellitus with polyneuropathy
110344	66o2.00	Diabetic on non-insulin injectable medication
110379	66o5.00	Diabetic on oral treatment and glucagon-like peptide 1
110393	13B1000	Diabetic carbohydrate counting diet
110400	C108F12	Type 1 diabetes mellitus with diabetic cataract
110997	C10y000	Diabetes mellitus, juvenile, + other specified manifestation
111106	C108A12	Type 1 diabetes mellitus without complication
111483	66o6.00	Diabetic on insulin and glucagon-like peptide 1
111798	C10FQ11	Type II diabetes mellitus with exudative maculopathy
112365	Lyu2900	[X]Pre-existing diabetes mellitus, unspecified
112402	C107y00	Other specified diabetes mellitus with periph circ comps
113115	C10Q.00	Maturity onset diabetes of the young type 5
113197	C108D12	Type 1 diabetes mellitus with nephropathy
113495	C109311	Type II diabetes mellitus with multiple complications
113609	C10FR11	Type II diabetes mellitus with gastroparesis

113863	C10ED11	Type I diabetes mellitus with nephropathy
113975	C108C11	Type I diabetes mellitus with polyneuropathy
114401	C108612	Type 1 diabetes mellitus with gangrene
114439	C10A200	Malnutrition-related diabetes mellitus with renal complicatn
115410	C108611	Type I diabetes mellitus with gangrene
115541	C10A400	Malnutrition-related diabetes mellitus wth neuro complicatns
115855	C10A700	Malnutrition-related diabetes mellitus without complications

CPRD Aurum: Diabetes		
**medcode**	**term**	**snomedconceptID**
302011	Diabetic peripheral angiopathy	127014009
9093013	Diabetic retinopathy	4855003
15518018	Secondary diabetes mellitus	8801005
65526011	Diabetic amyotrophy	39058009
73294018	Diabetic cataract	43959009
73466011	Non-insulin dependent diabetes mellitus	44054006
77727018	Insulin dependent diabetes mellitus	73211009
82373015	Diabetic polyneuropathy	49455004
96244016	Protein-deficient diabetes mellitus	57886004
98476015	Proliferative diabetic retinopathy	59276001
121589010	Diabetes mellitus	73211009
125705011	Diabetes mellitus due to insulin receptor antibodies	75682002
178798017	Diabetic ketoacidosis without coma	111556005
197761014	Type 2 diabetes mellitus	44054006
197984010	Type 1 diabetes mellitus	46635009
205225016	Diabetic nephropathy	127013003
216201011	Diabetic retinopathy screening	134395001
259365016	Glucose tolerance test indicates diabetes mellitus	166928007
264676010	Diabetic monitoring	170742000
264679015	Diabetic on diet only	170745003
264681018	Diabetic on oral treatment	170746002
264682013	Diabetic on insulin	170747006
264705017	Diabetic treatment changed	170761001
264707013	Diabetic - good control	170763003
264716012	Diabetic - poor control NOS	268519009
264717015	Diabetic - cooperative patient	170769004
264718013	Diabetic-uncooperative patient	170770003
264719017	Diabetic - follow-up default	170771004
264726017	Diabetes management plan given	170742000
264727014	Diabetic annual review	170777000
264728016	Annual diabetic blood test	170778005
264729012	Diabetic monitoring NOS	170742000
282568013	Diabetic crisis monitoring	182780004
282570016	Diabetic stabilisation	182781000
283544013	Diabetic emergency hospital admission	183472000
283889016	Diabetology D.V. requested	183722003
285751016	Attends diabetes monitoring	185753003
292466013	Diabetes mellitus without complication	111552007
292475010	Diabetes mellitus NOS with no mention of complication	111552007
292478012	Other specified diabetes mellitus with ketoacidosis	420422005
292479016	Diabetes mellitus NOS with ketoacidosis	420422005
292480018	Diabetes mellitus with hyperosmolar coma	422126006
292482014	Diabetes mellitus, juvenile type, with hyperosmolar coma	190330002
292483016	Diabetes mellitus, adult onset, with hyperosmolar coma	190331003
292484010	Hyperosmolar coma associated with diabetes mellitus	422126006
292488013	Coma associated with diabetes mellitus	420662003
292489017	Diabetes mellitus NOS with ketoacidotic coma	420422005
292495016	Other specified diabetes mellitus with renal complications	127013003
292496015	Diabetes mellitus with nephropathy NOS	127013003
292503016	Diabetes mellitus NOS with ophthalmic manifestation	25093002
292512019	Diabetes mellitus NOS with neurological manifestation	422088007
292523015	Diabetes mellitus NOS with peripheral circulatory disorder	421895002
292538019	Type I diabetes mellitus with ulcer	190368000
292540012	Type 1 diabetes mellitus with ulcer	190368000
292541011	Type 1 diabetes mellitus with gangrene	420825003
292543014	Type I diabetes mellitus with gangrene	420825003

292548017	Type 1 diabetes mellitus - poor control	444073006
292550013	Type I diabetes mellitus - poor control	444073006
292551012	Type I diabetes mellitus maturity onset	190372001
292553010	Type 1 diabetes mellitus maturity onset	190372001
292565014	Diabetes mellitus with multiple complications	385041000000108
292576013	Type II diabetes mellitus with multiple complications	190388001
292577016	Type 2 diabetes mellitus with multiple complications	190388001
292579018	Type 2 diabetes mellitus with ulcer	190389009
292581016	Type II diabetes mellitus with ulcer	190389009
292582011	Type II diabetes mellitus with gangrene	421631007
292583018	Type 2 diabetes mellitus with gangrene	421631007
292589019	Type II diabetes mellitus - poor control	443694000
292590011	Type II diabetes mellitus poorly controlled	443694000
292606016	Malnutrition-related diabetes mellitus with ketoacidosis	190406000
292612014	Malnutrition-related diabetes mellitus without complications	190412005
292617015	Diabetes mellitus with other specified manifestation	74627003
292621010	Diabetes mellitus NOS with other specified manifestation	73211009
292622015	Diabetes mellitus with unspecified complication	74627003
292626017	Diabetic complication	74627003
293756010	[X]Other specified diabetes mellitus	73211009
293759015	Diabetic renal disease	127013003
297492014	Diabetic mononeuritis multiplex	193141005
297510017	Diabetic mononeuritis NOS	193141005
297550019	Acute painful diabetic neuropathy	193183000
297551015	Chronic painful diabetic neuropathy	193184006
297552010	Asymptomatic diabetic neuropathy	193185007
297754014	Preproliferative diabetic retinopathy	193349004
297755010	Advanced diabetic maculopathy	193350004
297758012	Diabetic retinopathy NOS	4855003
297964010	Diabetic iritis	193489006
303846016	Nephrotic syndrome in diabetes mellitus	197605007
305158015	Diabetic glomerulopathy	309426007
306112011	Pre-existing diabetes mellitus, insulin-dependent	385051000000106
306113018	Pre-existing diabetes mellitus, non-insulin-dependent	199230006
306114012	Pre-existing malnutrition-related diabetes mellitus	199231005
308110013	Pre-existing diabetes mellitus	385051000000106
308360012	Cellulitis in diabetic foot	200687002
309177010	Ischaemic ulcer diabetic foot	201250006
309179013	Neuropathic diabetic ulcer - foot	201251005
309180011	Mixed diabetic ulcer - foot	201252003
309737011	Diabetic cheiroarthropathy	201723002
309738018	Diabetic cheiropathy	201723002
317347010	[D]Gangrene of toe in diabetic	195302000
317348017	[D]Widespread diabetic foot gangrene	195303005
345486016	Diabetic neuropathy	230572002
345487013	Diabetes mellitus with neuropathy	230572002
345492010	Diabetic mononeuropathy	230577008
347657010	Diabetic maculopathy	232020009
354316011	Kidney disorder due to diabetes mellitus	127013003
354508018	Clinical diabetic nephropathy	236500003
354509014	Proteinuric diabetic nephropathy	236500003
356078011	Secondary endocrine diabetes mellitus	237601000
356085010	Maturity onset diabetes in youth type 2	237604008
356110010	Megaloblastic anaemia, thiamine-responsive, with diabetes mellitus and sensorineural deafness	237617006
356111014	Insulin-dependent diabetes mellitus secretory diarrhoea syndrome	237618001
356118015	Diabetes-deafness syndrome maternally transmitted	237619009
356119011	Abnormal metabolic state in diabetes mellitus	237620003
356121018	Diabetic severe hyperglycaemia	237621004
399419012	Myasthenic syndrome due to diabetic amyotrophy	267604001
401531012	Diabetic - poor control	268519009
411891014	Unstable diabetes	11530004
429970018	Unstable type I diabetes mellitus	290002008
429971019	Unstable type 1 diabetes mellitus	290002008
429972014	Unstable insulin dependent diabetes mellitus	11530004
451410016	O/E - Right diabetic foot at risk	308105005

451411017	O/E - Left diabetic foot at risk	308106006
454092018	Diabetic hyperosmolar non-ketotic state	310505005
454093011	HONKS - Diabetic hyperosmolar non-ketotic state	310505005
455408014	Advanced diabetic retinal disease	311782002
457325013	Type I diabetes mellitus without complication	313435000
457326014	Type 1 diabetes mellitus without complication	313435000
457327017	Insulin-dependent diabetes without complication	111552007
457328010	Non-insulin-dependent diabetes mellitus without complication	313436004
457329019	Type 2 diabetes mellitus without complication	313436004
457330012	Type II diabetes mellitus without complication	313436004
458512016	Diabetic on insulin and oral treatment	314194001
459161015	Type 1 diabetes mellitus with hypoglycaemic coma	314771006
459162010	Insulin dependent diabetes mellitus with hypoglycaemic coma	237632004
459163017	Type I diabetes mellitus with hypoglycaemic coma	314771006
459167016	Type 2 diabetes mellitus with hypoglycaemic coma	719216001
459169018	Hypoglycaemic coma co-occurrent and due to diabetes mellitus type II	719216001
459292011	Type 1 diabetes mellitus with arthropathy	314893005
459293018	Insulin dependent diabetes mellitus with arthropathy	39710007
459294012	Type I diabetes mellitus with arthropathy	314893005
459296014	Type 1 diabetes mellitus with neuropathic arthropathy	71771000119100
459306016	Type II diabetes mellitus with peripheral angiopathy	314902007
459308015	Type 2 diabetes mellitus with peripheral angiopathy	314902007
459309011	Non-insulin dependent diabetes mellitus with arthropathy	314903002
459310018	Type 2 diabetes mellitus with arthropathy	314903002
459311019	Type II diabetes mellitus with arthropathy	314903002
459312014	Type II diabetes mellitus with neuropathic arthropathy	314904008
459313016	Type 2 diabetes mellitus with neuropathic arthropathy	314904008
459694018	Diabetic lipid lowering diet	315207000
483882011	Maturity-onset diabetes of the young	609561005
483886014	Maturity onset diabetes in youth	609561005
493773010	NIDDM - Non-insulin dependent diabetes mellitus	44054006
493774016	Type II diabetes mellitus	44054006
494564012	Type I diabetes mellitus	46635009
1223147012	Insulin treated Type II diabetes mellitus	237599002
1223148019	Insulin treated non-insulin dependent diabetes mellitus	237599002
1230890017	Autonomic neuropathy due to diabetes	50620007
1230929011	Secondary pancreatic diabetes mellitus	51002006
1484867016	Non proliferative diabetic retinopathy	390834004
1484887015	O/E - diabetic maculopathy present both eyes	390854003
1488393013	O/E - Right diabetic foot at low risk	394671009
1488394019	O/E - Right diabetic foot at high risk	394672002
1488395018	O/E - Right diabetic foot - ulcerated	394673007
1488396017	O/E - Left diabetic foot - ulcerated	394674001
1488397014	O/E - Left diabetic foot at low risk	394675000
1488398016	O/E - Left diabetic foot at high risk	394676004
1488403015	O/E - Left diabetic foot at moderate risk	394681008
1488404014	O/E - Right diabetic foot at moderate risk	394682001
1488441010	Diabetes medication review	394725008
1488898011	Hyperosmolar non-ketotic state in type 2 diabetes mellitus	395204000
1780286017	Diabetic peripheral neuropathy screening	401081006
1780311019	Type 1 diabetes mellitus with persistent microalbuminuria	401110002
1780386019	Diabetic foot examination	401191002
1785163015	High risk proliferative diabetic retinopathy	312907002
1785332013	Background diabetic retinopathy	390834004
2159948010	Diabetic retinopathy 12 month review	408384004
2159949019	Diabetic retinopathy 6 month review	408385003
2159960011	Diabetic retinopathy screening not indicated	408396006
2159973010	O/E - right eye background diabetic retinopathy	408409007
2159974016	O/E - left eye background diabetic retinopathy	408410002
2159975015	O/E - right eye preproliferative diabetic retinopathy	408411003
2159976019	O/E - left eye preproliferative diabetic retinopathy	408412005
2159977011	O/E - right eye proliferative diabetic retinopathy	408413000
2159978018	O/E - left eye proliferative diabetic retinopathy	408414006
2159979014	Maculopathy of right eye with diabetes mellitus	769244003
2159980012	Maculopathy of left eye with diabetes mellitus	769245002
2474726011	Pan retinal photocoagulation for diabetes	413180006

2476117016	Maturity onset diabetes in youth type 1	609562003
2532967014	Clinically significant macular oedema of right eye due to diabetes mellitus	769221001
2532976019	O/E - left chronic diabetic foot ulcer	414890007
2532977011	O/E - right chronic diabetic foot ulcer	414906009
2549896013	O/E - sight threatening diabetic retinopathy	417677008
2549901010	Diabetic patient unsuitable for digital retinal photography	417681008
2622193012	Diabetes mellitus with ketoacidosis	420422005
2674067015	Latent autoimmune diabetes mellitus in adult	426875007
2817479019	Hyperglycaemic crisis in diabetes mellitus	441656006
3513752015	Absence of lower limb due to diabetes mellitus	735200002
3514795011	Hyperosmolar hyperglycaemic coma due to diabetes mellitus without ketoacidosis	735537007
3515955014	Diabetes self management plan	735985000
3527174019	Diabetes mellitus caused by chemical	737212004
3636751012	Acute complication with diabetes mellitus	762489000
3636752017	Acute complication co-occurrent and due to diabetes mellitus	762489000
13751000006117	Other specified diabetes mellitus with multiple comps	385041000000108
13761000006115	Other specified diabetes mellitus with neurological comps	422088007
13771000006110	Other specified diabetes mellitus with ophthalmic complicatn	25093002
13781000006113	Other specified diabetes mellitus with other spec comps	73211009
13791000006111	Other specified diabetes mellitus with periph circ comps	421895002
13811000006110	Other specified diabetes mellitus with unspecified comps	74627003
63021000000116	Good compliance with diabetic diet	25351000000101
63041000000111	Poor compliance with diabetic diet	25361000000103
72651000006114	Unstable insulin dependent diabetes mellitus	11530004
72711000006117	Unstable type 1 diabetes mellitus	290002008
72721000006113	Unstable type I diabetes mellitus	290002008
84281000006115	Type 1 diabetes mellitus	46635009
84291000006117	Type 1 diabetes mellitus - poor control	444073006
84301000006116	Type 1 diabetes mellitus maturity onset	190372001
84311000006118	Type 1 diabetes mellitus with arthropathy	314893005
84321000006114	Type 1 diabetes mellitus with diabetic cataract	421920002
84331000006112	Type 1 diabetes mellitus with gangrene	420825003
84341000006119	Type 1 diabetes mellitus with hypoglycaemic coma	314771006
84361000006115	Type 1 diabetes mellitus with multiple complications	422228004
84371000006110	Type 1 diabetes mellitus with nephropathy	421893009
84381000006113	Type 1 diabetes mellitus with neurological complications	421468001
84391000006111	Type 1 diabetes mellitus with neuropathic arthropathy	71771000119100
84401000006113	Type 1 diabetes mellitus with ophthalmic complications	739681000
84421000006115	Type 1 diabetes mellitus with polyneuropathy	713705003
84431000006117	Type 1 diabetes mellitus with renal complications	421893009
84441000006110	Type 1 diabetes mellitus with retinopathy	420789003
84451000006112	Type 1 diabetes mellitus with ulcer	190368000
84461000006114	Type 1 diabetes mellitus without complication	313435000
84471000006119	Type 2 diabetes mellitus	44054006
84481000006116	Type 2 diabetes mellitus - poor control	443694000
84491000006118	Type 2 diabetes mellitus with arthropathy	314903002
84501000006114	Type 2 diabetes mellitus with diabetic cataract	420756003
84511000006112	Type 2 diabetes mellitus with gangrene	421631007
84521000006116	Type 2 diabetes mellitus with hypoglycaemic coma	719216001
84531000006118	Type 2 diabetes mellitus with mononeuropathy	420436000
84541000006111	Type 2 diabetes mellitus with multiple complications	190388001
84551000006113	Type 2 diabetes mellitus with nephropathy	420279001
84561000006110	Type 2 diabetes mellitus with neurological complications	421326000
84571000006115	Type 2 diabetes mellitus with neuropathic arthropathy	314904008
84581000006117	Type 2 diabetes mellitus with ophthalmic complications	422099009
84591000006119	Type 2 diabetes mellitus with peripheral angiopathy	314902007
84601000006110	Type 2 diabetes mellitus with polyneuropathy	713706002
84611000006113	Type 2 diabetes mellitus with renal complications	420279001
84621000006117	Type 2 diabetes mellitus with retinopathy	422034002
84631000006119	Type 2 diabetes mellitus with ulcer	190389009
84641000006112	Type 2 diabetes mellitus without complication	313436004
84651000006114	Type I diabetes mellitus	46635009
84661000006111	Type I diabetes mellitus poorly controlled	444073006
84671000006116	Type I diabetes mellitus maturity onset	190372001
84681000006118	Type I diabetes mellitus with arthropathy	314893005

84691000006115	Cataract due to diabetes mellitus type 1	421920002
84701000006115	Type I diabetes mellitus with gangrene	420825003
84711000006117	Type I diabetes mellitus with hypoglycaemic coma	314771006
84721000006113	Mononeuropathy with type 1 diabetes mellitus	420918009
84731000006111	Multiple complications of type 1 diabetes mellitus	422228004
84741000006118	Type I diabetes mellitus with nephropathy	421893009
84751000006116	Type I diabetes mellitus with neurological complications	421468001
84761000006119	Type I diabetes mellitus with neuropathic arthropathy	71771000119100
84771000006114	Disorder of eye with type 1 diabetes mellitus	739681000
84781000006112	Type I diabetes mellitus with peripheral angiopathy	31211000119101
84791000006110	Polyneuropathy due to diabetes mellitus type I	713705003
84801000006111	Type I diabetes mellitus with renal complications	421893009
84811000006114	Type I diabetes mellitus with retinopathy	420789003
84821000006118	Type I diabetes mellitus with ulcer	190368000
84831000006115	Type I diabetes mellitus without complication	313435000
84841000006113	Type II diabetes mellitus	44054006
84851000006110	Type II diabetes mellitus - poor control	443694000
84861000006112	Type II diabetes mellitus with arthropathy	314903002
84871000006117	Cataract due to diabetes mellitus type 2	420756003
84881000006119	Type II diabetes mellitus with gangrene	421631007
84891000006116	Type II diabetes mellitus with hypoglycaemic coma	719216001
84901000006117	Mononeuropathy with type 2 diabetes mellitus	420436000
84911000006119	Type II diabetes mellitus with multiple complications	190388001
84921000006110	Renal disorder due to type 2 diabetes mellitus	420279001
84941000006115	Type II diabetes mellitus with neuropathic arthropathy	314904008
84951000006118	Disorder of eye with type 2 diabetes mellitus	422099009
84961000006116	Type II diabetes mellitus with peripheral angiopathy	314902007
84971000006111	Type II diabetes mellitus with polyneuropathy	713706002
84981000006114	Type II diabetes mellitus with renal complications	420279001
84991000006112	Type II diabetes mellitus with retinopathy	422034002
85001000006117	Type II diabetes mellitus with ulcer	190389009
85011000006119	Type II diabetes mellitus without complication	313436004
169731000006118	Retinal abnormality - diabetes-related	4855003
189711000000119	Lipoatrophic diabetes mellitus without complication	112991000000101
189721000000113	Secondary diabetes mellitus without complication	8801005
189841000000113	Informed dissent for diabetes national audit	113141000000103
198461000000116	Diabetes mellitus associated with pancreatic disease	51002006
213141000006111	Pre-existing diabetes mellitus, unspecified	385051000000106
214921000006116	Polyneuropathy in diabetes	49455004
223291000000111	Maturity onset diabetes	44054006
280511000006113	Non-insulin dependent diabetes mellitus with hypoglyca coma	719216001
280521000006117	Non-insulin dependent diabetes mellitus with mononeuropathy	420436000
280531000006119	Non-insulin dependent diabetes mellitus with nephropathy	420279001
280541000006112	Non-insulin dependent diabetes mellitus with polyneuropathy	713706002
280551000006114	Non-insulin-dependent diabetes mellitus with ulcer	190389009
280561000006111	Non-insulin-dependent diabetes mellitus with peripheral angiopathy	314902007
280571000006116	Non-insulin dependent diabetes mellitus	44054006
280581000006118	Non-insulin-dependent diabetes mellitus with multiple complications	190388001
280591000006115	Non-insulin-dependent diabetes mellitus with neuro comps	421326000
281161000006114	Non-insulin depend diabetes mellitus with diabetic cataract	420756003
281171000006119	Type II diabetes mellitus uncontrolled	443694000
281181000006116	Non-insulin dependent diabetes mellitus with neuropathic arthropathy	314904008
281211000006117	Gangrene associated with type 2 diabetes mellitus	421631007
299601000000114	Gastroparesis with type 1 diabetes mellitus	713702000
299621000000117	Gastroparesis with type 2 diabetes mellitus	713703005
301611000000118	Diabetes monitoring lower risk albumin excretion	199481000000100
301641000000117	Diabetes monitoring higher risk albumin excretion	199491000000103
308071000000112	Patient consent given for addition to diabetic register	202151000000105
377001000006117	[X]Diabetes mellitus	73211009
396661000006115	Malnutrition-related diabetes mellitus with multiple complications	190411003
396671000006110	[X]Malnutrit-related diabetes mellitus with unspec complics	190411003
494831000000119	Diabetes mellitus associated with cystic fibrosis	426705001
587111000006111	Non-insulin-dependent diabetes mellitus with ophthalm comps	422099009
587521000006111	Non-insulin-dependent diabetes mellitus with renal comps	420279001
607851000006113	Date diabetic treatment stopped	170773001
616081000006113	Diabetes mellitus insulin-glucose infusion in acute myocardial infarction	315287002

616351000006115	Diabetes mellitus with gangrene	422275004
616381000006111	Diabetes mellitus with ketoacidotic coma	26298008
616391000006114	Nervous system disorder due to diabetes mellitus	422088007
616421000006118	Eye disorder due to diabetes mellitus	25093002
616441000006113	Peripheral vascular disorder due to diabetes mellitus	421895002
616451000006110	Diabetes mellitus with polyneuropathy	49455004
616461000006112	Diabetes mellitus with renal manifestation	127013003
616481000006119	Diabetes mellitus, adult onset, + neurological manifestation	421326000
616491000006116	Diabetes mellitus, adult onset, + ophthalmic manifestation	422099009
616501000006112	Disorder due to type 2 diabetes mellitus	422014003
616511000006110	Diabetes mellitus, adult onset, no mention of complication	313436004
616531000006116	Diabetes mellitus, adult onset, with ketoacidosis	421750000
616541000006114	Diabetes mellitus, adult onset, with ketoacidotic coma	421847006
616551000006111	Diabetes mellitus, adult onset, with renal manifestation	420279001
616561000006113	Diabetes mellitus, adult with gangrene	421631007
616571000006118	Diabetes mellitus, adult, + other specified manifestation	73211009
616581000006115	Diabetes mellitus, adult, + peripheral circulatory disorder	422166005
616591000006117	Diabetes mellitus, juvenile +peripheral circulatory disorder	421365002
616601000006113	Diabetes mellitus, juvenile type, + ophthalmic manifestation	739681000
616611000006111	Disorder due to type 1 diabetes mellitus	420868002
616621000006115	Type 1 diabetes mellitus without complication	313435000
616641000006110	Diabetes mellitus, juvenile type, with ketoacidosis	420270002
616651000006112	Diabetes mellitus, juvenile type, with ketoacidotic coma	421075007
616661000006114	Diabetes mellitus, juvenile type, with renal manifestation	421893009
616671000006119	Diabetes mellitus, juvenile, + neurological manifestation	421468001
616681000006116	Diabetes mellitus, juvenile, + other specified manifestation	73211009
616691000006118	Diabetes monitoring check done	270445003
616731000006114	Diabetes monitoring first letter	185756006
616741000006116	Diabetes monitoring second letter	185757002
616751000006119	Diabetes monitoring third letter	185758007
616831000006118	Diabetes with gangrene	422275004
616891000006119	Diabetic - moderate control	616891000006103
616921000006113	Diabetic amyotrophy	39058009
616971000006114	Diabetic Charcot's arthropathy	201724008
641581000006115	Non-insulin-dependent diabetes mellitus with retinopathy	422034002
674961000006118	Peripheral circulatory disorder associated with type 2 diabetes mellitus	422166005
719471000006119	Malnutrit-related diabetes mellitus with unspec complics	190411003
719481000006116	Malnutrit-related diabetes mellitus wth ophthalmic complicat	421256007
719531000006118	Malnutrition related diabetes mellitus	75524006
719541000006111	Malnutrition-related diabetes mellitus with coma	420996007
719561000006110	Malnutrition-related diabetes mellitus with multiple comps	190411003
719571000006115	Malnutrition-related diabetes mellitus with renal complications	190407009
719591000006119	Malnutrition-related diabetes mellitus wth neuro complicatns	420683009
719601000006110	Malnutrition-related diabetes mellitus with peripheral circulatory complications	190410002
720311000006119	Malnutrit-relat diabetes mellitus with other spec comps	190411003
733161000000116	Impaired vision due to diabetic retinopathy	373041000000101
746791000006111	Insulin-dependent diabetes without complication	111552007
764191000006112	Foot abnormality - diabetes related	280137006
764201000006110	Foot abnormality - diabetes related	280137006
771331000006116	Insulin dependent diabetes mellitus with arthropathy	39710007
771341000006114	Insulin dependent diabetes mellitus with diabetic cataract	43959009
771351000006111	Insulin dependent diabetes mellitus with gangrene	422275004
771361000006113	Insulin dependent diabetes mellitus with hypoglycaemic coma	237632004
771371000006118	Insulin dependent diabetes mellitus with mononeuropathy	230577008
771381000006115	Insulin dependent diabetes mellitus with multiple complicatn	385041000000108
771391000006117	Insulin dependent diabetes mellitus with nephropathy	127013003
771401000006115	Insulin dependent diabetes mellitus with polyneuropathy	49455004
771411000006117	Insulin dependent diabetes mellitus with retinopathy	4855003
771421000006113	Insulin dependent diabetes mellitus with ulcer	422183001
771481000006112	Insulin-dependent diabetes mellitus with neurological comps	422088007
771491000006110	Insulin-dependent diabetes mellitus with ophthalmic comps	25093002
771501000006119	Insulin-dependent diabetes mellitus with renal complications	127013003
772151000006116	Insulin dependent diabetes maturity onset	73211009
772161000006119	Insulin dependent diabetes mellitus	73211009
772171000006114	Insulin dependent diabetes mellitus	73211009
772181000006112	Insulin dependent diabetes mellitus - poor control	268519009

787101000006114	IDDM with peripheral circulatory disorder	421895002
787111000006112	IDDM-Insulin dependent diabetes mellitus	73211009
817471000006110	Haemoglobin A1c less than 7% indicating good diabetic control	165679005
817481000006113	Haemoglobin A1c greater than 10% indicating poor diabetic control	165681007
817491000006111	Haemoglobin A1c between 7%-10% indicating borderline diabetic control	165680008
840951000006119	Insulin treated Type 2 diabetes mellitus	237599002
841011000006112	Severe nonproliferative diabetic retinopathy	312905005
841351000006110	Insulin treated Type II diabetes mellitus	237599002
850691000006118	Hyperosmolar non-ketotic state in type 2 diabetes mellitus	395204000
856531000006119	Diabetic - GP eye check	856531000006103
856551000006114	Diabetic - hospital eye check	856551000006105
856611000006114	Laser treated diabetic retinopathy	856611000006105
856621000006118	Left proliferative diabetic retinopathy	856621000006102
856631000006115	Left laser treated diabetic retinopathy	856631000006104
856641000006113	Left diabetic maculopathy	856641000006109
856651000006110	Left advanced diabetic retinal disease	856651000006106
856661000006112	Left diabetic foot active ulceration	856661000006108
856671000006117	Left diabetic foot high risk	856671000006101
856681000006119	Left diabetic foot moderate risk	856681000006103
856691000006116	Left diabetic foot low current risk	856691000006100
856771000006110	Patient has been told has diabetes	856771000006106
857031000006113	Left non-proliferative diabetic retinopathy	857031000006109
857051000006118	Left preproliferative diabetic retinopathy	857051000006102
857411000006110	Right proliferative diabetic retinopathy	857411000006106
857421000006119	Right laser treated diabetic retinopathy	857421000006103
857431000006116	Right diabetic maculopathy	857431000006100
857441000006114	Right advanced diabetic retinal disease	857441000006105
857691000006119	Right diabetic foot active ulceration	857691000006103
857721000006112	Right diabetic foot high risk	857721000006108
857741000006117	Right diabetic foot moderate risk	857741000006101
857761000006118	Right diabetic foot low current risk	857761000006102
857971000006110	Right non-proliferative diabetic retinopathy	857971000006106
857981000006113	Right preproliferative diabetic retinopathy	857981000006109
881441000006111	Diabetes mellitus - juvenile	73211009
881451000006113	Diabetes mellitus -adult onset	532411000000102
881461000006110	Diabetes+ketoacidosis -no coma	420422005
881471000006115	Diabetes with coma	26298008
881481000006117	Diabetes + nephropathy	127013003
881491000006119	Diabetes + eye manifestation	25093002
881501000006110	Diabetes + neuropathy	230572002
881511000006113	Diabetes + periph.circulat.dis	422275004
881521000006117	Diabetes + other complications	658011000000104
905621000006113	[RFC] Diabetes	905621000006109
906711000006116	[RFC] Diabetic foot/leg ulcer	906711000006100
908831000006118	[RFC] Diabetes mellitus	908831000006102
910491000006117	[RFC] Diabetic leg ulcer	910491000006101
910501000006113	[RFC] Diabetic foot ulcer	910501000006109
913441000006119	Insulin-dependent diabetes mellitus with renal complications	127013003
913451000006117	Type 1 diabetes mellitus with renal complications	421893009
913461000006115	Type I diabetes mellitus with renal complications	421893009
913471000006110	Insulin-dependent diabetes mellitus with ophthalmic comps	25093002
913481000006113	Type 1 diabetes mellitus with ophthalmic complications	739681000
913491000006111	Type I diabetes mellitus with ophthalmic complications	739681000
913501000006115	Insulin-dependent diabetes mellitus with neurological comps	422088007
913511000006117	Type 1 diabetes mellitus with neurological complications	421468001
913521000006113	Neurological disorder with type 1 diabetes mellitus	421468001
913531000006111	Insulin dependent diabetes mellitus with multiple complicat	385041000000108
913541000006118	Type 1 diabetes mellitus with multiple complications	422228004
913551000006116	Type I diabetes mellitus with multiple complications	422228004
913591000006110	Skin ulcer associated with diabetes mellitus	422183001
913621000006112	Insulin dependent diabetes mellitus with gangrene	422275004
913651000006115	Insulin dependent diabetes mellitus with retinopathy	4855003
913661000006118	Retinopathy with type 1 diabetes mellitus	420789003
913671000006113	Type I diabetes mellitus with retinopathy	420789003
913681000006111	Insulin dependent diabetes mellitus - poor control	268519009
913711000006112	Insulin dependent diabetes maturity onset	73211009

913771000006115	Insulin dependent diabetes mellitus with mononeuropathy	230577008
913781000006117	Type 1 diabetes mellitus with mononeuropathy	420918009
913791000006119	Type I diabetes mellitus with mononeuropathy	420918009
913801000006118	Insulin dependent diabetes mellitus with polyneuropathy	49455004
913811000006115	Type 1 diabetes mellitus with polyneuropathy	713705003
913821000006111	Type I diabetes mellitus with polyneuropathy	713705003
913831000006114	Insulin dependent diabetes mellitus with nephropathy	127013003
913841000006116	Renal disorder associated with type 1 diabetes mellitus	421893009
913851000006119	Type I diabetes mellitus with nephropathy	421893009
913891000006113	Insulin dependent diabetes mellitus with diabetic cataract	43959009
913901000006112	Type 1 diabetes mellitus with diabetic cataract	421920002
913911000006110	Type I diabetes mellitus with diabetic cataract	421920002
913931000006116	Type 1 diabetes mellitus with peripheral angiopathy	31211000119101
913941000006114	Peripheral angiopathy due to type 1 diabetes mellitus	31211000119101
914031000006118	Renal disorder associated with type II diabetes mellitus	420279001
914041000006111	Type II diabetes mellitus with renal complications	420279001
914051000006113	Disorder of eye with type 2 diabetes mellitus	422099009
914061000006110	Type II diabetes mellitus with ophthalmic complications	422099009
914071000006115	Neurologic disorder associated with type 2 diabetes mellitus	421326000
914081000006117	Type II diabetes mellitus with neurological complications	421326000
914151000006112	Retinopathy with type 2 diabetes mellitus	422034002
914161000006114	Type II diabetes mellitus with retinopathy	422034002
914221000006113	Type 2 diabetes mellitus with mononeuropathy	420436000
914231000006111	Type II diabetes mellitus with mononeuropathy	420436000
914241000006118	Polyneuropathy due to type 2 diabetes mellitus	713706002
914251000006116	Type II diabetes mellitus with polyneuropathy	713706002
914271000006114	Type II diabetes mellitus with nephropathy	420279001
914301000006111	Type 2 diabetes mellitus with diabetic cataract	420756003
914311000006114	Type II diabetes mellitus with diabetic cataract	420756003
914391000006116	Insulin treated Type 2 diabetes mellitus	237599002
928461000006119	Persistent proteinuria associated with type 1 diabetes mellitus	420514000
928471000006114	Type I diabetes mellitus with persistent proteinuria	420514000
928491000006110	Type I diabetes mellitus with persistent microalbuminuria	401110002
928501000006119	Ketoacidosis in type 1 diabetes mellitus	420270002
928511000006116	Type I diabetes mellitus with ketoacidosis	420270002
928521000006112	Ketoacidotic coma in type 1 diabetes mellitus	421075007
928531000006110	Type I diabetes mellitus with ketoacidotic coma	421075007
928541000006117	Persistent proteinuria associated with type 2 diabetes mellitus	421986006
928551000006115	Type II diabetes mellitus with persistent proteinuria	421986006
928561000006118	Persistent microalbuminuria associated with type 2 diabetes mellitus	420715001
928571000006113	Type II diabetes mellitus with persistent microalbuminuria	420715001
928581000006111	Ketoacidosis in type 2 diabetes mellitus	421750000
928591000006114	Type II diabetes mellitus with ketoacidosis	421750000
928601000006118	Ketoacidotic coma in type 2 diabetes mellitus	421847006
928611000006115	Ketoacidotic coma in type II diabetes mellitus	421847006
932641000006116	Diabetes with coma	630521000000101
933231000006118	Hypo states in diabetes	658061000000102
938301000006114	Exudative maculopathy with type 1 diabetes mellitus	420486006
938311000006112	Type I diabetes mellitus with exudative maculopathy	420486006
938321000006116	Exudative maculopathy associated with type 2 diabetes mellitus	421779007
938331000006118	Exudative maculopathy with type 2 diabetes mellitus	421779007
967681000006119	Fibrocalculous pancreatic diabetes	2751001
967701000006116	Lipoatrophic diabetes mellitus	127012008
975251000006111	O/E - right eye stable treated proliferative diabetic retinopathy	414910007
975261000006113	O/E - left eye stable treated proliferative diabetic retinopathy	414894003
987441000006113	Six month diabetic review	987441000006109
1550571000000114	Jamaica type diabetes	75524006
1583891000006119	Ineligib for diab. eye screen: No longer categorised as diabetic	1583891000006103
1658861000000113	Type I diabetic dietary review	754101000000103
1658901000000118	Type II diabetic dietary review	754121000000107
1665751000000119	Type 1 diabetic dietary review	754101000000103
1666911000000113	O/E - Left diabetic foot at increased risk	756381000000100
1666931000000117	O/E - Right diabetic foot at increased risk	756391000000103
1667891000000113	Hyperosmolar non-ketotic state in type II diabetes mellitus	395204000
1667921000000117	Type II diabetes mellitus with gastroparesis	713703005
1667941000000112	Type I diabetes mellitus with gastroparesis	713702000

1694761000006113	Newly diagnosed diabetes	405749004
1696331000006114	Diabetic dermopathy	238982009
1704651000006111	Diabetic retinopathy screening administrative status	373031000000105
1734721000000114	Assessment of diabetic erectile dysfunction	473208009
1780981000006115	Diabetic on non-insulin injectable	1780981000006104
1804191000006114	Diabetic retinopathy screen done by optician	1804191000006105
1823921000006119	Diabetes mellitus confirmed	1823921000006103
1824931000006119	Home visit for diabetes monitoring	1824931000006103
1839671000006113	Diabetic short review	1839671000006109
1842001000006118	Diabetic annual review by practice nurse	1842001000006102
1850931000006111	Reason for influenza vaccine - diabetes mellitus	1850931000006107
1853141000006111	In-house diabetes foot screening	1853141000006107
1855351000006110	Conversion to insulin by diabetic specialist nurse	1855351000006106
1856641000006113	Diabetes monitoring in primary care	1856641000006109
1856651000006110	Diabetes monitoring in secondary care	1856651000006106
1856661000006112	Diabetes treatment type	1856661000006108
1873301000006114	Care contact service: diabetes	1873301000006105
1876341000006117	Diabetic 3 month review	1876341000006101
1884591000006118	Further diabetic monitoring	170742000
1932051000006114	3D study - problems with diabetes management	1932051000006105
1954041000006119	Diabetic - uses carbohydrate counting	1954041000006103
1960611000006113	Diabetic foot examination - action required	1960611000006109
1960621000006117	Diabetic foot examination - no action required	1960621000006101
1966311000006116	Diabetic on oral treatment and glucagon-like peptide 1 receptor agonist	976341000000101
1966321000006112	Diabetic on insulin and glucagon-like peptide 1 receptor agonist	976361000000100
1968641000006114	Maturity-onset diabetes of the young, type 5	609572000
2014951000006115	New diabetic foot ulcer	2014951000006104
2243331000000115	Erectile dysfunction due to diabetes mellitus	867891000000101
2249871000000113	In-house diabetic foot screening first appointment	870701000000106
2249951000000114	In-house diabetic foot screening follow-up appointment	870741000000109
2415191000000113	Diabetic peripheral neuropathic pain	944141000000108
2460251000000115	Diabetic on non-insulin injectable medication	719566006
2500921000000110	Diabetic carbohydrate counting diet	983451000000106
2576431000006117	DR - Diabetic retinopathy	4855003
2586691000000116	Under care of hospital-based diabetes specialist nurse	1024551000000109
2586731000000110	Under care of community-based diabetes specialist nurse	1024571000000100
2683581000006112	Labile diabetes	11530004
2683591000006110	Unstable diabetes mellitus	11530004
2867421000006114	Insulin dependent diabetes mellitus type 1A	23045005
2867431000006112	Insulin dependent diabetes mellitus type IA	23045005
2900951000006115	Diabetic eye disease	25093002
2906401000006113	Diabetic retinal microaneurysm	25412000
2920981000006117	Diabetic coma	26298008
3039941000006118	Pineal hyperplasia AND diabetes mellitus syndrome	33559001
3039971000006114	Pineal hyperplasia, insulin-resistant diabetes mellitus and somatic abnormalities	33559001
3050291000006112	Adult diabetes diet	34170007
3102571000006114	Diabetic urine	37398008
3209431000006116	Diabetes mellitus type II	44054006
3209441000006114	Diabetes mellitus type 2	44054006
3253231000006110	Diabetes mellitus type I	46635009
3253241000006117	Diabetes mellitus type 1	46635009
3319861000006118	Diabetic autonomic neuropathy	50620007
3459801000006118	Diabetes mellitus associated with hormonal aetiology	59079001
3511581000006114	Pretibial pigmental patches in diabetes	62260007
3649791000006119	Diabetes mellitus AND insipidus with optic atrophy AND deafness	70694009
3690681000006114	DM - Diabetes mellitus	73211009
4383621000006112	Diabetic mixed sensory-motor polyneuropathy	126534007
4392141000006116	Lipoatrophic diabetes	127012008
4392221000006118	Peripheral angiopathy due to diabetes mellitus	127014009
4636411000006110	Diet controlled diabetes mellitus	170745003
4636471000006118	Has seen dietitian - diabetes	170752001
4726201000006118	Diabetology domiciliary visit requested	183722003
4758011000006112	Type 2 diabetes mellitus with hyperosmolar coma	190331003
4758121000006115	Insulin-dependent diabetes maturity onset	190372001
4771421000006116	PPDR - Preproliferative diabetic retinopathy	193349004

4796321000006113	Pre-existing type 2 diabetes mellitus	199230006
4808881000006117	Diabetic hand syndrome	201723002
4808891000006119	Diabetic cheirarthropathy	201723002
4808921000006113	Diabetic neuropathic arthropathy	201724008
4929251000006115	Diabetic nurse	224543004
5009561000006114	Diabetic chronic painful polyneuropathy	230575000
5030691000006115	Proliferative diabetic retinopathy with new vessels on disc	232021008
5030741000006118	Proliferative diabetic retinopathy with new vessels elsewhere than on disc	232022001
5030771000006114	Diabetic traction retinal detachment	232023006
5095031000006114	Microalbuminuric diabetic nephropathy	236499007
5109561000006119	Maturity onset diabetes of the young, type 2	237604008
5109611000006116	MODY - Maturity onset diabetes in youth type 2	237604008
5109621000006112	MODY - Maturity onset diabetes glucokinase-related	237604008
5110071000006112	Hypoglycaemic event in diabetes	237632004
5110081000006110	Hypoglycemic event in diabetes	237632004
5110091000006113	Hypoglycaemic state in diabetes	237633009
5110101000006119	Hypoglycemic state in diabetes	237633009
5128751000006113	Diabetic thick skin syndrome	238983004
5128771000006115	Diabetic rubeosis	238984005
5192411000006114	Diabetic monitoring status	243860001
5505651000006113	Diabetes mellitus uncontrolled	268519009
5634901000006116	Diabetic foot	280137006
5933621000006118	Perceived control of insulin-dependent diabetes	304752002
5949691000006115	Referral by diabetic liaison nurse	306032006
5976641000006118	Admission to diabetic department	308253000
5979491000006119	Diabetes monitoring call	308505000
5987801000006113	Diabetologist	309350005
5988631000006117	Diabetes dietitian	309417009
5994181000006119	Diabetic department	309924002
5997431000006115	Diabetic liaison nurse	310185003
6022581000006116	Mild non-proliferative diabetic retinopathy	312903003
6022611000006112	Mild nonproliferative diabetic retinopathy	312903003
6022621000006116	Moderate nonproliferative diabetic retinopathy	312904009
6022671000006115	Proliferative diabetic retinopathy - non high risk	312906006
6022691000006119	Early proliferative diabetic retinopathy	312906006
6022721000006112	Proliferative diabetic retinopathy - high risk	312907002
6022761000006118	Proliferative diabetic retinopathy - quiescent	312908007
6022831000006119	Diabetic vitreous haemorrhage	312910009
6022841000006112	Diabetic vitreous hemorrhage	312910009
6022871000006116	Clinically significant diabetic macular oedema	312911008
6022921000006114	Diabetic macular oedema	312912001
6022931000006112	Diabetic macular edema	312912001
6030001000006116	Insulin-dependent diabetes without complication	313435000
6041331000006116	Diffuse diabetic maculopathy	314010006
6041341000006114	Focal diabetic maculopathy	314011005
6041411000006117	Ischaemic diabetic maculopathy	314014002
6041421000006113	Ischemic diabetic maculopathy	314014002
6041431000006111	Mixed diabetic maculopathy	314015001
6047101000006117	Diabetic optic papillopathy	314537004
6348911000006117	Diabetic foot ulcer	371087003
6444031000006114	Diabetic care assessment	385803003
6444051000006119	Diabetic care	385804009
6444101000006119	Diabetic care management	385806006
6515171000006112	Nonproliferative diabetic retinopathy	390834004
6515201000006111	NPDR - Non proliferative diabetic retinopathy	390834004
6515211000006114	BDR - Background diabetic retinopathy	390834004
6515451000006111	On examination - diabetic maculopathy present both eyes	390854003
6515471000006118	On examination - diabetic maculopathy absent both eyes	390855002
6546241000006111	On examination - Right diabetic foot at low risk	394671009
6546261000006110	On examination - Right diabetic foot at high risk	394672002
6546281000006117	On examination - Right diabetic foot - ulcerated	394673007
6546321000006111	On examination - Left diabetic foot at low risk	394675000
6546341000006116	On examination - Left diabetic foot at high risk	394676004
6546411000006113	On examination - Left diabetic foot at moderate risk	394681008
6546431000006119	On examination - Right diabetic foot at moderate risk	394682001
6612501000006110	Diabetic foot at risk	398819009

6630481000006114	Proliferative diabetic retinopathy - high risk with no macular oedema	399862001
6630561000006115	Diabetic macular oedema not clinically significant	399864000
6630571000006110	Diabetic macular edema not clinically significant	399864000
6630581000006113	Very severe proliferative diabetic retinopathy	399865004
6630591000006111	Diabetic retinal venous beading	399866003
6630631000006111	Diabetic intraretinal microvascular anomaly	399868002
6630641000006118	High risk proliferative diabetic retinopathy not amenable to photocoagulation	399869005
6630661000006119	Non-high-risk proliferative diabetic retinopathy with no macular oedema	399870006
6630701000006110	Visually threatening diabetic retinopathy	399871005
6630721000006117	Severe nonproliferative diabetic retinopathy with clinically significant macular oedema	399872003
6630751000006114	Severe nonproliferative diabetic retinopathy with no macular oedema	399873008
6630821000006112	Non-high-risk proliferative diabetic retinopathy with clinically significant macular oedema	399875001
6630851000006115	Very severe nonproliferative diabetic retinopathy	399876000
6670981000006118	Diabetic wet gangrene of the foot	402864004
6761781000006114	On examination - right eye background diabetic retinopathy	408409007
6761801000006113	On examination - left eye background diabetic retinopathy	408410002
6761821000006115	On examination - right eye preproliferative diabetic retinopathy	408411003
6761841000006110	On examination - left eye preproliferative diabetic retinopathy	408412005
6761861000006114	On examination - right eye proliferative diabetic retinopathy	408413000
6761881000006116	On examination - left eye proliferative diabetic retinopathy	408414006
6837961000006116	Panretinal photocoagulation for diabetes	413180006
6866541000006117	On examination - left eye stable treated proliferative diabetic retinopathy	414894003
6866781000006116	On examination - right chronic diabetic foot ulcer	414906009
6866901000006111	On examination - right eye stable treated proliferative diabetic retinopathy	414910007
6931861000006110	Infection of foot associated with diabetes	419100001
6953861000006110	Diabetic ketoacidosis	420422005
6953871000006115	Ketoacidosis in diabetes mellitus	420422005
6953891000006119	Diabetic acidosis	420422005
6953901000006115	DKA - diabetic ketoacidosis	420422005
6961081000006118	Gangrene associated with type 1 diabetes mellitus	420825003
6977421000006110	Ketoacidosis in type II diabetes mellitus	421750000
6985411000006117	Diabetic skin ulcer	422183001
7032031000006112	Diabetic peripheral neuropathy	424736006
7065531000006115	Latent autoimmune diabetes mellitus in adult (LADA)	426875007
7068831000006114	Diabetes mellitus due to cystic fibrosis	427089005
7081131000006113	Diabetic ophthalmoplegia	427943001
7281511000006118	Type 2 diabetes mellitus uncontrolled	443694000
7287611000006113	Type 1 diabetes mellitus uncontrolled	444073006
7287621000006117	Type I diabetes mellitus uncontrolled	444073006
7288191000006110	Type 2 diabetes mellitus well controlled	444110003
7299971000006116	Diabetes mellitus service	444913002
7305321000006114	Posttransplant diabetes mellitus	445260006
7483291000006111	Symptomatic diabetic peripheral neuropathy absent	473134007
7500191000006118	Maturity onset diabetes of the young, type 1	609562003
7500351000006110	Renal cysts and diabetes syndrome	609572000
7500501000006118	Diabetes mellitus, transient neonatal 1	609579009
7500521000006111	Diabetes mellitus, transient neonatal 2	609580007
7507871000006114	Diabetes self management plan	698360004
7567341000006113	Diabetes foot care clinic	702848001
7567371000006117	Diabetic retinopathy clinic	702850009
7613941000006113	Diabetic glomerulosclerosis	707221002
7700041000006112	Assessment of diabetic foot ulcer	713130008
7704351000006117	Neovascular glaucoma due to diabetes mellitus	713457002
7708051000006112	Gastroparesis due to diabetes mellitus type I	713702000
7708081000006116	Gastroparesis due to diabetes mellitus	713704004
7812121000006117	Diabetic retinopathy of eye not detected	721103006
7812131000006119	Diabetic retinopathy absent	721103006
7828041000006115	Diabetic retinal eye exam	722161008
7851731000006116	Diabetic mastopathy	724136006
7861571000006116	Radiculoplexoneuropathy due to diabetes mellitus	724810001
7861581000006118	Diabetic radiculoplexus neuropathy	724810001
7863801000006113	Lumbosacral plexopathy co-occurrent and due to diabetes mellitus	724997001
7863811000006111	Lumbosacral plexopathy with diabetes mellitus	724997001
7966561000006111	Mixed hyperlipidaemia due to type 2 diabetes mellitus	701000119103

7966611000006114	Chronic kidney disease stage 5 due to type 2 diabetes mellitus	711000119100
7966641000006113	Chronic kidney disease stage 4 due to type 2 diabetes mellitus	721000119107
7966691000006116	Chronic kidney disease stage 3 due to type 2 diabetes mellitus	731000119105
7966761000006115	Chronic kidney disease stage 2 due to type 2 diabetes mellitus	741000119101
7966791000006111	Chronic kidney disease stage 1 due to type 2 diabetes mellitus	751000119104
7966931000006114	Angina associated with type 2 diabetes mellitus	791000119109
7968751000006116	Diabetes mellitus type 2 without retinopathy	1481000119100
7968821000006118	Diabetic vitreous haemorrhage associated with type II diabetes mellitus	1491000119102
7968881000006119	Proliferative diabetic retinopathy due to type 2 diabetes mellitus	1501000119109
7968951000006118	Diabetic peripheral neuropathy associated with type II diabetes mellitus	1511000119107
7969001000006112	Foot ulcer due to type 2 diabetes mellitus	1521000119100
7969151000006110	Nonproliferative diabetic retinopathy due to type 2 diabetes mellitus	1551000119108
7969211000006115	Mixed hyperlipidaemia due to type 1 diabetes mellitus	1571000119104
7979721000006113	Diabetic foot exam not done	4551000175107
7979841000006113	Diabetic retinal eye exam not done	4581000175103
8006221000006115	Microalbuminuria due to type 1 diabetes mellitus	18521000119106
8012061000006117	Type 2 diabetic on insulin	24471000000103
8012071000006112	Type II diabetes on insulin	24471000000103
8012081000006110	Type 2 diabetic on diet only	24481000000101
8012091000006113	Type II diabetes on diet only	24481000000101
8014501000006115	Diabetes mellitus type 1 without retinopathy	31321000119102
8019521000006112	Nonproliferative diabetic retinopathy due to type 1 diabetes mellitus	60961000119107
8022481000006112	Peripheral neuropathy due to type 1 diabetes mellitus	71791000119104
8024771000006113	Rubeosis iridis due to type 1 diabetes mellitus	82581000119105
8026031000006115	Heel AND/OR midfoot ulcer due to type 2 diabetes mellitus	87451000119102
8026041000006113	Forefoot ulcer due to type 2 diabetes mellitus	87461000119100
8026061000006112	Heel AND/OR midfoot ulcer due to type 1 diabetes mellitus	87481000119109
8027391000006115	Chronic kidney disease stage 1 due to type 1 diabetes mellitus	90721000119101
8027411000006115	Chronic kidney disease stage 2 due to type 1 diabetes mellitus	90731000119103
8027451000006119	Chronic kidney disease stage 3 due to type 1 diabetes mellitus	90741000119107
8027471000006112	Chronic kidney disease stage 4 due to type 1 diabetes mellitus	90751000119109
8027491000006113	Chronic kidney disease stage 5 due to type 1 diabetes mellitus	90761000119106
8027521000006110	Microalbuminuria due to type 2 diabetes mellitus	90781000119102
8030601000006119	Macular oedema and retinopathy due to type 2 diabetes mellitus	97331000119101
8034861000006113	Leg ulcer due to type 2 diabetes mellitus	110171000119107
8035211000006110	Dyslipidemia with high density lipoprotein below reference range and triglyceride above reference range due to type 2 diabetes mellitus	111231000119109
8037841000006115	Hypoglycaemia due to type 2 diabetes mellitus	120731000119103
8043301000006116	Hyperlipidaemia due to type 2 diabetes mellitus	137931000119102
8043321000006114	Hyperlipidaemia due to type 1 diabetes mellitus	137941000119106
8043621000006117	Mild nonproliferative retinopathy due to type 2 diabetes mellitus	138911000119106
8044011000006117	Hypertension in chronic kidney disease stage 5 due to type 2 diabetes mellitus	140101000119109
8044061000006119	Hypertension in chronic kidney disease stage 3 due to type 2 diabetes mellitus	140121000119100
8044171000006110	Neuropathic toe ulcer due to type 2 diabetes mellitus	140381000119104
8044181000006113	Ulcer of toe due to type 2 diabetes mellitus	140391000119101
8044221000006116	Ischaemic foot ulcer due to type 2 diabetes mellitus	140521000119107
8048421000006113	Proteinuria due to type 2 diabetes mellitus	157141000119108
8064411000006119	Diabetic with adequate control	204061000000100
8077141000006113	Proteinuria due to type 1 diabetes mellitus	243421000119104
8119331000006114	Hyperglycaemia due to type 1 diabetes mellitus	367991000119101
8120381000006111	Neuropathy due to type 2 diabetes mellitus	368581000119106
8120401000006111	Hyperosmolar coma due to secondary diabetes mellitus	368601000119102
8120431000006115	Mild nonproliferative retinopathy due to secondary diabetes mellitus	368711000119106
8241041000006113	Symptomatic diabetic peripheral neuropathy	773001000000103
8242821000006115	No diabetic autonomic neuropathy	777601000000102
8259001000006114	Self management of diabetes	806961000000103
8267081000006115	Diabetes monitoring injection site not checked	819331000000104
8275381000006118	Endocrinology and diabetes	828901000000109
8440231000006115	Eating disorder co-occurrent with diabetes mellitus type 1	1067201000000106
11633211000006112	Atypical diabetes mellitus	530558861000132096
11633221000006116	ADM - atypical diabetes mellitus	530558861000132096
12078901000006118	Cataract of right eye due to diabetes mellitus	768792007
12078921000006112	Diabetic cataract of right eye	768792007
12078931000006114	Cataract of left eye due to diabetes mellitus	768793002
12078941000006116	Diabetic cataract of left eye	768793002
12078961000006116	Diabetic cataract of bilateral eyes	768794008

12078971000006112	Bilateral diabetic cataracts	768794008
12085021000006112	Preproliferative diabetic retinopathy of right eye	769181007
12085031000006112	Preproliferative retinopathy of right eye co-occurrent and due to diabetes mellitus	769181007
12085051000006116	Preproliferative diabetic retinopathy of left eye	769182000
12085071000006114	Mild nonproliferative diabetic retinopathy of right eye	769183005
12085091000006110	Mild non-proliferative diabetic retinopathy of right eye	769183005
12085121000006114	Mild nonproliferative diabetic retinopathy of left eye	769184004
12085141000006120	Mild non-proliferative diabetic retinopathy of left eye	769184004
12085171000006110	Moderate nonproliferative diabetic retinopathy of right eye	769185003
12085181000006112	Moderate non-proliferative diabetic retinopathy of right eye	769185003
12085211000006112	Moderate nonproliferative diabetic retinopathy of left eye	769186002
12085231000006118	Moderate non-proliferative diabetic retinopathy of left eye	769186002
12085241000006112	Moderate nonproliferative retinopathy of left eye due to diabetes mellitus	769186002
12085251000006112	Severe nonproliferative diabetic retinopathy of right eye	769187006
12085291000006120	Severe nonproliferative diabetic retinopathy of left eye	769188001
12085721000006110	Diabetic macular oedema of right eye	769217008
12085761000006116	Diabetic macular oedema of left eye	769218003
12085791000006112	Diabetic macular edema of left eye	769218003
12085801000006112	Macular oedema due to type 1 diabetes mellitus	769219006
12085841000006110	Macular oedema due to type 2 diabetes mellitus	769220000
12085891000006118	Clinically significant macular oedema of right eye co-occurrent and due to diabetes mellitus	769221001
12085921000006112	Clinically significant macular oedema of left eye due to diabetes mellitus	769222008
12086281000006112	Diabetic maculopathy of right eye	769244003
12086331000006116	Diabetic maculopathy of left eye	769245002
12086341000006114	Disorder of left macula co-occurrent and due to diabetes mellitus	769245002
12102571000006116	Clinically significant macular oedema with diabetes mellitus	770097006
12102611000006110	Clinically significant macular oedema due to diabetes mellitus	770097006
12117381000006110	Referral for diabetic care	1099151000000109
12119681000006110	Ketosis-prone diabetes mellitus	1102351000000105
12166111000006116	Retinopathy due to diabetes mellitus	4855003
12180791000006114	Diabetic lumbosacral plexopathy	39058009
12180901000006116	DRPN - diabetic radiculoplexus neuropathy	39181008
12185421000006112	Polyneuropathy due to diabetes mellitus	49455004
12189891000006110	PDR - proliferative diabetic retinopathy	59276001
12224731000006112	Moderate non-proliferative diabetic retinopathy	312904009
12224761000006116	Severe NPDR (nonproliferative diabetic retinopathy)	312905005
12224771000006110	Vitreous haemorrhage with diabetes mellitus	312910009
12224911000006116	Focal exudative diabetic maculopathy	314011005
12300391000006116	Macular oedema not clinically significant with diabetes mellitus	399864000
12363701000006116	Gastroparesis with diabetes mellitus	713704004
12370381000006112	Peripheral neuropathy with type 2 diabetes	1511000119107
12702381000006118	Diabetic oculopathy due to type I diabetes mellitus	739681000
12704401000006110	Diabetic oculopathy	25093002
12704411000006112	Peripheral circulatory disorder associated with diabetes mellitus	421895002
12704971000006112	Neurologic disorder associated with type 2 diabetes mellitus	421326000
12704991000006114	Ketoacidotic coma in type 2 diabetes mellitus	421847006
12705021000006112	Clinically significant macular oedema of right eye due to diabetes mellitus	769221001
12705071000006112	Gastroparesis due to type 1 diabetes mellitus	713702000
12705081000006112	Gastroparesis due to type 2 diabetes mellitus	713703005
12734741000006114	Registration for access to online diabetes self-management application	143911000000105
13488541000006116	Nonproliferative retinopathy of left eye due to diabetes mellitus	816177009
13488551000006116	Nonproliferative retinopathy of right eye due to diabetes mellitus	816178004
13767911000006120	Disorder of eye due to diabetes mellitus	25093002
13790061000006120	Neuropathic ulcer of foot due to diabetes mellitus	201251005
13790071000006114	Neuropathic arthropathy due to diabetes mellitus	201724008
13791991000006116	Proteinuric nephropathy due to diabetes mellitus	236500003
13810021000006112	Glomerulopathy due to diabetes mellitus	309426007
13810171000006112	Vitreous haemorrhage due to diabetes mellitus	312910009
13810181000006112	Macular oedema due to diabetes mellitus	312912001
13832391000006118	Infection of foot due to diabetes mellitus	419100001
13833601000006112	Hyperosmolar coma due to diabetes mellitus	422126006
13917091000006116	Neuropathic ulcer of heel due to type 2 diabetes mellitus	789569005
13949611000006116	No maculopathy of left eye due to diabetes mellitus	860709007
13949621000006112	No diabetic maculopathy of left eye	860709007

13949641000006116	No maculopathy of right eye due to diabetes mellitus	860710002
13949651000006116	No diabetic maculopathy of right eye	860710002
13949671000006112	No diabetic maculopathy of bilateral eyes	860711003
13949711000006112	Diabetic maculopathy of bilateral eyes present	860712005
13953211000006120	Ulcer of left foot due to diabetes mellitus	860978005
13953411000006118	At low risk of ulcer of right foot due to diabetes mellitus	863879002
13953421000006114	At low risk of ulcer of left foot due to diabetes mellitus	863880004
13965721000006114	Severe nonproliferative diabetic retinopathy with venous beading	870420005
13966631000006112	Newly diagnosed type 1 diabetes mellitus	870528001
13966641000006116	Newly diagnosed diabetes mellitus type 1	870528001
14015311000006110	Coronary artery disease due to type 2 diabetes mellitus	16891151000119104
14056961000006112	Ketoacidotic coma due to diabetes mellitus	26298008
14057771000006116	Lumbosacral radiculoplexus neuropathy due to diabetes mellitus	39058009
14057821000006112	Radiculoplexus neuropathy due to diabetes mellitus	39181008
14058781000006116	Autonomic neuropathy due to diabetes mellitus	50620007
14058811000006116	Pancreatogenic type 3c diabetes mellitus	51002006
14059421000006120	Proliferative retinopathy due to diabetes mellitus	59276001
14060651000006114	Complication due to diabetes mellitus	74627003
14067581000006118	Disorder of kidney due to diabetes mellitus	127013003
14070771000006116	Loss of hypoglycaemic warning due to diabetes mellitus	170766006
14071211000006112	Multiple complications due to type 2 diabetes mellitus	190388001
14071331000006110	Chronic painful neuropathy due to diabetes mellitus	193184006
14071341000006116	Asymptomatic neuropathy due to diabetes mellitus	193185007
14071361000006118	Preproliferative retinopathy due to diabetes mellitus	193349004
14071371000006112	Advanced maculopathy due to diabetes mellitus	193350004
14071441000006112	Cellulitis of foot due to diabetes mellitus	200687002
14071551000006112	Ischaemic ulcer of foot due to diabetes mellitus	201250006
14072461000006116	Neuropathy due to diabetes mellitus	230572002
14072481000006114	Chronic painful polyneuropathy due to diabetes mellitus	230575000
14072591000006112	Disorder of macula due to diabetes mellitus	232020009
14072601000006116	Proliferative retinopathy with optic disc neovascularisation due to diabetes mellitus	232021008
14072621000006112	Traction detachment of retina due to diabetes mellitus	232023006
14073061000006116	Abnormal metabolic state due to diabetes mellitus	237620003
14073071000006112	Severe hyperglycaemia due to diabetes mellitus	237621004
14073081000006114	Hypoglycaemic event due to diabetes	237632004
14073091000006112	Hypoglycaemia due to diabetes mellitus	237633009
14073101000006118	Nocturnal hypoglycaemia due to diabetes mellitus	237635002
14077381000006116	Hyperosmolar non-ketotic state due to diabetes mellitus	310505005
14077471000006114	Advanced retinal disease due to diabetes mellitus	311782002
14077691000006112	Mild nonproliferative retinopathy due to diabetes mellitus	312903003
14077701000006112	Moderate nonproliferative retinopathy due to diabetes mellitus	312904009
14077711000006112	Severe nonproliferative retinopathy due to diabetes mellitus	312905005
14077721000006116	Non-high-risk proliferative retinopathy due to diabetes mellitus	312906006
14077731000006116	High risk proliferative retinopathy due to diabetes mellitus	312907002
14077751000006112	Proliferative retinopathy with iris neovascularisation due to diabetes mellitus	312909004
14077861000006116	Focal exudative maculopathy due to diabetes mellitus	314011005
14077871000006112	Ischaemic maculopathy due to diabetes mellitus	314014002
14079681000006116	Nonproliferative retinopathy due to diabetes mellitus	390834004
14079871000006120	Hyperosmolar non-ketotic state due to type 2 diabetes mellitus	395204000
14083741000006114	Ketoacidosis due to type 1 diabetes mellitus	420270002
14083771000006118	Exudative maculopathy due to type 1 diabetes mellitus	420486006
14083781000006116	Persistent proteinuria due to type 1 diabetes mellitus	420514000
14083791000006116	Coma due to diabetes mellitus	420662003
14083811000006118	Persistent microalbuminuria due to type 2 diabetes mellitus	420715001
14083831000006112	Retinopathy due to type 1 diabetes mellitus	420789003
14083841000006120	Gangrene due to type 1 diabetes mellitus	420825003
14083901000006110	Persistent microalbuminuria due to type 1 diabetes mellitus	421305000
14083911000006112	Disorder of nervous system due to type 2 diabetes mellitus	421326000
14083931000006120	Hypoglycaemic coma due to type 1 diabetes mellitus	421437000
14083941000006112	Disorder of nervous system due to type 1 diabetes mellitus	421468001
14083961000006112	Gangrene due to type 2 diabetes mellitus	421631007
14083991000006116	Ketoacidosis due to type 2 diabetes mellitus	421750000
14084001000006112	Exudative maculopathy due to type 2 diabetes mellitus	421779007
14084021000006120	Renal disorder due to type 1 diabetes mellitus	421893009
14084031000006116	Non-ketotic non-hyperosmolar coma due to diabetes mellitus	421966007

14084041000006114	Persistent proteinuria due to type 2 diabetes mellitus	421986006
14084061000006112	Retinopathy due to type 2 diabetes mellitus	422034002
14084091000006116	Disorder of nervous system due to diabetes mellitus	422088007
14084101000006112	Disorder of eye due to type 2 diabetes mellitus	422099009
14084111000006114	Peripheral circulatory disorder due to type 2 diabetes mellitus	422166005
14084121000006118	Skin ulcer due to diabetes mellitus	422183001
14085781000006120	Macroalbuminuric nephropathy due to diabetes mellitus	445170001
14090011000006118	Hypoglycaemic coma due to type 2 diabetes mellitus	719216001
14091771000006116	Disorder of eye due to type 1 diabetes mellitus	739681000
14092431000006120	Disorder of right macula due to diabetes mellitus	769244003
14092441000006112	Disorder of left macula due to diabetes mellitus	769245002
14092491000006116	Erectile dysfunction due to diabetes mellitus	770096002
14094571000006112	Neuropathic arthropathy due to type 2 diabetes mellitus	781000119106
14095251000006116	Neuropathic ulcer of toe due to type 2 diabetes mellitus	140381000119104
14095271000006114	Neuropathic ulcer of foot due to type 2 diabetes mellitus	140531000119105
14136361000006116	Ketosis-prone diabetes mellitus	890171006
14181911000006116	Bilateral nonproliferative retinopathy due to diabetes mellitus type 2	16747661000119108
14182011000006110	Bilateral proliferative retinopathy of eyes due to diabetes mellitus type 1	16749781000119100
14500411000006110	Haemoglobin A1c less than 7 percent indicating good diabetic control	165679005
14500421000006120	Haemoglobin A1c between 7 percent to 10 percent indicating borderline diabetic control	165680008
14500431000006116	Haemoglobin A1c greater than 10 percent indicating poor diabetic control	165681007
14728721000006116	Neovascular glaucoma due to diabetes mellitus type 2	1196922005
14765381000006118	Diabetic neurotrophic keratitis	1217674007

Hospital episode statistics: Diabetes	
**icd**	**description**
E10	Type 1 diabetes mellitus
E11	Type 2 diabetes mellitus
E12	Malnutrition-related diabetes mellitus
E13	Other specified diabetes mellitus
E14	Unspecified diabetes mellitus
H36.0	Diabetic retinopathy

Clinical codes for deep vein thrombosis / pulmonary embolism

GOLD: clinical, referral, tests; Aurum: consultations, observations.

Additional sources of information in addition to clinical opinion and in-house code repository: Ruigómez et al. 2020: https://www.ncbi.nlm.nih.gov/pmc/articles/PMC7821274/)

CPRD GOLD: Deep vein thrombosis/pulmonary embolism		
**medcode**	**readcode**	**readterm**
428	G82z100	Thrombosis of vein NOS
431	G80z000	Phlebitis NOS
824	G801.11	Deep vein thrombosis
1224	SP12200	Post operative deep vein thrombosis
1264	G80z100	Thrombophlebitis NOS
1266	G401.00	Pulmonary embolism
2198	G82z011	Embolism of vein NOS
2199	G82zz00	Embolism and thrombosis NOS
3392	G801.13	DVT - Deep vein thrombosis
3466	G802.00	Phlebitis and thrombophlebitis of the leg NOS
3576	G801.00	Deep vein phlebitis and thrombophlebitis of the leg
4607	L414.00	Postnatal deep vein thrombosis
4717	G402.00	Pulmonary infarct
5503	G823.00	Embolism and thrombosis of the renal vein
5822	G80..00	Phlebitis and thrombophlebitis
7174	L43..00	Obstetric pulmonary embolism
9255	G802000	Thrombosis of vein of leg
9701	G401.12	Pulmonary embolus
11031	G800500	Thrombophlebitis of a superficial leg vein NOS
14657	G80yz00	Other phlebitis and thrombophlebitis NOS
14742	G80z.00	Phlebitis and thrombophlebitis NOS
15382	G801600	Thrombophlebitis of the femoral vein
16250	L414.12	Phlegmasia alba dolens - obstetric
16446	G821.00	Thrombophlebitis migrans

17452	G800.12	Saphenous vein thrombophlebitis
18121	G401000	Post operative pulmonary embolus
18830	L414.11	DVT - deep venous thrombosis, postnatal
20676	G820.00	Budd - Chiari syndrome (hepatic vein thrombosis)
22038	G801D00	Deep vein thrombosis of lower limb
23588	L414200	Postnatal deep vein thrombosis with postnatal complication
23667	L413.00	Antenatal deep vein thrombosis
23765	G82z111	Penis vein thrombosis
24444	G401.11	Infarction - pulmonary
24737	G800300	Thrombophlebitis of the long saphenous vein
25478	G801.12	Deep vein thrombosis, leg
26650	G82..00	Other venous embolism and thrombosis
26873	L413.11	DVT - deep venous thrombosis, antenatal
27284	G801z00	Deep vein phlebitis and thrombophlebitis of the leg NOS
27688	G82z.00	Embolism and thrombosis NOS
28404	G800z00	Superficial phlebitis and thrombophlebitis of the leg NOS
31313	L430.00	Obstetric air pulmonary embolism
32002	G801B00	Deep vein thrombophlebitis of the leg unspecified
35297	G82y.00	Other embolism and thrombosis
37849	G82z000	Embolus of vein NOS
38527	G800400	Thrombophlebitis of the short saphenous vein
42158	G801500	Deep vein phlebitis of the leg unspecified
42435	G80y.00	Other phlebitis and thrombophlebitis
42506	G801C00	Deep vein thrombosis of leg related to air travel
43555	G822.00	Embolism and thrombosis of the vena cava
44100	G80zz00	Phlebitis and thrombophlebitis NOS
44192	L431.00	Amniotic fluid pulmonary embolism
44404	L43z400	Obstetric pulmonary embolism NOS with postnatal complication
48920	G801E00	Deep vein thrombosis of leg related to intravenous drug use
49269	L432.00	Obstetric blood-clot pulmonary embolism
55661	G801700	Thrombophlebitis of the popliteal vein
57100	G820.11	Hepatic vein thrombosis
58023	G80y600	Thrombophlebitis of the external iliac vein
58166	G80y400	Thrombophlebitis of the common iliac vein
59059	L433.12	Septic obstetric embolism
61203	L413z00	Antenatal deep vein thrombosis NOS
61204	L414z00	Postnatal deep vein thrombosis NOS
61760	G801A00	Thrombophlebitis of the posterior tibial vein
65725	L413000	Antenatal deep vein thrombosis unspecified
66817	L431200	Amniotic fluid pulm embolism - delivered + p/n complication
67006	L096400	Pulmonary embolism following abortive pregnancy
68438	L43..11	Obstetric pulmonary embolus
69921	L414000	Postnatal deep vein thrombosis unspecified
70467	G80y800	Phlebitis and thrombophlebitis of the iliac vein NOS
73569	L43z.00	Obstetric pulmonary embolism NOS
73624	L431100	Amniotic fluid pulmonary embolism - delivered
91282	G80y700	Thrombophlebitis of the iliac vein unspecified
94405	L413100	Antenatal deep vein thrombosis - delivered
94496	G80y500	Thrombophlebitis of the internal iliac vein
95077	G801800	Thrombophlebitis of the anterior tibial vein
96209	G401100	Recurrent pulmonary embolism
97367	L43z100	Obstetric pulmonary embolism NOS - delivered
97808	G822000	Thrombosis of inferior vena cava
98526	G801F00	Deep vein thrombosis of peroneal vein
98639	L43zz00	Obstetric pulmonary embolism NOS
100103	G824.00	Axillary vein thrombosis
100502	L413200	Antenatal deep vein thrombosis with antenatal complication
101944	L43z000	Obstetric pulmonary embolism NOS, unspecified
104342	G801G00	Recurrent deep vein thrombosis
105403	G825.00	Thrombosis of subclavian vein
106482	G801900	Thrombophlebitis of the dorsalis pedis vein
106850	G826.00	Thrombosis of internal jugular vein
107851	G827.00	Thrombosis of external jugular vein
109337	L43yz00	Other obstetric pulmonary embolism NOS
110449	G801J00	Provoked deep vein thrombosis
110542	G801H00	Unprovoked deep vein thrombosis
112358	L414100	Postnatal deep vein thrombosis - delivered with p/n comp
112578	L432300	Obstetric blood-clot pulmonary embolism + a/n complication

112710	L432z00	Obstetric blood-clot pulmonary embolism NOS
113599	L432000	Obstetric blood-clot pulmonary embolism unspecified
113630	L432200	Obstetric blood-clot pulm embolism - delivered with p/n comp
114089	L43y.00	Other obstetric pulmonary embolism
115030	L431400	Amniotic fluid pulmonary embolism with p/n complication
115701	L432400	Obstetric blood-clot pulmonary embolism + p/n complication
115728	L432100	Obstetric blood-clot pulmonary embolism - delivered

CPRD Aurum: Deep vein thrombosis/pulmonary embolism		
**medcode**	**term**	**snomedconceptID**
52299017	Thrombophlebitis migrans	31268005
64466019	Hepatic vein thrombosis	82385007
98484016	Pulmonary embolism	59282003
216205019	Deep vein thrombosis of leg related to air travel	134399007
300649013	Phlebitis and thrombophlebitis	195394007
300659014	Thrombophlebitis of a superficial leg vein NOS	40283005
300660016	Superficial phlebitis and thrombophlebitis of the leg NOS	40283005
300672010	Thrombophlebitis of the femoral vein	195410000
300673017	Thrombophlebitis of the popliteal vein	195411001
300674011	Thrombophlebitis of the anterior tibial vein	195412008
300675012	Thrombophlebitis of the dorsalis pedis vein	770111009
300676013	Thrombophlebitis of the posterior tibial vein	195414009
300677016	Thrombophlebitis of deep veins of lower extremity	40198004
300681016	Deep vein phlebitis and thrombophlebitis of the leg NOS	266267005
300682011	Phlebitis and thrombophlebitis of the leg NOS	266267005
300691010	Thrombophlebitis of the common iliac vein	195425000
300692015	Thrombophlebitis of the internal iliac vein	195426004
300695018	Thrombophlebitis of the external iliac vein	195427008
300696017	Thrombophlebitis of iliac vein	42861008
300697014	Phlebitis and thrombophlebitis of the iliac vein NOS	276494008
300698016	Other phlebitis and thrombophlebitis NOS	195394007
300699012	Phlebitis and thrombophlebitis NOS	195394007
300700013	Phlebitis	61599003
300701012	Thrombophlebitis	64156001
300704016	Thromboembolism of vein	429098002
300710016	Embolism and thrombosis of the vena cava	195437003
300711017	Embolism and thrombosis of the renal vein	195438008
300712012	Other embolism and thrombosis	429098002
300713019	Embolism and thrombosis NOS	429098002
300714013	Embolus of vein NOS	234049002
300975010	Venous embolism	234049002
305585018	Pulmonary embolism following abortive pregnancy	609480009
307739018	Antenatal deep vein thrombosis unspecified	49956009
307740016	Antenatal deep vein thrombosis - delivered	200232006
307741017	Antenatal deep vein thrombosis with antenatal complication	200233001
307742012	Antenatal deep vein thrombosis NOS	49956009
307747018	Postnatal deep vein thrombosis unspecified	56272000
307749015	Postnatal deep vein thrombosis with postnatal complication	200238005
307750015	Postnatal deep vein thrombosis NOS	56272000
307815016	Obstetric pulmonary embolism	200284000
307816015	Obstetric pulmonary embolus	200284000
307832017	Obstetric blood-clot pulmonary embolism	200299000
307833010	Obstetric blood-clot pulmonary embolism unspecified	200299000
307834016	Obstetric blood-clot pulmonary embolism - delivered	200301007
307838018	Obstetric blood-clot pulmonary embolism NOS	200299000
350662018	Saphenous vein thrombophlebitis	234040003
395801015	Deep vein phlebitis and thrombophlebitis of the leg	266267005
395802010	Other phlebitis and thrombophlebitis	195394007
395803017	Venous thrombosis	111293003
418852012	Phlegmasia alba dolens - obstetric	280966008
419640012	Thrombosis of inferior vena cava	281595001
437622013	Axillary vein thrombosis	297156001
453210011	Thrombosis of vein of leg	309735004
495552014	Antenatal deep vein thrombosis	49956009
497563011	Postnatal deep vein thrombosis	56272000
497564017	DVT - deep venous thrombosis, postnatal	56272000

499917019	Pulmonary infarct	64662007
503317010	Penis vein thrombosis	76598006
1222321017	Embolism of vein NOS	234049002
2162148012	Deep vein thrombosis	128053003
2162149016	Deep venous thrombosis	128053003
2162422011	Deep vein thrombosis of lower limb	404223003
2534187010	Deep vein thrombosis of leg related to intravenous drug use	413956008
2795114019	Thrombosis of subclavian vein	438647008
100611000006111	Superficial thrombophlebitis of long saphenous vein	195396009
100641000006110	Superficial thrombophlebitis of short saphenous vein	195397000
149011000006118	Septic obstetric embolism	267284008
193601000006115	Pulmonary embolus	59282003
211691000006114	Postnatal deep vein thrombosis - delivered with postnatal complication	200237000
216571000006116	Postoperative deep vein thrombosis	213220000
216591000006115	Postoperative pulmonary embolus	194883006
218551000000118	Deep vein thrombosis, leg	266267005
231301000006116	Phlebitis and thrombophlebitis NOS	195394007
269411000006115	Obstetric blood-clot pulmonary embolism - delivered with postnatal complication	200302000
269431000006114	Obstetric blood-clot pulmonary embolism with antenatal complication	200303005
269441000006116	Obstetric blood-clot pulmonary embolism with postnatal complication	200304004
527221000006115	Budd-Chiari syndrome	82385007
632111000000118	Recurrent pulmonary embolism	438773007
632731000006118	Antenatal DVT	49956009
638821000006113	Embolism and thrombosis NOS	429098002
728841000006111	Pulmonary infarction	64662007
851241000006115	Axillary vein thrombosis	851241000006104
884741000006117	Venous embolism NOS	587851000000108
1119161000000115	Deep vein thrombosis of peroneal vein	443210003
1786921000006116	Recurrent deep vein thrombosis	1786921000006100
2144741000000111	Recurrent deep vein thrombosis	710167004
2234371000000119	Thrombosis of internal jugular vein	864191000000104
2234411000000115	Thrombosis of external jugular vein	864211000000100
2488341000000112	Unprovoked deep vein thrombosis	978421000000101
2488381000000116	Provoked deep vein thrombosis	978441000000108
2784341000006116	Deep vein thrombosis of portal vein	17920008
3123551000006116	Hepatic vein thrombosis	38739001
3308951000006115	Antepartum deep vein thrombosis	49956009
3414351000006118	Postpartum deep vein thrombosis	56272000
3462921000006119	PE - Pulmonary embolism	59282003
4403261000006113	DVT - Deep vein thrombosis	128053003
5057621000006119	Acute massive pulmonary embolism	233936003
5057651000006111	Subacute massive pulmonary embolism	233937007
5057661000006113	SAMPE - Subacute massive pulmonary embolism	233937007
5059301000006112	Iliofemoral deep vein thrombosis	234044007
5059321000006119	Ileofemoral deep vein thrombosis	234044007
6452821000006114	Pulmonary embolus care	386280004
6694751000006113	DVT - Deep vein thrombosis of lower limb	404223003
7567311000006114	Deep vein thrombosis clinic	702845003
7609581000006110	Acute pulmonary embolism	706870000
7699481000006113	Pulmonary embolism on long-term anticoagulation therapy	713078005
7848521000006117	Pulmonary embolism as current complication following acute myocardial infarction	723859005
7966351000006118	Acute deep vein thrombosis of lower limb	651000119108
7967281000006119	Pulmonary embolism with pulmonary infarction	1001000119102
7967291000006116	Pulmonary embolism with infarction	1001000119102
8042101000006112	Chronic pulmonary embolism	133971000119108
8361011000006114	Unprovoked DVT (deep vein thrombosis)	978421000000101
8361031000006115	Provoked DVT (deep vein thrombosis)	978441000000108
12107671000006116	Chronic deep vein thrombosis of left iliac vein	293461000119100
12107681000006116	Acute deep vein thrombosis of right iliac vein	293481000119109
12107691000006120	Acute deep vein thrombosis of left iliac vein	293491000119107
13949481000006112	Deep vein thrombosis of lower extremity due to intravenous drug use	860699005
14194181000006112	Acute DVT (deep vein thrombosis) of left peroneal vein	46557031000119104
14194871000006116	Acute DVT (deep vein thrombosis) of right peroneal vein	843433951000119040

Hospital episode statistics: Deep vein thrombosis/pulmonary embolism	
icd	description
I26	Pulmonary embolism
I80	Phlebitis and thrombophlebitis
I82	Other venous embolism and thrombosis
E13	Other specified diabetes mellitus
E14	Unspecified diabetes mellitus
O22.3	Deep phlebothrombosis in pregnancy
O87.1	Deep phlebothrombosis in the puerperium

Clinical codes for dysphagia

GOLD: clinical, referral, tests; Aurum: consultations, observations.

Additional sources of information in addition to clinical opinion and in-house code repository: Primary Care Domain Reference Set Portal: https://digital.nhs.uk/data-and-information/data-collections-and-data-sets/data-collections/quality-and-outcomes-framework-qof/quality-and-outcome-framework-qof-business-rules/primary-care-domain-reference-set-portal

CPRD GOLD: Dysphagia		
medcode	readcode	readterm
727	R072000	[D]Difficulty in swallowing
1241	194..11	Dysphagia
4281	R072.00	[D]Dysphagia
6300	1942.00	Difficulty swallowing solids
16693	ZV41612	[V]Problems with swallowing
18878	1944.00	Painful swallowing
20123	D00y.12	Plummer - Vinson syndrome
26042	1943.00	Difficulty swallowing liquids
30955	R072z00	[D]Dysphagia NOS
31347	D00y.11	Kelly - Paterson syndrome
35199	D00y012	Plummer-Vinson syndrome
36965	D00y011	Kelly-Paterson syndrome
41885	1944.11	Odynophagia
47814	P76E.00	Aber retro-oesophag subclavian artery causing dysphag lusori
55478	D00y000	Sideropenic dysphagia
108730	1945.00	Developmental dysphagia
110485	1946.00	Chokes when swallowing

CPRD Aurum: Dysphagia		
medcode	term	snomedconceptID
50595018	Odynophagia	30233002
50596017	Swallowing painful	30233002
67950018	Dysphagia	40739000
132937017	Plummer-Vinson syndrome	80126007
132939019	Sideropenic dysphagia	80126007
252548011	Difficulty swallowing solids	162024005
252550015	Difficulty swallowing liquids	162025006
317467017	[D]Dysphagia	40739000
317468010	Difficulty in swallowing	40739000
317469019	[D]Dysphagia NOS	40739000
339003012	Chokes when swallowing	225589000
1227659013	[V]Problems with swallowing	399122003
3506623013	Dysphagia due to and following non-traumatic intracerebral haemorrhage	290641000119107
219431000006113	Plummer - Vinson syndrome	80126007
281961000006111	Kelly - Paterson syndrome	80126007
295621000000119	Developmental dysphagia	196871000000106
437081000006119	Aberrant retro-oesophageal subclavian artery causing dysphagia lusoria	311808009
755101000006110	Kelly-Paterson syndrome	80126007
982661000006112	Dysphagia	982661000006108
2642911000000117	O/E - swallowing abnormality	1049221000000102
2811061000006117	Intermittent dysphagia	19597002
2986821000006119	Painful swallowing	30233002
2986831000006116	Pain on swallowing	30233002

3155971000006116	Swallowing difficult	40739000
3158501000006114	Oesophageal dysphagia	40890009
3661001000006117	Cricopharyngeal dysphagia	71363005
3662621000006114	Oropharyngeal dysphagia	71457002
3803181000006117	Paterson-Kelly syndrome	80126007
3803201000006116	Brown-Kelly-Paterson syndrome	80126007
3803211000006118	Plummer-Vinson-Patterson-Kelly syndrome	80126007
4545991000006110	Difficulty swallowing fluid	162025006
4943671000006110	Choking during swallowing	225589000
5272341000006114	Food sticks on swallowing	249485007
5272361000006113	Unable to swallow	249486008
5272371000006118	Unable to initiate swallowing	249487004
5739301000006114	Difficulty swallowing	288939007
5739331000006118	Unable to swallow food	288942001
5739371000006115	Difficulty swallowing food	288945004
5739421000006110	Unable to swallow fluid	288948002
5739591000006111	Unable to swallow saliva	288959006
5739631000006111	Difficulty swallowing saliva	288962009
5960401000006112	Unable to swallow solids	306770001
5960461000006113	Unable to swallow soft foods	306775006
5960501000006113	Difficulty swallowing soft foods	306778008
6009721000006110	Aberrant retro-esophageal subclavian artery causing dysphagia	311808009
6617701000006118	Swallowing problem	399122003
6617711000006115	Impaired swallowing	399122003
6617721000006111	Swallowing impairment	399122003
7112321000006112	Oral phase dysphagia	429975007
8008641000006112	Pharyngeal dysphagia	21101000119105
8056801000006119	Acquired dysphagia	196841000000100
8306391000006113	Unable to swallow tablets	877931000000103
8317511000006110	Acquired swallowing difficulty	898751000000102
12223741000006116	Food sticks on swallowing	288945004
14075931000006112	Difficulty in swallowing	288939007

Hospital episode statistics: Dysphagia	
**icd**	**description**
R13	Dysphagia

Clinical codes for epilepsy

GOLD: clinical, referral, tests; Aurum: consultations, observations.

Some process codes taken out but monitoring retained as commonly used among people with epilepsy; sensory and stress-induced epilepsy was retained, but alcohol and drug-induced epilepsy was removed. Individual seizures and status epilepticus removed. Epileptic dementia was included in dementia but not here.

CPRD GOLD: Epilepsy		
**medcode**	**readcode**	**readterm**
573	F25..00	Epilepsy
988	F251000	Grand mal (major) epilepsy
1715	F250011	Epileptic absences
2907	F250000	Petit mal (minor) epilepsy
3175	F254000	Temporal lobe epilepsy
3607	F25z.11	Fit (in known epileptic) NOS
4093	F253.11	Status epilepticus
4109	SC20000	Traumatic epilepsy
4602	667B.00	Nocturnal epilepsy
4801	F251300	Epileptic seizures - myoclonic
5117	F253.00	Grand mal status
5152	F251400	Epileptic seizures - tonic
5525	F255011	Focal epilepsy
5668	F251600	Grand mal seizure
6271	F25X.00	Status epilepticus, unspecified
6709	Eu05y11	[X]Epileptic psychosis NOS
6983	667..00	Epilepsy monitoring
7945	F256000	Hypsarrhythmia

8187	F251500	Tonic-clonic epilepsy
9569	F255000	Jacksonian, focal or motor epilepsy
9747	F25z.00	Epilepsy NOS
9886	F252.00	Petit mal status
9887	F25y200	Locl-rlt(foc)(part)idiop epilep&epilptic syn seiz locl onset
9979	F25yz00	Other forms of epilepsy NOS
11186	F250.00	Generalised nonconvulsive epilepsy
11394	F254500	Complex partial epileptic seizure
13220	667D.00	Epilepsy control poor
13221	667R.00	2 to 4 seizures a month
18471	F251200	Epileptic seizures - clonic
18899	667T.00	Daily seizures
19549	667S.00	1 to 7 seizures a week
19550	667C.00	Epilepsy control good
21885	F258.00	Post-ictal state
22341	1O30.00	Epilepsy confirmed
22804	F251011	Tonic-clonic epilepsy
22991	667N.00	Epilepsy severity
23415	F256100	Salaam attacks
23634	F254100	Psychomotor epilepsy
24309	F250200	Epileptic seizures - atonic
25330	F25y300	Complex partial status epilepticus
26015	F255.00	Partial epilepsy without impairment of consciousness
26144	F251.00	Generalised convulsive epilepsy
26733	F255y00	Partial epilepsy without impairment of consciousness OS
27526	F255z00	Partial epilepsy without impairment of consciousness NOS
30635	F25F.00	Photosensitive epilepsy
31830	F250300	Epileptic seizures - akinetic
31877	Eu05212	[X]Schizophrenia-like psychosis in epilepsy
31920	F254z00	Partial epilepsy with impairment of consciousness NOS
32288	F254.00	Partial epilepsy with impairment of consciousness
34079	F254400	Epileptic automatism
34792	F250500	Lennox-Gastaut syndrome
36203	F254200	Psychosensory epilepsy
36696	667Z.00	Epilepsy monitoring NOS
37592	F255200	Somatosensory epilepsy
37644	F132100	Progressive myoclonic epilepsy
37782	F251100	Neonatal myoclonic epilepsy
37906	F259.00	Early infant epileptic encephalopathy wth suppression bursts
38307	F25y.00	Other forms of epilepsy
38919	1B1W.00	Transient epileptic amnesia
39023	F256.12	West syndrome
39160	667V.00	Many seizures a day
40105	F255600	Simple partial epileptic seizure
40806	F251z00	Generalised convulsive epilepsy NOS
43679	Eu80300	[X]Acquired aphasia with epilepsy [Landau - Kleffner]
44252	F250z00	Generalised nonconvulsive epilepsy NOS
45757	9Of3.00	Epilepsy monitoring verbal invite
45927	F251y00	Other specified generalised convulsive epilepsy
46103	PK5..12	Epiloia
48134	F255100	Sensory induced epilepsy
48462	Eu06013	[X]Limbic epilepsy personality
49340	F251111	Otohara syndrome
49889	ZS82.00	Acquired epileptic aphasia
50012	6674.00	Epilepsy associated problems
50702	667H.00	Epilepsy prevents employment
51998	F259.11	Ohtahara syndrome
53483	F25y100	Gelastic epilepsy
55260	F25y000	Cursive (running) epilepsy
55665	F254300	Limbic system epilepsy
55739	F255400	Visual reflex epilepsy
56359	F25D.00	Menstrual epilepsy
59120	Fyu5200	[X]Other status epilepticus
59185	F250y00	Other specified generalised nonconvulsive epilepsy
65673	F25E.00	Stress-induced epilepsy
65699	F255012	Motor epilepsy

68486	F256.11	Lightning spasms
68946	F255500	Unilateral epilepsy
69831	Fyu5100	[X]Other epilepsy
71719	F257.00	Kojevnikov's epilepsy
71801	Fyu5900	[X]Status epilepticus, unspecified
72384	9Of4.00	Epilepsy monitoring telephone invite
73542	F255300	Visceral reflex epilepsy
96641	F25y500	Panayiotopoulos syndrome
98442	9Of5.00	Epilepsy monitoring call first letter
98536	9Of6.00	Epilepsy monitoring call second letter
98870	F255311	Partial epilepsy with autonomic symptoms
98977	9Of7.00	Epilepsy monitoring call third letter
99548	F250100	Pykno-epilepsy
99731	Fyu5000	[X]Other generalized epilepsy and epileptic syndromes

CPRD Aurum: Epilepsy		
**medcode**	**term**	**snomedconceptID**
12876013	Epiloia	7199000
23819016	Grand mal status	13973009
46968015	West syndrome	28055006
46970012	Hypsarrhythmia	28055006
46971011	Salaam attacks	28055006
46972016	Lightning spasms	28055006
148427014	Gelastic epilepsy	89525009
178739011	Epilepsy	84757009
199568011	Grand mal seizure	54200006
264629018	Epilepsy monitoring	170702005
264634019	Epilepsy associated problems	170706008
264645016	Epilepsy monitoring NOS	170702005
297289012	Other specified generalised convulsive epilepsy	65120008
297290015	Generalised convulsive epilepsy NOS	65120008
297295013	Partial epilepsy with impairment of consciousness	192999003
297296014	Temporal lobe epilepsy	193000002
297300010	Psychosensory epilepsy	193002005
297302019	Epileptic automatism	193004006
297303012	Partial epilepsy with impairment of consciousness NOS	192999003
297311019	Somatosensory epilepsy	193008009
297312014	Partial epilepsy with autonomic symptoms	193009001
297313016	Visceral reflex epilepsy	193009001
297314010	Visual reflex epilepsy	193010006
297315011	Unilateral epilepsy	193011005
297321010	Kojevnikov's epilepsy	241006
297324019	Other forms of epilepsy	84757009
297325018	Cursive (running) epilepsy	193021002
297330019	Other forms of epilepsy NOS	84757009
299334015	[X]Other generalized epilepsy and epileptic syndromes	84757009
299336018	[X]Other epilepsy	84757009
345225017	Focal epilepsy	230381009
345281011	Lennox-Gastaut syndrome	230418006
345302015	Ohtahara syndrome	230429005
345321011	Menstrual epilepsy	230444006
345323014	Nocturnal epilepsy	230445007
399395015	Progressive myoclonic epilepsy	267581004
399408014	Epilepsy NOS	84757009
450602015	Motor epilepsy	307356008
450603013	Jacksonian, focal or motor epilepsy	307357004
459233015	Epilepsy control good	314827004
459234014	Epilepsy control poor	314828009
472410017	Tonic-clonic epilepsy	352818000
472411018	Grand mal epilepsy	352818000
477355017	Psychomotor epilepsy	361123003
485041012	Post-ictal state	31758001
495804010	Pykno-epilepsy	50866000
500033015	Generalised convulsive epilepsy	65120008
512000013	Photosensitive epilepsy	95208000
1489345013	Transient epileptic amnesia	395689002

2159216019	Epilepsy severity	407616001
2159219014	2 to 4 seizures a month	407619008
2159220015	1 to 7 seizures a week	407620002
2159221016	Daily seizures	407621003
2159222011	Many seizures a day	407622005
2159227017	Epilepsy confirmed	407627004
2478828012	Traumatic epilepsy	75023009
2548491016	Epilepsy monitoring verbal invite	416090009
2550890017	Generalised non-convulsive epilepsy	192979009
3506708010	Pyridoxine-dependent epilepsy	734434007
3510617017	Intractable idiopathic partial epilepsy	290741000119102
3511291019	Recurrent complex partial epilepsy	116401000119105
10791000006116	Other specified generalised nonconvulsive epilepsy	192979009
103531000006110	Tonic-clonic epilepsy	352818000
141991000000116	Epilepsy prevents employment	93151000000108
148591000006114	Sensory induced epilepsy	79745005
223811000000118	Generalised epilepsy	19598007
230321000006117	Petit mal (minor) epilepsy	509341000000107
245341000006117	Localisation-related epilepsy	230381009
245351000006115	Partial epilepsy	230381009
245361000006118	Partial epilepsy without impairment of consciousness OS	230381009
253231000006116	Otohara syndrome	192990004
362161000006110	Acquired epileptic aphasia	230438007
379901000006113	[X]Epileptic psychosis NOS	111479008
395221000006119	[X]Limbic epilepsy personality	36217008
406781000000119	Epilepsy monitoring telephone invite	248371000000106
425631000006118	[X]Schizophrenia-like psychosis in epilepsy	5510009
632721000000111	Panayiotopoulos syndrome	230387008
647301000006112	Epileptic absences	509341000000107
736371000006112	Localization-related(focal)(partial)idiopathic epilepsy and epileptic syndromes with seizures of localised onset	193022009
743461000006119	Limbic system epilepsy	193003000
800721000006115	Generalised nonconvulsive epilepsy NOS	192979009
851121000006117	Partial epilepsy	851121000006101
883041000006118	Petit mal epilepsy	509341000000107
897431000006113	Post traumatic epilepsy	75023009
916571000006117	Epilepsy monitoring	916571000006101
918561000006111	Epilepsy associated problems	918561000006107
918601000006111	Nocturnal epilepsy	918601000006107
1855741000006114	Epilepsy monitoring in primary care	1855741000006105
1855761000006113	Epilepsy monitoring in secondary care	810161000000101
2589101000000111	SUDEP - sudden unexpected death in epilepsy	719425009
2612101000006119	Petit mal status	7033004
2811081000006110	Generalized epilepsy	19598007
3093151000006111	Idiopathic generalised epilepsy	36803009
3093161000006113	Idiopathic generalized epilepsy	36803009
3093171000006118	Primary generalised epilepsy	36803009
3210941000006113	Temporal-central focal epilepsy	44145005
3210951000006110	Centrotemporal epilepsy	44145005
3668321000006119	Symptomatic generalised epilepsy	71831005
3668331000006116	Symptomatic generalized epilepsy	71831005
3668341000006114	Secondary generalized epilepsy	71831005
3796891000006117	Reflex epilepsy	79745005
3796901000006118	Sensory-induced epilepsy	79745005
3878491000006111	Epileptic	84757009
3878501000006115	EP - Epilepsy	84757009
4052921000006118	Photogenic epilepsy	95208000
4769611000006117	TLE - Temporal lobe epilepsy	193000002
4769641000006118	Mesiobasal limbic epilepsy	193003000
5007201000006118	Primary inherited reading epilepsy	230389006
5007211000006115	Localisation-related symptomatic epilepsy	230390002
5007231000006114	Amygdalo-hippocampal epilepsy	230391003
5007251000006119	Lateral temporal epilepsy	230393000
5007261000006117	Frontal lobe epilepsy	230394006
5007271000006112	Supplementary motor epilepsy	230395007
5007291000006113	Anterior frontopolar epilepsy	230397004

5007321000006116	Opercular epilepsy	230400008
5007351000006113	Parietal lobe epilepsy	230403005
5007361000006110	Occipital lobe epilepsy	230404004
5007411000006119	Hemiplegia-hemiconvulsion-epilepsy syndrome	230407006
5007441000006115	Localisation-related cryptogenic epilepsy	230408001
5007521000006114	Myoclonic epilepsy of early childhood	230412007
5007541000006119	Epilepsy with grand mal seizures on awakening	230414008
5007551000006117	Cryptogenic generalised epilepsy	230415009
5007621000006113	Myoclonic astatic epilepsy	230421008
5007661000006119	Myoclonic absence epilepsy	230422001
5007771000006116	Cryptogenic myoclonic epilepsy	230427007
5007781000006118	Idiopathic myoclonic epilepsy	230428002
5007811000006116	Symptomatic myoclonic epilepsy	230430000
5007871000006113	Epilepsy undetermined whether focal or generalised	230435005
5007941000006110	Epilepsy with continuous spike wave during slow-wave sleep	230439004
5007971000006119	Secondary reading epilepsy	230440002
5008031000006115	Sleep-related epilepsy	230445007
5008101000006110	Eating epilepsy	230450001
5008121000006117	Decision-making epilepsy	230453004
5023531000006114	Epileptic psychosis	231449007
5067711000006114	Triple X syndrome, epilepsy, and hypogammaglobulinaemia	234639001
5067721000006118	Triple X syndrome, epilepsy, and hypogammaglobulinemia	234639001
5614161000006114	Localisation-related idiopathic epilepsy	278510009
6994761000006114	Refractory localisation-related epilepsy	422724001
7040931000006111	Intractable frontal lobe epilepsy	425237009
7306821000006113	Refractory epilepsy	445355009
7306831000006111	Intractable epilepsy	445355009
7512491000006112	Refractory myoclonic epilepsy	698762005
7512581000006113	Post-cerebrovascular accident epilepsy	698767004
7758091000006113	X-linked epilepsy with learning disability and behaviour disorder syndrome	717223008
7840671000006115	Epileptic encephalopathy	723125008
7861341000006115	Epilepsy due to stroke	724787004
7863641000006114	Epilepsy with mesial temporal sclerosis	724989008
7952741000006112	Epilepsy, microcephaly, skeletal dysplasia syndrome	733031004
7952761000006111	Epilepsy telangiectasia syndrome	733032006
8017491000006115	Primary generalised absence epilepsy	47391000119107
8017501000006111	Primary generalized absence epilepsy	47391000119107
8025071000006110	Intractable partial temporal lobe epilepsy with impairment of consciousness	84201000119105
8053261000006111	Atypical absence epilepsy	187931000119106
8079791000006114	Epilepsy monitoring telephone invitation	248371000000106
8191681000006117	Petit-mal epilepsy	509341000000107
11831381000006120	Infantile-onset symptomatic epilepsy syndrome	722762005
12027451000006112	SCN8A-related epilepsy with encephalopathy	765170001
12076991000006114	STXBP1 encephalopathy with epilepsy	768666006
13491541000006114	Early-onset epileptic encephalopathy and intellectual disability due to GRIN2A mutation	770431001
13493431000006116	Mesial temporal lobe epilepsy with hippocampal sclerosis	770643005
13499471000006116	Cortical dysplasia with focal epilepsy syndrome	771142009
13500261000006114	Infantile epileptic dyskinetic encephalopathy	771223000
13502801000006112	Autism epilepsy syndrome due to branched chain ketoacid dehydrogenase kinase deficiency	771448004
13503061000006114	Early-onset spastic ataxia, myoclonic epilepsy, neuropathy syndrome	771469002
13523971000006112	Autosomal recessive cerebellar ataxia, epilepsy, intellectual disability syndrome due to TUD deficiency	773498006
13572191000006116	KCNQ2-related epileptic encephalopathy	778001003
13572931000006116	Myoclonic epilepsy in non-progressive encephalopathy	778047006
13573141000006116	Cryptogenic late-onset epileptic spasms	778063003
13622561000006112	Congenital muscular dystrophy with intellectual disability and severe epilepsy	782772000
13632801000006112	Familial temporal lobe epilepsy	783739005
13637261000006112	Familial infantile myoclonic epilepsy	784342008
13637841000006112	Familial mesial temporal lobe epilepsy with febrile seizures	784372002
13791581000006112	Myoclonic epilepsy with ragged red fibres	230426003
13904831000006116	Alopecia, epilepsy, intellectual disability syndrome Moynahan type	788417006
14055041000006118	Scar epilepsy	322112361000132096
14552291000006112	Early-onset epilepsy, intellectual disability, brain anomalies syndrome	1172627007
14790031000006114	CNTNAP2-related developmental and epileptic encephalopathy	1230376005

Hospital episode statistics: Epilepsy	
icd	Description
G40	Epilepsy
G41	Status epilepticus
G06.8	Epileptic psychosis NOS
F80.3	Acquired aphasia with epilepsy [Landau-Kleffner]

Clinical codes for hypertension

GOLD: clinical, referral, tests; Aurum: consultations, observations.

Process measures (monitoring, referrals) not included. Pregnancy indicators retained, but unlikely to be relevant to people needing EOLC support.

Additional sources of information in addition to clinical opinion and in-house code repository: MiFoot study https://www.mifoot.org.uk/

CPRD GOLD: Hypertension		
medcode	readcode	readterm
204	G2...00	Hypertensive disease
245	G410.00	Primary pulmonary hypertension
351	G20..11	High blood pressure
799	G20..00	Essential hypertension
1894	G201.00	Benign essential hypertension
3712	G20z.11	Hypertension NOS
4372	G202.00	Systolic hypertension
4668	G22..00	Hypertensive renal disease
5433	F282.00	Benign intracranial hypertension
7057	G2z..00	Hypertensive disease NOS
7329	G24..00	Secondary hypertension
8732	G2...11	BP - hypertensive disease
8857	G21z011	Cardiomegaly - hypertensive
10818	G20z.00	Essential hypertension NOS
15106	G22z.00	Hypertensive renal disease NOS
15377	G200.00	Malignant essential hypertension
16059	G24z.00	Secondary hypertension NOS
16173	G21zz00	Hypertensive heart disease NOS
16292	G21..00	Hypertensive heart disease
18765	G2y..00	Other specified hypertensive disease
21837	G232.00	Hypertensive heart&renal dis wth (congestive) heart failure
25371	G241000	Secondary benign renovascular hypertension
26347	G8y3.00	Chronic peripheral venous hypertension
26631	Q000.00	Fetus or neonate affected by maternal hypertensive disease
28684	G233.00	Hypertensive heart and renal disease with renal failure
29310	G22z.11	Renal hypertension
30776	6629	Hypertension:follow-up default
31387	G24z000	Secondary renovascular hypertension NOS
31464	G21z.00	Hypertensive heart disease NOS
31755	G240.00	Secondary malignant hypertension
31816	G672.11	Hypertensive crisis
32423	G222.00	Hypertensive renal disease with renal failure
34065	G41y000	Secondary pulmonary hypertension
34744	G244.00	Hypertension secondary to endocrine disorders
39649	G220.00	Malignant hypertensive renal disease
42229	G24zz00	Secondary hypertension NOS
43935	G221.00	Benign hypertensive renal disease
50157	G210.00	Malignant hypertensive heart disease
51635	G241z00	Secondary benign hypertension NOS
52127	G211100	Benign hypertensive heart disease with CCF
52427	G211.00	Benign hypertensive heart disease
57288	G241.00	Secondary benign hypertension
57987	G234.00	Hyperten heart&renal dis+both(congestv)heart and renal fail

59383	G240000	Secondary malignant renovascular hypertension
61166	G21z000	Hypertensive heart disease NOS without CCF
61660	G211000	Benign hypertensive heart disease without CCF
62718	G21z100	Hypertensive heart disease NOS with CCF
63000	G231.00	Benign hypertensive heart and renal disease
63466	G23..00	Hypertensive heart and renal disease
67232	G230.00	Malignant hypertensive heart and renal disease
68659	G23z.00	Hypertensive heart and renal disease NOS
69753	Gyu2.00	[X]Hypertensive diseases
72668	G210100	Malignant hypertensive heart disease with CCF
73293	G240z00	Secondary malignant hypertension NOS
83473	G203.00	Diastolic hypertension
95334	G210000	Malignant hypertensive heart disease without CCF
97533	Gyu2100	[X]Hypertension secondary to other renal disorders
102444	G41y100	Thromboembolic pulmonary hypertension
102458	Gyu2000	[X]Other secondary hypertension
103046	G210z00	Malignant hypertensive heart disease NOS
105274	G28..00	Stage 2 hypertension (NICE - Nat Ins for Hth Clin Excl 2011)
105316	G25..11	Stage 1 hypertension
105371	G25..00	Stage 1 hypertension (NICE - Nat Ins for Hth Clin Excl 2011)
105487	G26..11	Severe hypertension
105938	G211z00	Benign hypertensive heart disease NOS
107704	G20..12	Primary hypertension
108136	G250.00	Stage 1 hyperten (NICE 2011) without evidnce end organ damge
109797	G251.00	Stage 1 hyperten (NICE 2011) with evidnce end organ damge

CPRD Aurum: Hypertension

medcode	term	snomedconceptID
1409014	Benign hypertensive renal disease	193003
3135013	Benign essential hypertension	1201005
43850011	Pulmonary arterial hypertension	11399002
47076011	Renal hypertension	38481006
53452019	Secondary hypertension	31992008
60444016	Benign hypertensive heart disease	36221001
64168014	Hypertensive disease	38341003
64172013	Elevated blood pressure	24184005
64282015	Hypertensive renal disease	38481006
80224019	Diastolic hypertension	48146000
84112010	Hypertensive crisis	50490005
90135019	Malignant hypertensive heart disease	54225002
93494011	Systolic hypertension	56218007
99042012	Essential hypertension	59621000
99047018	Primary hypertension	59621000
107545013	Hypertensive heart disease	64715009
108730018	Malignant hypertensive renal disease	65443008
109700019	Benign hypertensive heart AND renal disease	66052004
110659019	Malignant hypertensive heart AND renal disease	66610008
131046010	Malignant essential hypertension	78975002
143003017	Hypertensive heart AND renal disease	86234004
146259010	Secondary pulmonary hypertension	88223008
264473010	Hypertension:follow-up default	170579000
299650019	Malignant hypertensive heart disease NOS	54225002
299654011	Benign hypertensive heart disease NOS	36221001
299655012	Hypertensive heart disease NOS	64715009
299665018	Hypertensive renal disease with renal failure	49220004
299673010	Hypertensive heart and renal disease with renal failure	194780003
299675015	Hypertensive heart and renal disease NOS	86234004
299676019	Secondary malignant renovascular hypertension	194783001
299677011	Secondary malignant hypertension NOS	89242004
299678018	Secondary benign hypertension	194785008
299680012	Benign secondary hypertension	194785008
299681011	Hypertension secondary to endocrine disorders	194788005
299682016	Secondary hypertension NOS	31992008

299683014	Renovascular hypertension	123799005
299686018	Other specified hypertensive disease	38341003
299687010	Hypertensive disorder	38341003
300869017	[X]Hypertensive diseases	38341003
300870016	[X]Other secondary hypertension	31992008
300871017	[X]Hypertension secondary to other renal disorders	31992008
350517010	Thromboembolic pulmonary hypertension	233947005
395751018	Essential hypertension NOS	59621000
395753015	Hypertensive renal disease NOS	38481006
411508017	Cardiomegaly - hypertensive	275516004
2478822013	Secondary benign renovascular hypertension	73410007
3636694012	Combined diastolic and systolic hypertension	762463000
3636695013	Diastolic hypertension and systolic hypertension	762463000
3636696014	Diastolic hypertension co-occurrent with systolic hypertension	762463000
151161000006115	Malignant secondary hypertension	89242004
158241000006117	Secondary hypertension NOS	31992008
504901000006118	Benign hypertensive heart disease with congestive cardiac failure	194767001
504911000006115	Benign hypertensive heart disease without CCF	77970009
523801000006119	BP - hypertensive disease	38341003
728671000006119	Malignant hypertensive heart disease with congestive cardiac failure	83105008
728681000006116	Malignant hypertensive heart disease without congestive heart failure	36315003
741661000006118	Hypertensive heart disease NOS	64715009
741681000006111	Hypertensive heart disease NOS with CCF	64715009
741691000006114	Hypertensive heart disease NOS without CCF	64715009
741701000006114	Hypertensive heart and renal disease with (congestive) heart failure	194779001
789941000006117	Hypertensive heart and renal disease with both (congestive) heart failure and renal failure	194781004
790121000006116	Hypertension	38341003
884121000006111	Malignant hypertension	78975002
884131000006114	Hypertensive renal + heart dis	86234004
1749291000006113	Thromboembolic pulmonary hypertension	1749291000006109
1806071000006118	Stage 1 hypertension	1806071000006102
1806081000006115	Stage 2 hypertension	1806081000006104
1806141000006113	Severe hypertension	1806141000006109
1823901000006112	Hypertension confirmed	1823901000006108
1908711000006115	Stage 1 hypertension (NICE 2011) without evidence of end organ damage	908631000000108
1908721000006111	Stage 1 hypertension (NICE 2011) with evidence of end organ damage	908651000000101
2193021000000110	Severe hypertension	843841000000109
2193031000000112	Stage 1 hypertension	843821000000102
2670511000006119	Benign hypertension	10725009
2674471000006117	Progressive pulmonary hypertension	10964002
2737251000006112	Renal sclerosis with hypertension	14973001
2951231000006117	Renal hypertension	28119000
3117411000006118	High blood pressure	38341003
3117421000006114	Hypertensive vascular disease	38341003
3117461000006115	Systemic arterial hypertension	38341003
3117481000006113	HT - Hypertension	38341003
3117511000006117	HTN - Hypertension	38341003
3117521000006113	Hypertensive disorder, systemic arterial	38341003
3119661000006114	Hypertensive nephropathy	38481006
3128041000006117	Renal arterial hypertension	39018007
3244761000006113	Hypertensive heart failure	46113002
3250801000006112	Low-renin essential hypertension	46481004
3296351000006114	Hypertensive renal failure	49220004
3437381000006110	Parenchymal renal hypertension	57684003
3468491000006113	Idiopathic hypertension	59621000
3468501000006117	Systemic primary arterial hypertension	59621000
3470001000006117	Sustained diastolic hypertension	59720008
3489281000006119	Hypertensive heart disease without congestive heart failure	60899001
3552501000006119	Hypertensive cardiopathy	64715009
3565371000006115	Labile diastolic hypertension	65518004
3642801000006112	Malignant hypertension	70272006
3654631000006113	Pulmonary hypertension	70995007

3654641000006115	PHT - Pulmonary hypertension	70995007
3710191000006113	Secondary diastolic hypertension	74451002
3784371000006115	Accelerated essential hypertension	78975002
3950631000006119	Accelerated secondary hypertension	89242004
4356081000006115	Hypertension due to renovascular disease	123799005
4775831000006118	Hypertension secondary to endocrine disorder	194788005
5057801000006113	Chronic thromboembolic pulmonary hypertension	233947005
5590581000006114	Pulmonary hypertension with occult mitral stenosis	276793003
6214691000006113	Post-capillary pulmonary hypertension	360573001
6214751000006112	Pulmonary hypertension secondary to raised pulmonary vascular resistance	360578005
6349461000006119	Labile essential hypertension	371125006
6592161000006112	Hypertension with albuminuria	397748008
7052811000006113	Right heart failure due to pulmonary hypertension	426012001
7084301000006117	Hypertensive left ventricular hypertrophy	428163005
7103581000006114	Systolic essential hypertension	429457004
7487571000006119	Hypertensive nephrosclerosis	473392002
7501601000006111	Precapillary pulmonary hypertension	697896007
7501621000006118	Idiopathic pulmonary arterial hypertension	697898008
7501711000006118	Pulmonary arterial hypertension associated with connective tissue disease	697903007
7501811000006110	Pulmonary hypertension due to lung disease and/or hypoxia	697910001
7501941000006116	Pulmonary hypertension in sarcoidosis	697921005
7593681000006118	Hypertension concurrent and due to end stage renal disease on dialysis	704667004
7609711000006110	Hypertensive crisis	706882009
7617191000006113	Chronic pulmonary thromboembolism without pulmonary hypertension	707413005
8030421000006114	Hypertensive heart AND chronic kidney disease stage 4	96721000119103
8030431000006112	Hypertensive heart AND chronic kidney disease stage 3	96731000119100
8030441000006119	Hypertensive heart AND chronic kidney disease stage 2	96741000119109
8030451000006117	Hypertensive heart AND chronic kidney disease stage 1	96751000119106
8036941000006111	Chronic kidney disease stage 1 due to hypertension	117681000119102
8040661000006119	Chronic kidney disease stage 4 due to hypertension	129151000119102
8040671000006114	Chronic kidney disease stage 5 due to hypertension	129161000119100
8040681000006112	Chronic kidney disease stage 3 due to hypertension	129171000119106
8040691000006110	Chronic kidney disease stage 2 due to hypertension	129181000119109
8044011000006117	Hypertension in chronic kidney disease stage 5 due to type 2 diabetes mellitus	140101000119109
8044061000006119	Hypertension in chronic kidney disease stage 3 due to type 2 diabetes mellitus	140121000119100
8087071000006112	Chronic kidney disease stage 3 due to benign hypertension	284991000119104
8087661000006111	Malignant hypertensive chronic kidney disease stage 1	285851000119102
8286321000006117	Resistant hypertension	845891000000103
8286581000006114	Stage 2 hypertension	846371000000103
8298681000006113	Nocturnal hypertension	863191000000102
9897511000006110	Labile systemic arterial hypertension	16229371000119106
13941061000006112	Mild pulmonary hypertension	840306007
13941071000006116	Moderate pulmonary hypertension	840307003
13941081000006118	Severe pulmonary hypertension	840308008
14639941000006112	Cardiomegaly due to hypertension	275516004

Hospital episode statistics: Hypertension	
**icd**	**description**
I10	Essential (primary) hypertension
I11	Hypertensive heart disease
I12	Hypertensive renal disease
I13	Hypertensive heart and renal disease
I15	Secondary hypertension

Clinical codes for mental ill-health (other than autism)

GOLD: clinical, referral, tests; Aurum: consultations, observations.

Restricted to psychotic disorders, bipolar disorders, anxiety, depression, attention deficity hyperactivity disorders (ADHD), eating disorders, and personality disorders. Phobic anxiety disorders not included. Process measures (monitoring, referrals) not included.

Additional sources of information in addition to clinical opinion and in-house code repository: MiFoot study https://www.mifoot.org.uk/; Morriss et al. 2021: https://journals.plos.org/plosone/article?id=10.1371/journal.pone.0245722; Morgan et al. 2023: https://clinicalcodes.rss.mhs.man.ac.uk/medcodes/article/201/codelist/res201-personality-disorders/
Hire et al. 2015: https://clinicalcodes.rss.mhs.man.ac.uk/medcodes/article/28/codelist/res28-adhd-medcodes/
Hoile et al. 2019: https://clinicalcodes.rss.mhs.man.ac.uk/medcodes/article/78/codelist/res38-eating-disorder/

CPRD GOLD: Mental ill-health (other than autism)			
**medcode**	**readcode**	**readterm**	**interpretation**
62449	Eu21.14	[X]Prepsychotic schizophrenia	Psychotic disorders
16764	Eu20000	[X]Paranoid schizophrenia	Psychotic disorders
33410	Eu25z11	[X]Schizoaffective psychosis NOS	Psychotic disorders
39316	Eu21.00	[X]Schizotypal disorder	Psychotic disorders
102475	Eu1A500	[X]Mental behav disord due crack cocaine: psychotic disorder	Psychotic disorders
44503	Eu23y00	[X]Other acute and transient psychotic disorders	Psychotic disorders
99000	E107.11	Cyclic schizophrenia	Psychotic disorders
23538	E13y100	Brief reactive psychosis	Psychotic disorders
49223	Eu22z00	[X]Persistent delusional disorder, unspecified	Psychotic disorders
25283	R001200	[D]Hallucinations, olfactory	Psychotic disorders
62405	Eu22100	[X]Delusional misidentification syndrome	Psychotic disorders
26002	E02z.00	Drug psychosis NOS	Psychotic disorders
25546	E102.00	Catatonic schizophrenia	Psychotic disorders
34966	Eu20z00	[X]Schizophrenia, unspecified	Psychotic disorders
35274	Eu25111	[X]Schizoaffective psychosis, depressive type	Psychotic disorders
49879	Eu15500	[X]Mental/behav dis oth stims inc caffeine: psychotic dis	Psychotic disorders
59096	Eu23211	[X]Brief schizophreniform disorder	Psychotic disorders
34236	Eu20.00	[X]Schizophrenia	Psychotic disorders
58862	E107000	Unspecified schizo-affective schizophrenia	Psychotic disorders
33425	E11zz00	Other affective psychosis NOS	Psychotic disorders
39625	E04z.00	Chronic organic psychosis NOS	Psychotic disorders
22644	286..11	Poor insight into psychotic condition	Psychotic disorders
4843	Eu22015	[X]Paranoia	Psychotic disorders
50964	Eu11500	[X]Mental & behav dis due to use opioids: psychotic disorder	Psychotic disorders
44498	E100400	Acute exacerbation of chronic schizophrenia	Psychotic disorders
64131	R001300	[D]Hallucinations, tactile	Psychotic disorders
9422	Eu25.00	[X]Schizoaffective disorders	Psychotic disorders
8766	Eu0z.12	[X]Symptomatic psychosis NOS	Psychotic disorders
97919	E101400	Acute exacerbation of chronic hebephrenic schizophrenia	Psychotic disorders
49565	Eu14500	[X]Mental & behav dis due to use cocaine: psychotic disorder	Psychotic disorders
34168	Eu23z00	[X]Acute and transient psychotic disorder, unspecified	Psychotic disorders
112529	Eu17500	[X]Mental & behav dis due to use tobacco: psychotic disorder	Psychotic disorders
50060	Eu20011	[X]Paraphrenic schizophrenia	Psychotic disorders
68111	E01yz00	Other alcoholic psychosis NOS	Psychotic disorders
48054	E101z00	Hebephrenic schizophrenia NOS	Psychotic disorders
49852	Eu21.16	[X]Pseudoneurotic schizophrenia	Psychotic disorders
33847	Eu25000	[X]Schizoaffective disorder, manic type	Psychotic disorders
53761	Ryu5300	[X]Other hallucinations	Psychotic disorders
32222	E100.00	Simple schizophrenia	Psychotic disorders
33383	E103000	Unspecified paranoid schizophrenia	Psychotic disorders
36720	Eu23000	[X]Acute polymorphic psychot disord without symp of schizoph	Psychotic disorders
57666	E100300	Acute exacerbation of subchronic schizophrenia	Psychotic disorders
31707	Eu23z11	[X]Brief reactive psychosis NOS	Psychotic disorders
41022	Eu25112	[X]Schizophreniform psychosis, depressive type	Psychotic disorders
53985	Eu20111	[X]Disorganised schizophrenia	Psychotic disorders
50248	Eu22y12	[X]Involutional paranoid state	Psychotic disorders
92994	E10y.11	Cenesthopathic schizophrenia	Psychotic disorders
694	Eu2z.11	[X]Psychosis NOS	Psychotic disorders
63867	E102z00	Catatonic schizophrenia NOS	Psychotic disorders
16905	Eu25011	[X]Schizoaffective psychosis, manic type	Psychotic disorders
60013	Eu20300	[X]Undifferentiated schizophrenia	Psychotic disorders
91547	Eu20311	[X]Atypical schizophrenia	Psychotic disorders
101054	Eu32900	[X]Single major depr ep, severe with psych, psych in remiss	Psychotic disorders
20228	E13..11	Reactive psychoses	Psychotic disorders
1915	1BH..00	Delusions	Psychotic disorders

11172	Eu22012	[X]Paranoid state	Psychotic disorders
41992	E11z.00	Other and unspecified affective psychoses	Psychotic disorders
20785	Eu20400	[X]Post-schizophrenic depression	Psychotic disorders
14965	E13z.00	Nonorganic psychosis NOS	Psychotic disorders
51322	E103300	Acute exacerbation of subchronic paranoid schizophrenia	Psychotic disorders
35848	Eu20600	[X]Simple schizophrenia	Psychotic disorders
854	E10..00	Schizophrenic disorders	Psychotic disorders
66410	E105.00	Latent schizophrenia	Psychotic disorders
11055	Eu25100	[X]Schizoaffective disorder, depressive type	Psychotic disorders
30985	Eu2y.00	[X]Other nonorganic psychotic disorders	Psychotic disorders
65127	Eu22014	[X]Sensitiver Beziehungswahn	Psychotic disorders
33670	E01y.00	Other alcoholic psychosis	Psychotic disorders
28562	Eu22.00	[X]Persistent delusional disorders	Psychotic disorders
9281	E103z00	Paranoid schizophrenia NOS	Psychotic disorders
43462	1BH0.00	Delusion of persecution	Psychotic disorders
26119	E13yz00	Other reactive psychoses NOS	Psychotic disorders
16537	E1y..00	Other specified non-organic psychoses	Psychotic disorders
61501	Eu20200	[X]Catatonic schizophrenia	Psychotic disorders
24387	Eu04.13	[X]Acute / subacute infective psychosis	Psychotic disorders
35877	Eu20213	[X]Schizophrenic catatonia	Psychotic disorders
56143	E141.00	Disintegrative psychosis	Psychotic disorders
53625	E100z00	Simple schizophrenia NOS	Psychotic disorders
94001	Eu20y12	[X]Schizophreniform disord NOS	Psychotic disorders
43405	Eu20100	[X]Hebephrenic schizophrenia	Psychotic disorders
70884	Eu23212	[X]Brief schizophrenifrm psych	Psychotic disorders
50218	E110400	Single manic episode, severe, with psychosis	Psychotic disorders
25019	Eu23.00	[X]Acute and transient psychotic disorders	Psychotic disorders
64993	Eu21.13	[X]Latent schizophrenia	Psychotic disorders
49420	Eu20y00	[X]Other schizophrenia	Psychotic disorders
39062	E10y.00	Other schizophrenia	Psychotic disorders
3984	E100200	Chronic schizophrenic	Psychotic disorders
27770	Eu23312	[X]Psychogenic paranoid psychosis	Psychotic disorders
66077	Eu22y00	[X]Other persistent delusional disorders	Psychotic disorders
58532	Eu25y00	[X]Other schizoaffective disorders	Psychotic disorders
29651	Eu23z12	[X]Reactive psychosis	Psychotic disorders
11778	Eu23200	[X]Acute schizophrenia-like psychotic disorder	Psychotic disorders
20572	Eu20211	[X]Catatonic stupor	Psychotic disorders
33338	E10y000	Atypical schizophrenia	Psychotic disorders
34389	Eu22000	[X]Delusional disorder	Psychotic disorders
61969	E212200	Schizotypal personality	Psychotic disorders
94604	Eu23214	[X]Schizophrenic reaction	Psychotic disorders
2455	R001.00	[D]Hallucinations	Psychotic disorders
67651	E01z.00	Alcoholic psychosis NOS	Psychotic disorders
55479	1BH2.00	Ideas of reference	Psychotic disorders
17614	Eu53111	[X]Puerperal psychosis NOS	Psychotic disorders
64264	Eu20500	[X]Residual schizophrenia	Psychotic disorders
64533	Eu20212	[X]Schizophrenic catalepsy	Psychotic disorders
31738	Eu2y.11	[X]Chronic hallucinatory psychosis	Psychotic disorders
31493	Eu20214	[X]Schizophrenic flexibilatis cerea	Psychotic disorders
22188	E1z..00	Non-organic psychosis NOS	Psychotic disorders
69138	Eu13500	[X]Mental & behav dis due to seds/hypntcs: psychotic disordr	Psychotic disorders
53032	E103400	Acute exacerbation of chronic paranoid schizophrenia	Psychotic disorders
63478	E107400	Acute exacerbation of chronic schizo-affective schizophrenia	Psychotic disorders
4390	285..11	Psychotic condition, insight present	Psychotic disorders
21985	Eu0z.11	[X]Organic psychosis NOS	Psychotic disorders
58716	E102000	Unspecified catatonic schizophrenia	Psychotic disorders
51903	Eu25012	[X]Schizophreniform psychosis, manic type	Psychotic disorders
102446	E105z00	Latent schizophrenia NOS	Psychotic disorders
15958	E1...00	Non-organic psychoses	Psychotic disorders
12064	R001400	[D]Visual hallucinations	Psychotic disorders
73295	E100.11	Schizophrenia simplex	Psychotic disorders
21455	Eu23012	[X]Cycloid psychosis	Psychotic disorders
8407	E10z.00	Schizophrenia NOS	Psychotic disorders
15053	E133.00	Acute paranoid reaction	Psychotic disorders
12120	R001000	[D]Hallucinations, auditory	Psychotic disorders
57376	ZS7C611	Schizophrenic language	Psychotic disorders

58866	E107300	Acute exacerbation subchronic schizo-affective schizophrenia	Psychotic disorders
47947	Eu22013	[X]Paraphrenia - late	Psychotic disorders
41207	E141100	Residual disintegrative psychoses	Psychotic disorders
11670	Eu10611	[X]Korsakov's psychosis, alcohol induced	Psychotic disorders
18053	Eu20y13	[X]Schizophrenifrm psychos NOS	Psychotic disorders
24345	E134.00	Psychogenic paranoid psychosis	Psychotic disorders
23616	E100100	Subchronic schizophrenia	Psychotic disorders
38429	Eu12500	[X]Mental & behav dis due to cannabinoids: psychotic disordr	Psychotic disorders
11106	E011100	Korsakov's alcoholic psychosis with peripheral neuritis	Psychotic disorders
3890	E121.00	Chronic paranoid psychosis	Psychotic disorders
11244	Eu2z.00	[X]Unspecified nonorganic psychosis	Psychotic disorders
26143	Eu23112	[X]Cycloid psychosis with symptoms of schizophrenia	Psychotic disorders
54387	Eu21.12	[X]Borderline schizophrenia	Psychotic disorders
19916	R001z00	[D]Hallucinations NOS	Psychotic disorders
32875	1BH1.00	Grandiose delusions	Psychotic disorders
21595	Eu23100	[X]Acute polymorphic psychot disord with symp of schizophren	Psychotic disorders
44307	Eu23300	[X]Other acute predominantly delusional psychotic disorders	Psychotic disorders
17982	1BH..11	Delusion	Psychotic disorders
2117	E107.00	Schizo-affective schizophrenia	Psychotic disorders
22643	1BH3.00	Paranoid ideation	Psychotic disorders
24107	Eu20511	[X]Chronic undifferentiated schizophrenia	Psychotic disorders
12353	Eu10500	[X]Mental & behav dis due to use alcohol: psychotic disorder	Psychotic disorders
55236	Eu22y13	[X]Paranoia querulans	Psychotic disorders
40981	Eu22y11	[X]Delusional dysmorphophobia	Psychotic disorders
3636	E13z.11	Psychotic episode NOS	Psychotic disorders
15733	E100000	Unspecified schizophrenia	Psychotic disorders
31984	E13..00	Other nonorganic psychoses	Psychotic disorders
2113	Eu22011	[X]Paranoid psychosis	Psychotic disorders
29937	E131.00	Acute hysterical psychosis	Psychotic disorders
16333	E13y.00	Other reactive psychoses	Psychotic disorders
576	E104.00	Acute schizophrenic episode	Psychotic disorders
53848	Eu84314	[X]Symbiotic psychosis	Psychotic disorders
2114	E03y300	Unspecified puerperal psychosis	Psychotic disorders
12771	E12z.00	Paranoid psychosis NOS	Psychotic disorders
33693	Eu25200	[X]Schizoaffective disorder, mixed type	Psychotic disorders
57993	Eu03.11	[X]Korsakov's psychosis, nonalcoholic	Psychotic disorders
1494	E103.00	Paranoid schizophrenia	Psychotic disorders
23835	E040.11	Korsakoff's non-alcoholic psychosis	Psychotic disorders
31362	E103200	Chronic paranoid schizophrenia	Psychotic disorders
54983	Eu16500	[X]Mental & behav dis due to hallucinogens: psychotic disord	Psychotic disorders
7233	E0...00	Organic psychotic conditions	Psychotic disorders
43800	E107200	Chronic schizo-affective schizophrenia	Psychotic disorders
30619	E101.00	Hebephrenic schizophrenia	Psychotic disorders
28168	Eu44.14	[X]Hysterical psychosis	Psychotic disorders
91511	Eu21.11	[X]Latent schizophrenic reaction	Psychotic disorders
17607	Eu10514	[X]Alcoholic psychosis NOS	Psychotic disorders
4500	E011000	Korsakov's alcoholic psychosis	Psychotic disorders
94299	E105200	Chronic latent schizophrenia	Psychotic disorders
99070	E10y100	Coenesthopathic schizophrenia	Psychotic disorders
99199	E102100	Subchronic catatonic schizophrenia	Psychotic disorders
49761	E10yz00	Other schizophrenia NOS	Psychotic disorders
37681	Eu25z00	[X]Schizoaffective disorder, unspecified	Psychotic disorders
10575	E107z00	Schizo-affective schizophrenia NOS	Psychotic disorders
62222	Eu84312	[X]Disintegrative psychosis	Psychotic disorders
37580	Eu25212	[X]Mixed schizophrenic and affective psychosis	Psychotic disorders
40386	Eu21.15	[X]Prodromal schizophrenia	Psychotic disorders
53990	R001100	[D]Hallucinations, gustatory	Psychotic disorders
11973	Eu24.13	[X]Induced psychotic disorder	Psychotic disorders
101153	Eu32A00	[X]Recurr major depr ep, severe with psych, psych in remiss	Psychotic disorders
98618	Eu18500	[X]Mental & behav dis due to vol solvents: psychotic disordr	Psychotic disorders
38063	E106.00	Residual schizophrenia	Psychotic disorders
66506	E101000	Unspecified hebephrenic schizophrenia	Psychotic disorders
17281	Eu2..00	[X]Schizophrenia, schizotypal and delusional disorders	Psychotic disorders
102311	E105000	Unspecified latent schizophrenia	Psychotic disorders
61098	E107100	Subchronic schizo-affective schizophrenia	Psychotic disorders

55221	Eu22111	[X]Capgras syndrome	Psychotic disorders
24640	E110200	Single manic episode, moderate	Bipolar disorders
27739	E111200	Recurrent manic episodes, moderate	Bipolar disorders
73924	Eu31y11	[X]Bipolar II disorder	Bipolar disorders
15923	E115000	Bipolar affective disorder, currently depressed, unspecified	Bipolar disorders
54195	E116400	Mixed bipolar affective disorder, severe, with psychosis	Bipolar disorders
14728	E110100	Single manic episode, mild	Bipolar disorders
32088	Eu30y00	[X]Other manic episodes	Bipolar disorders
18909	E110.11	Hypomanic psychoses	Bipolar disorders
31535	E116000	Mixed bipolar affective disorder, unspecified	Bipolar disorders
21540	Eu34000	[X]Cyclothymia	Bipolar disorders
22713	1S42.00	Manic mood	Bipolar disorders
11596	E11y000	Unspecified manic-depressive psychoses	Bipolar disorders
5726	Eu3..00	[X]Mood - affective disorders	Bipolar disorders
16808	Eu31000	[X]Bipolar affective disorder, current episode hypomanic	Bipolar disorders
44513	Eu30z00	[X]Manic episode, unspecified	Bipolar disorders
36126	E114100	Bipolar affective disorder, currently manic, mild	Bipolar disorders
8567	E11..11	Bipolar psychoses	Bipolar disorders
55829	E114400	Bipolar affect disord, currently manic,severe with psychosis	Bipolar disorders
28277	Eu31200	[X]Bipolar affect disorder cur epi manic with psychotic symp	Bipolar disorders
36611	E110z00	Manic disorder, single episode NOS	Bipolar disorders
65811	E111300	Recurrent manic episodes, severe without mention psychosis	Bipolar disorders
48632	Eu30212	[X]Mania with mood-incongruent psychotic symptoms	Bipolar disorders
29579	Eu3yy00	[X]Other specified mood affective disorders	Bipolar disorders
28008	Eu3y.00	[X]Other mood affective disorders	Bipolar disorders
21065	Eu30200	[X]Mania with psychotic symptoms	Bipolar disorders
1531	Eu31.11	[X]Manic-depressive illness	Bipolar disorders
6710	Eu31.12	[X]Manic-depressive psychosis	Bipolar disorders
63284	E116300	Mixed bipolar affective disorder, severe, without psychosis	Bipolar disorders
68647	E117200	Unspecified bipolar affective disorder, moderate	Bipolar disorders
33426	E11yz00	Other and unspecified manic-depressive psychoses NOS	Bipolar disorders
63701	E115400	Bipolar affect disord, now depressed, severe with psychosis	Bipolar disorders
4732	Eu31500	[X]Bipolar affect dis cur epi severe depres with psyc symp	Bipolar disorders
37070	E110.00	Manic disorder, single episode	Bipolar disorders
73423	E117300	Unspecified bipolar affective disorder, severe, no psychosis	Bipolar disorders
53840	Eu31y00	[X]Other bipolar affective disorders	Bipolar disorders
31633	Eu3z.11	[X]Affective psychosis NOS	Bipolar disorders
24689	E116100	Mixed bipolar affective disorder, mild	Bipolar disorders
4677	E115.00	Bipolar affective disorder, currently depressed	Bipolar disorders
19967	E111000	Recurrent manic episodes, unspecified	Bipolar disorders
26161	E11..13	Manic psychoses	Bipolar disorders
26299	Eu31100	[X]Bipolar affect disorder cur epi manic wout psychotic symp	Bipolar disorders
42857	Eu34.00	[X]Persistent mood affective disorders	Bipolar disorders
33751	Eu31z00	[X]Bipolar affective disorder, unspecified	Bipolar disorders
63583	E116z00	Mixed bipolar affective disorder, NOS	Bipolar disorders
68326	E117400	Unspecified bipolar affective disorder,severe with psychosis	Bipolar disorders
35607	E115300	Bipolar affect disord, now depressed, severe, no psychosis	Bipolar disorders
26227	E111.00	Recurrent manic episodes	Bipolar disorders
2741	Eu30000	[X]Hypomania	Bipolar disorders
30688	Eu3y011	[X]Mixed affective episode	Bipolar disorders
35734	E115100	Bipolar affective disorder, currently depressed, mild	Bipolar disorders
17385	E114.11	Manic-depressive - now manic	Bipolar disorders
23713	Eu31400	[X]Bipol aff disord, curr epis sev depress, no psychot symp	Bipolar disorders
31316	E116.00	Mixed bipolar affective disorder	Bipolar disorders
14656	E11..00	Affective psychoses	Bipolar disorders
50998	Eu3y000	[X]Other single mood affective disorders	Bipolar disorders
16347	E114300	Bipolar affect disord, currently manic, severe, no psychosis	Bipolar disorders
27986	E117z00	Unspecified bipolar affective disorder, NOS	Bipolar disorders
12173	Eu30.00	[X]Manic episode	Bipolar disorders
16562	Eu31300	[X]Bipolar affect disorder cur epi mild or moderate depressn	Bipolar disorders
46425	E111100	Recurrent manic episodes, mild	Bipolar disorders
4678	Eu30z11	[X]Mania NOS	Bipolar disorders
37090	Eu3z.00	[X]Unspecified mood affective disorder	Bipolar disorders
63150	E116200	Mixed bipolar affective disorder, moderate	Bipolar disorders
27890	E115200	Bipolar affective disorder, currently depressed, moderate	Bipolar disorders
6874	Eu31.00	[X]Bipolar affective disorder	Bipolar disorders

13024	Eu30100	[X]Mania without psychotic symptoms	Bipolar disorders
63698	E117100	Unspecified bipolar affective disorder, mild	Bipolar disorders
14784	E117.00	Unspecified bipolar affective disorder	Bipolar disorders
49763	E117000	Unspecified bipolar affective disorder, unspecified	Bipolar disorders
50243	Eu34y00	[X]Other persistent mood affective disorders	Bipolar disorders
70399	E11y300	Other mixed manic-depressive psychoses	Bipolar disorders
57605	E114z00	Bipolar affective disorder, currently manic, NOS	Bipolar disorders
60178	E11y.00	Other and unspecified manic-depressive psychoses	Bipolar disorders
46415	E111z00	Recurrent manic episode NOS	Bipolar disorders
3702	E114.00	Bipolar affective disorder, currently manic	Bipolar disorders
12831	E115.11	Manic-depressive - now depressed	Bipolar disorders
51032	Eu31y12	[X]Recurrent manic episodes	Bipolar disorders
37296	E115z00	Bipolar affective disorder, currently depressed, NOS	Bipolar disorders
44693	Eu31600	[X]Bipolar affective disorder, current episode mixed	Bipolar disorders
29921	Eu3y100	[X]Other recurrent mood affective disorders	Bipolar disorders
70925	E11y100	Atypical manic disorder	Bipolar disorders
9521	Eu30.11	[X]Bipolar disorder, single manic episode	Bipolar disorders
37102	Eu30211	[X]Mania with mood-congruent psychotic symptoms	Bipolar disorders
46434	E114200	Bipolar affective disorder, currently manic, moderate	Bipolar disorders
32295	E111400	Recurrent manic episodes, severe, with psychosis	Bipolar disorders
35738	E114000	Bipolar affective disorder, currently manic, unspecified	Bipolar disorders
39767	Eu34z00	[X]Persistent mood affective disorder, unspecified	Bipolar disorders
20110	E110000	Single manic episode, unspecified	Bipolar disorders
28863	Eu32314	[X]Single episode of reactive depressive psychosis	Depression
52678	Eu32312	[X]Single episode of psychogenic depressive psychosis	Depression
29342	E113100	Recurrent major depressive episodes, mild	Depression
2639	E204.11	Postnatal depression	Depression
32845	Eu92000	[X]Depressive conduct disorder	Depression
18510	Eu32.12	[X]Single episode of psychogenic depression	Depression
37764	Eu33316	[X]Recurrent severe episodes/reactive depressive psychosis	Depression
19054	Eu3y111	[X]Recurrent brief depressive episodes	Depression
2923	62T1.00	Puerperal depression	Depression
1055	E135.00	Agitated depression	Depression
47009	Eu33300	[X]Recurrent depress disorder cur epi severe with psyc symp	Depression
543	Eu32z11	[X]Depression NOS	Depression
13307	Eu53011	[X]Postnatal depression NOS	Depression
41989	Eu32211	[X]Single episode agitated depressn w'out psychotic symptoms	Depression
32159	E112400	Single major depressive episode, severe, with psychosis	Depression
98346	Eu32500	[X]Major depression, mild	Depression
11329	Eu33211	[X]Endogenous depression without psychotic symptoms	Depression
8902	Eu33.13	[X]Recurrent episodes of reactive depression	Depression
59386	Eu32213	[X]Single episode vital depression w'out psychotic symptoms	Depression
15155	E112200	Single major depressive episode, moderate	Depression
324	E2B..00	Depressive disorder NEC	Depression
65435	9k40.00	Depression - enhanced service completed	Depression
28248	Eu32z13	[X]Prolonged single episode of reactive depression	Depression
10610	E112.00	Single major depressive episode	Depression
101401	8ID..00	Postnatal depression not discussed	Depression
7011	E112z00	Single major depressive episode NOS	Depression
98417	Eu32800	[X]Major depression, severe with psychotic symptoms	Depression
32941	Eu33313	[X]Recurr severe episodes/major depression+psychotic symptom	Depression
7604	Eu32.13	[X]Single episode of reactive depression	Depression
11913	Eu41200	[X]Mixed anxiety and depressive disorder	Depression
4979	Eu53012	[X]Postpartum depression NOS	Depression
11717	Eu32000	[X]Mild depressive episode	Depression
595	E112.14	Endogenous depression	Depression
6932	E113.11	Endogenous depression - recurrent	Depression
29784	Eu33000	[X]Recurrent depressive disorder, current episode mild	Depression
1996	1B17.00	Depressed	Depression
3292	Eu33.00	[X]Recurrent depressive disorder	Depression
28677	Eu33312	[X]Manic-depress psychosis,depressed type+psychotic symptoms	Depression
1908	2257.00	O/E - depressed	Depression
25697	E113300	Recurrent major depressive episodes, severe, no psychosis	Depression
33469	Eu33200	[X]Recurr depress disorder cur epi severe without psyc sympt	Depression

6854	Eu32y00	[X]Other depressive episodes	Depression
7749	Eu41211	[X]Mild anxiety depression	Depression
15099	E113.00	Recurrent major depressive episode	Depression
24117	Eu32311	[X]Single episode of major depression and psychotic symptoms	Depression
2560	E11..12	Depressive psychoses	Depression
16861	Eu33315	[X]Recurrent severe episodes of psychotic depression	Depression
25563	E113z00	Recurrent major depressive episode NOS	Depression
655	E200300	Anxiety with depression	Depression
6546	E112.12	Endogenous depression first episode	Depression
10015	1BT..00	Depressed mood	Depression
23731	Eu33311	[X]Endogenous depression with psychotic symptoms	Depression
36616	Eu33z11	[X]Monopolar depression NOS	Depression
8584	Eu34111	[X]Depressive neurosis	Depression
9667	Eu32200	[X]Severe depressive episode without psychotic symptoms	Depression
24171	E113400	Recurrent major depressive episodes, severe, with psychosis	Depression
10438	1B1U.11	Depressive symptoms	Depression
47731	Eu33y00	[X]Other recurrent depressive disorders	Depression
98252	Eu32600	[X]Major depression, moderately severe	Depression
5879	E112.11	Agitated depression	Depression
31757	Eu33314	[X]Recurr severe episodes/psychogenic depressive psychosis	Depression
35671	E113000	Recurrent major depressive episodes, unspecified	Depression
9211	Eu32100	[X]Moderate depressive episode	Depression
10667	Eu32400	[X]Mild depression	Depression
24112	Eu32313	[X]Single episode of psychotic depression	Depression
6950	E112.13	Endogenous depression first episode	Depression
4639	Eu32.00	[X]Depressive episode	Depression
12099	Eu32300	[X]Severe depressive episode with psychotic symptoms	Depression
1131	E204.00	Neurotic depression reactive type	Depression
56609	Eu32y12	[X]Single episode of masked depression NOS	Depression
8478	E130.00	Reactive depressive psychosis	Depression
98414	Eu32700	[X]Major depression, severe without psychotic symptoms	Depression
22806	Eu32212	[X]Single episode major depression w'out psychotic symptoms	Depression
10720	Eu32y11	[X]Atypical depression	Depression
16506	E112100	Single major depressive episode, mild	Depression
2972	E2B0.00	Postviral depression	Depression
4323	E2B1.00	Chronic depression	Depression
27491	E11y200	Atypical depressive disorder	Depression
34390	E112000	Single major depressive episode, unspecified	Depression
103677	Eu32B00	[X]Antenatal depression	Depression
6482	E113700	Recurrent depression	Depression
3291	Eu32z12	[X]Depressive disorder NOS	Depression
14709	E113200	Recurrent major depressive episodes, moderate	Depression
9796	1B1U.00	Symptoms of depression	Depression
15219	E112300	Single major depressive episode, severe, without psychosis	Depression
9183	E11z200	Masked depression	Depression
29451	Eu33213	[X]Manic-depress psychosis,depressd,no psychotic symptoms	Depression
5987	Eu32z14	[X] Reactive depression NOS	Depression
17770	E130.11	Psychotic reactive depression	Depression
29520	Eu33100	[X]Recurrent depressive disorder, current episode moderate	Depression
7737	Eu34113	[X]Neurotic depression	Depression
73991	Eu33214	[X]Vital depression, recurrent without psychotic symptoms	Depression
15220	Eu34114	[X]Persistant anxiety depression	Depression
19696	Eu33.12	[X]Recurrent episodes of psychogenic depression	Depression
11252	Eu33212	[X]Major depression, recurrent without psychotic symptoms	Depression
28756	Eu33.14	[X]Seasonal depressive disorder	Depression
2970	Eu32z00	[X]Depressive episode, unspecified	Depression
44300	Eu33z00	[X]Recurrent depressive disorder, unspecified	Depression
4171	Eu43100	[X]Post - traumatic stress disorder	Anxiety disorders
70779	Eu43013	[X]Combat fatigue	Anxiety disorders
14780	E20z.00	Neurotic disorder NOS	Anxiety disorders
20634	Eu42000	[X]Predominantly obsessional thoughts or ruminations	Anxiety disorders
20773	Eu05400	[X]Organic anxiety disorder	Anxiety disorders
1758	E200400	Chronic anxiety	Anxiety disorders
38809	Eu42y00	[X]Other obsessive-compulsive disorders	Anxiety disorders

22721	Eu42z00	[X]Obsessive-compulsive disorder, unspecified	Anxiety disorders
101725	Eu43400	[X]Chron post-traumatic stress disorder follow military comb	Anxiety disorders
23838	Eu41z00	[X]Anxiety disorder, unspecified	Anxiety disorders
47365	E203.11	Anancastic neurosis	Anxiety disorders
962	Eu41111	[X]Anxiety neurosis	Anxiety disorders
5385	Eu41.00	[X]Other anxiety disorders	Anxiety disorders
3208	E203.00	Obsessive-compulsive disorders	Anxiety disorders
21836	Eu42.12	[X]Obsessive-compulsive neurosis	Anxiety disorders
2030	E203100	Obsessional neurosis	Anxiety disorders
113199	Eu43500	[X]Delayed post-traumat stress disorder follow military comb	Anxiety disorders
24066	Eu41y00	[X]Other specified anxiety disorders	Anxiety disorders
8205	Eu41000	[X]Panic disorder [episodic paroxysmal anxiety]	Anxiety disorders
4659	E200200	Generalised anxiety disorder	Anxiety disorders
21753	Eu43y00	[X]Other reactions to severe stress	Anxiety disorders
4634	E200500	Recurrent anxiety	Anxiety disorders
4069	E200100	Panic disorder	Anxiety disorders
28167	Eu41y11	[X]Anxiety hysteria	Anxiety disorders
44321	Eu41300	[X]Other mixed anxiety disorders	Anxiety disorders
18399	Eu42200	[X]Mixed obsessional thoughts and acts	Anxiety disorders
10344	Eu41100	[X]Generalized anxiety disorder	Anxiety disorders
5304	Eu42.00	[X]Obsessive - compulsive disorder	Anxiety disorders
5678	E203000	Compulsive neurosis	Anxiety disorders
101785	Eu43300	[X]Acute post-traumatic stress disorder follow military comb	Anxiety disorders
15566	E203z00	Obsessive-compulsive disorder NOS	Anxiety disorders
26285	Eu9y700	[X]Attention deficit disorder	Attention deficit hyperactivity disorders
24808	ZS91.11	ADD - Attention deficit disorder	Attention deficit hyperactivity disorders
37994	ZS9..00	Disorders of attention and motor control	Attention deficit hyperactivity disorders
55322	Eu90200	[X]Deficits in attention, motor control and perception	Attention deficit hyperactivity disorders
1458	Eu90.00	[X]Hyperkinetic disorders	Attention deficit hyperactivity disorders
45799	Eu90111	[X]Hyperkinetic disorder associated with conduct disorder	Attention deficit hyperactivity disorders
37894	ZS94.00	Minimal brain dysfunction	Attention deficit hyperactivity disorders
45263	E2E2.00	Hyperkinetic conduct disorder	Attention deficit hyperactivity disorders
9715	E2E0100	Attention deficit with hyperactivity	Attention deficit hyperactivity disorders
24753	ZS91.12	[X]Attention deficit disorder	Attention deficit hyperactivity disorders
5565	E2E0.00	Child attention deficit disorder	Attention deficit hyperactivity disorders
9972	E2E..11	Overactive child syndrome	Attention deficit hyperactivity disorders
6519	Eu90011	[X]Attention deficit hyperactivity disorder	Attention deficit hyperactivity disorders
35161	ZS93.11	DAMP - Deficits in attention motor control and perception	Attention deficit hyperactivity disorders
28543	ZS91.00	Attention deficit disorder	Attention deficit hyperactivity disorders
10918	1P00.00	Hyperactive behaviour	Attention deficit hyperactivity disorders
34199	E2E0000	Attention deficit without hyperactivity	Attention deficit hyperactivity disorders
96770	Eu90z12	[X]Hyperkinetic syndrome NOS	Attention deficit hyperactivity disorders
39920	ZS93.00	Deficits in attention motor control and perception	Attention deficit hyperactivity disorders
6510	Eu90y00	[X]Other hyperkinetic disorders	Attention deficit hyperactivity disorders
6512	Eu90000	[X]Disturbance of activity and attention	Attention deficit hyperactivity disorders
61701	ZS94.11	MBD - Minimal brain dysfunction	Attention deficit hyperactivity disorders
58069	E2E1.00	Hyperkinesis with developmental delay	Attention deficit hyperactivity disorders
33505	Eu90100	[X]Hyperkinetic conduct disorder	Attention deficit hyperactivity disorders
20467	E2E0z00	Child attention deficit disorder NOS	Attention deficit hyperactivity disorders
50015	Eu90z00	[X]Hyperkinetic disorder, unspecified	Attention deficit hyperactivity disorders
25469	E2Ey.00	Other hyperkinetic manifestation	Attention deficit hyperactivity disorders
41769	E2Ez.00	Hyperkinetic syndrome NOS	Attention deficit hyperactivity disorders
32892	E275z00	Non-organic eating disorder NOS	Eating disorders
98900	R036100	[D]Hyperalimentation	Eating disorders
34995	Eu50y00	[X]Other eating disorders	Eating disorders
9581	Eu50200	[X]Bulimia nervosa	Eating disorders
96475	Eu50212	[X]Hyperorexia nervosa	Eating disorders
60373	1614.11	Hyperalimentation - symptom	Eating disorders
31227	1614.12	Polyphagia symptom	Eating disorders
49601	Fy05.00	Nocturnal sleep-related eating disorder	Eating disorders
17439	Eu50411	[X]Psychogenic overeating	Eating disorders
15235	R036.00	[D]Polyphagia	Eating disorders
17642	R036000	[D]Excessive eating	Eating disorders
2135	E271.00	Anorexia nervosa	Eating disorders
36946	Eu50z00	[X]Eating disorder, unspecified	Eating disorders

7608	1612.00	Appetite loss - anorexia	Eating disorders
6583	Eu50211	[X]Bulimia NOS	Eating disorders
30570	Eu50000	[X]Anorexia nervosa	Eating disorders
7744	1612.11	Anorexia symptom	Eating disorders
11608	E275111	Compulsive eating disorder	Eating disorders
6159	Eu50.00	[X]Eating disorders	Eating disorders
72870	R036z00	[D]Polyphagia NOS	Eating disorders
39383	Eu50400	[X]Overeating associated with other psychological disturbncs	Eating disorders
53746	R030z00	[D]Anorexia NOS	Eating disorders
92992	U1B3.11	[X]Starvation	Eating disorders
44544	E275000	Unspecified non-organic eating disorder	Eating disorders
912	R030.00	[D]Anorexia	Eating disorders
35490	1614.00	Excessive eating - polyphagia	Eating disorders
605	R036011	[D]Bulimia NOS	Eating disorders
33863	Eu50300	[X]Atypical bulimia nervosa	Eating disorders
7743	E275.00	Other and unspecified non-organic eating disorders	Eating disorders
4377	E275100	Bulimia (non-organic overeating)	Eating disorders
62150	Eu50y11	[X]Pica in adults	Eating disorders
23420	SN42100	Starvation	Eating disorders
61236	E275y00	Other specified non-organic eating disorder	Eating disorders
34929	Eu50100	[X]Atypical anorexia nervosa	Eating disorders
51497	E211z00	Affective personality disorder NOS	Personality disorders
20881	E214.00	Compulsive personality disorders	Personality disorders
53335	Eu60y14	[X]Narcissistic personality disorder	Personality disorders
70899	E21y600	Masochistic personality disorder	Personality disorders
30603	Eu61.00	[X]Mixed and other personality disorders	Personality disorders
49600	Eu60y00	[X]Other specific personality disorders	Personality disorders
67130	E212000	Unspecified schizoid personality disorder	Personality disorders
49779	Eu60y13	[X]Immature personality disorder	Personality disorders
21671	Eu60214	[X]Psychopathic personality disorder	Personality disorders
31632	Eu60200	[X]Dissocial personality disorder	Personality disorders
59589	Eu06000	[X]Organic personality disorder	Personality disorders
71250	Eu21.17	[X]Pseudopsychopathic schizophrenia	Personality disorders
48687	Eu60014	[X]Sensitive paranoid personality disorder	Personality disorders
21077	E21y700	Psychoneurotic personality disorder	Personality disorders
39535	Eu60711	[X]Asthenic personality disorder	Personality disorders
5652	E210.00	Paranoid personality disorder	Personality disorders
42496	Eu60z00	[X]Personality disorder, unspecified	Personality disorders
23977	E217.00	Antisocial or sociopathic personality disorder	Personality disorders
8424	Eu60600	[X]Anxious [avoidant] personality disorder	Personality disorders
32869	Eu60212	[X]Antisocial personality disorder	Personality disorders
15960	E21yz00	Other personality disorder NOS	Personality disorders
43690	Eu60400	[X]Histrionic personality disorder	Personality disorders
69000	Eu60013	[X]Querulant personality disorder	Personality disorders
44242	E215000	Unspecified histrionic personality disorder	Personality disorders
33741	Eu60713	[X]Passive personality disorder	Personality disorders
10455	E211200	Depressive personality disorder	Personality disorders
38371	Eu60100	[X]Schizoid personality disorder	Personality disorders
23597	E213.00	Explosive personality disorder	Personality disorders
10290	Eu34112	[X]Depressive personality disorder	Personality disorders
1364	E21y500	Immature personality disorder	Personality disorders
4515	E216.00	Inadequate personality disorder	Personality disorders
31789	Eu60312	[X]Borderline personality disorder	Personality disorders
34896	Eu6yy00	[X]Other specified disorders of adult personality/behaviour	Personality disorders
26859	Eu21.18	[X]Schizotypal personality disorder	Personality disorders
55969	Eu60y11	[X]Eccentric personality disorder	Personality disorders
18565	E21y200	Borderline personality disorder	Personality disorders
71431	Eu60y12	[X]Haltlose type personality disorder	Personality disorders
38031	Eu60712	[X]Inadequate personality disorder	Personality disorders
2076	E21..00	Personality disorders	Personality disorders
37289	E21y000	Narcissistic personality disorder	Personality disorders
27481	E215.00	Histrionic personality disorders	Personality disorders
99428	Eu94211	[X]Affectionless psychopathy	Personality disorders
60522	E215z00	Histrionic personality disorder NOS	Personality disorders
50188	Eu60.00	[X]Specific personality disorders	Personality disorders
12707	E211300	Cyclothymic personality disorder	Personality disorders

14747	E212z00	Schizoid personality disorder NOS	Personality disorders
21338	Eu60000	[X]Paranoid personality disorder	Personality disorders
28227	E21..11	Neurotic personality disorder	Personality disorders
25146	E21y.00	Other personality disorders	Personality disorders
27803	E21y400	Eccentric personality disorder	Personality disorders
26839	Eu34011	[X]Affective personality disorder	Personality disorders
93492	Eu06011	[X]Organic pseudopsychopathic personality	Personality disorders
64838	Eu6y.00	[X]Other disorders of adult personality and behaviour	Personality disorders
40057	E214.11	Anancastic personality	Personality disorders
52465	Eu60500	[X]Anankastic personality disorder	Personality disorders
35642	E21y100	Avoidant personality disorder	Personality disorders
7745	Eu60300	[X]Emotionally unstable personality disorder	Personality disorders
17420	Eu60513	[X]Obsessive-compulsive personality disorder	Personality disorders
14979	E211.00	Affective personality disorder	Personality disorders
792	E21z.11	Psychopathic personality	Personality disorders
30395	E214000	Anankastic personality	Personality disorders
45188	Eu60215	[X]Sociopathic personality disorder	Personality disorders
58693	Eu60313	[X]Explosive personality disorder	Personality disorders
35763	E21y300	Passive-aggressive personality disorder	Personality disorders
15098	E21z.00	Personality disorder NOS	Personality disorders
34456	E214z00	Compulsive personality disorder NOS	Personality disorders
21005	Eu6..00	[X]Disorders of adult personality and behaviour	Personality disorders
27945	Eu60411	[X]Hysterical personality disorder	Personality disorders
50348	Eu60y16	[X]Psychoneurotic personality disorder	Personality disorders
59008	Eu60714	[X]Self defeating personality disorder	Personality disorders
20839	Eu60311	[X]Aggressive personality disorder	Personality disorders
12228	E211100	Hypomanic personality disorder	Personality disorders
56502	Eu60213	[X]Asocial personality disorder	Personality disorders
4759	E215.11	Hysterical personality disorders	Personality disorders
16178	E211000	Unspecified affective personality disorder	Personality disorders
38100	Eu60511	[X]Compulsive personality disorder	Personality disorders
22259	Eu60512	[X]Obsessional personality disorder	Personality disorders
39777	Eu6z.00	[X]Unspecified disorder of adult personality and behaviour	Personality disorders
51375	Eu84511	[X]Autistic psychopathy	Personality disorders
31819	Eu60700	[X]Dependent personality disorder	Personality disorders
3369	E212.00	Schizoid personality disorder	Personality disorders
22103	E02y400	Drug-induced personality disorder	Personality disorders
112875	Eu60y15	[X]Passive-aggressive personality disorder	Personality disorders
111667	Eu60011	[X]Expansive paranoid personality disorder	Personality disorders
105029	Eu60211	[X]Amoral personality disorder	Personality disorders

CPRD Aurum: Mental ill-health (other than autism)			
**medcode**	**term**	**snomedconceptID**	**interpretation**
853201000006116	Obsessional compulsive psychosis	853201000006100	Anxiety disorders
1755931000006116	Chronic post-traumatic stress disorder following military combat	699241002	Anxiety disorders
1755921000006119	Acute posttraumatic stress disorder following military combat	446175003	Anxiety disorders
295016017	Obsessional neurosis	191738003	Anxiety disorders
403931000006116	Organic anxiety disorder	17496003	Anxiety disorders
400341000006118	[X]Obsessive-compulsive neurosis	191736004	Anxiety disorders
269821000006113	Obsessive-compulsive disorder	191736004	Anxiety disorders
5024071000006118	Anxiety depression	231504006	Anxiety disorders
423021000006114	Post-traumatic stress disorder	47505003	Anxiety disorders
1210253015	Panic disorder	371631005	Anxiety disorders
296271019	[X]Other reactions to severe stress	271952001	Anxiety disorders
295011010	Anancastic neurosis	191736004	Anxiety disorders
1755941000006114	Delayed posttraumatic stress disorder following military combat	446180007	Anxiety disorders
296251011	[X]Predominantly obsessional thoughts or ruminations	67698009	Anxiety disorders
2848311000006117	GAD - Generalised anxiety disorder	21897009	Anxiety disorders
295017014	Obsessive-compulsive disorder NOS	191736004	Anxiety disorders
401881014	[X]Other specified anxiety disorders	197480006	Anxiety disorders
296245018	[X]Other mixed anxiety disorders	231504006	Anxiety disorders
296253014	[X]Mixed obsessional thoughts and acts	191736004	Anxiety disorders
481154010	Generalised anxiety disorder	21897009	Anxiety disorders
6358151000006115	Episodic paroxysmal anxiety disorder	371631005	Anxiety disorders

2848301000006115	GAD - Generalized anxiety disorder	21897009	Anxiety disorders
488201000006114	Anxiety disorder	197480006	Anxiety disorders
7591941000006119	GAD-7 - Generalised Anxiety Disorder 7	704501007	Anxiety disorders
296238018	[X]Other anxiety disorders	197480006	Anxiety disorders
296255019	[X]Obsessive-compulsive disorder, unspecified	191736004	Anxiety disorders
296254015	[X]Other obsessive-compulsive disorders	191736004	Anxiety disorders
294960010	Chronic anxiety	191708009	Anxiety disorders
295015018	Compulsive neurosis	191737008	Anxiety disorders
2848291000006116	Generalized anxiety disorder	21897009	Anxiety disorders
371231000006113	[X]Combat fatigue	67195008	Anxiety disorders
294961014	Recurrent anxiety	191709001	Anxiety disorders
3142901000006117	Cannabis-induced anxiety disorder	39951001	Anxiety disorders
363651000006111	Anxiety neurosis	207363009	Anxiety disorders
2907991000006110	Social anxiety disorder	25501002	Anxiety disorders
400321000006113	[X]Obsessive - compulsive disorder	191736004	Anxiety disorders
363641000006114	[X]Anxiety hysteria	197480006	Anxiety disorders
296249012	[X]Anxiety disorder, unspecified	197480006	Anxiety disorders
401783019	Neurotic disorder NOS	111475002	Anxiety disorders
296239014	[X]Panic disorder [episodic paroxysmal anxiety]	371631005	Anxiety disorders
388071000006116	[X]Generalized anxiety disorder	21897009	Anxiety disorders
12718211000006116	[X]Disturbance of activity and attention	464511000000105	Attention deficit hyperactivity disorders
4998371000006119	Disorders of attention and motor control	229712006	Attention deficit hyperactivity disorders
914491000006114	Deficits in attention motor control and perception	229715008	Attention deficit hyperactivity disorders
295622013	Hyperkinesis with developmental delay	192131001	Attention deficit hyperactivity disorders
4998391000006118	DAMP - Deficits in attention motor control and perception	229715008	Attention deficit hyperactivity disorders
493917012	Hyperactive behaviour	44548000	Attention deficit hyperactivity disorders
296687014	[X]Disturbance of activity and attention	464511000000105	Attention deficit hyperactivity disorders
513731000006112	Bipolar affect disord, now depressed, severe, no psychosis	61403008	Bipolar disorders
294818012	Recurrent manic episode NOS	191590005	Bipolar disorders
367121000006119	[X]Bipolar affective disorder, current episode mixed	192362008	Bipolar disorders
294877011	Mixed bipolar affective disorder, NOS	191636007	Bipolar disorders
294874016	Mixed bipolar affective disorder, severe, with psychosis	191641004	Bipolar disorders
513871000006118	Bipolar disorder	13746004	Bipolar disorders
396801000006115	Manic-depressive psychosis	13746004	Bipolar disorders
294895014	Mixed bipolar I disorder	16506000	Bipolar disorders
294893019	Atypical manic disorder	191658009	Bipolar disorders
398541000006116	Bipolar affective disorder , current episode mixed	192362008	Bipolar disorders
294871012	Mixed bipolar affective disorder, mild	191638008	Bipolar disorders
294860011	Manic-depressive - now depressed	191627008	Bipolar disorders
396781000006119	[X]Manic-depress psychosis,depressed type+psychotic symptoms	765176007	Bipolar disorders
513751000006117	Bipolar affective disorder, current episode depression	191627008	Bipolar disorders
294849019	Bipolar affective disorder, currently manic, mild	191620005	Bipolar disorders
401879012	[X]Unspecified mood affective disorder	46206005	Bipolar disorders
701071000006111	Mixed bipolar affective disorder, severe	764591000000108	Bipolar disorders
367151000006111	[X]Bipolar disorder, single manic episode	268619003	Bipolar disorders
294862015	Bipolar affective disorder, currently depressed, mild	191629006	Bipolar disorders
401865016	[X]Other bipolar affective disorders	13746004	Bipolar disorders
3468391000006115	Severe bipolar I disorder, most recent episode depressed with psychotic features	59617007	Bipolar disorders
396741000006113	[X]Manic episode	268619003	Bipolar disorders
2730071000000117	Mania with mood-incongruent psychotic features	1089671000000106	Bipolar disorders
367071000006119	Bipolar affective disorder, currently manic, severe, with psychosis	191623007	Bipolar disorders
294858014	Bipolar affective disorder, currently manic, NOS	191618007	Bipolar disorders
396711000006114	[X]Mania with mood-incongruent psychotic symptoms	231494001	Bipolar disorders
882311000006110	Manic-depressive psychoses	13746004	Bipolar disorders
367111000006110	Bipolar affective disorder, current episode hypomanic	31446002	Bipolar disorders
294802018	Single manic episode, unspecified	268619003	Bipolar disorders

294863013	Bipolar affective disorder, currently depressed, moderate	191630001	Bipolar disorders
1785871000006117	[X]Bipolar II disorder	83225003	Bipolar disorders
294882016	Mild bipolar disorder	13313007	Bipolar disorders
367081000006116	[X]Bipolar affect disorder cur epi manic wout psychotic symp	191618007	Bipolar disorders
82171000006116	Severe bipolar disorder with psychotic features	4441000	Bipolar disorders
294896010	Other and unspecified manic-depressive psychoses NOS	13746004	Bipolar disorders
401863011	[X]Mania with psychotic symptoms	231494001	Bipolar disorders
367161000006113	[X]Bipolar II disorder	83225003	Bipolar disorders
401765010	Manic disorder, single episode	268619003	Bipolar disorders
296118012	[X]Other manic episodes	231494001	Bipolar disorders
294811018	Recurrent manic episodes, unspecified	191590005	Bipolar disorders
362781000006116	[X]Affective psychosis NOS	46206005	Bipolar disorders
401878016	[X]Other recurrent mood affective disorders	386782008	Bipolar disorders
401877014	[X]Other single mood affective disorders	46206005	Bipolar disorders
513801000006112	Bipolar affective disorder, current episode manic	191618007	Bipolar disorders
296208012	[X]Persistent mood affective disorder, unspecified	386782008	Bipolar disorders
424661000006111	[X]Recurrent manic episodes	191590005	Bipolar disorders
294861010	Bipolar affective disorder, currently depressed, unspecified	191627008	Bipolar disorders
513721000006114	Bipolar affect disord, now depressed, severe with psychosis	765176007	Bipolar disorders
294812013	Recurrent manic episodes, mild	191592002	Bipolar disorders
294809010	Manic disorder, single episode NOS	268619003	Bipolar disorders
296110017	Mania	231494001	Bipolar disorders
5023451000006115	Organic bipolar disorder	231444002	Bipolar disorders
389401000006111	Hypomania	231496004	Bipolar disorders
294848010	Bipolar affective disorder, currently manic, unspecified	191618007	Bipolar disorders
13716031000006110	Bipolar disorder caused by drug	16238741000119104	Bipolar disorders
294872017	Mixed bipolar affective disorder, moderate	191639000	Bipolar disorders
296135011	[X]Bipolar affective disorder, unspecified	13746004	Bipolar disorders
396691000006111	[X]Mania NOS	268619003	Bipolar disorders
294815010	Recurrent manic episodes, severe, with psychosis	191595000	Bipolar disorders
294880012	Unspecified bipolar affective disorder	13746004	Bipolar disorders
294813015	Recurrent manic episodes, moderate	191593007	Bipolar disorders
294868016	Bipolar affective disorder, currently depressed, NOS	191627008	Bipolar disorders
789221000006116	Hypomanic psychoses	231496004	Bipolar disorders
513691000006116	Severe manic bipolar I disorder without psychotic features	162004	Bipolar disorders
294870013	Mixed bipolar affective disorder, unspecified	191636007	Bipolar disorders
12059211000006120	Bipolar disorder, most recent episode manic	767632000	Bipolar disorders
367061000006114	Psychosis and severe depression co-occurrent and due to bipolar affective disorder	765176007	Bipolar disorders
82151000006114	Severe bipolar disorder without psychotic features	53049002	Bipolar disorders
294810017	Recurrent manic episodes	191590005	Bipolar disorders
294891017	Other and unspecified manic-depressive psychoses	13746004	Bipolar disorders
223601000000119	Manic psychosis	231494001	Bipolar disorders
367051000006112	Severe depressed bipolar I disorder without psychotic features	61403008	Bipolar disorders
296207019	[X]Other persistent mood affective disorders	386782008	Bipolar disorders
294804017	Single manic episode, moderate	191584006	Bipolar disorders
367101000006112	Bipolar affective disorder	13746004	Bipolar disorders
294850019	Bipolar affective disorder, currently manic, moderate	191621009	Bipolar disorders
294803011	Single manic episode, mild	191583000	Bipolar disorders
2730091000000118	Mania with psychotic features	1089681000000108	Bipolar disorders
294847017	Manic-depressive - now manic	191618007	Bipolar disorders
12059251000006118	Bipolar I disorder, most recent episode depression	767636002	Bipolar disorders
396701000006111	[X]Mania with mood-congruent psychotic symptoms	231494001	Bipolar disorders
294888017	Unspecified bipolar affective disorder, NOS	13746004	Bipolar disorders
396791000006116	Manic-depressive illness	13746004	Bipolar disorders
294881011	Unspecified bipolar affective disorder, unspecified	13746004	Bipolar disorders
513701000006116	Bipolar affect disord, currently manic,severe with psychosis	191623007	Bipolar disorders
296209016	[X]Other mood affective disorders	46206005	Bipolar disorders
2960491000006119	Severe manic bipolar I disorder with psychotic features	28663008	Bipolar disorders

367091000006118	[X]Bipolar affect disorder cur epi mild or moderate depressn	191630001	Bipolar disorders
396771000006117	[X] Manic-depressive psychosis, depressed type without psychotic symptoms	36474008	Bipolar disorders
182871000006115	Recurrent manic episodes, severe	764621000000106	Bipolar disorders
296218019	[X]Other specified mood affective disorders	46206005	Bipolar disorders
294892012	Unspecified manic-depressive psychoses	13746004	Bipolar disorders
2730051000000114	Mania with mood-congruent psychotic features	1089661000000104	Bipolar disorders
2157096015	Manic mood	405273008	Bipolar disorders
294883014	Moderate bipolar disorder	79584002	Bipolar disorders
12059201000006116	Bipolar disorder, most recent episode depression	767631007	Bipolar disorders
401864017	[X]Manic episode, unspecified	268619003	Bipolar disorders
294869012	Mixed bipolar affective disorder	191636007	Bipolar disorders
1975981000006114	Mild depressive episode, without somatic syndrome	1975981000006105	Depression
5106071000006112	Mild postnatal psychosis	237351003	Depression
13974511000006116	Episode of depression	871840004	Depression
346973011	Masked depression	231500002	Depression
423141000006115	[X]Postpartum depression NOS	58703003	Depression
473201000006114	Mood disorder	46206005	Depression
294844012	Recurrent depression	191616006	Depression
294824018	Single major depressive episode, unspecified	36923009	Depression
3071801000006112	Depressed	35489007	Depression
2540711000006115	Chronic recurrent major depressive disorder	2618002	Depression
2164006016	Depressed	366979004	Depression
294825017	Mild major depression, single episode	79298009	Depression
14627061000006116	Depressive disorder caused by drug	191495003	Depression
13716011000006116	Depressive disorder caused by amphetamine	16238181000119100	Depression
1823881000006110	Depression confirmed	1823881000006106	Depression
5359021000006116	Depression	255339005	Depression
2164005017	Depressed mood	366979004	Depression
2724681000006112	Chronic major depressive disorder, single episode	14183003	Depression
294840015	Recurrent major depressive episodes, severe, with psychosis	191613003	Depression
3087971000006112	Severe recurrent major depression without psychotic features	36474008	Depression
3767531000006119	Severe major depression, single episode, with psychotic features, mood-congruent	77911002	Depression
14452261000006112	Persistent depressive disorder	1153575004	Depression
294826016	Moderate major depression, single episode	15639000	Depression
1976231000006110	Recurrent depressive disorder, current episode mild, with somatic syndrome	1976231000006106	Depression
5247381000006117	Depressive delusion of catastrophe	247686005	Depression
7991681000006114	Perinatal depression	10211000132109	Depression
294918011	Psychotic reactive depression	191676002	Depression
1976021000006115	Moderate depressive episode, without somatic syndrome	1976021000006104	Depression
12127541000006116	Recurrent severe major depressive disorder co-occurrent with anxiety	16264821000119108	Depression
9901161000006120	Mild major depressive disorder co-occurrent with anxiety single episode	16265951000119108	Depression
2798691000006112	Moderate recurrent major depression	18818009	Depression
424671000006116	[X]Recurrent severe episodes of psychotic depression	191613003	Depression
3743451000006110	Severe major depression, single episode, without psychotic features	76441001	Depression
410861011	Endogenous depression - recurrent	274948002	Depression
424561000006110	[X]Recurrent brief depressive episodes	40568001	Depression
295537016	Chronic depression	192080009	Depression
346972018	Endogenous depression first episode	231499006	Depression
401873013	[X]Recurrent depressive disorder	191616006	Depression
474171000006112	Agitated depression	83458005	Depression
294894013	Atypical depressive disorder	191659001	Depression
401766011	Major depression, single episode	36923009	Depression
376701000006116	[X]Depressive conduct disorder	231542000	Depression
8465321000006112	Reactive depression, prolonged single episode	1086661000000108	Depression
1715771000006112	Mild major depression	87512008	Depression
396081000006116	[X]Major depression, recurrent without psychotic symptoms	268621008	Depression
882821000006110	Moderate depression	465441000000108	Depression

426991000006118	[X]Single episode vital depression w'out psychotic symptoms	310497006	Depression
424551000006113	[X]Recurr severe episodes/psychogenic depressive psychosis	191613003	Depression
399961000006118	Depressive neurosis	78667006	Depression
432511000006119	[X]Vital depression, recurrent without psychotic symptoms	310497006	Depression
1976211000006116	Recurrent depressive disorder, current episode mild, without somatic syndrome	1976211000006100	Depression
401866015	[X]Severe depressive episode without psychotic symptoms	310497006	Depression
3094951000006111	Major depressive disorder, single episode	36923009	Depression
426921000006115	[X]Single episode of major depression and psychotic symptoms	191604000	Depression
401872015	[X]Depressive episode, unspecified	35489007	Depression
882681000006110	Depression NOS	609311000000100	Depression
2620391000000111	Maternal postnatal depression	1038261000000100	Depression
426931000006117	[X]Single episode of masked depression NOS	231500002	Depression
1972111000006114	Organic depressive disorder	1972111000006105	Depression
401876017	[X]Recurrent depressive disorder, unspecified	191616006	Depression
423611000006111	[X]Prolonged single episode of reactive depression	87414006	Depression
1494612017	Depressive symptoms	394924000	Depression
138421012	Agitated depression	83458005	Depression
882401000006115	Reactive (neurotic) depression	87414006	Depression
7803711000006118	Moderately severe major depression single episode	720453001	Depression
1488626018	Symptoms of depression	394924000	Depression
12127551000006118	Recurrent moderate major depressive disorder co-occurrent with anxiety	16264901000119108	Depression
1976271000006113	Recurrent depressive disorder, current episode moderate, with somatic syndrome	1976271000006109	Depression
376721000006114	Depressive episode	35489007	Depression
2914841000006115	Major depressive disorder, single episode with postpartum onset	25922000	Depression
426941000006110	[X]Single episode of psychogenic depression	87414006	Depression
379781000006118	[X]Endogenous depression without psychotic symptoms	300706003	Depression
253619019	O/E - depressed	162722001	Depression
1715191000006112	Severe major depression with psychotic features	73867007	Depression
2729991000000112	Recurrent depression with current severe episode without psychotic features	1089631000000109	Depression
426951000006112	[X]Single episode of psychogenic depressive psychosis	191676002	Depression
398841000006119	[X]Monopolar depression NOS	35489007	Depression
5248911000006110	Depression worse in morning	247801000	Depression
7695931000006118	Acute depression	712823008	Depression
1715181000006114	Moderate major depression	832007	Depression
7127661000006118	Major depressive disorder, single episode, severe with psychotic features	430852001	Depression
424531000006118	[X]Recurr depress disorder cur epi severe without psyc sympt	310497006	Depression
294917018	Reactive depressive psychosis	191676002	Depression
1715781000006110	Severe major depression without psychotic features	75084000	Depression
1231868010	Puerperal depression	58703003	Depression
6337501000006112	Major depression	370143000	Depression
424541000006111	[X]Recurr severe episodes/major depression+psychotic symptom	28475009	Depression
419841000006116	[X]Persistant anxiety depression	231504006	Depression
7965191000006118	Severe recurrent major depression	281000119103	Depression
3628471000006116	Major depressive disorder, single episode with catatonic features	69392006	Depression
7882721000006118	Major depression with psychotic features	726772006	Depression
882671000006112	Depression	609311000000100	Depression
5106051000006119	Mild postnatal depression	237349002	Depression
398561000006117	[X]Mixed anxiety and depressive disorder	231504006	Depression
14013791000006120	Depressive disorder caused by methamphetamine	16238221000119108	Depression
441826016	Endogenous depression	300706003	Depression
5106061000006117	Severe postnatal depression	237350002	Depression
294845013	Recurrent major depressive episode NOS	268621008	Depression
2730011000000110	Recurrent depression with current moderate episode	1089641000000100	Depression

1976251000006115	Recurrent depressive disorder, current episode moderate, without somatic syndrome	1976251000006104	Depression
8465361000006118	Reactive depression, first episode	1086691000000102	Depression
642461000006116	Endogenous depression first episode	231499006	Depression
613791000006115	Depressive psychoses	35489007	Depression
1785881000006119	Antenatal depression	790961000000101	Depression
296180012	[X]Recurrent depressive disorder, current episode mild	310495003	Depression
294836012	Recurrent major depressive episodes, unspecified	268621008	Depression
882421000006113	Depressive personality	78667006	Depression
401869010	[X]Severe depressive episode with psychotic symptoms	191604000	Depression
296199015	[X]Other recurrent depressive disorders	191616006	Depression
13902421000006110	Antenatal depression	788120007	Depression
488211000006112	Mixed anxiety and depressive disorder	231504006	Depression
379771000006116	[X]Endogenous depression with psychotic symptoms	73867007	Depression
5024511000006118	Depressive conduct disorder	231542000	Depression
376291000006118	Cyclothymia	76105009	Depression
3140411000006112	Recurrent major depressive disorder with catatonic features	39809009	Depression
3578851000006118	Recurrent major depression	66344007	Depression
294832014	Single major depressive episode NOS	36923009	Depression
376711000006118	[X]Depressive disorder NOS	35489007	Depression
182721000006111	Recurrent major depressive episodes	268621008	Depression
9901261000006114	Moderate major depressive disorder co-occurrent with anxiety single episode	16266831000119100	Depression
182771000006112	Recurrent major depressive episodes, severe	764611000000100	Depression
366561000006119	[X]Atypical depression	191659001	Depression
9901281000006116	Severe major depressive disorder co-occurrent with anxiety single episode	16266991000119108	Depression
7127651000006115	Severe major depression, single episode, with psychotic features	430852001	Depression
3578861000006116	Recurrent major depressive disorder	66344007	Depression
424641000006112	[X]Recurrent episodes of psychogenic depression	191616006	Depression
882811000006119	Mild depression	430421000000104	Depression
426891000006114	[X] Single episode agitated depression without psychotic symptoms	310497006	Depression
3286331000006115	Minor depressive disorder	48589009	Depression
376741000006119	[X]Depressive neurosis	78667006	Depression
1975991000006112	Mild depressive episode, with somatic syndrome	1975991000006108	Depression
1976051000006112	Moderate depressive episode, with somatic syndrome	1976051000006108	Depression
7803731000006112	Minimal major depression single episode	720454007	Depression
3042901000006110	Severe major depression with psychotic features, mood-congruent	33736005	Depression
426971000006119	[X]Single episode of reactive depression	87414006	Depression
296181011	[X]Recurrent depressive disorder, current episode moderate	310496002	Depression
296137015	Mild depression	310495003	Depression
5248931000006116	Depression worse later in day	247802007	Depression
13974501000006116	Depressive episode	871840004	Depression
295535012	Depressive disorder	35489007	Depression
359121000006116	Reactive depression	87414006	Depression
3071791000006111	Depressive illness	35489007	Depression
425751000006115	[X]Seasonal depressive disorder	247803002	Depression
424651000006114	[X]Recurrent episodes of reactive depression	191616006	Depression
426961000006114	[X]Single episode of psychotic depression	191604000	Depression
424571000006115	[X]Recurrent depress disorder cur epi severe with psyc symp	28475009	Depression
3153071000006116	Recurrent brief depressive disorder	40568001	Depression
401871010	[X]Other depressive episodes	35489007	Depression
295536013	Postviral depression	192079006	Depression
213641000000111	[X]Mild depression	310495003	Depression
294828015	Single major depressive episode, severe, with psychosis	191604000	Depression
6337491000006116	Major depressive disorder	370143000	Depression
3149661000006117	Mild recurrent major depression	40379007	Depression
2957311000006113	Severe recurrent major depression with psychotic features	28475009	Depression
398351000006110	[X]Mild anxiety depression	231504006	Depression

142541000006115	Severe major depression, single episode	251000119105	Depression
12127531000006112	Recurrent mild major depressive disorder co-occurrent with anxiety	16264621000119108	Depression
223741000000112	Severe depression	310497006	Depression
7382291000006112	Severe major depression	450714000	Depression
296138013	[X]Moderate depressive episode	310496002	Depression
294838013	Recurrent major depressive episodes, moderate	191611001	Depression
294837015	Recurrent major depressive episodes, mild	191610000	Depression
398851000006117	Affective disorder	46206005	Depression
2729751000000118	Recurrent depression with current severe episode and psychotic features	1089511000000100	Depression
882831000006113	Severe depression	397701000000102	Depression
223651000000118	Postpartum depression	58703003	Depression
853871000006111	Post natal depression	853871000006107	Depression
7788661000006119	Moderately severe depression	719593009	Depression
426981000006116	[X]Single episode of reactive depressive psychosis	191676002	Depression
376691000006116	Depression	35489007	Depression
6000711000006112	Moderate depression	310496002	Depression
423131000006113	[X]Postnatal depression NOS	58703003	Depression
3733211000006112	Mood disorder, condition with depressive features	75837004	Depression
675861000006113	Reactive depression (situational)	87414006	Depression
94597012	Anorexia nervosa	56882008	Eating disorders
401889011	[X]Other eating disorders	72366004	Eating disorders
368061000006113	[X]Bulimia NOS	78004001	Eating disorders
3799251000006119	Anorexia	79890006	Eating disorders
334547013	[X]Starvation	217640002	Eating disorders
2159265012	Nocturnal sleep-related eating disorder	407666007	Eating disorders
3423851000006118	AN - Anorexia nervosa	56882008	Eating disorders
1222496015	Bulimia	78004001	Eating disorders
882611000006115	Eating disorder NOS - psychog.	608241000000102	Eating disorders
317235018	[D]Anorexia NOS	79890006	Eating disorders
368051000006111	Bulimia nervosa	78004001	Eating disorders
295451018	Other specified non-organic eating disorder	72366004	Eating disorders
1620911000006112	Eating disorders	1620911000006108	Eating disorders
424061000006117	[X]Psychogenic overeating	270902002	Eating disorders
830401000006110	Hyperalimentation - symptom	212572003	Eating disorders
324685013	Starvation	212968006	Eating disorders
363321000006111	[X]Anorexia nervosa	56882008	Eating disorders
7230101000006110	Binge eating disorder	439960005	Eating disorders
389311000006116	[X]Hyperorexia nervosa	78004001	Eating disorders
296379015	[X]Eating disorder, unspecified	72366004	Eating disorders
317258015	[D]Hyperalimentation	27029007	Eating disorders
317233013	[D]Anorexia	496871000000107	Eating disorders
3763801000006119	Anorexia nervosa, restricting subtype	77675002	Eating disorders
8440231000006115	Eating disorder co-occurrent with diabetes mellitus type 1	1067201000000106	Eating disorders
988931000006112	Eating disorder NOS - psychog.	608251000000104	Eating disorders
417851000006113	Overeating associated with other psychological disturbances	270902002	Eating disorders
527421000006116	Bulimia (non-organic overeating)	78004001	Eating disorders
420741000006110	[X]Pica in adults	14077003	Eating disorders
960281000006115	Anorexia	960281000006104	Eating disorders
3799261000006117	Anorexic	79890006	Eating disorders
397863019	Excessive eating - polyphagia	267023007	Eating disorders
317255017	[D]Polyphagia	267023007	Eating disorders
295452013	Non-organic eating disorder NOS	72366004	Eating disorders
317259011	[D]Polyphagia NOS	267023007	Eating disorders
366521000006113	Atypical anorexia nervosa	231522009	Eating disorders
296361019	Eating disorder	72366004	Eating disorders
317257013	[D]Excessive eating	267023007	Eating disorders
366541000006118	Atypical bulimia nervosa	231523004	Eating disorders
372208017	Anorexia symptom	249468005	Eating disorders
577901000006119	Compulsive eating disorder	78004001	Eating disorders
407104010	Polyphagia symptom	272059005	Eating disorders
404431014	Appetite loss - anorexia	249468005	Eating disorders
12717671000006110	[D]Anorexia	496871000000107	Eating disorders
295437018	Unspecified non-organic eating disorder	72366004	Eating disorders

295436010	Other and unspecified non-organic eating disorders	72366004	Eating disorders
424331000006112	[X]Querulant personality disorder	13601005	Personality disorders
295080016	Immature personality disorder	191773001	Personality disorders
295081017	Masochistic personality disorder	191774007	Personality disorders
425591000006118	[X]Schizoid personality disorder	52954000	Personality disorders
605901000006114	Cyclothymic personality disorder	76105009	Personality disorders
2567501000006116	Cluster B personality disorder	4306003	Personality disorders
33677019	Borderline personality disorder	20010003	Personality disorders
5506461000006116	Introverted personality disorder	268633003	Personality disorders
425911000006118	[X]Sensitive paranoid personality disorder	13601005	Personality disorders
2520381000006114	Anancastic personality disorder	1376001	Personality disorders
295048018	Affective personality disorder NOS	76105009	Personality disorders
2927101000006111	Sociopathic personality disorder	26665006	Personality disorders
362771000006119	[X]Affective personality disorder	76105009	Personality disorders
296488011	[X]Personality disorder, unspecified	33449004	Personality disorders
418531000006117	[X]Passive personality disorder	84466009	Personality disorders
389491000006116	[X]Immature personality disorder	33449004	Personality disorders
423741000006116	[X]Pseudopsychopathic schizophrenia	31027006	Personality disorders
230091000006118	Personality disorder	33449004	Personality disorders
62962010	Avoidant personality disorder	37746008	Personality disorders
404021000006116	Organic personality disorder	36217008	Personality disorders
363691000006117	Anxious personality disorder	231528008	Personality disorders
379511000006118	[X]Eccentric personality disorder	33449004	Personality disorders
295064014	Unspecified histrionic personality disorder	55341008	Personality disorders
401898014	[X]Other specific personality disorders	33449004	Personality disorders
3398571000006119	Hysterical personality disorder	55341008	Personality disorders
388871000006111	[X]Histrionic personality disorder	55341008	Personality disorders
1495439012	Affective personality disorder	76105009	Personality disorders
481481000006110	Anankastic personality	1376001	Personality disorders
386711000006110	[X]Fanatic paranoid personality disorder	13601005	Personality disorders
295079019	Eccentric personality disorder	191772006	Personality disorders
577931000006110	Compulsive personality disorder	473456000	Personality disorders
401785014	Psychoneurotic personality disorder	268634009	Personality disorders
362761000006114	[X]Affectionless psychopathy	192616009	Personality disorders
5024351000006113	Impulsive personality disorder	231527003	Personality disorders
425841000006112	[X]Self defeating personality disorder	84466009	Personality disorders
295077017	Other personality disorders	33449004	Personality disorders
363081000006112	[X]Amoral personality disorder	26665006	Personality disorders
362851000006118	[X]Aggressive personality disorder	191765005	Personality disorders
295063015	Compulsive personality disorder NOS	473456000	Personality disorders
88108012	Schizoid personality disorder	52954000	Personality disorders
414781000006118	[X]Other specified disorders of adult personality and behaviour	943161000000107	Personality disorders
294691019	Drug-induced personality disorder	191496002	Personality disorders
782871000006113	Inadequate personality disorder	84466009	Personality disorders
378071000006112	Dissocial personality disorder	26665006	Personality disorders
23211011	Paranoid personality disorder	13601005	Personality disorders
347011014	Explosive personality disorder	231527003	Personality disorders
401786010	Other personality disorder NOS	33449004	Personality disorders
613781000006118	Depressive personality disorder	78667006	Personality disorders
418541000006110	[X]Passive-aggressive personality disorder	33449004	Personality disorders
3225431000006114	Passive aggressive personality disorder	44966003	Personality disorders
367941000006111	[X]Borderline personality disorder	20010003	Personality disorders
376751000006117	Depressive personality disorder	1084061000000106	Personality disorders
389581000006115	[X]Inadequate personality disorder	84466009	Personality disorders
1230168015	Passive-aggressive personality disorder	44966003	Personality disorders
295044016	Affective personality disorder	76105009	Personality disorders
296489015	[X]Mixed and other personality disorders	33449004	Personality disorders
427091000006119	[X]Sociopathic personality disorder	26665006	Personality disorders
424201000006112	[X]Psychoneurotic personality disorder	268634009	Personality disorders
389441000006113	[X]Hysterical personality disorder	55341008	Personality disorders
824751000006111	Histrionic personality disorder	55341008	Personality disorders
5024321000006116	Manipulative personality disorder	231525006	Personality disorders
363221000006116	Anankastic personality disorder	1376001	Personality disorders
1976641000006119	Mixed personality disorders	1976641000006103	Personality disorders
295049014	Unspecified schizoid personality disorder	52954000	Personality disorders

424211000006110	[X]Psychopathic personality disorder	26665006	Personality disorders
487991000006113	Antisocial or sociopathic personality disorder	26665006	Personality disorders
2927081000006115	Psychopathic personality disorder	26665006	Personality disorders
296549011	[X]Unspecified disorder of adult personality and behaviour	943161000000107	Personality disorders
481471000006112	Anancastic personality	1376001	Personality disorders
295045015	Hypomanic personality disorder	191753006	Personality disorders
296543012	[X]Other disorders of adult personality and behaviour	943161000000107	Personality disorders
7488401000006113	Obsessional personality disorder	473457009	Personality disorders
787571000006113	Hysterical personality disorders	55341008	Personality disorders
295067019	Histrionic personality disorder NOS	55341008	Personality disorders
133885012	Narcissistic personality disorder	80711002	Personality disorders
363921000006112	[X]Asocial personality disorder	26665006	Personality disorders
295052018	Schizoid personality disorder NOS	52954000	Personality disorders
425731000006110	[X]Schizotypal personality disorder	31027006	Personality disorders
199821000006114	Psychopathic personality	26665006	Personality disorders
388391000006117	[X]Haltlose type personality disorder	33449004	Personality disorders
296460017	[X]Specific personality disorders	33449004	Personality disorders
366591000006110	[X]Autistic psychopathy	23560001	Personality disorders
2520371000006111	Obsessive compulsive personality disorder	1376001	Personality disorders
3009221000006112	Multiple personality disorder	31611000	Personality disorders
404031000006118	[X]Organic pseudopsychopathic personality	36217008	Personality disorders
400351000006116	[X]Obsessive-compulsive personality disorder	1376001	Personality disorders
400311000006117	[X]Obsessional personality disorder	473457009	Personality disorders
399451000006114	[X]Narcissistic personality disorder	80711002	Personality disorders
675911000006115	Neurotic personality disorder	33449004	Personality disorders
380611000006117	[X]Explosive personality disorder	191765005	Personality disorders
380231000006114	[X]Expansive paranoid personality disorder	13601005	Personality disorders
371341000006110	[X]Compulsive personality disorder	1376001	Personality disorders
366421000006114	[X]Asthenic personality disorder	84466009	Personality disorders
418201000006111	[X]Paranoid personality disorder	13601005	Personality disorders
296458019	[X]Disorders of adult personality and behaviour	943161000000107	Personality disorders
401787018	Personality disorder NOS	33449004	Personality disorders
363561000006112	Antisocial personality disorder	26665006	Personality disorders
376671000006117	Dependent personality disorder	84466009	Personaliy disorders
294897018	Affective psychosis	441704009	Psychotic disorders
418261000006112	[X]Paraphrenic schizophrenia	64905009	Psychotic disorders
4763211000006118	Drug-induced psychosis	191483003	Psychotic disorders
346896012	Reactive psychoses	231437006	Psychotic disorders
362391000006117	Acute polymorphic psychotic disorder without symptoms of schizophrenia	712824002	Psychotic disorders
294902017	Other affective psychosis NOS	441704009	Psychotic disorders
1975211000006110	Post-schizophrenic depression, episodic with progressive deficit	1975211000006106	Psychotic disorders
14798821000006116	Psychosis caused by ethanol	1231846001	Psychotic disorders
28758018	Subchronic schizophrenia	16990005	Psychotic disorders
428441000006116	[X]Symbiotic psychosis	35919005	Psychotic disorders
155281000006119	Schizotypal personality	31027006	Psychotic disorders
14798841000006110	Alcohol-induced psychosis	1231846001	Psychotic disorders
401855010	[X]Other schizophrenia	58214004	Psychotic disorders
294676016	Alcoholic psychosis NOS	42344001	Psychotic disorders
52897013	Chronic paranoid schizophrenia	31658008	Psychotic disorders
389661000006114	[X]Induced psychotic disorder	61831009	Psychotic disorders
367951000006113	Borderline schizophrenia	274952002	Psychotic disorders
1972921000006115	Mental & behav dis due to seds/hypntcs: psychotic disordr, predominantly polymorphic	1972921000006104	Psychotic disorders
294724012	Non-organic psychoses	191525009	Psychotic disorders
1973061000006116	Mental & behav dis due to hallucinogens: psychotic disord, predominantly manic symptoms	1973061000006100	Psychotic disorders
294787015	Coenesthopathic schizophrenia	191577003	Psychotic disorders
294727017	Simple schizophrenia	191527001	Psychotic disorders
1971761000006117	Mental & behav dis due to seds/hypntcs: resid & late-onset psychot dis, late-onset psychotic disorder	1971761000006101	Psychotic disorders
1973001000006117	Mental & behav dis due to hallucinogens: psychotic disord, predominantly polymorphic	1973001000006101	Psychotic disorders
425511000006111	Schizoaffective disorder, manic type	271428004	Psychotic disorders
1971501000006112	Mental & behav dis due to use opioids: psychotic disorder, predominantly delusional	1971501000006108	Psychotic disorders

362221000006114	[X]Acute / subacute infective psychosis	191502008	Psychotic disorders
370981000006112	[X]Chronic hallucinatory psychosis	480111000000107	Psychotic disorders
1973041000006115	Mental & behav dis due to hallucinogens: psychotic disord, predominantly depressive symptoms	1973041000006104	Psychotic disorders
397641000006116	Cocaine-induced psychosis	943101000000108	Psychotic disorders
294742019	Catatonic schizophrenia	191542003	Psychotic disorders
423471000006117	Prodromal schizophrenia	247804008	Psychotic disorders
294731011	Acute exacerbation of chronic schizophrenia	191531007	Psychotic disorders
8342781000006115	Psychoactive substance-induced psychosis	943121000000104	Psychotic disorders
362981000006119	[X]Alcoholic psychosis NOS	42344001	Psychotic disorders
1973611000006112	Mental & behav dis due to use cocaine: psychotic disorder, predominantly delusional	1973611000006108	Psychotic disorders
1972321000006116	Mental & behav dis due to cannabinoids: psychotic disordr, predominantly polymorphic	1972321000006100	Psychotic disorders
215841000000114	Delusional disorder	48500005	Psychotic disorders
318027012	[X]Other hallucinations	7011001	Psychotic disorders
981141000006111	Delusions	981141000006107	Psychotic disorders
317003012	[D]Hallucinations	7011001	Psychotic disorders
1972351000006113	Mental & behav dis due to cannabinoids: psychotic disordr, predominantly manic symptoms	1972351000006109	Psychotic disorders
294693016	Drug psychosis NOS	191483003	Psychotic disorders
882301000006112	Acute schizo affective psychosis	191567000	Psychotic disorders
2790111000006113	Postnatal psychosis	18260003	Psychotic disorders
366571000006114	[X]Atypical schizophrenia	111484002	Psychotic disorders
12480451000006112	[X]Schizophrenia, schizotypal and delusional disorders	417601000000102	Psychotic disorders
492910018	Ideas of reference	41189006	Psychotic disorders
294741014	Hebephrenic schizophrenia NOS	35252006	Psychotic disorders
1972971000006119	Mental & behav dis due to seds/hypntcs: psychotic disordr, mixed	1972971000006103	Psychotic disorders
3504751000006114	Induced psychosis	61831009	Psychotic disorders
424681000006118	Recurrent reactive depressive episodes, severe, with psychosis	1086471000000103	Psychotic disorders
1974761000006115	Mental & behav dis due to use tobacco: psychotic disorder, predominantly depressive symptoms	1974761000006104	Psychotic disorders
425671000006115	[X]Schizophrenic reaction	278853003	Psychotic disorders
2548181016	Paranoid ideation	417233008	Psychotic disorders
425691000006119	[X]Schizophreniform psychosis, depressive type	84760002	Psychotic disorders
294764011	Latent schizophrenia	191559008	Psychotic disorders
10122017	Brief reactive psychosis	5464005	Psychotic disorders
43595011	Residual schizophrenia	26025008	Psychotic disorders
5128631000006110	Cutaneous monosymptomatic delusional psychosis	238972008	Psychotic disorders
425651000006113	[X]Schizophrenic catatonia	191542003	Psychotic disorders
882251000006117	Puerperal psychosis	551591000000100	Psychotic disorders
1975731000006111	Acute schizophrenia-like psychotic disorder, with associated acute stress	1975731000006107	Psychotic disorders
914471000006113	[X]Delusional misidentification syndrome	44906001	Psychotic disorders
8089301000006110	Reactive depressive psychosis, single episode	288751000119101	Psychotic disorders
1976111000006110	Mental & behav dis due to use alcohol: resid & late-onset psychot dis, late-onset psychotic disorder	1976111000006106	Psychotic disorders
7859641000006116	Cocaine-induced psychotic disorder	724689006	Psychotic disorders
1972281000006110	Mental & behav dis due to cannabinoids: psychotic disordr, predominantly delusional	1972281000006106	Psychotic disorders
1972331000006118	Mental & behav dis due to cannabinoids: psychotic disordr, predominantly depressive symptoms	1972331000006102	Psychotic disorders
424071000006112	[X]Psychogenic paranoid psychosis	191680007	Psychotic disorders
630831000006113	Drug psychosis	191483003	Psychotic disorders
418221000006118	[X]Paranoid schizophrenia	64905009	Psychotic disorders
423041000006119	[X]Post-schizophrenic depression	231485007	Psychotic disorders
1971631000006117	Mental & behav dis due to use opioids: psychotic disorder, mixed	1971631000006101	Psychotic disorders
370451000006110	[X]Catatonic schizophrenia	191542003	Psychotic disorders
1972031000006116	Mental & behav dis due to hallucinogens: psychotic disord, predominantly delusional	1972031000006100	Psychotic disorders
1972051000006111	Mental & behav dis due to hallucinogens: psychotic disord, predominantly hallucinatory	1972051000006107	Psychotic disorders
914461000006118	[X]Capgras syndrome	44906001	Psychotic disorders
155141000006116	Schizoaffective schizophrenia	191567000	Psychotic disorders
397661000006117	Opioid-induced psychosis	943071000000104	Psychotic disorders

294757014	Acute exacerbation of subchronic paranoid schizophrenia	191554003	Psychotic disorders
425681000006117	[X]Schizophreniform disord NOS	58214004	Psychotic disorders
424511000006112	[X]Reactive psychosis	231437006	Psychotic disorders
425501000006113	Schizoaffective disorder, depressive type	84760002	Psychotic disorders
1972901000006113	Mental & behav dis due to seds/hypntcs: psychotic disordr, predominantly hallucinatory	1972901000006109	Psychotic disorders
1973581000006116	Mental & behav dis due to use cocaine: psychotic disorder, schizophrenia-like	1973581000006100	Psychotic disorders
1971531000006116	Mental & behav dis due to use opioids: psychotic disorder, predominantly polymorphic	1971531000006100	Psychotic disorders
1975631000006119	Mental and behav dis due to other stimulants inc caffeine: psychotic dis, predominantly polymorphic	1975631000006103	Psychotic disorders
317008015	[D]Visual hallucinations	64269007	Psychotic disorders
369805011	Grandiose delusions	247667002	Psychotic disorders
1976921000006116	Post-schizophrenic depression, other	1976921000006100	Psychotic disorders
1975181000006111	Mental & behav dis due to use alcohol: psychotic disorder, predominantly delusional	1975181000006107	Psychotic disorders
1975821000006118	Other acute and transient psychotic disorders, without associated acute stress	1975821000006102	Psychotic disorders
1234861017	Chronic schizophrenic	83746006	Psychotic disorders
401771016	Non-organic psychosis NOS	191525009	Psychotic disorders
296095012	[X]Other schizoaffective disorders	68890003	Psychotic disorders
78531000006116	Unspecified schizo-affective schizophrenia	191567000	Psychotic disorders
107878010	Paranoid schizophrenia	64905009	Psychotic disorders
401862018	[X]Unspecified nonorganic psychosis	191525009	Psychotic disorders
3700911000006115	Psychotic depression	73867007	Psychotic disorders
317009011	[D]Hallucinations NOS	7011001	Psychotic disorders
418181000006110	[X]Paranoia querulans	231487004	Psychotic disorders
155161000006117	Schizo-affective schizophrenia NOS	191567000	Psychotic disorders
1973081000006114	Mental & behav dis due to vol solvents: psychotic disordr, predominantly hallucinatory	1973081000006105	Psychotic disorders
296040018	[X]Schizophrenia, unspecified	58214004	Psychotic disorders
1974321000006110	Mental and behav dis mlti drg use/oth psych subs: resid/late psychot dis, late-onset psychotic disorder	1974321000006106	Psychotic disorders
294721016	Chronic organic psychosis NOS	191447007	Psychotic disorders
2730111000000111	Acute predominantly delusional psychotic disorder	1089691000000105	Psychotic disorders
1975191000006114	Post-schizophrenic depression, continuous	1975191000006105	Psychotic disorders
1972301000006114	Mental & behav dis due to cannabinoids: psychotic disordr, predominantly hallucinatory	1972301000006105	Psychotic disorders
3504721000006117	Induced psychotic disorder	61831009	Psychotic disorders
5568191000006118	Acute polymorphic psychotic disorder	274953007	Psychotic disorders
294924017	Psychogenic paranoid psychosis	191680007	Psychotic disorders
1452014	Delusion of persecution	216004	Psychotic disorders
1975721000006113	Mental and behav dis due to other stimulants inc caffeine psychotic dis, mixed	1975721000006109	Psychotic disorders
14472391000006112	Acute psychosis	65971000052100	Psychotic disorders
362421000006113	Acute schizophrenia-like psychotic disorder	278853003	Psychotic disorders
1974791000006111	Mental & behav dis due to use tobacco: psychotic disorder, predominantly manic symptoms	1974791000006107	Psychotic disorders
378051000006119	[X]Disorganised schizophrenia	35252006	Psychotic disorders
4763231000006112	Drug induced psychosis	191483003	Psychotic disorders
1973131000006114	Mental & behav dis due to vol solvents: psychotic disordr, mixed	1973131000006105	Psychotic disorders
556631000006116	Chronic schizoaffective schizophrenia	191570001	Psychotic disorders
401768012	Chronic paranoid psychosis	268622001	Psychotic disorders
1973121000006111	Mental & behav dis due to vol solvents: psychotic disordr, predominantly manic symptoms	1973121000006107	Psychotic disorders
1975781000006112	Other acute predominantly delusional psychotic disorders, with associated acute stress	1975781000006108	Psychotic disorders
215691000006112	Poor insight into psychotic condition	12200008	Psychotic disorders
1975561000006110	Mental and behav dis due to other stimulants inc caffeine: psychotic dis, schizophrenia-like	1975561000006106	Psychotic disorders
1975651000006114	Mental and behav dis due to other stimulants inc caffeine: psychotic dis, predominantly depressive symptoms	1975651000006105	Psychotic disorders
1974001000006119	Mental & behav dis due to use cocaine: resid & late-onset psychot dis, late-onset psychotic disorder	1974001000006103	Psychotic disorders
1974711000006118	Mental & behav dis due to use tobacco: psychotic disorder, predominantly hallucinatory	1974711000006102	Psychotic disorders

3636410011	Psychotic disorder caused by stimulant	762325009	Psychotic disorders
1975321000006114	Post-schizophrenic depression, course uncertain, period of observation too short	1975321000006105	Psychotic disorders
1976191000006117	Mental and behav dis due to other stimulants inc caffeine: resid/late-onset psycht dis, late-onset psychotic disorder	1976191000006101	Psychotic disorders
1973141000006116	Mental and behav dis due to vol solvents: resid & late-onset psychotic dis, flashbacks	1973141000006100	Psychotic disorders
397581000006111	Cannabis-induced psychosis	943081000000102	Psychotic disorders
1975341000006119	Mental & behav dis due to use alcohol: psychotic disorder, predominantly polymorphic	1975341000006103	Psychotic disorders
1974651000006110	Mental & behav dis due to use tobacco: psychotic disorder, schizophrenia-like	1974651000006106	Psychotic disorders
1973161000006117	Mental and behav dis due to vol solvents: resid & late-onset psychotic dis, residual affective disorder	1973161000006101	Psychotic disorders
294929010	Other reactive psychoses NOS	231437006	Psychotic disorders
1973731000006110	Mental & behav dis due to use cocaine: psychotic disorder, predominantly depressive symptoms	1973731000006106	Psychotic disorders
5517971000006118	Mixed schizophrenic and affective psychosis	270901009	Psychotic disorders
401764014	Other schizophrenia	58214004	Psychotic disorders
223611000000117	Non-organic psychosis	191525009	Psychotic disorders
1973071000006111	Mental & behav dis due to vol solvents: psychotic disordr, predominantly delusional	1973071000006107	Psychotic disorders
42731000006112	Organic psychotic condition	191447007	Psychotic disorders
1975291000006117	Mental & behav dis due to use alcohol: psychotic disorder, predominantly hallucinatory	1975291000006101	Psychotic disorders
1973181000006110	Mental and behav dis due to vol solvents: resid & late-onset psychotic dis, other persisting cognitive impairment	1973181000006106	Psychotic disorders
1974811000006110	Mental & behav dis due to use tobacco: psychotic disorder, mixed	1974811000006106	Psychotic disorders
3627341000006115	Psychosis	69322001	Psychotic disorders
294949018	Other specified non-organic psychoses	191525009	Psychotic disorders
1975761000006119	Other acute predominantly delusional psychotic disorders, without associated acute stress	1975761000006103	Psychotic disorders
1971571000006118	Mental & behav dis due to use opioids: psychotic disorder, predominantly depressive symptoms	1971571000006102	Psychotic disorders
294675017	Other alcoholic psychosis NOS	42344001	Psychotic disorders
1975851000006110	Other acute and transient psychotic disorders, with associated acute stress	1975851000006106	Psychotic disorders
1972941000006110	Mental & behav dis due to seds/hypntcs: psychotic disordr, predominantly depressive symptoms	1972941000006106	Psychotic disorders
294926015	Other reactive psychoses	231437006	Psychotic disorders
1973091000006112	Mental & behav dis due to hallucinogens: psychotic disord, mixed	1973091000006108	Psychotic disorders
296066015	[X]Persistent delusional disorder, unspecified	231487004	Psychotic disorders
1975461000006114	Mental & behav dis due to use alcohol: psychotic disorder, mixed	1975461000006105	Psychotic disorders
1973101000006118	Mental & behav dis due to vol solvents: psychotic disordr, predominantly polymorphic	1973101000006102	Psychotic disorders
362271000006110	Acute transient psychotic disorder	231489001	Psychotic disorders
1973781000006111	Mental and behav dis due to hallucinogens: resid & late-onset psychot dis, late-onset psychotic disorder	1973781000006107	Psychotic disorders
368011000006110	[X]Brief schizophrenifrm psych	278853003	Psychotic disorders
294730012	Acute exacerbation of subchronic schizophrenia	111482003	Psychotic disorders
370461000006112	[X]Catatonic stupor	191542003	Psychotic disorders
1973751000006115	Mental & behav dis due to use cocaine: psychotic disorder, predominantly manic symptoms	1973751000006104	Psychotic disorders
1972861000006118	Mental & behav dis due to seds/hypntcs: psychotic disordr, schizophrenia-like	1972861000006102	Psychotic disorders
25461000006115	Other nonorganic psychoses	191525009	Psychotic disorders
425711000006116	[X]Schizophrenifrm psychos NOS	58214004	Psychotic disorders
1972461000006116	Mental & behav dis due to cannabinoids: resid & late-onset psychot dis, late-onset psychotic disorder	1972461000006100	Psychotic disorders
418251000006110	Late paraphrenia	38295006	Psychotic disorders
1975711000006117	Acute schizophrenia-like psychotic disorder, without associated acute stress	1975711000006101	Psychotic disorders
1975871000006117	Acute and transient psychotic disorder, unspecified, without associated acute stress	1975871000006101	Psychotic disorders
425921000006114	[X]Sensitiver Beziehungswahn	41189006	Psychotic disorders
215851000000112	[X]Paranoid psychosis	191667009	Psychotic disorders

294771018	Latent schizophrenia NOS	191559008	Psychotic disorders
423271000006116	Prepsychotic schizophrenia	247804008	Psychotic disorders
1975261000006113	Post-schizophrenic depression, episodic remittent	1975261000006109	Psychotic disorders
3182591000006114	Alcohol induced psychosis	42344001	Psychotic disorders
294753013	Catatonic schizophrenia NOS	191542003	Psychotic disorders
294806015	Single manic episode, severe, with psychosis	191586008	Psychotic disorders
425601000006114	[X]Schizophrenia	58214004	Psychotic disorders
401856011	Schizotypal personality disorder	31027006	Psychotic disorders
1973791000006114	Mental & behav dis due to use cocaine: psychotic disorder, mixed	1973791000006105	Psychotic disorders
13667221000006114	Psychotic behaviour caused by ingestible alcohol	787768005	Psychotic disorders
2586801000006115	Brief psychotic disorder	5464005	Psychotic disorders
294726014	Schizophrenia simplex	191527001	Psychotic disorders
401857019	[X]Other persistent delusional disorders	231487004	Psychotic disorders
5023901000006110	Post-schizophrenic depression	231485007	Psychotic disorders
456731000006115	Acute exacerbation of chronic schizo-affective schizophrenia	191572009	Psychotic disorders
1971521000006119	Mental & behav dis due to use opioids: psychotic disorder, predominantly hallucinatory	1971521000006103	Psychotic disorders
425521000006115	Schizoaffective disorder, mixed type	270901009	Psychotic disorders
317006016	Olfactory hallucinations	39672001	Psychotic disorders
294789017	Other schizophrenia NOS	58214004	Psychotic disorders
424841000006113	[X]Residual schizophrenia	26025008	Psychotic disorders
367991000006119	[X]Brief reactive psychosis NOS	231489001	Psychotic disorders
424281000006115	[X]Puerperal psychosis NOS	199260001	Psychotic disorders
1975141000006117	Mental & behav dis due to use alcohol: psychotic disorder, schizophrenia-like	1975141000006101	Psychotic disorders
425551000006112	[X]Schizoaffective psychosis NOS	68890003	Psychotic disorders
425571000006119	[X]Schizoaffective psychosis, manic type	271428004	Psychotic disorders
397691000006113	Volatile inhalant-induced psychosis	943151000000109	Psychotic disorders
419861000006117	Persistent delusional disorder	231487004	Psychotic disorders
376501000006110	[X]Delusional dysmorphophobia	231487004	Psychotic disorders
294744018	Unspecified catatonic schizophrenia	191542003	Psychotic disorders
7849381000006118	Cannabis-induced psychotic disorder	723936000	Psychotic disorders
294919015	Acute hysterical psychosis	191677006	Psychotic disorders
294760019	Paranoid schizophrenia NOS	64905009	Psychotic disorders
296031017	Undifferentiated schizophrenia	111484002	Psychotic disorders
5023391000006116	Reactive psychosis	231437006	Psychotic disorders
425541000006110	Schizoaffective disorder	68890003	Psychotic disorders
480834016	Delusions	2073000	Psychotic disorders
425561000006114	[X]Schizoaffective psychosis, depressive type	84760002	Psychotic disorders
1973111000006115	Mental & behav dis due to vol solvents: psychotic disordr, predominantly depressive symptoms	1973111000006104	Psychotic disorders
1971741000006116	Mental & behav dis due to use opioids: resid & late-onset psychot dis, late-onset psychotic disorder	1971741000006100	Psychotic disorders
401763015	Acute schizophrenic episode	268617001	Psychotic disorders
1973151000006119	Mental and behav dis due to vol solvents: resid & late-onset psychotic dis, personality or behaviour disorder	1973151000006103	Psychotic disorders
402504012	Psychotic condition, insight present	268957000	Psychotic disorders
1975371000006110	Mental & behav dis due to use alcohol: psychotic disorder, predominantly depressive symptoms	1975371000006106	Psychotic disorders
1975231000006116	Post-schizophrenic depression, episodic with stable deficit	1975231000006100	Psychotic disorders
294739013	Acute exacerbation of chronic hebephrenic schizophrenia	191539009	Psychotic disorders
1973691000006119	Mental & behav dis due to use cocaine: psychotic disorder, predominantly polymorphic	1973691000006103	Psychotic disorders
7696261000006113	Acute polymorphic psychotic disorder with symptoms of schizophrenia	712850003	Psychotic disorders
401860014	[X]Acute and transient psychotic disorder, unspecified	231489001	Psychotic disorders
296096013	[X]Schizoaffective disorder, unspecified	68890003	Psychotic disorders
3527206014	Secondary psychotic syndrome with hallucinations and delusions	737225007	Psychotic disorders
1971471000006117	Mental & behav dis due to use opioids: psychotic disorder, schizophrenia-like	1971471000006101	Psychotic disorders
294735019	Unspecified hebephrenic schizophrenia	35252006	Psychotic disorders
294913019	Paranoid psychosis	191667009	Psychotic disorders
294725013	Schizophrenic disorders	191526005	Psychotic disorders
418231000006115	[X]Paranoid state	191667009	Psychotic disorders

294773015	Cyclic schizophrenia	191567000	Psychotic disorders
1975611000006113	Mental and behav dis due to other stimulants inc caffeine: psychotic dis, predominantly hallucinatory	1975611000006109	Psychotic disorders
1975701000006115	Mental and behav dis due to other stimulants inc caffeine: psychotic dis, predominantly manic symptoms	1975701000006104	Psychotic disorders
1974741000006119	Mental & behav dis due to use tobacco: psychotic disorder, predominantly polymorphic	1974741000006103	Psychotic disorders
7859631000006114	Psychotic disorder caused by cocaine	724689006	Psychotic disorders
294767016	Chronic latent schizophrenia	191562006	Psychotic disorders
294673012	Other alcoholic psychosis	42344001	Psychotic disorders
296022017	[X]Schizophrenia, schizotypal and delusional disorders	417601000000102	Psychotic disorders
401770015	Acute paranoid reaction	268624000	Psychotic disorders
1974981000006110	Mental & behav dis due to tobacco: resid & late-onset psychot dis, late-onset psychotic disorder	1974981000006106	Psychotic disorders
294765012	Unspecified latent schizophrenia	191559008	Psychotic disorders
1972881000006111	Mental & behav dis due to seds/hypntcs: psychotic disordr, predominantly delusional	1972881000006107	Psychotic disorders
1972361000006110	Mental & behav dis due to cannabinoids: psychotic disordr, mixed	1972361000006106	Psychotic disorders
397621000006111	Alcohol-induced psychosis	42344001	Psychotic disorders
1973651000006113	Mental & behav dis due to use cocaine: psychotic disorder, predominantly hallucinatory	1973651000006109	Psychotic disorders
425701000006119	[X]Schizophreniform psychosis, manic type	271428004	Psychotic disorders
5023541000006116	Psychosis associated with intensive care	231450007	Psychotic disorders
1975901000006117	Acute and transient psychotic disorder, unspecified, with associated acute stress	1975901000006101	Psychotic disorders
1975411000006111	Mental & behav dis due to use alcohol: psychotic disorder, predominantly manic symptoms	1975411000006107	Psychotic disorders
388741000006110	[X]Hebephrenic schizophrenia	35252006	Psychotic disorders
1972011000006110	Mental & behav dis due to hallucinogens: psychotic disord, schizophrenia-like	1972011000006106	Psychotic disorders
371031000006115	[X]Chronic undifferentiated schizophrenia	111484002	Psychotic disorders
819351000006115	Hebephrenic schizophrenia	35252006	Psychotic disorders
404041000006111	[X]Organic psychosis NOS	111479008	Psychotic disorders
395031000006114	[X]Latent schizophrenic reaction	191559008	Psychotic disorders
296083012	[X]Other acute and transient psychotic disorders	231489001	Psychotic disorders
1972961000006114	Mental & behav dis due to seds/hypntcs: psychotic disordr, predominantly manic symptoms	1972961000006105	Psychotic disorders
376281000006116	Cycloid psychosis	307417003	Psychotic disorders
294714016	Postpartum psychosis	18260003	Psychotic disorders
425641000006111	[X]Schizophrenic catalepsy	191542003	Psychotic disorders
317004018	Auditory hallucinations	45150006	Psychotic disorders
398631000006113	Mixed schizophrenic and affective pschosis	270901009	Psychotic disorders
362381000006115	Acute polymorphic psychotic disorder co-occurrent with symptoms of schizophrenia	712850003	Psychotic disorders
1973961000006115	Ment/behav dis mlti drug use/oth psyc sbs: psychotc dis, predominantly depressive symptoms	1973961000006104	Psychotic disorders
423731000006114	[X]Pseudoneurotic schizophrenia	31027006	Psychotic disorders
71539017	Subchronic catatonic schizophrenia	42868002	Psychotic disorders
1974681000006119	Mental & behav dis due to use tobacco: psychotic disorder, predominantly delusional	1974681000006103	Psychotic disorders
123611000006110	Subchronic schizo-affective schizophrenia	191569002	Psychotic disorders
1971601000006113	Mental & behav dis due to use opioids: psychotic disorder, predominantly manic symptoms	1971601000006109	Psychotic disorders
412201000006113	[X]Other nonorganic psychotic disorders	191525009	Psychotic disorders
294728010	Unspecified schizophrenia	58214004	Psychotic disorders
401859016	[X]Other acute predominantly delusional psychotic disorders	231489001	Psychotic disorders
317007013	Tactile hallucinations	66609003	Psychotic disorders
294734015	Simple schizophrenia NOS	191527001	Psychotic disorders
476741000006111	Alcohol-induced psychosis	42344001	Psychotic disorders
294758016	Acute exacerbation of chronic paranoid schizophrenia	191555002	Psychotic disorders
1973051000006118	Mental & behav dis due to vol solvents: psychotic disordr, schizophrenia-like	1973051000006102	Psychotic disorders
215871000000115	[X]Paranoia	417233008	Psychotic disorders
5106081000006110	Severe postnatal psychosis	237352005	Psychotic disorders
346895011	Psychotic disorder	69322001	Psychotic disorders
1219653018	Atypical schizophrenia	111484002	Psychotic disorders
456801000006115	Acute exacerbation subchronic schizo-affective schizophrenia	191571002	Psychotic disorders

294788013	Cenesthopathic schizophrenia	191577003	Psychotic disorders
389451000006110	[X]Hysterical psychosis	44376007	Psychotic disorders
1975581000006117	Mental and behav dis due to other stimulants inc caffeine: psychotic dis, predominantly delusional	1975581000006101	Psychotic disorders
4569010	Delusion	2073000	Psychotic disorders
294754019	Unspecified paranoid schizophrenia	64905009	Psychotic disorders
368001000006112	[X]Brief schizophreniform disorder	278853003	Psychotic disorders
1972261000006117	Mental & behav dis due to cannabinoids: psychotic disordr, schizophrenia-like	1972261000006101	Psychotic disorders
294790014	Schizophrenia	58214004	Psychotic disorders
7860831000006112	Manic symptoms with primary psychotic disorder	724758000	Psychotic disorders
394461000006110	[X]Involutional paranoid state	231487004	Psychotic disorders
317005017	Gustatory hallucinations	29139005	Psychotic disorders
395021000006111	[X]Latent schizophrenia	191559008	Psychotic disorders
1973191000006113	Mental and behav dis due to vol solvents: resid & late-onset psychotic dis, late-onset psychotic disorder	1973191000006109	Psychotic disorders
424231000006116	Psychotic	69322001	Psychotic disorders
294743012	[X]Schizophrenic flexibilatis cerea	191542003	Psychotic disorders
376271000006119	[X]Cycloid psychosis	307417003	Psychotic disorders
12736721000006110	FEP - first episode psychosis	1127191000000108	Psychotic disorders
1976411000006115	Other recurrent mood affective disorders, recurrent brief depressive disorder	1976411000006104	Psychotic disorders
426881000006111	[X]Simple schizophrenia	191527001	Psychotic disorders
398741000006114	[X]Mental and behavioural disorders due to use of other stimulants, including caffeine: residual and late onset psychotic disorder	762325009	Psychotic disordesr
398751000006111	[X]Mnt/bh dis vol solvents: resid & late-onset psychotic dis	91388009	Psychotic disordesr
980141000006119	[X]Mental and behavioural disorders due to use of crack cocaine: psychotic disorder	46975003	Psychotic disordesr
397591000006114	[X]Mental and behavioural disorders due to use of hallucinogens: psychotic disorder	943131000000102	Psychotic disordesr
397681000006110	[X]Mental and behavioural disorders due to use of tobacco: psychotic disorder	30310000	Psychotic disordesr
398281000006112	[X]Mental and behavioural disorders due to use of other stimulants, including caffeine: psychotic disorder	762325009	Psychotic disordesr
397541000006117	[X]Mental and behavioural disorders due to multiple drug use and use of other psychoactive substances: psychotic disorder	191483003	Psychotic disordesr
397601000006118	[X]Mental and behavioural disorders due to use of sedatives or hypnotics: psychotic disorder	943091000000100	Psychotic disordesr
397181000006117	[X]Mental and behavioural disorders due to use of tobacco: residual and late-onset psychotic disorder	30310000	Psychotic disordesr

Hospital episode statistics: Mental ill-health (other than autism)	
**icd**	**Description**
F20	Schizophrenia
F21	Schizotypal disorder
F22	Persistent delusional disorders
F23	Acute and transient psychotic disorders
F24	Induced delusional disorder
F25	Schizoaffective disorders
F26	Other nonorganic psychotic disorders
F29	Unspecified nonorganic psychosis
F30	Manic episode
F31	Bipolar affective disorder
F32	Depressive episode
F33	Recurrent depressive disorder
F34	Persistent mood [affective] disorders
F38	Other mood [affective] disorders
F39	Unspecified mood [affective] disorder
F41	Other anxiety disorders
F42	Obsessive-compulsive disorder
F43	Reaction to severe stress, and adjustment disorders
F44	Dissociative [conversion] disorders
F45	Somatoform disorders
F48	Other neurotic disorders
F50	Eating disorders

F60	Specific personality disorders
F61	Mixed and other personality disorders
F90.0	Disturbance of activity and attention (ADHD)

Clinical codes for osteoporosis

GOLD: clinical, referral, tests; Aurum: consultations, observations.

Process measures (e.g. referrals, monitoring) not included.

Additional sources of information in addition to clinical opinion and in-house code repository: Zghebi et al. 2021: https://clinicalcodes.rss.mhs.man.ac.uk/medcodes/article/180/codelist/osteoporosis/; Kwon et al. 2022: https://www.ncbi.nlm.nih.gov/pmc/articles/PMC9823608/

CPRD GOLD: Osteoporosis		
**medcode**	**readcode**	**readterm**
38395	N331B00	Postmenopausal osteoporosis with pathological fracture
277	N330.00	Osteoporosis
57301	NyuB000	[X]Other osteoporosis with pathological fracture
13987	58E4.00	Forearm DXA scan result osteoporotic
14967	N330000	Osteoporosis, unspecified
16857	N330C00	Osteoporosis localized to spine
93655	N330700	Postsurgical malabsorption osteoporosis
31580	N330A00	Osteoporosis in endocrine disorders
96342	58EV.00	Femoral neck DEXA scan result osteoporotic
34798	N330z00	Osteoporosis NOS
68019	N331400	Postsurgical malabsorption osteoporosis with path fracture
40428	N330300	Idiopathic osteoporosis
54232	N330800	Localized osteoporosis - Lequesne
51891	585O.00	Quantitative ultrasound scan of heel - result osteoporotic
60433	N330900	Osteoporosis in multiple myelomatosis
102730	NyuB200	[X]Osteoporosis in other disorders classified elsewhere
24093	N330500	Drug-induced osteoporosis
39334	N331200	Postoophorectomy osteoporosis with pathological fracture
39217	58EM.00	Lumbar DXA scan result osteoporotic
48772	N331A00	Osteoporosis + pathological fracture cervical vertebrae
12673	N331900	Osteoporosis + pathological fracture thoracic vertebrae
16307	N330100	Senile osteoporosis
42354	58EG.00	Hip DXA scan result osteoporotic
18825	NyuB800	[X]Unspecified osteoporosis with pathological fracture
41755	NyuB100	[X]Other osteoporosis
25650	N330D00	Osteoporosis due to corticosteroids
70349	N330600	Postoophorectomy osteoporosis
17377	N331800	Osteoporosis + pathological fracture lumbar vertebrae
9700	N330200	Postmenopausal osteoporosis
46510	58EA.00	Heel DXA scan result osteoporotic
3346	N330B00	Vertebral osteoporosis
62702	N330400	Dissuse osteoporosis
93497	N331N00	Fragility fracture
33526	N331300	Osteoporosis of disuse with pathological fracture
27597	N331600	Idiopathic osteoporosis with pathological fracture
46894	N331500	Drug-induced osteoporosis with pathological fracture

CPRD Aurum: Osteoporosis		
**medcode**	**Term**	**snomedconceptID**
24883018	Drug-induced osteoporosis	14651005
30475015	Senile osteoporosis	18040001
54058015	Postmenopausal osteoporosis	102447009
107806013	Osteoporosis	64859006
312124013	Osteoporosis. Unspecified	64859006
312133010	Postoophorectomy osteoporosis	203433000
312136019	Localised osteoporosis - Lequesne	203435007
312137011	Osteoporosis in multiple myelomatosis	739301006
312138018	Osteoporosis in endocrine disorders	203437004
312139014	Vertebral osteoporosis	203438009

312142015	Osteoporosis NOS	64859006
312156018	Postoophorectomy osteoporosis with pathological fracture	203444008
312157010	Osteoporosis of disuse with pathological fracture	203445009
312159013	Drug-induced osteoporosis with pathological fracture	203447001
312160015	Idiopathic osteoporosis with pathological fracture	203448006
312165013	Postmenopausal osteoporosis with pathological fracture	203453001
312681016	[X]Other osteoporosis with pathological fracture	443165006
312682011	[X]Other osteoporosis	64859006
312683018	[X]Osteoporosis in other disorders classified elsewhere	64859006
312689019	[X]Unspecified osteoporosis with pathological fracture	443165006
453226012	Osteoporosis localised to spine	309745002
1484866013	Osteoporosis due to corticosteroids	390833005
1485088019	Forearm DXA scan result osteoporotic	391060008
1485093016	Heel DXA scan result osteoporotic	391065003
1485098013	Hip DXA scan result osteoporotic	391070005
1485103010	Lumbar DXA scan result osteoporotic	391075000
1485109014	Quantitative ultrasound scan of heel - result osteoporotic	391081008
2475469012	Posttraumatic osteoporosis	15743005
3515008018	Osteoporosis due to malabsorption	735618008
40871000006119	Osteoporosis with pathological fracture of cervical vertebrae	203452006
40881000006116	Osteoporosis with pathological fracture of lumbar vertebrae	203450003
40891000006118	Osteoporosis with pathological fracture of thoracic vertebrae	203451004
212431000006119	Postsurgical malabsorption osteoporosis	203434006
212441000006112	Post-surgical malabsorption osteoporosis with pathological fracture	203446005
545121000000114	Fragility fracture	306171000000106
625381000006110	Dissuse osteoporosis	53174001
668551000000116	Femoral neck DXA scan result osteoporotic	440100002
786501000006117	Idiopathic generalised osteoporosis	203429007
1823981000006115	Osteoporosis confirmed	1823981000006104
2552271000006117	Idiopathic osteoporosis	3345002
3021381000006110	Menopausal osteoporosis	32369003
3021401000006110	Type 1 osteoporosis	32369003
3362291000006115	Disuse osteoporosis	53174001
3555091000006111	OP - Osteoporosis	64859006
5144201000006113	Secondary generalised osteoporosis	240157009
5144261000006114	Secondary localised osteoporosis	240162005
5588801000006119	Primary osteoporosis	276661002
5588811000006116	Age-related osteoporosis	276661002
5992471000006115	Osteoporosis localized to spine	309745002
6515141000006116	Osteoporosis due to corticosteroid	390833005
7573051000006110	Secondary osteoporosis	703264005
7617261000006112	Osteoporosis due to cystic fibrosis	707419009
13622711000006116	X-linked osteoporosis with fractures	782785002

Hospital episode statistics: Osteoporosis	
**icd**	**description**
M80	Osteoporosis with pathological fracture
M81	Osteoporosis without pathological fracture
M82	Osteoporosis in diseases elsewhere classified

Clinical codes for respiratory disorders

GOLD: clinical, referral, tests; Aurum: consultations, observations.

Process measures (e.g. referrals, monitoring) not included.

Additional sources of information in addition to clinical opinion and in-house code repository: Davidson et al. 2021: https://datacompass.lshtm.ac.uk/id/eprint/2214/; https://datacompass.lshtm.ac.uk/id/eprint/2208/

COPD: chronic obstructive pulmonary disorders

CPRD GOLD: Respiratory disorders			
**medcode**	**readcode**	**readterm**	**interpretation**
78	H33..00	Asthma	Asthma
185	H333.00	Acute exacerbation of asthma	Asthma

232	H33z100	Asthma attack	Asthma
233	H33z011	Severe asthma attack	Asthma
1555	H33..11	Bronchial asthma	Asthma
2290	H330.11	Allergic asthma	Asthma
3018	663V100	Mild asthma	Asthma
3366	663V300	Severe asthma	Asthma
3458	663V000	Occasional asthma	Asthma
3665	H331.11	Late onset asthma	Asthma
4442	H33z.00	Asthma unspecified	Asthma
4606	H33zz11	Exercise induced asthma	Asthma
4892	H33z000	Status asthmaticus NOS	Asthma
5267	H331.00	Intrinsic asthma	Asthma
5627	H330011	Hay fever with asthma	Asthma
5867	173A.00	Exercise induced asthma	Asthma
6707	H330111	Extrinsic asthma with asthma attack	Asthma
7146	H330.00	Extrinsic (atopic) asthma	Asthma
7731	H330.14	Pollen asthma	Asthma
8335	H33z111	Asthma attack NOS	Asthma
10487	663j.00	Asthma - currently active	Asthma
11370	1O2..00	Asthma confirmed	Asthma
12987	H33z200	Late-onset asthma	Asthma
13065	663V200	Moderate asthma	Asthma
13066	663h.00	Asthma - currently dormant	Asthma
14777	H330000	Extrinsic asthma without status asthmaticus	Asthma
15248	H330.13	Hay fever with asthma	Asthma
16070	H33zz00	Asthma NOS	Asthma
18323	H331111	Intrinsic asthma with asthma attack	Asthma
21232	H33zz12	Allergic asthma NEC	Asthma
22752	173c.00	Occupational asthma	Asthma
25796	H332.00	Mixed asthma	Asthma
27926	H330100	Extrinsic asthma with status asthmaticus	Asthma
29325	H331000	Intrinsic asthma without status asthmaticus	Asthma
39478	H35y700	Wood asthma	Asthma
40823	H334.00	Brittle asthma	Asthma
41017	1780.00	Aspirin induced asthma	Asthma
45073	H331z00	Intrinsic asthma NOS	Asthma
45782	H330z00	Extrinsic asthma NOS	Asthma
47684	H47y000	Detergent asthma	Asthma
58196	H331100	Intrinsic asthma with status asthmaticus	Asthma
73522	173d.00	Work aggravated asthma	Asthma
93353	H35y600	Sequoiosis (red-cedar asthma)	Asthma
106805	H335.00	Chronic asthma with fixed airflow obstruction	Asthma
794	H32..00	Emphysema	COPD
998	H3...11	Chronic obstructive airways disease	COPD
1001	H3...00	Chronic obstructive pulmonary disease	COPD
1446	H312200	Acute exacerbation of chronic obstructive airways disease	COPD
3243	H31..00	Chronic bronchitis	COPD
5710	H3z..00	Chronic obstructive airways disease NOS	COPD
5798	H312000	Chronic asthmatic bronchitis	COPD
5909	H312011	Chronic wheezy bronchitis	COPD
7884	H3y1.00	Chron obstruct pulmonary dis wth acute exacerbation, unspec	COPD
9876	H38..00	Severe chronic obstructive pulmonary disease	COPD
10802	H37..00	Moderate chronic obstructive pulmonary disease	COPD
10863	H36..00	Mild chronic obstructive pulmonary disease	COPD
10980	H322.00	Centrilobular emphysema	COPD
11150	H311.00	Mucopurulent chronic bronchitis	COPD
12166	H3y..00	Other specified chronic obstructive airways disease	COPD
14798	H312100	Emphysematous bronchitis	COPD
15157	H31z.00	Chronic bronchitis NOS	COPD
16410	H32yz00	Other emphysema NOS	COPD
21061	H3y0.00	Chronic obstruct pulmonary dis with acute lower resp infectn	COPD
22905	H581.00	Interstitial emphysema	COPD
23492	H320z00	Chronic bullous emphysema NOS	COPD
24248	H313.00	Mixed simple and mucopurulent chronic bronchitis	COPD
25603	H310.00	Simple chronic bronchitis	COPD
26306	H320.00	Chronic bullous emphysema	COPD

27819	H312.00	Obstructive chronic bronchitis	COPD
33450	H32z.00	Emphysema NOS	COPD
37247	H3z..11	Chronic obstructive pulmonary disease NOS	COPD
37959	H311100	Fetid chronic bronchitis	COPD
40159	H311000	Purulent chronic bronchitis	COPD
40788	H32y.00	Other emphysema	COPD
44525	H312z00	Obstructive chronic bronchitis NOS	COPD
45089	H31y100	Chronic tracheobronchitis	COPD
46578	H321.00	Panlobular emphysema	COPD
54893	H582.00	Compensatory emphysema	COPD
56860	H320000	Segmental bullous emphysema	COPD
59263	H32y111	Acute interstitial emphysema	COPD
60188	H320200	Giant bullous emphysema	COPD
61118	H310z00	Simple chronic bronchitis NOS	COPD
61513	H311z00	Mucopurulent chronic bronchitis NOS	COPD
63479	H32y200	MacLeod's unilateral emphysema	COPD
64721	H464000	Chronic emphysema due to chemical fumes	COPD
65733	Hyu3100	[X]Other specified chronic obstructive pulmonary disease	COPD
66043	H31y.00	Other chronic bronchitis	COPD
66058	Hyu3000	[X]Other emphysema	COPD
67040	H3y..11	Other specified chronic obstructive pulmonary disease	COPD
68066	H31yz00	Other chronic bronchitis NOS	COPD
68662	H320100	Zonal bullous emphysema	COPD
70787	H32y100	Atrophic (senile) emphysema	COPD
92955	H32y000	Acute vesicular emphysema	COPD
93568	H39..00	Very severe chronic obstructive pulmonary disease	COPD
99536	H320300	Bullous emphysema with collapse	COPD
103733	H320311	Tension pneumatocoele	COPD
104608	H3A..00	End stage chronic obstructive airways disease	COPD
109958	H3B..00	Asthma-chronic obstructive pulmonary disease overlap syndrom	COPD
2195	H34..00	Bronchiectasis	Other respiratory conditions
3859	H57y200	Pulmonary sarcoidosis	Other respiratory conditions
4084	663K.00	Airways obstructn irreversible	Other respiratory conditions
5005	H410.00	Pleural plaque disease due to asbestosis	Other respiratory conditions
5519	H563.12	Cryptogenic fibrosing alveolitis	Other respiratory conditions
6051	H563100	Diffuse pulmonary fibrosis	Other respiratory conditions
6181	H061400	Obliterating fibrous bronchiolitis	Other respiratory conditions
6220	C370.00	Cystic fibrosis	Other respiratory conditions
6837	H563.00	Idiopathic fibrosing alveolitis	Other respiratory conditions
7092	H30..12	Recurrent wheezy bronchitis	Other respiratory conditions
7791	H55..00	Postinflammatory pulmonary fibrosis	Other respiratory conditions
8303	H41..00	Asbestosis	Other respiratory conditions
9954	H570.00	Rheumatoid lung	Other respiratory conditions
11312	H35..00	Extrinsic allergic alveolitis	Other respiratory conditions
11833	H35z100	Hypersensitivity pneumonitis NOS	Other respiratory conditions
13264	C370.11	Fibrocystic disease	Other respiratory conditions
15588	H350.00	Farmers' lung	Other respiratory conditions
15626	H310000	Chronic catarrhal bronchitis	Other respiratory conditions
15693	A115.00	Tuberculous bronchiectasis	Other respiratory conditions
18914	C370200	Cystic fibrosis with pulmonary manifestations	Other respiratory conditions
19492	H40..00	Coal workers' pneumoconiosis	Other respiratory conditions
20364	H340.00	Recurrent bronchiectasis	Other respiratory conditions
21257	H4...12	Occupational lung disease	Other respiratory conditions
22536	H4y1000	Chronic pulmonary fibrosis following radiation	Other respiratory conditions
22835	H564.00	Bronchiolitis obliterans organising pneumonia	Other respiratory conditions
23446	H42z.00	Silica pneumoconiosis NOS	Other respiratory conditions
23461	H43z.00	Pneumoconiosis due to inorganic dust NOS	Other respiratory conditions
24137	C370.12	Mucoviscidosis	Other respiratory conditions
24814	H591.00	Chronic respiratory failure	Other respiratory conditions
25013	H4...11	Pneumoconioses	Other respiratory conditions
26082	H541000	Chronic pulmonary oedema	Other respiratory conditions
26125	H312300	Bronchiolitis obliterans	Other respiratory conditions
26278	H357.00	'Ventilation' pneumonitis	Other respiratory conditions
26442	H441.00	Cannabinosis	Other respiratory conditions
27345	H352.00	Bird-fancier's lung	Other respiratory conditions
27348	H561.00	Idiopathic pulmonary haemosiderosis	Other respiratory conditions

28229	H563z00	Idiopathic fibrosing alveolitis NOS	Other respiratory conditions
28853	N04y012	Fibrosing alveolitis associated with rheumatoid arthritis	Other respiratory conditions
29966	C350300	Idiopathic pulmonary haemosiderosis	Other respiratory conditions
30235	H434.00	Siderosis	Other respiratory conditions
31423	H45..00	Pneumoconiosis NOS	Other respiratory conditions
31447	H352100	Pigeon-fanciers' lung	Other respiratory conditions
31564	H57y400	Lung disease with systemic lupus erythematosus	Other respiratory conditions
31724	N04y000	Rheumatoid lung	Other respiratory conditions
32679	H34z.00	Bronchiectasis NOS	Other respiratory conditions
33980	AD50.00	Sarcoidosis of lung	Other respiratory conditions
36240	H352z00	Bird-fancier's lung NOS	Other respiratory conditions
36622	C370111	Meconium ileus in cystic fibrosis	Other respiratory conditions
37365	H440.00	Byssinosis	Other respiratory conditions
40953	H55..11	Cirrhosis of lung	Other respiratory conditions
41491	H341.00	Post-infective bronchiectasis	Other respiratory conditions
41694	H355.00	Mushroom workers' lung	Other respiratory conditions
42940	H57y100	Lung disease with polymyositis	Other respiratory conditions
43285	H48..00	Progressive massive fibrosis	Other respiratory conditions
43417	H4y2100	Chronic drug-induced interstitial lung disorders	Other respiratory conditions
44015	H4y2.00	Drug-induced interstitial lung disorders	Other respiratory conditions
45427	H35y500	Pituitary snuff-takers' disease	Other respiratory conditions
46436	N042100	Rheumatoid lung disease	Other respiratory conditions
46460	H42..00	Silica and silicate pneumoconiosis	Other respiratory conditions
46795	Q317100	Prematurity with interstitial pulmonary fibrosis	Other respiratory conditions
46977	H35z.00	Allergic alveolitis and pneumonitis NOS	Other respiratory conditions
47142	H464.00	Chronic respiratory conditions due to chemical fumes	Other respiratory conditions
47364	H57y300	Lung disease with Sjogren's disease	Other respiratory conditions
47782	H464200	Chronic pulmonary fibrosis due to chemical fumes	Other respiratory conditions
49194	H432.00	Berylliosis	Other respiratory conditions
49770	C370z00	Cystic fibrosis NOS	Other respiratory conditions
50374	H4y1z00	Chronic pulmonary radiation disease NOS	Other respiratory conditions
51410	H41z.00	Asbestosis NOS	Other respiratory conditions
51858	H35y.00	Other allergic alveolitis	Other respiratory conditions
53095	H35zz00	Allergic alveolitis and pneumonitis NOS	Other respiratory conditions
53943	H35y100	Coffee-workers' lung	Other respiratory conditions
54010	H57y000	Pulmonary amyloidosis	Other respiratory conditions
54822	H352000	Budgerigar-fanciers' lung	Other respiratory conditions
55552	H35yz00	Other allergic alveolitis NOS	Other respiratory conditions
56427	P861.00	Congenital bronchiectasis	Other respiratory conditions
56652	H356.00	Maple bark strippers' lung	Other respiratory conditions
56838	N04y011	Caplan's syndrome	Other respiratory conditions
58841	AD52.00	Sarcoidosis of lung with sarcoidosis of lymph nodes	Other respiratory conditions
59188	Hy02.00	Chronic pulmonary insufficiency following surgery	Other respiratory conditions
60313	H44..00	Pneumopathy due to inhalation of other dust	Other respiratory conditions
60805	H420.00	Talc pneumoconiosis	Other respiratory conditions
62200	H351.00	Bagassosis	Other respiratory conditions
62233	H421.00	Simple silicosis	Other respiratory conditions
62442	H35z000	Allergic extrinsic alveolitis NOS	Other respiratory conditions
63172	H450.00	Pneumoconiosis associated with tuberculosis	Other respiratory conditions
63216	H464100	Obliterative bronchiolitis due to chemical fumes	Other respiratory conditions
64799	H571.00	Rheumatic pneumonia	Other respiratory conditions
65060	Hyu5000	[X]Other interstitial pulmonary diseases with fibrosis	Other respiratory conditions
65344	C370000	Cystic fibrosis with no meconium ileus	Other respiratory conditions
65376	H43..00	Pneumoconiosis due to other inorganic dust	Other respiratory conditions
67709	H354.00	Malt workers' lung	Other respiratory conditions
69017	C370100	Cystic fibrosis with meconium ileus	Other respiratory conditions
69452	H35y800	Air-conditioner and humidifier lung	Other respiratory conditions
69914	H4y1.00	Chronic pulmonary radiation disease	Other respiratory conditions
70286	H35y200	Fish-meal workers' lung	Other respiratory conditions
70815	H464z00	Chronic respiratory conditions due to chemical fumes NOS	Other respiratory conditions
71853	H422.00	Complicated silicosis	Other respiratory conditions
73065	C370y00	Cystic fibrosis with other manifestations	Other respiratory conditions
73414	H44z.00	Pneumopathy due to inhalation of other dust NOS	Other respiratory conditions
89206	H423.00	Massive silicotic fibrosis	Other respiratory conditions
91989	H35y000	Cheese-washers' lung	Other respiratory conditions
93206	H353.00	Suberosis (cork-handlers' lung)	Other respiratory conditions

93577	H435.00	Stannosis	Other respiratory conditions
94486	H593.00	Chronic type 2 respiratory failure	Other respiratory conditions
94575	H433.00	Graphite fibrosis of lung	Other respiratory conditions
94894	H431.00	Bauxite fibrosis of lung	Other respiratory conditions
94946	H592.00	Chronic type 1 respiratory failure	Other respiratory conditions
94996	H572.00	Lung disease with systemic sclerosis	Other respiratory conditions
100610	C370400	Arthropathy in cystic fibrosis	Other respiratory conditions
100994	H410.11	Asbestos-induced pleural plaque	Other respiratory conditions
102922	C370800	Cystic fibrosis related cirrhosis	Other respiratory conditions
103472	H563200	Pulmonary fibrosis	Other respiratory conditions
103475	H564.11	Cryptogenic organising pneumonia	Other respiratory conditions
103559	H563300	Usual interstitial pneumonitis	Other respiratory conditions
103637	H35..11	Hypersensitivity pneumonitis	Other respiratory conditions
103753	H563.13	Idiopathic pulmonary fibrosis	Other respiratory conditions
104915	H58y700	Interstitial lung disease due to connective tissue disease	Other respiratory conditions
105939	Hyu4000	[X]Pneumoconiosis due to other dust containing silica	Other respiratory conditions
106432	C370900	Exacerbation of cystic fibrosis	Other respiratory conditions
109815	H58y600	Interstitial lung disease due to collagen vascular disease	Other respiratory conditions
110454	C370700	Liver disease due to cystic fibrosis	Other respiratory conditions
111668	H32yz11	Sawyer - Jones syndrome	Other respiratory conditions
113768	C370600	Fibrosing colonopathy	Other respiratory conditions

CPRD Aurum: Respiratory disorders			
**medcode**	**term**	**snomedconceptID**	**interpretation**
21390015	Intrinsic asthma without status asthmaticus	12428000	Asthma
69311016	Detergent asthma	41553006	Asthma
94731013	Wood asthma	56968009	Asthma
95786019	Occupational asthma	57607007	Asthma
98546013	Life threatening acute exacerbation of intrinsic asthma	1086711000000100	Asthma
104872017	Extrinsic asthma without status asthmaticus	63088003	Asthma
151338014	Life threatening acute exacerbation of allergic asthma	1086701000000102	Asthma
301480018	Bronchial asthma	195967001	Asthma
301485011	Asthma	195967001	Asthma
301499010	Extrinsic asthma	424643009	Asthma
301508013	Intrinsic asthma NOS	266361008	Asthma
301509017	Mixed asthma	195977004	Asthma
301511014	Asthma unspecified	195967001	Asthma
338238011	Brittle asthma	225057002	Asthma
350148014	Late onset asthma	233679003	Asthma
350149018	Late-onset asthma	233679003	Asthma
350151019	Acute exacerbation of allergic asthma	708093000	Asthma
350152014	Allergic asthma NEC	389145006	Asthma
350153016	Hay fever with asthma	233683003	Asthma
350154010	Pollen asthma	233683003	Asthma
350156012	Acute exacerbation of intrinsic asthma	708094006	Asthma
396114013	Intrinsic asthma	266361008	Asthma
396118011	Life threatening acute exacerbation of asthma	734904007	Asthma
396119015	Asthma attack	708038006	Asthma
396120014	Asthma NOS	195967001	Asthma
409865018	Asthma attack NOS	708038006	Asthma
419211018	Acute exacerbation of asthma	708038006	Asthma
456163018	Asthma - currently active	312453004	Asthma
456164012	Asthma - currently dormant	312454005	Asthma
1208969012	Mild asthma	370218001	Asthma
1208970013	Moderate asthma	370219009	Asthma
1208971012	Occasional asthma	370220003	Asthma
1208972017	Severe asthma	370221004	Asthma
1483199016	Allergic asthma	389145006	Asthma
1780388018	Asthma confirmed	401193004	Asthma
3511374015	Moderate acute exacerbation of asthma	734905008	Asthma
3514925011	Acute severe exacerbation of asthma co-occurrent and due to allergic asthma	735587000	Asthma
3514928013	Uncomplicated allergic asthma	735588005	Asthma
3514930010	Uncomplicated non-allergic asthma	735589002	Asthma

3637387011	Exacerbation of allergic asthma	762521001	Asthma
145961000006117	Acute severe exacerbation of asthma	708090002	Asthma
149741000006116	Sequoiosis (red-cedar asthma)	23315001	Asthma
655601000006113	Exercise-induced asthma	31387002	Asthma
655611000006111	Exercise induced asthma	31387002	Asthma
660351000006118	Allergic atopic asthma	389145006	Asthma
817361000006114	Hay fever with asthma	233683003	Asthma
885291000006115	Extrinsic asthma - atopy	389145006	Asthma
929091000006111	Aspirin-induced asthma	407674008	Asthma
955631000006116	Occupational asthma	955631000006100	Asthma
983771000006118	Work aggravated asthma	57607007	Asthma
2009981000006110	Difficult asthma	2009981000006106	Asthma
2010031000006116	Acute infective exacerbation of asthma	2010031000006100	Asthma
2010041000006114	Acute non-infective exacerbation of asthma	2010041000006105	Asthma
2240591000000119	Chronic asthma with fixed airflow obstruction	866881000000101	Asthma
2685251000006117	Millers' asthma	11641008	Asthma
2789151000006118	Asthmatoid wheeze	18197001	Asthma
3005801000006117	EIA - Exercise-induced asthma	31387002	Asthma
3005811000006119	Exercise induced asthma	31387002	Asthma
3047731000006119	Bakers' asthma	34015007	Asthma
3435971000006119	Industrial asthma	57607007	Asthma
4009591000006110	Chemical-induced asthma	92807009	Asthma
4020511000006119	Drug-induced asthma	93432008	Asthma
4781531000006113	Asthmatic	195967001	Asthma
5054341000006110	Late-onset asthma	233679003	Asthma
5054381000006116	Sulphite-induced asthma	233688007	Asthma
5054401000006116	Factitious asthma	233690008	Asthma
5054431000006112	Asthmatic pulmonary eosinophilia	233691007	Asthma
5492441000006114	Non-allergic asthma	266361008	Asthma
5649191000006110	Exacerbation of asthma	281239006	Asthma
6298421000006116	Eosinophilic asthma	367542003	Asthma
6512381000006116	Atopic asthma	389145006	Asthma
6550131000006111	Nocturnal asthma	395022009	Asthma
6720721000006111	Asthmatic bronchitis	405944004	Asthma
6782071000006115	Cough variant asthma	409663006	Asthma
7015991000006110	Non-IgE mediated allergic asthma	423889005	Asthma
7021801000006116	Substance induced asthma	424199006	Asthma
7030311000006117	IgE-mediated allergic asthma	424643009	Asthma
7030321000006113	IgE mediated asthma	424643009	Asthma
7049011000006110	Acute exacerbation of chronic bronchitis	425748003	Asthma
7052221000006118	Exacerbation of intermittent asthma	425969006	Asthma
7062421000006112	Severe persistent asthma	426656000	Asthma
7067211000006115	Mild persistent asthma	426979002	Asthma
7071891000006112	Moderate persistent asthma	427295004	Asthma
7076331000006112	Intermittent asthma	427603009	Asthma
7077481000006119	Mild intermittent asthma	427679007	Asthma
7307941000006113	Seasonal asthma	445427006	Asthma
7617601000006114	Uncomplicated asthma	707444001	Asthma
7617611000006112	Exacerbation of mild persistent asthma	707445000	Asthma
7617621000006116	Exacerbation of moderate persistent asthma	707446004	Asthma
7617631000006118	Exacerbation of severe persistent asthma	707447008	Asthma
7618791000006110	Uncomplicated mild persistent asthma	707511009	Asthma
7618811000006114	Uncomplicated severe persistent asthma	707513007	Asthma
7626521000006110	Acute severe exacerbation of severe persistent asthma	707979007	Asthma
7626531000006113	Acute severe exacerbation of moderate persistent asthma	707980005	Asthma
7626541000006115	Acute severe exacerbation of mild persistent asthma	707981009	Asthma
7628271000006114	Acute exacerbation of extrinsic asthma	708093000	Asthma
7628301000006111	Acute severe exacerbation of allergic asthma	708095007	Asthma
7628321000006118	Acute severe exacerbation of immunoglobin E-mediated allergic asthma	708095007	Asthma
7628331000006115	Acute severe exacerbation of intrinsic asthma	708096008	Asthma
7961271000006111	Acute severe refractory exacerbation of asthma	733858005	Asthma
7961281000006114	Acute severe asthma	733858005	Asthma
7965491000006114	Asthma with irreversible airway obstruction	401000119107	Asthma

7966291000006118	Intermittent asthma well controlled	641000119106	Asthma
7970131000006117	Intermittent asthma uncontrolled	1741000119102	Asthma
7970171000006119	Acute exacerbation of chronic obstructive airways disease with asthma	1751000119100	Asthma
8031201000006110	Acute exacerbation of asthma co-occurrent with allergic rhinitis	99031000119107	Asthma
8038961000006111	Mild persistent asthma co-occurrent with allergic rhinitis	125011000119100	Asthma
8038971000006116	Intermittent asthma co-occurrent with allergic rhinitis	125021000119107	Asthma
8042571000006111	Acute exacerbation of moderate persistent asthma	135171000119106	Asthma
8042581000006114	Acute exacerbation of mild persistent asthma	135181000119109	Asthma
8555681000006115	Steroid dependent asthma	2360001000004109	Asthma
9314961000006112	Acute severe exacerbation of asthma co-occurrent with allergic rhinitis	10674711000119104	Asthma
9315071000006116	Severe controlled persistent asthma	10675391000119100	Asthma
9315091000006116	Severe persistent allergic asthma	10675431000119106	Asthma
9315111000006112	Acute severe exacerbation of severe persistent asthma co-occurrent with allergic rhinitis	10675551000119104	Asthma
9315171000006116	Severe persistent asthma uncontrolled co-occurrent with allergic rhinitis	10675711000119106	Asthma
9315181000006118	Severe uncontrolled persistent asthma	10675751000119108	Asthma
9315201000006116	Mild persistent allergic asthma	10675871000119106	Asthma
9315221000006110	Acute severe exacerbation of mild persistent allergic asthma	10675911000119108	Asthma
9315231000006112	Acute severe exacerbation of mild persistent allergic asthma co-occurrent with allergic rhinitis	10675991000119100	Asthma
9315291000006112	Mild persistent allergic asthma uncontrolled	10676151000119100	Asthma
9315301000006112	Mild persistent asthma uncontrolled	10676191000119106	Asthma
9315321000006116	Mild persistent asthma uncontrolled co-occurrent with allergic rhinitis	10676231000119102	Asthma
9315351000006112	Moderate persistent allergic asthma	10676391000119108	Asthma
9315371000006120	Acute severe exacerbation of moderate persistent allergic asthma	10676431000119104	Asthma
9315391000006118	Acute severe exacerbation of moderate persistent asthma co-occurrent with allergic rhinitis	10676511000119108	Asthma
9315431000006112	Moderate persistent allergic asthma uncontrolled	10676631000119100	Asthma
9315461000006116	Moderate persistent asthma uncontrolled	10676711000119104	Asthma
9317311000006120	Chronic obstructive asthma co-occurrent with acute exacerbation of asthma	10692721000119102	Asthma
9934311000006112	Oral steroid-dependent asthma	16584951000119100	Asthma
11922851000006116	Acute asthma	304527002	Asthma
12618341000006116	Severe asthma with fungal sensitisation	1103911000000103	Asthma
13619851000006112	Acute severe exacerbation of allergic asthma	782513000	Asthma
13619961000006120	Exacerbation of allergic asthma due to infection	782520007	Asthma
13659841000006116	Near fatal asthma	786836003	Asthma
13775501000006112	Allergic asthma without status asthmaticus	63088003	Asthma
13930321000006112	Thunderstorm asthma	829976001	Asthma
13997031000006116	Intermittent allergic asthma	10674991000119104	Asthma
14504061000006110	Acute asthma	281239006	Asthma
14504071000006116	Asthma attack	281239006	Asthma
9337016	Panlobular emphysema	4981000	COPD
19421011	Interstitial pulmonary emphysema	77690003	COPD
27096010	Giant bullous emphysema	16003001	COPD
55602016	Compensatory emphysema	33325001	COPD
87480013	Chronic tracheobronchitis	52571006	COPD
105519017	Chronic bronchitis	63480004	COPD
113497011	Centrilobular emphysema	68328006	COPD
123588010	Mucopurulent chronic bronchitis	74417001	COPD
139979010	Fetid chronic bronchitis	84409004	COPD
216596014	End stage chronic obstructive airways disease	135836000	COPD
285100019	Emphysematous bronchitis	185086009	COPD
285104011	Obstructive chronic bronchitis	185086009	COPD
301444018	Simple chronic bronchitis NOS	61937009	COPD
301448015	Mucopurulent chronic bronchitis NOS	74417001	COPD
301450011	Chronic asthmatic bronchitis	195949008	COPD
301451010	Chronic wheezy bronchitis	195949008	COPD
301453013	Acute exacerbation of chronic obstructive airways disease	195951007	COPD

301455018	Obstructive chronic bronchitis NOS	185086009	COPD
301456017	Mixed simple and mucopurulent chronic bronchitis	195953005	COPD
301457014	Other chronic bronchitis	63480004	COPD
301458016	Other chronic bronchitis NOS	63480004	COPD
301459012	Chronic bronchitis NOS	63480004	COPD
301460019	Chronic bullous emphysema	195957006	COPD
301463017	Segmental bullous emphysema	195958001	COPD
301464011	Zonal bullous emphysema	195959009	COPD
301468014	Chronic bullous emphysema NOS	195957006	COPD
301469018	Other emphysema	87433001	COPD
301470017	Acute vesicular emphysema	195963002	COPD
301477019	Emphysema NOS	87433001	COPD
301539010	Other specified chronic obstructive airways disease	13645005	COPD
301545019	Chronic obstructive airway disease	13645005	COPD
301572010	Chronic emphysema due to chemical fumes	196026004	COPD
301835010	[X]Other emphysema	87433001	COPD
301836011	[X]Other specified chronic obstructive pulmonary disease	13645005	COPD
396108018	Bullous emphysema with collapse	266355005	COPD
396109014	Atrophic (senile) emphysema	266356006	COPD
396110016	Other emphysema NOS	87433001	COPD
424365019	Acute infective exacerbation of chronic obstructive airways disease	285381006	COPD
457168017	Mild chronic obstructive pulmonary disease	313296004	COPD
457169013	Moderate chronic obstructive pulmonary disease	313297008	COPD
457171013	Severe chronic obstructive pulmonary disease	313299006	COPD
475431013	Chronic obstructive pulmonary disease	13645005	COPD
506053014	Purulent chronic bronchitis	84409004	COPD
508561017	Simple chronic bronchitis	61937009	COPD
1222334016	Other specified chronic obstructive pulmonary disease	13645005	COPD
1222335015	Chronic obstructive pulmonary disease NOS	13645005	COPD
1230190015	MacLeod's unilateral emphysema	45145000	COPD
109301000006114	Tension pneumatocoele	275503004	COPD
457581000006111	Acute interstitial emphysema	77690003	COPD
516801000000112	Very severe chronic obstructive pulmonary disease	293991000000106	COPD
553211000006119	Chron obstruct pulmonary dis wth acute exacerbation unspec	195951007	COPD
555461000006119	Chronic obstructive pulmonary disease with acute lower respiratory infection	196001008	COPD
555471000006114	COAD - Chronic obstructive airways disease	13645005	COPD
640491000006111	Pulmonary emphysema	87433001	COPD
851261000006116	Chronic bronchitis, acute exac	851261000006100	COPD
990641000006110	Chronic obst. pulm. dis. NOS	13645005	COPD
990651000006112	Chr. airway obstruction NOS	13645005	COPD
1823851000006119	Chronic obstructive pulmonary disease confirmed	1823851000006103	COPD
1948051000006112	Asthma-chronic obstructive pulmonary disease overlap syndrome	10692761000119108	COPD
2010061000006113	Acute non-infective exacerbation of chronic obstructive pulmonary disease	847091000000104	COPD
2578881000006118	Panacinar emphysema	4981000	COPD
2716321000006116	COPD - Chronic obstructive pulmonary disease	13645005	COPD
3145221000006111	Obliterative bronchiolitis	40100001	COPD
3228301000006115	Unilateral emphysema	45145000	COPD
3611791000006110	Centriacinar emphysema	68328006	COPD
3764021000006117	Interstitial emphysema of lung	77690003	COPD
3873191000006110	Fetid chronic bronchitis	84409004	COPD
3921361000006112	Emphysema of lung	87433001	COPD
4510801000006114	End stage chronic obstructive pulmonary disease	135836000	COPD
4733031000006114	Chronic obstructive bronchitis	185086009	COPD
4781431000006114	Acute exacerbation of chronic obstructive pulmonary disease	195951007	COPD
4781461000006117	Emphysematous bulla	195957006	COPD
4781471000006112	Bullous emphysema	195957006	COPD
7258581000006114	Acute exacerbation of chronic asthmatic bronchitis	442025000	COPD

8033221000006118	Chronic obstructive lung disease co-occurrent with acute bronchitis	106001000119101	COPD
8090191000006114	Acute exacerbation of chronic obstructive bronchitis	293241000119100	COPD
8287171000006115	Acute non-infective exacerbation of COPD (chronic obstructive pulmonary disease)	847091000000104	COPD
9317331000006112	Asthma-COPD overlap syndrome (ACOS)	10692761000119108	COPD
9317341000006116	ACOS - asthma-chronic obstructive pulmonary disease overlap syndrome	10692761000119108	COPD
11932321000006118	The Global Initiative for Chronic Obstructive Lung Disease (GOLD) classification	11932321000006102	COPD
11932331000006116	COPD GOLD group A	11932331000006104	COPD
11932341000006112	COPD GOLD group B	11932341000006108	COPD
11932351000006110	COPD GOLD group C	11932351000006106	COPD
11932361000006112	COPD GOLD group D	11932361000006108	COPD
12116291000006116	GOLD (Global Initiative for Chronic Obstructive Lung Disease) 2017 group	1097861000000108	COPD
12116301000006116	GOLD (Global Initiative for Chronic Obstructive Lung Disease) 2017 group A	1097871000000101	COPD
12116311000006116	GOLD (Global Initiative for Chronic Obstructive Lung Disease) 2017 group B	1097881000000104	COPD
12116321000006112	GOLD (Global Initiative for Chronic Obstructive Lung Disease) 2017 group C	1097891000000102	COPD
12116331000006112	GOLD (Global Initiative for Chronic Obstructive Lung Disease) 2017 group D	1097901000000101	COPD
13651461000006116	Chronic bronchitis co-occurrent with wheeze	785736001	COPD
2411019	Silicosis	805002	Other respiratory conditions
13455018	Rheumatic pneumonia	7548000	Other respiratory conditions
14610012	Berylliosis	8247009	Other respiratory conditions
20833017	Budgerigar-fanciers' lung	12088005	Other respiratory conditions
21163015	Bronchiectasis	12295008	Other respiratory conditions
24973018	Bauxite fibrosis of lung	14700006	Other respiratory conditions
28128015	Coffee-workers' lung	16623004	Other respiratory conditions
29395011	Graphite fibrosis of lung	17385007	Other respiratory conditions
31510012	Farmers' lung	18690003	Other respiratory conditions
37941013	Asbestosis	22607003	Other respiratory conditions
38677017	Tuberculous bronchiectasis	23022004	Other respiratory conditions
40879015	Pulmonary sarcoidosis	24369008	Other respiratory conditions
49227017	Coal workers' pneumoconiosis	29422001	Other respiratory conditions
67242018	Chronic respiratory failure	39871006	Other respiratory conditions
75302014	Idiopathic fibrosing alveolitis	700250006	Other respiratory conditions
79212010	Simple silicosis	47515009	Other respiratory conditions
83019014	Complicated silicosis	49840000	Other respiratory conditions
85415012	Stannosis	51277007	Other respiratory conditions
85960018	Pulmonary fibrosis	51615001	Other respiratory conditions
85961019	Cirrhosis of lung	266368002	Other respiratory conditions
87088014	Mushroom workers' lung	52333004	Other respiratory conditions
111723011	Bagassosis	67242002	Other respiratory conditions
121482015	Talc pneumoconiosis	73144008	Other respiratory conditions
121968010	Fish-meal workers' lung	73448002	Other respiratory conditions
128792019	Congenital bronchiectasis	77593006	Other respiratory conditions
130626012	Cannabinosis	78723001	Other respiratory conditions
141566014	Pigeon-fanciers' lung	85407005	Other respiratory conditions
142162019	Byssinosis	85761009	Other respiratory conditions
142759013	Cystic fibrosis with meconium ileus	86092005	Other respiratory conditions
142869013	Occupational lung disease	40122008	Other respiratory conditions
178500017	Extrinsic allergic alveolitis	37471005	Other respiratory conditions
189221016	Hypersensitivity pneumonitis	37471005	Other respiratory conditions
196654011	Cryptogenic fibrosing alveolitis	700250006	Other respiratory conditions
264537018	Airways obstruction irreversible	170628003	Other respiratory conditions
287930013	Sarcoidosis of lung with sarcoidosis of lymph nodes	187233002	Other respiratory conditions
293528019	Cystic fibrosis	190905008	Other respiratory conditions
293530017	Fibrocystic disease	190905008	Other respiratory conditions
293532013	Mucoviscidosis	190905008	Other respiratory conditions
293539016	Cystic fibrosis NOS	190905008	Other respiratory conditions
301127010	Obliterating fibrous bronchiolitis	40100001	Other respiratory conditions
301524014	Recurrent bronchiectasis	195984007	Other respiratory conditions

301525010	Post-infective bronchiectasis	195985008	Other respiratory conditions
301526011	Bronchiectasis NOS	12295008	Other respiratory conditions
301527019	Bird-fanciers' lung	69339004	Other respiratory conditions
301528012	Other allergic alveolitis	37471005	Other respiratory conditions
301529016	Pituitary snuff-takers' disease	195989002	Other respiratory conditions
301530014	Air-conditioner and humidifier lung	195990006	Other respiratory conditions
301531013	Other allergic alveolitis NOS	37471005	Other respiratory conditions
301532018	Allergic alveolitis and pneumonitis NOS	37471005	Other respiratory conditions
301533011	Allergic extrinsic alveolitis NOS	37471005	Other respiratory conditions
301534017	Hypersensitivity pneumonitis NOS	37471005	Other respiratory conditions
301552017	Asbestosis NOS	22607003	Other respiratory conditions
301555015	Massive silicotic fibrosis	40640008	Other respiratory conditions
301556019	Silica pneumoconiosis NOS	805002	Other respiratory conditions
301557011	Pneumoconiosis due to inorganic dust	17996008	Other respiratory conditions
301558018	Pneumoconiosis due to inorganic dust NOS	17996008	Other respiratory conditions
301559014	Pneumonopathy due to inhalation of dust	74015002	Other respiratory conditions
301561017	Pneumopathy due to inhalation of other dust NOS	74015002	Other respiratory conditions
301562012	Pneumoconiosis	40122008	Other respiratory conditions
301563019	Pneumoconiosis associated with tuberculosis	196017002	Other respiratory conditions
301571015	Chronic respiratory conditions due to chemical fumes	69454006	Other respiratory conditions
301575012	Chronic pulmonary fibrosis due to chemical fumes	196028003	Other respiratory conditions
301576013	Chronic respiratory conditions due to chemical fumes NOS	50963007	Other respiratory conditions
301609011	Chronic pulmonary radiation disease	196049002	Other respiratory conditions
301610018	Chronic pulmonary radiation disease NOS	196049002	Other respiratory conditions
301611019	Drug-induced interstitial lung disorders	196051003	Other respiratory conditions
301613016	Chronic drug-induced interstitial lung disorders	196053000	Other respiratory conditions
301709019	Idiopathic fibrosing alveolitis NOS	700250006	Other respiratory conditions
301716018	Lung disease with systemic sclerosis	196133001	Other respiratory conditions
301718017	Pulmonary amyloidosis	196135008	Other respiratory conditions
301719013	Lung disease with polymyositis	196136009	Other respiratory conditions
301720019	Lung disease with Sjogren's disease	196137000	Other respiratory conditions
301721015	Lung disease with systemic lupus erythematosus	196138005	Other respiratory conditions
301795015	Chronic pulmonary insufficiency following surgery	196190001	Other respiratory conditions
301838012	[X]Pneumoconiosis due to other dust containing silica	805002	Other respiratory conditions
301839016	[X]Pneumoconiosis due to other specified inorganic dusts	40122008	Other respiratory conditions
301852014	[X]Other interstitial pulmonary diseases with fibrosis	233703007	Other respiratory conditions
311496011	Fibrosing alveolitis associated with rheumatoid arthritis	10713006	Other respiratory conditions
350120014	Asbestos-induced pleural plaque	233659006	Other respiratory conditions
350121013	Pleural plaque disease due to asbestosis	233659006	Other respiratory conditions
350255018	Progressive massive fibrosis	233749003	Other respiratory conditions
451084014	Cystic fibrosis with other manifestations	190905008	Other respiratory conditions
474819014	Cryptogenic organising pneumonia	719218000	Other respiratory conditions
474828010	Bronchiolitis obliterans organising pneumonia	719218000	Other respiratory conditions
492443010	Bronchiolitis obliterans	40100001	Other respiratory conditions
492658013	Idiopathic pulmonary haemosiderosis	40527005	Other respiratory conditions
494669012	Chronic pulmonary oedema	46847001	Other respiratory conditions
499259012	Siderosis	62371005	Other respiratory conditions
508562012	Chronic catarrhal bronchitis	89549007	Other respiratory conditions
1223250017	Sarcoidosis of lung	24369008	Other respiratory conditions
1224939016	Malt workers' lung	25897000	Other respiratory conditions
1227346010	Cystic fibrosis with combined manifestations	190905008	Other respiratory conditions
1233152013	Bird-fancier's lung	69339004	Other respiratory conditions
1235159019	Maple bark strippers' lung	86638007	Other respiratory conditions
1495466012	Cystic fibrosis with no meconium ileus	81423003	Other respiratory conditions
1495490013	Meconium ileus in cystic fibrosis	86092005	Other respiratory conditions
1778239014	Rheumatoid lung disease	398726004	Other respiratory conditions
1786505011	Caplan's syndrome	398640008	Other respiratory conditions
1786545019	Rheumatoid lung	398726004	Other respiratory conditions
2156690018	Cheese-washers' lung	404807005	Other respiratory conditions
2475606014	Usual interstitial pneumonitis	196125002	Other respiratory conditions
2675247013	Liver disease due to cystic fibrosis	427022004	Other respiratory conditions

2676188013	Interstitial lung disease due to collagen vascular disease	427123006	Other respiratory conditions
124281000006119	Suberosis (cork-handlers' lung)	13394002	Other respiratory conditions
141591000006118	Silica and silicate pneumoconiosis	805002	Other respiratory conditions
161781000006116	Sawyer - Jones syndrome	45145000	Other respiratory conditions
162301000006117	Rheumatoid lung	398726004	Other respiratory conditions
183191000006115	Recurrent wheezy bronchitis	195949008	Other respiratory conditions
207831000006116	Interstitial pulmonary fibrosis of prematurity	90610005	Other respiratory conditions
211391000006118	Post-inflammatory pulmonary fibrosis	266368002	Other respiratory conditions
219651000006116	Pneumoconioses	40122008	Other respiratory conditions
269761000006112	Obliterative bronchiolitis due to chemical fumes	196027008	Other respiratory conditions
291961000006112	Ventilation" pneumonitis	48347002	Other respiratory conditions
477131000006116	Allergic alveolitis and pneumonitis NOS	37471005	Other respiratory conditions
556271000006115	Chronic radiation pulmonary fibrosis	71193007	Other respiratory conditions
606441000006110	Cystic fibrosis of the lung	86555001	Other respiratory conditions
618361000000112	Chronic type 1 respiratory failure	428173007	Other respiratory conditions
618371000000117	Chronic type 2 respiratory failure	426896000	Other respiratory conditions
621121000006112	Diffuse interstitial pulmonary fibrosis	196125002	Other respiratory conditions
786581000006114	Idiopathic pulmonary haemosiderosis	40527005	Other respiratory conditions
885281000006118	Mucopurulent chr. bronchitis	74417001	Other respiratory conditions
885331000006110	Coalworker's pneumoconiosis	29422001	Other respiratory conditions
885371000006113	Chronic chemical resp. disease	196025000	Other respiratory conditions
885431000006115	Radiation lung fibrosis	71193007	Other respiratory conditions
885491000006116	Pulmonary fibrosis	51615001	Other respiratory conditions
1174311000000111	Arthropathy in cystic fibrosis	526071000000104	Other respiratory conditions
1174401000000116	Fibrosing colonopathy	445090007	Other respiratory conditions
1709341000006117	Liver disease due to cystic fibrosis	1709341000006101	Other respiratory conditions
1736361000000112	Cystic fibrosis related cirrhosis	776981000000103	Other respiratory conditions
1747711000000111	Interstitial lung disease due to connective tissue disease	711379004	Other respiratory conditions
1763651000006110	Pancreatic sufficient cystic fibrosis	1763651000006106	Other respiratory conditions
1786131000006116	Idiopathic pulmonary fibrosis	700250006	Other respiratory conditions
1854771000006117	Cystic fibrosis manifested by failure to thrive	1854771000006101	Other respiratory conditions
1854791000006116	Cystic fibrosis with liver manifestations	1854791000006100	Other respiratory conditions
1854801000006115	Cystic fibrosis manifested by pancreatitis	1854801000006104	Other respiratory conditions
1854831000006111	Cystic fibrosis manifested by bronchiectasis	1854831000006107	Other respiratory conditions
1854851000006116	Cystic fibrosis manifested by male infertility	1854851000006100	Other respiratory conditions
1854861000006119	Cystic fibrosis manifested by malnutrition	1854861000006103	Other respiratory conditions
1854901000006114	Cystic fibrosis manifested by malabsorption	1854901000006105	Other respiratory conditions
1854911000006112	Cystic fibrosis manifested by nasal polyp/sinus disease	1854911000006108	Other respiratory conditions
2222561000000111	Exacerbation of cystic fibrosis	859041000000103	Other respiratory conditions
2511231000006117	Pneumoconiosis due to silica	805002	Other respiratory conditions
2670341000006112	Diffuse interstitial rheumatoid disease of lung	10713006	Other respiratory conditions
2692491000006117	Budgerigar-fanciers' disease	12088005	Other respiratory conditions
2716231000006114	Chronic obstructive lung disease	13645005	Other respiratory conditions
2716241000006116	Chronic airway obstruction	13645005	Other respiratory conditions
2716261000006117	Chronic airflow obstruction	13645005	Other respiratory conditions
2716271000006112	CAFL - Chronic airflow limitation	13645005	Other respiratory conditions
2716291000006113	CAO - Chronic airflow obstruction	13645005	Other respiratory conditions
2716301000006114	Chronic airflow limitation	13645005	Other respiratory conditions
2716341000006111	Chronic airway disease	13645005	Other respiratory conditions
2716351000006113	COLD - Chronic obstructive lung disease	13645005	Other respiratory conditions
2732761000006118	Bauxite fibrosis of lung	14700006	Other respiratory conditions
2796661000006115	Farmers lung	18690003	Other respiratory conditions
2860341000006111	Asbestos pneumoconiosis	22607003	Other respiratory conditions
2860351000006113	Pulmonary asbestosis	22607003	Other respiratory conditions
2867041000006113	Post-tuberculous bronchiectasis	23022004	Other respiratory conditions
2973161000006118	Coal worker's lung	29422001	Other respiratory conditions
3103601000006113	Allergic alveolitis	37471005	Other respiratory conditions
3152501000006111	IPH - Idiopathic pulmonary hemosiderosis	40527005	Other respiratory conditions
3154151000006111	Massive silicotic fibrosis of lung	40640008	Other respiratory conditions
3228331000006111	Swyer-James syndrome	45145000	Other respiratory conditions
3282201000006119	Humidifier lung	48347002	Other respiratory conditions
3282211000006116	Air conditioner lung	48347002	Other respiratory conditions
3282221000006112	Ventilation pneumonitis	48347002	Other respiratory conditions
3336391000006117	Fibrosis of lung	51615001	Other respiratory conditions

3336411000006117	Cirrhosis of lung	51615001	Other respiratory conditions
3513691000006110	Pulmonary siderosis	62371005	Other respiratory conditions
3513801000006119	Iron pneumoconiosis	62371005	Other respiratory conditions
3657901000006117	Fibrosis of lung following radiation	71193007	Other respiratory conditions
3689711000006118	Pneumoconiosis due to talc	73144008	Other respiratory conditions
3888281000006116	Pigeon-fanciers' disease	85407005	Other respiratory conditions
3888321000006110	Pigeon breeders' lung	85407005	Other respiratory conditions
3907561000006119	Pulmonary cystic fibrosis	86555001	Other respiratory conditions
3955621000006112	Catarrhal bronchitis	89549007	Other respiratory conditions
4760431000006115	CF - Cystic fibrosis	190905008	Other respiratory conditions
4781421000006111	Acute exacerbation of COPD	195951007	Other respiratory conditions
4781831000006111	Toxic bronchiolitis obliterans	196027008	Other respiratory conditions
4782051000006118	Drug-induced interstitial lung disorder	196051003	Other respiratory conditions
4782341000006118	Fibrosing alveolitis	196125002	Other respiratory conditions
4782361000006119	UIP - Usual interstitial pneumonitis	196125002	Other respiratory conditions
4782401000006112	Scleroderma lung disease	196133001	Other respiratory conditions
4782481000006115	Lung disease with Sjögren disease	196137000	Other respiratory conditions
5055241000006116	Complicated pneumoconiosis	233749003	Other respiratory conditions
7065871000006119	Chronic hypercapnic respiratory failure	426896000	Other respiratory conditions
7065881000006116	Chronic type II respiratory failure	426896000	Other respiratory conditions
7084411000006112	Chronic hypoxaemic respiratory failure	428173007	Other respiratory conditions
7084441000006111	Chronic type I respiratory failure	428173007	Other respiratory conditions
7532701000006115	Usual interstitial pneumonia	700250006	Other respiratory conditions
13947281000006118	Chronic respiratory condition caused by vapours	846635004	Other respiratory conditions

Hospital episode statistics: Respiratory disorders	
**icd**	**description**
J40	Bronchitis, not specified as acute or chronic
J41	Simple and mucopurulent chronic bronchitis
J42	Unspecified chronic bronchitis
J43	Emphysema
J44	Other chronic obstructive pulmonary disease
J45	Asthma
J46	Status asthmaticus
J47	Bronchiectasis

Clinical codes for sensory impairments

GOLD: clinical, referral, tests; Aurum: consultations, observations.

Restricted to visual and hearing impairments. Process measures removed – including user of blind dogs, visual aids, hearing aids. Certification of blindness and/or deafness included.

CPRD GOLD: Sensory impairments			
**medcode**	**readcode**	**readterm**	**interpretation**
160	8D36.00	Removable artificial eye	visual impairment
510	6689.00	Registered blind	visual impairment
555	668B.00	Poor visual acuity	visual impairment
1444	F49z.00	Visual loss NOS	visual impairment
1990	F490.00	Blindness, both eyes	visual impairment
2746	6688.00	Registered partially sighted	visual impairment
3268	1B75.00	Loss of vision	visual impairment
3839	22EF.00	O/E - has one eye	visual impairment
3851	F495000	Blindness, one eye, unspecified	visual impairment
3852	2B7A.11	O/E - blind L-eye	visual impairment
3976	F49..13	Partial sight	visual impairment
6020	F49..11	Impaired vision	visual impairment
6661	ZV41000	[V]Problems with sight	visual impairment
6768	6688.11	Registered partially blind	visual impairment
7203	F492.00	Low vision, both eyes	visual impairment
8367	F49..00	Blindness and low vision	visual impairment
9645	F49..14	Sight impaired	visual impairment
10388	2B6A.11	O/E - blind R-eye	visual impairment
10868	F404100	Blind hypotensive eye	visual impairment
12493	F49..12	Low vision	visual impairment
13152	668C.00	Certificate of vision impairment	visual impairment

15269	F495.00	Profound impairment, one eye	visual impairment
18819	F49z000	Charles Bonnet syndrome	visual impairment
20256	F493.00	Visual loss, both eyes unqualified	visual impairment
20585	F4H7.00	Visual cortex disorder	visual impairment
20607	F496000	Low vision, one eye, unspecified	visual impairment
21491	22E6.12	O/E - glass eye	visual impairment
23742	F49z.11	Acquired blindness	visual impairment
25168	F496200	Lesser eye: severe VI, Better eye: near normal vision	visual impairment
25547	F49y.00	Visual loss, one eye, unqualified	visual impairment
25831	F496.00	Low vision, one eye	visual impairment
26722	2B6A.00	O/E-R-eye perceives light only	visual impairment
28993	22E6.11	O/E - false eye	visual impairment
29387	F491000	One eye blind, one eye low vision	visual impairment
31884	22E6.13	O/E - prosthetic eye	visual impairment
32044	SJ0z.11	Blindness - traumatic - NOS	visual impairment
32467	F4H7300	Cortical blindness	visual impairment
33014	2B6B.00	O/E - R-eye completely blind	visual impairment
33015	2B69.00	O/E -R-eye counts fingers only	visual impairment
33016	2B7B.00	O/E - L-eye completely blind	visual impairment
33017	2B7C.00	O/E - L-eye sees hand movements	visual impairment
33447	F492z00	Low vision, both eyes NOS	visual impairment
34957	F4H6100	Visual pathway disorder due to vascular disorder	visual impairment
35515	2B79.00	O/E -L-eye counts fingers only	visual impairment
37086	F404200	Blind hypertensive eye	visual impairment
37893	F496z00	Low vision, one eye NOS	visual impairment
38000	F495A00	Acquired blindness, one eye	visual impairment
38166	2B6W.00	O/E - R-eye visual acuity (corrected) 4/60	visual impairment
38167	2B7A.00	O/E-L-eye perceives light only	visual impairment
38168	2B7W.00	O/E - L-eye visual acuity (corrected) 4/60	visual impairment
39674	F4H..11	Visual path disorder	visual impairment
40451	F492000	Low vision, both eyes unspecified	visual impairment
41060	2B6R.00	O/E - pinhole R-eye perceives light only	visual impairment
42262	F490900	Acquired blindness, both eyes	visual impairment
44954	F495z00	Profound impairment one eye NOS	visual impairment
45030	2B6C.00	O/E - R-eye sees hand movements	visual impairment
45786	2B6T.00	O/E - R-eye visual acuity (corrected) 1/60	visual impairment
46492	F4Hz.00	Disorder of optic nerve or visual pathway NOS	visual impairment
47158	2B6Q.00	O/E - pinhole R-eye counts fingers only	visual impairment
47911	2B6V.00	O/E - R-eye visual acuity (corrected) 2/60	visual impairment
47912	2B7T.00	O/E - L-eye visual acuity (corrected) 1/60	visual impairment
47956	F490z00	Blindness both eyes NOS	visual impairment
48983	2B6P.00	O/E - pinhole R-eye sees hand movements	visual impairment
49283	2B7Q.00	O/E - pinhole L-eye counts fingers only	visual impairment
49336	22E6.00	O/E - glass (prosthetic) eye	visual impairment
50679	F491300	Better eye: severe VI, Lesser eye: near total VI	visual impairment
50925	2B7P.00	O/E - pinhole L-eye sees hand movements	visual impairment
51274	2B6S.00	O/E - pinhole R-eye completely blind	visual impairment
54513	2B7S.00	O/E - pinhole L-eye completely blind	visual impairment
54514	2B7R.00	O/E - pinhole L-eye perceives light only	visual impairment
54962	F491z00	One eye blind, one eye low vision NOS	visual impairment
55108	F490000	Unspecified blindness both eyes	visual impairment
55436	F490100	Both eyes total visual impairment	visual impairment
55844	F491.00	Better eye: low vision, Lesser eye: profound VI	visual impairment
56951	F101200	Spielmeyer-Vogt (Batten) disease	visual impairment
57631	F4H7z00	Visual cortex disorder NOS	visual impairment
60032	2B7V.00	O/E - L-eye visual acuity (corrected) 2/60	visual impairment
60043	F495400	Lesser eye: near total VI, Better eye: unspecified	visual impairment
60184	F4H6.00	Other visual pathway disorder	visual impairment
60401	F494.00	Legal blindness USA	visual impairment
62657	F496500	Lesser eye: moderate VI, Better eye: near normal vision	visual impairment
63045	F495100	Lesser eye: total visual impairment, Better eye: unspecified	visual impairment
63732	FyuL100	[X]Other visual disturbances	visual impairment
63928	F495500	Lesser eye: near total VI, Better eye: near normal vision	visual impairment
65144	F495600	Lesser eye: near total VI, Better eye: normal vision	visual impairment
65569	2B7X.00	O/E - L-eye visual acuity (corrected) 5/60	visual impairment
66372	F4H7100	Visual cortex disorder due to vascular disorder	visual impairment

66391	2B6X.00	O/E - R-eye visual acuity (corrected) 5/60	visual impairment
66731	F492200	Better eye: severe VI, Lesser eye: severe VI	visual impairment
67594	F491400	Better eye: severe VI, Lesser eye: profound VI	visual impairment
67765	F496300	Lesser eye: severe VI, Better eye: normal vision	visual impairment
67872	F496600	Lesser eye: moderate VI, Better eye: normal vision	visual impairment
68386	F492400	Better eye: moderate VI, Lesser eye: severe VI	visual impairment
68391	F491500	Better eye: moderate VI, Lesser eye: blind, unspecified	visual impairment
69998	F495800	Lesser eye: profound VI, Better eye: near normal vision	visual impairment
71319	F492500	Better eye: moderate VI, Lesser eye: moderate VI	visual impairment
72411	F491700	Better eye: moderate VI, Lesser eye: near total VI	visual impairment
73224	F492300	Better eye: moderate VI, Lesser eye: low vision unspecified	visual impairment
73534	F4H6z00	Other visual pathway disorder NOS	visual impairment
96455	F490400	Better eye: near total VI, Lesser eye: near total VI	visual impairment
98637	FyuL.00	[X]Visual disturbances and blindness	visual impairment
98672	F490200	Better eye: near total VI, Lesser eye: unspecified	visual impairment
99165	F495200	Lesser eye: total VI, Better eye: near normal vision	visual impairment
99166	F495300	Lesser eye: total VI, Better eye: normal vision	visual impairment
99191	F490600	Better eye: profound VI, Lesser eye: total VI	visual impairment
99504	F491100	Better eye: severe VI, Lesser eye: blind, unspecified	visual impairment
100755	F496400	Lesser eye: moderate VI, Better eye: unspecified	visual impairment
100999	668D.00	Registered sight impaired	visual impairment
101051	6689.11	Registered severely sight impaired	visual impairment
101881	2BBr.00	Impaired vision due to diabetic retinopathy	visual impairment
103907	Fy1..00	Combined visual and hearing impairment	visual impairment
104077	F49A.00	Blindness, monocular	visual impairment
104087	F496100	Lesser eye: severe VI, Better eye: unspecified	visual impairment
104853	F497.00	Severe visual impairment, binocular	visual impairment
105206	F49B.00	Severe visual impairment, monocular	visual impairment
105493	F49C.00	Moderate visual impairment, monocular	visual impairment
105665	F498.00	Moderate visual impairment, binocular	visual impairment
105752	FyuJ.00	[X]Disorders of optic nerve and visual pathway	visual impairment
108199	F49D.00	Visual impairment	visual impairment
108703	Fy1..12	Deafblind	visual impairment
113993	Fy1..11	Dual sensory impairment - deafblind	visual impairment
412	F59..00	Hearing loss	hearing impairment
467	F59..11	Deafness	hearing impairment
536	F591.00	Sensorineural hearing loss	hearing impairment
686	F59z.00	Deafness NOS	hearing impairment
1151	F590000	Unspecified conductive hearing loss	hearing impairment
1681	1C13100	Unilateral deafness	hearing impairment
1774	1C13200	Partial deafness	hearing impairment
2061	F591000	Unspecified perceptive hearing loss	hearing impairment
2348	F591.13	Perceptive deafness	hearing impairment
2552	F581211	Noise induced deafness	hearing impairment
2625	F591.11	High frequency deafness	hearing impairment
3171	F591211	Nerve deafness	hearing impairment
3747	F593.00	Deaf mutism, NEC	hearing impairment
4035	1C13300	Bilateral deafness	hearing impairment
5149	2BL4.00	O/E - very deaf	hearing impairment
6846	F591200	Neural hearing loss	hearing impairment
7085	2BM2.11	O/E - conductive deafness	hearing impairment
7301	F592100	Mixed conductive and sensorineural hearing loss, bilateral	hearing impairment
7825	2BM3.11	O/E - perceptive deafness	hearing impairment
7891	ZE87.17	HOH - Hard of hearing	hearing impairment
8033	F591.12	Low frequency deafness	hearing impairment
8941	F590.00	Conductive hearing loss	hearing impairment
9830	ZE87.00	Hearing loss	hearing impairment
9882	F592.11	Mixed hearing loss	hearing impairment
10112	F591600	Sensorineural hearing loss, bilateral	hearing impairment
10367	ZE87.11	Deafness	hearing impairment
10665	F591400	Congenital sensorineural deafness	hearing impairment
12324	ZE87.16	HL - Hearing loss	hearing impairment
12339	SJ15.12	Deafness - traumatic - NOS	hearing impairment
12692	ZE87.15	HI - Hearing impairment	hearing impairment
12829	F591.14	Perceptive hearing loss	hearing impairment
13113	2BL3.00	O/E - significantly deaf	hearing impairment

13114	2BL..11	O/E - deaf	hearing impairment
13115	2BL2.00	O/E - slightly deaf	hearing impairment
14805	1C13.00	Deafness	hearing impairment
14893	A560200	Rubella deafness	hearing impairment
14983	F592.00	Mixed conductive and sensorineural deafness	hearing impairment
15940	F581200	Noise-induced hearing loss	hearing impairment
16393	F591z00	Perceptive hearing loss NOS	hearing impairment
16648	F59z.11	Chronic deafness	hearing impairment
18008	ZE87.18	Hearing impairment	hearing impairment
18520	F591100	Sensory hearing loss	hearing impairment
18945	F590500	Conductive hearing loss, bilateral	hearing impairment
19068	ZE87.19	Hearing impaired	hearing impairment
19084	F594.00	High frequency deafness	hearing impairment
20715	2BM4.00	O/E - High tone deafness	hearing impairment
22102	ZE87.13	Hard of hearing	hearing impairment
26539	2BL5.00	O/E - completely deaf	hearing impairment
30033	ZE87.20	Hearing impaired	hearing impairment
30355	2BM3.00	O/E tune fork=perceptive deaf	hearing impairment
31374	P40z.11	Deafness due to congenital anomaly NEC	hearing impairment
33583	F590z00	Conductive hearing loss NOS	hearing impairment
38563	F59y.00	Other specified forms of hearing loss	hearing impairment
42893	2BM2.00	O/E -tune fork=conductive deaf	hearing impairment
44282	F595.00	Low frequency deafness	hearing impairment
45751	F590.11	Conductive deafness	hearing impairment
47440	F591500	Ototoxicity - deafness	hearing impairment
53800	FyuU100	[X]Other specified hearing loss	hearing impairment
54116	F591y00	Combined perceptive hearing loss	hearing impairment
62906	F590y00	Combined conductive hearing loss	hearing impairment
69894	F591300	Central hearing loss	hearing impairment
71827	FyuU000	[X]Deaf mutism, not elsewhere classified	hearing impairment
96245	F5A..00	Hearing impairment	hearing impairment
98253	F596.00	Maternally inherited deafness	hearing impairment
100127	F598.00	Moderate acquired hearing loss	hearing impairment
100276	F599.00	Severe acquired hearing loss	hearing impairment
100654	F59A.00	Profound acquired hearing loss	hearing impairment
100736	F591800	Congenital prelingual deafness	hearing impairment
102069	F591511	Drug ototoxicity - deafness	hearing impairment
103907	Fy1..00	Combined visual and hearing impairment	hearing impairment
106927	PKyP.00	Diab insipidus,diab mell,optic atrophy and deafness	hearing impairment
107323	F591A00	Bilateral congenital sensorineural hearing loss	hearing impairment
107350	F591C00	Moderate sensorineural hearing loss	hearing impairment
107364	F591900	Bilateral profound sensorineural hearing loss	hearing impairment
107607	F591B00	Profound sensorineural hearing loss	hearing impairment
107610	F591E00	Severe sensorineural hearing loss	hearing impairment
108703	Fy1..12	Deafblind	hearing impairment
113993	Fy1..11	Dual sensory impairment - deafblind	hearing impairment
115285	PKyz800	DOOR - Deafness, triphalangeal thumbs, onychodystrophy	hearing impairment

CPRD Aurum: Sensory impairments			
medcode	term	snomedconceptID	interpretation
1529013	Blind hypertensive eye	264008	visual impairment
14191011	Impaired vision	397540003	visual impairment
113901010	Cortical blindness	68574006	visual impairment
253776012	O/E - false eye	162812004	visual impairment
253777015	O/E - prosthetic eye	162812004	visual impairment
253778013	O/E - glass eye	162812004	visual impairment
253779017	O/E - glass (prosthetic) eye	162812004	visual impairment
253791014	O/E - has one eye	162821003	visual impairment
255271016	O/E -R-eye counts fingers only	163944007	visual impairment
255275013	O/E - blind R-eye	163946009	visual impairment
255276014	O/E - R-eye completely blind	163946009	visual impairment
255288011	O/E -L-eye counts fingers only	163958009	visual impairment
255292016	O/E - blind L-eye	163960006	visual impairment
255293014	O/E - L-eye completely blind	163960006	visual impairment
264661019	Registered blind	170727003	visual impairment
264662014	Poor visual acuity	170728008	visual impairment

298252011	Unspecified blindness both eyes	193699007	visual impairment
298262016	Blindness both eyes NOS	193699007	visual impairment
298264015	One eye blind, one eye low vision	193712007	visual impairment
298276015	One eye blind, one eye low vision NOS	193711000	visual impairment
298277012	Low vision, both eyes	193722001	visual impairment
298278019	Low vision, both eyes unspecified	193722001	visual impairment
298284016	Low vision, both eyes NOS	193722001	visual impairment
298285015	Blind or low vision - both eyes	267727004	visual impairment
298286019	Legal blindness USA	193731001	visual impairment
298287011	Profound impairment, one eye	193732008	visual impairment
298288018	Blindness of one eye	22950006	visual impairment
298289014	Lesser eye: total visual impairment, Better eye: unspecified	193732008	visual impairment
298299016	Profound impairment one eye NOS	193732008	visual impairment
298300012	Low vision, one eye	193745007	visual impairment
298301011	Low vision, one eye, unspecified	193745007	visual impairment
298308017	Low vision, one eye NOS	193745007	visual impairment
298309013	Blind or low vision - one eye only	267728009	visual impairment
298313018	Charles Bonnet syndrome	193756007	visual impairment
298761010	Other visual pathway disorder	54767005	visual impairment
298763013	Visual pathway disorder due to vascular disorder	194064000	visual impairment
298768016	Other visual pathway disorder NOS	54767005	visual impairment
298770013	Visual cortex disorder due to vascular disorder	194069005	visual impairment
298772017	Visual cortex disorder NOS	128329001	visual impairment
299508012	[X]Other visual disturbances	63102001	visual impairment
347842017	Acquired blindness	105597003	visual impairment
399455016	Visual loss NOS	240311000000103	visual impairment
402527016	O/E-R-eye perceives light only	268973006	visual impairment
402528014	O/E-L-eye perceives light only	268974000	visual impairment
442202014	Registered partially sighted	300994001	visual impairment
451387013	O/E - R-eye sees hand movements	308082007	visual impairment
451388015	O/E - L-eye sees hand movements	308083002	visual impairment
456798010	Acquired blindness, both eyes	193709009	visual impairment
456799019	Acquired blindness, one eye	22950006	visual impairment
460633016	[V]Problems with sight	397540003	visual impairment
474370011	Visual cortex disorder	128329001	visual impairment
493267010	Blind hypotensive eye	4229009	visual impairment
2163955016	O/E - pinhole L-eye sees hand movements	408315006	visual impairment
2163956015	O/E - pinhole L-eye counts fingers only	408316007	visual impairment
2163957012	O/E - pinhole L-eye perceives light only	408317003	visual impairment
2163958019	O/E - pinhole L-eye completely blind	408318008	visual impairment
2163959010	O/E - pinhole R-eye sees hand movements	408319000	visual impairment
2163960017	O/E - pinhole R-eye counts fingers only	408320006	visual impairment
2163961018	O/E - pinhole R-eye perceives light only	408321005	visual impairment
2163962013	O/E - pinhole R-eye completely blind	408322003	visual impairment
2163963015	O/E - R-eye visual acuity (corrected) 1/60	408323008	visual impairment
2163964014	O/E - R-eye visual acuity (corrected) 2/60	408324002	visual impairment
2163965010	O/E - R-eye visual acuity (corrected) 4/60	408325001	visual impairment
2163966011	O/E - R-eye visual acuity (corrected) 5/60	408326000	visual impairment
2163968012	O/E - L-eye visual acuity (corrected) 1/60	408328004	visual impairment
2163969016	O/E - L-eye visual acuity (corrected) 2/60	408329007	visual impairment
2163970015	O/E - L-eye visual acuity (corrected) 4/60	408330002	visual impairment
2163971016	O/E - L-eye visual acuity (corrected) 5/60	408331003	visual impairment
2164154014	Low vision	7973008	visual impairment
2164155010	Sight impaired	397540003	visual impairment
2164157019	Visual impairment	397540003	visual impairment
2164158012	Partial sight	7973008	visual impairment
2538048016	Removable artificial eye	414928009	visual impairment
2619503012	Both eyes total visual impairment	193699007	visual impairment
2773231012	Combined visual and hearing impairment	433147009	visual impairment
17791000000110	Registered partially blind	2531000000100	visual impairment
61901000006116	Visual path disorder	54767005	visual impairment
308101000000115	Certificate of vision impairment	727391000000102	visual impairment
510181000006115	Better eye: low vision, Lesser eye: profound visual impairment	193711000	visual impairment
510191000006117	Better eye: moderate VI, Lesser eye: blind, unspecified	193711000	visual impairment
510201000006119	Better eye: moderate VI, Lesser eye: low vision unspecified	193722001	visual impairment

510211000006116	Better eye: moderate visual impairment, Lesser eye: moderate visual impairment	193728002	visual impairment
510221000006112	Better eye: moderate visual impairment, Lesser eye: near total visual impairment	193719003	visual impairment
510231000006110	Better eye: moderate visual impairment, Lesser eye: profound visual impairment	193720009	visual impairment
510241000006117	Better eye: moderate visual impairment, Lesser eye: severe visual impairment	193727007	visual impairment
510251000006115	Better eye: moderate visual impairment, Lesser eye: total visual impairment	193718006	visual impairment
510261000006118	Better eye: near total visual impairment, Lesser eye: near total visual impairment	193704004	visual impairment
510271000006113	Better eye: near total visual impairment, Lesser eye: total visual impairment	193703005	visual impairment
510281000006111	Better eye: near total VI, Lesser eye: unspecified	193699007	visual impairment
510291000006114	Better eye: profound visual impairment, Lesser eye: near total visual impairment	193707006	visual impairment
510301000006110	Better eye: profound visual impairment, Lesser eye: profound visual impairment	193708001	visual impairment
510331000006119	Better eye: severe VI, Lesser eye: blind, unspecified	193711000	visual impairment
510361000006111	Better eye: severe visual impairment, Lesser eye: profound visual impairment	193716005	visual impairment
510371000006116	Better eye: severe visual impairment, Lesser eye: severe visual impairment	193725004	visual impairment
510381000006118	Better eye: severe visual impairment, Lesser eye: total visual impairment	193714008	visual impairment
515781000006114	Blindness - traumatic - NOS	212141007	visual impairment
515791000006112	Blindness and low vision	397540003	visual impairment
515811000006111	Blindness - both eyes	193699007	visual impairment
733161000000116	Impaired vision due to diabetic retinopathy	373041000000101	visual impairment
734661000006116	Loss of vision	240311000000103	visual impairment
734951000006114	Lesser eye: near total visual impairment, Better eye: normal vision	111519001	visual impairment
747311000006117	Lesser eye: moderate visual impairment, Better eye: near normal vision	193751002	visual impairment
747331000006111	Lesser eye: moderate VI, Better eye: unspecified	193745007	visual impairment
747341000006118	Lesser eye: near total visual impairment, Better eye: near normal vision	193738007	visual impairment
747351000006116	Lesser eye: near total VI, Better eye: unspecified	193732008	visual impairment
747361000006119	Lesser eye: profound visual impairment, Better eye: near normal vision	193741003	visual impairment
747371000006114	Lesser eye: profound visual impairment, Better eye: normal vision	193742005	visual impairment
747391000006110	Lesser eye: severe visual impairment, Better eye: near normal vision	193748009	visual impairment
747401000006112	Lesser eye: severe visual impairment, Better eye: normal vision	193749001	visual impairment
747411000006110	Lesser eye: severe VI, Better eye: unspecified	193745007	visual impairment
747421000006119	Lesser eye: total visual impairment, Better eye: near normal vision	193735005	visual impairment
747431000006116	Lesser eye: total visual impairment, Better eye: normal vision	193736006	visual impairment
855291000006110	Blind (subjectively)	855291000006106	visual impairment
857631000006118	Vision - blind despite any aid	857631000006102	visual impairment
883361000006117	Blind/low vision - both eyes	193699007	visual impairment
883371000006112	Blind - both eyes	193699007	visual impairment
883381000006110	Blind/low vision -one eye only	193745007	visual impairment
883391000006113	Blindness/low vision NOS	566991000000106	visual impairment
915071000006116	Visual impairment: difficulty using stairs	915071000006100	visual impairment
915081000006118	Visual impairment: avoids driving	915081000006102	visual impairment
988991000006111	Blind/low vision - both eyes	267727004	visual impairment
989001000006111	Blind/low vision -one eye only	267728009	visual impairment
1563201000000117	Registered sight impaired	713031000000100	visual impairment
1564221000000111	Registered severely sight impaired	170727003	visual impairment
1816041000006111	Blindness, monocular	22950006	visual impairment
2168181000000116	Severe visual impairment, binocular	813871000000108	visual impairment
2168191000000119	Severe visual impairment, monocular	813881000000105	visual impairment
2168201000000117	Moderate visual impairment, binocular	813891000000107	visual impairment
2168211000000115	Moderate visual impairment, monocular	813901000000108	visual impairment
2267571000000111	Deafblind	433147009	visual impairment

2267601000000116	Dual sensory impairment - deafblind	433147009	visual impairment
2627081000006116	Sight impaired	7973008	visual impairment
2627141000006114	Poor vision	7973008	visual impairment
2865981000006110	Blind eye	22950006	visual impairment
2877491000006115	Traumatic blindness	23653003	visual impairment
3572471000006112	Legal blindness	65956007	visual impairment
3930491000006117	Transient monocular blindness	88032003	visual impairment
4057501000006117	Congenital blindness	95486002	visual impairment
4121051000006114	Blindness	105597003	visual impairment
4771631000006115	Congenital retinal blindness	193413001	visual impairment
4943361000006111	Painful blind eye	225569001	visual impairment
5192741000006116	Blind registration	243886006	visual impairment
5564611000006114	Blind left eye	274571007	visual impairment
5564631000006115	Blind right eye	274572000	visual impairment
5999711000006112	Blindness certification	310418000	visual impairment
6588751000006112	Severe visual impairment	397541004	visual impairment
6588761000006114	Moderate visual impairment	397542006	visual impairment
6760581000006111	O/E - pinhole left eye completely blind	408318008	visual impairment
6760701000006113	O/E - pinhole right eye completely blind	408322003	visual impairment
6849021000006119	Cortical visual impairment	413924001	visual impairment
7973421000006117	England and Wales blind certification	2481000000102	visual impairment
7973431000006119	Certification of blindness - England and Wales	2481000000102	visual impairment
8068071000006111	Bilateral visual impairment	218951000119100	visual impairment
8110491000006111	Blind right eye, normal vision left eye	343021000119100	visual impairment
8110501000006115	Blind right eye, low vision left eye	343031000119102	visual impairment
8110511000006117	Blind left eye, normal vision right eye	343041000119106	visual impairment
8110521000006113	Blind left eye, low vision right eye	343051000119108	visual impairment
8262581000006110	Severe binocular visual impairment	813871000000108	visual impairment
8262601000006117	Severe monocular visual impairment	813881000000105	visual impairment
8262621000006110	Moderate binocular visual impairment	813891000000107	visual impairment
8262641000006115	Moderate monocular visual impairment	813901000000108	visual impairment
11998211000006114	Bilateral acquired blindness of eyes	193709009	visual impairment
12027631000006118	Deaf-blind	765178008	visual impairment
25785014	Deafness	15188001	hearing impairment
25788011	Hearing loss	15188001	hearing impairment
70962017	Low frequency deafness	42538001	hearing impairment
73470015	Conductive hearing loss	44057004	hearing impairment
73473018	Conductive deafness	44057004	hearing impairment
100850012	Sensorineural hearing loss	60700002	hearing impairment
100854015	Sensorineural deafness	60700002	hearing impairment
100856018	Perceptive hearing loss	60700002	hearing impairment
100857010	Perceptive deafness	60700002	hearing impairment
113719012	Central hearing loss	68467004	hearing impairment
121849011	Neural hearing loss	73371001	hearing impairment
121916015	Noise-induced hearing loss	73415002	hearing impairment
141844010	Sensory hearing loss	85571008	hearing impairment
253161014	Unilateral deafness	162342008	hearing impairment
253163012	Bilateral deafness	162344009	hearing impairment
255425013	O/E - slightly deaf	164060004	hearing impairment
255426014	O/E - significantly deaf	164061000	hearing impairment
255427017	O/E - very deaf	164062007	hearing impairment
255428010	O/E - completely deaf	164063002	hearing impairment
255433014	O/E -tune fork=conductive deaf	164068006	hearing impairment
255434015	O/E - conductive deafness	44057004	hearing impairment
255435019	O/E - perceptive deafness	164069003	hearing impairment
255436018	O/E tune fork=perceptive deaf	164069003	hearing impairment
255437010	O/E - High tone deafness	164070002	hearing impairment
286780017	Rubella deafness	186570004	hearing impairment
299240012	Unspecified conductive hearing loss	44057004	hearing impairment
299245019	Conductive hearing loss, bilateral	194417009	hearing impairment
299247010	Combined conductive hearing loss	194419007	hearing impairment
299248017	Conductive hearing loss NOS	44057004	hearing impairment
299255015	Unspecified perceptive hearing loss	60700002	hearing impairment
299259014	Sensorineural hearing loss, bilateral	194424005	hearing impairment
299261017	Combined perceptive hearing loss	194426007	hearing impairment
299262012	Perceptive hearing loss NOS	60700002	hearing impairment

299264013	Mixed conductive and sensorineural hearing loss, bilateral	194429000	hearing impairment
299265014	Deaf mutism	61947007	hearing impairment
299266010	Other specified forms of hearing loss	118230007	hearing impairment
299550012	[X]Deaf mutism, not elsewhere classified	61947007	hearing impairment
299551011	[X]Other specified hearing loss	15188001	hearing impairment
348075014	Chronic deafness	232325008	hearing impairment
348076010	High frequency deafness	232326009	hearing impairment
354552014	Renal tubular acidosis with progressive nerve deafness	236532003	hearing impairment
356110010	Megaloblastic anaemia, thiamine-responsive, with diabetes mellitus and sensorineural deafness	237617006	hearing impairment
356118015	Diabetes-deafness syndrome maternally transmitted	237619009	hearing impairment
358234016	DOOR - Deafness, triphalangeal thumbs, onychodystrophy	239047003	hearing impairment
377153018	Deafness due to congenital anomaly NEC	204224006	hearing impairment
399507018	Ototoxicity - deafness	267677007	hearing impairment
399508011	Deafness NOS	272033007	hearing impairment
411470013	Drug ototoxicity - deafness	275482009	hearing impairment
411844019	O/E - deaf	275879008	hearing impairment
470343013	Partial deafness	343087000	hearing impairment
477221017	Hearing impaired	15188001	hearing impairment
502417018	Nerve deafness	73371001	hearing impairment
512317011	Congenital sensorineural deafness	95828007	hearing impairment
1234118017	Mixed conductive and sensorineural deafness	77507001	hearing impairment
1234119013	Mixed hearing loss	77507001	hearing impairment
1234121015	Mixed deafness	77507001	hearing impairment
2773231012	Combined visual and hearing impairment	433147009	hearing impairment
3527478013	Congenital conductive hearing loss	737344003	hearing impairment
148551000006115	Sensorineural hearing loss, unilateral with unrestricted hearing on the contralateral side	194425006	hearing impairment
578361000006118	Conductive hearing loss, unilateral with unrestricted hearing on the contralateral side	194418004	hearing impairment
587171000006119	Noise induced deafness	73415002	hearing impairment
608101000006118	Deafness	15188001	hearing impairment
608121000006111	Deafness - traumatic - NOS	9441002	hearing impairment
622201000000110	Maternally inherited deafness	95828007	hearing impairment
700951000006112	Mixed conductive and sensorineural hearing loss, unilateral with unrestricted hearing on the contralateral side	194428008	hearing impairment
733941000006114	Low frequency deafness	42538001	hearing impairment
822981000006112	High frequency deafness	232326009	hearing impairment
883891000006118	Deaf mutism NOS	694231000000100	hearing impairment
883901000006119	Hearing loss NOS	679391000000103	hearing impairment
918681000006119	Deafness	918681000006103	hearing impairment
918701000006116	Unilateral deafness	918701000006100	hearing impairment
918711000006118	Partial deafness	918711000006102	hearing impairment
918721000006114	Bilateral deafness	918721000006105	hearing impairment
982621000006118	Hearing impaired	982621000006102	hearing impairment
989081000006119	Hearing loss NOS	15188001	hearing impairment
1173591000000110	Congenital prelingual deafness	699238006	hearing impairment
1173631000000110	Moderate acquired hearing loss	737048006	hearing impairment
1173651000000115	Severe acquired hearing loss	737049003	hearing impairment
1173671000000112	Profound acquired hearing loss	737050003	hearing impairment
1846681000006117	Amyloid nephropathy with deafness and urticaria	15123008	hearing impairment
1848531000006116	Diabetes insipidus, diabetes mellitus, optic atrophy and deafness	70694009	hearing impairment
2226281000000117	Moderate sensorineural hearing loss	860801000000109	hearing impairment
2226301000000116	Severe sensorineural hearing loss	860811000000106	hearing impairment
2226361000000117	Profound sensorineural hearing loss	700454004	hearing impairment
2264121000000111	Bilateral profound sensorineural hearing loss	877191000000104	hearing impairment
2264161000000115	Bilateral congenital sensorineural hearing loss	877211000000100	hearing impairment
2267571000000111	Deafblind	433147009	hearing impairment
2267601000000116	Dual sensory impairment - deafblind	433147009	hearing impairment
2559891000006111	Inner ear conductive hearing loss	3820005	hearing impairment
2636241000006117	Complete deafness	8531006	hearing impairment
2740721000006112	Hard of hearing	15188001	hearing impairment
2740731000006110	Hearing impairment	15188001	hearing impairment
2740761000006118	HL - Hearing loss	15188001	hearing impairment
2740771000006113	HI - Hearing impairment	15188001	hearing impairment
2985711000006110	Functional hearing loss	30169000	hearing impairment

2985721000006119	FHL - Functional hearing loss	30169000	hearing impairment
3209511000006111	CHL - Conductive hearing loss	44057004	hearing impairment
3243751000006113	Bone conduction deafness	46050004	hearing impairment
3289101000006117	Upper frequency deafness	48758008	hearing impairment
3289111000006119	High frequency hearing loss	48758008	hearing impairment
3439761000006114	Retinitis pigmentosa-deafness syndrome	57838006	hearing impairment
3486051000006114	Neurosensory deafness	60700002	hearing impairment
3486081000006118	SND - Sensorineural deafness	60700002	hearing impairment
3486091000006115	Sensory-neural deafness	60700002	hearing impairment
3486101000006114	Sensory-neural hearing loss	60700002	hearing impairment
3486111000006112	SNHL - Sensorineural hearing loss	60700002	hearing impairment
3503111000006111	Middle ear conductive hearing loss	61743004	hearing impairment
3649791000006119	Diabetes mellitus AND insipidus with optic atrophy AND deafness	70694009	hearing impairment
3693771000006111	NIHL - Noise-induced hearing loss	73415002	hearing impairment
3761211000006116	Mixed conductive AND sensorineural hearing loss	77507001	hearing impairment
3761251000006115	MHL - Mixed hearing loss	77507001	hearing impairment
3890851000006110	Cochlear hearing loss	85571008	hearing impairment
3890861000006112	Inner ear hearing loss	85571008	hearing impairment
4062721000006113	Bilateral hearing loss	95820000	hearing impairment
4062831000006115	Congenital deafness	95828007	hearing impairment
4574411000006111	O/E - conductive deafness	164068006	hearing impairment
4775301000006119	Unilateral conductive hearing loss with unrestricted hearing on the contralateral side	194418004	hearing impairment
4775341000006117	Unilateral sensorineural hearing loss with unrestricted hearing on the contralateral side	194425006	hearing impairment
4775371000006113	Unilateral mixed conductive and sensorineural hearing loss with unrestricted hearing on the contralateral side	194428008	hearing impairment
5034811000006119	X-linked sensorineural hearing loss	232329002	hearing impairment
5034821000006110	Recessive sensorineural hearing loss	232330007	hearing impairment
5034841000006115	Postnatal acquired sensorineural hearing loss	232332004	hearing impairment
5034851000006118	Hearing loss associated with syndrome	232333009	hearing impairment
5095461000006114	Prune belly syndrome with pulmonic stenosis, mental retardation and deafness	236529001	hearing impairment
7046801000006112	Unilateral neural hearing loss	425601005	hearing impairment
7052361000006119	Unilateral sensory hearing loss	425980006	hearing impairment
7078511000006111	Asymmetrical hearing loss	427772009	hearing impairment
7078521000006115	Asymmetric hearing loss	427772009	hearing impairment
7095141000006116	Asymmetrical sensorineural hearing loss	428887009	hearing impairment
7095151000006119	Asymmetric sensorineural hearing loss	428887009	hearing impairment
7129341000006117	Bilateral neural hearing loss	430977001	hearing impairment
7129481000006119	Bilateral sensory hearing loss	430985005	hearing impairment
7265651000006117	Unilateral conductive hearing loss	442535004	hearing impairment
7487941000006119	Deafness of right ear	473421004	hearing impairment
7487951000006117	Deafness of left ear	473422006	hearing impairment
7487961000006115	Hearing loss of right ear	473423001	hearing impairment
7487971000006110	Hearing loss of left ear	473424007	hearing impairment
7493431000006112	Acquired sensorineural hearing loss	609125008	hearing impairment
7535361000006110	Congenital sensorineural hearing loss	700453005	hearing impairment
7811761000006116	Deafness and intellectual disability Martin Probst type syndrome	721087008	hearing impairment
7815121000006111	Acquired hearing loss	721294001	hearing impairment
7850381000006114	Retinitis pigmentosa, intellectual disability, deafness, hypogenitalism syndrome	724001005	hearing impairment
7854191000006115	HDR (hypoparathyroidism, sensorineural deafness, renal disease) syndrome	724282009	hearing impairment
7953161000006117	Deafness, small bowel diverticulosis, neuropathy syndrome	733071009	hearing impairment
7976771000006115	Severe hearing loss	3561000119106	hearing impairment
8094261000006115	Registered deaf	298651000000102	hearing impairment
8094571000006113	Registered hearing impaired	299201000000108	hearing impairment
8317121000006110	Bilateral severe sensorineural hearing loss	898061000000101	hearing impairment
8317141000006115	Bilateral moderate sensorineural hearing loss	898071000000108	hearing impairment
11902971000006110	Unilateral deafness	162342008	hearing impairment
12027631000006118	Deaf-blind	765178008	hearing impairment
13843791000006114	Congenital sensorineural deafness	700453005	hearing impairment
14160411000006116	Conductive hearing loss of right ear	1010236009	hearing impairment
14160441000006116	Conductive hearing loss of left ear	1010238005	hearing impairment

14161921000006110	Conductive hearing loss of left ear with normal hearing on right side	1010439000	hearing impairment
14161931000006112	Conductive hearing loss of right ear with normal hearing on left side	1010440003	hearing impairment
14161941000006116	Sensorineural hearing loss of right ear with normal hearing on left side	1010441004	hearing impairment
14161951000006118	Sensorineural hearing loss of left ear with normal hearing on right side	1010442006	hearing impairment
14165181000006112	Mixed conductive and sensorineural hearing loss of left ear with normal hearing on right side	1052205008	hearing impairment
14165191000006112	Mixed conductive and sensorineural hearing loss of right ear with normal hearing on left side	1052206009	hearing impairment
14169481000006116	Sensorineural hearing loss of right ear	1119386008	hearing impairment
14169491000006116	Sensorineural hearing loss of left ear	1119387004	hearing impairment

Hospital episode statistics: Sensory impairments		
**icd**	**Description**	**interpretation**
H54	Visual impairment including blindness	visual impairment
H90	Conductive and sensorinueral hearing loss	hearing impairment
H91.0	Ototoxic hearing loss	hearing impairment
H91.2	Sudden idiopathic hearing loss	hearing impairment
H91.3	Deaf mutism, not elsewhere classified	hearing impairment
H93.0	Transient ischaemic deafness	hearing impairment

Table S4: Clinical codes for causes of death

icd	description
A00–B99	Infections/parasitic diseases
C00–D48	All Cancers
C18, C19, C20	Cancers – colorectal
C34	Cancers – lung
C50, D05	Cancers – breast
C00-D48 (other than breast, colorectal, lung)	Cancers – other
E00–E90	Endocrine, nutritional or metabolic disorders
F00–F04	Mental and behavioural disorders – Dementia
F00–F99	Mental and behavioural disorders:
F00–F99 (other than dementia)	Mental and behavioural disorders: other
G00–G99	Diseases of the central nervous system:
G00–G99 (other than epilepsy)	Diseases of the central nervous system: Other
G40,G41	Diseases of the central nervous system - epilepsy:
I00–I99	Circulatory system diseases
I20–I25	Circulatory system diseases - IHD:
I60–I69	Circulatory system diseases - Cerebrovascular:
I00–I99 (other than IHD, cerebrovascular)	Circulatory system diseases: other
J00–J99	Respiratory diseases:
J00-J99 (other than aspiration pneumonia)	Respiratory diseases – other
J69	Respiratory diseases - aspiration pneumonia
K00–K93	Digestive system disorders
M00–M99	Musculoskeletal disorders
N00–N99	Genitourinary disorders
Q00–Q99	Congenital/Chromosomal conditions
S00–X59	Injuries/Accidents
D50–D89, H00–H89, P91, R00–R99, X69–X99, Y00–Y89	Others

Table S5: Clinical codes for primary care consultations and referrals

Clinical codes for face-to-face consultations

For Aurum consultations, rows highlighted in grey indicate that the visit was by a practice nurse before identifying the staff role.

CPRD GOLD: Face-to-face consultations		
**Code**	**Description**	**Interpretation**
1	Clinic	Face-to-face/telephone/video consultation
2	Night visit/Deputising service	Face-to-face/telephone/video consultation
3	Follow-up/routine visit	Face-to-face/telephone/video consultation
4	Night visit, Local rota	Face-to-face/telephone/video consultation
6	Night visit, practice	Face-to-face/telephone/video consultation
7	Out of hours, Practice	Face-to-face/telephone/video consultation
8	Out of hours, Non Practice	Face-to-face/telephone/video consultation
9	Surgery consultation	Face-to-face/telephone/video consultation
10	Telephone call from a patient	Face-to-face/telephone/video consultation
11	Acute visit	Face-to-face/telephone/video consultation
18	Emergency Consultation	Face-to-face/telephone/video consultation
21	Telephone call to a patient	Face-to-face/telephone/video consultation
24	Children's home visit	Face-to-face/telephone/video consultation
27	Home Visit	Face-to-face/telephone/video consultation
28	Hotel Visit	Face-to-face/telephone/video consultation
30	Nursing Home Visit	Face-to-face/telephone/video consultation
31	Residential Home Visit	Face-to-face/telephone/video consultation
32	Twilight Visit	Face-to-face/telephone/video consultation
33	Triage	Face-to-face/telephone/video consultation
34	Walk-in Centre	Face-to-face/telephone/video consultation
50	Night Visit	Face-to-face/telephone/video consultation
55	Telephone Consultation	Face-to-face/telephone/video consultation

CPRD Aurum: Face-to-face consultations		
**medcode**	**Term**	**snomedCTConceptId**

34111000000115	Out of hours consultation at surgery	14161000000107
34361000000116	Weekend consultation at surgery	14351000000106
34771000000112	Bank holiday surgery consultation	14601000000102
62151000000116	Telephone consultation	386472008
189731000000110	Consultation for minor injury	113011000000100
407021000000116	Follow-up consultation for minor injury	248491000000101
958171000006116	Consultation for minor injury	958171000006100
285405016	New patient consultation	185387006
418934011	Follow-up consultation	281036007
600831000000112	Consultation via multimedia	325911000000101
600851000000117	Consultation via video conference	325921000000107
1161431000000116	Telephone consultation for suspected swine flu	520141000000108
19773013	Consultation	11429006
1672851000006116	Face to face consultation	1672851000006100
1672881000006112	Face to face consultation with relative/carer	1672881000006108
1771751000006112	Consultation for minor illness	1771751000006108
1742101000006116	Telephone consultation for suspected influenza A virus subtype H1N1	520141000000108
1778971000006110	Consultation via telemedicine web camera	1778971000006106
1778981000006113	Consultation via Typetalk relay service	1778981000006109
1805381000006112	1800 IM SICK telephone consultation	1805381000006108
1809161000006119	Urgent consultation	1809161000006103
1809171000006114	Routine consultation	1809171000006105
1779001000006111	Other consultation medium used	1779001000006107
1778991000006111	Consultation via SMS text message	1778991000006107
1809181000006112	Emergency consultation	1809181000006108
1809191000006110	Extended hours consultation	1809191000006106
1823481000006113	Acrimonious doctor/patient consultation	1823481000006109
1849981000006119	Follow up telephone consultation	1849981000006103
1849991000006116	First telephone consultation	1849991000006100
1875321000006115	Pre-referral CAMHS consultation	1875321000006104
1876881000006117	Satellite surgery consultation	1876881000006101
1908141000006115	Joint consultation - General Practitioner registrar and General Practitioner trainer	903041000000104
1934711000006116	Extended consultation for multiple long term conditions	1934711000006100
1935971000006114	Extended consultation for cancer	1935971000006105
1937761000006112	Extended consultation for new long term condition	1937761000006108
1947151000006112	Joint consultation with practice nurse and community diabetes specialist nurse	956781000000107
1947161000006114	Joint consultation with General Practitioner and community diabetes specialist nurse	956921000000109
1958191000006115	Extended consultation for complex condition	1958191000006104
2178721000000112	Consultation for complex sexual health need	839551000000107
2322391000000118	Joint consultation	903001000000102
2509371000006113	Consultation for radiotherapy	680007
2594421000006115	Consultation for hearing and/or speech problem	5947002
6509771000006113	Weight reduction consultation	388975008
6509681000006117	Weight maintenance consultation	388970003
6957931000006119	Pain consultation	420650002
6980881000006118	Consultation for acute pain	421946003
7353351000006112	Telemedicine consultation with patient	448337001
7507551000006118	Consultation for minor operation	698313007
7507561000006116	Consultation for treatment	698314001
7786391000006118	Consultation via video conference	719410009
8012271000006118	Telephone consultation	24681000000104
8105141000006115	Consultation	325851000000107
8105151000006118	Face to face consultation	325861000000105
8105161000006116	Remote consultation	325871000000103
8105171000006111	Remote verbal consultation	325881000000101
8105181000006114	Consultation by telephone	325891000000104
8105191000006112	Remote non-verbal consultation	325901000000103
8238881000006115	Consultation with patient	766841000000106
8361081000006119	Remote verbal consultation	978801000000107
8361131000006119	Consultation via multimedia	978871000000104
12990411000006114	Telephone consultation for suspected 2019-nCoV (novel coronavirus)	1240451000000106
12990421000006118	Telephone consultation for suspected Wuhan 2019-nCoV (novel coronavirus)	1240451000000106
13012191000006114	Telephone consultation for suspected SARS-CoV-2 (severe acute respiratory syndrome coronavirus 2)	1240451000000106
14393881000006110	Consultation via video consultation	14393881000006106

1920601000006115	Care home visit for annual patient review	1920601000006104
1920611000006117	Care home visit for mid-year patient review	1920611000006101
1957781000006118	Home visit post fall assessment	1957781000006102
2015311000006119	Telehealth intervention - follow-up home visit	2015311000006103
2290511000000115	Care home visit	889311000000109
2290551000000116	Care home visit for initial patient assessment	889331000000101
2290591000000112	Care home visit for follow-up patient review	889351000000108
3315521000006111	HV - Home visit	50357006
4726521000006114	General medicine domiciliary visit done	183738009
4735601000006111	Home visit for urgent condition	185466002
4735621000006118	Home visit for acute condition	185467006
4735641000006113	Home visit for chronic condition	185468001
4735891000006117	In-house physio - domiciliary visit	185493004
7029911000006114	Prenatal visit	424619006
13959081000006116	Annual visit	866149003
301141000000112	Seen in GP unit	199281000000101
8059021000006112	Seen in general practitioner unit	199281000000101
8059041000006117	Seen in GP (general practitioner) unit	199281000000101
285155016	Seen in dietician clinic	185176006
285156015	Seen in lipid clinic	185177002
285157012	Seen in cholesterol clinic	185178007
285223014	Seen in diabetic clinic	185229005
285238010	Seen in general medical clinic	185241003
285239019	Seen in asthma clinic	185242005
285264016	Seen in geriatric clinic	185263007
285265015	Seen in hypertension clinic	185264001
451322017	Seen in clinic	308021002
1681001000006111	Seen in general practitioner disease modifying antirheumatic drug monitoring clinic	364801000000104
1773381000006119	Seen in in-house nurse-led diabetic clinic	1773381000006103
1809141000006118	Seen in injection clinic	1809141000006102
1824591000006113	Seen in ear care clinic	1824591000006109
1839331000006117	Seen in chronic obstructive pulmonary disease clinic	1839331000006101
1928501000006119	Seen in rapid access clinic at GP surgery	1928501000006103
1969931000006119	Seen in influenza vaccination clinic	1969931000006103
2004011000006115	Seen in virtual clinic	2004011000006104
2174091000000117	Seen in injection clinic	837441000000102
5946691000006114	Seen in primary health care clinic	305906009
1958701000006114	Online communication	1958701000006105
1672471000006112	Clinic	257585005
1672871000006114	GP Surgery	1672871000006105
5475371000006112	GP surgery	264358009
285327012	Telephone encounter	185317003
1672621000006114	Walk-in clinic	81234003
3821241000006110	Walk-in centre	81234003
247812016	Practice nurse	159002008
285281013	Seen by practice nurse	185279008
408001000027113	Practice Nurse	1716971000006102
973851000006114	** Practice nurse	973851000006105
1796041000000119	Medication review by practice nurse	803361000000109
1842001000006118	Diabetic annual review by practice nurse	1842001000006102

Clinical codes for staff role to identify GP or practice nurse

CPRD GOLD: Consultations	
**role description**	**role**
Senior Partner	General practitioner (GP)
Partner	General practitioner (GP)
Assistant	General practitioner (GP)
Associate	General practitioner (GP)
Non-commercial local rota of less than 10 GPS	General practitioner (GP)
Salaried Partner	General practitioner (GP)
Locum	General practitioner (GP)
GP Registrar	General practitioner (GP)
Sole Practitioner	General practitioner (GP)
GP Retainer	General practitioner (GP)

Practice Nurse	Practice nurse

CPRD Aurum: Staff role		
**job category**	**job**	**interpretation**
4	General Medical Practitioner	General Practitioner
5	Salaried General Practitioner	General Practitioner
15	Associate Practitioner	General Practitioner
24	GP Registrar	General Practitioner
31	Sessional GP	General Practitioner
181	Locum GP	General Practitioner
27	Specialist Nurse Practitioner	Practice Nurse
33	Nurse Consultant	Practice Nurse
47	Staff Nurse	Practice Nurse
48	Enrolled Nurse	Practice Nurse
55	Associate Practitioner - Nurse	Practice Nurse
59	Student Practice Nurse	Practice Nurse
60	Nurse Manager	Practice Nurse
61	Sister/Charge Nurse	Practice Nurse
185	Advanced Practitioner	Practice Nurse

Clinical codes for palliative care referrals

EOLC codes in the referral file were also treated as palliative care referrals for CPRD GOLD.

CPRD GOLD: Palliative care referrals		
**medcode**	**readcode**	**Readterm**
9755	8H7g.00	Referral to palliative care service
13628	ZL5AP00	Referral to palliative care physician
22288	8HH7.00	Referred to community specialist palliative care team
26352	8H6A.00	Refer to terminal care consult
34531	8H7L.00	Refer for terminal care
22073	8HY..00	Referral to hospice

CPRD Aurum: Palliative care referrals		
**medcode**	**term**	**snomedconceptID**
283686014	Refer to terminal care consult	183569005
283721013	Refer for terminal care	183595007
449190016	Referral to palliative care service	306237005
449142011	Referral to hospice	306205009
904821000006111	Palliative medicine referral	904821000006107
1575191000006116	Palliative Medical Referral	1575191000006100
1776661000006111	Reason for referral: End of Life Support	1776661000006107
1969711000006110	Referral to palliative care outpatient service	1969711000006106
5953491000006118	Referral for palliative care	306237005
5954251000006113	Referral to palliative care physician	306288008
8453471000006112	Referral to community specialist palliative care team	1078411000000101
63101000000114	Referred to community specialist palliative care team	25411000000109
1775411000006115	Referred to End of Life Care Service	1775411000006104
2733521000000119	Referral to end of life care service	1091311000000108
7786451000006117	Referral to hospice at home service	719414000
5948441000006115	Referral by palliative care physician	305981001

Table S6: Clinical codes for covariates

Clinical codes for ethnicity

Closest ethnicity code to study entry was taken. GOLD: clinical, referral, tests; Aurum: consultations, observations

CPRD GOLD: Ethnicity			
**medcode**	**readcode**	**readterm**	**interpretation**
12446	9S10.00	White British	White
26467	9S13.00	White Scottish	White
26310	9S14.00	Other white British ethnic group	White
98111	9i00.00	White British - ethnic category 2001 census	White
12352	9i20.00	English - ethnic category 2001 census	White
12436	9i21.00	Scottish - ethnic category 2001 census	White
12681	9i22.00	Welsh - ethnic category 2001 census	White
28887	9i23.00	Cornish - ethnic category 2001 census	White
24837	9S11.00	White Irish	White
24270	9SA9.00	Irish (NMO)	White
47601	9SI..00	Irish traveller	White
12532	9i1..00	Irish - ethnic category 2001 census	White
98213	9i10.00	White Irish - ethnic category 2001 census	White
42294	9i24.00	Northern Irish - ethnic category 2001 census	White
55223	9i2C.00	Irish Traveller - ethnic category 2001 census	White
12444	9S12.00	Other white ethnic group	White
45947	9SAA.00	Greek/Greek Cypriot (NMO)	White
45955	9SAA.11	Greek (NMO)	White
47949	9SAA.12	Greek Cypriot (NMO)	White
32066	9SAB.00	Turkish/Turkish Cypriot (NMO)	White
32126	9SAB.11	Turkish (NMO)	White
32069	9SAB.12	Turkish Cypriot (NMO)	White
12633	9SAC.00	Other European (NMO)	White
45008	9T1..00	New Zealand ethnic groups	White
96789	9T1Y.00	Other New Zealand ethnic group	White
71425	9T1Z.00	New Zealand ethnic group NOS	White
12421	9i2..00	Other White background - ethnic category 2001 census	White
32778	9i26.00	Cypriot (part not stated) - ethnic category 2001 census	White
12355	9i27.00	Greek - ethnic category 2001 census	White
12769	9i28.00	Greek Cypriot - ethnic category 2001 census	White
12746	9i29.00	Turkish - ethnic category 2001 census	White
32413	9i2A.00	Turkish Cypriot - ethnic category 2001 census	White
12412	9i2B.00	Italian - ethnic category 2001 census	White
55113	9i2D.00	Traveller - ethnic category 2001 census	White
42290	9i2E.00	Gypsy/Romany - ethnic category 2001 census	White
12467	9i2F.00	Polish - ethnic category 2001 census	White
12433	9i2G.00	Baltic Estonian/Latvian/Lithuanian - ethn categ 2001 census	White
28973	9i2H.00	Commonwealth (Russian) Indep States - ethn categ 2001 census	White
26341	9i2J.00	Kosovan - ethnic category 2001 census	White
25422	9i2K.00	Albanian - ethnic category 2001 census	White
46956	9i2L.00	Bosnian - ethnic category 2001 census	White
28866	9i2M.00	Croatian - ethnic category 2001 census	White
47074	9i2N.00	Serbian - ethnic category 2001 census	White
28936	9i2P.00	Other republics former Yugoslavia - ethnic categ 2001 census	White
12591	9i2T.00	Other White or White unspecified ethnic category 2001 census	White
22467	9S1..00	White	White
35459	9SB3.00	Other ethnic, mixed white orig	White
40102	9i25.00	Ulster Scots - ethnic category 2001 census	White
26391	9i2Q.00	Mixed Irish and other White - ethnic category 2001 census	White
12402	9i2R.00	Oth White European/European unsp/Mixed European 2001 census	White
28900	9i2S.00	Other mixed White - ethnic category 2001 census	White
32425	9SB5.00	Black Caribbean and White	Mixed
32443	9SB6.00	Black African and White	Mixed
12742	9i3..00	White and Black Caribbean - ethnic category 2001 census	Mixed
12437	9i4..00	White and Black African - ethnic category 2001 census	Mixed
32401	9SB2.00	Other ethnic, Asian/White orig	Mixed

12638	9i5..00	White and Asian - ethnic category 2001 census	Mixed
47965	9S45.00	Black E Afric Asia/Indo-Caribb	Mixed
57753	9S45.11	Black East African Asian	Mixed
57763	9S45.12	Black Indo-Caribbean	Mixed
48005	9S46.00	Black Indian sub-continent	Mixed
35350	9S47.00	Black - other Asian	Mixed
32165	9S52.00	Other Black - Black/Asian orig	Mixed
38097	9SA6.00	E Afric Asian/Indo-Carib (NMO)	Mixed
12795	9i60.00	Black and Asian - ethnic category 2001 census	Mixed
49940	9i61.00	Black and Chinese - ethnic category 2001 census	Mixed
32399	9iA7.00	Caribbean Asian - ethnic category 2001 census	Mixed
12696	9SB..00	Other ethnic, mixed origin	Mixed
32420	9SB4.00	Other ethnic, other mixed orig	Mixed
12873	9i6..00	Other Mixed background - ethnic category 2001 census	Mixed
12706	9i63.00	Chinese and White - ethnic category 2001 census	Mixed
47005	9i64.00	Asian and Chinese - ethnic category 2001 census	Mixed
32408	9i65.00	Other Mixed or Mixed unspecified ethnic category 2001 census	Mixed
25623	9S51.00	Other Black - Black/White orig	Mixed
47401	9SB1.00	Other ethnic, Black/White orig	Mixed
40110	9i62.00	Black and White - ethnic category 2001 census	Mixed
12482	9S6..00	Indian	S.Asian
39696	9SA7.00	Indian sub-continent (NMO)	S.Asian
12414	9i7..00	Indian or British Indian - ethnic category 2001 census	S.Asian
26392	9iA1.00	Punjabi - ethnic category 2001 census	S.Asian
24690	9S7..00	Pakistani	S.Asian
12460	9i8..00	Pakistani or British Pakistani - ethnic category 2001 census	S.Asian
64133	9iA2.00	Kashmiri - ethnic category 2001 census	S.Asian
24740	9S8..00	Bangladeshi	S.Asian
28888	9i9..00	Bangladeshi or British Bangladeshi - ethn categ 2001 census	S.Asian
46818	9SA6.11	East African Asian (NMO)	S.Asian
26379	9SA8.00	Other Asian (NMO)	S.Asian
12668	9SH..00	Other Asian ethnic group	S.Asian
12513	9iA..00	Other Asian background - ethnic category 2001 census	S.Asian
47077	9iA3.00	East African Asian - ethnic category 2001 census	S.Asian
12653	9iA8.00	British Asian - ethnic category 2001 census	S.Asian
46056	9iA9.00	Mixed Asian - ethnic category 2001 census	S.Asian
28935	9iAA.00	Other Asian or Asian unspecified ethnic category 2001 census	S.Asian
12632	9S2..00	Black Caribbean	Black
57435	9S42.00	Black Caribbean/W.I./Guyana	Black
47950	9S42.11	Black Caribbean	Black
47997	9S42.12	Black West Indian	Black
32100	9S42.13	Black Guyana	Black
54593	9SA3.00	Caribbean I./W.I./Guyana (NMO)	Black
57094	9SA3.11	Caribbean Island (NMO)	Black
57075	9SA3.12	West Indian (NMO)	Black
93144	9SA3.13	Guyana (NMO)	Black
12432	9iB..00	Caribbean - ethnic category 2001 census	Black
12778	9S3..00	Black African	Black
35412	9S44.00	Black - other African country	Black
47969	9SA5.00	Other African countries (NMO)	Black
12350	9iC..00	African - ethnic category 2001 census	Black
12443	9iD0.00	Somali - ethnic category 2001 census	Black
32886	9iD1.00	Nigerian - ethnic category 2001 census	Black
24339	9S4..00	Black, other, non-mixed origin	Black
12452	9S41.00	Black British	Black
41329	9S43.00	Black N African/Arab/Iranian	Black
46812	9S43.11	Black North African	Black
57752	9S43.12	Black Arab	Black
50286	9S43.13	Black Iranian	Black
26312	9S48.00	Black Black - other	Black
25676	9S5..00	Black - other, mixed	Black
32136	9SG..00	Other black ethnic group	Black
32389	9iD..00	Other Black background - ethnic category 2001 census	Black
40097	9iD2.00	Black British - ethnic category 2001 census	Black
40096	9iD3.00	Mixed Black - ethnic category 2001 census	Black
46047	9iD4.00	Other Black or Black unspecified ethnic category 2001 census	Black

24272	9S9..00	Chinese	Other ethnic group
12468	9iE..00	Chinese - ethnic category 2001 census	Other ethnic group
30280	9SA..00	Other ethnic non-mixed (NMO)	Other ethnic group
32110	9SA1.00	Brit. ethnic minor. spec.(NMO)	Other ethnic group
57764	9SA2.00	Brit. ethnic minor. unsp (NMO)	Other ethnic group
24962	9SA4.00	N African Arab/Iranian (NMO)	Other ethnic group
47285	9SA4.11	North African Arab (NMO)	Other ethnic group
25082	9SA4.12	Iranian (NMO)	Other ethnic group
41214	9SAD.00	Other ethnic NEC (NMO)	Other ethnic group
25411	9SC..00	Vietnamese	Other ethnic group
12757	9SJ..00	Other ethnic group	Other ethnic group
46752	9T1A.00	Other Pacific ethnic group	Other ethnic group
12608	9iA4.00	Sri Lankan - ethnic category 2001 census	S.Asian
12760	9iA5.00	Tamil - ethnic category 2001 census	Other ethnic group
12887	9iA6.00	Sinhalese - ethnic category 2001 census	Other ethnic group
12434	9iF..00	Other - ethnic category 2001 census	Other ethnic group
12719	9iF0.00	Vietnamese - ethnic category 2001 census	Other ethnic group
12473	9iF1.00	Japanese - ethnic category 2001 census	Other ethnic group
12420	9iF2.00	Filipino - ethnic category 2001 census	Other ethnic group
12730	9iF3.00	Malaysian - ethnic category 2001 census	Other ethnic group
63872	9iF4.00	Buddhist - ethnic category 2001 census	Other ethnic group
56127	9iF5.00	Hindu - ethnic category 2001 census	Other ethnic group
46063	9iF6.00	Jewish - ethnic category 2001 census	Other ethnic group
47091	9iF7.00	Muslim - ethnic category 2001 census	Other ethnic group
49658	9iF8.00	Sikh - ethnic category 2001 census	Other ethnic group
46059	9iF9.00	Arab - ethnic category 2001 census	Other ethnic group
47028	9iFA.00	North African - ethnic category 2001 census	Other ethnic group
28909	9iFB.00	Mid East (excl Israeli, Iranian & Arab) - eth cat 2001 cens	Other ethnic group
46964	9iFC.00	Israeli - ethnic category 2001 census	Other ethnic group
25937	9iFD.00	Iranian - ethnic category 2001 census	Other ethnic group
45964	9iFE.00	Kurdish - ethnic category 2001 census	Other ethnic group
25451	9iFF.00	Moroccan - ethnic category 2001 census	Other ethnic group
26246	9iFG.00	Latin American - ethnic category 2001 census	Other ethnic group
12756	9iFH.00	South and Central American - ethnic category 2001 census	Other ethnic group
32382	9iFJ.00	Mauritian/Seychellois/Maldivian/St Helena eth cat 2001census	Other ethnic group
26455	9iFK.00	Any other group - ethnic category 2001 census	Other ethnic group
93749	916E.00	Patient ethnicity unknown	Ethnic group not specified
10196	9S...00	Ethnic groups (1991 census)	Ethnic group not specified
12429	9SD..00	Ethnic group not given - patient refused	Ethnic group not specified
24340	9SE..00	Ethnic group not recorded	Ethnic group not specified
45199	9SZ..00	Ethnic groups (census) NOS	Ethnic group not specified
23955	9T...00	Ethnicity and other related nationality data	Ethnic group not specified
12435	9i...00	Ethnic category - 2001 census	Ethnic group not specified
12351	9i0..00	British or mixed British - ethnic category 2001 census	Ethnic group not specified
12459	9iG..00	Ethnic category not stated - 2001 census	Ethnic group not specified
110417	9t0..00	Ethnic category - 2011 census England and Wales	Ethnic group not specified
110420	9t00.00	White:Eng/Welsh/Scot/NI/Brit - England and Wales 2011 census	White
110556	9t01.00	White: Irish - England and Wales ethnic category 2011 census	White
111386	9t02.00	White: Gypsy/Irish Traveller - Eng+Wales eth cat 2011 census	White
110407	9t03.00	White: other White backgrd- Eng+Wales ethnic cat 2011 census	White
110445	9t04.00	Mixed: White+Black Caribbean - Eng+Wales eth cat 2011 census	Mixed
110421	9t05.00	Mixed: White+Black African - Eng+Wales eth cat 2011 census	Mixed
110652	9t06.00	Mixed: White+Asian - Eng+Wales ethnic category 2011 census	Mixed
110477	9t08.00	Asian/Asian Brit: Indian - Eng+Wales ethnic cat 2011 census	S.Asian
110464	9t09.00	Asian/Asian British:Pakistani- Eng+Wales eth cat 2011 census	S.Asian
110590	9t0A.00	Asian/Asian Brit: Bangladeshi- Eng+Wales eth cat 2011 census	S.Asian
110922	9t0B.00	Asian/Asian Brit: Chinese - Eng+Wales ethnic cat 2011 census	Other ethnic group
111743	9t0C.00	Asian/Asian Brit: other Asian- Eng+Wales eth cat 2011 census	S.Asian
110437	9t0D.00	Black/African/Carib/Black Brit: African- Eng+Wales 2011 cens	Black
110436	9t0E.00	Black/African/Caribbn/Black Brit: Caribbean - Eng+Wales 2011	Black
110540	9t0F.00	Black/Afr/Carib/Black Brit: other Black- Eng+Wales 2011 cens	Black
110555	9t0G.00	Other ethnic group: Arab - Eng+Wales ethnic cat 2011 census	Other ethnic group
110742	9t0H.00	Other ethnic: any other grp - Eng+Wales eth cat 2011 census	Other ethnic group
110661	9t12.00	Mixed: White and Black Caribbean - NI ethnic cat 2011 census	Mixed

110651	9t13.00	Mixed: White and Black African - NI ethnic cat 2011 census	Mixed
110471	9t14.00	Mixed: White and Asian - NI ethnic category 2011 census	Mixed
110536	9t15.00	Mixed: other Mixed/multiple ethnic backgrd - NI 2011 census	Mixed
110422	9t16.00	Asian or Asian British: Indian - NI ethnic cat 2011 census	S.Asian
110538	9t17.00	Asian/Asian British: Pakistani - NI ethnic cat 2011 census	S.Asian
110720	9t18.00	Asian/Asian British: Bangladeshi - NI ethnic cat 2011 census	S.Asian
112363	9t19.00	Asian/Asian British: Chinese - NI ethnic cat 2011 census	Other ethnic group
110780	9t1E.00	Other ethnic group: Arab - NI ethnic category 2011 census	Other ethnic group
110646	9t1F.00	Other ethnic group: any other grp- NI ethnic cat 2011 census	Other ethnic group
110962	9t2..00	Ethnic category - 2011 census Scotland	Ethnic group not specified
110432	9t20.00	White: Scottish - Scotland ethnic category 2011 census	White
110694	9t21.00	White: other British - Scotland ethnic category 2011 census	White
110687	9t22.00	White: Irish - Scotland ethnic category 2011 census	White
110465	9t24.00	White: Polish - Scotland ethnic category 2011 census	White
110695	9t25.00	White: other White ethnic grp- Scotland ethnic cat 2011 cens	White
110696	9t26.00	Mixed/multiple ethnic grps: any- Scot ethnic cat 2011 census	Mixed
110460	9t27.00	Asian: Pakistani/Pakistani Scot/Pakistani Brit- Scot 2011	S.Asian
111368	9t28.00	Asian: Indian, Indian Scot/Indian Brit- Scotland 2011 census	S.Asian
112225	9t29.00	Bangladeshi, Bangladeshi Scot or Bangladeshi Brit- Scot 2011	S.Asian
111064	9t2A.00	Asian: Chinese - Scotland ethnic category 2011 census	Other ethnic group
110855	9t2B.00	Asian: other Asian group - Scotland ethnic cat 2011 census	S.Asian
111059	9t2C.00	African: African/African Scot/African Brit - Scotland 2011	Black
110655	9t2D.00	African: any other African - Scotland ethnic cat 2011 census	Black
112216	9t2G.00	Carib/Black: any other Black/Caribbean grp - Scotland 2011	Black
112245	9t2H.00	Other ethnic grp: Arab/Arab Scot/Arab British- Scotland 2011	Other ethnic group
111806	9t2J.00	Other ethnic grp: any other ethnic grp- Scotland 2011 census	Other ethnic group

CPRD Aurum: Ethnicity		
**medcode**	**term**	**interpretation**
250224013	Asian origin	S.Asian
250228011	Indian origin	S.Asian
250231012	West Indian origin	Black
196641000006110	RACE: Caucasian	White
196631000006117	RACE: Bangladeshi	S.Asian
196681000006116	RACE: Mixed	Mixed
250243013	Race: West indian	Black
196611000006111	RACE: Afro-caucasian	Mixed
196721000006111	RACE: Pakistani	S.Asian
2537217015	Race: White	White
1751621000006114	NHS Sickle Cell and Thalassaemia Screening Programme family origin African or African-Caribbean (black)	Black
1751651000006117	NHS Sickle Cell and Thalassaemia Screening Programme family origin South Asia (Asian)	S.Asian
1751661000006115	NHS Sickle Cell and Thalassaemia Screening Programme India or African-Indian family origin	S.Asian
1751671000006110	NHS Sickle Cell and Thalassaemia Screening Programme Pakistan family origin	S.Asian
1751681000006113	NHS Sickle Cell and Thalassaemia Screening Programme Bangladesh family origin	S.Asian
1751691000006111	NHS Sickle Cell and Thalassaemia Screening Programme family origin South East Asia (Asian)	S.Asian
1751721000006118	NHS Sickle Cell and Thalassaemia Screening Programme Malaysia, Vietnam, Philippines, Cambodia, Laos family origin	Other ethnic group
1751731000006115	NHS Sickle Cell and Thalassaemia Screening Programme family origin other non-European excluding African or African-Caribbean, South or South East Asian (other)	Other ethnic group
1751771000006117	NHS Sickle Cell and Thalassaemia Screening Programme family origin Southern and other European (white)	White
1751811000006117	NHS Sickle Cell and Thalassaemia Screening Programme family origin United Kingdom (white)	White
1751821000006113	NHS Sickle Cell and Thalassaemia Screening Programme family origin Northern European (white)	White
412016016	O/E - Asian origin	S.Asian
158341000000117	British or mixed British - ethnic category 2001 census	White
1063981000000117	White British - ethnic category 2001 census	White
141301000000110	Irish - ethnic category 2001 census	White
1064041000000111	White Irish - ethnic category 2001 census	White
141311000000112	Other White background - ethnic category 2001 census	White
157281000000117	English - ethnic category 2001 census	White

141431000000111	Scottish - ethnic category 2001 census	White
141441000000119	Welsh - ethnic category 2001 census	White
141461000000118	Cornish - ethnic category 2001 census	White
141451000000116	Northern Irish - ethnic category 2001 census	White
142691000000116	Ulster Scots - ethnic category 2001 census	White
141661000000115	Cypriot (part not stated) - ethnic category 2001 census	White
142701000000116	Greek - ethnic category 2001 census	White
142711000000119	Greek Cypriot - ethnic category 2001 census	White
156921000000110	Turkish - ethnic category 2001 census	White
142721000000113	Turkish Cypriot - ethnic category 2001 census	White
158481000000115	Italian - ethnic category 2001 census	White
138171000000114	Irish Traveller - ethnic category 2001 census	White
138181000000111	Traveller - ethnic category 2001 census	White
138191000000113	Gypsy/Romany - ethnic category 2001 census	White
138201000000110	Polish - ethnic category 2001 census	White
142741000000118	Kosovan - ethnic category 2001 census	White
138231000000116	Albanian - ethnic category 2001 census	White
142751000000115	Bosnian - ethnic category 2001 census	White
142761000000117	Croatian - ethnic category 2001 census	White
157991000000110	Serbian - ethnic category 2001 census	White
937371000006116	Other republics former Yugoslavia - ethnic categ 2001 census	White
142781000000114	Mixed Irish and other White - ethnic category 2001 census	White
937391000006115	Oth White European/European unsp/Mixed European 2001 census	White
142791000000111	Other mixed White - ethnic category 2001 census	White
937411000006115	Other White or White unspecified ethnic category 2001 census	White
141321000000118	White and Black Caribbean - ethnic category 2001 census	Mixed
141331000000116	White and Black African - ethnic category 2001 census	Mixed
141341000000113	White and Asian - ethnic category 2001 census	Mixed
141351000000111	Other Mixed background - ethnic category 2001 census	Mixed
141471000000113	Black and Asian - ethnic category 2001 census	Mixed
141481000000110	Black and Chinese - ethnic category 2001 census	Mixed
157291000000115	Black and White - ethnic category 2001 census	Mixed
141491000000112	Chinese and White - ethnic category 2001 census	Mixed
158361000000116	Asian and Chinese - ethnic category 2001 census	Mixed
937511000006119	Other Mixed or Mixed unspecified ethnic category 2001 census	Mixed
157271000000119	Indian or British Indian - ethnic category 2001 census	S.Asian
141361000000114	Pakistani or British Pakistani - ethnic category 2001 census	S.Asian
937541000006115	Bangladeshi or British Bangladeshi - ethn categ 2001 census	S.Asian
141381000000117	Other Asian background - ethnic category 2001 census	S.Asian
141521000000110	Punjabi - ethnic category 2001 census	S.Asian
141531000000112	Kashmiri - ethnic category 2001 census	S.Asian
141541000000115	East African Asian - ethnic category 2001 census	S.Asian
136081000000111	Sri Lankan - ethnic category 2001 census	S.Asian
141551000000117	Tamil - ethnic category 2001 census	Other ethnic group
157301000000116	Sinhalese - ethnic category 2001 census	Other ethnic group
141571000000114	Caribbean Asian - ethnic category 2001 census	Mixed
141561000000119	British Asian - ethnic category 2001 census	S.Asian
141511000000116	Mixed Asian - ethnic category 2001 census	Mixed
937651000006117	Other Asian or Asian unspecified ethnic category 2001 census	S.Asian
154401000000118	Caribbean - ethnic category 2001 census	Black
141391000000115	African - ethnic category 2001 census	Black
158351000000119	Other Black background - ethnic category 2001 census	Black
141591000000113	Somali - ethnic category 2001 census	Black
141601000000119	Nigerian - ethnic category 2001 census	Black
157311000000119	Black British - ethnic category 2001 census	Black
158371000000111	Mixed Black - ethnic category 2001 census	Black
937731000006115	Other Black or Black unspecified ethnic category 2001 census	Black
141401000000117	Chinese - ethnic category 2001 census	Other ethnic group
141411000000115	Other - ethnic category 2001 census	Other ethnic group
141621000000111	Vietnamese - ethnic category 2001 census	Other ethnic group
141631000000113	Japanese - ethnic category 2001 census	Other ethnic group
141641000000116	Filipino - ethnic category 2001 census	Other ethnic group
141651000000118	Malaysian - ethnic category 2001 census	Other ethnic group
142881000000117	Buddhist - ethnic category 2001 census	Other ethnic group
157351000000115	Hindu - ethnic category 2001 census	Other ethnic group
138241000000113	Jewish - ethnic category 2001 census	Other ethnic group

138281000000117	Muslim - ethnic category 2001 census	Other ethnic group
142891000000115	Sikh - ethnic category 2001 census	Other ethnic group
138251000000111	Arab - ethnic category 2001 census	Other ethnic group
142811000000112	North African - ethnic category 2001 census	Other ethnic group
937871000006114	Mid East (excl Israeli, Iranian & Arab) - eth cat 2001 cens	Other ethnic group
142831000000116	Israeli - ethnic category 2001 census	Other ethnic group
138261000000114	Iranian - ethnic category 2001 census	Other ethnic group
142841000000113	Kurdish - ethnic category 2001 census	Other ethnic group
142851000000111	Moroccan - ethnic category 2001 census	Other ethnic group
142861000000114	Latin American - ethnic category 2001 census	Other ethnic group
138271000000119	South and Central American - ethnic category 2001 census	Other ethnic group
142901000000119	Any other group - ethnic category 2001 census	Not specified
141421000000114	Ethnic category not stated - 2001 census	Not specified
285925010	White	White
459726019	White British	White
459727011	White Irish	White
459728018	Other white ethnic group	White
1780407014	White Scottish	White
1780408016	Other white British ethnic group	White
514611000006111	Black Caribbean	Black
30683015	Black African	Black
285931013	Black, other, non-mixed origin	Black
285932018	Black British	Black
405064011	Black Caribbean/W.I./Guyana	Black
285930014	Black Caribbean	Black
453109012	Black West Indian	Black
453110019	Black Guyana	Black
405065012	Black N African/Arab/Iranian	Black
411573013	Black North African	Black
411574019	Black Arab	Black
411575018	Black Iranian	Black
285943014	Black - other African country	Black
514651000006112	Black E Afric Asia/Indo-Caribb	Black
411576017	Black East African Asian	Black
411577014	Black Indo-Caribbean	Black
285948017	Black Indian sub-continent	Black
285949013	Black - other Asian	Black
285950013	Black Black - other	Black
285951012	Black - other, mixed	Black
285952017	Other Black - Black/White orig	Mixed
285953010	Other Black - Black/Asian orig	Mixed
285954016	Indian	S.Asian
285955015	Pakistani	S.Asian
285956019	Bangladeshi	S.Asian
56590016	Chinese	Other ethnic group
285958018	Other ethnic non-mixed (NMO)	Other ethnic group
285959014	Brit. ethnic minor. spec.(NMO)	Other ethnic group
285960016	Brit. ethnic minor. unsp (NMO)	Other ethnic group
405067016	Caribbean I./W.I./Guyana (NMO)	Black
411578016	Caribbean Island (NMO)	Black
411579012	West Indian (NMO)	Black
411580010	Guyana (NMO)	Black
405068014	N African Arab/Iranian (NMO)	Other ethnic group
411581014	North African Arab (NMO)	Other ethnic group
411582019	Iranian (NMO)	Other ethnic group
285971015	Other African countries (NMO)	Black
405069018	E Afric Asian/Indo-Carib (NMO)	Mixed
411583012	East African Asian (NMO)	S.Asian
411584018	Indo-Caribbean (NMO)	Mixed
285976013	Indian sub-continent (NMO)	S.Asian
285977016	Other Asian (NMO)	S.Asian
285978014	Irish (NMO)	White
405070017	Greek/Greek Cypriot (NMO)	White
411594011	Greek (NMO)	White
411595012	Greek Cypriot (NMO)	White
405071018	Turkish/Turkish Cypriot (NMO)	White

411596013	Turkish (NMO)	White
411597016	Turkish Cypriot (NMO)	White
285987017	Other European (NMO)	White
285988010	Other ethnic NEC (NMO)	Other ethnic group
285989019	Other ethnic, mixed origin	Mixed
285990011	Other ethnic, Black/White orig	Mixed
285991010	Other ethnic, Asian/White orig	Mixed
285992015	Other ethnic, mixed white orig	Mixed
285993013	Other ethnic, other mixed orig	Mixed
460153018	Black Caribbean and White	Mixed
460154012	Black African and White	Mixed
456650013	Vietnamese	Other ethnic group
456651012	Ethnic group not given - patient refused	Not specified
456652017	Ethnic group not recorded	Not specified
459782019	Other black ethnic group	Black
459784018	Other Asian ethnic group	S.Asian
459786016	Irish traveller	White
459785017	Other ethnic group	Other ethnic group
286003011	Ethnic groups (census) NOS	Not specified
1968051000006116	White:Eng/Welsh/Scot/NI/Brit - England and Wales 2011 census	White
2484671000000118	White: Irish - England and Wales ethnic category 2011 census	White
1968071000006114	White: Gypsy/Irish Traveller - Eng+Wales eth cat 2011 census	White
1968081000006112	White: other White backgrd- Eng+Wales ethnic cat 2011 census	White
1968091000006110	Mixed: White+Black Caribbean - Eng+Wales eth cat 2011 census	Mixed
1968101000006116	Mixed: White+Black African - Eng+Wales eth cat 2011 census	Mixed
1968111000006118	Mixed: White+Asian - Eng+Wales ethnic category 2011 census	Mixed
1968121000006114	Mixed: other Mixed/multiple backgrd - Eng+Wales 2011 census	Mixed
1968131000006112	Asian/Asian Brit: Indian - Eng+Wales ethnic cat 2011 census	S.Asian
1968141000006119	Asian/Asian British:Pakistani- Eng+Wales eth cat 2011 census	S.Asian
1968151000006117	Asian/Asian Brit: Bangladeshi- Eng+Wales eth cat 2011 census	S.Asian
1968161000006115	Asian/Asian Brit: Chinese - Eng+Wales ethnic cat 2011 census	S.Asian
1968171000006110	Asian/Asian Brit: other Asian- Eng+Wales eth cat 2011 census	S.Asian
1968181000006113	Black/African/Carib/Black Brit: African- Eng+Wales 2011 cens	Black
1968191000006111	Black/African/Caribbn/Black Brit: Caribbean - Eng+Wales 2011	Black
1968201000006114	Black/Afr/Carib/Black Brit: other Black- Eng+Wales 2011 cens	Black
1968211000006112	Other ethnic group: Arab - Eng+Wales ethnic cat 2011 census	Other ethnic group
1968221000006116	Other ethnic: any other grp - Eng+Wales eth cat 2011 census	Other ethnic group
286006015	New Zealand ethnic groups	White
2486161000000112	White - Northern Ireland ethnic category 2011 census	White
1968251000006113	Irish Traveller - Northern Ireland ethnic cat 2011 census	White
1968261000006110	Mixed: White and Black Caribbean - NI ethnic cat 2011 census	Mixed
1968271000006115	Mixed: White and Black African - NI ethnic cat 2011 census	Mixed
1968281000006117	Mixed: White and Asian - NI ethnic category 2011 census	Mixed
1968291000006119	Mixed: other Mixed/multiple ethnic backgrd - NI 2011 census	Mixed
1968301000006118	Asian or Asian British: Indian - NI ethnic cat 2011 census	S.Asian
1968311000006115	Asian/Asian British: Pakistani - NI ethnic cat 2011 census	S.Asian
1968321000006111	Asian/Asian British: Bangladeshi - NI ethnic cat 2011 census	S.Asian
1968331000006114	Asian/Asian British: Chinese - NI ethnic cat 2011 census	Other ethnic group
286017019	Other Pacific ethnic group	Other ethnic group
1968341000006116	Asian/Asian British: other Asian - NI ethnic cat 2011 census	Other ethnic group
286018012	South East Asian	S.Asian
1968351000006119	Black/Afri/Carib/Black Brit: African- NI eth cat 2011 census	Black
1968361000006117	Black/Afri/Carib/Black Brit: Caribbean- NI eth cat 2011 cens	Black
781081000006113	Indian	S.Asian
1968371000006112	Black/Afri/Carib/Black Brit: other - NI eth cat 2011 census	Black
1968381000006110	Other ethnic group: Arab - NI ethnic category 2011 census	Other ethnic group
286020010	Other Asian	Other ethnic group
1968391000006113	Other ethnic group: any other grp- NI ethnic cat 2011 census	Other ethnic group
286021014	Other New Zealand ethnic group	Other ethnic group
286022019	New Zealand ethnic group NOS	White
2487281000000112	White: Scottish - Scotland ethnic category 2011 census	White
2487321000000116	White: other British - Scotland ethnic category 2011 census	White
2487361000000112	White: Irish - Scotland ethnic category 2011 census	White
1968441000006112	White: Gypsy/Irish Traveller - Scotland ethnic cat 2011 cens	White
2487481000000113	White: Polish - Scotland ethnic category 2011 census	White
1968461000006111	White: other White ethnic grp- Scotland ethnic cat 2011 cens	White

1968471000006116	Mixed/multiple ethnic grps: any- Scot ethnic cat 2011 census	Mixed
1968481000006118	Asian: Pakistani/Pakistani Scot/Pakistani Brit- Scot 2011	S.Asian
1968491000006115	Asian: Indian, Indian Scot/Indian Brit- Scotland 2011 census	S.Asian
1968501000006111	Bangladeshi, Bangladeshi Scot or Bangladeshi Brit- Scot 2011	S.Asian
1968511000006114	Asian: Chinese - Scotland ethnic category 2011 census	Other ethnic group
1968521000006118	Asian: other Asian group - Scotland ethnic cat 2011 census	Other ethnic group
1968541000006113	African: any other African - Scotland ethnic cat 2011 census	Black
1968551000006110	Carib/Black: Caribbean/Carib Scot/Carib Brit- Scotland 2011	Black
1968561000006112	Carib/Black: Black/Black Scot/Black Brit- Scotland 2011 cens	Black
1968571000006117	Carib/Black: any other Black/Caribbean grp - Scotland 2011	Black
1968581000006119	Other ethnic grp: Arab/Arab Scot/Arab British- Scotland 2011	Other ethnic group
1968591000006116	Other ethnic grp: any other ethnic grp- Scotland 2011 census	Other ethnic group
1564961000006118	Irish	White
2645811000000115	Roma ethnic group	Other ethnic group
459730016	Black - ethnic group	Black
12009371000006110	Patient declined to provide information about ethnic group	Not specified
459729014	Mixed ethnic census group	Mixed
4740381000006118	Other ethnic, mixed white origin	White
4740401000006118	Other ethnic, other mixed origin	Mixed
4740341000006112	Other ethnic, Black/White origin	Mixed
4740361000006111	Other ethnic, Asian/White origin	Mixed
1564921000006112	Indian	S.Asian
1565441000006111	Pakistani	S.Asian
5580031000006118	On examination - Asian origin	S.Asian
6846371000006111	Caucasian	White
1565951000006119	Vietnamese	Other ethnic group
1564291000006119	Bangladeshi	S.Asian
1564941000006117	Iranian	Other ethnic group
1564951000006115	Iraqi	Other ethnic group

Hospital episode statistics: Ethnicity	
**icd**	**description**
Bangladeshi	S.Asian
Bl_Afric	Black
Bl_Carib	Black
Bl_Other	Black
Chinese	Other ethnic group
Indian	S.Asian
Mixed	Mixed
Oth_Asian	S.Asian
Other	Other ethnic group
Pakistani	S.Asian
Unknown	Not known
White	White

Clinical codes for smoking

Closest measure prior to cohort entry date was taken. GOLD: clinical, referral, tests; Aurum: consultations, observations. For Aurum, any values>0 linked with the additional codes (number of units) were coded as ‘Yes’. Values>150 were ignored.

CPRD GOLD: Smoking			
**medcode**	**readcode**	**readterm**	**interpretation**
33	1371.00	Never smoked tobacco	never smoker
60	137L.00	Current non-smoker	never smoker
90	137S.00	Ex smoker	ex smoker
93	137P.00	Cigarette smoker	current smoker
776	137K.00	Stopped smoking	ex smoker
1822	1376.00	Very heavy smoker - 40+cigs/d	current smoker
1823	137P.11	Smoker	current smoker
1878	1374.00	Moderate smoker - 10-19 cigs/d	current smoker
2111	6791.00	Health ed. - smoking	current smoker
3568	1375.00	Heavy smoker - 20-39 cigs/day	current smoker
7622	8CAL.00	Smoking cessation advice	current smoker

10558	137R.00	Current smoker	current smoker
10742	8HTK.00	Referral to stop-smoking clinic	current smoker
11356	9N2k.00	Seen by smoking cessation advisor	current smoker
11788	1371.11	Non-smoker	never smoker
12240	137G.00	Trying to give up smoking	current smoker
12878	137T.00	Date ceased smoking	ex smoker
12941	1372.11	Occasional smoker	current smoker
12943	137J.00	Cigar smoker	current smoker
12944	1373.00	Light smoker - 1-9 cigs/day	current smoker
12945	137M.00	Rolls own cigarettes	current smoker
12946	137F.00	Ex-smoker - amount unknown	ex smoker
12947	137H.00	Pipe smoker	current smoker
12951	137Q.11	Smoking restarted	current smoker
12952	137Q.00	Smoking started	current smoker
12954	ZV4K000	[V]Tobacco use	current smoker
12955	1379.00	Ex-moderate smoker (10-19/day)	ex smoker
12956	137A.00	Ex-heavy smoker (20-39/day)	ex smoker
12957	1378.00	Ex-light smoker (1-9/day)	ex smoker
12958	1372.00	Trivial smoker - < 1 cig/day	current smoker
12959	137B.00	Ex-very heavy smoker (40+/day)	ex smoker
12960	137Z.00	Tobacco consumption NOS	current smoker
12961	1377.00	Ex-trivial smoker (<1/day)	ex smoker
12962	137E.00	Tobacco consumption unknown	current smoker
12963	137Y.00	Cigar consumption	current smoker
12964	137C.00	Keeps trying to stop smoking	current smoker
12965	137X.00	Cigarette consumption	current smoker
12966	137V.00	Smoking reduced	current smoker
12967	137a.00	Pipe tobacco consumption	current smoker
13351	137I.00	Passive smoker	never smoker
16717	H310100	Smokers' cough	current smoker
18573	8H7i.00	Referral to smoking cessation advisor	current smoker
18926	67H1.00	Lifestyle advice regarding smoking	current smoker
19488	137O.00	Ex cigar smoker	ex smoker
26470	137N.00	Ex pipe smoker	ex smoker
30423	137c.00	Thinking about stopping smoking	current smoker
30762	137d.00	Not interested in stopping smoking	current smoker
31114	137b.00	Ready to stop smoking	current smoker
32687	E251.00	Tobacco dependence	current smoker
32973	137W.00	Chews tobacco	current smoker
34126	13p0.00	Negotiated date for cessation of smoking	current smoker
35055	ZV6D800	[V]Tobacco abuse counselling	current smoker
38112	13p5.00	Smoking cessation programme start date	current smoker
41042	8CAg.00	Smoking cessation advice provided by community pharmacist	current smoker
41979	137e.00	Smoking restarted	current smoker
46300	137g.00	Cigarette pack-years	current smoker
46321	137f.00	Reason for restarting smoking	current smoker
46654	137D.00	Admitted tobacco cons untrue ?	current smoker
62686	137h.00	Minutes from waking to first tobacco consumption	current smoker
68658	E251z00	Tobacco dependence NOS	current smoker
70746	E251100	Tobacco dependence, continuous	current smoker
72706	E251300	Tobacco dependence in remission	current smoker
95610	E251000	Tobacco dependence, unspecified	current smoker
97210	137j.00	Ex-cigarette smoker	ex smoker
98137	67H6.00	Brief intervention for smoking cessation	current smoker
98154	8HkQ.00	Referral to NHS stop smoking service	current smoker
98177	9kn..00	Non-smoker annual review - enhanced services administration	never smoker
98347	9ko..00	Current smoker annual review - enhanced services admin	current smoker
98447	9km..00	Ex-smoker annual review - enhanced services administration	ex smoker
99838	137K000	Recently stopped smoking	ex smoker
100099	8IAj.00	Smoking cessation advice declined	current smoker
100495	137l.00	Ex roll-up cigarette smoker	ex smoker

100963	9km..11	Ex-smoker annual review	ex smoker
101338	137m.00	Failed attempt to stop smoking	current smoker
101764	13p5000	Practice based smoking cessation programme start date	current smoker
101878	9kn..11	Non-smoker annual review	never smoker
102361	9NS0200	Referral for smoking cessation service offered	current smoker
103507	8CdB.00	Stop smoking service opportunity signposted	current smoker
104310	9ko..11	Current smoker annual review	current smoker
105501	137o.00	Waterpipe tobacco consumption	current smoker
106359	8T08.00	Referral to smoking cessation service	current smoker
106391	8IEo.00	Referral to smoking cessation service declined	current smoker
106891	137i.00	Ex-tobacco chewer	ex smoker
108835	E251200	Tobacco dependence, episodic	current smoker
110692	8B31G00	Varenicline smoking cessation therapy offered	current smoker

CPRD Aurum: Smoking

medcode	term	interpretation
14866014	Non-smoker	never smoker
72373013	Passive smoker	never smoker
78013015	Smokers' cough	current smoker
99639019	Cigar smoker	current smoker
108938018	Cigarette smoker	current smoker
128130017	Smoker	current smoker
136515019	Pipe smoker	current smoker
216212011	Smoking reduced	current smoker
250363016	Ex-trivial cigarette smoker (<1/day)	ex smoker
250364010	Ex-light cigarette smoker (1-9/day)	ex smoker
250365011	Ex-moderate cigarette smoker (10-19/day)	ex smoker
250366012	Ex-heavy cigarette smoker (20-39/day)	ex smoker
250367015	Ex-very heavy cigarette smoker (40+/day)	ex smoker
250368013	Keeps trying to stop smoking	current smoker
250369017	Admitted tobacco consumption possibly untrue	current smoker
250370016	Tobacco consumption unknown	current smoker
250371017	Ex-smoker - amount unknown	ex smoker
250372012	Trying to give up smoking	current smoker
250373019	Stopped smoking	ex smoker
250374013	Current non-smoker	never smoker
250375014	Rolls own cigarettes	current smoker
250385010	Date ceased smoking	ex smoker
250387019	Tobacco consumption NOS	current smoker
295256013	Tobacco dependence, unspecified	current smoker
295257016	Tobacco dependence, continuous	current smoker
295258014	Tobacco dependence, episodic	current smoker
295259018	Tobacco dependence in remission	current smoker
295260011	Tobacco dependence NOS	current smoker
338608011	Smoking cessation advice	current smoker
342602019	Ex-tobacco chewer	ex smoker
344793011	Cigarette consumption	current smoker
344794017	Cigar consumption	current smoker
344795016	Pipe tobacco consumption	current smoker
397732011	Never smoked tobacco	never smoker
397733018	Occasional smoker	current smoker
418914010	Ex-cigarette smoker	ex smoker
459722017	Referral to stop-smoking clinic	current smoker
460828018	Tobacco user	current smoker
461114016	Counselling about tobacco use	current smoker
503483019	Current smoker	current smoker
504769011	Chews tobacco	current smoker
1484932017	Smoking cessation milestones	current smoker
1484933010	Negotiated date for cessation of smoking	current smoker
1488576014	Thinking about stopping smoking	current smoker
1488577017	Ready to stop smoking	current smoker
1488578010	Not interested in stopping smoking	current smoker
1489355012	Referral to smoking cessation advisor	current smoker

1773560017	Smoking cessation programme start date	current smoker
1780273014	Seen by smoking cessation advisor	current smoker
1780360012	Reason for restarting smoking	current smoker
1780396011	Cigarette pack-years	current smoker
2474719011	Minutes from waking to first tobacco consumption	current smoker
2669652019	Smoking started	current smoker
2670126018	Smoking restarted	current smoker
2982426011	Provision of smoking cessation leaflet	current smoker
3513199018	Ex-smoker for less than 1 year	ex smoker
67621000006112	Very heavy cigarette smoker (40+ cigs/day)	current smoker
88471000006112	Trivial cigarette smoker (less than one cigarette/day)	current smoker
102951000006115	Tobacco dependence	current smoker
137791000006118	Smoking restarted	current smoker
303501000000115	Smoking cessation advice provided by community pharmacist	current smoker
604961000006114	Current Smoker NOS	current smoker
649821000006115	Ex-cigar smoker	ex smoker
649831000006117	Ex-pipe smoker	ex smoker
649841000006110	Ex-smoker	ex smoker
649851000006112	Ex- Rolled Tobacco Smoker	ex smoker
649861000006114	Ex-Cigarette Smoker	ex smoker
700121000006118	Moderate cigarette smoker (10-19 cigs/day)	current smoker
743331000006116	Light cigarette smoker (1-9 cigs/day)	current smoker
818061000006111	Smoking cessation education	current smoker
819331000006110	Heavy cigarette smoker (20-39 cigs/day)	current smoker
852111000006118	Gradual smoking reduction	current smoker
852981000006111	Rolls own cigarettes	current smoker
853001000006110	Ex-smoker NOS	ex smoker
854021000006115	Cigarette smoker	current smoker
854051000006112	Ex-pipe smoker	ex smoker
854071000006119	Current smoker	current smoker
854111000006110	Past smoker	ex smoker
854151000006111	Date stopped smoking	never smoker
854951000006113	Grade A non-smoker	never smoker
854961000006110	Grade B light smoker (1-10/day)	current smoker
854981000006117	Grade C moderate smoker (11-20/day)	current smoker
855001000006114	Grade D heavy smoker (>20 Day)	current smoker
903041000006110	EX-Smoker NOS	ex smoker
903051000006112	Tobacco Consumption Nil	never smoker
903981000006117	Not interested in stopping smoking	current smoker
904001000006111	Ready to stop smoking	current smoker
904021000006118	Previous smoking quit attempts	current smoker
904031000006115	Thinking about stopping smoking	current smoker
904111000006113	Carbon monoxide validation confirms non-smoker	never smoker
904121000006117	Carbon monoxide validation confirms smoker	current smoker
909391000006117	[RFC] Smoking cessation	never smoker
936011000006114	Advice on effects of smoking on health	current smoker
961581000006114	Smokes/uses tobacco products	current smoker
1009271000006118	Non Smoker - Nos	never smoker
1059701000000119	Ex roll-up cigarette smoker	ex smoker
1123751000000113	Non-smoker annual review - enhanced services administration	never smoker
1123951000000110	Ex-smoker annual review - enhanced services administration	ex smoker
1125741000000111	Brief intervention for smoking cessation	current smoker
1151791000000117	Recently stopped smoking	ex smoker
1152111000000118	Current smoker annual review	current smoker
1154431000000112	Non-smoker annual review	never smoker
1154471000000114	Ex-smoker annual review	ex smoker
1175011000000112	Smoking cessation programme declined	current smoker
1176421000000118	Smoking cessation advice declined	current smoker
1563081000000115	Practice based smoking cessation programme start date	current smoker
1591651000006110	Smoking cessation referral declined	current smoker
1592611000000110	Failed attempt to stop smoking	current smoker
1626121000006116	Smoking cessation drug therapy - varenicline	current smoker
1709641000000115	Referral for smoking cessation service offered	current smoker

1714541000006110	Current smoker annual review - enhanced services admin	current smoker
1715591000006117	Referral to National Health Service stop smoking service	current smoker
1750931000000118	Stop smoking service opportunity signposted	current smoker
1777011000006118	Reason for referral: Smoking Cessation	current smoker
1778181000006110	Group session: Stop smoking education programme	current smoker
1809121000006113	Waterpipe tobacco consumption	current smoker
1819411000006114	Smoking increased	current smoker
2170961000000116	Waterpipe tobacco consumption	current smoker
2251911000000114	Referral to smoking cessation service	current smoker
2462391000000119	Varenicline smoking cessation therapy declined	current smoker
2462431000000110	Varenicline smoking cessation therapy offered	current smoker
2717211000000112	Signposting to smoking cessation service	current smoker
2735181000000113	Ex-smoker amount unknown	ex smoker
2735201000000112	Ex-very heavy smoker (40+/day)	ex smoker
2735281000000119	Ex-heavy smoker (20-39/day)	ex smoker
2735331000000112	Ex-moderate smoker (10-19/day)	ex smoker
2735381000000111	Ex-light smoker (1-9/day)	ex smoker
2735421000000119	Ex-trivial smoker (<1/day)	ex smoker
2741221000000116	Referral for brief intervention for smoking cessation	current smoker
3374141000006117	Tolerant ex-smoker	ex smoker
3419101000006116	Moderate smoker (20 or less per day)	current smoker
3422221000006116	Heavy smoker (over 20 per day)	current smoker
3582451000006114	Cigarette smoking tobacco	current smoker
3874641000006110	Pipe smoking tobacco	current smoker
3959111000006111	Tobacco dependence syndrome	current smoker
4180331000006118	Tobacco use	current smoker
4939241000006115	Advice on effects of smoking on health	current smoker
4939251000006118	Advice on smoking	current smoker
4948551000006111	Drops unextinguished cigarettes	current smoker
4980561000006117	Time since stopped smoking	ex smoker
4980911000006113	Chews loose leaf tobacco	current smoker
5003141000006118	Trivial cigarette smoker	current smoker
5003151000006116	Light cigarette smoker	current smoker
5003161000006119	Moderate cigarette smoker	current smoker
5003171000006114	Heavy cigarette smoker	current smoker
5003181000006112	Very heavy cigarette smoker	current smoker
5003191000006110	Chain smoker	current smoker
5074201000006119	Smokers keratosis	current smoker
5495901000006112	Amount and type of tobacco smoked	current smoker
5495941000006114	Occasional smoker	current smoker
5495951000006111	Occasional cigarette smoker (less than one cigarette/day)	current smoker
6217151000006116	Intolerant ex-smoker	ex smoker
6217281000006116	Aggressive ex-smoker	ex smoker
6427931000006116	Smoking cessation assistance	current smoker
6718071000006115	Current non smoker but past smoking history unknown	never smoker
7368961000006112	Stopped smoking during pregnancy	ex smoker
7368971000006117	Stopped smoking before pregnancy	ex smoker
7375991000006118	Smokes tobacco daily	current smoker
7559591000006110	Tobacco use cessation education	current smoker
7569061000006118	Never used tobacco	never smoker
7650811000006116	Assessment of readiness for smoking cessation	current smoker
7696001000006118	Patient request for smoking cessation information	current smoker
8017571000006117	Ex-smoker for more than 1 year	ex smoker
8063181000006116	Wants to stop smoking	current smoker
8153371000006117	Occasional tobacco smoker	current smoker
8190531000006112	Referral to NHS (National Health Service) stop smoking service	current smoker
8196151000006112	Smoking cessation leaflet given	current smoker
11904991000006116	Occasional cigarette smoker	current smoker
14494021000006110	Former smoker	ex smoker
14933931000006118	Community Pharmacy Smoking Cessation Service	current smoker
854021000006115	Cigarette smoker	current smoker
854051000006112	Ex-pipe smoker	ex smoker
854071000006119	Current smoker	current smoker

854111000006110	Past smoker	ex smoker
854151000006111	Date stopped smoking	never smoker
854951000006113	Grade A non-smoker	never smoker
854961000006110	Grade B light smoker (1-10/day)	current smoker
854981000006117	Grade C moderate smoker (11-20/day)	current smoker
855001000006114	Grade D heavy smoker (>20 Day)	current smoker
903041000006110	EX-Smoker NOS	ex smoker
903051000006112	Tobacco Consumption Nil	never smoker
903981000006117	Not interested in stopping smoking	current smoker
904001000006111	Ready to stop smoking	current smoker
904021000006118	Previous smoking quit attempts	current smoker
904031000006115	Thinking about stopping smoking	current smoker
904111000006113	Carbon monoxide validation confirms non-smoker	never smoker
904121000006117	Carbon monoxide validation confirms smoker	current smoker
909391000006117	[RFC] Smoking cessation	never smoker
936011000006114	Advice on effects of smoking on health	current smoker
961581000006114	Smokes/uses tobacco products	current smoker
1009271000006118	Non Smoker - Nos	never smoker
1059701000000119	Ex roll-up cigarette smoker	ex smoker
1123751000000113	Non-smoker annual review - enhanced services administration	never smoker
1123951000000110	Ex-smoker annual review - enhanced services administration	ex smoker
1125741000000111	Brief intervention for smoking cessation	current smoker
1151791000000117	Recently stopped smoking	ex smoker
1152111000000118	Current smoker annual review	current smoker
1154431000000112	Non-smoker annual review	never smoker
1154471000000114	Ex-smoker annual review	ex smoker
1175011000000112	Smoking cessation programme declined	current smoker
1176421000000118	Smoking cessation advice declined	current smoker
1563081000000115	Practice based smoking cessation programme start date	current smoker
1591651000006110	Smoking cessation referral declined	current smoker
1592611000000110	Failed attempt to stop smoking	current smoker
1626121000006116	Smoking cessation drug therapy - varenicline	current smoker
1709641000000115	Referral for smoking cessation service offered	current smoker
1714541000006110	Current smoker annual review - enhanced services admin	current smoker
1715591000006117	Referral to National Health Service stop smoking service	current smoker
1750931000000118	Stop smoking service opportunity signposted	current smoker
1777011000006118	Reason for referral: Smoking Cessation	current smoker
1778181000006110	Group session: Stop smoking education programme	current smoker
1809121000006113	Waterpipe tobacco consumption	current smoker
1819411000006114	Smoking increased	current smoker
2170961000000116	Waterpipe tobacco consumption	current smoker
2251911000000114	Referral to smoking cessation service	current smoker
2462391000000119	Varenicline smoking cessation therapy declined	current smoker
2462431000000110	Varenicline smoking cessation therapy offered	current smoker
2717211000000112	Signposting to smoking cessation service	current smoker
2735181000000113	Ex-smoker amount unknown	ex smoker
2735201000000112	Ex-very heavy smoker (40+/day)	ex smoker
2735281000000119	Ex-heavy smoker (20-39/day)	ex smoker
2735331000000112	Ex-moderate smoker (10-19/day)	ex smoker
2735381000000111	Ex-light smoker (1-9/day)	ex smoker
2735421000000119	Ex-trivial smoker (<1/day)	ex smoker
2741221000000116	Referral for brief intervention for smoking cessation	current smoker
3374141000006117	Tolerant ex-smoker	ex smoker
3419101000006116	Moderate smoker (20 or less per day)	current smoker
3422221000006116	Heavy smoker (over 20 per day)	current smoker
3582451000006114	Cigarette smoking tobacco	current smoker
3874641000006110	Pipe smoking tobacco	current smoker
3959111000006111	Tobacco dependence syndrome	current smoker
4180331000006118	Tobacco use	current smoker
4939241000006115	Advice on effects of smoking on health	current smoker
4939251000006118	Advice on smoking	current smoker
4948551000006111	Drops unextinguished cigarettes	current smoker
4980561000006117	Time since stopped smoking	ex smoker

4980911000006113	Chews loose leaf tobacco	current smoker
5003141000006118	Trivial cigarette smoker	current smoker
5003151000006116	Light cigarette smoker	current smoker
5003161000006119	Moderate cigarette smoker	current smoker
5003171000006114	Heavy cigarette smoker	current smoker
5003181000006112	Very heavy cigarette smoker	current smoker
5003191000006110	Chain smoker	current smoker
5074201000006119	Smokers keratosis	current smoker
5495901000006112	Amount and type of tobacco smoked	current smoker
5495941000006114	Occasional smoker	current smoker
5495951000006111	Occasional cigarette smoker (less than one cigarette/day)	current smoker
6217151000006116	Intolerant ex-smoker	ex smoker
6217281000006116	Aggressive ex-smoker	ex smoker
6427931000006116	Smoking cessation assistance	current smoker
6718071000006115	Current non smoker but past smoking history unknown	never smoker
7368961000006112	Stopped smoking during pregnancy	ex smoker
7368971000006117	Stopped smoking before pregnancy	ex smoker
7375991000006118	Smokes tobacco daily	current smoker
7559591000006110	Tobacco use cessation education	current smoker
7569061000006118	Never used tobacco	never smoker
7650811000006116	Assessment of readiness for smoking cessation	current smoker
7696001000006118	Patient request for smoking cessation information	current smoker
8017571000006117	Ex-smoker for more than 1 year	ex smoker
8063181000006116	Wants to stop smoking	current smoker
8153371000006117	Occasional tobacco smoker	current smoker
8190531000006112	Referral to NHS (National Health Service) stop smoking service	current smoker
8196151000006112	Smoking cessation leaflet given	current smoker
11904991000006116	Occasional cigarette smoker	current smoker
14494021000006110	Former smoker	ex smoker
14933931000006118	Community Pharmacy Smoking Cessation Service	current smoker
342574011	Total time smoked	value>0 = yes
1484934016	Smoking status at 4 weeks	value>0 = yes
102921000006112	Tobacco smoking consumption	value>0 = yes
137421000006113	Smoke inhalation	value>0 = yes
137721000006115	Smoker - amount smoked	value>0 = yes
137751000006112	Smoking Age	value>0 = yes
137771000006119	Smoking Age Started	value>0 = yes
137811000006119	Smoking Status	value>0 = yes
1809131000006111	Total time smoked	value>0 = yes
4074571000006117	Cigarette smoke	value>0 = yes
4980581000006110	Age at starting smoking	value>0 = yes

Clinical codes for alcohol

Closest measure prior to cohort entry date was taken. GOLD: clinical, referral, tests; Aurum: consultations, observations. For Aurum, any values>0 linked with the additional codes (number of units) were coded as ‘Yes’. Values>150 were ignored.

CPRD GOLD: Alcohol			
**medcode**	**readcode**	**readterm**	**interpretation**
322	1364.00	Moderate drinker - 3-6u/day	Yes
385	1362.11	Drinks rarely	Yes
669	E250000	Nondependent alcohol abuse, unspecified	Yes
749	1362.12	Drinks occasionally	Yes
956	136J.00	Social drinker	Yes
967	1367.00	Stopped drinking alcohol	Ex
1399	E23..12	Alcohol problem drinking	Yes
1618	1365.00	Heavy drinker - 7-9u/day	Yes
2081	E23..11	Alcoholism	Yes
2082	E01y000	Alcohol withdrawal syndrome	Yes
2083	8BA8.00	alcohol detoxification	Yes
2084	E23..00	Alcohol dependence syndrome	Yes

2689	136G.00	Beer drinker	Yes
2925	F375.00	Alcoholic polyneuropathy	Yes
3216	J611.00	Acute alcoholic hepatitis	Yes
3782	E250.14	Intoxication - alcohol	Yes
4447	1361.12	Non-drinker alcohol	No
4500	E011000	Korsakov's alcoholic psychosis	Yes
4506	J153.00	Alcoholic gastritis	Yes
4743	J612.00	Alcoholic cirrhosis of liver	Yes
4915	G555.00	Alcoholic cardiomyopathy	Yes
5611	Eu10.00	[X]Mental and behavioural disorders due to use of alcohol	Yes
5740	E230.00	Acute alcoholic intoxication in alcoholism	Yes
5758	Eu10212	[X]Chronic alcoholism	Yes
6169	E23z.00	Alcohol dependence syndrome NOS	Yes
6467	Eu10511	[X]Alcoholic hallucinosis	Yes
7545	ZV4KC00	[V] Alcohol use	Yes
7602	J617000	Chronic alcoholic hepatitis	Yes
7746	E250.00	Nondependent alcohol abuse	Yes
7885	J613.00	Alcoholic liver damage unspecified	Yes
7943	J617.00	Alcoholic hepatitis	Yes
8030	ZV6D600	[V]Alcohol abuse counselling and surveillance	Yes
8363	G852300	Oesophageal varices in alcoholic cirrhosis of the liver	Yes
8388	ZV57A00	[V]Alcohol rehabilitation	Yes
8999	136P.00	Heavy drinker	Yes
9169	R103.00	[D]Alcohol blood level excessive	Yes
9489	9NN2.00	Under care of community alcohol team	Yes
9508	Eu10011	[X]Acute alcoholic drunkenness	Yes
9849	8H7p.00	Referral to community alcohol team	Yes
10161	2577.11	O/E - alcoholic breath	Yes
10458	ZV79100	[V]Screening for alcoholism	Yes
10691	J610.00	Alcoholic fatty liver	Yes
11106	E011100	Korsakov's alcoholic psychosis with peripheral neuritis	Yes
11670	Eu10611	[X]Korsakov's psychosis, alcohol induced	Yes
11740	9k1..00	Alcohol misuse - enhanced services administration	Yes
12271	E250.11	Drunkenness NOS	Yes
12353	Eu10500	[X]Mental & behav dis due to use alcohol: psychotic disorder	Yes
12949	1361.00	Teetotaller	No
12950	U800.00	[X]Eviden of alcohl involv blood alcohl level <20 mg/100 ml	Yes
12968	136H.00	Drinks beer and spirits	Yes
12969	136I.00	Drinks wine	Yes
12970	1361.11	Non drinker alcohol	No
12971	136F.00	Spirit drinker	Yes
12972	1363.00	Light drinker - 1-2u/day	Yes
12974	E250200	Nondependent alcohol abuse, episodic	Yes
12975	1362.00	Trivial drinker - <1u/day	Yes
12976	1369.00	Suspect alcohol abuse - denied	Yes
12977	1366.00	Very heavy drinker - >9u/day	Yes
12979	136M.00	Current non drinker	No
12980	136N.00	Light drinker	Yes
12982	136K.00	Alcohol intake above recommended sensible limits	Yes
12983	136E.00	Ex-very heavy drinker-(>9u/d)	Ex
12984	136Q.00	Very heavy drinker	Yes
12985	136O.00	Moderate drinker	Yes
16225	E010.00	Alcohol withdrawal delirium	Yes
16237	E01..00	Alcoholic psychoses	Yes
16587	ZV11311	[V]Problems related to lifestyle alcohol use	Yes
17259	Eu10411	[X]Delirium tremens, alcohol induced	Yes
17330	J613000	Alcoholic hepatic failure	Yes
17607	Eu10514	[X]Alcoholic psychosis NOS	Yes
17777	E250.13	Inebriety NOS	Yes
18636	E011200	Wernicke-Korsakov syndrome	Yes
19401	136R.00	Binge drinker	Yes
19493	136D.00	Ex-heavy drinker - (7-9u/day)	Ex
19494	136S.00	Hazardous alcohol use	Yes

19495	136C.00	Ex-moderate drinker - (3-6u/d)	Ex
20407	E014.11	Drunkenness - pathological	Yes
20514	Eu10300	[X]Mental and behav dis due to use alcohol: withdrawal state	Yes
20762	E011.00	Alcohol amnestic syndrome	Yes
21624	E230200	Episodic acute alcoholic intoxication in alcoholism	Yes
21650	8H35.00	Admitted to alcohol detoxification centre	Yes
21713	J612000	Alcoholic fibrosis and sclerosis of liver	Yes
21879	Eu10100	[X]Mental and behav dis due to use of alcohol: harmful use	Yes
22933	136A.00	Ex-trivial drinker (<1u/day)	Ex
23610	E250100	Nondependent alcohol abuse, continuous	Yes
23978	U81..00	[X]Evid of alcohol involv determind by level of intoxication	Yes
24064	E231100	Continuous chronic alcoholism	Yes
24485	E231300	Chronic alcoholism in remission	Ex
24735	2577	O/E - breath - alcohol smell	Yes
24984	J671000	Alcohol-induced chronic pancreatitis	Yes
25110	E013.00	Alcohol withdrawal hallucinosis	Yes
26106	E231200	Episodic chronic alcoholism	Yes
26323	Eu10711	[X]Alcoholic dementia NOS	Yes
26471	136B.00	Ex-light drinker - (1-2u/day)	Ex
26472	136L.00	Alcohol intake within recommended sensible limits	Yes
27342	E012.11	Alcoholic dementia NOS	Yes
27518	E250.12	Hangover (alcohol)	Yes
27670	L255300	Maternal care for (suspected) damage to fetus from alcohol	Yes
28150	E250z00	Nondependent alcohol abuse NOS	Yes
28780	Eu10211	[X]Alcohol addiction	Yes
29691	8G32.00	Aversion therapy - alcoholism	Yes
30162	Eu10513	[X]Alcoholic paranoia	Yes
30404	E015.00	Alcoholic paranoia	Yes
30460	Z4B1.00	Alcoholism counselling	Yes
30604	F25B.00	Alcohol-induced epilepsy	Yes
30695	136T.00	Harmful alcohol use	Yes
31443	E231.00	Chronic alcoholism	Yes
31569	E250300	Nondependent alcohol abuse in remission	Ex
31742	F394100	Alcoholic myopathy	Yes
32454	U806.00	[X]Eviden of alcoh involv blood alcoh level 120-199mg/100ml	Yes
32927	Eu10800	[X]Alcohol withdrawal-induced seizure	Yes
32964	66e0.00	alcohol abuse monitoring	Yes
33635	E231z00	Chronic alcoholism NOS	Yes
33670	E01y.00	Other alcoholic psychosis	Yes
33839	F144000	Cerebellar ataxia due to alcoholism	Yes
35330	9k11.00	Alcohol consumption counselling	Yes
36296	E230z00	Acute alcoholic intoxication in alcoholism NOS	Yes
36748	F11x011	Alcoholic encephalopathy	Yes
37264	8CE1.00	Alcohol leaflet given	Yes
37691	Eu10712	[X]Chronic alcoholic brain syndrome	Yes
37946	E012000	Chronic alcoholic brain syndrome	Yes
38061	1B1c.00	Alcohol induced hallucinations	Yes
39327	Eu10200	[X]Mental and behav dis due to use alcohol: dependence syndr	Yes
39726	U808.00	[X]Eviden alcoh involv blood alcoh level 240mg/100ml or more	Yes
39738	U813.00	[X]Evid alcoh invl determ by levl intox very sev alcoh intox	Yes
39799	Eu10600	[X]Mental and behav dis due to use alcohol: amnesic syndrome	Yes
40530	E230000	Acute alcoholic intoxication, unspecified, in alcoholism	Yes
40541	T900.00	Accidental poisoning by alcoholic beverages	Yes
41920	E011z00	Alcohol amnestic syndrome NOS	Yes
41983	Z191.00	Alcohol detoxification	Yes
42305	ZRk6.00	Severity of alcohol dependence questionnaire	Yes
43193	E231000	Unspecified chronic alcoholism	Yes
44019	U80..00	[X]Evidence of alcohl involv determin by blood alcohl level	Yes
44299	Eu10000	[X]Mental & behav dis due to use alcohol: acute intoxication	Yes
44686	U812.00	[X]Evid alcoh invol determ by level of intox sev alcoh intox	Yes
44783	1D19.00	Pain in lymph nodes after alcohol consumption	Yes
45169	Eu10y00	[X]Men & behav dis due to use alcohol: oth men & behav dis	Yes
46677	Z191100	alcohol withdrawal regime	Yes

47123	9k14.00	Alcohol counselling by other agencies	Yes
47555	F11x000	Cerebral degeneration due to alcoholism	Yes
47907	U807.00	[X]Eviden of alcoh involv blood alcoh level 200-239mg/100ml	Yes
50625	U814.00	[X]Evid alch invl detrm by levl intox alch invl not oth spec	Yes
53428	U804.00	[X]Eviden of alcohl involv blood alcohl level 80-99mg/100ml	Yes
54209	ZC2H.00	Advice to change alcohol intake	Yes
54504	U805.00	[X]Eviden of alcoh involv blood alcoh level 100-119mg/100ml	Yes
54505	E012.00	Other alcoholic dementia	Yes
56410	7P22100	Delivery of rehabilitation for alcohol addiction	Yes
56947	E230100	Continuous acute alcoholic intoxication in alcoholism	Yes
57202	U801.00	[X]Eviden of alcohl involv blood alcohl level 20-39mg/100ml	Yes
57242	U803.00	[X]Eviden of alcohl involv blood alcohl level 60-79mg/100ml	Yes
57714	E230.11	Alcohol dependence with acute alcoholic intoxication	Yes
57939	E014.00	Pathological alcohol intoxication	Yes
59079	U802.00	[X]Eviden of alcohl involv blood alcohl level 40-59mg/100ml	Yes
59574	E230300	Acute alcoholic intoxication in remission, in alcoholism	Ex
61383	Z191200	Planned reduction of alcohol consumption	Yes
62000	Eu10700	[X]Mental and behavioural disorders due to use of alcohol: residual and late-onset psychotic disorde	Yes
62299	ZRk9.11	SADD - Short alcohol dependence data	Yes
62300	ZRk9.00	Short alcohol dependence data	Yes
63529	9k12.00	Alcohol misuse - enhanced service completed	Yes
64101	Eu10400	[X]Men & behav dis due alcohl: withdrawl state with delirium	Yes
64389	Eu10z00	[X]Ment & behav dis due use alcohol: unsp ment & behav dis	Yes
64409	Z191400	Self-monitoring of alcohol intake	Yes
65754	C150500	Alcohol-induced pseudo-Cushing's syndrome	Yes
65932	Eu10512	[X]Alcoholic jealousy	Yes
66831	ZRk6.11	SADQ - Severity of alcohol dependence questionnaire	Yes
67651	E01z.00	Alcoholic psychosis NOS	Yes
68111	E01yz00	Other alcoholic psychosis NOS	Yes
73480	U811.00	[X]Evid alcoh invol determ by level of intox mod alcoh intox	Yes
84218	13ZY.00	Disqualified from driving due to excess alcohol	Yes
87505	E247.11	Absinthe addiction	Yes
90714	9k15.00	Alcohol screen - AUDIT completed	Yes
94485	9k18.00	Alcohol screen - AUDIT PC completed	Yes
94553	8HkG.00	Referral to specialist alcohol treatment service	Yes
94670	136W.00	Alcohol misuse	Yes
95181	Z191211	Alcohol reduction programme	Yes
96053	9k1A.00	Brief intervention for excessive alcohol consumptn completed	Yes
96054	9k1B.00	Extended intervention for excessive alcohol consumption completed	Yes
96993	8HkJ.00	Referral to alcohol brief intervention service	Yes
97126	136X.00	Alcohol units consumed on heaviest drinking day	Yes
97163	ZC22200	Advice to change alcoholic drink intake	Yes
97261	8IAF.00	brief intervention for excessive alcohol consumptn declined	Yes
97309	8CAv.00	advised to contact primary care alcohol worker	Yes
97680	8IAJ.00	declined referral to specialist alcohol treatment service	Yes
99877	136b.00	Feels should cut down drinking	Yes
101718	136Y.00	Drinks in morning to get rid of hangover	Yes
102247	8IAt.00	Extended intervention for excessive alcohol consumption declined	Yes
102448	136c.00	Higher risk drinking	Yes
102577	38Dz.00	Severity of alcohol dependence questionnaire	Yes
102665	136a.00	Increasing risk drinking	Yes
102770	38Dz.11	SADQ - Severity of alcohol dependence questionnaire	Yes
103230	136d.00	Lower risk drinking	Yes
103459	8IEA.00	Referral to community alcohol team declined	Yes
103698	U80z.00	[X]Evid alcoh invol detrm by pres alcoh in bld levl not spec	Yes
104611	J670800	Alcohol-induced acute pancreatitis	Yes
105273	U810.00	[X]Evid alcoh invol determ by levl of intox mild alcoh intox	Yes
108644	9NJz.00	In-house alcohol detoxification	Yes
109105	8W2..00	Refer to MH services deferred until alcohol misuse resolved	Yes
109241	8IH4.00	Alcohol Use Disorders Identification Test declined	Yes
109668	8CdK.00	Specialist alcohol treatment service signposted	Yes
109701	9Nz9.00	Emergency dept attendanc related to personl alcohl consumptn	Yes

109800	9NzA.00	Hospital attendance related to personal alcohol consumption	Yes
110494	8BAu.00	Alcohol harm reduction programme	Yes
115844	8BAw.00	Alcohol twelve step programme	Yes

CPRD Aurum: Alcohol		
**medcode**	**term**	**interpretation**
4525015	Alcoholic gastritis	Yes
12878014	Alcoholism	Yes
14103017	Alcohol-induced polyneuropathy	Yes
15243013	Alcohol withdrawal delirium	Yes
17390014	Acute alcoholic hepatitis	Yes
47085011	Social drinker	Yes
73012011	Moderate drinker	Yes
83834016	Alcoholic fatty liver	Yes
106878015	Alcohol detoxification	Yes
110629012	Chronic alcoholism	Yes
138527014	Alcoholic cardiomyopathy	Yes
144179014	Heavy drinker	Yes
216585018	Under care of community alcohol team	Yes
250312016	Light drinker - 1-2u/day	Yes
250313014	Moderate drinker - 3-6u/day	Yes
250314015	Heavy drinker - 7-9u/day	Yes
250316018	Very heavy drinker - >9u/day	Yes
250317010	Stopped drinking alcohol	Ex
250321015	Ex-trivial drinker (<1u/day)	Ex
250322010	Ex-light drinker - (1-2u/day)	Ex
250323017	Ex-moderate drinker - (3-6u/d)	Ex
250324011	Ex-heavy drinker - (7-9u/day)	Ex
250329018	Ex-very heavy drinker-(>9u/d)	Ex
250331010	Spirit drinker	Yes
250332015	Beer drinker	Yes
250333013	Drinks beer and spirits	Yes
250334019	Drinks wine	Yes
250335018	Alcohol intake above recommended sensible limits	Yes
250340014	Alcohol intake within recommended sensible limits	Yes
254305017	O/E - breath - alcohol smell	Yes
254306016	O/E - alcoholic breath	Yes
283028013	Alcohol leaflet given	Yes
283451018	Aversion therapy - alcoholism	Yes
283559019	Admitted to alcohol detoxification centre	Yes
291352018	Non-drinker alcohol	No
291355016	Teetotaller	No
294662017	Korsakov's alcoholic psychosis with peripheral neuritis	Yes
294664016	Alcohol amnestic syndrome NOS	Yes
294668018	Chronic alcoholic brain syndrome	Yes
294669014	Alcohol withdrawal hallucinosis	Yes
294670010	Drunkenness - pathological	Yes
294671014	Pathological alcohol intoxication	Yes
294672019	Alcoholic paranoia	Yes
294673012	Other alcoholic psychosis	Yes
294674018	Alcohol withdrawal syndrome	Yes
294675017	Other alcoholic psychosis NOS	Yes
294676016	Alcoholic psychosis NOS	Yes
295126010	Alcohol dependence with acute alcoholic intoxication	Yes
295127018	Acute alcoholic intoxication in alcoholism	Yes
295128011	Acute alcoholic intoxication, unspecified, in alcoholism	Yes
295129015	Continuous acute alcoholic intoxication in alcoholism	Yes
295130013	Episodic acute alcoholic intoxication in alcoholism	Yes
295131012	Acute alcoholic intoxication in remission, in alcoholism	Ex
295132017	Acute alcoholic intoxication in alcoholism NOS	Yes
295136019	Alcohol dependence	Yes
295139014	Continuous chronic alcoholism	Yes
295141010	Episodic chronic alcoholism	Yes
295142015	Chronic alcoholism in remission	Ex
295143013	Chronic alcoholism NOS	Yes
295144019	Alcohol dependence syndrome NOS	Yes

295251015	Nondependent alcohol abuse, unspecified	Yes
295252010	Nondependent alcohol abuse, continuous	Yes
295253017	Nondependent alcohol abuse, episodic	Yes
295254011	Nondependent alcohol abuse in remission	Ex
295255012	Nondependent alcohol abuse NOS	Yes
295765017	Alcohol-induced organic mental disorder	Yes
297011014	Alcoholic encephalopathy	Yes
303399018	Alcoholic liver damage	Yes
306615012	Maternal care for (suspected) damage to fetus from alcohol	Yes
328738016	Accidental poisoning by alcoholic beverages	Yes
342320016	Light drinker	Yes
342322012	Very heavy drinker	Yes
342361016	Drinks in morning to get rid of hangover	Yes
342366014	Binge drinker	Yes
342421016	Feels should cut down drinking	Yes
346929012	Alcoholic dementia	Yes
346930019	Inebriety NOS	Yes
346931015	Drunkenness	Yes
346934011	Absinthe addiction	Yes
353587018	Alcoholic hepatitis	Yes
353594015	Alcoholic fibrosis and sclerosis of liver	Yes
353595019	Alcoholic hepatic failure	Yes
353687012	Alcohol-induced acute pancreatitis	Yes
353698013	Alcohol-induced chronic pancreatitis	Yes
356292013	Alcohol-induced pseudo-Cushing's syndrome	Yes
397729013	Trivial drinker - <1u/day	Yes
401760017	Other alcoholic dementia	Yes
401797010	Nondependent alcohol abuse	Yes
409423019	Severity of alcohol dependence questionnaire score	Yes
409424013	SADQ - Severity of alcohol dependence questionnaire	Yes
451082013	[V] Alcohol use	Yes
451124014	Chronic alcoholic hepatitis	Yes
453265010	Oesophageal varices in alcoholic cirrhosis of the liver	Yes
459715013	Pain in lymph nodes after alcohol consumption	Yes
460926018	[V]Alcohol rehabilitation	Yes
461112017	Alcoholism counselling	Yes
461292011	Screening for alcohol abuse	Yes
478024019	Alcohol-induced epilepsy	Yes
478046010	Cerebellar ataxia due to alcoholism	Yes
500478011	Alcohol dependence syndrome	Yes
502017010	Alcohol problem drinking	Yes
1484890014	Referral to community alcohol team	Yes
2532939017	Alcohol consumption counselling	Yes
2534258017	Alcohol counselling by other agencies	Yes
2549849017	Alcohol induced hallucinations	Yes
117761000006116	Denies alcohol abuse	Yes
217461000000118	Non - drinker	No
223671000000110	Hangover from alcohol	Yes
226571000000116	Alcohol misuse enhanced service completed	Yes
236441000000113	Alcohol misuse enhanced services administration	Yes
299061000000117	Hazardous alcohol use	Yes
299081000000114	Harmful alcohol use	Yes
315091000000117	Alcohol withdrawal-induced seizure	Yes
362251000006117	Alcohol intoxication	Yes
362921000006118	[X]Alcohol addiction	Yes
362941000006113	[X]Alcoholic dementia NOS	Yes
362951000006110	Alcoholic hallucinosis	Yes
362961000006112	[X]Alcoholic jealousy	Yes
362971000006117	[X]Alcoholic paranoia	Yes
362981000006119	[X]Alcoholic psychosis NOS	Yes
370961000006119	[X]Chronic alcoholic brain syndrome	Yes
370971000006114	[X]Chronic alcoholism	Yes
371501000000110	Delivery of rehabilitation for alcohol addiction	Yes
376451000006113	[X]Delirium tremens, alcohol induced	Yes
380031000006116	[X]Evid of alcohol involv determind by level of intoxication	Yes
380081000006115	Finding of alcohol in blood	Yes

380091000006117	[X]Eviden of alcohl involv blood alcohl level 20-39mg/100ml	Yes
380101000006111	[X]Eviden of alcohl involv blood alcohl level 40-59mg/100ml	Yes
380111000006114	[X]Eviden of alcohl involv blood alcohl level 60-79mg/100ml	Yes
380121000006118	[X]Eviden of alcohl involv blood alcohl level 80-99mg/100ml	Yes
380131000006115	[X]Evidence of alcohl involv determin by blood alcohl level	Yes
397211000006118	[X]Men & behav dis due alcohl: withdrawl state with delirium	Yes
397241000006119	[X]Men & behav dis due to use alcohol: oth men & behav dis	Yes
397431000006111	[X]Ment & behav dis due use alcohol: unsp ment & behav dis	Yes
397611000006115	[X]Mental & behav dis due to use alcohol: acute intoxication	Yes
397621000006111	Alcohol-induced psychosis	Yes
397791000006118	Alcohol amnestic disorder	Yes
397801000006117	[X]Mental and behav dis due to use alcohol: dependence syndr	Yes
397811000006119	[X]Mental and behav dis due to use alcohol: withdrawal state	Yes
397881000006114	Alcohol abuse	Yes
405461000000114	Alcohol abuse monitoring	Yes
476361000006114	Alcohol amnestic syndrome	Yes
476611000006116	Alcoholic cirrhosis of liver	Yes
476701000006114	Alcohol myopathy	Yes
476741000006111	Alcohol-induced psychosis	Yes
480271000000113	Disqualified from driving due to excess alcohol	Yes
542611000006114	Cerebral degeneration due to alcoholism	Yes
587231000006111	Non drinker alcohol	No
606991000000117	Alcohol misuse	Yes
622961000000116	Referral to specialist alcohol treatment service	Yes
629811000006113	Occasional drinker	Yes
629821000006117	Drinks rarely	Yes
739501000000112	Advised to contact primary care alcohol worker	Yes
740641000000115	Referral to alcohol brief intervention service	Yes
750531000000113	Declined referral to specialist alcohol treatment service	Yes
750721000000118	Number of alcohol units consumed on heaviest drinking day	Yes
753331000006118	Korsakov alcoholic psychosis	Yes
769451000006111	Intoxication - alcohol	Yes
831091000006115	Referral to alcohol abuse team	Yes
831101000006114	Referral to alcohol counsellor	Yes
852151000006117	AUDIT score >5 alcohol dependence syndrome	Yes
852161000006115	AUDIT score >5 alcohol problem drinker	Yes
852381000006110	Alcohol reduction-maintain abstinence	Ex
852391000006113	Not interested in reducing alcohol	Yes
852401000006110	Ready to start reducing alcohol	Yes
852411000006113	Thinking about reducing alcohol	Yes
854651000006117	Alcohol overdose	Yes
855071000006115	Grade A none drinker or rare	No
882221000006114	Other alcoholic psychoses	Yes
882531000006110	Non-dependent abuse of alcohol	Yes
886501000006115	Alcoholic liver damage NOS	Yes
940781000006116	Evidence of alcohol withdrawal	Yes
961571000006111	Excessive use of alcohol	Yes
970391000006118	Substance misuse of Alcohol	Yes
970401000006116	Misuse of mixture of Alcohol	Yes
970411000006118	Misuse of Alcohol unspecified	Yes
983121000006115	Misuse free - alcohol free	No
1590881000006113	Alcohol screen - alcohol use disorder identification test completed	Yes
1590911000006113	Alcohol screen - alcohol use disorder identification test Piccinelli consumption questions completed	Yes
1675391000006112	Referral by community alcohol team	Yes
1680121000006110	Brief intervention for excessive alcohol consumption completed	Yes
1680131000006113	Extended intervention for excessive alcohol consumption completed	Yes
1704281000006117	Brief intervention for excessive alcohol consumption declined	Yes
1738341000000110	Increasing risk drinking	Yes
1738381000000119	Higher risk drinking	Yes
1738421000000111	Lower risk drinking	Yes
1745961000006111	Extended intervention for excessive alcohol consumption declined	Yes
1746481000000112	Referral to community alcohol team declined	Yes
1754191000006119	Extended intervention for excessive alcohol consumption declined	Yes
1779661000006118	Alcohol detoxification declined	Yes
1806211000006115	Unsuccessful attempts to reduce alcohol consumption	Yes

1808491000006113	Alcohol-related legal or disciplinary problem	Yes
1808611000006118	AREP - Alcohol rehabilitation education program	Yes
1878791000006112	In-house alcohol detoxification	Yes
1926131000006117	Referral to mental health services deferred until alcohol misuse resolved	Yes
1947501000006118	Emergency department attendance related to personal alcohol consumption	Yes
1972151000006110	Mental & behav dis due to use alcohol: acute intoxication, uncomplicated	Yes
1972161000006112	Mental & behav dis due to use alcohol: acute intoxication, with trauma or other bodily injury	Yes
1972271000006112	Mental & behav dis due to use alcohol: acute intoxication, with other medical complications	Yes
1973241000006113	Mental & behav dis due to use alcohol: acute intoxication, with delirium	Yes
1973321000006113	Mental & behav dis due to use alcohol: acute intoxication, with perceptual distortions	Yes
1973411000006110	Mental & behav dis due to use alcohol: acute intoxication, with coma	Yes
1973441000006114	Mental & behav dis due to use alcohol: acute intoxication, with convulsions	Yes
1973481000006115	Mental & behav dis due to use alcohol: acute intoxication, pathological intoxication	Yes
1973701000006119	Mental and behav dis due to use alcohol: dependence syndr, currently abstinent	Ex
1973811000006113	Mental and behav dis due to use alcohol: dependence syndr, currently abstinent, but in a protected environment	Ex
1973871000006116	Mental and behav dis due to use alcohol: dependence syndr, currently on a clinically supervised maintenance or replacement regime [controlled dependence]	Yes
1974021000006112	Mental and behav dis due to use alcohol: dependence syndr, currently abstinent, but receiving treatment with aversive or blocking drugs	Ex
1974071000006113	Mental and behav dis due to use alcohol: dependence syndr, currently using the substance [active dependence]	Yes
1974111000006117	Mental and behav dis due to use alcohol: dependence syndr, continuous use	Yes
1974141000006118	Mental and behav dis due to use alcohol: dependence syndr, episodic use [dipsomania]	Yes
1974191000006110	Mental and behav dis due to use alcohol: withdrawal state, uncomplicated	Yes
1974231000006117	Mental and behav dis due to use alcohol: withdrawal state, with convulsions	Yes
1974301000006117	Mental & behav dis due to use alcohol: withdrawl state with delirium, without convulsions	Yes
1974521000006117	Mental & behav dis due to use alcohol: withdrawl state with delirium, with convulsions	Yes
1975141000006117	Mental & behav dis due to use alcohol: psychotic disorder, schizophrenia-like	Yes
1975181000006111	Mental & behav dis due to use alcohol: psychotic disorder, predominantly delusional	Yes
1975291000006117	Mental & behav dis due to use alcohol: psychotic disorder, predominantly hallucinatory	Yes
1975341000006119	Mental & behav dis due to use alcohol: psychotic disorder, predominantly polymorphic	Yes
1975371000006110	Mental & behav dis due to use alcohol: psychotic disorder, predominantly depressive symptoms	Yes
1975411000006111	Mental & behav dis due to use alcohol: psychotic disorder, predominantly manic symptoms	Yes
1975461000006114	Mental & behav dis due to use alcohol: psychotic disorder, mixed	Yes
1975511000006112	Mental & behav dis due to use alcohol: resid & late-onset psychot dis, flashbacks	Yes
1975541000006111	Mental & behav dis due to use alcohol: resid & late-onset psychot dis, personality or behaviour disorder	Yes
1975591000006119	Mental & behav dis due to use alcohol: resid & late-onset psychot dis, dementia	Yes
1975661000006111	Mental & behav dis due to use alcohol: resid & late-onset psychot dis, residual affective disorder	Yes
1976071000006119	Mental & behav dis due to use alcohol: resid & late-onset psychot dis, other persisting cognitive impairment	Yes
1976111000006110	Mental & behav dis due to use alcohol: resid & late-onset psychot dis, late-onset psychotic disorder	Yes
2298341000000114	In-house alcohol detoxification	Yes
2367071000000110	Alcohol Use Disorders Identification Test declined	Yes
2419601000000110	Specialist alcohol treatment service signposted	Yes
2454281000000112	Hospital attendance related to personal alcohol consumption	Yes
2462911000000112	Withdrawn from alcohol detoxification programme	Yes
2475831000000114	Alcohol harm reduction programme	Yes
2485391000000114	Alcohol twelve step programme	Yes
2502061000006114	Current drinker	Yes
2502071000006119	Alcohol user	Yes
2502081000006116	Drinks alcohol	Yes
2502091000006118	Current drinker of alcohol	Yes
2612481000006116	Alcohol hallucinosis	Yes
2614651000006115	Alcoholism	Yes
2626141000006112	Alcoholic polyneuropathy	Yes
2626161000006111	Alcoholic peripheral neuropathy	Yes

2658601000006116	Acute alcoholic liver disease	Yes
2716771000000111	Signposting to alcohol misuse service	Yes
2740361000006119	AA - Alcohol abuse	Ex
2740831000000114	Harmful use of alcohol	Yes
2796011000006111	Alcohol intoxication delirium	Yes
2819381000006112	Alcohol rehabilitation and detoxification	Yes
2881581000006119	Combined alcohol and drug rehabilitation and detoxification	Yes
2911271000006113	Acute alcoholism	Yes
2911291000006114	Acute alcohol intoxication	Yes
2950191000006118	Smell of alcohol on breath	Yes
2969641000006116	Alcohol-related disorder	Yes
2998891000006117	Drinking	Yes
3073921000006112	Alcohol rehabilitation	Yes
3122331000006110	Patient referral for alcoholism rehabilitation	Yes
3165681000006118	Alcoholic liver disease	Yes
3165691000006115	ALD - Alcoholic liver disease	Yes
3182591000006114	Alcohol induced psychosis	Yes
3315041000006111	Alcoholic steatosis	Yes
3315061000006110	Alcoholic fatty liver disease	Yes
3360071000006111	Alcohol	Yes
3402361000006113	Alcoholic ketoacidosis	Yes
3510721000006113	Combined alcohol and drug rehabilitation	Yes
3545831000006115	Detoxication psychiatric therapy for alcoholism	Yes
3545841000006113	Detoxication therapy for alcoholism	Yes
3630141000006115	Korsakov syndrome - alcoholic	Yes
3630161000006116	Amnesic syndrome due to alcohol	Yes
3630171000006111	Alcoholic amnestic syndrome	Yes
3843491000006118	Ex-drinker	Ex
3846821000006114	Alcohol poisoning	Yes
3859091000006114	Dilated cardiomyopathy secondary to alcohol	Yes
3859111000006117	Dilated cardiomyopathy caused by alcohol	Yes
3890681000006118	Uncomplicated alcohol withdrawal	Yes
3913321000006111	Drinks heavily	Yes
3916281000006114	Combined alcohol and drug detoxification	Yes
4064221000006110	Drug interaction with alcohol	Yes
4064231000006113	Alcohol interaction with drug	Yes
4120341000006116	Never drinks	No
4120351000006119	Does not drink alcohol	No
4120371000006112	Non - drinker alcohol	No
4120381000006110	Current non-drinker of alcohol	No
4120391000006113	Non-drinker for personal reasons	No
4533231000006117	Very heavy drinker - greater than 9 units/day	Yes
4533271000006119	Ex-trivial drinker - less than 1 unit/day	Yes
4533331000006113	Ex-very heavy drinker - greater than 9 units/day	Yes
4533441000006111	XS - Excessive alcohol consumption	Yes
4533451000006113	Excessive alcohol consumption	Yes
4533461000006110	Excessive alcohol use	Yes
4977911000006115	Lifetime non-drinker	No
4977931000006114	Lifetime teetotaller	No
4977971000006112	Drinks on special occasions	Yes
4977991000006113	Fairly heavy drinker	Yes
4978011000006117	Secret drinker	Yes
4978031000006111	Problem drinker	Yes
4978041000006118	Alcoholic	Yes
4978061000006119	Dependent drinker	Yes
4978121000006114	Drinks at home	Yes
4978131000006112	Home drinker	Yes
4978141000006119	Drinks at other people's homes	Yes
4978151000006117	Drinks in public places	Yes
4978171000006110	Street drinker	Yes
4978201000006114	Drinks in public houses	Yes
4978241000006111	Company when drinking	Yes
4978251000006113	Drinks alone	Yes
4978281000006117	Drinks in company	Yes
4978321000006111	Drinks with friends	Yes
4978411000006113	Drinks alcohol evenly through week	Yes

4978421000006117	Drinks alcohol unevenly through week	Yes
4978431000006119	Weekend drinker	Yes
4978461000006111	Episodic drinker	Yes
4978471000006116	Alcoholic binges exceeding sensible amounts	Yes
4978481000006118	Alcoholic binges exceeding safe amounts	Yes
4978491000006115	Daily drinker	Yes
4978501000006111	Drinks alcohol daily	Yes
4978511000006114	Habitual drinker	Yes
4978531000006115	Drinking episode	Yes
4978541000006113	Drinking bout	Yes
4978551000006110	Drinking session	Yes
4978561000006112	Heavy drinking session	Yes
4978571000006117	Drinking binge	Yes
4978581000006119	Alcohol binge	Yes
4978591000006116	Binge drinking	Yes
4978651000006111	Priority of drink-related activities	Yes
4978661000006113	Salience of drink-related activities	Yes
4978681000006115	Undue priority given to drink-related activities	Yes
4978731000006113	Ability to control drinking	Yes
4978751000006118	Able to control drinking	Yes
4978761000006116	Unable to control drinking	Yes
4978781000006114	Unable to control drinking once started	Yes
4978801000006113	Unable to stop drinking before intoxication	Yes
4978811000006111	Use of stratagems to control drinking	Yes
4978821000006115	Has no stratagem to control drinking	Yes
4978831000006117	Uses stratagems to control drinking	Yes
4978851000006112	Tolerance to alcohol	Yes
4978881000006116	Physical tolerance to alcohol	Yes
4978891000006118	Psychological tolerance to alcohol	Yes
4978931000006110	Charged with drink driving offence	Yes
4978951000006115	Convicted of drink driving	Yes
4978971000006113	Persistent effect of alcohol	Yes
4978991000006114	Feels effect of alcohol at work	Yes
4979011000006112	Attitude to drinking	Yes
4979021000006116	Ashamed of drinking	Yes
4979041000006111	Feels drinking is out of control	Yes
4979051000006113	Feels afraid of being an alcoholic	Yes
4979061000006110	Craving for alcohol	Yes
5063221000006116	Alcohol-related macrocytosis	Yes
5110231000006116	Alcohol-induced hypoglycaemia	Yes
5110241000006114	Alcohol-induced hypoglycemia	Yes
5689771000006115	Persistent alcohol abuse	Yes
5689781000006117	Chronic alcohol abuse	Yes
5886961000006112	Abstinent alcoholic	Ex
5887581000006113	Alcohol-induced cerebellar ataxia	Yes
5980911000006115	Alcohol-induced flushing	Yes
5981291000006110	Alcohol withdrawal-induced convulsion	Yes
6047161000006116	Alcohol related optic neuropathy	Yes
6221451000006112	Alcohol-related fit	Yes
6221531000006118	Alcoholic cerebellar degeneration	Yes
6221541000006111	Alcoholic cerebellar degeneration syndrome	Yes
6455201000006114	Substance use treatment: alcohol withdrawal	Yes
6769881000006115	Alcohol abuse control	Yes
6769891000006117	Alcohol abuse prevention assessment	Yes
6769921000006111	Alcohol abuse prevention education	Yes
6881361000006118	Suspected alcohol abuse	Yes
6947461000006115	Alcoholic cirrhosis	Yes
6947471000006110	Alcoholic liver cirrhosis	Yes
7084901000006118	Alcohol intake within recommended daily limit	Yes
7108471000006117	Alcohol intake exceeds recommended daily limit	Yes
7229341000006119	Self-monitoring of alcohol intake	Yes
7240391000006119	Alcohol abuse cessation behavior	Yes
7264381000006117	Referral to specialist alcohol treatment service declined by patient	Yes
7267751000006119	High alcohol level in blood	Yes
7275011000006117	AUDIT (alcohol use disorders identification test) score	Yes
7309181000006113	Alcohol-induced pancreatitis	Yes

7309201000006114	Alcoholic pancreatitis	Yes
7334601000006116	Drinks alcoholic cider	Yes
7518451000006115	Thrombocytopaenia due to alcohol	Yes
7587641000006118	Admits alcohol use	Yes
7613321000006119	Alcohol reduction programme	Yes
7613331000006116	Alcohol reduction program	Yes
7700651000006119	Chronic alcoholic liver disease	Yes
7703061000006115	Acute on chronic alcoholic liver disease	Yes
7706231000006114	Mild alcohol dependence	Yes
7710581000006113	Severe alcohol dependence	Yes
7725141000006111	Moderate alcohol dependence	Yes
7785371000006113	Withdrawn from alcohol detoxification program	Yes
7791901000006117	Alcohol induced disorder	Yes
7821631000006114	Fibrosis of liver caused by alcohol	Yes
7821641000006116	Alcoholic fibrosis of liver	Yes
7858041000006119	Alcohol-related neurological disorder	Yes
7967731000006110	Decreased alcohol consumption	Yes
8048151000006113	Chronic pancreatitis due to chronic alcoholism	Yes
8088741000006119	Alcohol induced disorder co-occurrent and due to alcohol dependence	Yes
8088751000006117	Alcohol dependence with alcohol induced disorder	Yes
8103541000006113	Feels alcohol consumption is not a problem	Yes
8106921000006119	Feels it is not necessary to reduce alcohol consumption	Yes
8121241000006115	Seen by counsellor for alcohol misuse	Yes
8229891000006112	Drinks real ale	Yes
8259601000006117	Referral to alcohol misuse clinic	Yes
8364771000006117	Injury following alcohol use	Yes
8458711000006115	Ascites due to alcoholic cirrhosis	Yes
9344021000006112	Unhealthy alcohol drinking behaviour	Yes
9344031000006110	Unhealthy alcohol drinking behavior	Yes
12031691000006112	Alcoholic steatohepatitis	Yes
12112021000006112	Management of alcohol intake	Yes
12117371000006112	Referral to alcohol misuse service	Yes
12456651000006116	[V]Alcohol rehabilitation	Yes
13628811000006110	Lifetime non-drinker of alcohol	No
13770661000006116	Referral to alcoholism rehabilitation service	Yes
13996301000006112	Seen by community alcohol team	Yes
14146421000006116	Oesophageal varices due to cirrhosis of liver caused by alcohol	Yes
14447031000006112	Acute alcohol intoxication	Yes
14798841000006110	Alcohol-induced psychosis	Yes
14798871000006120	Alcoholic cardiomyopathy	Yes
14798891000006118	Dilated cardiomyopathy caused by alcohol	Yes
14799161000006114	Cognitive impairment caused by alcohol	Yes
14799371000006116	Alcohol-induced organic mental disorder	Yes
408548014	Alcohol use disorders identification test score	Value>0 = Yes
1221271018	Alcohol consumption	Value>0 = Yes
2532935011	Alcohol questionnaire completed	Value>0 = Yes
240741000006114	Education about alcohol consumption	Value>0 = Yes
476411000006118	Alcohol consumption NOS	Value>0 = Yes
476421000006114	Alcohol consumption screen	Value>0 = Yes
476431000006112	Alcohol consumption screening	Value>0 = Yes
492591000000114	Alcohol screen - fast alcohol screening test completed	Value>0 = Yes
538891000000111	FAST (Fast Alcohol Screening Test) score	Value>0 = Yes
556651000000116	Alcohol units consumed per week	Value>0 = Yes
622791000000112	Single alcohol screening questionnaire	Value>0 = Yes
936001000006111	Advice relating to alcohol consumption	Value>0 = Yes
940771000006119	Alcohol consumption in the week before custody	Value>0 = Yes
1127841000000113	Breath alcohol level	Value>0 = Yes
1659811000006114	Alcohol Use Disorders Identification Test - Piccinelli Consumption score	Value>0 = Yes
1660411000006112	AUDIT-C (Alcohol Use Disorders Identification Test - Consumption) score	Value>0 = Yes
1899411000006110	AUDIT score - frequency of drinking alcohol	Value>0 = Yes
1899421000006119	AUDIT score - units of alcohol drunk on a	Value>0 = Yes
1899461000006113	AUDIT score - freq needs morning alcoholic drink in	Value>0 = Yes
1899511000006111	AUDIT-C score - frequency of drinking alcohol	Value>0 = Yes
1899521000006115	AUDIT-C score - units of alcohol drunk on a	Value>0 = Yes
4533141000006118	Alcohol intake	Value>0 = Yes
4987151000006111	alcohol units/day	Value>0 = Yes

4987161000006113	alcohol units/week	Value>0 = Yes
8458781000006110	Alcohol units consumed per day	Value>0 = Yes

Clinical codes for body mass index (BMI)

Closest measure prior to cohort entry date was taken. GOLD: clinical, referral, tests; Aurum: consultations, observations. Where there were no BMI measurements, BMI was derived from height (converted into metres) and weight (kilograms).

The below codes were used. Additional codes were also searched (also see below) and height and weight were derived using the 'values' variable. The cleaning algorithm was based on Bhaskaran et al. (2013)*. Weights were ignored if <25.4kg (<4 stone) and ≥222 kg (35 stone); heights were ignored if <4ft or >7ft, BMI <10 and BMI >80 ignored.

HES data were also searched and ICD-10 code E66 ("obesity") was used to categorise obesity.

CPRD GOLD: BMI		
**medcode**	**readcode**	**readterm**
**UNDERWEIGHT**		
**BMI measurement <18.5 kg/m2**		
126	22A6.00	O/E - Underweight
12530	R034800	[D]Underweight
**NORMAL WEIGHT**		
**BMI measurement 18.5–25 kg/m2**		
28946	22K1.00	Body Mass Index normal K/M2
44291	22K8.00	Body mass index 20-24 - normal
**OVERWEIGHT**		
**BMI measurement 25–29 kg/m2**		
9015	22K4.00	Body mass index index 25-29 - overweight
2839	22A4.11	O/E - overweight
103499	22AA.00	Overweight
**OBESE**		
**BMI measurement 30–39 kg/m2**		
59780	222A.00	O/E - obese
7984	22A5.11	O/E - obese
28937	22K2.00	Body Mass Index high K/M2
13278	22K5.00	Body mass index 30+ - obesity
108694	22KC.00	Obese class I (body mass index 30.0 - 34.9)
108610	22KD.00	Obese class II (body mass index 35.0 - 39.9)
11461	66C..00	Obesity monitoring
38658	66C1.00	Initial obesity assessment
29538	66C2.00	Follow-up obesity assessment
3176	66C4.00	Has seen dietician - obesity
38632	66C6.00	Treatment of obesity started
110196	66Ce.00	Telehealth obesity monitoring
104724	66CL.00	Risk health associa overweight obesity, at no increased risk
102150	66CM.00	Risk health associ overweight and obesity, at increased risk
106010	66CP.00	Risk health associ overweight and obesity, at very high risk
104887	66CS.00	Inter risk hlth overwght obesity adv diet phys act cons drug
101800	66CT.00	Inter risk hlth owt ob adv diet phy ac cons dgs cons surgery
110415	66CX.00	Obesity multidisciplinary case review
40153	66CZ.00	Obesity monitoring NOS
32843	9OK..00	Obesity monitoring admin.
21744	9OK..11	Obesity clinic administration
52036	9OK3.00	Obesity monitoring default
49409	9OK4.00	Obesity monitoring 1st letter
55586	9OK5.00	Obesity monitoring 2nd letter
55585	9OK6.00	Obesity monitoring 3rd letter
70950	9OK7.00	Obesity monitoring verbal inv.
67517	9OK8.00	Obesity monitor phone invite
47439	9OKA.00	Obesity monitoring check done
52735	9OKZ.00	Obesity monitoring admin.NOS
66406	C38..00	Obesity and other hyperalimentation
430	C380.00	Obesity

38799	C380000	Obesity due to excess calories
49250	C380100	Drug-induced obesity
38059	C380200	Extreme obesity with alveolar hypoventilation
8854	C380300	Morbid obesity
22695	C380400	Central obesity
25968	C380500	Generalised obesity
104129	C380600	Adult-onset obesity
104421	C380700	Lifelong obesity
24755	C38y.11	Pickwickian syndrome
38294	C38y000	Pickwickian syndrome
103574	C38y011	Obesity hypoventilation syndrome
70898	C38z.00	Obesity and other hyperalimentation NOS
101217	66CR.00	Int risk health ass overwt ob advice about diet physical act
102514	66CN.00	Risk health associated overweight and obesity, at high risk
108147	8T11.00	Referral to multidisciplinary obesity clinic
52782	Cyu7.00	[X]Obesity and other hyperalimentation
11401	C38z000	Simple obesity NOS
**SEVERELY OBESE (coded as obese)**		
**BMI measurement 40+ kg/m2**		
108478	22KE.00	Obese class III (BMI equal to or greater than 40.0)
22556	22K7.00	Body mass index 40+ - severely obese

**CPRD Aurum: BMI**	
**medcode**	**term**
**UNDERWEIGHT**	
**BMI measurement <18.5 kg/m2**	
253687013	O/E - Underweight
2159952010	Underweight
1808061000006114	Body mass index less than 18.5
910261000006112	[RFC] Underweight
5256251000006115	Patient underweight
7068841000006116	Body mass index less than 16.5
**NORMAL WEIGHT**	
**BMI measurement 18.5–25 kg/m2**	
253844013	Normal body mass index
1808071000006119	Body mass index 18.5-24.9
2474325012	Body mass index 20-24 - normal
3070611000006119	Normal BMI (body mass index)
**OVERWEIGHT**	
**BMI measurement 25–29 kg/m2**	
253847018	Body mass index index 25-29 - overweight
411922013	O/E - overweight
4553701000006115	Body mass index 25-29 - overweight
4553721000006113	BMI 25-29 - overweight
5117851000006111	Patient overweight
910251000006110	[RFC] Overweight
356960015	Overweight
7833991000006118	Overweight in adulthood with body mass index of 25 or more but less than 30
**OBESE**	
**BMI measurement 30–39 kg/m2**	
280881000006115	O/E - obese
4553741000006118	BMI 30+ - obesity
4636861000006111	Has seen dietitian - obesity
253576010	O/E - obese
5999801000006118	Obesity monitoring invitation
253845014	Increased body mass index
253848011	Body mass index 30+ - obesity
2350241000000116	Obese class I (body mass index 30.0 - 34.9)
2350261000000115	Obese class II (body mass index 35.0 - 39.9)
2865221000006115	Impaired glucose tolerance in obese
401534016	Obesity monitoring
264752011	Initial obesity assessment
264753018	Follow-up obesity assessment
264756014	Has seen dietician - obesity

264758010	Treatment of obesity started
2423851000000119	Telehealth obesity monitoring
1753271000006112	Risk to health associated with overweight and obesity, at no increased risk
1753281000006110	Risk to health associated with overweight and obesity, at increased risk
1753291000006113	Risk to health associated with overweight and obesity, at high risk
1753301000006114	Risk to health associated with overweight and obesity, at very high risk
1753311000006112	Intervention for risk to health associated with overweight and obesity, general advice on healthy weight and lifestyle
1753321000006116	Intervention for risk to health associated with overweight and obesity, advice about diet and physical activity
1753331000006118	Intervention for risk to health associated with overweight and obesity, advice about diet and physical activity, consider drugs
1753341000006111	Intervention for risk to health associated with overweight and obesity, advice about diet and physical activity, consider drugs, consider surgery
2310101000000115	Obesity multidisciplinary case review
264769013	Obesity monitoring NOS
2303211000000117	Referral to multidisciplinary obesity clinic
21621000000119	Obesity monitoring administration
26111000000114	Obesity clinic administration
285741017	Attends obesity monitoring
285743019	Obesity monitoring default
270301000006112	Obesity monitoring first letter
270311000006110	Obesity monitoring second letter
270321000006119	Obesity monitoring third letter
270381000006115	Obesity monitoring verbal invite
270281000006113	Obesity monitoring telephone invite
285750015	Obesity monitoring check done
16131000000113	Obesity monitoring admin.NOS
293631011	Obesity and other hyperalimentation
881831000006116	Hyperalimentation incl.obesity
2535065012	Obesity
2537960015	Obesity due to excess calories
293634015	Drug-induced obesity
293635019	Extreme obesity with alveolar hypoventilation
356968010	Morbid obesity
370698013	Central obesity
356966014	Generalised obesity
436946011	Adult-onset obesity
434770018	Lifelong obesity
232631000006113	Pickwickian syndrome
2535848016	Pickwickian syndrome
1803041000000114	Obesity hypoventilation syndrome
451432013	Simple obesity
293802014	[X]Obesity and other hyperalimentation
6866991000006116	Obese
857321000006113	Moderately obese
857911000006118	Very obese
907161000006115	[RFC] Obesity
7573871000006112	Lymphoedema associated with obesity
7758811000006114	Obesity due to melanocortin 4 receptor deficiency
8024931000006112	Severe obesity
12008191000006116	Obesity care
12113161000006112	Obese class I
12113181000006116	Obese class II
13926671000006120	Obese class III
**SEVERELY OBESE (coded as obese)**	
**BMI measurement 40+ kg/m2**	
1900331000006113	Obese class III (body mass index equal to or greater than 40.0)
2160062010	Body mass index 40+ - severely obese
6763391000006114	Body mass index 40+ - morbidly obese
6763401000006111	BMI (body mass index) 40+ - severely obese

CPRD Aurum: BMI codes with values associated with them

medcode	term	interpretation

100716012	Body mass index	BMI value
252191014	Weight increasing	Weight value
252192019	Weight decreasing	Weight value
253669010	Standing height	Height value
253676017	O/E - height NOS	Height value
253677014	Body weight	Weight value
253688015	O/E - weight NOS	Weight value
253866018	Height and weight	Height/weight value
451201014	Weight monitoring	Weight value
1780175010	Baseline weight	Weight value
3514400010	Current body weight	Weight value
59281000006111	Weight screening	Weight value
59311000006113	Weight symptom	Weight value
923861000006112	Body mass index	BMI value
1910911000006118	Reported weight	Weight value
1910921000006114	Estimated height	Height value
1910931000006112	Reported height	Height value
2196071000000116	Baseline body mass index	BMI value
2934311000006118	Weight	Weight value
3315711000006110	Body height measure	Height value
3315721000006119	Body height	Height value
3484801000006114	BMI - Body mass index	BMI value
6251151000006116	Body weight measure	Weight value
7879461000006116	Weight	Weight value
8286991000006119	Baseline BMI (body mass index)	BMI value

* Bhaskaran K, Forbes HJ, Douglas I, Leon DA, Smeeth L. Representativeness and optimal use of body mass index (BMI) in the UK Clinical Practice Research Datalink (CPRD). BMJ Open. 2013 Sep 13;3(9):e003389

Figure S1: Data flow diagram

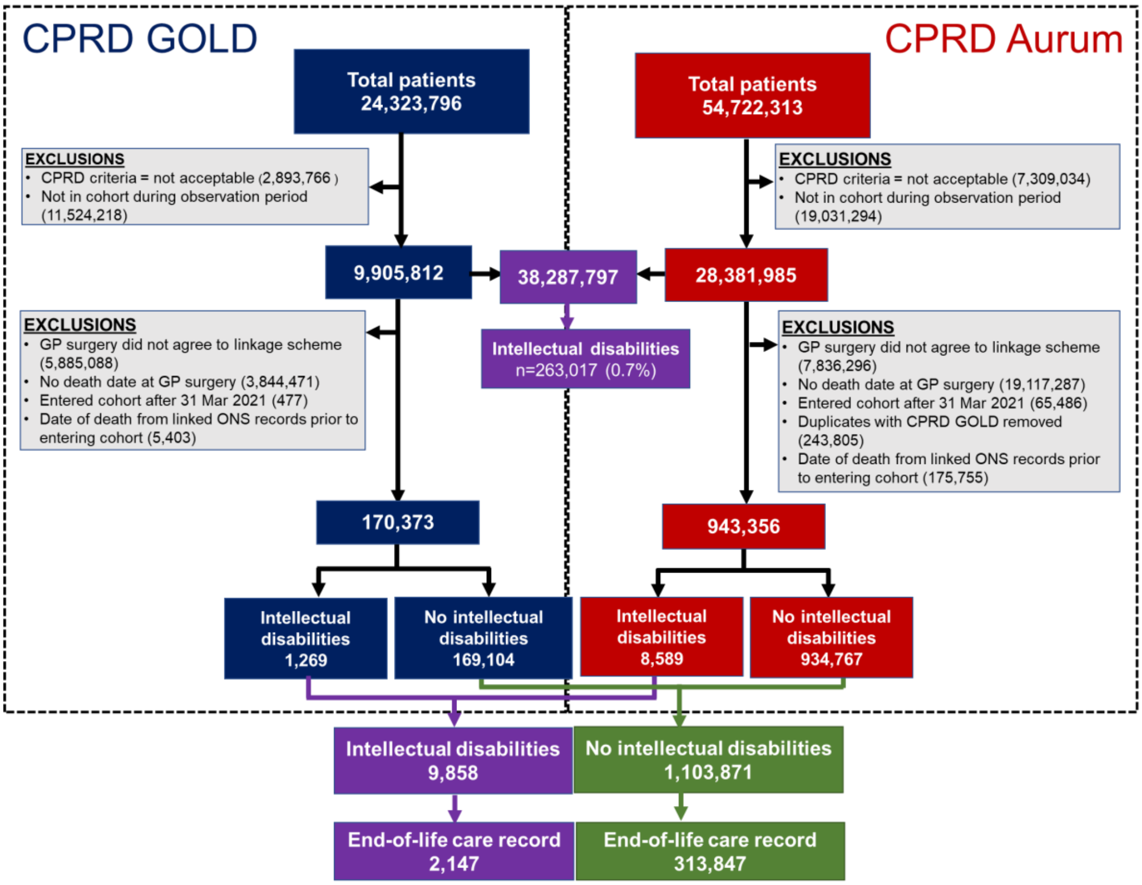

Note: 191 patients with intellectual disability with a first intellectual disability diagnosis after their first end-of-life care record were excluded to avoid immortal time bias*. These individuals form part of the second exclusion (i.e., not in the cohort during the observation period; <0.01% of exclusions in this category)

* Tyrer F, Bhaskaran K, Rutherford MJ. Immortal time bias for life-long conditions in retrospective observational studies using electronic health records. BMC Med Res Methodol. 2022 Mar 27;22(1):86. doi: 10.1186/s12874-022-01581-1.

Figure S2: Unadjusted survival (days) after first EOLC record by intellectual disability status

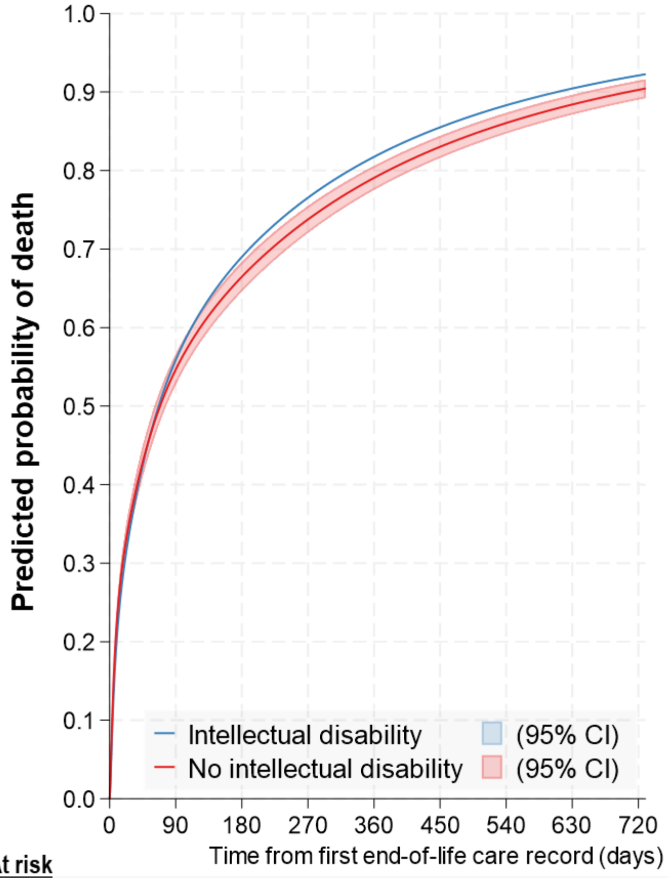

At risk									
ID	2147	975	711	571	460	354	298	252	208
No ID	313847	139157	97629	73613	57260	45479	36763	30218	24839

Figure S3: Crude rates of healthcare utilisation (primary care consultations, referrals and hospitalisations by month)*

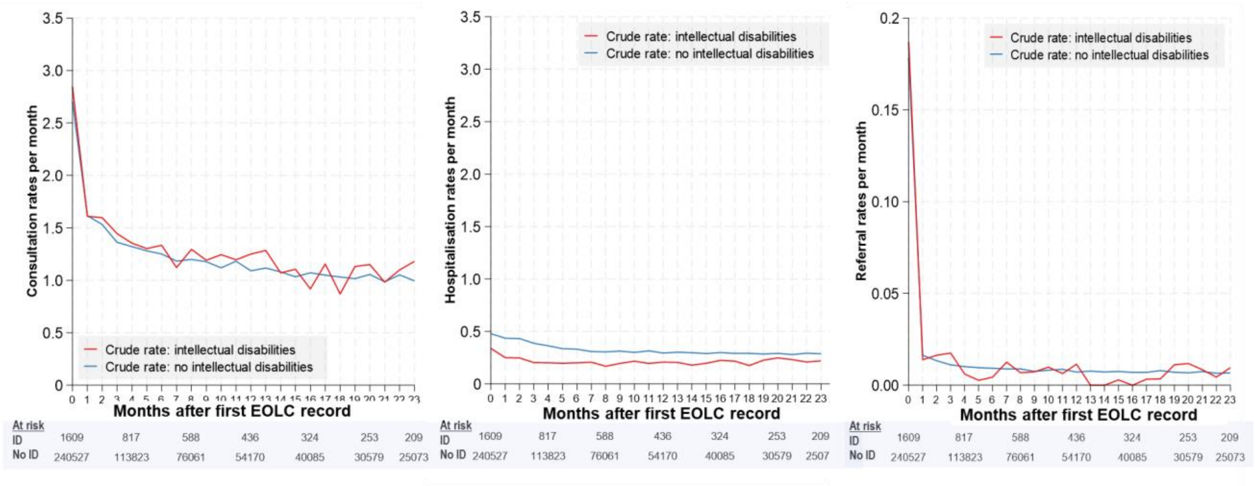

* Please note y-axis change for referral rates
